# An air- and moisture-stable ruthenium precatalyst for diverse reactivity

**DOI:** 10.1038/s41557-024-01481-5

**Published:** 2024-04-03

**Authors:** Gillian McArthur, Jamie H. Docherty, Mishra Deepak Hareram, Marco Simonetti, Iñigo J. Vitorica-Yrezabal, James J. Douglas, Igor Larrosa

**Affiliations:** 1 Department of Chemistry, University of Manchester, Manchester, UK; 2 Department of Chemistry, Lancaster University, Lancaster, UK; 3 bp, Low Carbon Innovation Centre, Saltend Chemicals Park, Hull, UK; 4 Early Chemical Development, Pharmaceutical Sciences, R&D, AstraZeneca, Macclesfield, UK

**Keywords:** Catalyst synthesis, Synthetic chemistry methodology, Homogeneous catalysis, Synthetic chemistry methodology

## Abstract

Versatile, efficient and robust (pre)catalysts are pivotal in accelerating the discovery and optimization of chemical reactions, shaping diverse synthetic fields such as cross-coupling, C–H functionalization and polymer chemistry. Yet, their scarcity in certain domains has hindered the advancement and adoption of new applications. Here we present a highly reactive air- and moisture-stable ruthenium precatalyst [(^*t*^BuCN)_5_Ru(H_2_O)](BF_4_)_2_, featuring a key exchangeable water ligand. This versatile precatalyst drives an array of transformations, including late-stage C(*sp*^2^)–H arylation, primary/secondary alkylation, methylation, hydrogen/deuterium exchange, C(*sp*^3^)–H oxidation, alkene isomerization and oxidative cleavage, consistently outperforming conventionally used ruthenium (pre)catalysts. The generality and applicability of this precatalyst is exemplified through the potential for rapid screening and optimization of photocatalytic reactions with a suite of in situ generated ruthenium photocatalysts containing hitherto unknown complexes, and through the rapid discovery of reactivities previously unreported for ruthenium. The diverse applicability observed is suggestive of a generic platform for reaction simplification and accelerated synthetic discovery that will enable broader applicability and accessibility to state-of-the-art ruthenium catalysis.

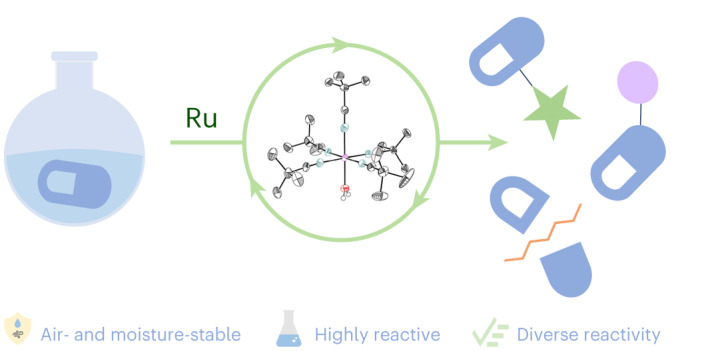

## Abstract (web summary)

Despite the widespread utility of ruthenium catalysts, many protocols for their use require high temperatures or light irradiation. Now, the synthesis of an air- and moisture-stable ruthenium precatalyst has been reported. This versatile catalyst drives an array of transformations and enables rapid screening and optimization of reactions, revealing previously unknown in situ generated ruthenium complexes.

## Main

**Fig. 1:**
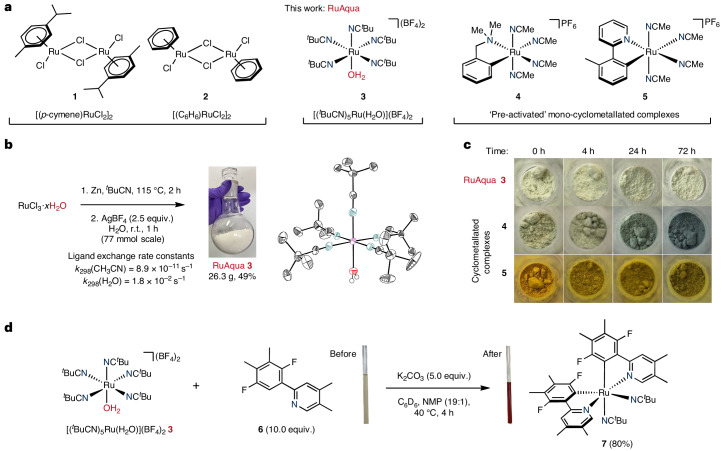
Design and synthesis of an air- and moisture-stable ruthenium(II) precatalyst. **a**, Selection of ruthenium(II) precatalysts typically used for application, discovery and synthetic method development within C–H functionalization chemistry. Broadly available air-stable precatalysts such as **1** and **2** exhibit poor levels of reactivity under mild reaction conditions and have high barriers that must be overcome to form active catalysts. Preactivated complexes such as **4** and **5** are highly reactive and operate under mild conditions but are extremely air sensitive. The air-stable complex [(^*t*^BuCN)_5_Ru(H_2_O)](BF_4_)_2_ (**3**) provides an alternative that has broad reactivity without the need for harsh reaction conditions. **b**, Left: synthesis of complex **3** by zinc reduction of ruthenium(III) trichloride and chloride-to-tetrafluoroborate metathesis. Right: X-ray crystal structure of **3** with 50% probability thermal ellipsoids; BF_4_ counterions are omitted for clarity. Color coding: lilac, Ru; red, O; blue, N; grey, C. Ligand exchange rate constants for [Ru(H_2_O)_6_]^2+^ and [Ru(NCMe)_6_]^2+^ are given in ref. ^15^. **c**, Air-stability test of solid complex **3**, **4** and **5** over 72 h. **d**, NMR study of stoichiometric arene C(*sp*^2^)–H bond activation under mild reaction conditions using **3** to give biscyclometallated species **7**—a key species required for reactivity with halide nucleophiles. Yield was determined by ^19^F NMR using 1,4-difluorobenzene as an internal standard. r.t., room temperature.

Synthetic chemistry has experienced substantial progress through the development of innovative catalysts, capable of modifying both simple and complex molecules with high efficiency and selectivity^1,2^. One crucial aspect of these advances has been the advent of robust catalysts that can be easily used and that operate under mild conditions, broadening their utility and applicability^3,4^. For example, the versatility of complexes such as palladium acetate or bis(cyclooctadiene)nickel(0), and their ability to form in situ new complexes, have been pivotal in shaping the development of palladium^5,6^ and nickel catalysis, respectively^7,8^. Ruthenium catalysts have exhibited powerful versatility for a broad selection of applications^9^. For example, a variety of synthetically powerful C–H functionalization reactions has been demonstrated using ruthenium catalysis^10,11^. However, despite their widespread utility, many of the developed protocols have necessitated either high reaction temperatures (80–140 °C) or light irradiation, which has limited overall ease of use and their suitability for the diversification of delicate high-complexity substrates and biomolecules^12^. This is a common occurrence when widely applied η^6^-arene coordinated ruthenium species such as [(*p*-cymene)RuCl_2_]_2_ **1** (ref. ^13^) and benzene analogue **2** are used, as they typically require additional energy to access active catalyst species (Fig. 1a).

In 2018, we reported the development of a monocyclometallated ruthenium catalyst, [(C_6_H_4_CH_2_NMe_2_)Ru(MeCN)_4_]PF_6_ **4**, that showed high activity at moderate temperatures (35–50 °C) for the C(*sp*^2^)–H arylation of arenes^12^. This precatalyst allowed for the direct late-stage C(*sp*^2^)–H arylation and alkylation of a wide array of pharmaceuticals and other biologically relevant molecules. Despite its powerful reactivity, the considerable air sensitivity of **4** has limited its synthetic applicability, requiring specialized storage and handling techniques that have prevented general adoption in most synthetic laboratories and within industrial settings. Key to any broadly applicable synthetic innovation is the use of operationally simple reagents and precatalysts that enable use by both the specialist and non-expert scientist.

Considering these limitations, the development of air-stable precatalysts with similar transformative power as air-sensitive complexes such as **4** is critical for increasing the accessibility and use of ruthenium catalysis. Therefore, we questioned the feasibility of designing a synthetically accessible precatalyst that would contain a balanced selection of ligands with varied labilities that would be both air- and moisture-stable and possess high levels of native reactivity. In this article, we present the development and broad applicability of an air- and moisture-stable ruthenium precatalyst, [(^*t*^BuCN)_5_Ru(H_2_O)](BF_4_)_2_ ‘RuAqua’ **3**, which displays a similar reactivity profile in C(*sp*^2^)–H functionalization to air-sensitive monocyclometallated complexes **4** and **5**, and does not require strict air-free handling or storage. Importantly, the versatility of RuAqua **3** extends to a diverse suite of synthetic transformations, including C–H functionalization, alkene isomerization, alkene oxidative cleavage and reductive transformations. Moreover, the labile ligand sphere allows for facile photocatalyst formation via mechanochemical milling, and additionally enables the rapid screening, optimization and selection of photocatalysts through in situ generation of photocatalyst species. The ease of use of RuAqua **3** should provide access to a broad suite of ruthenium-catalysed transformations to the widest possible userbase.

## Results and discussion

### Design, synthesis and stability of RuAqua

Successful realization of an air- and moisture-stable ruthenium precatalyst that imparted comparable levels of reactivity to that observed for monocyclometallated complexes such as **4** and **5** hinged on the design of a suitably balanced ligand set. The selected ligand framework would be key to render the resulting complex air- and moisture-stable but at the same time allow for facile access to catalytically active species in the presence of added substrates or reagents. From our previous work, we mechanistically posited that cyclometallated complex **4** underwent precatalyst activation from rapid ligand exchange when excess directing group arenes were added^12^. Guided by this hypothesis, we considered that a hexaaquaruthenium(II) complex containing six water ligands, that is, [Ru(H_2_O)_6_]^2+^, could be a suitable starting point because monodentate aqua ligands are generally more labile than other strongly coordinating heteroatom donors or multidentate ligand frameworks. Given the high exchange rate constant observed for aqua ligands within [Ru(H_2_O)_6_]^2+^ (*k* = 1.8 × 10^−2^ s^−1^), the use of this class of complex would conceptually allow for rapid precatalyst activation by fast sequential ligand exchange reactions^14^. However, Ludi and co-workers previously reported that hexaaquaruthenium(II) complexes underwent rapid oxidation in an aqueous solution and thus indicated that these species were not stable to atmospheric oxygen^15^.

We previously reported the use of an air- and moisture-stable hexakis(pivalonitrile)ruthenium(II) complex [Ru(^*t*^BuCN)_6_](BF_4_)_2_ for the C–H functionalization of benzoic acid derivatives, fluoroarenes and arenes bearing nitrogen-based directing groups^16,17^. However, in each of these applications this complex required high reaction temperatures to enable precatalyst activation by thermally induced ligand exchange. The necessity for elevated reaction temperatures is coherent with the low nitrile ligand exchange rate constant observed for the analogous hexakis-acetonitrile complex [Ru(MeCN)_6_]^2+^ (*k* = 8.9 × 10^−11^ s^−1^), that is, eight orders of magnitude slower than that of the corresponding aqua complex^14^.

To apply this knowledge, we considered an alternative precatalyst design using a hybrid mixed ligand set that consisted of both nitrile and aqua ligands. We reasoned that aqua ligands contained within any complex would essentially serve as pseudo-vacant sites to enable rapid reactivity and provide facile access to catalytically active species. Combined with the additional stability bestowed by nitrile ligands, this mixed ligand sphere design would confer both high levels of stability and reactivity in the presence of Lewis basic reactants.

To evaluate this hypothesis, we required synthetic access to a complex bearing a hybrid combination of both nitrile and aqua ligands. Therefore, we proceeded to synthesize a nitrile-containing complex starting with the zinc reduction of commercially available ruthenium(III) trichloride hydrate in pivalonitrile (^*t*^BuCN) as a solvent to give a putative ruthenium(II) dichloride intermediate bearing four pivalonitrile ligands. From here, the non-isolated intermediate species was subjected to reaction with excess silver tetrafluoroborate that proceeded in water to give [(^*t*^BuCN)_5_Ru(H_2_O)](BF_4_)_2_ (**3**) following metathesis and ligand redistribution in 49% yield (Fig. 1b). Notably, this procedure could be conducted on a decagramme scale to generate >26 g of complex **3**. The air- and moisture-stability of complex **3** was directly compared with that of cyclometallated species **4** and **5** (Fig. 1c). Whereas visible degradation of solid samples of sensitive complexes **4** and **5** was observed within hours, the mixed hybrid complex **3** showed robust tolerance to air (stable >1 year). Similarly, while benzylamine containing cyclometallated complex **4** decomposes in oxygenated solutions at room temperature within minutes, **3** proved stable to the open air in acetone, dichloromethane and methanol solutions (Supplementary Table 1). Additional characterization, including ultraviolet–visible spectroscopy, cyclic voltammetry and thermogravimetric analysis, was conducted to further understand the physical properties of complex **3** (Supplementary Information, pages 14–20). With the stability of **3** established, the second key design criteria of reactivity under mild conditions was tested by direct application to C(*sp*^2^)–H bond activation (Fig. 1d). Specifically, the reaction of **3** with phenylpyridine derivative **6** was achieved under mild reaction conditions (40 °C) to give biscyclometallated species **7** (80%, 4 h). Taken together, the stability of **3** and the ability to easily participate in C(*sp*^2^)–H bond activation without excessive applied heat or light irradiation was indicative of a viable and generally applicable alternative to many predecessor precatalysts.

### RuAqua as a precatalyst for C(*sp*^2^)–H functionalization

Given that **3** was compatible with stoichiometric C(*sp*^2^)–H bond activation, the true test of utility was the application of substoichiometric quantities of **3** as a precatalyst to a range of synthetic transformations. Therefore, we initially targeted arene C(*sp*^2^)–H arylation using aryl halide electrophiles as coupling partners (Table 1)^12^. Direct arylation of 2-(*o*-tolyl)pyridine **8a** using 5-bromo-*m*-xylene **9a** proceeded using **3** (5 mol%) as a precatalyst under mild heating (40 °C) to selectively give arylated product **10a** in excellent yield (92%). Notably, the analogous reaction using 4-iodoanisole **9b** could be performed within an industrial setting (AstraZeneca) on a 5 g scale using dimethylcarbonate as the solvent in 78% yield (Supplementary Information, pages 26–27). This reactivity underscores the practical superiority and advantage of RuAqua **3** in comparison to predecessor precatalysts. Precatalyst **3** further demonstrated applicability to the direct late-stage functionalization of **8a** using a selection of biologically active molecules, including haloperidol **9c**, chlormezanone **9d**, chlorpropham **9e** and Ladasten **9f**. Similarly, the identity of the directing-group arene was varied to utilize the benzodiazepine motif within diazepam **8b**, and the imidazo[1,2-a]pyridine group of zolimidine **8c**. In both cases, **8b** and **8c** underwent arylation with aryl halide electrophiles to give **11g** and **11h** in excellent yields (86% and 95%). The drug–drug conjugate **10i** was effectively synthesized in useful yield (50%) through the direct coupling of zolpidem derivative **8d** with clozapine **9h**. In each reaction using biologically active molecules as coupling partners, the procedure using **3** as a precatalyst selectively gave the coupled products in excellent yields and as single regioisomers.

**Table 1:**
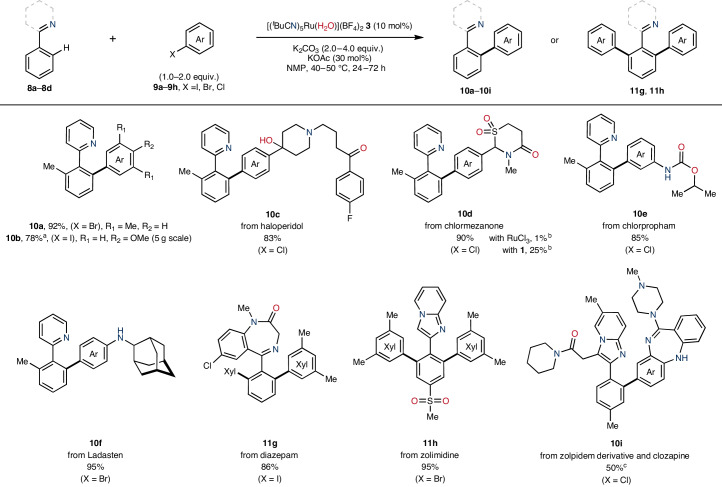
Precatalyst applicability for the direct C(*sp*^2^)–H bond arylation of directing-group arenes using (pseudeo)halide electrophiles under mild reaction conditions

^a^Process-relevant conditions: **3** (3.5 mol%), 4-iodoanisole (1.05 equiv.), K_2_CO_3_ (3.0 equiv.), TBAOAc (30 mol%), DMC (0.5 M), 50 °C, 22 h.

^b^Yields determined by ^1^H NMR spectroscopy using an internal standard.

^c^**3** (20 mol%), 144 h.

Applicability of **3** as a precatalyst was further extended beyond C(*sp*^2^)–H arylation to include *ortho*-selective C(*sp*^2^)–H alkylation with a selection of both primary and secondary halide electrophiles (Table 2, upper)^18,19^. Using the primary alkyl halide *n*-octylbromide **12a**, alkylation of several unique directing-group arenes was achieved. Specifically, successful *ortho*-selective alkylation was achieved in good to excellent yields giving products including aryl-pyridine **14a**, aryl-pyrimidine **14b**, aryl-isoquinoline **14c**, aryl-pyrazole **14d**, diazepam **14e** and aryl-ketone **14f** motifs. Similar applicability was observed in the *ortho*-selective alkylation using secondary alkyl bromides (Table 2, lower). A bromide derivative of oxaprozin **13a** containing a bromo-piperidine group underwent successful alkylation of 2-(*o*-tolyl)pyridine **8a** to give **15a** in good yield and as a single regioisomer. Similarly, pyrimidine **8f** was selectively alkylated using 4-bromotetrahydro-2H-pyran **13b** to give pyrimidine derivative **15b** in excellent yield. Substrates bearing increased levels of structural complexity were also applicable to secondary *ortho*-selective alkylation. For example, diazepam **8b** underwent *ortho*-alkylation using 4-bromotetrahydro-2H-pyran **13b** in moderate yield giving **15c** in 36% yield, and bromo-substituted epiandrosterone derivative **13c** was coupled with **8j** in excellent yield to give **15d** as a single diastereomer. Attempts to carry out these reactions with RuCl_3_ or ruthenium complex **1** led to low or no reactivity under the same reaction conditions (see **10d**, **14c** and **15b**).

**Table 2:**
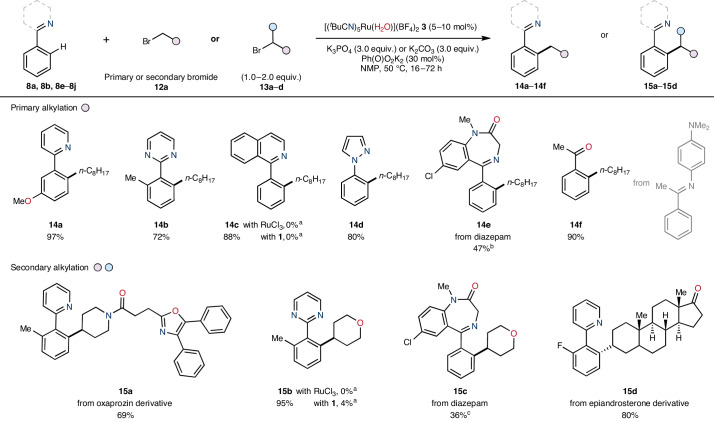
Precatalyst applicability for the direct C(*sp*^2^)–H bond alkylation of directing-group arenes using halide electrophiles under mild reaction conditions

^a^Yields determined by ^1^H NMR spectroscopy using an internal standard.

^b^**3** (20 mol%).

^c^96 h.

**Fig. 2:**
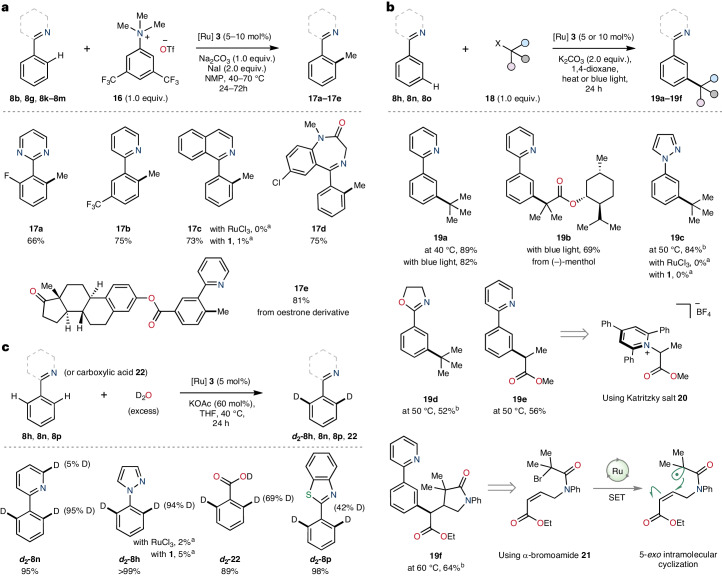
RuAqua 3 as a precatalyst for selective *ortho*- and monoselective arene methylation and applicability to distal *meta*-functionalization. **a**, Ruthenium-catalysed *ortho*-selective C(*sp*^2^)–H methylation using trimethylanilinium salt **16**. **b**, *Meta*-selective C(*sp*^2^)–H functionalization reactions. Applicability of **3** as a precatalyst for *meta*-selective alkylation was demonstrated using tertiary alkyl halides, an α-bromoester (to give **19b**), Katritzky salt **20** and using tandem-radical cyclization substrate **21**. Blue light, 440 nm; background temperature, 35 °C. SET, single-electron transfer. **c**, Hydrogen/deuterium exchange. ^2^D incorporation determined by integration of residual ^1^H resonances. ^a^Yields determined by ^1^H NMR spectroscopy using an internal standard. ^b^**3** (10 mol%).

Considering the catalytic capability and tolerance of RuAqua **3** when applied to C(sp^2^)–H bond coupling of complex molecules containing extensive functional group diversity, it was plausible to further expand this class of reactivity. We previously reported a mild synthetic strategy for the direct C(*sp*^2^)–H bond methylation of arenes bearing nitrogen-based directing groups using *N,N,N*-trimethylanilinium salts^20^. The developed procedure used air-sensitive precatalyst **4** to achieve *ortho*-selective arene methylation. Therefore, RuAqua **3** was tested as an air-stable alternative under comparable reaction conditions to that found optimal for **4** (Fig. 2a). Using electron-deficient anilinium triflate **16** as a source of methyl-coupling partner, the direct methylation of aryl-pyrimidine **8k** (**17a**), aryl-pyridine **8l** (**17b**), aryl-isoquinoline **8g** (**17c**) and diazepam **8b** (**17d**) was achieved, giving products in good yields. Application to an aryl-pyridine bearing a structurally complex oestrone unit **8m** was additionally achieved in excellent yield (**17e**).

A core strength of ruthenium catalysis within C(*sp*^2^)–H functionalization has been the ability to, with precision, predict and select the site of reactivity within arenes bearing nitrogen-directing groups^18–23^. For example, primary alkyl electrophiles and aryl electrophiles have shown a strong bias for *ortho*-C(*sp*^2^)–H functionalization^12,18,19^. Contrastingly, ruthenium catalysis using tertiary alkyl halides has demonstrated a propensity for *meta*-selective addition^21–23^. Predominantly, this class of reactions has been facilitated through the use of η^6^-arene containing precatalysts such as [(*p*-cymene)RuCl_2_]_2_ **1**, and at high temperatures or under continuous light irradiation. We therefore queried if RuAqua **3** could serve as a suitable precatalyst for *meta*-selective alkylation, and if this reactivity could be achieved under milder conditions than had been used previously (Fig. 2b). Accordingly, **3** was used as the precatalyst for the *meta*-selective coupling between 2-phenylpyridine **8n** and *tert*-butylbromide **18a** at both 40 °C or under blue light irradiation, which in each case gave **19a** in excellent yields and as a single regioisomer. Reactivity of this class operating under mild heating represents a meaningful improvement compared with previous reports that have typically required high temperatures (for example, 100–120 °C). The *meta*-selective reactivity was further exemplified using (−)-menthol-derivatized α-bromoester **18b** which required 440 nm light to generate **19b** in good yield. Alternative nitrogen-directing groups were similarly compatible. Specifically, aryl-pyrazole **8h** and aryl-oxazoline **8o** underwent successful reaction with *tert*-butylbromide **18a** at 50 °C to give the *meta*-alkylated products **19c** and **19d** in excellent and good yields, respectively. Use of Katritzky salt **20** further demonstrated the capability of RuAqua **3** to serve as a generally applicable precatalyst for *meta*-selective alkylation reactions^24^. For example, reaction of 2-phenylpyridine **8n** with **20** using **3** as a precatalyst gave *meta*-alkylated arene **19e** in good yield. Use of α-bromoamide **21** bearing a pendant acrylate allowed for the formation of γ-lactam **19f** following 5-*exo* intramolecular ring closure and subsequent *meta*-C(*sp*^2^)–H coupling^25^.

Given the demonstrated proficiency of RuAqua **3** in facilitating C(*sp*^2^)–H functionalization reactions, it was reasonable to hypothesize the presence of reactive aryl-ruthenium intermediates within the reaction mechanism. If this were the case, the addition of deuterium sources could potentially result in the incorporation of deuterium into the reacting substrate through reversible C(*sp*^2^)–H activation processes. To explore this possibility, we conducted a series of hydrogen/deuterium exchange experiments using *d*_2_-D_2_O (Fig. 2c). These tests revealed substantial deuterium incorporation at the C(*sp*^2^)–H activation site within the arene. Specifically, 2-phenylpyridine **8n** underwent hydrogen/deuterium exchange primarily at the *ortho* sites with remarkable levels of incorporation (95%). Similarly, 1-phenylpyrazole **8h**, benzoic acid **22** and 2-phenylbenzothiazole **8p** underwent C(*sp*^2^)–H activation and hydrogen/deuterium exchange with varying degrees of success, ranging from modest to excellent incorporations.

### RuAqua as a general precatalyst and synthetic precursor

**Fig. 3:**
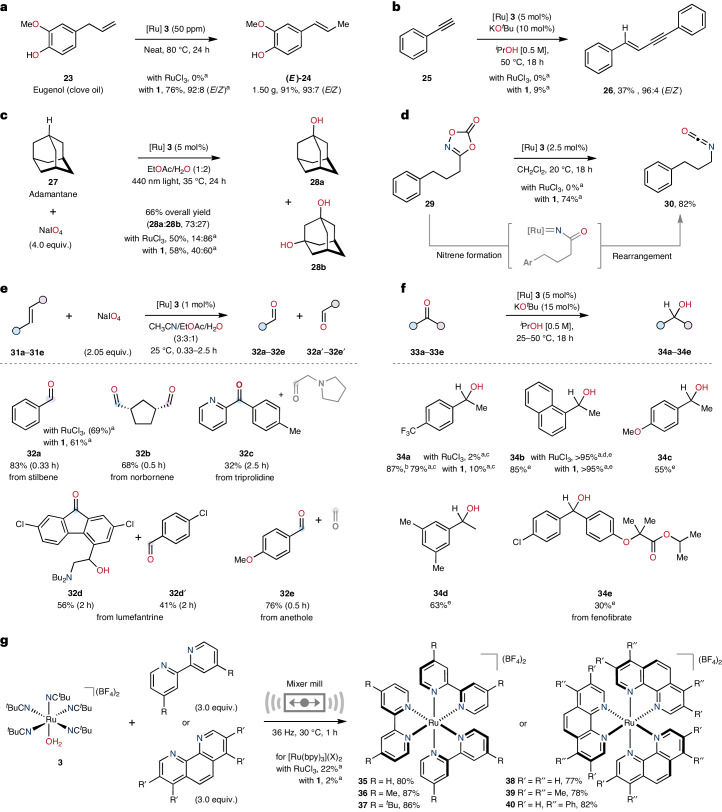
Generic applicability of RuAqua 3 for several synthetic transformations. **a**, Ruthenium precatalyst **3** is effective for the alkene isomerization of eugenol. **b**, Alkyne hydroalkynylation. **c**, C(*sp*^3^)–H bond oxidation using **3** as a precatalyst. **d**, Curtius rearrangement of a 1,4,2-dioxazol-5-one group. **e**, Alkene cleavage using sodium periodate catalysed by **3**. Volatile or unstable structures (grey fragments) were not isolated. **f**, Ketone transfer hydrogenation using isopropanol as the terminal reductant. **g**, Use of **3** as a synthetic precursor for photocatalyst formation using a mixer mill. ^a^Yields determined by ^1^H NMR spectroscopy using an internal standard. ^b^Reaction run at 25 °C. ^c^Reaction run at 25 °C for 8 h. ^d^Reaction was found to have low reproducibility with conversion varying between 10% and 95%. ^e^50 °C.

The comprehensive versatility exhibited by RuAqua **3** towards several reaction classes within C(*sp*^2^)–H bond functionalization suggested the possibility of wider generality. We recognized that the availability of an air-stable high-utility precatalyst that could be applied in various synthetic transformations would be broadly beneficial to the scientific community. Thus, we questioned the expected capabilities of a generic ruthenium precatalyst (Fig. 3). Reaction of eugenol **23** with parts per million quantities of **3** as a precatalyst allowed for efficient gramme-scale alkene isomerization to give β-methylstyrene derivative **24** in excellent yield and with high diasteroselectivity (Fig. 3a). This reactivity compares favourably with that of previous precedent using organometallic ruthenium complexes (that is, Ru(η^3^-methylallyl)_2_(1,5-cyclooctadiene, COD)) at elevated temperatures (150 °C) and with the lower reactivity displayed by both RuCl_3_ and **1** (ref. ^26^). RuAqua **3** further demonstrated reactivity in the anti-Markovnikov 1,2-hydroalkynylation of phenylacetylene **25** to give enyne **26** (Fig. 3b). Direct tertiary-C(*sp*^3^)–H bond oxidation of adamantane **27** was achieved using **3** as the precatalyst with excess sodium periodate to give both adamantan-1-ol **28a** and adamantan-1,3-diol **28b** in good overall yield (Fig. 3c)^27^. Moreover, the Curtius rearrangement of **29** containing the 1,4,2-dioxazol-5-one unit was facilitated using **3** to give isocyanate **30** in excellent yield (Fig. 3d). Additionally, RuAqua **3** proved effective for the oxidative cleavage of alkenes using sodium periodate (Fig. 3e)^28^. For example, stilbene **31a** underwent efficient cleavage to give benzaldehyde **32a** in good yield. Similarly, norbornene **31b**, triprolidine **31c**, lumefantrine **31d** and anethole **31e** were all applicable to RuAqua **3** catalysed oxidative cleavage to give their respective ketone and/or aldehyde products **32b**–**32e** (and **32b′**–**32e′**). The transfer hydrogenation of ketones could additionally be facilitated using **3** (Fig. 3f)^29^. Specifically, we targeted the reduction of simple ketones **33a**–**33d** using isopropanol as the terminal reductant which gave alcohols **34a**–**34d** in moderate to excellent yield. Moreover, the transfer hydrogenation showed reactivity when directly applied to the biologically active substrate fenofibrate **33e**, which gave the corresponding alcohol **34e**, albeit in low yield. Importantly, the reactivity demonstrated by RuAqua **3** was either comparable or far superior to that of alternative ruthenium precatalysts for this range of established catalytic transformations.

The synthetic utility of RuAqua **3** as a stoichiometric complex precursor was demonstrated through the synthesis of a range of ruthenium photocatalyst species (Fig. 3g). Given the established lability of the monodentate ligands within RuAqua **3**, we considered the ability of multidentate ligands to occupy the coordination sphere of ruthenium by displacement of the ligand set within **3**. To probe this possibility, **3** was reacted with excess bipyridine using mechanochemical force in a solvent-free mixer mill (36 Hz, 1 h), which gave the well-established photocatalyst [Ru(bpy)_3_]^2+^ **35** in excellent yield (80%)^30^. This reactivity was extended to a range of analogous bipyridine and phenanthroline ligands to give a structurally differentiated set of photocatalyst species **36**–**40** in excellent yields in all cases (77–87%). Classically, [Ru(bpy)_3_]^2+^ **35** and analogous derivatives, such as **36**–**40**, have typically been synthesized using RuCl_3_ as a precursor, under reducing conditions and at high temperatures^31^. Therefore, the ability to rapidly form this family of high-reactivity complexes within a mixer mill provides a useful alternative that lowers the synthetic effort required to generate wide photocatalyst libraries.

### RuAqua as a generic precatalyst for accelerated discovery

**Fig. 4:**
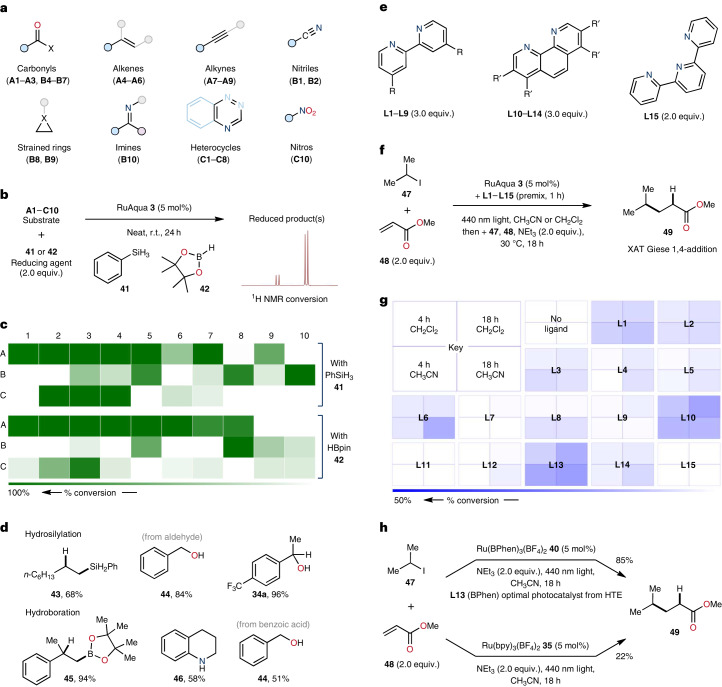
Use of RuAqua 3 as a platform precatalyst for discovery. **a**–**d**, Discovery platform for reduction reactions using **3** as the precatalyst. **a**, Selection of unsaturated compounds explored. **b**, Reaction set-up: reactions were conducted neat and at ambient temperature (19 °C) for 24 h. **c**, Results: conversion percentages of starting materials were determined by ^1^H NMR spectroscopy using an internal standard. **d**, Reactions were validated for select entries for both silane and borane reductants. **e**–**h**, High-throughput experimentation (HTE) photocatalyst selection by in situ formation of Ru(bpy)_3_^2+^ analogues applied to the XAT radical 1,4-addition. **e**, Ligands examined during screening. **f**, Reaction set-up: **3** premixed with **L1**–**L15** in CH_3_CN/CH_2_Cl_2_ and irradiated for 1 h; **47**, **48** and NEt_3_ (2.0 equiv.) were then added, and the mixture was further irradiated for 18 h. **g**, Results: reaction yields were determined by gas chromatography with flame ionization detection using an internal standard. **h**, Photocatalyst **40** (from **L13**) was isolated and used directly in the reaction and compared to [Ru(bpy)_3_](BF_4_)_2_.

The established applicability of RuAqua **3** for a diverse suite of synthetic transformations is indicative of a broadly useful synthetic tool. In particular, the generalizability exhibited by **3** offers potential applicability as a baseline precatalyst for the accelerated discovery of previously unreported reactions through high-throughput screening. To assess this concept, we applied RuAqua **3** as a precatalyst in combination with both a reductant and a variety of potentially reducible compounds (Fig. 4). A varied substrate pool was selected as reaction targets, including carbonyls, alkenes, alkynes, nitriles, strained rings, imines, heterocycles and a nitro compound (Fig. 4a). Separately, each substrate was simultaneously reacted with both phenylsilane **41** and pinacolborane **42** using **3** as a precatalyst, in the absence of solvent and at room temperature (Fig. 4b)^32^. After 24 h, the conversions for each reaction were determined by ^1^H NMR spectroscopy using an internal standard. Using silane reductant **41**, 20 reactions showed reactivity out of 30 total reactions (67% hit rate, Fig. 4c, top). Similarly, 21 reactions showed reactivity using borane **42** (70% hit rate, Fig. 4c, bottom). In general, using both silane **41** and borane **42**, carbonyl compounds (**A1**–**A3, B4**–**B7**), alkenes (**A4**–**A6**) and alkynes (**A7**–**A9**) underwent hydrosilylation and hydroboration (Supplementary Figs. 13 and 14). Nitriles (**B1**, **B2**) and nitro compound (**C10**) proved unreactive under reaction with silane **41**. However, using borane **42** allowed for partial reaction of a nitro compound (**C10**). Notably, several heterocycles (within **C1**–**C8**) showed reactivity, highlighting the possibility of using RuAqua **3** as a precatalyst for heterocycle hydrofunctionalization^33,34^. In fact, the reaction of quinoline (**C2**) with borane **42** resulted in clean heterocycle hydrogenation to give the corresponding tetrahydroquinoline **46** in good yield (58% isolated), a transformation previously unknown with ruthenium^35,36^. Reaction results were validated by selection of specific examples and performing replicate reactions with product isolations (Fig. 4d).

One powerful tenet of modern high-throughput screening and optimization methods is the modularity imparted by the choice of reactants, metal salts, ligands and solvents^37^. Conceptually, multifactorial reaction optimizations rely on the ability to use libraries of multiple components to combinatorically survey broad reaction space to find optimally efficient reaction conditions^38^. For synthetic catalysis, these strategies have benefited from the ability of available precursors to undergo in situ complexation with added ligand libraries. This allows for the rapid assessment of many metal (pre)catalysts without the need for prior isolation and screening of each individual complex. The lability of the monodentate ligand sphere within RuAqua **3** therefore presented an opportunity to test if this concept could be applied within ruthenium catalysis. We specifically questioned if the in situ formation of ruthenium photocatalyst species would demonstrate this concept and allow for simultaneous screening of large ligand libraries in the optimization of Dexter energy transfer or photoredox catalysis without the need to separately synthesize each photocatalyst. This hypothesis was explored via the halogen-atom-transfer (XAT) radical Giese-type addition of isopropyl iodide **47** to methyl acrylate **48** (ref. ^39^). A range of nitrogen-based ligands, including bipyridine derivatives (**L1**–**L9**), phenanthroline derivatives (**L10**–**L14**) and terpyridine **L15**, were precomplexed with RuAqua **3** (Fig. 4e). This process was conducted for 1 h in either acetonitrile or dichloromethane under blue light irradiation, followed by the addition of reactants **47** and **48**. The reactions were sampled at 4 h and 18 h to create a reactivity map for all ligands in both solvents (Fig. 4f). The results indicated that bathophenanthroline (BPhen) ligand **L13** performed optimally in acetonitrile and phenanthroline (Phen) ligand **L10** in dichloromethane (Fig. 4g). Importantly, commonly used bipyridine (**L1**) led to poor reactivity in both solvents. To validate these findings, the isolated photocatalyst species **35** and **40** were tested in acetonitrile under the same reaction conditions (Fig. 4h). These results affirmed our high-throughput screen, with Ru(BPhen)_3_^2+^ **40** delivering a product yield of 85%, vastly outperforming Ru(bpy)_3_^2+^ **35**, which gave only 22%. Taken together, these results illustrate the generic applicability of RuAqua **3** as a platform precatalyst for the discovery of synthetic inventions and for expedited multifactorial optimizations.

In summary, we have outlined the design and synthesis of an air- and moisture-stable ruthenium precatalyst [(^*t*^BuCN)_5_Ru(H_2_O)](BF_4_)_2_ (**3**). Notably, synthetic access to decagramme quantities of **3** after two short and simple synthetic steps highlights the potential for ease of manufacture and widespread adoption. Application of **3** as a precatalyst demonstrated a generally applicable alternative to many predecessor complexes for several different C(*sp*^2^)–H bond functionalization reactions. Namely, **3** proved effective for *ortho*-C(*sp*^2^)–H arylation, alkylation and methylation and for *meta*-C(*sp*^2^)–H alkylation. Further capability for a range of diverse synthetic transformations was highlighted by successful demonstration of alkene isomerization, hydroalkynylation, C(*sp*^3^)–H oxidation, Curtius rearrangement, alkene cleavage and transfer hydrogenation. Moreover, the suitability for **3** to be used as a generic precatalyst for discovery was exemplified through successful application to the reduction of several classes of reducible compounds. Importantly, the labile ligand environment allowed for in situ formation of photocatalyst species, facilitating high-throughput screening and accelerating reaction optimization through catalyst selection. The broad generality and operational simplicity of RuAqua **3** provides a platform for the discovery and development of widely accessible ruthenium-catalysed synthetic transformations.

## Methods

### Preparation of RuAqua 3

The reaction was set up in an argon-filled glovebox: RuCl_3_·*x*H_2_O (1.0 equiv., assuming *x* = 3), zinc dust (<10 μm, 4.0 equiv.) and pivalonitrile were loaded in an Ace pressure tube which was subsequently wrapped in Teflon tape and parafilm. The sealed tube was taken out of the glovebox and heated for 2 h at 115 °C behind a blast shield. The reaction mixture was cooled to room temperature and the pivalonitrile removed under reduced pressure. The resulting mixture was diluted with HPLC-grade water before being filtered through a small plug of Celite to remove all solids. AgBF_4_ (2.5 equiv.) was added and the reaction was vigorously stirred for 1 h at room temperature before filtering through a small plug of Celite and evaporating to dryness. The residue was dissolved in acetone, filtered through a small plug of Celite and evaporated to dryness. This was then dissolved in CH_2_Cl_2_, filtered through a small plug of Celite and evaporated to dryness. The residue was dissolved in CH_2_Cl_2_ and precipitated with Et_2_O, affording a light-yellow solid. The solid was collected, dissolved in CH_2_Cl_2_ and precipitated with Et_2_O. The last precipitation step was reiterated until RuAqua was obtained as a fine, light-yellow powder.

## Online content

Any methods, additional references, Nature Portfolio reporting summaries, source data, extended data, supplementary information, acknowledgements, peer review information; details of author contributions and competing interests; and statements of data and code availability are available at 10.1038/s41557-024-01481-5.

### Supplementary information

Supplementary Information Experimental data, procedural details, synthesis and characterization data, Supplementary Figs. 1–14 and Tables 1–6.

Supplementary Data Crystallographic data for compound **3** (CCDC reference 2264775).

Supplementary Video Video of mechanochemical mixer mill set-up.

## Data Availability

The data supporting the findings of this work are provided in the Supplementary Information. Crystallographic data for the structure reported in this article have been deposited at the Cambridge Crystallographic Data Centre, under deposition number CCDC 2264775 (**3**). Copies of the data can be obtained free of charge via https://www.ccdc.cam.ac.uk/structures/.

## References

[1] Brown DG, Boström J (2016). Analysis of past and present synthetic methodologies on medicinal chemistry: where have all the new reactions gone?. J. Med. Chem..

[2] Cernak T, Dykstra KD, Tyagarajan S, Vachal P, Krska SW (2016). The medicinal chemist’s toolbox for late stage functionalization of drug-like molecules. Chem. Soc. Rev..

[3] Johansson Seechurn CCC, Kitching MO, Colacot TJ, Snieckus V (2012). Palladium-catalyzed cross-coupling: a historical contextual perspective to the 2010 Nobel Prize. Angew. Chem. Int. Ed..

[4] Shaw MH, Twilton J, MacMillan DWC (2016). Photoredox catalysis in organic chemistry. J. Org. Chem..

[5] Christmann U, Vilar R (2005). Monoligated palladium species as catalysts in cross-coupling reactions. Angew. Chem. Int. Ed..

[6] Lee HG, Milner PJ, Buchwald SL (2014). Pd-catalyzed nucleophilic fluorination of aryl bromides. J. Am. Chem. Soc..

[7] Nattmann L, Saeb R, Nöthling N, Cornella J (2020). An air-stable binary Ni(0)–olefin catalyst. Nat. Catal..

[8] Tran VT (2020). Ni(COD)(DQ): an air-stable 18-electron nickel(0)-olefin precatalyst. Angew. Chem. Int. Ed..

[9] Naota T, Takaya H, Murahashi S (1998). Ruthenium-catalyzed reactions for organic synthesis. Chem. Rev..

[10] Wencel-Delord J, Dröge T, Liu F, Glorius F (2011). Towards mild metal-catalyzed C–H bond activation. Chem. Soc. Rev..

[11] Arockiam PB, Bruneau C, Dixneuf PH (2012). Ruthenium(II)-catalyzed C–H bond activation and functionalization. Chem. Rev..

[12] Simonetti M, Cannas DM, Just-Baringo X, Vitorica-Yrezabal IJ, Larrosa I (2018). Cyclometallated ruthenium catalyst enables late-stage directed arylation of pharmaceuticals. Nat. Chem..

[13] Bennett MA, Huang TN, Matheson TW, Smith AK (1982). (η^6^-Hexamethylbenzene)ruthenium complexes. Inorg. Synth..

[14] Rapaport I, Helm L, Merbach AE, Bernhard P, Ludi A (1988). High-pressure NMR kinetics. Part 34. Variable-temperature and variable-pressure NMR kinetic study of solvent exchange on hexaaquaruthenium(3+) and -(2+) and hexakis(acetonitrile)ruthenium(2+). Inorg. Chem..

[15] Bernhard P, Buergi HB, Hauser J, Lehmann H, Ludi A (1982). Syntheses and crystal and molecular structures of hexaaquaruthenium(II) *p*-toluenesulfonate and hexaaquaruthenium(III) *p*-toluenesulfonate, Ru(H_2_O)_6_(C_7_H_7_SO_3_)_2_ and [Ru(H_2_O)_6_](C_7_H_7_SO_3_)_3_·3H_2_O. Inorg. Chem..

[16] Simonetti M (2017). Ruthenium-catalyzed C–H arylation of benzoic acids and indole carboxylic acids with aryl halides. Chem. Eur. J..

[17] Simonetti M (2016). Ru-catalyzed C–H arylation of fluoroarenes with aryl halides. J. Am. Chem. Soc..

[18] Wheatley M (2021). Ru-catalyzed room-temperature alkylation and late-stage alkylation of arenes with primary alkyl bromides. Chem Catalysis.

[19] Wang GW, Wheatley M, Simonetti M, Cannas DM, Larrosa I (2020). Cyclometalated ruthenium catalyst enables *ortho*-selective C–H alkylation with secondary alkyl bromides. Chem.

[20] Hogg A (2022). Ruthenium-catalyzed monoselective C–H methylation and d_3_-methylation of arenes. JACS Au.

[21] Li J (2017). Ruthenium(II)-catalysed remote C–H alkylations as a versatile platform to *meta*-decorated arenes. Nat. Commun..

[22] Sagadevan A, Greaney MF (2019). Meta-selective C-H activation of arenes at room temperature using visible light: dual-function ruthenium catalysis. Angew. Chem. Int. Ed..

[23] Gandeepan P, Koeller J, Korvorapun K, Mohr J, Ackermann L (2019). Visible-light-enabled ruthenium-catalyzed meta-C-H alkylation at room temperature. Angew. Chem. Int. Ed..

[24] Zhu ZF, Chen GL, Liu F (2021). Ruthenium-catalysed *meta*-selective CAr–H bond alkylation via a deaminative strategy. Chem. Commun..

[25] Gou XY (2021). Ruthenium-catalyzed radical cyclization/*meta*-selective C–H alkylation of arenes via *σ*-activation strategy. ACS Catal..

[26] Sanz-Navarro S (2022). Parts-per-million of ruthenium catalyze the selective chain-walking reaction of terminal alkenes. Nat. Commun..

[27] Mack JBC, Gipson JD, Bois JD, Sigman MS (2017). Ruthenium-catalyzed C–H hydroxylation in aqueous acid enables selective functionalization of amine derivatives. J. Am. Chem. Soc..

[28] Yang D, Zhang C (2001). Ruthenium-catalyzed oxidative cleavage of olefins to aldehydes. J. Org. Chem..

[29] Wang D, Astruc D (2015). The golden age of transfer hydrogenation. Chem. Rev..

[30] Teegardin K, Day JI, Chan J, Weaver J (2016). Advances in photocatalysis: a microreview of visible light mediated ruthenium and iridium catalyzed organic transformations. Org. Process Res. Dev..

[31] Broomhead, J. A. & Young, C. G. in *Inorganic Syntheses*, Vol. 28 (ed. Angelici, R. J.) 338–340 (Wiley, 1990).

[32] Agahi R (2019). Regiodivergent hydrosilylation, hydrogenation, [2*π* + 2*π*]-cycloaddition and C–H borylation using counterion activated Earth-abundant metal catalysis. Chem. Sci..

[33] Behera D, Thiyagarajan S, Anjalikrishna PK, Suresh CH, Gunanathan C (2021). Ruthenium(II)-catalyzed regioselective 1,2-hydrosilylation of N-heteroarenes and tetrel bonding mechanism. ACS Catal..

[34] Ma X, Mane MV, Cavallo L, Nolan SP (2023). Ruthenium-catalyzed regioselective 1,2-hydrosilylation of N-heteroarenes. Eur. J. Org. Chem..

[35] Kaithal A, Chatterjee B, Gunanathan C (2016). Ruthenium-catalyzed regioselective 1,4-hydroboration of pyridines. Org. Lett..

[36] Pandey VK, Sahoo S, Rit A (2022). Simple silver(I)-salt catalyzed selective hydroboration of isocyanates, pyridines, and quinolines. Chem. Commun..

[37] Renom-Carrasco M, Lefort L (2018). Ligand libraries for high throughput screening of homogeneous catalysts. Chem. Soc. Rev..

[38] Isbrandt ES, Sullivan RJ, Newman SG (2019). High throughput strategies for the discovery and optimization of catalytic reactions. Angew. Chem. Int. Ed..

[39] Constantin T (2020). Aminoalkyl radicals as halogen-atom transfer agents for activation of alkyl and aryl halides. Science.

