# Supplementary material for: An air- and moisture-stable ruthenium precatalyst for diverse reactivity
Source: Nat Chem. 2024 Apr 3;16(7):1141–50. doi: 10.1038/s41557-024-01481-5 (PMC11230907; doi:10.1038/s41557-024-01481-5)
Supplement: Supplementary file 1 — Experimental data, procedural details, synthesis and characterization data, Supplementary Figs. 1–14 and Tables 1–6. [file 41557_2024_1481_MOESM1_ESM.pdf]

nature chemistry

Article https://doi.org/10.1038/s41557-024-01481-5

An air- and moisture-stable ruthenium precatalyst for diverse reactivity

In the format provided by the authors and unedited

Table of Contents

General Information ... 4

Synthesis and Analysis of RuAqua ... 6

General Procedure A: Preparation of RuAqua ... 6

Synthesis of RuAqua ... 7

Recycling of Pivalonitrile in RuAqua Synthesis ... 9

Air- and Moisture-Stability of RuAqua ... 10

Exposure of $[(C_6H_4CH_2NMe_2)Ru(MeCN)_4]PF_6$ **4** Solution to Atmosphere ... 11

RuAqua Catalyst Stress Test ... 12

Solubility of RuAqua ... 13

Thermogravimetric Analysis (TGA) of RuAqua ... 14

CV of RuAqua ... 15

UV-Vis of RuAqua ... 16

Cost of RuAqua Synthesis ... 17

Trace Metal Analysis of RuAqua ... 18

Synthesis and Application of RuAqua with Alternative Counterions ... 19

Comparison of RuAqua with $[Ru(^tBuCN)_6](BF_4)_2$ and $[Ru(p\text{-cymene})Cl_2]_2$ ... 27

RuAqua catalysed C–H Arylation of Arenes with Aryl (Pseudo)Halides ... 29

Attainment of Starting Materials: ... 29

General Procedure B: RuAqua catalysed arylation of DG-containing arenes with aryl (pseudo)halides ... 35

Further Examples of RuAqua catalysed C–H Arylation ... 48

The Need for Inert Conditions During C–H Functionalisation Reactions ... 53

The Potential for Reduced Catalyst Loadings ... 54

Effect of Pivalic Acid on *ortho*-C–H Arylations ... 55

RuAqua Catalysed C–H Alkylation of Arenes with Primary Alkyl Bromides ... 56

Attainment of Starting Materials ... 56

General Procedure C: RuAqua Catalysed Primary Alkylation of DG-Containing Arenes with Primary Alkyl Bromides ... 63

Further examples of RuAqua Catalysed C–H Primary Alkylation ... 71

RuAqua Catalysed C–H Alkylation of Arenes with Secondary Alkyl Bromides ... 78

Attainment of Starting Materials ... 78

General Procedure D: RuAqua Catalysed Secondary Alkylation of Arenes with Secondary Alkyl Bromides ... 84

Further examples of RuAqua Catalysed C–H Secondary Alkylation ... 90

RuAqua Catalysed C–H Secondary Alkylation Using Acyclic Alkyl Bromides 97
RuAqua Catalysed C–H Methylation of Arenes with Anilinium Salt 98
Attainment of Starting Materials 98
General Procedure E: RuAqua Catalysed Methylation of Arenes with Anilinium Salt **16** 104
Further Examples of RuAqua Catalysed C–H Methylation 112
Meta-Selective C(sp^2)–H Functionalisation Reactions 119
Attainment of Starting Materials 119
General Procedure F: *Meta*-Functionalisation with RuAqua 123
RuAqua Catalysed H / D Exchange 132
Attainment of Starting Materials 132
General Procedure G: RuAqua catalysed H / D exchange 133
RuAqua Catalysed Alkene Isomerisation 139
Synthesis of (*E*)-2-methoxy-4-(prop-1-en-1-yl)phenol **24** 139
RuAqua Catalysed Hydroalkynylation 141
Synthesis of (*E*)-But-1-en-3-yne-1,4-diyldibenzene **26** 141
RuAqua Catalysed C(sp^3)–H Oxidation 142
Synthesis of Adamantan-1-ol **28a** and adamantane-1,3-diol **28b** 142
RuAqua Catalysed Curtius Rearrangement 144
Synthesis of (3-isocyanatopropyl)benzene **30** 144
RuAqua Catalysed Oxidative Alkene Cleavage 146
Attainment of Starting Materials 146
General Procedure H: Oxidative Cleavage of Alkenes 147
RuAqua Catalysed Transfer Hydrogenation 154
Attainment of Starting Materials 154
General Procedure I: RuAqua Catalysed Transfer Hydrogenation 155
Photocatalyst Synthesis via Mixer Mill 163
Attainment of Starting Materials 163
General Procedure J: Synthesis of Tris(bipyridine)ruthenium Derivatives 164
RuAqua as a Platform pre-Catalyst for the Discovery of New Reactions 171
Simulated High-Throughput Reaction Discovery Using RuAqua 171
Substrate Reduction using Phenylsilane 172
Substrate Reduction using Pinacolborane 173
General Procedure K: Validation of RuAqua Catalysed Hydrosilylation 174
General Procedure L: Validation of RuAqua Catalysed Hydroboration 178
High Throughput Experimentation for Selection of Photocatalyst via *in situ* Generation 182
Formation and Evaluation of Photocatalysts Generated from RuAqua 182

General Procedure M: Direct Employment of Photocatalysts in Giese 1,4-addition 184
NMR Spectra 186
Crystallographic section. 434
References 436

General Information

Reaction Setup: All reactions using RuAqua were set up and ran under argon using standard Schlenk technique unless otherwise indicated. Inorganic bases and additives such as K_2CO_3 and KOAc were dried in a vacuum oven at 80 °C for 48 hours prior to use. Reactions using light were set up using a Kessil lamp (blue light, PR160L 440 nm).

Solvents and liquid reagents: Solvents were degassed with three freeze-pump-thaw cycles prior to use and stored over 4 Å molecular sieves. Liquid reagents were degassed by purging with argon/nitrogen for 10 minutes unless otherwise stated.

NMR Spectroscopy: ^{1}H NMR, ^{19}F NMR and ^{13}C NMR spectra were recorded at 400 or 500 MHz on Bruker instruments. ^{1}H NMR are referenced to the residual solvent peak at 7.26 ($CDCl_3$), 5.35 (CD_2Cl_2), 3.31 (CD_3OD), 2.50 ($(CD_3)_2SO$) or 2.05 ppm ($(CD_3)_2CO$). ppm values are quoted to 2 decimal places, with coupling constants (J) to the nearest 0.1 Hz. ^{13}C NMR spectra were recorded at 126 or 100 MHz and quoted in ppm to 1 decimal place with coupling constants (J) to the nearest 0.1 Hz. The spectra were referenced to the residual solvent peak at 77.16 ppm ($CDCl_3$), 53.84 ppm (CD_2Cl_2), 49.00 (CD_3OD), 39.52 ($(CD_3)_2SO$) or 29.84 ppm ($(CD_3)_2CO$). ^{19}F NMR spectra recorded at 471 or 376 MHz in $CDCl_3$ and quoted in ppm to 1 decimal place with coupling constants (J) to the nearest 0.1 Hz. ^{11}B NMR were recorded at 128 MHz in $CDCl_3$ and are reported to 1 decimal place. ^{2}D NMR were recorded at 77 MHz in $CHCl_3$ and are reported to 2 decimal places. The residual solvent peak was referenced to 77.16 ppm.

IR Spectroscopy: IR spectra were recorded using a Thermo Scientific Nicolet iS5 FTIR machine, relevant bands are quoted in cm^{-1}.

Mass Spectroscopy: High resolution mass spectra were performed by the School of Chemistry Mass Spectrometry Service (University of Manchester) employing a Thermo Finnigan MAT95XP spectrometer.

Melting Points: Melting points were obtained using a Stuart SMP11 apparatus and are uncorrected.

Chromatography: Column chromatography was carried out on silica gel (particle size 40–63 µm) using using a Biotage Isolera™ Four with 10, 25 or 50 g SNAP cartridges using the indicated conditions.

Thermogravimetric analysis (TGA): TGA was performed using a TA instrument SDT650 under a nitrogen atmosphere from 30–500 °C at 10 °C / min.

Trace metal analysis: Trace metal analysis was performed using a Perkin Elmer NexION 350D ICP-MS.

UV-Vis: UV/Vis absorption spectra were recorded on an Agilent Cary 60 spectrometer.

Cyclic Voltammetry (CVs): Cyclic voltammetry (CV) experiments were conducted using a PalmSens4 potentiostat, controlled using PSTrace 5.9 software. The working electrode was a GC disc (3 mm dia., BASi part number MF-2012), the counter electrode was a Pt-wire (BASi part number MW-4130) and a Ag/AgCl reference electrode was used (BASi part number – MF-2052).

Ball Milling: Ball Milling reactions were carried out on an IST 636 mixer mill from Insolido Technologies. Reactions were carried out in 15 mL smartsnap stainless steel jars from Form-Tech Scientific. Stainless steel ball bearings were purchased from amazon with 4 × 7 mm ball bearings used in each reaction.

Chemicals: All the starting materials and solvents were purchased from Acros (Fisher), Aldrich (Merck), Alfa Aesar (Fisher), Fluorochem and Generon and used without further purification unless otherwise stated.

Synthesis and Analysis of RuAqua

General Procedure A: Preparation of RuAqua

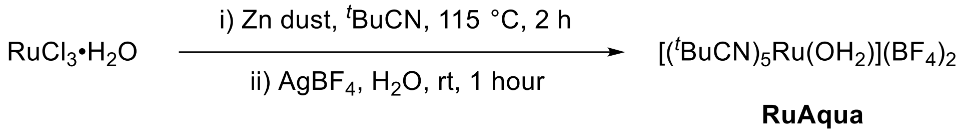

Reaction was set up in an argon filled glovebox: $RuCl_3$ •×H_2O (1.0 equiv., assuming × = 3), Zn dust (<10 µm, 4.0 equiv.) and pivalonitrile were loaded in an Ace pressure tube which was subsequently wrapped in Teflon tape and parafilm. The sealed tube was taken out of the glovebox and heated for 2 h at 115 °C behind a blast shield. The reaction mixture was cooled to room temperature and the pivalonitrile removed under reduced pressure. The resulting mixture was diluted with HPLC-grade water before being filtered through a small plug of Celite® to remove all solids. $AgBF_4$ (2.5 equiv.) was added and the reaction was vigorously stirred for 1 h at room temperature before filtering through a small plug of Celite® and evaporating to dryness. The residue was dissolved in acetone, filtered through a small plug of Celite® and evaporated to dryness. This was then dissolved in CH_2Cl_2, filtered through a small plug of Celite® and evaporated to dryness. The residue was dissolved in CH_2Cl_2 and precipitated with Et_2O affording a light-yellow solid. The solid was collected, dissolved in CH_2Cl_2 and precipitated with Et_2O. The last precipitation step was reiterated until **RuAqua** was obtained as a fine, light-yellow powder.

^{1}H NMR: (500 MHz, CD_2Cl_2)

1.43 (s, 9H), 1.38 (s, 36H).

^{13}C NMR: (126 MHz, CD_2Cl_2)

135.8, 133.8, 30.8, 30.4, 28.3, 27.9.

^{19}F NMR: (376 MHz, CD_2Cl_2)

-150.0 (2 x resonances)

IR: V_{max} (neat, cm^{-1})

3394 (br), 2970, 2940, 2279, 1467, 1244, 1078, 995.

MS: HRMS (ESI^+)

Calculated for $[Ru(H_2O)(^tBuCN)_5]^+(BF_4)$: 622.2848, found: 622.2820.

m.p: Recrystallized *via* slow diffusion of diethyl ether into solution in CH_2Cl_2.

180–182 °C (decomp.)

Synthesis of RuAqua

i) Reaction was set up in an argon filled glovebox: The general procedure A was applied using $RuCl_3 \bullet xH_2O$ (1.5 g, 5.7 mmol, 1 equiv.), zinc dust (1.5 g, 22.9 mmol, 4.0 equiv.) and pivalonitrile (35 mL). After allowing the reaction mixture to stir and removing the pivalonitrile under reduced pressure, HPLC grade water (150 mL) was added and the resulting mixture was filtered through a small plug of celite ensuring all solids were removed. $AgBF_4$ (2.8 g, 14.5 mmol, 2.5 equiv.) was added and the resulting solution and was allowed to stir at room temperature for 1 hour. The product was precipitated with Et_2O from a solution in CH_2Cl_2 5 times giving **RuAqua** as a fine light-yellow powder (2.2 g, 54% yield).

ii) Reaction was set up in an argon filled glovebox: The general procedure A was applied using of $RuCl_3 \bullet xH_2O$ (20.0 g, 76.5 mmol, 1 equiv.), zinc dust (20.0 g, 307 mmol, 4.0 equiv.) of and pivalonitrile (460 mL). This was performed over two reaction vessels in two halves due to limitations in container size. After allowing the reaction mixture to stir, the reaction mixtures were combined and the pivalonitrile was removed under reduced pressure. HPLC grade water (2.00 L) was added and the resulting mixture filtered through a small plug of celite taking care to ensure that all solids were removed. This was again split equally into two reaction vessels due to limitations in container size. $AgBF_4$ (37.4 g total, 192 mmol, 2.5 equiv.) was added and the resulting solutions were allowed to stir at room temperature for 1 hour. The mixtures were once again combined and the product was precipitated with Et_2O from a solution in CH_2Cl_2 6 times giving **RuAqua** as a fine light-yellow powder (26.3 g, 49% yield). See following for images of larger scale synthesis.

i)

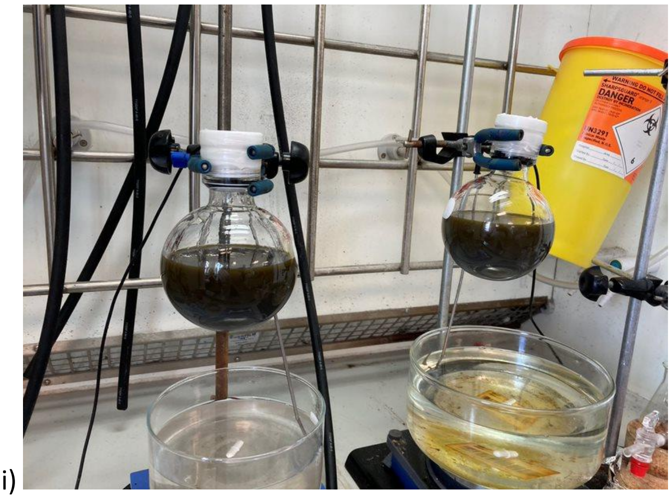

ii)

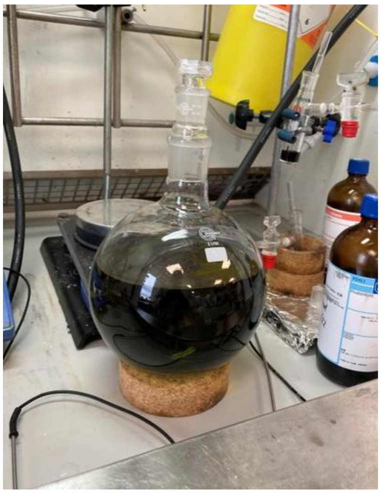

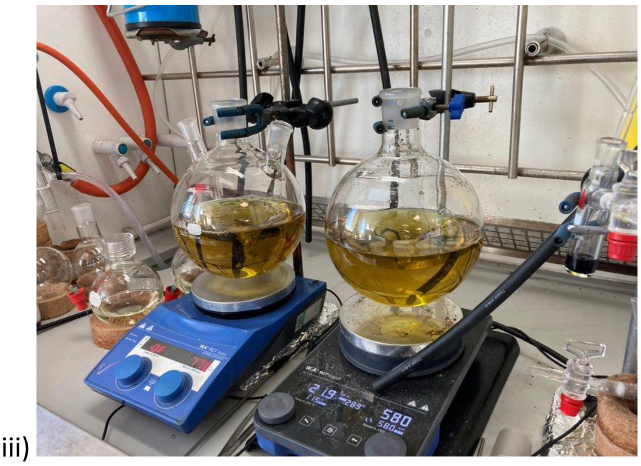

iii)

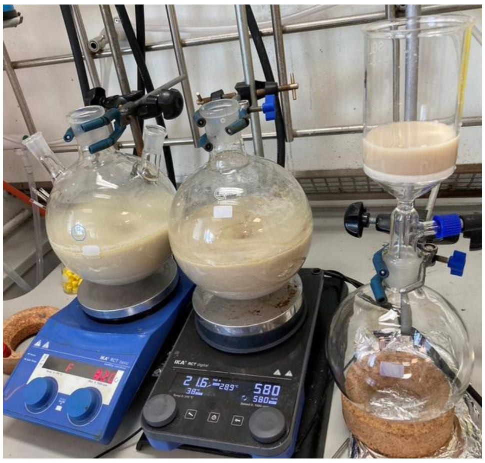

iv)

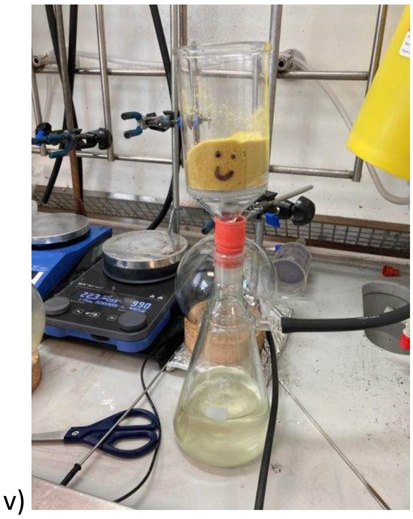

v)

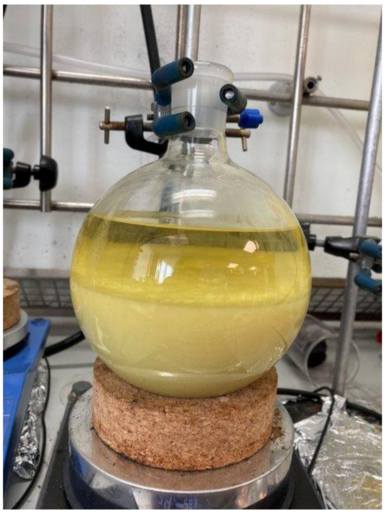

vi)

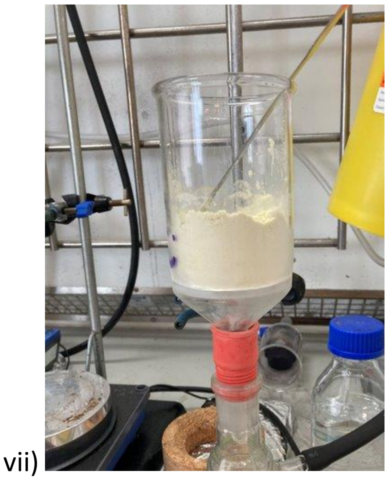

vii)

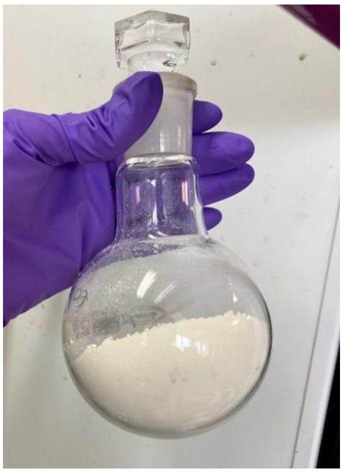

viii)

Figure 1 Large scale synthesis of **RuAqua**. i) $RuCl_3$ and Zn in ^{t}BuCN split over two containers ii) combined mixture after removal of pivalonitrile and dilution with H_2O iii) reaction mixture in water prior to addition of $AgBF_4$ iv) reaction after mixing with $AgBF_4$ for 1 h v) filtration after first precipitation vi) precipitation with Et_2O from CH_2Cl_2 vii) filtration after all precipitations viii) RBF containing final product **RuAqua**

Recycling of Pivalonitrile in RuAqua Synthesis

During the synthesis of **RuAqua**, the remaining pivalonitrile is removed under reduced pressure before the addition of $AgBF_4$ and water. The pivalonitrile could be taken and reused directly from the rotary evaporator without the need for purification. Before removing excess solvent, the rotary evaporator was cleaned by flushing with acetone several times allowing any residual acetone to evaporate before further use. The pivalonitrile was then removed from the reaction mixture under reduced pressure. From the 460 mL of pivalonitrile added to the reaction, approximately 400 mL of pivalonitrile was recollected.

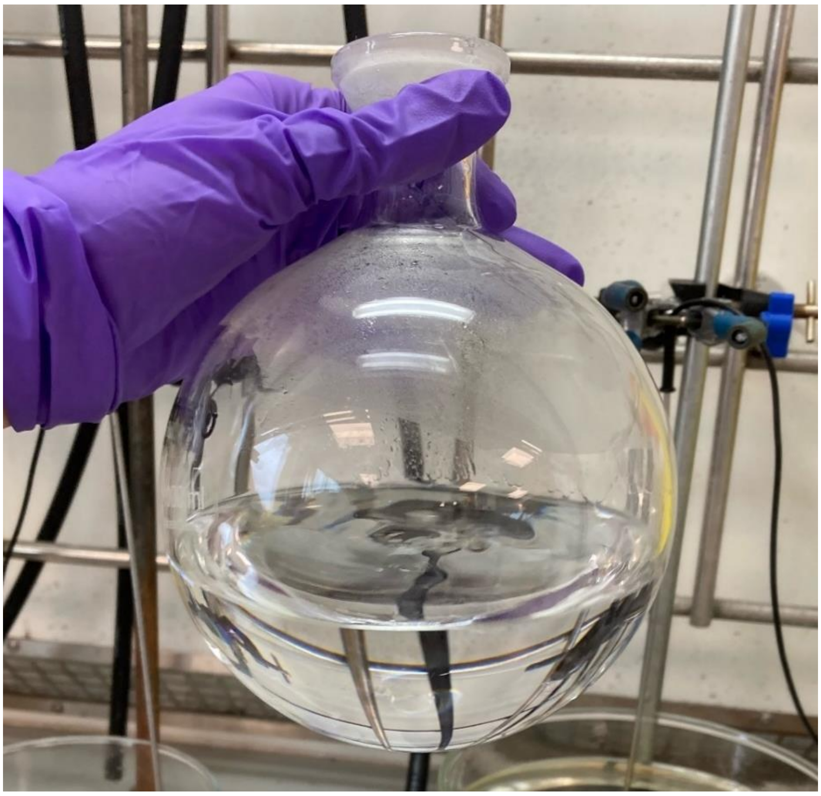

Figure 2 Pivalonitrile in collection flask from rotary evaporator

RuAqua was synthesized using the recycled pivalonitrile in comparable yield. Reaction was set up in an argon filled glovebox: General Procedure A was applied using 1.5 g (5.7 mmol) of $RuCl_3$ •×H_2O, zinc dust (1.5 g, 22.9 mmol, 4.0 equiv.) of and 35 mL of recycled pivalonitrile. After allowing the reaction mixture to stir and removing the pivalonitrile under reduced pressure, HPLC grade water (150 mL) was added and the resulting mixture filtered through a small plug of celite ensuring all solids were removed. $AgBF_4$ (2.8 g, 14.5 mmol) was added and the resulting solution and was allowed to stir at room temperature for 1 hour. The product was precipitated with Et_2O from a solution in CH_2Cl_2 5 times giving **RuAqua** as a fine light-yellow powder (1.90 g, 47% yield).

Air- and Moisture-Stability of RuAqua

After its initial synthesis, a small sample of the first ever batch of **RuAqua** was kept in a standard sample vial to assess its air stability. Comparison of NMR data between the first ever batch after over a year of storage was compared to a freshly made batch. These NMR spectra are shown below.

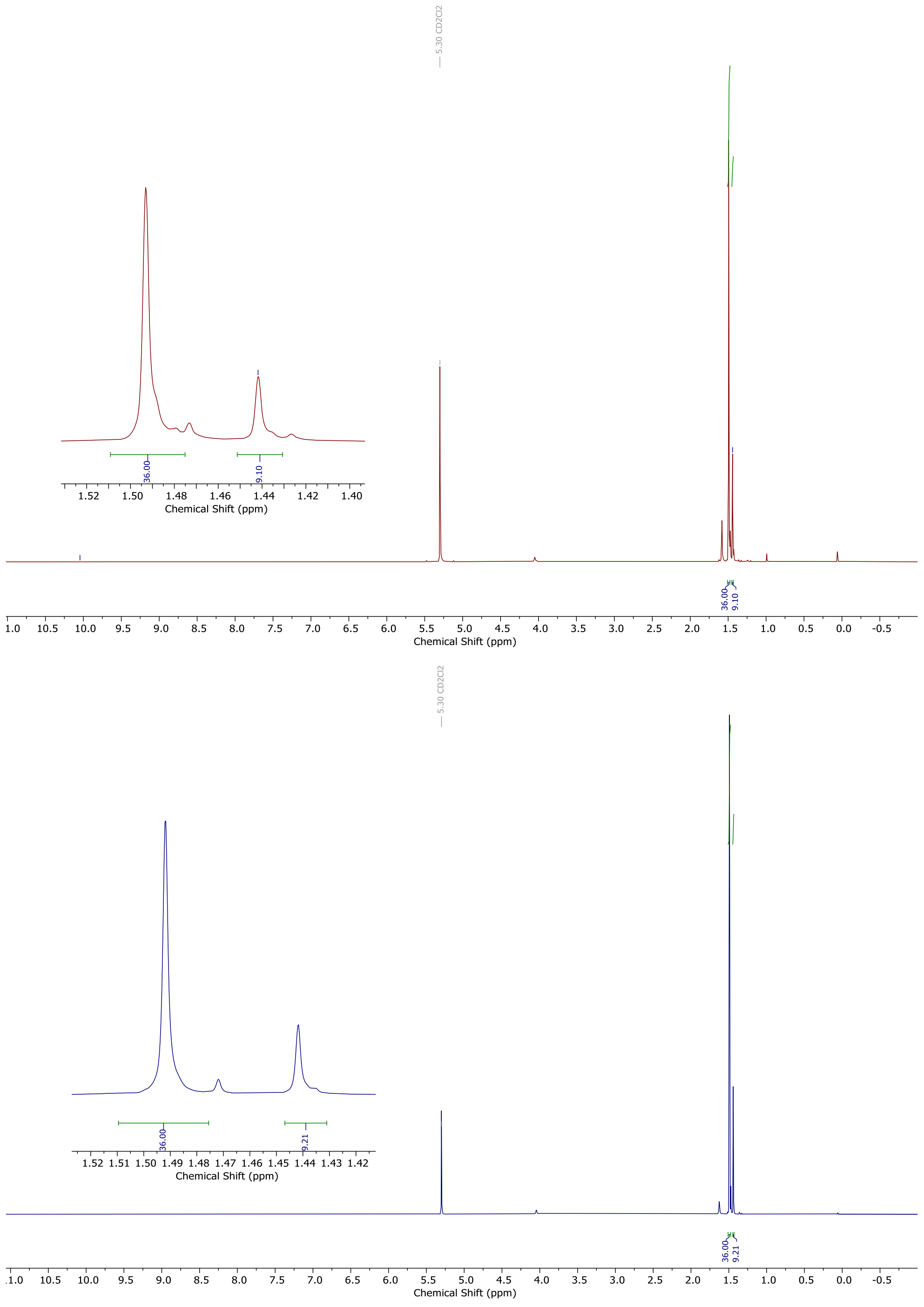

Figure 3 Air-stability of **RuAqua**. Top (red): new batch of **RuAqua**. Bottom (blue): over 1 year old batch of **RuAqua**.

Exposure of [($C_6H_4CH_2NMe_2$)Ru(MeCN)$_4$]PF_6 4 Solution to Atmosphere

In comparison, [($C_6H_4CH_2NMe_2$)Ru(MeCN)$_4$]PF_6 **4** decays when exposed to atmospheric conditions. This speed of decay is increased when in solution rapidly changing from yellow to dark blue/black. In an argon filled glovebox, **4** was placed into a small sample vial dissolved in acetone and capped before being removed. The cap was removed as the timer started. Images shown are over the course of 2 minutes. Each of the four images shown were taken approximately 30 seconds apart.

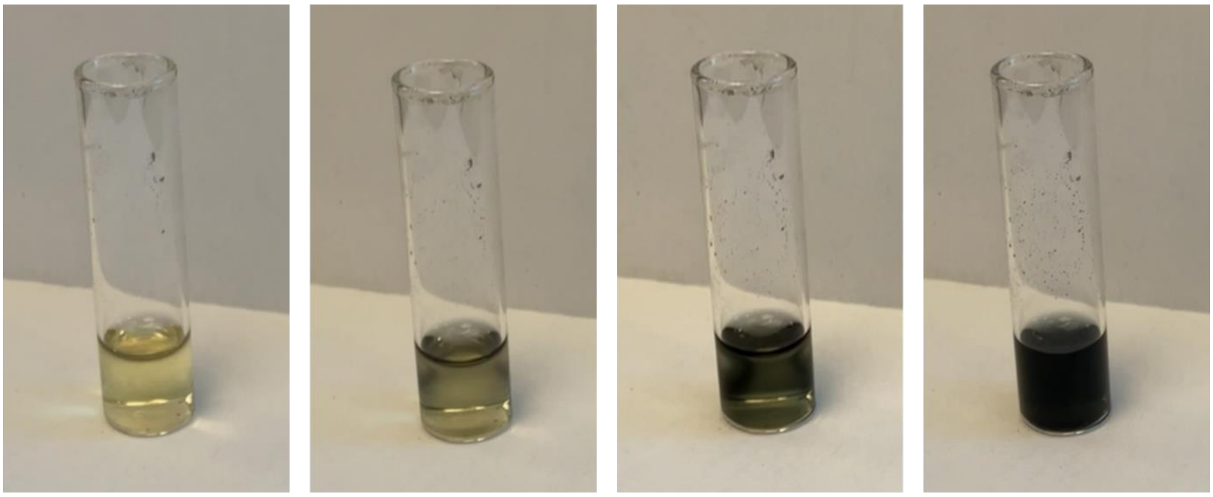

Figure 4 Decay of [($C_6H_4CH_2NMe_2$)Ru(MeCN)$_4$]PF_6 **4** in solution over the course of 2 minutes

RuAqua Catalyst Stress Test

To further assess the stability of **RuAqua**, the pre-catalyst was exposed to a variety of conditions before being used in a C–H arylation reaction. Conditions include leaving the pre-catalyst in solution or exposed to heat or light for several hours to examine how this would affect its catalytic activity. Procedure: To a microwave vial was added **RuAqua** which was then exposed to the indicated stress. After the allocated time, and removing any solvents added under reduced pressure, the catalyst was used in a test C–H arylation reaction. Reactions were set up outside of the glovebox using Schlenk technique: To a 10 mL Schlenk tube equipped with a magnetic stirrer bar was added 4-iodo-anisole (94 mg, 0.4 mmol, 1 equiv.), the indicated sample of **RuAqua** (14.2 mg, 0.02 mmol, 5 mol%), K_2CO_3 (111 mg, 0.8 mmol, 2 equiv.), KOAc (12 mg, 0.1 mol, 30 mol%). Degassed *o*-tolyl pyridine (68 mg, 0.4 mmol, 1 equiv.) and NMP (0.4 mL, 1 M with respect to **8a**) and the reaction was stirred at 40 °C for 16 h. Yields of reactions were calculated using quantitative ^{1}H NMR using nitromethane as an internal standard.

Stress tests were inspired by previously published work[1].

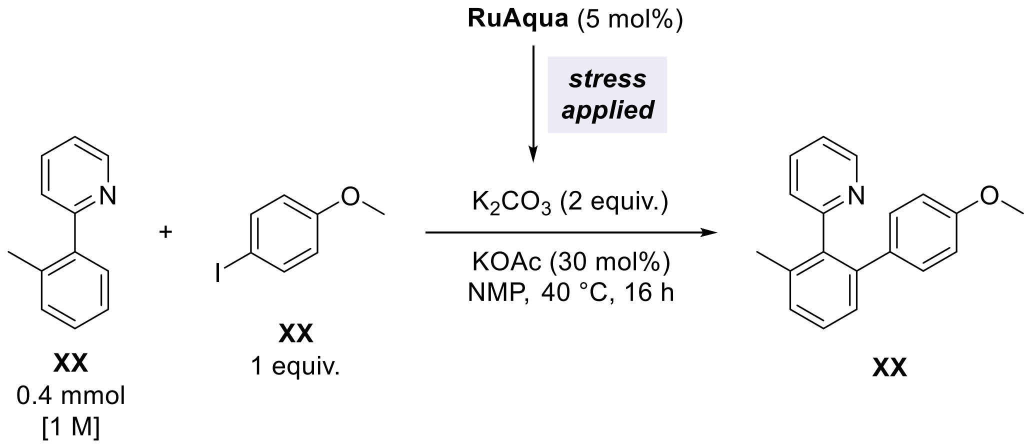

Type of Stress	Time Stress was applied (h)	Yield of 10b (%)
None (control)	N/A	99
MeOH + Air	5	89
DCM + Air	5	91
Acetone + Air	5	89
Vac Oven (80 °C)	5	92
Blue light (Kessil lamp, 440 nm) + Air	12	64

Solubility of RuAqua

Approximately 15 mg of **RuAqua** was added to a vial before adding 1 mL of the indicated solvent. The resulting solution was filtered through a small pad of celite before removing the solvent under reduced pressure and recording the mass of solid to assess to solubility of **RuAqua** in a given solvent. Results are outlined below.

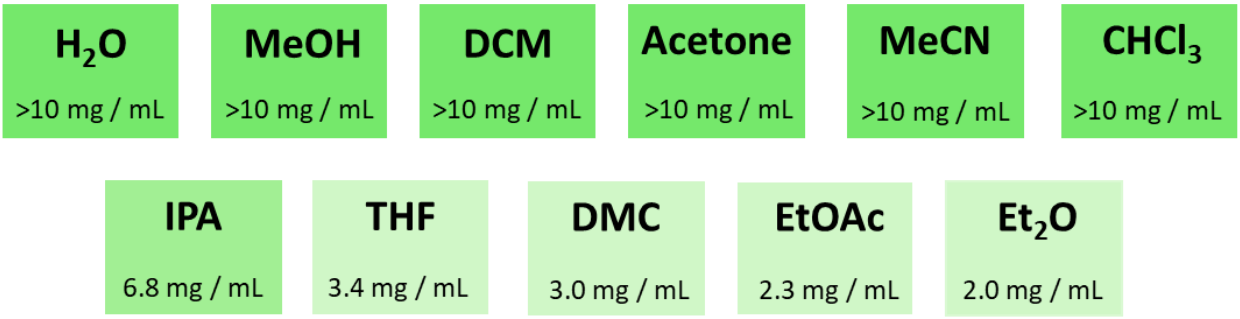

Figure 5 Solubility of **RuAqua** in various solvents

Thermogravimetric Analysis (TGA) of RuAqua

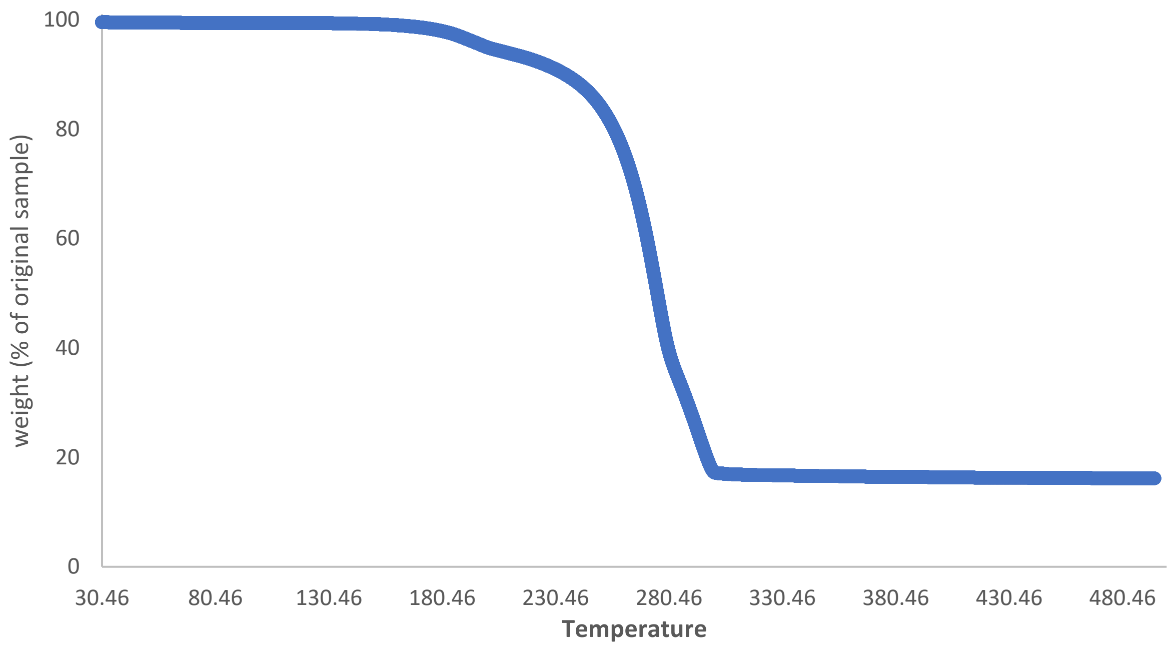

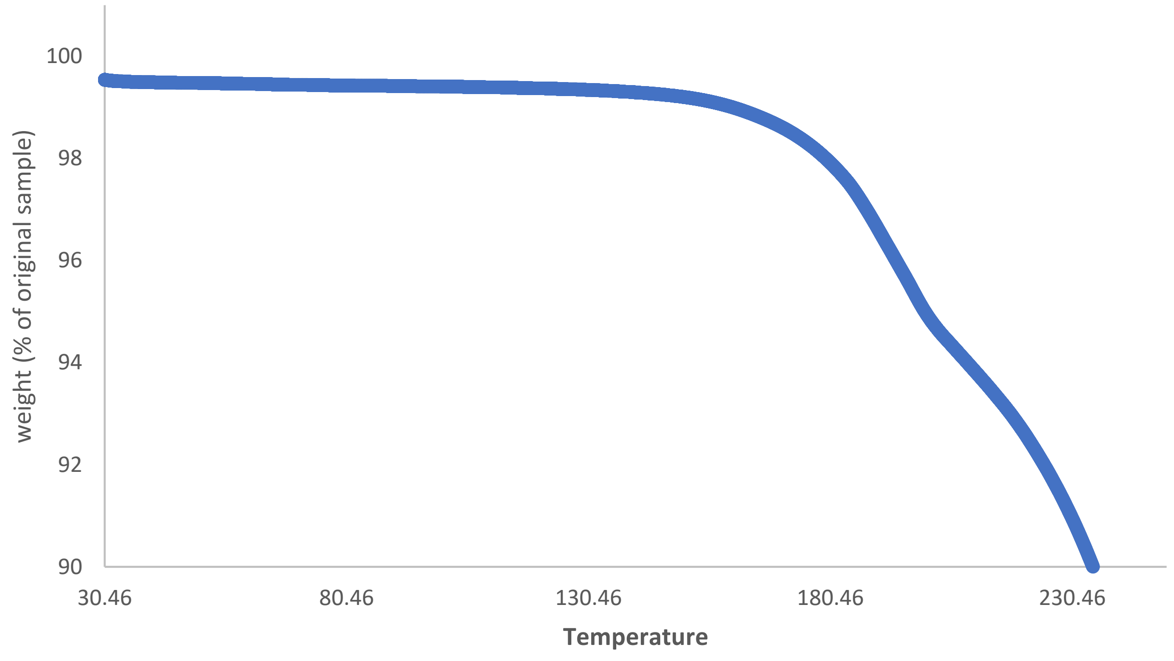

Figure 6 TGA of **RuAqua**

CV of RuAqua

Cyclic Voltammetry experiments were conducted with in a 10 mL glass vial fitted with a glassy carbon working electrode (3 mm diameter, BASi), a Ag/AgCl reference electrode and a platinum wire counter electrode. The solution of interest was purged with N_2 for 5 minutes before data collection. After data collection, ferrocene (5 mM) was added and an additional scan was run. The parent data was referenced relative to the $Fc^{+/0}$ couple that was recorded.

$E_{p/2}$ = 1.0 V

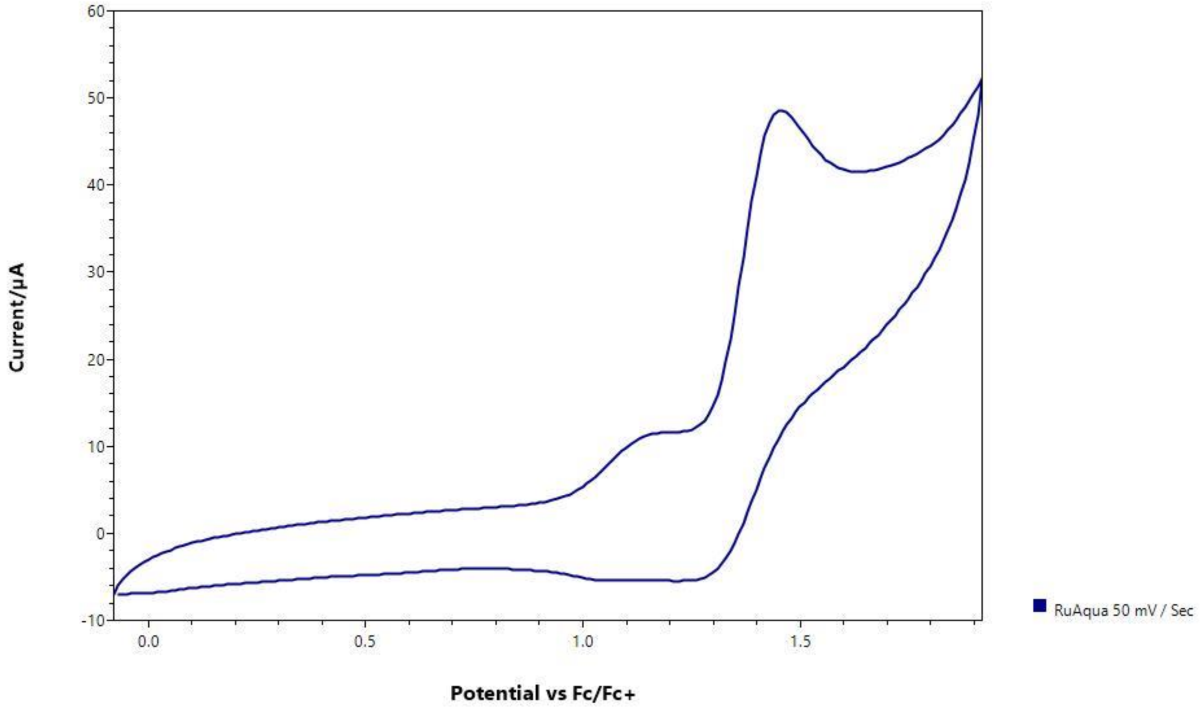

Figure 7 Cyclic voltammogram of **RuAqua** in MeCN. **RuAqua** (8.0 mM) and $LiClO_4$ (0.1 M). Scan rate: 50 mV / s.

UV-Vis of RuAqua

A 3 mM solution of **RuAqua** was prepared and analysed in a quartz cuvette.

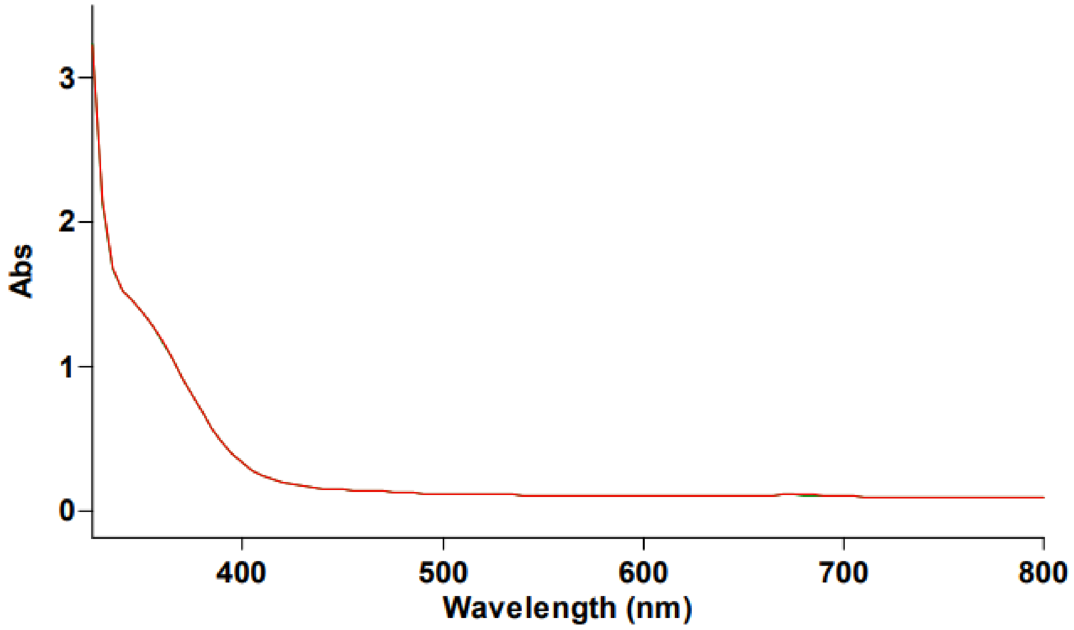

Figure 8 UV-Vis of **RuAqua**

Cost of RuAqua Synthesis

Pricing is calculated using costs of materials purchased for 26.3 g **RuAqua** synthesis on 06.04.2023.

Table 2: Cost of **RuAqua** Synthesis

Reagent	Purchased From	Price / Bottle (£)	Price / Amount Used (£)
**$RuCl_3$•H_2O**	Fischer	1089.44 for 100 g	217.89 for 20 g
**^{t}BuCN**	TCI	266.00 for 500 mL	244.72 for 460 mL
**Zn dust (>10 nm)**	Merck	7.70 for 100 g	1.54 for 20 g
**$AgBF_4$**	Fluorochem	149.60 for 100 g	55.95 for 37.4 g
**Water (HPLC grade)**	Merck	3.94 for 2.5 L	3.15 for 2 L
**Other Solvents (Et_2O, CH_2Cl_2, Acetone)**	Fischer	-	Approx. 18
		**Total for 26.3 grams**	541.25
		**Cost / gram**	20.58

When the pivalonitrile is recycled (90% recovery) the cost can be brought down to approximately £12.20 / gram of ***RuAqua****.*

Trace Metal Analysis of RuAqua

Method is based on BS EN 16965:2013 for Trace Metals in Soils. This method uses a 5 point linear calibration with a 99.999% minimum regression, with an internal standard to correct for instrument drift and matrix interferences. Results are quantified against this curve in mg / L, or ppm, at a limit of detection of 0.005 mg / L, any figures below this should be noted as being below the limit of detection (<LOD); including any quantities below zero, as the instrument below these levels cannot discern peaks from background noise. Each sample was measured three times, and an average of these replicates is calculated by the instrument. The %RSD variance between each replicate is less than 2.5%.

Table 3 Trace metal analysis of **RuAqua**

Trace Element Identity	Concentration (Mg / L)	Concentration (ppm)
**Rh 103**	0.006	3.146
**Re 187**	0.000	0.085
**Au 197**	0.033	17.599
**Ir 193**	0.024	12.742
**Pt 195**	0.001	0.460
**Pd 106**	0.112	59.432
**Zn 66 Helium KED**	0.177	94.030
**Co 59 Helium KED**	0.002	1.273
**Ni 60 Helium KED**	0.009	4.752
**Ag 107**	0.021	11.017
**Cu 63 Helium KED**	0.013	6.979

Synthesis and Application of RuAqua with Alternative Counterions

$$\underset{\text{3.8 mmol}}{RuCl_3{\bullet}H_2O} \xrightarrow[\text{ii) AgOTf, } H_2O\text{, rt, 1 hour}]{\text{i) Zn dust, }^{t}BuCN\text{, 115 °C, 2 h}} \underset{\substack{8\% \\ \textbf{RuAqua-OTf}}}{[Ru(H_2O)(^{t}BuCN)_5](OTf)_2}$$

Reaction was set up in an argon filled glovebox. The General Procedure A was applied using $RuCl_3 \bullet xH_2O$ (1.0 g, 3.8 mmol, 1 equiv.), zinc dust (1.0 g, 15.3 mmol, 4.0 equiv.) and pivalonitrile (23 mL). After allowing the reaction mixture to stir and removing the pivalonitrile under reduced pressure, HPLC grade water (100 mL) was added and the resulting mixture was filtered through a small plug of celite ensuring all solids were removed. AgOTf (2.4 g, 9.5 mmol, 2.5 equiv.) was added and the resulting solution and was allowed to stir at room temperature for 1 hour. The product was precipitated with Et_2O from a solution in CH_2Cl_2 3 times giving **RuAqua-OTf** as a light-yellow powder (262 mg, 8% yield).

*The synthesis of **RuAqua** with various other counterions, including PF_6, SbF_6 and NO_3 was also attempted using the above method but was unsuccessful.*

^{1}H NMR: (400 MHz, CD_2Cl_2)

1.53 (s, 36H), 1.48 (s, 9H).

^{13}C NMR: (101 MHz, CD_2Cl_2)

135.8, 134.0, 122.7 (q, J = 320.1 Hz), 31.1, 30.8, 28.8, 28.4.

^{19}F NMR: (376 MHz, CD_2Cl_2)

-76.84, -76.85.

MS: HRMS (ESI$^+$)

Calculated for $[Ru(H_2O)(^tBuCN)_5]^+(OTf)$: 684.2339, found: 684.2337.

IR: V_{max} (neat, cm^{-1})

3327 (br), 2981, 2940, 2898, 2879, 1278, 1170, 1027, 636.

mp: 140–144 °C (decomp., CH_2Cl_2).

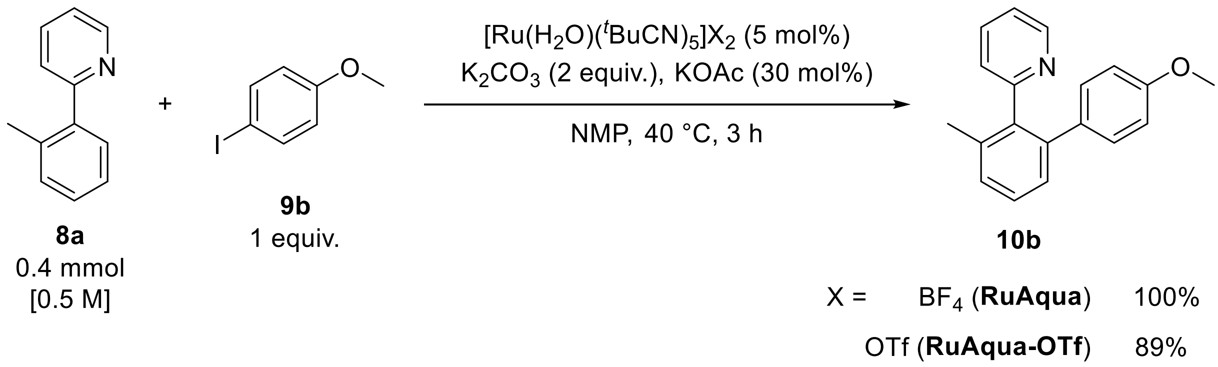

Experimental Procedure:

Reactions were set up outside of the glovebox using Schlenk technique. To a 10 mL Schlenk tube equipped with a magnetic stirrer bar was added 4-iodo-anisole **9b** (94 mg, 0.4 mmol, 1 equiv.), K_2CO_3 (111 mg, 0.8 mmol, 2 equiv.), KOAc (12.0 mg, 0.12 mmol, 30 mol%) and either **RuAqua** (14.2 mg, 0.02 mmol, 5 mol%) or **RuAqua-OTf** (16.7 mg, 0.02 mmol, 5 mol%) before performing 3 x 5 min evac-refill cycles using nitrogen. Degassed 2-(*o*-tolyl)pyridine **8a** (68 mg, 0.4 mmol, 1 equiv.) and NMP (0.8 mL, 0.5 M with respect to **8a**) was then added and the reaction was allowed to stir for 3 hours at 40 °C. Yields were analysed by quantitative ^{1}H NMR using 1,3,5-trimethoxybenzene as an internal standard. **RuAqua** bearing BF_4 counterions gave 100% conversion while **RuAqua-OTf** gave 89% conversion.

Formation of Biscyclometallated Intermediate 7 from RuAqua

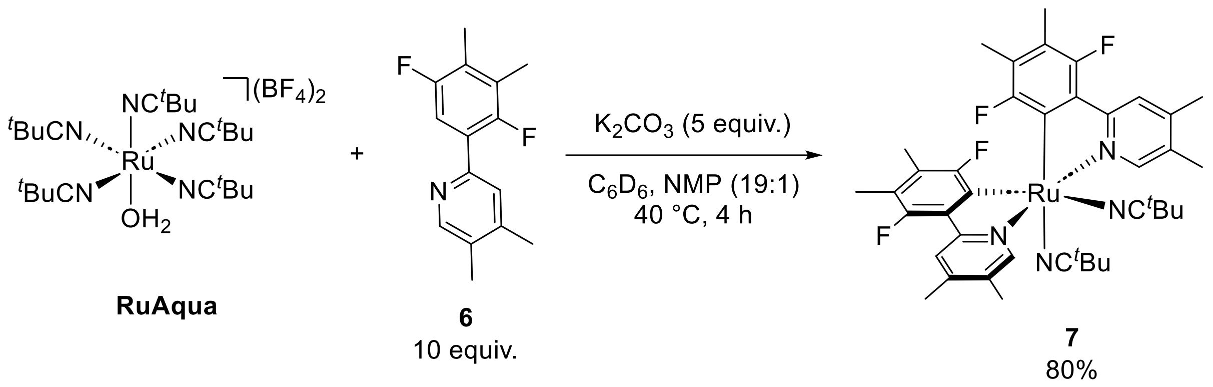

Reaction was set up in an argon filled glovebox: To a microwave vial fitted with a magnetic stirrer bead was added **RuAqua** (14.2 mg, 0.02 mmol, 1 equiv.), phenylpyridine derivative **6** (49 mg, 0.20 mmol, 10 equiv.), K_2CO_3 (14 mg, 0.10 mmol, 5 equiv.) and a stock solution of 1,4-difluorobenzene (0.04 mmol in 0.1 mL of C_6D_6:NMP (19:1)). A further 0.3 mL of C_6D_6:NMP (19:1) was added to the microwave vial and the resulting mixture was capped and left to stir at 40 °C for 4 hours. After this time, quantitative ^{19}F NMR indicated conversion of 80% to the biscyclometallated species **7**. HRMS (ESI$^+$) also indicated formation of species **7**. Due to the large excess of phenylpyridine derivative **6**, peaks for the resulting product were not overly clear by ^{1}H NMR.

To obtain ^{1}H and ^{13}C NMR, the reaction was repeated on twice the scale using 2 equivalents of phenylpyridine derivative **6** instead of 10 equivalents. 1,4-difluorobenzene was omitted from this reaction as the goal was simply to collect further characterisation for intermediate **6** rather than measure conversion.

^{1}H NMR: (400 MHz, C_6D_6)

9.25 (s, 2H), 8.46 (s, 2H) (Peaks from ^{t}BuCN ligands obscured by NMP in reaction mixture).

^{13}C NMR: (176 MHz, C_6D_6)

170.7 (d, *J* = 55.3 Hz), 167.4 (d, *J* = 224.4 Hz), 164.8 (d, *J* = 7.5 Hz), 156.5 (d, *J* = 247.1 Hz), 151.1, 141.7, 133.2 (dd, *J* = 21.6, 5.1 Hz), 128.4, 127.3, 123.2 (d, *J* = 21.6 Hz), 122.1 (dd, *J* = 26.7, 4.5 Hz), 113.7 (dd, *J* = 19.9, 3.7 Hz), 28.1, 27.7, 19.3, 16.5, 11.8 (d, *J* = 4.5 Hz), 10.9 (d, *J* = 7.0 Hz).

^{19}F NMR: (376 MHz, C_6D_6)

-107.5 (d, *J* = 21.5 Hz), -125.5 (d, *J* = 20.8 Hz).

MS: HRMS (ESI^+)

Calculated for $[Ru(C_{15}H_{15}F_2N)_2(^tBuCN)_2]$: 760.2702, found: 760.2686.

Data is consistent with analogous species in the literature[2].

^{19}F NMR (376 MHz, C_6D_6) of reaction mixture (10 equiv. of 6 used):

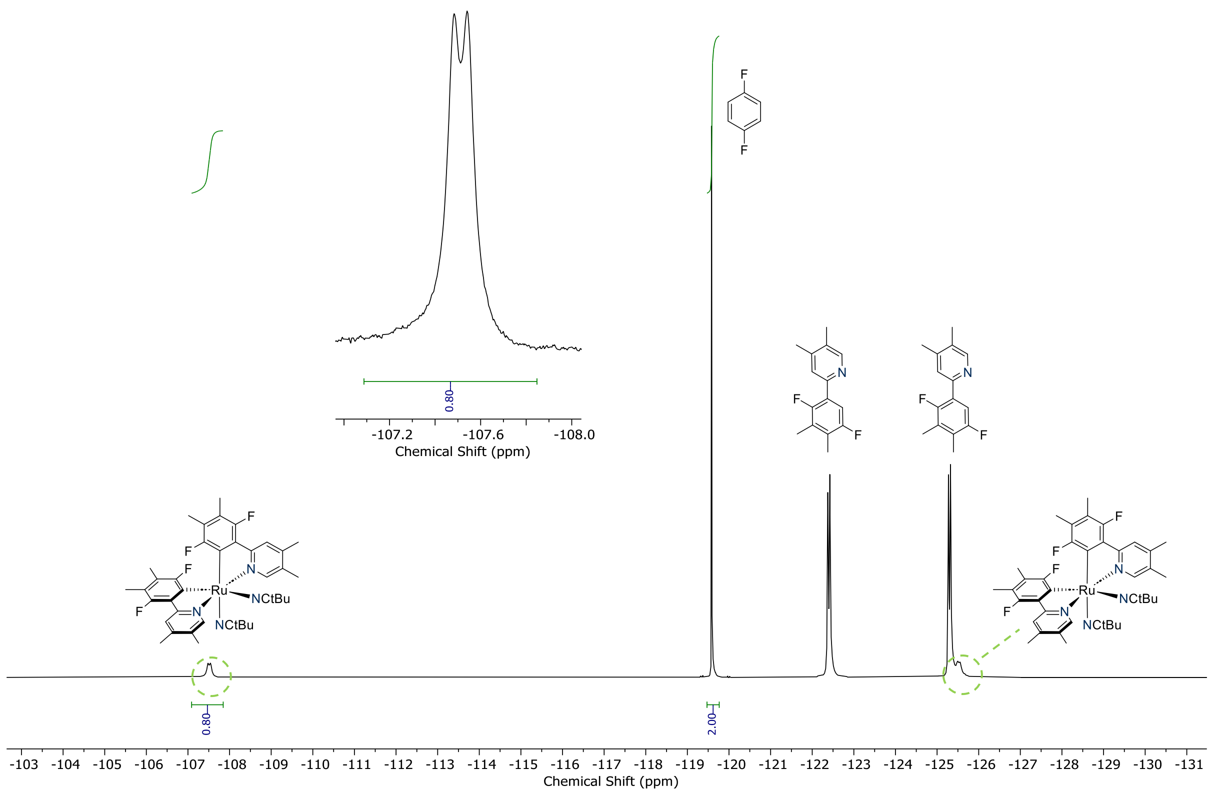

^{1}H NMR (400 MHz, C_6D_6) of reaction mixture (2 equiv. of 6 used):

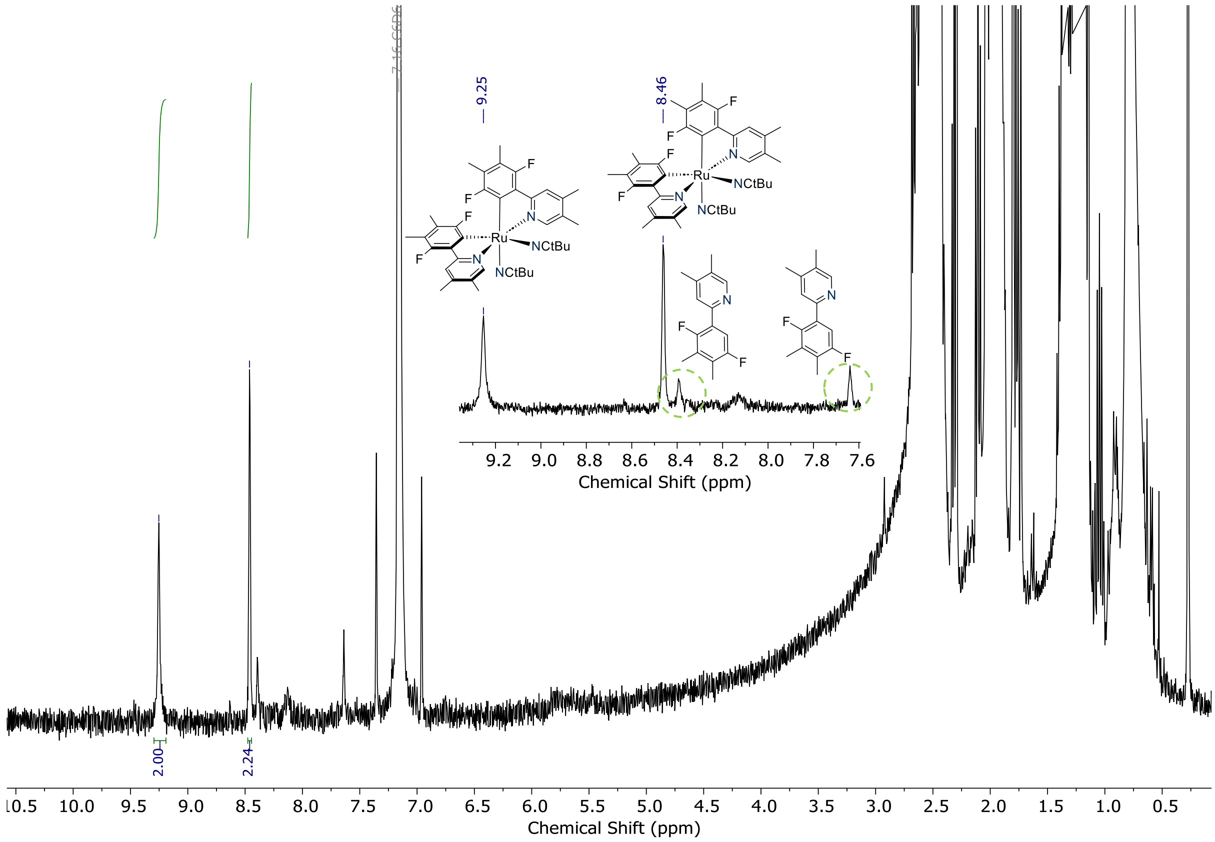

^{13}C NMR (176 MHz, C_6D_6) of reaction mixture (2 equiv. of 6 used):

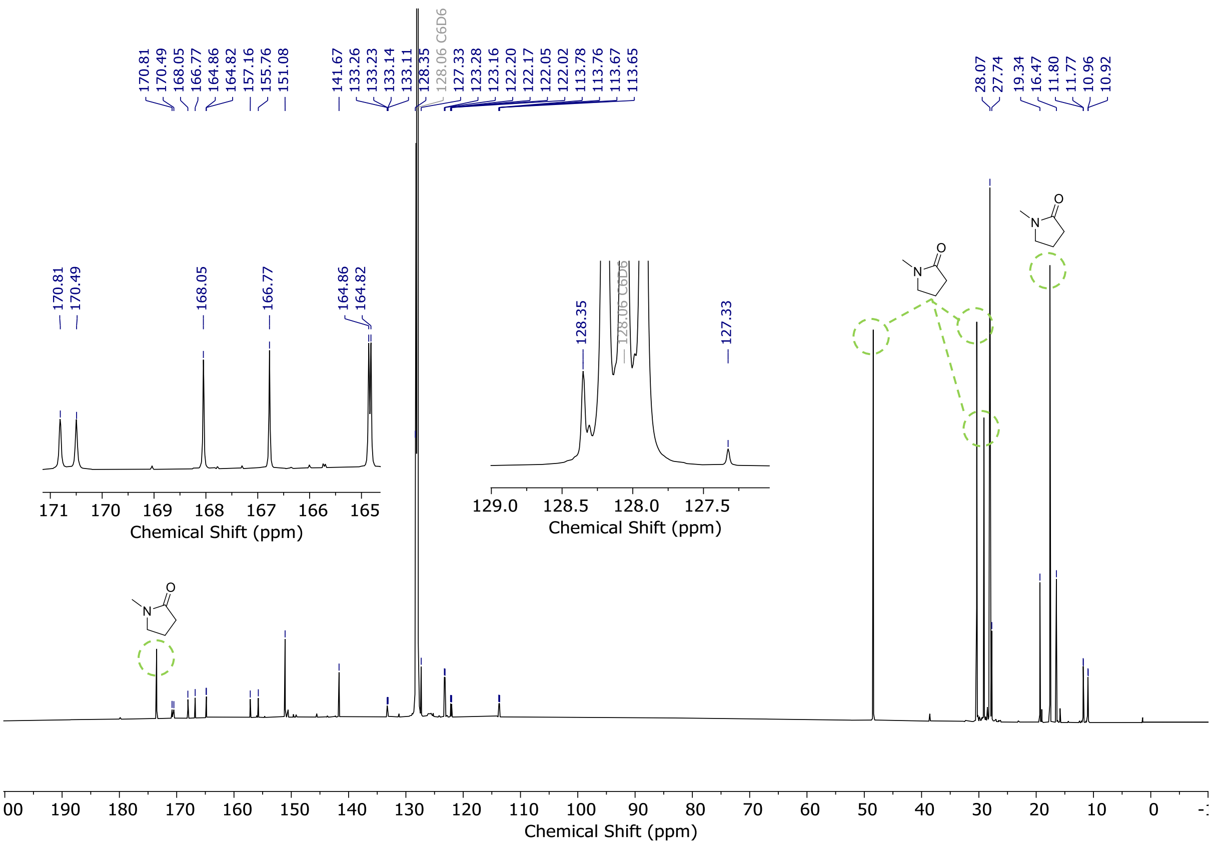

^{19}F NMR (659 MHz, C_6D_6) of reaction mixture (2 equiv. of 6 used):

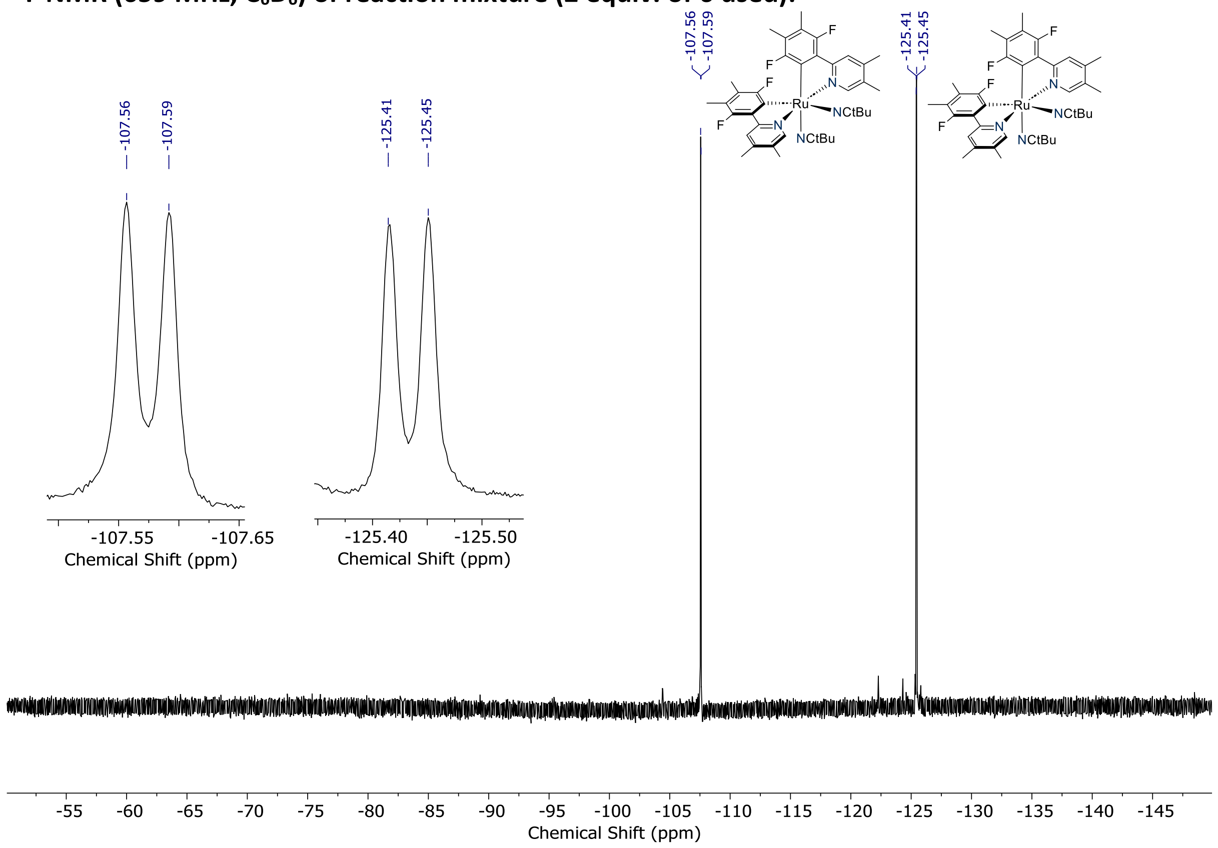

Comparison of RuAqua with [Ru(^{t}BuCN)$_6$](BF$_4$)$_2$ and [Ru(*p*-cymene)Cl$_2$]$_2$

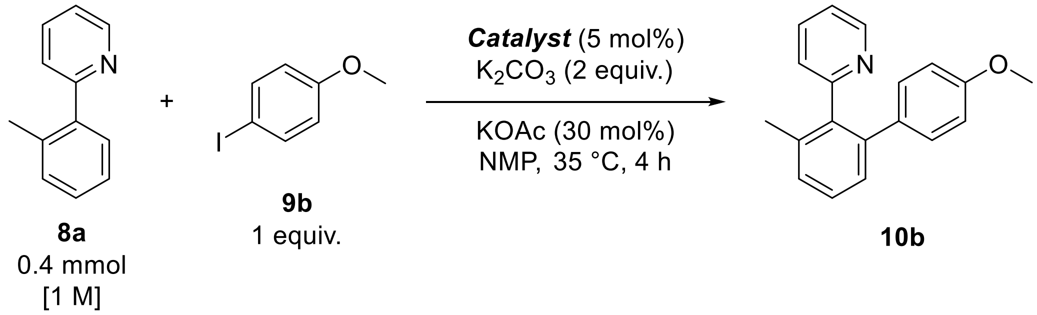

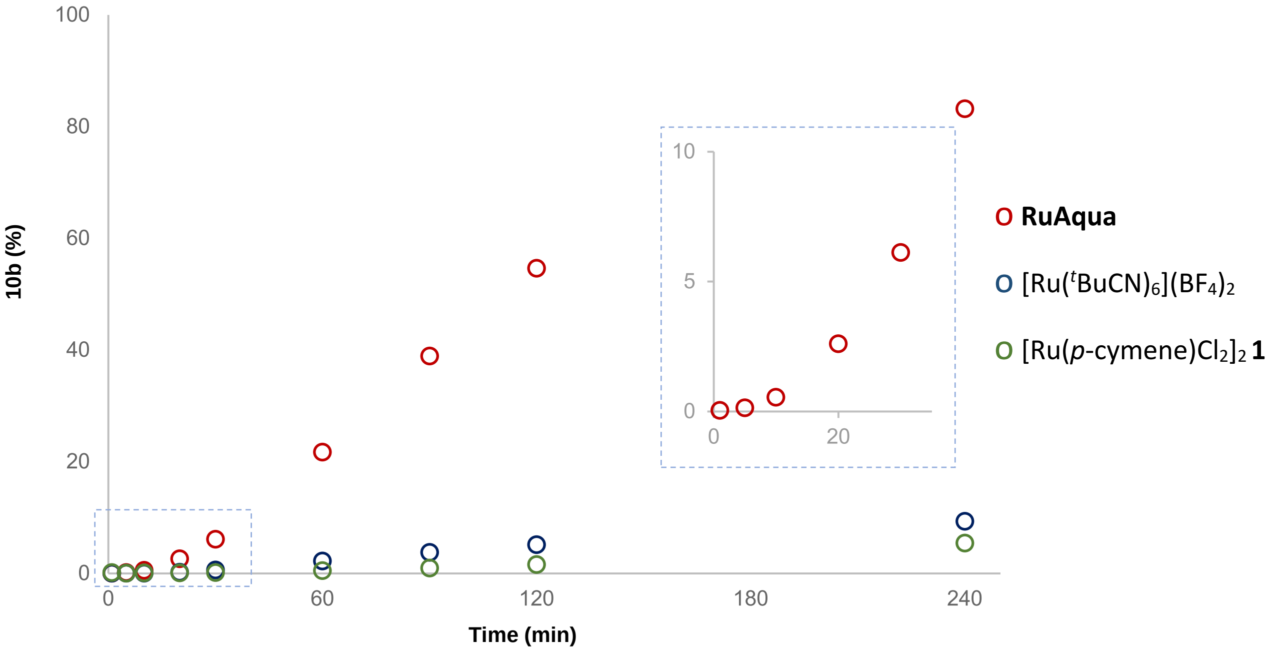

Figure 9 Comparison of **RuAqua** with other air-stable catalysts.

Reaction was set up in an argon filled glovebox: K_2CO_3 (111 mg, 0.8 mmol, 2.0 equiv.) and KOAc (12 mg, 0.12 mmol, 0.3 equiv.) were weighed into a microwave vial equipped with a magnetic stirrer bar. A stock solution of 2-(*o*-tolyl)pyridine **8a** (558 mg, 3.3 mmol), 4-iodo-anisole **9b** (772 mg, 3.3 mol) and internal standard biphenyl (35 mg, 0. 2 mmol) was prepared in NMP (5 mL) and 0.6 mL of this stock solution was added to the microwave vial *via* syringe. This mixture was allowed to stir at 35 °C for 10 minutes before adding the indicated catalyst as a stock solution in NMP (0.02 mmol in 0.2 mL). As this was added the reaction clock started at 0 minutes. Aliquots of approximately 20 µL were then taken throughout the first 4 h of the reaction at specified time points. Each aliquot was added to approximately 1 mL of a solution of 1% pyridine in Et_2O (v/v), before being passed through a short plug of silica into a GC vial ready for analysis. The reaction was then monitored by GC-FID.

Characterisation for 2-(4'-methoxy-3-methyl-[1,1'-biphenyl]-2-yl)pyridine 10b

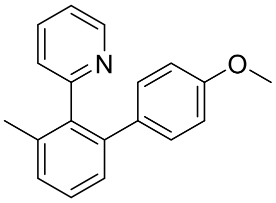

10b

Pure product was isolated for GC-FID calibration via column chromatography eluting with 5–15% EtOAc in hexane to give the product **10b** as an off white wax.

^{1}H NMR: (500 MHz, $CDCl_3$)

8.56 (ddd, *J* = 4.9, 1.9, 1.0 Hz, 1H), 7.38 (td, *J* = 7.7, 1.8 Hz, 1H), 7.26 (t, *J* = 7.6 Hz, 1H), 7.20 – 7.15 (m, 2H), 7.01 (ddd, *J* = 7.6, 5.0, 1.2 Hz, 1H), 6.93 – 6.88 (m, 2H), 6.82 – 6.78 (m, 1H), 6.62 – 6.57 (m, 2H), 3.65 (s, 3H), 2.09 (s, 3H).

^{13}C NMR: (126 MHz, $CDCl_3$)

159.9, 158.2, 149.0, 140.9, 139.5, 136.8, 135.9, 134.2, 130.8, 129.2, 128.1, 127.7 125.7, 121.4, 113.2, 55.2, 20.6.

IR: V_{max} (neat/cm^{-1})

3059, 2998, 2955, 2930, 2834, 1608, 1584, 1510, 1457, 1244, 1177, 1028, 832, 791, 748.

Spectroscopic data matched those previously reported[2].

RuAqua catalysed C–H Arylation of Arenes with Aryl (Pseudo)Halides

Attainment of Starting Materials:

Directing Group Substrates:

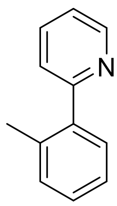

8a

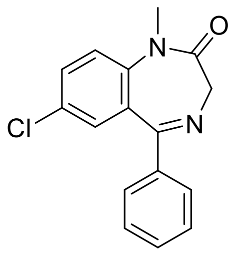

8b
diazepam

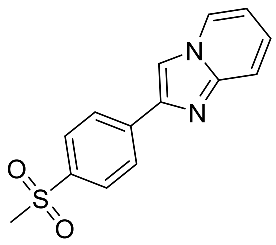

8c
zolimidine

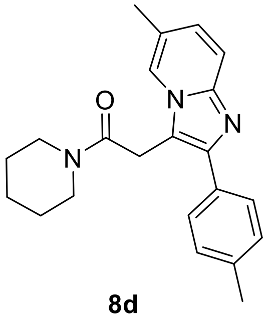

8d
zolpidem derivative

Compound **8b** was commercially available.

The following substrates were synthesized according to described procedures: **8a**[3], **8c**[4] and **8d**[5].

Preparation of Starting Material 8a

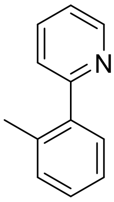

8a

Compound **8a** was synthesised according to a previously described procedure with data in accordance.[3]

^{1}H NMR: (400 MHz, $CDCl_3$)

8.58 (d, *J* = 4.9 Hz, 1H), 7.59 (td, *J* = 7.7, 2.0 Hz, 1H), 7.27 (t, *J* = 7.0 Hz, 2H), 7.20 – 7.12 (m, 3H), 7.12 – 7.07 (m, 1H), 2.25 (s, 3H).

^{13}C NMR: (126 MHz, $CDCl_3$)

160.1, 149.2, 140.5, 136.2, 135.8, 130.8, 129.7, 128.3, 125.9, 124.1, 121.7, 20.3.

Preparation of Starting Material 8c

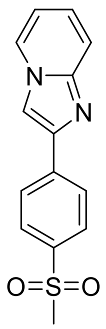

8c

Compound **8c** was synthesised according to a previously described procedure with data in accordance.[4]

^{1}H NMR: (500 MHz, $CDCl_3$)

8.20 – 8.12 (m, 3H), 8.06 – 7.96 (m, 3H), 7.68 (d, J = 9.1 Hz, 1H), 7.29 – 7.21 (m, 2H), 6.86 (t, J = 6.8 Hz, 1H), 3.09 (s, 3H).

^{13}C NMR: (126 MHz, $CDCl_3$)

145.8, 143.4, 139.4, 139.0, 128.0, 126.7, 125.9, 125.8, 117.8, 113.3, 109.6, 44.6.

Preparation of Starting Material 8d

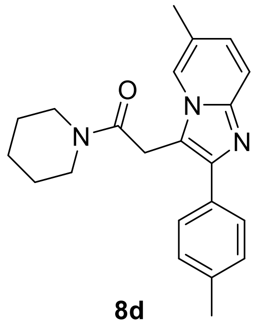

8d

Compound **8d** was synthesised according to a previously described procedure with data in accordance.[5]

^{1}H NMR: (400 MHz, $CDCl_3$)

8.06 (s, 1H), 7.57 – 7.50 (m, 3H), 7.28-7.24 (m, 2H), 7.03 (dd, J = 9.2, 1.3 Hz, 1H), 4.09 (s, 2H), 3.56 – 3.48 (m, 2H), 3.25 – 3.16 (m, 2H), 2.40 (s, 3H), 2.34 (s, 3H), 1.56 – 1.49 (m, 2H), 1.48 – 1.40 (m, 2H), 1.23 – 1.15 (m, 2H).

^{13}C NMR: (101 MHz, $CDCl_3$)

166.6, 144.3, 143.8, 137.6, 132.0, 129.4, 128.5, 127.6, 122.4, 121.8, 116.7, 113.9, 47.2, 43.4, 30.6, 26.5, 25.8, 24.4, 21.4, 18.6.

Aryl (Pseudo)Halides:

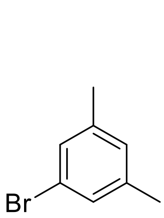

9a

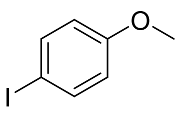

9b

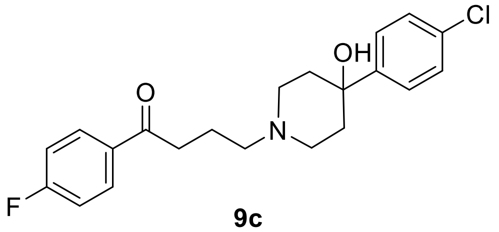

9c
haloperidol

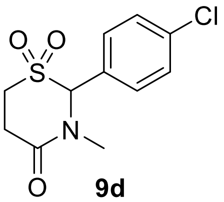

9d
chlormezanone

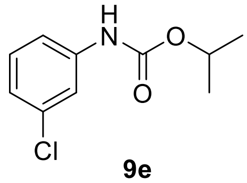

9e
chlorpropham

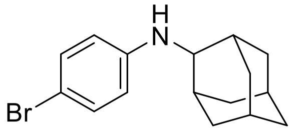

9f
ladasten

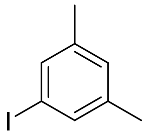

9g

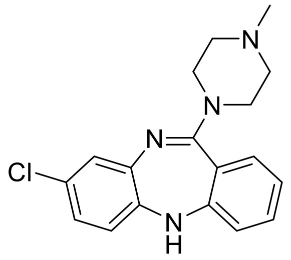

9h
clozapine

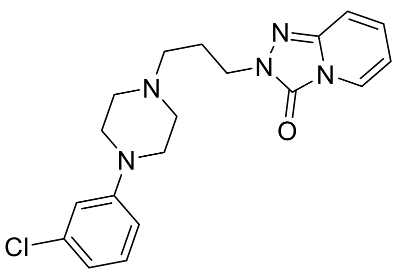

9i
trazodone

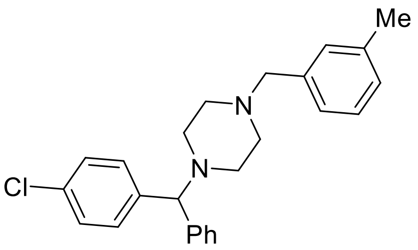

9j
meclizine

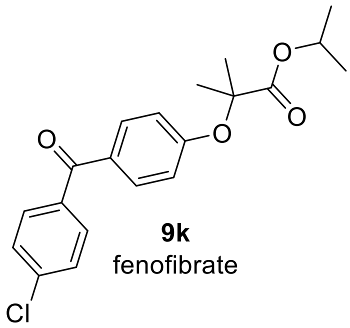

9k
fenofibrate

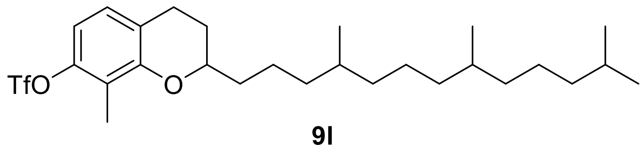

9l
from (δ)-tocopherol

Compounds **9a**, **9b**, **9c**, **9d**, **9e**, **9f**, **9g**, **9h**, **9i**, **9j** and **9k** were commercially available.

9l was prepared according to previously reported procedures[2].

Preparation of Starting Material 9l

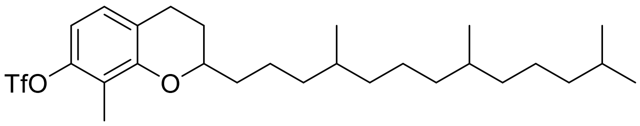

9l

Compound **9l** was synthesised according to a previously described procedure with data in accordance.[2]

^{1}H NMR: (400 MHz, $CDCl_3$)

6.88 (d, J = 2.8 Hz, 1 H), 6.83 (d, J = 2.8 Hz, 1 H), 2.71–2.84 (m, 2 H), 2.19 (s, 3 H), 1.72–1.91 (m, 2 H), 1.01–1.68 (m, 22 H), 0.84–0.94 (m, 12 H).

^{13}C NMR: (101 MHz, $CDCl_3$)

151.8, 141.6, 128.5, 121.8, 120.9, 119.2, 118.8 (q, J = 320.0 Hz), 77.0, 40.3, 39.5, 37.6, 37.6, 37.5, 37.5, 33.0, 32.8, 30.8, 28.1, 25.0, 24.6, 24.3, 22.9, 22.8, 22.6, 21.1, 19.9, 19.8, 16.3.

^{19}F NMR: (376 MHz, $CDCl_3$)

–73.1.

General Procedure B: RuAqua catalysed arylation of DG-containing arenes with aryl (pseudo)halides

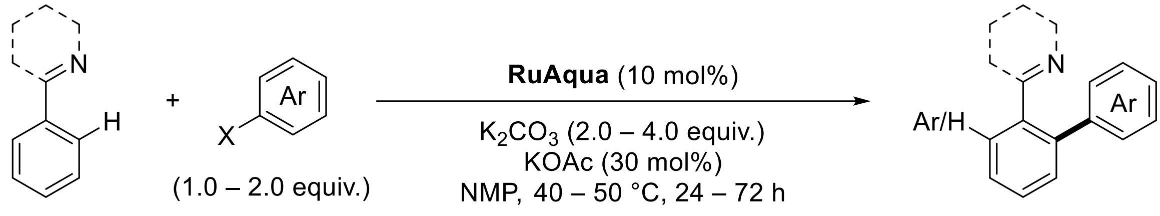

8 **9** **10/11**

Reaction set up outside of the glovebox using Schlenk technique: Reaction conditions adapted from previously reported procedures[2]. A 10 mL Schlenk tube equipped with a magnetic stirring bar was charged with **RuAqua** (28.4 mg, 0.04 mmol, 10 mol%), KOAc (12 mg, 0.12 mmol, 30 mol%), K_2CO_3 (2–4 equiv.), the appropriate DG-containing arene **8** (0.4 mmol, 1.0 equiv.), the appropriate aryl halide **9** (0.4 mmol, 1.0 equiv.) and NMP (0.8 mL, 0.5 M with respect to **8**) first adding all solids to the reaction vessel before performing 3 x 5 min evac-refill cycles with subsequent addition of oils/solvents. The reaction was then stirred at the stated temperature for the indicated time. Upon completion, the crude mixture was loaded on a silica gel column and purified by flash chromatography using the noted conditions.

Synthesis of 2-(3,3',5'-trimethyl-[1,1'-biphenyl]-2-yl)pyridine 10a

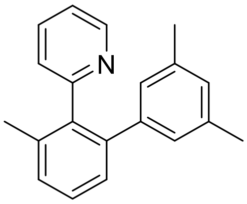

10a

Reaction set up outside of the glovebox using Schlenk technique: The general procedure B was applied using 2-(*o*-tolyl)pyridine **8a** (68 mg, 0.4 mmol, 1.0 equiv.), Br-*m*-xylene **9a** (74 mg, 0.4 mmol, 1.0 equiv.) and K_2CO_3 (111 mg,0.8 mmol, 2.0 equiv.) for 3 h at 40 °C. Column chromatography eluting with 5–15% EtOAc in hexane afforded the product **10a** as a colourless oil (101 mg, 92% yield).

^{1}H NMR: (400 MHz, $CDCl_3$)

8.68 – 8.62 (m, 1H), 7.46 (td, *J* = 7.7, 1.8 Hz, 1H), 7.35 (m, 1H), 7.37 – 7.30 (m, 2H), 7.12 – 7.06 (m, 1H), 6.92 (d, *J* = 7.7 Hz, 1H), 6.77 (s, 1H), 6.72 (s, 2H), 2.21 (s, 3H), 2.16 (s, 6H).

^{13}C NMR: (101 MHz, $CDCl_3$)

159.9, 148.7, 141.5, 141.5, 139.4, 137.0, 136.6, 135.7, 129.3, 128.0, 127.9, 127.9, 127.6, 125.7, 121.2, 21.2, 20.6.

IR: V_{max} (neat/cm^{-1})

3048, 2916, 2829, 1582, 1459, 1026, 790, 754, 704.

Spectroscopic data matched those previously reported[2].

Large Scale Synthesis of 2-(4'-methoxy-3-methyl-[1,1'-biphenyl]-2-yl)pyridine 10b Carried Out at AstraZeneca

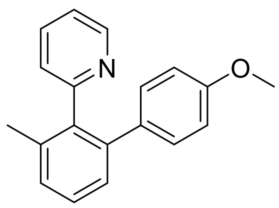

10b

Reaction set up outside of the glovebox using purging with N_2 to achieve an inert atmosphere: The following reaction was conducted within the research laboratory of AstraZeneca, Macclesfield, UK. 2-(*o*-tolyl)pyridine **8a** and **RuAqua** were prepared at the University of Manchester. All other reagents were purchased from the following supplier and used without purification. TBAOAc (Fluorochem, ~90% containing AcOH, used without adjusting for purity), K_2CO_3 (Sigma-Aldrich, powered), 4-iodo-anisole **9b** (Sigma-Aldrich) and dimethyl carbonate (Sigma-Aldrich). The reaction was conducted using a Easymax-402 (Mettler Toledo) fitted with a 100 mL reaction insert. A 1-piece glass reactor set fitted with a condenser, oxygen sensor and overhead stirrer was used to ensure adequate exclusion of oxygen and mixing representative of a larger scale vessel (**Fig S10**, A).

To the reaction vessel was added the solid reagents TBAOAc (2.67 g, 8.86 mmol, 30 mol%), K_2CO_3 (12.3 g, 89.0 mmol, 3.00 equiv.), 4-iodo-anisole **9b** (7.26 g, 31.0 mmol, 1.05 equiv.) and **RuAqua** (523 mg, 0.74 mmol, 2.50 mol%) (**Fig S10**, B). The reactor was flushed with N_2 to <0.05% with a high N_2 flow, before reducing the N_2 flow and partially submerging the outlet tube in water to create a positive backpressure within the reactor. Dimethyl carbonate (59 mL, 2.0 L/mol), that had previously been degassed via N_2 bubbling, was added via syringe and the stirrer set to 400 rpm to ensure good mixing of the heterogenous pale yellow mixture. 2-(*o*-tolyl)pyridine **8a** (5.00 g, 29.5 mmol, 1.00 equiv.) that had been degassed via N_2 bubbling, was added directly via syringe and the reaction became a dark red/brown heterogenous mixture. The jacket temperature was set to 50 °C providing a steady internal reaction temperature of 49–50 °C during the course of the reaction. After 5.5 h it was determined that the reaction had stalled (two consecutive UHPLC MS samples 1 h apart with no change in profile) and additional **RuAqua** (200 mg, 0.28 mmol, 1.00 mol%) was added via syringe as a slurry in 2 mL of degassed dimethyl carbonate. The reaction was stirred for a further 16.5 h (overnight) upon which UHPLCMS indicated full consumption of the 2-(*o*-tolyl)pyridine (**Fig S10**). The dark red/brown heterogenous mixture (**Fig S10**, C) was diluted with MTBE (50 mL) and filtered through a ~100 mm diameter filter containing ~30 mm of silica. The reaction vessel was washed out with MTBE (20 mL) through the filter then the filter finally flushed with MTBE (100 mL) to give a dark green/black filtrate

(**Fig S10**, D). The filtrate was concentrated to a dark green/black oil under reduced pressure to give a crude solution of 2-(4'-methoxy-3-methyl-[1,1'-biphenyl]-2-yl)pyridine **10b** (9.29 g, 67.9 w/w%, 22.9 mmol, 78% yield). The purity was calculated via ^{1}H NMR vs 1,2,4,5-Tetrachloro-3-nitrobenzene and is the average of two samples. ^{1}H NMR is in accordance with that previously reported, containing 4-iodoanisole and dimethyl carbonate.

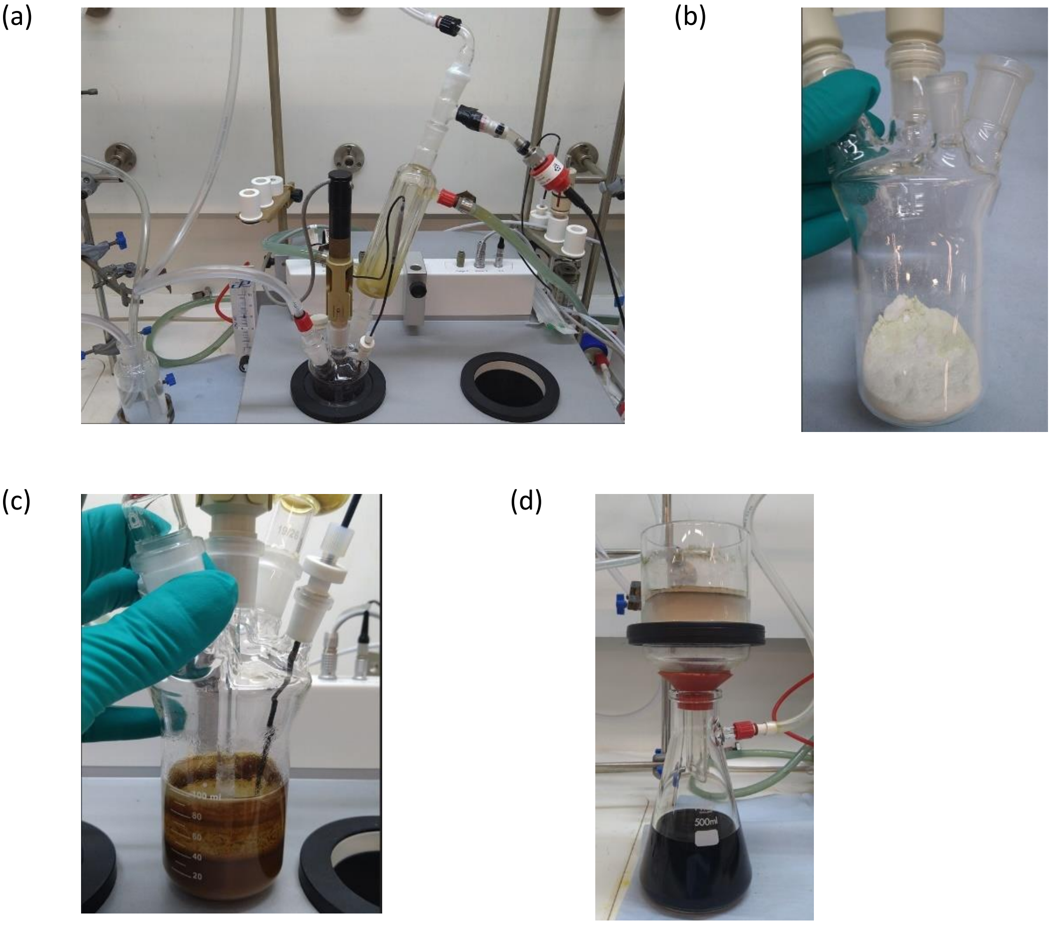

Figure 10 Use of **RuAqua** for C–H Arylation at AstraZeneca (a) Reaction set up (b) Solids in 1-piece glass vessel (c) Reaction mixture after 22 h prior to filtration (d) Filter and collected filtrate.

Synthesis of [2-(*o*-tolyl)pyridine]–[haloperidol] 10c

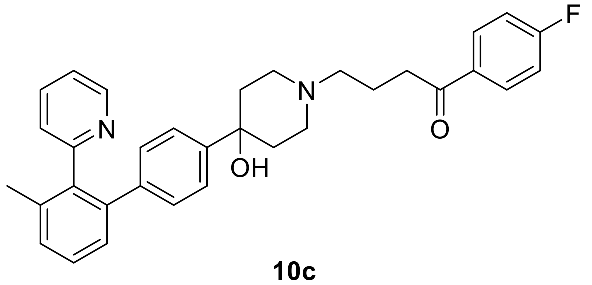

10c

Reaction set up outside of the glovebox using Schlenk technique: The general procedure B was applied using 2-(*o*-tolyl)pyridine **8a** (68 mg, 0.4 mmol, 1.0 equiv.), Haloperidol **9c** (150 mg, 0.4 mmol, 1.0 equiv.) and K_2CO_3 (111 mg, 0.8 mmol, 2.0 equiv.) at 40 °C for 48 h. Column chromatography eluting with 1–5% MeOH in CH_2Cl_2 afforded the product **10c** as a colourless amorphous solid (168 mg, 83%).

^{1}H NMR: (500 MHz, $CDCl_3$)

8.60 (dd, *J* = 5.0, 0.8 Hz, 1H), 8.03 – 7.97 (m, 2H), 7.44 (td, *J* = 7.7, 1.8 Hz, 1H), 7.35 (t, *J* = 7.6 Hz, 1H), 7.30 – 7.28 (m, 1H), 7.25 – 7.23 (m, 1H), 7.20 (d, *J* = 8.5 Hz, 2H), 7.15 – 7.07 (m, 3H), 7.03 (d, *J* = 8.4 Hz, 2H), 6.87 (d, *J* = 7.8 Hz, 1H), 3.00 (t, *J* = 7.0 Hz, 2H), 2.91 – 2.75 (m, 2H), 2.63 – 2.41 (m, 4H), 2.16 (s, 3H), 2.11 – 1.96 (m, 4H), 1.66 (d, *J* = 12.2 Hz, 2H).

^{13}C NMR: (126 MHz, $CDCl_3$)

197.9, 165.7 (d, *J* = 254.8 Hz), 159.4, 148.7, 145.7, 140.7, 140.2, 139.2, 136.8, 136.0, 133.3, 130.7 (d, *J* = 9.3 Hz), 129.5, 129.4, 128.2, 127.7, 125.7, 123.9, 121.6, 115.7 (d, *J* = 21.5 Hz), 70.2, 57.4, 49.2, 37.2, 36.0, 20.7, 20.5.

^{19}F NMR: (376 MHz, $CDCl_3$)

-105.3.

IR: V_{max} (neat/cm^{-1})

3333 (br), 3059, 2980, 2818, 1682, 1594, 1459, 1229.

m.p: 140–143 °C (MeOH)

Spectroscopic data matched those previously reported[2].

Synthesis of [2-(*o*-tolyl)pyridine]–[chlormezanone] 10d

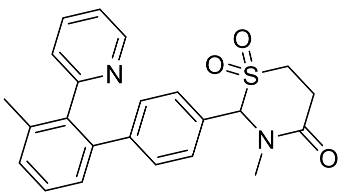

10d

a) Reaction set up outside of the glovebox using Schlenk technique: The general procedure B was applied using 2-(*o*-tolyl)pyridine **8a** (68 mg, 0.4 mmol, 1.0 equiv.), Chlormezanone **9d** (109 mg, 0.4 mmol, 1.0 equiv.) and K_2CO_3 (111 mg, 0.8 mmol, 2.0 equiv.) at 40 °C for 24 h. Column chromatography eluting with 80–90% EtOAc in hexane afforded the product **10d** as an off-white wax (146 mg, 90%).

b) Reaction was set up in a microwave vial in an argon filled glovebox: The general procedure B was applied using $RuCl_3$ (8.1 mg, 0.04 mmol, 10 mol%) in place of **RuAqua**, 2-(*o*-tolyl)pyridine **8a** (68 mg, 0.4 mmol, 1.0 equiv.), Chlormezanone **9d** (109 mg, 0.4 mmol, 1.0 equiv.) and K_2CO_3 (111 mg, 0.8 mmol, 2.0 equiv.). After stirring for 24 hours at 40 °C, the crude reaction mixture was analysed by quantitative ^{1}H NMR using nitromethane as an internal standard indicating 1% conversion to the product **10d**.

c) Reaction was set up in a microwave vial in an argon filled glovebox: The general procedure B was applied using [Ru(*p*-cymene)Cl_2]$_2$ (12.4 mg, 0.02 mmol, 10 mol% with respect to Ru) in place of **RuAqua**, 2-(*o*-tolyl)pyridine **8a** (68 mg, 0.4 mmol, 1.0 equiv.), Chlormezanone **9d** (109 mg, 0.4 mmol, 1.0 equiv.) and K_2CO_3 (111 mg, 0.8 mmol, 2.0 equiv.). After stirring for 24 hours at 40 °C, the crude reaction mixture was analysed by ^{1}H NMR using nitromethane as an internal standard indicating 25% conversion to the product **10d**.

^{1}H NMR: (400 MHz, $CDCl_3$)

8.60 (d, *J* = 5.4 Hz, 1H), 7.44 (t, *J* = 6.7 Hz, 1H), 7.41 – 7.29 (m, 2H), 7.29 – 7.24 (m, 2H), 7.21 – 7.05 (m, 5H), 6.88 (d, *J* = 7.8 Hz, 1H), 5.17 (d, *J* = 2.3 Hz, 1H), 3.31 – 2.91 (m, 4H), 2.87 (s, 3H), 2.19 (s, 3H).

^{13}C NMR: (101 MHz, $CDCl_3$)

166.1, 159.2, 149.0, 144.1, 139.9, 139.4, 136.9, 135.7, 130.5, 130.1, 128.3, 127.9, 127.4, 125.6, 121.5, 80.4, 43.3, 36.1, 30.5, 20.5.

IR: V_{max} (neat/cm^{-1})

3067, 3048, 2927, 1660, 1378, 1319, 1129.

Spectroscopic data matched those previously reported[2].

Synthesis of [2-(*o*-tolyl)pyridine]–[chlorpropham] 10e

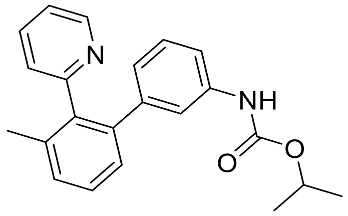

10e

Reaction set up outside of the glovebox using Schlenk technique: The general procedure B was applied using 2-(*o*-tolyl)pyridine **8a** (68 mg, 0.4 mmol, 1.0 equiv.), Chlorpropham **9e** (85 mg, 0.4 mmol, 1.0 equiv.) and K_2CO_3 (111 mg, 0.4 mmol, 2.0 equiv.) allowing the reaction mixture to stir for 24 hours at 40 °C. Column chromatography eluting with 50% Et_2O in hexane afforded the product **10e** as a colourless amorphous solid (118 mg, 85%).

^{1}H NMR: (400 MHz, $CDCl_3$)

8.65 – 8.60 (m, 1H), 7.46 (td, *J* = 7.7, 1.8 Hz, 1H), 7.37 – 7.23 (m, 4H), 7.09 (ddd, *J* = 7.7, 4.9, 1.2 Hz, 1H), 7.06 – 6.99 (m, 2H), 6.91 (d, *J* = 7.8 Hz, 1H), 6.68 (d, *J* = 7.6 Hz, 1H), 6.36 (bs, 1H), 4.98 (hept, *J* = 6.2 Hz, 1H), 2.17 (s, 3H), 1.28 (d, *J* = 6.2 Hz, 6H).

^{13}C NMR: (126 MHz, $CDCl_3$)

159.6, 153.2, 149.0, 142.7, 140.9, 139.4, 137.7, 136.8, 135.9, 129.7, 128.4, 128.2, 127.7, 125.7, 125.0, 121.5, 119.8, 116.6, 68.8, 22.2, 20.6.

IR: V_{max} (neat/cm^{-1})

3192, 3022, 2974, 2937, 1710, 1588, 1558, 1224, 1116.

m.p: 65–68 °C (hexane)

Spectroscopic data matched those previously reported[2].

Synthesis of [2-(*o*-tolyl)pyridine]–[ladasten] 10f

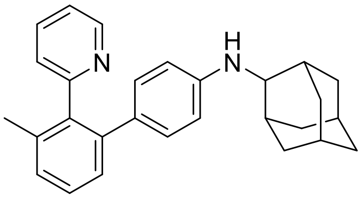

10f

Reaction set up outside of the glovebox using Schlenk technique: The general procedure B was applied using 2-(*o*-tolyl)pyridine **8a** (28 mg, 0.16 mmol, 1.0 equiv.), a grinded pill of Ladasten containing 50 mg of Bromantane **9f** (0.16 mmol, 1.0 equiv.), KOAc (4.8 mg, 0.05 mmol, 30 mol%) and K_2CO_3 (45 mg, 0.33 mmol, 2.0 equiv.). The resulting mixture was stirred at 40 °C for 72 h. Column chromatography eluting with 30% Et_2O in hexane afforded the product **10f** as an off-white amorphous solid (61 mg, 95%). The excipients contained in a pill of Ladasten are potato starch, magnesium stearate and microcrystalline cellulose in unknown amounts.

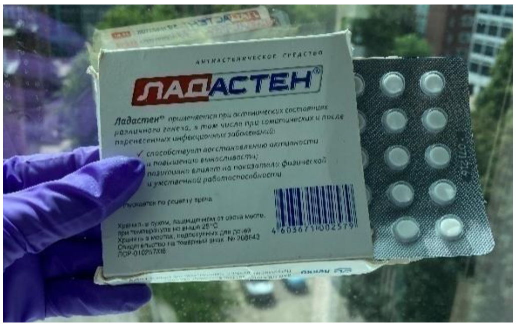

Figure 11 Image of ladasten pill used in reaction

^{1}H NMR: (500 MHz, $CDCl_3$)

8.66 (dd, *J* = 4.9, 2.9 Hz, 1H), 7.48 (td, *J* = 7.7, 1.9 Hz, 1H), 7.31 (t, *J* = 7.6 Hz, 1H), 7.29 – 7.21 (m, 2H), 7.15 – 7.09 (m, 1H), 6.91 (d, *J* = 7.8 Hz, 1H), 6.86 (d, *J* = 8.5 Hz, 2H), 6.38 (d, *J* = 8.5 Hz, 2H), 3.89 (bs, 1H), 3.46 (bs, 1H), 2.16 (s, 3H), 1.96 (s, 2H), 1.99 – 1.85 (m, 5H), 1.84 – 1.70 (m, 5H), 1.62 – 1.54 (m, 2H).

^{13}C NMR: (126 MHz, $CDCl_3$)

160.4, 148.9, 145.9, 141.4, 139.2, 136.7, 135.9, 130.7, 130.1, 128.7, 128.1, 127.7, 125.8, 121.2, 112.4, 56.9, 37.8, 37.5, 31.8, 31.7, 27.5, 27.4, 20.7.

m.p: 220–224 °C (hexane)

Spectroscopic data matched those previously reported[2].

Synthesis of [diazepam]-[5-*m*-xylene] 11g

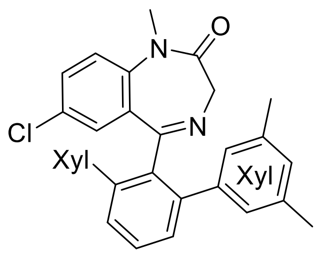

11g

Reaction set up outside of the glovebox using Schlenk technique: The general procedure B was applied using Diazepam **8b** (57 mg, 0.2 mmol, 1.0 equiv.), 5-iodo-*m*-xylene **9g** (58 µL, 0.4 mmol, 2.0 equiv.) and K_2CO_3 (83 mg, 0.6 mmol, 3.0 equiv.). The resulting mixture was stirred at 40 °C for 72 h. Column chromatography eluting with 40% Et_2O in hexane afforded the product **11g** as a colourless amorphous solid (85 mg, 86%).

^{1}H NMR: (400 MHz, $CDCl_3$)

7.55 – 7.40 (m, 2H), 7.23 (dd, *J* = 8.8, 2.6 Hz, 1H), 7.20 – 7.13 (m, 1H), 7.09 – 7.01 (m, 3H), 6.92 (bs, 1H), 6.80 (d, *J* = 8.9 Hz, 1H), 6.75 (bs, 1H), 6.55 (bs, 2H), 4.50 (d, *J* = 11.0 Hz, 1H), 3.31 (d, *J* = 10.9 Hz, 1H), 2.89 (s, 3H), 2.29 (s, 6H), 2.13 (s, 6H).

^{13}C NMR: (101 MHz, $CDCl_3$)

169.2, 168.3, 142.6, 142.3, 141.3, 141.0, 140.7, 137.2, 137.0, 136.7, 131.9, 130.5, 129.6, 129.1, 128.8, 128.6, 128.5, 128.1, 127.5, 127.3, 121.3, 56.0, 34.5, 21.4, 21.2.

IR: V_{max} (neat/cm^{-1})

2980, 2840, 1685, 1611, 1479, 1398, 1335.

m.p: 173–175 °C (hexane)

Spectroscopic data matched those previously reported[2].

Synthesis of [zolimidine]–[5-*m*-xylene] 11h

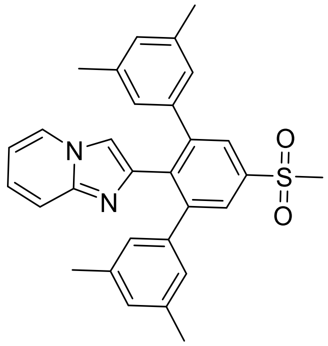

11h

Reaction set up outside of the glovebox using Schlenk technique: The general procedure B was applied using Zolimidine **8c** (109 mg, 0.4 mmol, 1.0 equiv.), 5-Br-*m*-xylene **9a** (109 µL, 0.8 mmol, 2.0 equiv.) and K_2CO_3 (166 mg, 1.2 mmol, 3 equiv.). The resulting mixture was stirred at 40 °C for 72 h. Column chromatography eluting with 80% Et_2O in hexane afforded the product **11h** as a pale-yellow amorphous solid (177 mg, 95%).

^{1}H NMR: (500 MHz, $CDCl_3$)

7.95 (s, 2H), 7.83 (dt, *J* = 6.9, 1.2 Hz, 1H), 7.44 (dd, *J* = 9.2, 1.1 Hz, 1H), 7.07 – 7.02 (m, 1H), 6.98 (s, 1H), 6.88 (s, 4H), 6.79 (s, 2H), 6.67 – 6.62 (m, 1H), 3.12 (s, 3H), 2.14 (s, 12H).

^{13}C NMR: (126 MHz, $CDCl_3$)

145.2, 144.0, 142.7, 140.0, 139.8, 137.5, 137.1, 128.8, 127.3, 127.2, 125.4, 124.0, 117.4, 112.3, 112.0, 44.6, 21.1.

IR: V_{max} (neat/cm^{-1})

3052, 2980, 2928, 1602, 1499, 1369, 1146.

m.p: 228–230 °C (hexane, decomp)

Spectroscopic data matched those previously reported[2].

Synthesis of [zolpidem derivative]-[clozapine] 10i

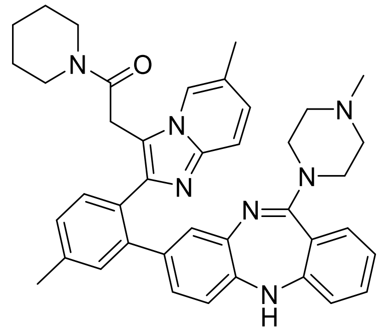

10i

Reaction set up outside of the glovebox using Schlenk technique: The general procedure B was applied using zolpidem derivative **8d** (70 mg, 0.2 mmol, 1.0 equiv.), Clozapine **9h** (65 mg, 0.2 mmol, 1.0 equiv.), **RuAqua** (28.4 mg, 0.04 mmol, 20 mol%), KOAc (5.9 mg, 0.06 mmol, 30 mol%) and K_2CO_3 (55 mg, 0.4 mmol, 2.0 equiv.) in NMP (0.4 mL, 0.5 M with respect to **8d**) at 40 °C for 144 h. Column chromatography eluting with 10–20% MeOH in CH_2Cl_2 afforded the product **10i** as a dark yellow amorphous solid (64 mg, 50%).

^{1}H NMR: (400 MHz, d_4-MeOD)

7.78 (s, 1H), 7.44 – 7.37 (m, 2H), 7.29 – 7.16 (m, 4H), 7.11 (dd, *J* = 9.3, 1.8 Hz, 1H), 7.01 – 6.86 (m, 3H), 6.58 – 6.48 (m, 2H), 3.49 – 3.19 (m, 9H), 3.05 (s, 2H), 2.52 (s, 4H), 2.37 (s, 3H), 2.32 (s, 3H), 2.25 (s, 3H), 1.49 (p, *J* = 5.7 Hz, 2H), 1.40 – 1.10 (m, 4H).

^{13}C NMR: (101 MHz, d_4-MeOD)

168.0, 164.6, 155.6, 145.1, 144.0, 143.6, 142.2, 141.4, 140.0, 137.8, 133.3, 133.0, 131.6, 131.3, 130.4, 129.1, 128.9, 128.5, 126.2, 124.3, 123.8, 123.6, 123.3, 121.2, 120.2, 116.7, 116.3, 55.6, 48.1, 47.9, 46.0, 44.1, 29.6, 27.3, 26.6, 25.2, 21.4, 18.3.

IR: V_{max} (neat/cm^{-1})

3295, 2921, 2848, 2790, 2345, 2189, 1600, 1561, 1452,1365, 1227, 1004 883, 774.

MS: HRMS (ESI$^+$)

calculated for $C_{40}H_{43}N_7O$ $[M+H]^+$: 638.3602, found: 638.3600.

m.p: 154—156 °C (MeOH, decomp.)

Further Examples of RuAqua catalysed C–H Arylation

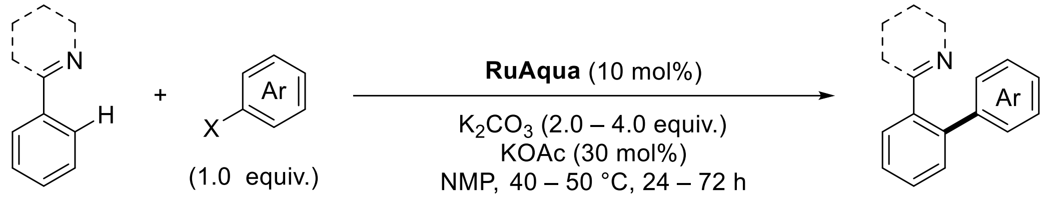

8 **9** **10**

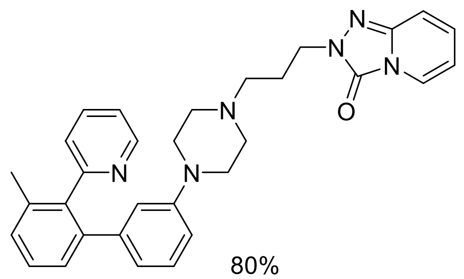

80%
10j
from trazadone

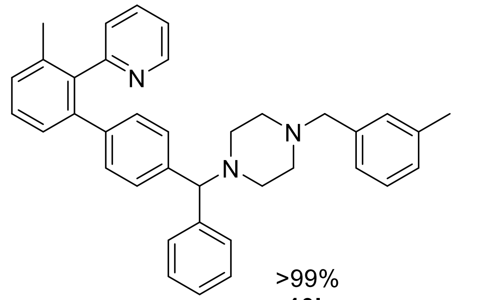

>99%
10k
from meclizine

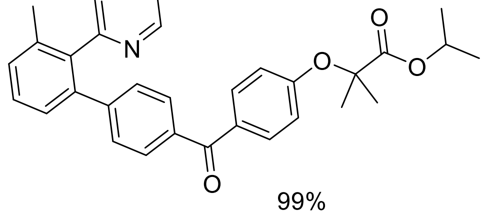

99%
10l
from fenofibrate

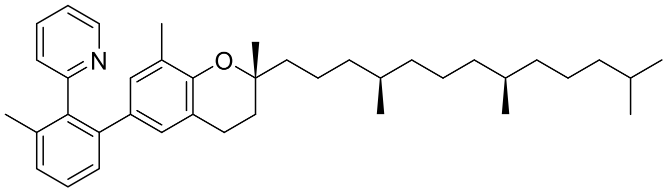

90%
10m
from δ-tocopherol

Synthesis of [2-(*o*-tolyl)pyridine]–[trazodone] 10j

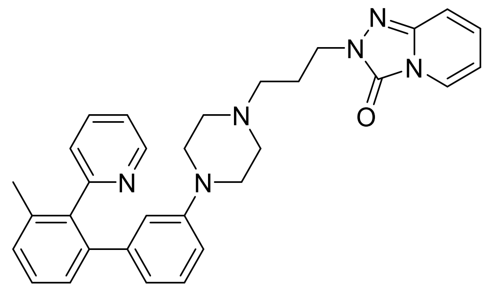

10j

Reaction set up outside of the glovebox using Schlenk technique: The general procedure B was applied using 2-(*o*-tolyl)pyridine **8a** (68 mg, 0.4 mmol, 1.0 equiv.), Trazodone • HCl **9i** (163 mg, 0.4 mmol, 1.0 equiv.) and K_2CO_3 (166 mg, 1.2 mmol, 3.0 equiv.) at 50 °C for 48 h. Column chromatography eluting with 1–5% MeOH in CH_2Cl_2 afforded the product **10j** as an off-white amorphous solid (167 mg, 80%).

^{1}H NMR: (500 MHz, $CDCl_3$)

8.65 – 8.59 (m, 1H), 7.75 (dt, *J* = 7.0, 1.2 Hz, 1H), 7.44 (td, *J* = 7.6, 1.8 Hz, 1H), 7.36 – 7.31 (m, 1H), 7.31 – 7.23 (m, 2H), 7.12 – 7.01 (m, 4H), 6.90 – 6.80 (d, *J* = 8.9 Hz, 1H), 6.65 (m, 2H), 6.57 – 6.51 (m, 1H), 6.50 – 6.43 (m, 1H), 4.07 (t, *J* = 7.0 Hz, 2H), 2.90 – 2.85 (m, 4H), 2.55 – 2.40 (m, 6H), 2.17 (s, 3H), 2.04 (quint, *J* = 7.1 Hz, 2H).

^{13}C NMR: (126 MHz, $CDCl_3$)

160.0, 150.5, 148.8, 148.7, 142.4, 141.8, 141.6, 139.3, 136.8, 136.0, 129.9, 129.4, 128.5, 128.1, 127.6, 125.7, 123.9, 121.4, 121.0, 118.2, 115.5, 114.1, 110.6, 55.7, 53.2, 49.0, 44.6, 26.2, 20.6.

IR: V_{max} (neat/cm^{-1})

2997, 2916, 1631, 1600, 1566, 1498, 1304.

m.p: 60–62 °C (MeOH)

Spectroscopic data matched those previously reported[2].

Synthesis of [2-(*o*-tolyl)pyridine]–[meclizine] 10k

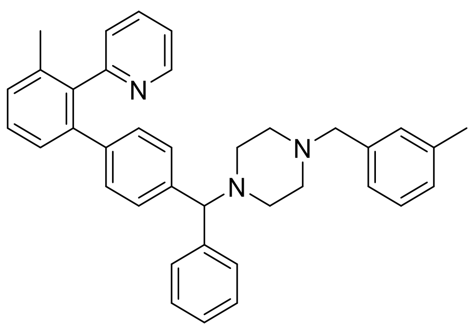

10k

Reaction set up outside of the glovebox using Schlenk technique: The general procedure B was applied using 2-(*o*-tolyl)pyridine **8a** (68 mg, 0.4 mmol, 1.0 equiv.), Meclizine • 2HCl **9j** (186 mg, 0.4 mmol, 1.0 equiv.) and K_2CO_3 (221 mg, 1.6 mmol, 4.0 equiv.) at 50 °C for 24 h. Column chromatography eluting with 50% Et_2O in hexane afforded the product **10k** as a colourless amorphous solid (210 mg, >99%).

^{1}H NMR: (400 MHz, $CDCl_3$)

8.60 – 8.55 (m, 1H), 7.36 – 7.26 (m, 3H), 7.25 – 7.20 (m, 5H), 7.20 – 7.00 (m, 8H), 6.93 (d, *J* = 8.3 Hz, 2H), 6.77 (d, *J* = 7.8 Hz, 1H), 4.15 (s, 1H), 3.47 – 3.40 (m, 2H), 2.55 – 2.22 (m, 11H), 2.16 (s, 3H).

^{13}C NMR: (101 MHz, $CDCl_3$)

159.7, 148.9, 142.7, 141.3, 140.6, 140.4, 139.5, 138.1, 137.9, 136.7, 135.6, 130.1, 129.8, 129.5, 128.4, 128.2 (2 x resonances), 128.1, 127.9, 127.5, 127.4, 126.9, 126.5, 125.9, 121.3, 75.7, 63.2, 53.5, 51.8, 21.5, 20.6.

IR: V_{max} (neat/cm^{-1})

2980, 2960, 2803, 1593, 1451, 1132, 1010.

m.p: 167–169 °C (hexane)

Spectroscopic data matched those previously reported[2].

Synthesis of [2-(*o*-tolyl)pyridine]–[fenofibrate] 10l

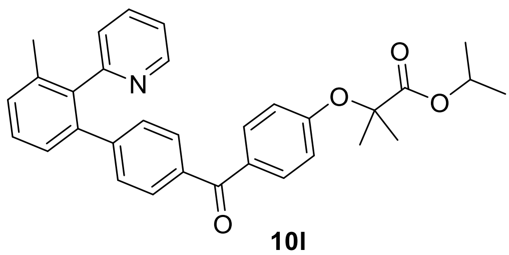

10l

Reaction set up outside of the glovebox using Schlenk technique: The general procedure B was applied using 2-(*o*-tolyl)pyridine **8a** (68 mg, 0.4 mmol, 1.0 equiv.), Fenofibrate **9k** (144 mg, 0.4 mmol, 1.0 equiv.) and K_2CO_3 (111 mg, 0.8 mmol, 2.0 equiv.). The resulting mixture was stirred at 40 °C for 24 h. Column chromatography eluting with 30–60% Et_2O in hexane afforded the product **10l** as an off-white amorphous solid (196 mg, 99%).

^{1}H NMR: (500 MHz, $CDCl_3$)

8.73 – 8.62 (m, 1H), 7.71 – 7.65 (m, 2H), 7.57 – 7.52 (m, 2H), 7.44 (t, *J* = 7.5 Hz, 1H), 7.37 (d, *J* = 7.0 Hz, 1H), 7.32 (d, *J* = 7.9 Hz, 1H), 7.20 (d, *J* = 8.4 Hz, 2H), 7.01 (td, *J* = 7.4, 2.5 Hz, 1H), 6.86 – 6.80 (m, 2H), 5.08 (hept, *J* = 6.3 Hz, 1H), 2.24 (s, 3H), 1.65 (s, 6H), 1.19 (d, *J* = 6.4 Hz, 6H).

^{13}C NMR: (101 MHz, $CDCl_3$)

195.2, 173.1, 159.4, 159.2, 149.0, 145.7, 140.2, 139.3, 136.9, 135.9, 135.8, 131.9, 130.7, 130.0, 129.5, 129.3, 128.2, 127.4, 125.6, 121.6, 117.1, 79.3, 69.3, 25.4, 21.5, 20.4.

IR: V_{max} (neat/cm^{-1})

3064, 2985, 1716, 1641, 1597, 1287, 1248, 1175, 1147.

m.p: 130–134 °C (hexane)

Spectroscopic data matched those previously reported[2].

Synthesis of [2-(*o*-tolyl)pyridine]–[δ-tocopherol] 10m

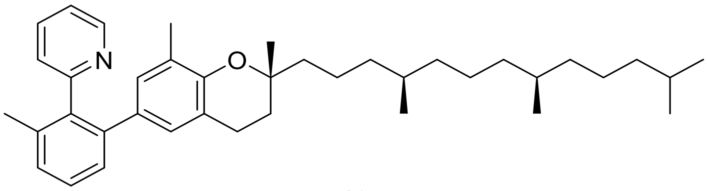

10m

Reaction set up outside of the glovebox using Schlenk technique: The general procedure B was applied using 2-(*o*-tolyl)pyridine **8a** (34 mg, 0.2 mmol, 1.0 equiv.), δ-Tocopherol-OTf **9l** (107 mg, 0.2 mmol, 1.0 equiv.), **RuAqua** (14.2 mg, 0.02 mmol, 10 mol%), KOAc (5.9 mg, 0.06 mmol, 0.3 equiv.) and K_2CO_3 (55 mg, 0.4 mmol, 2.0 equiv.) allowing the reaction mixture to stir in NMP (0.4 mL, 0.5 M with respect to **8a**) at 40 °C for 24 hours. Column chromatography eluting with 10% Et_2O in hexane afforded the product **10m** as a colourless oil (86 mg, 90%).

^{1}H NMR: (400 MHz, $CDCl_3$)

8.64 (d, *J* = 4.6 Hz, 1H), 7.46 (td, *J* = 7.7, 2.0 Hz, 1H), 7.35 – 7.27 (m, 2H), 7.23 (d, *J* = 7.2 Hz, 1H), 7.08 (dd, *J* = 8.0, 4.3 Hz, 1H), 6.89 (d, *J* = 7.7 Hz, 1H), 6.66 (s, 1H), 6.55 (s, 1H), 2.58 – 2.47 (m, 2H), 2.17 (s, 3H), 1.96 (s, 3H), 1.60 – 1.76 (m, 2H), 1.59 – 0.97 (m, 24H), 0.80 – 0.91 (m, 12H).

^{13}C NMR: (101 MHz, $CDCl_3$)

160.5, 151.0, 148.9, 141.6, 139.6, 136.8, 135.9, 132.4, 130.1, 129.0, 128.6, 128.2, 127.8, 126.0, 125.5, 121.3, 119.9, 76.3, 40.4, 39.7, 37.8, 37.6, 33.1, 33.0, 31.6, 28.3, 25.1, 24.8, 24.4, 23.1, 23.0, 22.5, 21.3, 20.9, 20.1, 20.0, 16.2.

IR: V_{max} (neat/cm^{-1})

2924, 2866, 1585, 1486, 1459, 1377, 1225, 1201, 1149, 1111.

Spectroscopic data matched those previously reported[2].

The Need for Inert Conditions During C–H Functionalisation Reactions

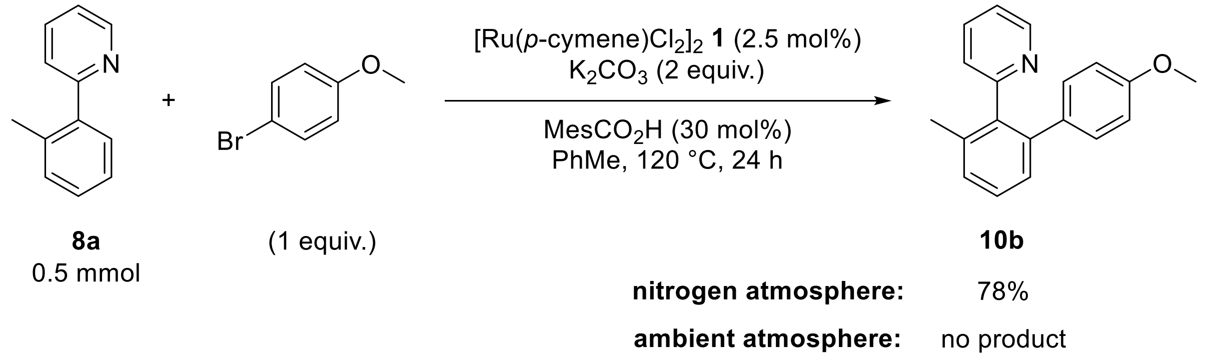

Despite the air-and moisture stability of **RuAqua**, the C–H arylation does not proceed without an inert atmosphere. This is due to the formation of air- and moisture sensitive intermediates such as structure **7**. The formation of this intermediate means that the reaction requires inert conditions regardless of the pre-catalyst used to access this reaction pathway. To demonstrate this, the reaction was performed with [Ru(*p*-cymene)Cl_2]$_2$ **1** using previously optimised conditions[6] both under air and under nitrogen. The reaction ran under ambient conditions gave no conversion to the desired product while the reaction ran under nitrogen gave proceeded with 78% conversion.

Experimental Procedure:

Reactions were set up outside of the glovebox.

Nitrogen atmosphere: [Ru(*p*-cymene)Cl_2]$_2$ **1** (7.7 mg, 12 μmol, 5 mol% with respect to Ru), $MesCO_2H$ (25 mg, 0.15 mmol, 30 mol%) and K_2CO_3 (138 mg, 1.00 mmol, 2 equiv.) were weighed into a microwave vial loaded with a stirrer bar. The microwave vial was capped before performing 3 x 5 min evac-refill cycles with N_2. Degassed 2-(*o*-tolyl)pyridine **8a** (84 mg, 0.50 mmol, 1 equiv.), 4-bromo-anisole (140 mg, 0.75 mmol, 1.5 equiv.) and toluene (2 mL) were added to the vessel which was then wrapped in parafilm before allowing the reaction to stir for 24 h at 120 °C. After this time, quantitative ^{1}H NMR using 1,3,5-trimethoxybenzene as an internal standard indicated 78% conversion to the desired product.

Ambient atmosphere: [Ru(*p*-cymene)Cl_2]$_2$ **1** (7.7 mg, 12 μmol, 5 mol% with respect to Ru), $MesCO_2H$ (25 mg, 0.15 mmol, 30 mol%) and K_2CO_3 (138 mg, 1.00 mmol, 2 equiv.) were weighed into a microwave vial loaded with a stirrer bar. 2-(*o*-tolyl)pyridine **8a** (84 mg, 0.50 mmol, 1 equiv.), 4-bromo-anisole (140 mg, 0.75 mmol, 1.5 equiv.) and toluene (2 mL) were added to the vessel which was then capped before allowing the reaction to stir for 24 h at 120 °C. After this time, ^{1}H NMR indicated no formation of the desired product.

The Potential for Reduced Catalyst Loadings

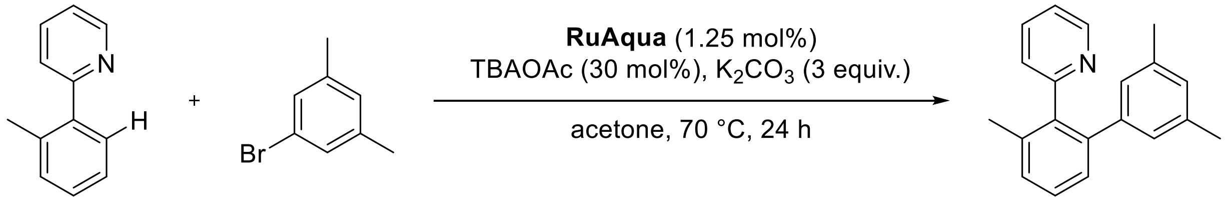

8a 1 mmol | **9a** 1 equiv. | **10a** 73%

To allow direct comparison to the literature, conditions for used with **RuAqua** were not reoptimized. As a result of this, there is the potential to further improve the conditions for each reaction class. For example, the C–H Arylation of 2-(*o*-tolyl)pyridine **8a** can be performed with reduced catalyst loadings and in acetone as a solvent to avoid the use of NMP when elevated temperatures are used.

Experimental Procedure:

Reaction was setup outside of the glovebox. To a microwave vial equipped with a magnetic stirrer bar was added 2-(*o*-tolyl)pyridine **8a** (169 mg, 1 mmol, 1 equiv.), 5-bromo-*m*-xylene **9a** (185 mg, 1 mmol, 1 equiv.), K_2CO_3 (416 mg, 3 mmol, 3 equiv.) and tetrabutylammonium acetate (90 mg, 0.3 mmol, 30 mol%). The vial was capped before performing 3 x 5 min evac-refill cycles using nitrogen. A stock solution of **RuAqua** (8.9 mg in 1 mL of acetone which was previously degassed via freeze-pump-thaw) was then added and the reaction was stirred at 70 °C for 24 hours. After this time, quantitative ^{1}H NMR using 1,3,5-trimethoxybenzene as an internal standard indicated 73% conversion to the desired product **10a**.

Effect of Pivalic Acid on *ortho*-C–H Arylations

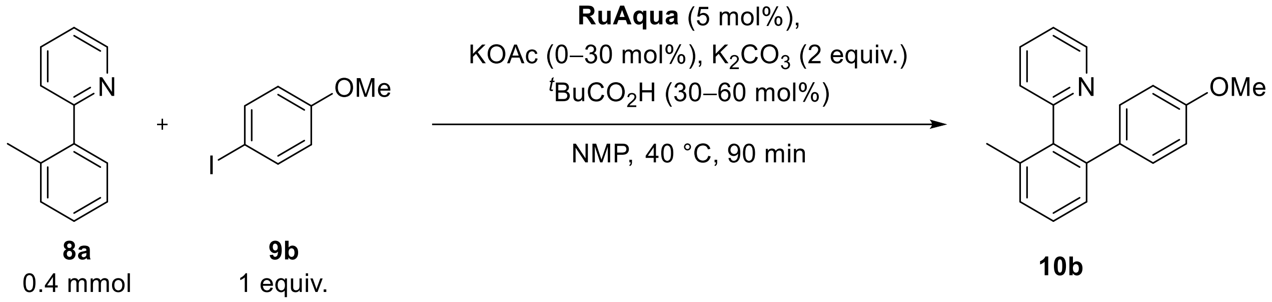

The effect of adding small amounts of pivalic acid to the reaction was explored to assess whether or not this would enhance reactivity. Unfortunately, addition of pivalic acid to *ortho*-arylation reactions results in reaction inhibition. Specifically, when pivalic acid is used (with and without KOAc) the rate of reaction is significantly reduced.

Experimental procedure:

Reaction set up outside of the glovebox using Schlenk Technique: The general procedure B was applied using 2-(*o*-tolyl)pyridine **8a** (68 mg, 0.4 mmol, 1.0 equiv.), 4-iodo-anisole **9b** (94 mg, 0.4 mmol, 1.0 equiv.) and K_2CO_3 (111 mg, 0.8 mmol, 2.0 equiv.) with varying amounts of KOAc and $^tBuCO_2H$ added to the reaction which was stirred at 40 °C for 90 mins. 1,3,5-trimethoxybenzene was added as an internal standard and the reaction mixtures analysed by quantitative ^1H NMR.

Table 4: Effect of Pivalic Acid on Ortho-C–H Arylation

KOAc (mol%)	Pivalic Acid (mol%)	Yield 10b (%)
30	0	60
30	30	6
30	60	16
0	30	23

RuAqua Catalysed C–H Alkylation of Arenes with Primary Alkyl Bromides

Attainment of Starting Materials

Directing Group Substrates:

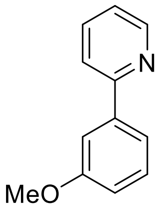

8e

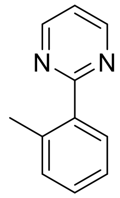

8f

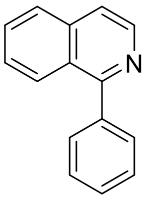

8g

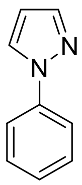

8h

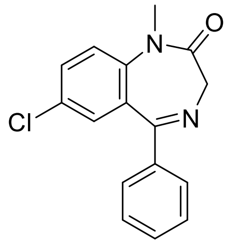

8b

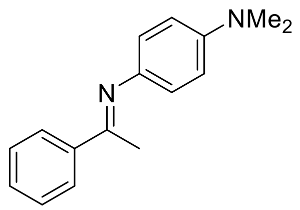

8i

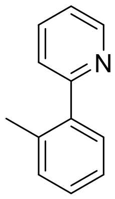

8a

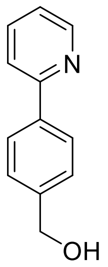

8q

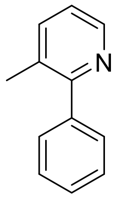

8r

8g, **8h**, **8b**, an **8r** were commercially available. **8r** was purified via column chromatography eluting with 0–10% EtOAc in hexane prior to use.

8a[3], **8e**[7], **8f**[8], **8i**[9] and **8q**[10] were prepared according to previously published protocols. The synthesis of **8a** has been discussed in a previous section.

Preparation of Starting Material 8e

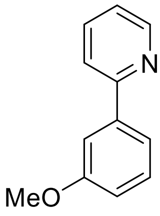

8e

Compound **8e** was prepared according to a previously reported procedure with data in accordance.[7]

^{1}H NMR: (400 MHz, $CDCl_3$)

8.70 (d, *J* = 4.9 Hz, 1H), 7.81 (d, *J* = 6.8 Hz, 1H), 7.78 (dd, *J* = 7.6, 1.9 Hz, 1H), 7.68 (td, *J* = 7.7, 2.0 Hz, 1H), 7.43 – 7.32 (m, 1H), 7.24 – 7.15 (m, 1H), 7.08 (t, *J* = 7.5 Hz, 1H), 7.00 (d, *J* = 8.3 Hz, 1H), 3.84 (s, 3H).

^{13}C NMR: (126 MHz, $CDCl_3$)

157.0, 156.2, 149.4, 135.7, 131.2, 130.0, 129.2, 125.2, 121.7, 121.1, 111.4, 55.6.

Preparation of Starting Material 8f

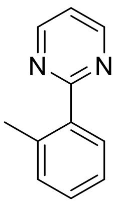

8f

Compound **8f** was prepared according to a previously reported procedure with data in accordance.[8]

^{1}H NMR: (500 MHz, $CDCl_3$)

8.83 (d, *J* = 4.9 Hz, 2H), 7.83 – 7.78 (m, 1H), 7.37 – 7.27 (m, 3H), 7.19 (t, *J* = 4.9 Hz, 1H), 2.55 (s, 3H).

^{13}C NMR: (126 MHz, $CDCl_3$)

167.8, 156.9, 138.2, 137.3, 131.4, 130.5, 129.5, 126.0, 118.6, 21.1.

Preparation of Starting Material 8i

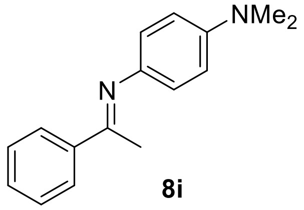

8i

Compound **8i** was prepared according to a previously reported procedure with data in accordance.[9]

^{1}H NMR: (400 MHz, $CDCl_3$)

8.02 – 7.95 (m, 2H), 7.49 – 7.43 (m, 3H), 6.80 (d, J = 1.2 Hz, 4H), 2.97 (s, 6H), 2.31 (s, 3H).

^{13}C NMR: (126 MHz, $CDCl_3$)

165.0, 147.5, 141.7, 140.2, 130.2, 128.4, 127.2, 121.2, 113.5, 41.3, 17.4.

Preparation of Starting Material 8q

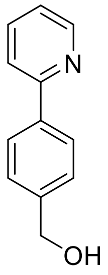

8q

Compound **8q** was prepared according to a previously reported procedure with data in accordance.[10]

^{1}H NMR: (400 MHz, $CDCl_3$)

8.64 – 8.51 (m, 1H), 7.83 (d, *J* = 8.3 Hz, 2H), 7.72 – 7.53 (m, 2H), 7.33 (d, *J* = 8.6 Hz, 2H), 7.19 – 7.07 (m, 1H), 4.62 (s, 2H), 2.73 (s, 1H).

^{13}C NMR: (126 MHz, $CDCl_3$)

157.4, 149.6, 142.2, 138.4, 137.0, 127.3, 127.2, 122.2, 120.8, 64.8.

Primary Alkyl Bromides:

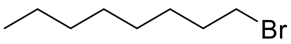

12a

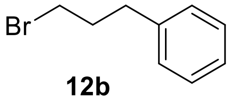

12b

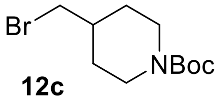

12c

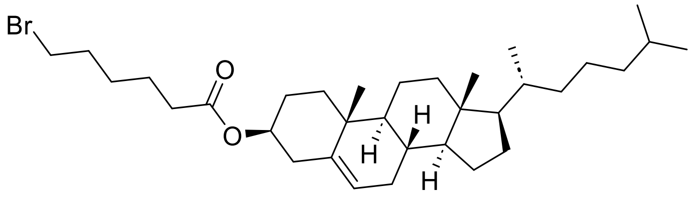

12d

Compounds **12a**, **12b** and **12c** were commercially available.

Substrate **12d** was synthesized according to described procedures[11].

Preparation of Starting Material 12d

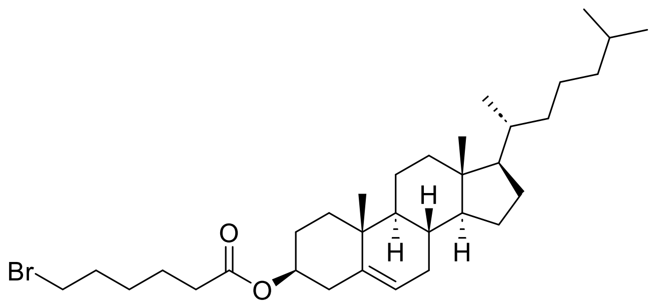

12d

Compound **12d** was prepared according to a previously reported procedure with data in accordance.[11]

^{1}H NMR: (500 MHz, $CDCl_3$)

5.30 (d, *J* = 4.9 Hz, 1H), 4.60 – 4.47 (m, 1H), 3.34 (t, *J* = 6.8 Hz, 2H), 2.33 – 2.09 (m, 4H), 2.08 – 1.86 (m, 2H), 1.87 – 1.66 (m, 5H), 1.64 – 1.54 (m, 2H), 1.53 – 0.87 (m, 26H), 0.85 (d, *J* = 6.5 Hz, 3H), 0.80 (dd, *J* = 6.6, 2.3 Hz, 6H), 0.61 (s, 3H).

^{13}C NMR: (126 MHz, $CDCl_3$)

173.0, 139.8, 122.8, 74.0, 56.8, 56.3, 50.2, 42.5, 39.9, 39.7, 38.3, 37.1, 36.7, 36.3, 35.9, 34.6, 33.7, 32.6, 32.1, 32.0, 28.4, 28.2, 28.0, 27.8, 24.4, 24.3, 24.0, 23.0, 22.7, 21.2, 19.5, 18.9, 12.0.

General Procedure C: RuAqua Catalysed Primary Alkylation of DG-Containing Arenes with Primary Alkyl Bromides

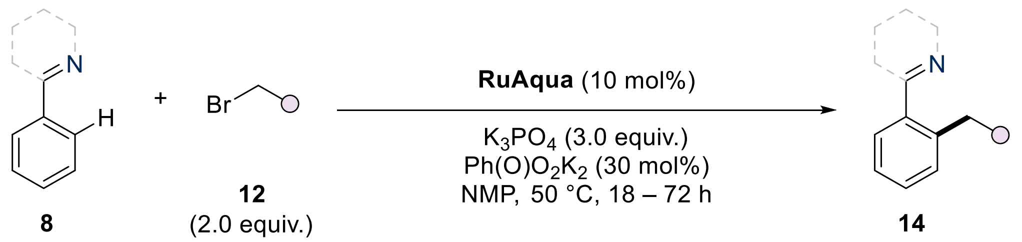

Reaction set up outside of the glovebox using Schlenk technique: Reaction conditions adapted from previously reported procedure[12]. A 10 mL Schlenk tube was charged with **RuAqua** (28.4 mg, 0.04 mmol, 10 mol%), K_3PO_4 (255 mg, 1.20 mmol, 3 equiv.), and potassium phenylphosphonate (29 mg, 0.12 mmol, 30 mol%). The appropriate DG-containing arene **8** (0.4 mmol, 1.0 equiv.) and primary alkyl bromide **12** (0.8 mmol, 2.0 equiv.) were then added, followed by NMP (2 mL, 0.2 M with respect to **8**) first adding all solids to the reaction vessel before performing 3 x 5 min evac-refill cycles with subsequent addition of degassed oils/solvents. The reaction mixture was then stirred at 50 °C for 18 h. Upon completion, the reaction mixture was diluted with water (10 mL) and washed with Et_2O (3 x 10 mL), before being dried over $MgSO_4$, filtered, and concentrated under reduced pressure. The crude mixture was then loaded onto a silica gel column and purified by flash chromatography using the indicated conditions.

$PhP(O)O_2K_2$ was prepared following according to previously reported procedure[12].

Synthesis of 2-(5-methoxy-2-octylphenyl)pyridine 14a

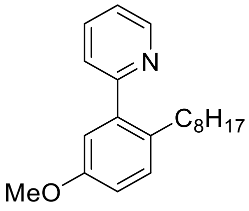

14a

Reaction set up outside of the glovebox using Schlenk technique: The reaction was carried out following general procedure C using 2-(3-methoxyphenyl)pyridine **8e** (72 mg, 0.4 mmol, 1.0 equiv.) and 1-bromooctane **12a** (139 µL, 0.8 mmol, 2 equiv.). The crude reaction mixture was purified by column chromatography eluting with a 1:1 mixture of EtOAc and CH_2Cl_2 (0–10%) in hexane to give the compound **14a** (116 mg, 97%) as a colourless oil.

^{1}H NMR: (500 MHz, $CDCl_3$)

8.68 (d, *J* = 4.9 Hz, 1H), 7.70 (td, *J* = 7.6, 2.3 Hz, 1H), 7.30 – 7.18 (m, 3H), 6.89 (d, *J* = 7.8 Hz, 1H), 6.79 (d, *J* = 8.2 Hz, 1H), 3.67 (s, 3H), 2.38 – 2.30 (m, 2H), 1.42 – 1.32 (p, *J* = 7.4 Hz, 2H), 1.28 – 1.04 (m, 10H), 0.83 (t, *J* = 7.2 Hz, 3H).

^{13}C NMR: (126 MHz, $CDCl_3$)

157.1, 157.0, 149.3, 142.8, 135.7, 129.6, 128.9, 125.8, 121.7, 121.6, 108.3, 55.7, 33.0, 31.9, 31.0, 29.5, 29.2, 29.1, 22.7, 14.2.

IR: V_{max} (neat/cm^{-1})

3060, 2954, 2923, 2853, 1586, 1466, 1254, 742.

Spectroscopic data matched those previously reported[12].

Synthesis of 2-(2-methyl-6-octylphenyl)pyrimidine 14b

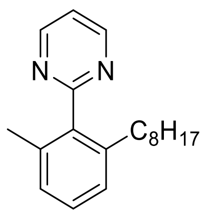

14b

Reaction set up outside of the glovebox using Schlenk technique: The reaction was carried out following general procedure C using 2-phenylpyrimidine **8f** (68 mg, 0.4 mmol, 1.0 equiv.) and 1-bromooctane **12a** (139 µL, 0.8 mmol, 2.0 equiv.). The crude reaction mixture was purified by column chromatography eluting with a 1:1 mixture of EtOAc (0–20%) and CH_2Cl_2 in hexane to give 2-(2-methyl-6-octylphenyl)pyrimidine **14b** (81 mg, 72%) as a colourless oil.

^{1}H NMR: (500 MHz, $CDCl_3$)

8.85 (d, *J* = 4.9 Hz, 2H), 7.27 – 7.18 (m, 2H), 7.08 (t, *J* = 8.5 Hz, 2H), 2.35 – 2.29 (m, 2H), 2.03 (s, 3H), 1.36 (p, *J* = 7.5 Hz, 2H), 1.27 – 1.03 (m, 10H), 0.82 (t, *J* = 7.2 Hz, 3H).

^{13}C NMR: (126 MHz, $CDCl_3$)

168.6, 157.0, 140.3, 138.8, 135.4, 128.4, 127.6, 126.8, 118.8, 33.4, 31.9, 30.9, 29.5, 29.3, 29.1, 22.7, 19.9, 14.1.

IR: V_{max} (neat/cm^{-1})

3056, 2923, 2854, 1597, 1583, 1444, 1408, 778.

Spectroscopic data matched those previously reported[12].

Synthesis of 1-(2-octylphenyl)isoquinoline 14c

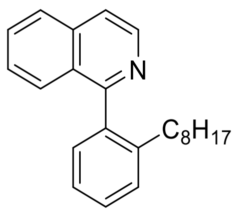

14c

a) Reaction set up outside of the glovebox using Schlenk technique: The reaction was carried out following general procedure C using 1-phenylisoquinoline **8g** (82 mg, 0.4 mmol, 1.0 equiv.) and 1-bromooctane **12a** (139 µL, 0.8 mmol, 2.0 equiv.) allowing the reaction to stir for 24 hours. The crude reaction mixture was purified by column chromatography eluting with a 1:1 mixture of EtOAc and CH_2Cl_2 (0–10%) in hexane to give 1-(2-octylphenyl)isoquinoline **14c** (112 mg, 88%) as a colourless oil.

b) Reaction was set up in a microwave vial in an argon filled glovebox: The general procedure C was applied using $RuCl_3$ (8.1 mg, 0.04 mmol, 10 mol%) in place of **RuAqua**, 1-phenylisoquinoline **8g** (82 mg, 0.4 mmol, 1.0 equiv.) and 1-bromooctane **12a** (139 µL, 0.8 mmol, 2.0 equiv.) allowing the reaction to stir for 24 hours. The crude reaction mixture was analysed by quantitative ^{1}H NMR using nitromethane as an internal standard with no product **14c** observed.

c) Reaction was set up in a microwave vial in an argon filled glovebox: The general procedure C was applied using $[Ru(p\text{-cymene})Cl_2]_2$ (12.4 mg, 0.02 mmol, 10 mol% with respect to Ru) in place of **RuAqua**, 1-phenylisoquinoline **8g** (82 mg, 0.4 mmol, 1.0 equiv.) and 1-bromooctane **12a** (139 µL, 0.8 mmol, 2.0 equiv.) allowing the reaction to stir for 24 hours. The crude reaction mixture was analysed by quantitative ^{1}H NMR using nitromethane as an internal standard with no product observed.

^{1}H NMR: (400 MHz, $CDCl_3$)

8.53 (d, *J* = 5.7 Hz, 1H), 7.78 (d, *J* = 8.1 Hz, 1H), 7.62 – 7.52 (m, 3H), 7.41 – 7.27 (m, 3H), 7.22 (d, *J* = 3.3 Hz, 2H), 2.46 – 2.33 (m, 1H), 2.30 – 2.18 (m, 1H), 1.39 – 1.16 (m, 2H), 1.16 – 0.82 (m, 10H), 0.74 (t, *J* = 7.2 Hz, 3H).

^{13}C NMR: (101 MHz, $CDCl_3$)

161.5, 142.1, 141.5, 138.7, 136.4, 130.1, 129.8, 129.4, 128.4, 127.8, 127.6, 127.1, 126.8, 125.5, 119.8, 33.2, 31.8, 30.8, 29.2, 29.1, 29.0, 22.6, 14.1.

IR: V_{max} (neat/cm^{-1})

3051, 2954, 2922, 2853, 1457, 1357, 976, 747.

Spectroscopic data matched those previously reported[12].

Synthesis of 1-(2-octylphenyl)-1H-pyrazole 14d

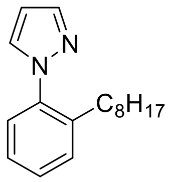

14d

Reaction set up outside of the glovebox using Schlenk technique: The reaction was carried out following general procedure C using 1-phenyl-1H-pyrazole **8h** (58 mg, 0.4 mmol, 1.0 equiv.) and 1-bromooctane **12a** (139 µL, 0.8 mmol, 2.0 equiv.) allowing the reaction mixture to stir for 18 h. The crude reaction mixture was purified by column chromatography eluting with a 1:1 mixture of EtOAc and CH_2Cl_2 (0–10%) in hexane to give 1-(2-octylphenyl)-1H-pyrazole **14d** (82 mg, 80%) as a yellow oil.

^{1}H NMR: (400 MHz, $CDCl_3$)

7.75 – 7.70 (s, 1H), 7.57 (d, *J* = 2.6 Hz, 1H), 7.38 – 7.31 (m, 2H), 7.30 – 7.24 (m, 2H), 6.49 – 6.41 (m, 1H), 2.59 – 2.46 (m, 2H), 1.44 – 1.36 (m, 2H), 1.30 – 1.18 (m, 10H), 0.87 (t, *J* = 7.0 Hz, 3H).

^{13}C NMR: (101 MHz, $CDCl_3$)

140.3, 139.8, 139.2, 130.8, 130.4, 128.7, 126.8, 126.6, 106.2, 32.0, 31.4, 30.7, 29.6, 29.4, 29.3, 22.8, 14.2.

IR: V_{max} (neat/cm^{-1})

2954, 2923, 2853, 1583, 1459, 1379, 938, 747.

Spectroscopic data matched those previously reported[12].

Synthesis of [diazepam]-[*n*-octyl] 14e

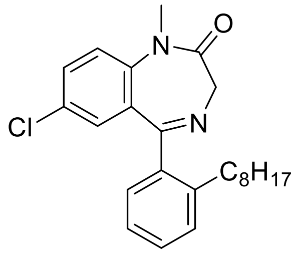

14e

Reaction set up outside of the glovebox using Schlenk technique: The reaction was carried out following general procedure C using diazepam **8b** (57 mg, 0.2 mmol, 1.0 equiv.), K_3PO_4 (127 mg, 0.4 mmol, 2.0 equiv.), $PhP(O)O_2K_2$ (14 mg, 0.06 mmol, 30 mol%), **RuAqua** (28.4 mg, 0.04 mmol, 20 mol%) and 1-bromooctane **12a** (35 µL, 0.4 mmol). The resulting reaction mixture was allowed to stir for 24 hours. The crude reaction mixture was purified by column chromatography eluting with 0–30% EtOAc in hexane to give the product **14e** (37 mg, 47%) as a colourless oil.

^{1}H NMR: (500 MHz, $CDCl_3$)

7.47 – 7.42 (m, 1H), 7.37 – 7.30 (m, 2H), 7.25 (d, *J* = 8.7 Hz, 2H), 7.18 (d, *J* = 7.8 Hz, 1H), 7.03 (d, *J* = 2.7 Hz, 1H), 4.83 (d, *J* = 10.8 Hz, 1H), 3.78 (d, *J* = 10.8 Hz, 1H), 3.41 (s, 3H), 2.37 – 2.26 (m, 1H), 2.20 – 2.06 (m, 1H), 1.38 – 0.92 (m, 12H), 0.83 (t, *J* = 7.1 Hz, 3H).

^{13}C NMR: (126 MHz, $CDCl_3$)

171.1, 169.8, 141.6, 141.3, 138.5, 132.0, 131.5, 130.1, 130.0, 129.9, 129.6, 129.3, 126.1, 122.5, 56.9, 35.0, 33.6, 31.9, 31.1, 29.9, 29.5, 29.3, 22.8, 14.2

Spectroscopic data matched those previously reported[12].

Synthesis of 1-(2-octylphenyl)ethan-1-one 14f

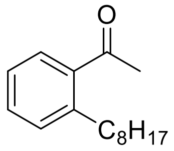

14f

Reaction set up outside of the glovebox using Schlenk technique: The reaction was carried out following general procedure C using (*E*)-*N*,*N*-dimethyl-4-((1- phenylethylidene)amino)aniline **8i** (95 mg, 0.4 mmol) and 1-bromooctane **12a** (138 µL, 0.8 mmol). K_2CO_3 (111 mg, 0.8 mmol, 2.0 equiv.) was used as the base in place of K_3PO_4 and KOAc (12 mg, 0.12 mmol, 30 mol%) as the additive in place of $PhP(O)O_2K_2$. After 72 hours, HCl (2 M, 2 mL) was added to the reaction mixture, and stirred for a further 1 hour, before being washed with ether. The crude reaction mixture was purified by column chromatography eluting with 0–5% EtOAc in hexane to give the product **14f** as a colourless oil (84 mg, 90%).

^{1}H NMR: (500 MHz, $CDCl_3$)

7.61 (dd, *J* = 8.3, 1.3 Hz, 1H), 7.38 (td, *J* = 7.2, 1.4 Hz, 1H), 7.28 – 7.21 (m, 2H), 2.87 – 2.79 (m, 2H), 2.57 (s, 3H), 1.61 – 1.50 (m, 2H), 1.40 – 1.21 (m, 10H), 0.94 – 0.80 (m, 3H).

^{13}C NMR: (101 MHz, $CDCl_3$)

202.3, 142.9, 138.1, 131.2, 131.1, 128.9, 125.6, 34.0, 31.9, 30.0, 29.8, 29.5, 29.3, 27.2, 22.7, 14.1.

IR: V_{max} (neat/cm^{-1})

2955, 2923, 2853, 1685, 1353, 1247, 756.

Spectroscopic data matched those previously reported[12].

Further examples of RuAqua Catalysed C–H Primary Alkylation

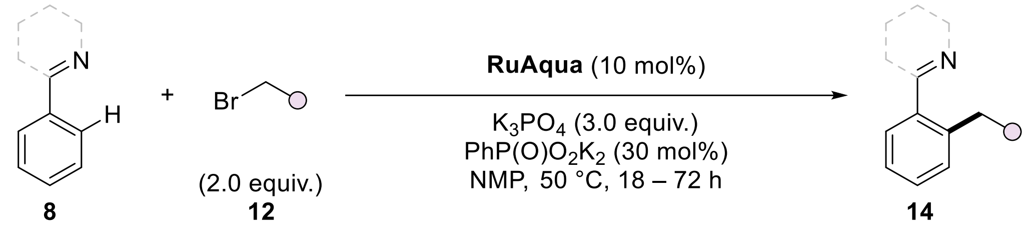

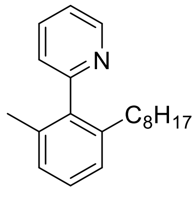

89%
14g

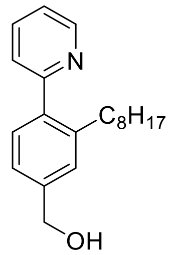

51%
14h

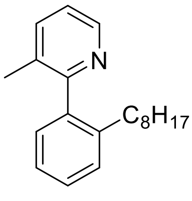

75%
14i

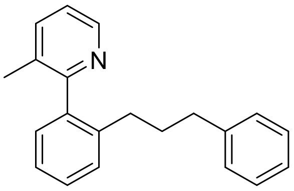

92%
14j

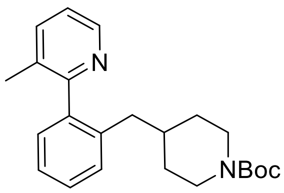

74%
14k

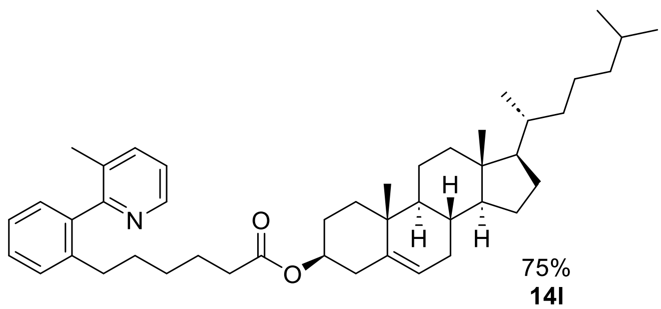

75%
14l

Synthesis of 2-(2-methyl-6-octylphenyl)pyridine 14g

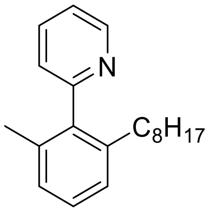

14g

Reaction set up outside of the glovebox using Schlenk technique: The reaction was carried out following general procedure C with 2-(*o*-tolyl)pyridine **8a** (68 mg, 0.4 mmol, 1 equiv.) and 1-bromooctane **12a** (139 µL, 0.8 mmol, 2 equiv.) allowing the reaction mixture to stir for 72 hours. The crude reaction mixture was purified via column chromatography eluting with a 1:1 mixture of EtOAc and CH_2Cl_2 (0–10%) in hexane to give the product **14g** (100 mg, 89%) as a light brown oil.

^{1}H NMR: (400 MHz, $CDCl_3$)

8.72 (d, *J* = 4.9 Hz, 1H), 7.75 (td, *J* = 7.7, 2.1 Hz, 1H), 7.29 – 7.19 (m, 3H), 7.15 – 7.05 (m, 2H), 2.32 (t, *J* = 7.9 Hz, 2H), 2.03 (s, 3H), 1.47 – 1.32 (m, 2H), 1.30 – 1.05 (m, 10H), 0.86 (t, *J* = 7.1 Hz, 3H).

^{13}C NMR: (101 MHz, $CDCl_3$)

159.9, 149.5, 140.7, 140.2, 136.0, 135.8, 127.9, 127.5, 126.7, 124.7, 121.6, 33.5, 31.9, 31.1, 29.5, 29.2, 29.1, 22.7, 20.3, 14.1.

Spectroscopic data matched those previously reported[12].

Synthesis of (3-octyl-4-(pyridin-2-yl)phenyl)methanol 14h

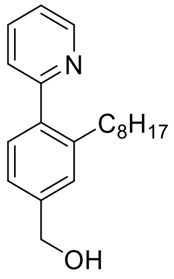

14h

Reaction set up outside of the glovebox using Schlenk technique: The reaction was carried out following general procedure C using methyl 3-(pyridin-2-yl)benzoate **8q** (85 mg, 0.4 mmol, 1.0 equiv.) and 1-bromooctane **12a** (139 µL, 0.8 mmol, 2.0 equiv.). The crude reaction mixture was purified by column chromatography eluting with a 1:1 mixture of EtOAc and CH_2Cl_2 (0–20%) in hexane to give methyl 4-octyl-3-(pyridin-2-yl)benzoate **14h** (67 mg, 51%) as a colourless oil.

^{1}H NMR: (500 MHz, $CDCl_3$)

8.68 (d, *J* = 4.9 Hz, 1H), 7.70 (td, *J* = 7.6, 2.3 Hz, 1H), 7.30 – 7.18 (m, 3H), 6.89 (d, *J* = 7.8 Hz, 1H), 6.79 (d, *J* = 8.2 Hz, 1H), 3.67 (s, 3H), 2.40 – 2.29 (m, 2H), 1.37 (p, *J* = 7.4 Hz, 2H), 1.29 – 1.03 (m, 10H), 0.83 (t, *J* = 7.2 Hz, 3H).

^{13}C NMR: (126 MHz, $CDCl_3$)

160.2, 149.2, 141.3, 141.1, 139.5, 136.3, 130.0, 128.1, 124.3, 124.2, 121.8, 65.0, 33.1, 32.0, 31.4, 29.6, 29.4, 29.3, 22.8, 14.3.

IR: V_{max} (neat/cm^{-1})

3277 (OH), 2953, 2923, 2853, 1587, 1466, 1428, 1025, 789, 735.

Spectroscopic data matched those previously reported[12].

Synthesis of 3-methyl-2-(2-octylphenyl)pyridine 14i

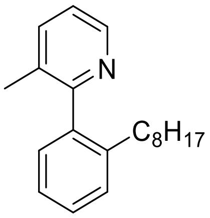

14i

Reaction set up outside of the glovebox using Schlenk technique: The reaction was carried out following general procedure C using 3-methyl-2-phenylpyridine **8r** (68 mg, 0.4 mmol, 1.0 equiv.) and 1-bromooctane **12a** (139 µL, 0.8 mmol, 2.0 equiv.). The crude reaction mixture was purified by column eluting with a 1:1 mixture of EtOAc and CH_2Cl_2 (0–10%) in hexane to give 3-methyl-2-(2-octylphenyl)pyridine **14i** (85 mg, 75%) as a colourless oil.

^{1}H NMR: (500 MHz, $CDCl_3$)

8.53 – 8.46 (m, 1H), 7.59 – 7.55 (m, 1H), 7.27 (d, *J* = 7.9 Hz, 2H), 7.20 (td, *J* = 6.9, 2.3 Hz, 1H), 7.15 (dd, *J* = 7.7, 4.8 Hz, 1H), 7.10 (d, *J* = 7.9 Hz, 1H), 2.32 – 2.77 (m, 2H), 2.07 (s, 3H), 1.35 (s, 2H), 1.25 – 1.01 (m, 10H), 0.81 (t, *J* = 7.2 Hz, 3H).

^{13}C NMR: (101 MHz, $CDCl_3$)

159.8, 146.6, 140.6, 140.0, 137.7, 131.6, 129.4, 128.8, 128.0, 125.7, 122.2, 33.0, 31.9, 30.7, 29.5, 29.3, 29.2, 22.8, 19.4, 14.2.

IR: V_{max} (neat/cm^{-1})

3055, 2953, 2923, 1567, 1423, 1380, 1116, 1023, 752.

Spectroscopic data matched those previously reported[12].

Synthesis of 3-methyl-2-(2-(3-phenylpropyl)phenyl)pyridine 14j

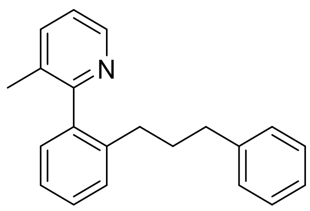

14j

Reaction set up outside of the glovebox using Schlenk technique: The reaction was carried out following general procedure C using 3-methyl-2-phenylpyridine **8r** (68 mg, 0.4 mmol, 1.0 equiv.) and (3-bromopropyl)benzene **12b** (159 mg, 0.4 mmol, 1.0 equiv.). The crude reaction mixture was purified by column chromatography eluting with a 1:1 mixture of EtOAc and CH_2Cl_2 (0–10%) in hexane to give the product **14j** (106 mg, 92%) as a colourless oil.

^{1}H NMR: (500 MHz, $CDCl_3$)

8.43 (d, *J* = 4.7 Hz, 1H), 7.47 (d, *J* = 7.8 Hz, 1H), 7.29 – 7.22 (m, 2H), 7.19 (td, *J* = 6.9, 2.3 Hz, 1H), 7.17 – 7.04 (m, 5H), 6.96 (d, *J* = 7.0 Hz, 2H), 2.41 (t, *J* = 7.6 Hz, 4H), 2.02 (s, 3H), 1.77 – 1.61 (m, 2H).

^{13}C NMR: (126 MHz, $CDCl_3$)

159.5, 146.6, 142.2, 140.0, 137.7, 131.5, 129.4, 128.9, 128.3, 128.2, 128.1, 125.9, 125.6, 122.2, 35.6, 32.6, 32.2, 19.3.

IR: V_{max} (neat/cm^{-1})

3059, 3025, 2931, 2819, 1583, 1569, 1494, 1422, 1024, 745, 698.

Spectroscopic data matched those previously reported[12].

Synthesis of *tert*-butyl 4-(2-(3-methylpyridin-2-yl)benzyl)piperidine-1-carboxylate 14k

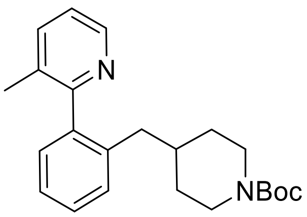

14k

Reaction set up outside of the glovebox using Schlenk technique: The reaction was carried out following general procedure C using 3-methyl-2- phenylpyridine **8r** (68 mg, 0.4 mmol, 1.0 equiv.) and *tert*-butyl 4-(bromomethyl)piperidine-1-carboxylate **12c** (223 mg, 0.8 mmol, 2.0 equiv.). The crude reaction mixture was purified by column chromatography eluting with a 1:1 mixture of EtOAc and CH_2Cl_2 (0–10%) in hexane to give the product **14k** (109 mg, 74%) as a light yellow oil.

^{1}H NMR (400 MHz, $CDCl_3$)

8.42 (dd, *J* = 4.8, 1.8 Hz, 1H), 7.50 (d, *J* = 7.7 Hz, 1H), 7.27 – 7.14 (m, 3H), 7.14 – 7.06 (m, 2H), 3.87 (s, 2H), 2.57 – 2.16 (m, 4H), 2.01 (s, 3H), 1.49 – 1.30 (s, 12H), 0.96 – 0.85 (m, 2H).

^{13}C NMR: (101 MHz, $CDCl_3$)

159.4, 154.8, 146.6, 140.3, 138.0, 137.8, 131.5, 130.1, 129.0, 127.8, 126.1, 122.3, 79.1, 44.0, 39.9, 37.3, 32.0, 28.5, 19.2.

IR: V_{max} (neat/cm^{-1})

2978, 2927, 2849, 1686, 1420, 1364, 1160, 1117, 791.

Spectroscopic data matched those previously reported[12].

Synthesis of [3-Me-2-Phenyl-pyridine]-[cholesterol derivative] 14l

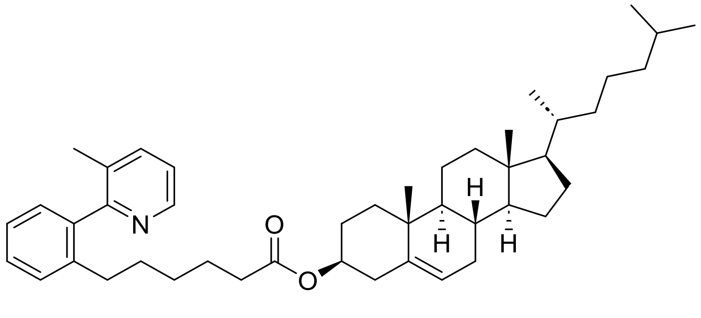

14l

Reaction set up outside of the glovebox using Schlenk technique: The reaction was carried out following general procedure C using 3-methyl-2-phenylpyridine **8r** (34 mg, 0.2 mmol, 1.0 equiv.), cholesterol derivative **12d** (226 mg, 0.4 mmol, 2.0 equiv.), K_3PO_4 (127 mg, 0.4 mmol, 2.0 equiv.), $PhP(O)O_2K_2$ (14 mg, 0.06 mmol, 30 mol%) and **RuAqua** (14.2 mg, 0.02 mmol, 10 mol%). The resulting reaction mixture was allowed to stir for 24 hours. The crude reaction mixture was purified by column chromatography eluting with a 1:1 mixture of EtOAc and CH_2Cl_2 in hexane to give the product **14l** (98 mg, 75%) as a light brown oil.

^{1}H NMR: (400 MHz, $CDCl_3$)

8.46 (dd, *J* = 4.8, 1.9 Hz, 1H), 7.54 (dd, *J* = 7.7, 1.8 Hz, 1H), 7.31 – 7.18 (m, 3H), 7.15 (dd, *J* = 7.7, 4.8 Hz, 1H), 7.13 – 7.08 (m, 1H), 5.33 (d, *J* = 4.9 Hz, 1H), 4.63 – 4.52 (m, 1H), 2.49 – 2.29 (m, 2H), 2.24 (d, *J* = 8.3 Hz, 2H), 2.12 (t, *J* = 7.6 Hz, 2H), 2.07 (s, 3H), 2.02 – 1.88 (m, 2H), 1.86 – 1.73 (m, 3H), 1.59 – 1.02 (m, 24H), 1.02 – 0.90 (m, 6H), 0.88 (d, *J* = 6.6 Hz, 3H), 0.83 (dd, *J* = 6.6, 1.8 Hz, 6H), 0.65 (s, 3H).

^{13}C NMR: (101 MHz, $CDCl_3$)

173.2, 159.7, 146.7, 140.2, 140.0, 139.8, 137.8, 131.6, 129.4, 128.8, 128.1, 125.8, 122.7, 122.3, 73.7, 56.8, 56.2, 50.1, 42.4, 39.8, 39.6, 38.3, 37.1, 36.7, 36.3, 35.9, 34.6, 32.8, 32.0 (2 x resonances), 30.3, 28.9, 28.4, 28.1, 27.9, 24.8, 24.4, 23.9, 22.9, 22.7, 21.1, 19.4, (2 x resonances), 18.8, 12.0.

IR: V_{max} (neat/cm^{-1})

2934, 2866, 1731, 1672, 1462, 1442, 1380, 1257, 1170, 1022, 754.

Spectroscopic data matched those previously reported[12].

RuAqua Catalysed C–H Alkylation of Arenes with Secondary Alkyl Bromides

Attainment of Starting Materials

Directing Group Substrates:

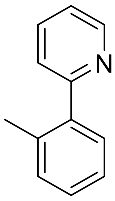

8a

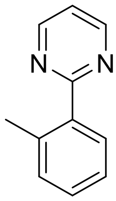

8f

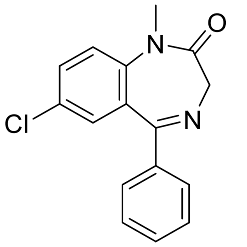

8b

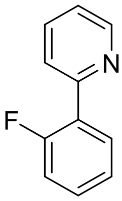

8j

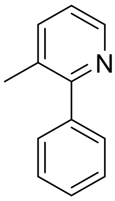

8r

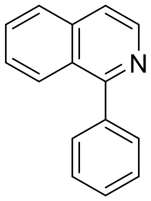

8g

Compounds **8b**, **8g** and **8r** were commercially available. Compound **8r** was purified prior to use via column chromatography eluting with 0–10% EtOAc in hexane.

Compounds **8a**[3], **8f**[8] and **8j**[13] were prepared according to previously reported procedures. The preparation of compounds **8a** and **8f** has been discussed in previous sections.

Preparation of Starting Material 8j

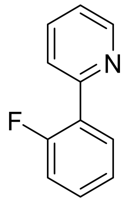

8j

Compound **8j** was prepared according to a previously published procedure with data in accordance.[13]

^{1}H NMR: (400 MHz, $CDCl_3$)

8.76 (s, 1H), 8.01 (d, *J* = 7.9 Hz, 1H), 7.86 – 7.71 (m, 2H), 7.46 – 7.35 (m, 1H), 7.35 – 7.24 (m, 2H), 7.19 (t, *J* = 8.7 Hz, 1H).

^{13}C NMR: (101 MHz, $CDCl_3$)

160.6 (d, *J* = 249.4 Hz), 153.5 (d, *J* = 2.1 Hz), 149.9, 136.5, 131.2 (d, *J* = 3.0 Hz), 130.5 (d, *J* = 8.5 Hz), 127.6 (d, *J* = 11.7 Hz), 124.69 (d, *J* = 5.5 Hz), 124.63, 122.52, 116.30 (d, *J* = 23.0 Hz).

^{19}F NMR: (376 MHz, $CDCl_3$)

–117.8.

Secondary Alkyl Bromides:

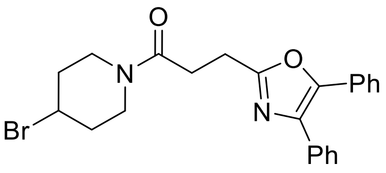

13a
from oxaprozin

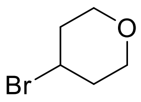

13b

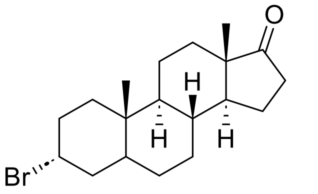

13c
from epiandrosterone

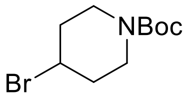

13d

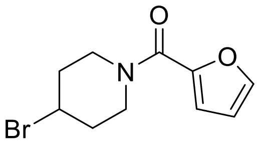

13e

Compounds **13b** and **13d** are commercially available.

Substrates **13a**[14], **13c**[14] and **13e**[15] were synthesized according to described procedures.

Preparation of Starting Material 13a

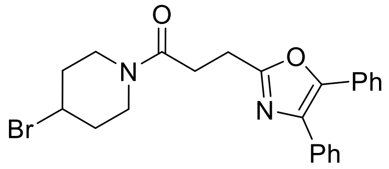

13a

Compound **13a** was prepared according to a previously reported procedure with data in accordance.[14]

^{1}H NMR: (400 MHz, $CDCl_3$)

7.68 – 7.51 (m, 4H), 7.42 – 7.31 (m, 6H), 4.40 (tt, *J* = 7.3, 3.7 Hz, 1H), 3.92 – 3.73 (m, 2H), 3.64 (ddd, *J* = 13.5, 7.2, 3.8 Hz, 1H), 3.47 (ddd, *J* = 13.5, 7.2, 3.8 Hz, 1H), 3.22 (dd, *J* = 8.8, 6.5 Hz, 2H), 2.93 (dd, *J* = 8.8, 6.5 Hz, 2H), 2.19 – 2.05 (m, 2H), 2.05 – 1.92 (m, 2H).

^{13}C NMR: (101 MHz, $CDCl_3$)

169.4, 162.5, 145.3, 135.0, 132.5, 128.9, 128.6, 128.5, 128.4, 128.0, 127.9, 126.4, 48.9, 43.5, 40.0, 35.9, 35.2, 29.8, 23.7.

Preparation of Starting Material 13c

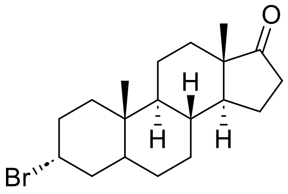

13c

Compound **13c** was prepared according to a previously reported procedure with data in accordance.[14]

^{1}H NMR: (400 MHz, $CDCl_3$)

4.80 – 4.70 (m, 1H), 2.46 (dd, *J* = 19.3, 8.9 Hz, 1H), 2.17 – 2.04 (m, 1H), 2.02 – 1.90 (m, 3H), 1.86 – 1.68 (m, 6H), 1.64 – 1.47 (m, 4H), 1.39 – 1.22 (m, 5H), 1.16 – 1.02 (m, 1H), 0.96 – 0.90 (m, 1H), 0.88 (s, 3H), 0.84 (s, 3H).

^{13}C NMR: (101 MHz, $CDCl_3$)

221.5, 55.8, 54.1, 51.5, 47.9, 40.3, 37.3, 36.5, 36.0, 35.1, 33.0, 31.6, 31.1, 30.8, 27.7, 21.9, 20.2, 14.0, 12.5.

Preparation of Starting Material 13e

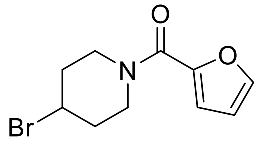

13e

Compound **13e** was prepared according to a previously reported procedure with data in accordance.[15]

^{1}H NMR: (400 MHz, $CDCl_3$)

7.47 (d, *J* = 1.7 Hz, 1H), 6.99 (d, *J* = 3.4 Hz, 1H), 6.48 (dd, *J* = 3.4, 1.7 Hz, 1H), 4.46 (tt, *J* = 7.3, 3.8 Hz, 1H), 4.07 – 3.90 (m, 2H), 3.73 (ddd, *J* = 13.9, 7.3, 3.8 Hz, 2H), 2.30 – 2.13 (m, 2H), 2.12 – 1.89 (m, 2H).

^{13}C NMR: (101 MHz, $CDCl_3$)

159.2, 147.8, 143.6, 116.4, 111.3, 49.0, 44.1 (br), 42.3 (br), 35.8. *(Dynamic NMR effect was observed).*

General Procedure D: RuAqua Catalysed Secondary Alkylation of Arenes with Secondary Alkyl Bromides

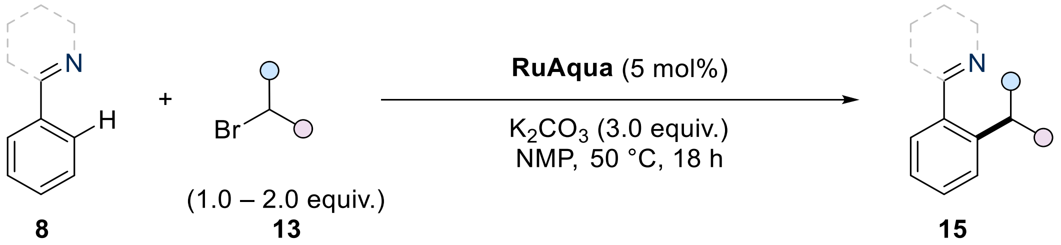

Reaction set up outside of the glovebox using Schlenk technique: Reaction conditions adapted from previously reported procedure[14]. A 10 mL Schlenk tube equipped with a magnetic stirring bar was charged with **RuAqua** (5–10 mol%), K_2CO_3 (3.0 equiv.) the appropriate DG-containing arene **8**, secondary alkyl bromide **13** (1.0–3.0 equiv.) and NMP (0.2 M with respect to **8**) first adding all solids to the reaction vessel before performing 3 x 5 min evac-refill cycles with subsequent addition of oils/solvents. The vial was then stirred at 50 °C for the indicated time. The reaction was then allowed to cool to room temperature before being quenched with H_2O (20 mL) and extracted with Et_2O (3 × 20 mL). The organic extracts were combined, washed with brine (15 mL), dried over $MgSO_4$, filtered and concentrated under reduced pressure. The residue was purified by column chromatography using the noted conditions to yield the desired product **15**.

Synthesis of 3-(4,5-diphenyloxazol-2-yl)-1-(4-(3-methyl-2-(pyridin-2-yl)phenyl)piperidin-1-yl)propan-1-one 15a from oxaprozin derivative

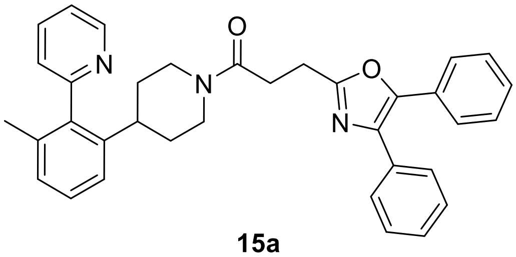

15a

Reaction set up outside of the glovebox using Schlenk technique: General Procedure D was applied using 2-(*o*-tolyl)-pyridine **8a** (68 mg, 0.40 mmol, 1.0 equiv.), oxaprozin derivative **13a** (194 mg, 0.42 mmol, 1.1 equiv.) and **RuAqua** (14.2 mg, 0.02 mmol, 5 mol%). The reaction was stirred at 50 °C for 48 h. The crude mixture was purified by column chromatography eluting with 65% EtOAc in hexane to yield the title compound **15a** (146 mg, 69%) as a colourless oil.

^{1}H NMR: (400 MHz, $CDCl_3$)

8.72 (d, *J* = 5.0 Hz, 1H), 7.78 (d, *J* = 15.3 Hz, 1H), 7.62 (dd, *J* = 8.2, 1.6 Hz, 2H), 7.57 (dd, *J* = 8.2, 1.6 Hz, 2H), 7.39 – 7.27 (m, 7H), 7.25 (t, *J* = 7.7 Hz, 2H), 7.10 (dd, *J* = 15.1, 7.6 Hz, 2H), 4.68 (d, *J* = 13.0 Hz, 1H), 3.93 (d, *J* = 13.4 Hz, 1H), 3.34 – 3.09 (m, 2H), 3.04 – 2.68 (m, 3H), 2.49 – 2.21 (m, 2H), 2.00 (s, 3H), 1.93 – 1.75 (m, 1H), 1.75 – 1.46 (m, 3H).

^{13}C NMR: (101 MHz, $CDCl_3$)

169.3, 162.9, 159.7, 149.8, 145.4, 142.9, 140.0, 136.4, 136.2, 135.2, 132.7, 129.1, 128.7 (2 x resonances), 128.5 (2 x resonances), 128.2, 128.1 (2 x resonances), 126.5, 124.7, 123.5, 122.0, 46.3, 42.7, 39.4, 33.8, 32.8, 30.1, 24.0, 20.6.

IR: V_{max} (neat/cm^{-1})

3057, 2980, 2922, 2852, 1735, 1640, 1443, 761, 693.

Spectroscopic data matched those previously reported[14].

Synthesis of 2-(2-methyl-6-(tetrahydro-2H-pyran-4-yl)phenyl)pyrimidine 15b

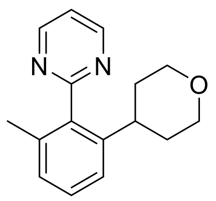

15b

a) Reaction set up outside of the glovebox using Schlenk technique: General Procedure D was applied using 2-(*o*-tolyl)pyrimidine **8f** (68 mg, 0.40 mmol, 1.0 equiv.), **13b** (47 µL, 0.42 mmol, 1.1 equiv.) and **RuAqua** (14.2 mg, 0.02 mmol, 5 mol%). The reaction was stirred at 50 °C for 18 h. The crude mixture was purified by column chromatography eluting with 50% EtOAc in hexane to yield the title compound **15b** (82 mg, 95%) as a colourless amorphous solid.

b) Reaction was set up in a microwave vial in an argon filled glovebox: The general procedure D was applied using $RuCl_3$ (4.1 mg, 0.02 mmol, 5 mol%) in place of **RuAqua**, 2-(*o*-tolyl)pyrimidine **8f** (68 mg, 0.4 mmol, 1.0 equiv.) and **13b** (47 µL, 0.42 mmol, 1.1 equiv.). The reaction was stirred at 50 °C for 18 h. The crude reaction mixture was analysed by quantitative ^{1}H NMR using nitromethane as an internal standard with no product **15b** observed.

c) Reaction was set up in a microwave vial in an argon filled glovebox: The general procedure D was applied using $[Ru(p\text{-cymene})Cl_2]_2$ (6.2 mg, 0.01 mmol, 5 mol% with respect to Ru) in place of **RuAqua**, 2-(*o*-tolyl)pyrimidine **8f** (68 mg, 0.4 mmol, 1.0 equiv.) and **13b** (47 µL, 0.42 mmol, 1.1 equiv.). The reaction was stirred at 50 °C for 18 h. The crude reaction mixture was analysed by quantitative ^{1}H NMR using nitromethane as an internal standard indicating 4% conversion to the product **15b**.

d) Reaction set up outside of the glovebox using Schlenk technique: The general procedure D was applied using $[(C_6H_4CH_2NMe_2)Ru(MeCN)_4]PF_6$ **4** (10.9 mg, 0.02 mmol, 5 mol%) that had been stored as a solid outside of the glovebox for 72 hours in place of **RuAqua**, 2-(*o*-tolyl)pyrimidine **8f** (68 mg, 0.4 mmol, 1.0 equiv.) and **13b** (47 µL, 0.42 mmol, 1.1 equiv.). The reaction was stirred at 50 °C for 18 h. The crude reaction mixture was analysed by quantitative ^{1}H NMR using 1,3,5-trimethoxybenzene as an internal standard indicated no formation of product **15b**.

e) Reaction set up outside of the glovebox using Schlenk technique: The general procedure D was applied using $[Ru(PPy)(MeCN)_4]PF_6$ (11.3 mg, 0.02 mmol, 5 mol%) that had been stored as a solid outside of the glovebox for 72 hours in place of **RuAqua**, 2-(*o*-tolyl)pyrimidine **8f** (68 mg, 0.4 mmol, 1.0 equiv.) and **13b** (47 µL, 0.42 mmol, 1.1 equiv.). The reaction was stirred at 50 °C for 18 h. The crude reaction mixture was analysed by quantitative ^{1}H NMR using 1,3,5-

trimethoxybenzene as an internal standard indicated conversion of 15% to the desired product **15b**.

^{1}H NMR: (400 MHz, $CDCl_3$)

8.83 (d, *J* = 5.0 Hz, 2H), 7.31 – 7.22 (m, 2H), 7.19 (d, *J* = 7.7 Hz, 1H), 7.09 (d, *J* = 7.3 Hz, 1H), 3.90 (dd, *J* = 11.1, 5.1 Hz, 2H), 3.15 (td, *J* = 11.7, 2.3 Hz, 2H), 2.31 (tt, *J* = 11.9, 3.8 Hz, 1H), 1.98 (s, 3H), 1.79 – 1.71 (m, *J* = 12.5, 4.3 Hz, 2H), 1.62 (dd, *J* = 13.1, 2.3 Hz, 2H).

^{13}C NMR: (101 MHz, $CDCl_3$)

168.7, 157.3, 143.1, 138.8, 135.7, 129.0, 128.3, 123.8, 119.3, 68.6, 38.8, 33.9, 20.2.

IR: V_{max} (neat/cm^{-1})

3065, 2972, 2945, 2933, 2855, 2830, 1553, 1410, 1191, 785, 619.

m.p: 128–130 °C (EtOAc)

Spectroscopic data matched those previously reported[14].

Synthesis of 7-chloro-1-methyl-5-(2-(tetrahydro-2H-pyran-4-yl)phenyl)-1,3-dihydro-2H-benzo[e][1,4]diazepin-2-one 15c

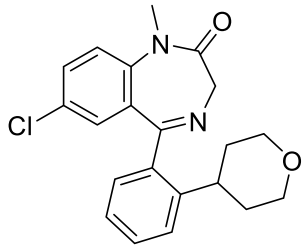

15c

Reaction set up outside of the glovebox using Schlenk technique: General Procedure D was applied using Diazepam **8b** (85 mg, 0.3 mmol, 1.0 equiv.), **13b** (74 mg, 0.5 mmol, 1.5 equiv.) and **RuAqua** (21.3 mg, 0.03 mmol, 10 mol%). The reaction was stirred at 50 °C for 48 h. The crude mixture was purified by column chromatography eluting with 65% EtOAc in hexane to yield compound **15c** (35 mg, 36%) as a colourless oil.

^{1}H NMR: (500 MHz, $CDCl_3$)

7.44 – 7.34 (m, 2H), 7.31 – 7.15 (m, 4H), 6.97 (d, *J* = 2.6 Hz, 1H), 4.80 (d, *J* = 10.8 Hz, 1H), 3.97 – 3.67 (m, 3H), 3.42 (s, 3H), 3.11 (t, *J* = 11.7 Hz, 1H), 2.90 (t, *J* = 12.2 Hz, 1H), 2.33 (t, *J* = 12.1 Hz, 1H), 1.76 – 1.56 (m, 2H), 1.48 (s, 1H), 1.06 (s, 1H).

^{13}C NMR: (126 MHz, $CDCl_3$)

170.8, 169.8, 143.7, 141.3, 138.1, 132.1, 131.5, 130.1, 129.6, 129.4, 129.2, 126.8, 126.3, 122.3, 68.6, 68.3, 56.8, 38.4, 35.0, 33.9, 33.0.

Spectroscopic data matched those previously reported[14].

Synthesis of (8*R*,9*S*,10*S*,13*S*,14*S*)-3-(3-fluoro-2-(pyridin-2-yl)phenyl)-10,13-dimethylhexadecahydro-17H-cyclopenta[a]phenanthren-17-one 15d from epiandrosterone derivative

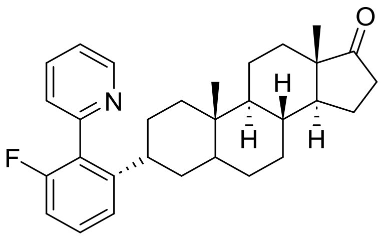

15d

Reaction set up outside of the glovebox using Schlenk technique: General Procedure D was applied using 2-(*o*-fluoro)-pyridine **8j** (69 mg, 0.4 mmol, 1.0 equiv.), **α-13c** (212 mg, 0.6 mmol, 1.5 equiv.) and **RuAqua** (28.4 mg, 0.04 mmol). The reaction was stirred at 50 °C for 18 h. The crude mixture was purified by column chromatography eluting with 10% EtOAc in hexane to yield the title compound **15d** (142 mg, 80%) as a colourless amorphous solid.

^{1}H NMR: (500 MHz, $CDCl_3$)

8.74 – 8.69 (m, 1H), 7.76 (td, *J* = 7.8, 2.0 Hz, 1H), 7.38 – 7.27 (m, 3H), 7.19 (d, *J* = 7.8 Hz, 1H), 6.96 (t, *J* = 8.8 Hz, 1H), 2.53 – 2.37 (m, 2H), 2.10 – 1.98 (m, 1H), 1.94 – 1.84 (m, 1H), 1.81 – 1.32 (m, 10H), 1.31 – 1.11 (m, 5H), 1.08 – 0.71 (m, 9H), 0.69 – 0.60 (m, 1H).

^{13}C NMR: (126 MHz, $CDCl_3$)

221.4, 160.0 (d, *J* = 245.0 Hz), 154.5, 149.6, 148.3 (d, *J* = 1.9 Hz), 136.1, 129.6 (d, *J* = 8.8 Hz), 128.0 (d, *J* = 15.1 Hz), 125.6, 122.4, 121.9 (d, *J* = 3.0 Hz), 112.8 (d, *J* = 22.5 Hz), 54.6, 51.6, 47.9, 46.8, 40.5 (d, *J* = 2.3 Hz), 38.7, 35.9, 35.1, 31.7, 30.9, 29.8, 28.5, 21.8, 20.3, 13.9, 12.5.

^{19}F NMR: (376 MHz, $CDCl_3$)

-115.6.

IR: V_{max} (neat/cm^{-1})

3066, 2979, 2922, 2880, 2853, 1732, 1400, 1240, 775, 714.

m.p: 158–161 °C (EtOAc)

Spectroscopic data matched those previously reported[14].

Further examples of RuAqua Catalysed C–H Secondary Alkylation

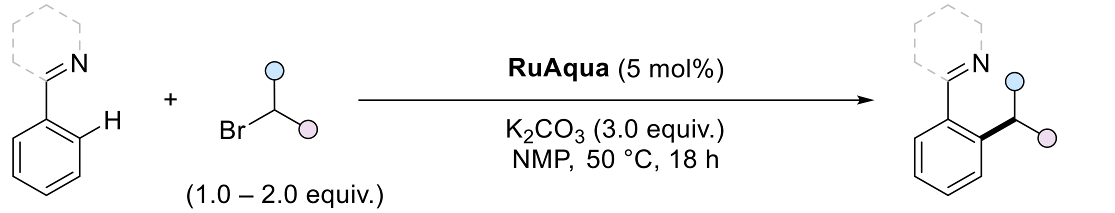

8 **13** **15**

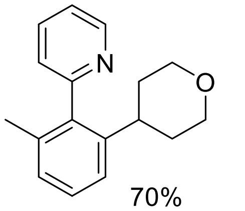

70%
15e

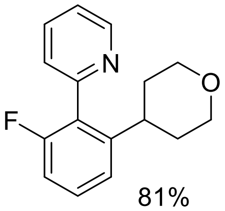

81%
15f

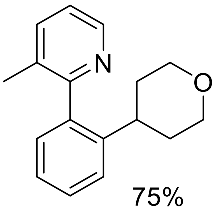

75%
15g

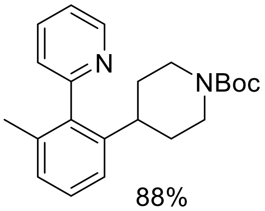

88%
15h

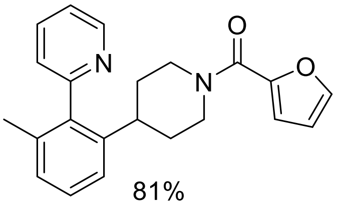

81%
15i

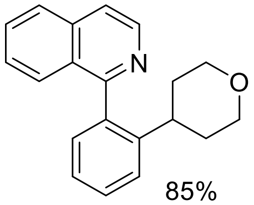

85%
15j

Synthesis of 2-(2-methyl-6-(tetrahydro-2H-pyran-4-yl)phenyl)pyridine 15e

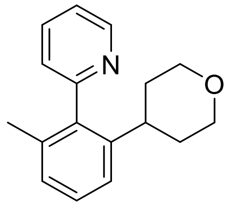

15e

Reaction set up outside of the glovebox using Schlenk technique: The general procedure D was applied using 2-(*o*-tolyl)pyridine **8a** (68 mg, 0.40 mmol, 1.0 equiv.), 4-bromotetrahydro-2H-pyran **13b** (47 µL, 0.42 mmol, 1.1 equiv.) and **RuAqua** (14.2 mg, 0.02 mmol, 5 mol%). The reaction was stirred at 50 °C for 18 h. The crude mixture was purified by column chromatography eluting with 50% EtOAc in hexane to yield the title compound **15e** (71 mg, 70%) as a colourless amorphous solid.

^{1}H NMR: (400 MHz, $CDCl_3$)

8.70 (d, *J* = 5.5 Hz, 1H), 7.74 (t, *J* = 7.7 Hz, 1H), 7.33 – 7.15 (m, 4H), 7.10 (d, *J* = 7.5 Hz, 1H), 3.91 (d, *J* = 9.4 Hz, 2H), 3.16 (t, *J* = 11.4 Hz, 2H), 2.47 – 2.33 (m, 1H), 1.99 (s, 3H), 1.90 – 1.60 (m, 3H), 1.50 (d, *J* = 13.4 Hz, 1H).

^{13}C NMR: (101 MHz, $CDCl_3$)

159.9, 149.9, 143.7, 140.1, 136.4, 136.2, 128.6, 128.1, 124.8, 123.8, 122.1, 68.7, 68.5, 38.4, 34.1, 34.0, 20.7.

IR: V_{max} (neat/cm^{-1})

3044, 2963, 2917, 2827, 1582, 1128, 1086, 784, 754, 621.

m.p: 81–83 °C (EtOAc)

Spectroscopic data matched those previously reported[14].

Synthesis of 2-(2-fluoro-6-(tetrahydro-2H-pyran-4-yl)phenyl)pyridine 15f

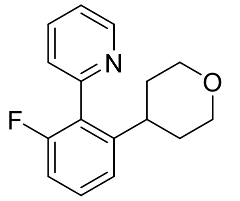

15f

Reaction set up outside of the glovebox using Schlenk technique: The general procedure D was applied using 2-(2-fluorophenyl)pyridine **8j** (69 mg, 0.40 mmol, 1.0 equiv.), 4-4-bromotetrahydro-2H-pyran **13b** (47 µL, 0.42 mmol, 1.1 equiv.) and **RuAqua** (14.2 mg, 0.02 mmol, 5 mol%). The reaction was stirred at 50 °C for 18 h. The crude mixture was purified by column chromatography eluting with 10% EtOAc in hexane to yield the title compound **15f** (98 mg, 81%) as a colourless oil.

^{1}H NMR: (400 MHz, $CDCl_3$)

8.66 (d, *J* = 5.0 Hz, 1H), 7.70 (m, 1H), 7.34 – 7.15 (m, 3H), 7.11 (d, *J* = 7.9 Hz, 1H), 7.00 – 6.93 (m, 1H), 3.95 – 3.80 (m, 2H), 3.20–3.10 (t, *J* = 11.8 Hz, 2H), 2.75 – 2.60 (m, 1H), 1.80 – 1.65 (m, 2H), 1.63 – 1.50 (m, 2H).

^{13}C NMR: (101 MHz, $CDCl_3$)

160.2 (d, *J* = 245.5 Hz), 154.3, 149.8, 146.8 (d, *J* = 2.0 Hz), 136.4, 130.1 (d, *J* = 8.8 Hz), 128.2 (d, *J* = 15.6 Hz), 125.9 (d, *J* = 2.0 Hz), 122.7, 122.1 (d, *J* = 3.4 Hz), 113.5 (d, *J* = 22.5 Hz), 68.5, 37.7 (d, *J* = 2.4 Hz), 33.9.

^{19}F NMR: (376 MHz, $CDCl_3$)

–115.4.

IR: V_{max} (neat/cm^{-1})

3057, 2947, 2919, 2757, 1584, 1567, 1231, 1125, 1083, 804, 779, 748.

Spectroscopic data matched those previously reported[14].

Synthesis of 3-methyl-2-(2-(tetrahydro-2H-pyran-4-yl)phenyl)pyridine 15g

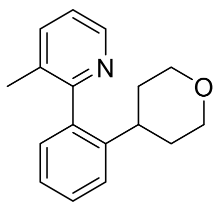

15g

Reaction set up outside of the glovebox using Schlenk technique: The general procedure D was applied using 3-Me-2-phenylpyridine **8r** (64 µL, 0.4 mmol, 1.0 equiv.), 4-bromotetrahydro-2H-pyran **13b** (67 µL, 0.6 mmol, 1.5 equiv.) and **RuAqua** (14.2 mg, 0.02 mmol, 5 mol%). The reaction was stirred at 50 °C for 18 h. The ^{1}H NMR analysis of the reaction crude indicated a greater than 20:1 ratio between *ortho*-product and *meta*-product. The crude mixture was purified by column chromatography eluting with 30% EtOAc in hexane to yield the title compound **15g** (76 mg, 75%) as a colourless oil.

^{1}H NMR: (500 MHz, $CDCl_3$)

8.50 (dd, *J* = 4.8, 2.4 Hz, 1H), 7.60 (ddd, *J* = 7.8, 1.8, 0.9 Hz, 1H), 7.42 – 7.36 (m, 2H), 7.30 – 7.24 (m, 2H), 7.22 (dd, *J* = 7.7, 4.8 Hz, 1H), 7.13 (dt, *J* = 7.5, 1.1 Hz, 1H), 3.94 (dt, *J* = 11.4, 3.2 Hz, 2H), 3.28 – 3.17 (m, 2H), 2.51 (ddd, *J* = 15.7, 11.5, 4.1 Hz, 1H), 2.10 (s, 3H), 1.96 – 1.65 (m, 3H), 1.47 (s, 1H).

^{13}C NMR: (126 MHz, $CDCl_3$)

159.8, 147.0, 143.5, 139.9, 138.1, 131.9, 129.2, 128.7, 126.6, 126.4, 122.6, 68.8, 38.2, 19.8.

IR: V_{max} (neat/cm^{-1})

3050, 2980, 2952, 2934, 2843, 1581, 1566, 1115, 752.

Spectroscopic data matched those previously reported[14].

Synthesis of *tert*-butyl 4-(3-methyl-2-(pyridin-2-yl)phenyl)piperidine-1-carboxylate 15h

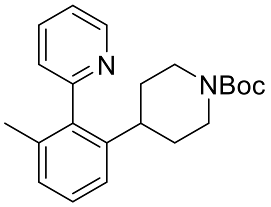

15h

Reaction set up outside of the glovebox using Schlenk technique: The general procedure D was applied using 2-(*o*-tolyl)pyridine **8a** (68 mg, 0.4 mmol, 1.0 equiv.), *tert*-butyl 4-bromopiperidine-1-carboxylate **13d** (132 mg, 0.5 mmol, 1.3 equiv.) and **RuAqua** (14.2 mg, 0.02 mmol, 5 mol%). The reaction was stirred at 50 °C for 18 h. The crude mixture was purified by column chromatography eluting with 35% EtOAc in hexane to yield the title compound **15h** (124 mg, 88%) as a colourless oil.

^{1}H NMR: (400 MHz, $CDCl_3$)

8.41 (dd, *J* = 4.9, 1.8 Hz, 1H), 7.51 (dd, *J* = 7.8, 1.8 Hz, 1H), 7.32 – 7.22 (m, 2H), 7.22 – 7.08 (m, 2H), 7.04 (dd, *J* = 7.5, 1.4 Hz, 1H), 4.36 – 3.70 (m, 2H), 2.50 – 2.22 (m, 3H), 2.01 (s, 3H), 1.84 – 1.40 (m, 4H), 1.36 (s, 9H).

^{13}C NMR: (101 MHz, $CDCl_3$)

159.4, 154.8, 146.6, 143.2, 139.5, 137.8, 131.6, 128.9, 128.4, 126.1 (2 x resonances), 122.3, 79.3, 44.4, 38.9, 33.3, 32.3, 28.5, 19.5.

IR: V_{max} (neat/cm^{-1})

3050, 2976, 2931, 2851, 1738, 1687, 1420, 1229, 1162, 792, 754.

MS: HRMS (ESI$^+$)

Calculated for $C_{22}H_{29}O_2N_2$: 353.2224, found: 353.2225.

Synthesis of furan-2-yl(4-(3-methyl-2-(pyridin-2-yl)phenyl)piperidin-1-yl)methanone 15i

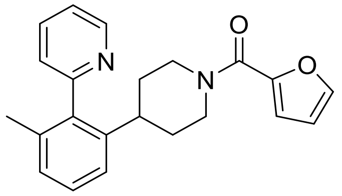

15i

Reaction set up outside of the glovebox using Schlenk technique: General procedure D was applied using 2-(*o*-tolyl)pyridine **8a** (64 µL, 0.4 mmol, 1 equiv.), (4-bromopiperidin-1-yl)(furan-2-yl)methanone **13e** (208 mg, 0.6 mmol, 1.5 equiv.) and **RuAqua** (14.2 mg, 0.02 mmol, 5 mol%). The reaction was allowed to stir at 50 °C for 18 h. The crude mixture was purified by column chromatography eluting with 50% EtOAc in hexane to yield the title compound **15i** (133 mg, 81%) as a colourless oil.

^{1}H NMR: (500 MHz, $CDCl_3$)

8.50 (d, *J* = 4.9 Hz, 1H), 7.60 (d, *J* = 7.6 Hz, 1H), 7.44 (s, 1H), 7.39 – 7.30 (m, 2H), 7.27 – 7.18 (m, 2H), 7.13 (d, *J* = 7.6 Hz, 1H), 6.92 (d, *J* = 3.4 Hz, 1H), 6.44 (dd, *J* = 3.5, 1.8 Hz, 1H), 4.55 (bs, 2H), 3.22 – 2.40 (m, 3H), 2.11 (s, 3H), 2.04 – 1.50 (m, 4H).

^{13}C NMR: (101 MHz, $CDCl_3$)

159.4, 159.3, 148.2, 146.7, 143.6, 142.7, 139.6, 138.0, 131.7, 129.0, 128.5, 126.2, 122.5, 115.8, 111.2, 47.0, 43.1, 39.2, 33.8, 32.8, 19.6.

IR: V_{max} (neat/cm^{-1})

3110, 3055, 2981, 2922, 2854, 1734, 1619, 1430, 1239, 997, 752.

MS: HRMS (ESI$^+$)

Calculated for $C_{22}H_{23}O_2N_2$: 347.1754, found: 347.1745.

Synthesis of 1-(2-(tetrahydro-2H-pyran-4-yl)phenyl)isoquinoline 15j

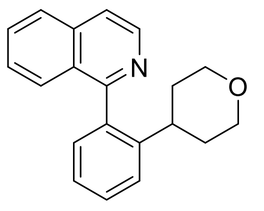

15j

Reaction set up outside of the glovebox using Schlenk technique: General Procedure D was applied using **8g** (94 mg, 0.40 mmol, 1.0 equiv.), **13b** (47 µL, 0.42 mmol, 1.1 equiv.) and **RuAqua** (14.2 mg, 0.02 mmol). The reaction was stirred at 50 °C for 18 h. The crude mixture was purified by column chromatography eluting with 50% EtOAc in hexane to yield the title compound **15j** (98 mg, 85%) as a colourless oil.

^{1}H NMR: (400 MHz, $CDCl_3$)

8.61 (d, *J* = 5.7 Hz, 1H), 7.91 (d, *J* = 8.3 Hz, 1H), 7.74 – 7.66 (m, 2H), 7.63 (d, *J* = 7.6 Hz, 1H), 7.55 – 7.40 (m, 3H), 7.42 – 7.14 (m, 2H), 3.95 – 3.87 (m, 1H), 3.88 – 3.77 (m, 1H), 3.15 (td, *J* = 11.4, 3.7 Hz, 1H), 3.03 – 2.93 (m, 1H), 2.50 (m, 1H), 1.90 – 1.71 (m, 3H), 1.41 – 1.33 (m, 1H).

^{13}C NMR: (101 MHz, $CDCl_3$)

161.2, 144.3, 142.1, 138.3, 136.3, 130.2, 129.9, 128.9, 128.0, 127.4, 127.2, 126.9, 126.4, 125.9, 120.0, 68.4, 68.2, 38.0, 34.1, 33.3.

IR: V_{max} (neat/cm^{-1})

3051, 2919, 2848, 1647, 1499, 751.

Spectroscopic data matched those previously reported[14].

RuAqua Catalysed C–H Secondary Alkylation Using Acyclic Alkyl Bromides

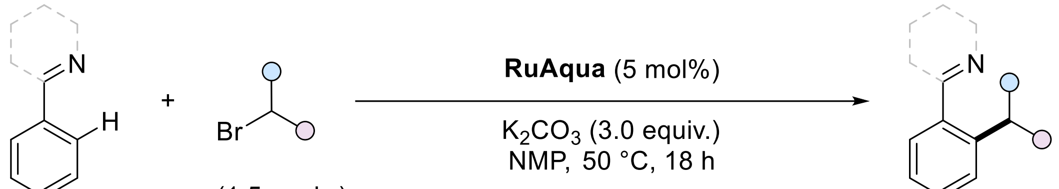

(1.5 equiv.)

8 **13** **15**

Unsuccessful substrates:

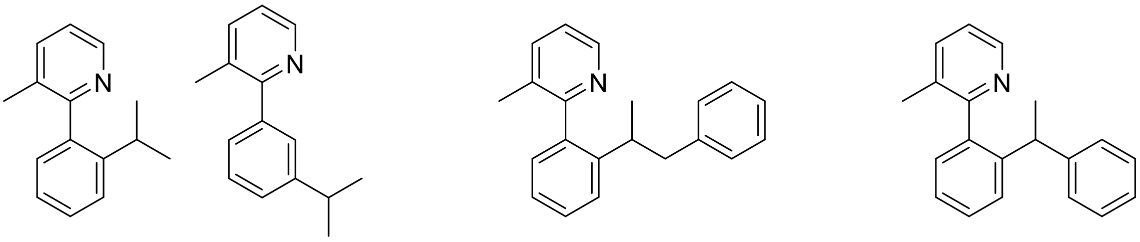

15k **15k'** **15l** **15m**

28% overall yield
mixture of *ortho*- and *meta*-
functionalised product
(**15k**:**15k'** = 2.1:1)

trace by GCMS

trace by GCMS

To further expand the scope of **RuAqua** catalysed C–H secondary alkylation, several acyclic secondary alkyl bromides were explored. Unfortunately, these either gave a mixture of *ortho*- and *meta*-functionalised products (**15k** and **15k'**) or only trace amounts of the product could be detected by GCMS.

Experimental Procedure

Reactions were set up outside the glovebox using Schlenk technique. Alkyl bromides were commercially available and used directly without further purification. The general procedure D was applied using 3-Me-2-phenylpyridine **8r** (64 µL, 0.4 mmol, 1.0 equiv.), the appropriate alkyl bromide **13** (0.6 mmol, 1.5 equiv.) and **RuAqua** (14.2 mg, 0.02 mmol, 5 mol%). The reaction was stirred at 50 °C for 72 h. GCMS analysis showed only trace amounts of products **15l** and **15m**. For **15k** and **15k'** the crude mixture was purified by column chromatography eluting with 10% EtOAc in hexane to give a mixture of **15k** and **15k'** (2.1:1 ratio, 24 mg, 28% overall) as a colourless oil. NMR data was in accordance with previous reports.[14]

RuAqua Catalysed C–H Methylation of Arenes with Anilinium Salt

Attainment of Starting Materials

Directing Group Substrates:

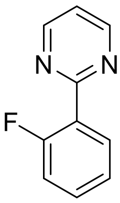

8k

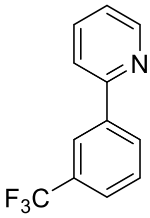

8l

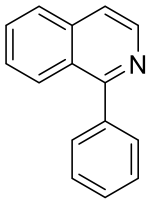

8g

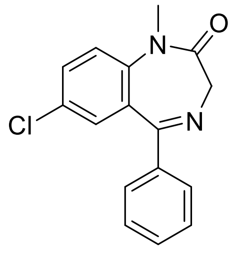

8b

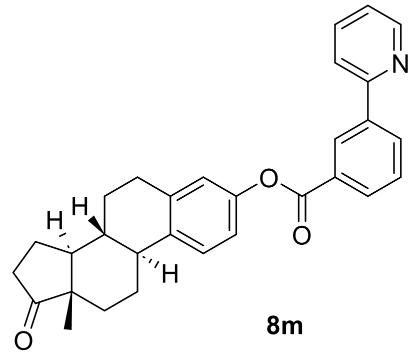

8m

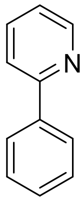

8n

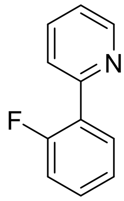

8j

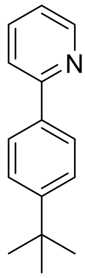

8s

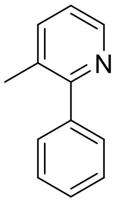

8r

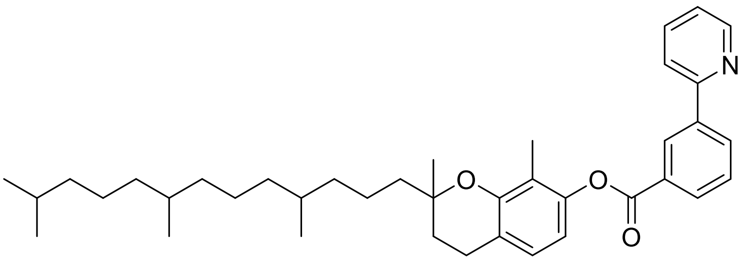

8t

Compounds **8b**, **8g, 8n** and **8r** were commercially available. **8n** and **8r** were purified via column chromatography eluting with 0–10% EtOAc in hexane prior to use removing colour from the reactants.

Compounds **8j**[13], **8k**[15], **8l**[3], **8m**[5], **8s**[17] and **8t**[5] were prepared according to previously reported protocols. The preparation of **8j** has been discussed in previous sections.

Preparation of Starting Material 8k

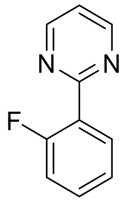

8k

Compound **8k** was prepared to a previously reported procedure with data in accordance.[15]

^{1}H NMR: (500 MHz, $CDCl_3$)

8.87 (dd, *J* = 4.9, 1.4 Hz, 2H), 8.07 (t, *J* = 7.8 Hz, 1H), 7.49 – 7.41 (m, 1H), 7.32 – 7.14 (m, 3H).

^{13}C NMR: (126 MHz, $CDCl_3$)

163.5 (d, *J* = 4.4 Hz), 161.2 (d, *J* = 254.8 Hz), 157.3, 132.0, 132.0, 126.5 (d, *J* = 9.8 Hz), 124.3 (d, *J* = 3.9 Hz), 119.2, 116.9 (d, *J* = 22.5 Hz).

^{19}F NMR: (471 MHz, $CDCl_3$)

–115.3.

Preparation of Starting Material 8l

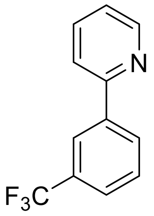

8l

Compound **8l** was prepared to a previously reported procedure with data in accordance.[3]

^{1}H NMR: (500 MHz, $CDCl_3$)

8.22 (d, *J* = 4.9 Hz, 1H), 7.78 (s, 1H), 7.67 (d, *J* = 7.8 Hz, 1H), 7.34 – 7.21 (m, 2H), 7.19 – 7.13 (m, 1H), 7.08 (t, *J* = 7.7 Hz, 1H), 6.82 – 6.74 (m, 1H).

^{13}C NMR: (126 MHz, $CDCl_3$)

156.0, 150.0, 140.2, 137.2, 131.4 (q, *J* = 32.3 Hz), 130.2, 129.4, 125.7 (q, *J* = 3.9 Hz), 124.3 (q, J = 272.6 Hz) 123.94 (q, *J* = 3.9 Hz), 123.0, 120.7.

^{19}F NMR: (471 MHz, $CDCl_3$)

–62.6.

Preparation of Starting Material 8m

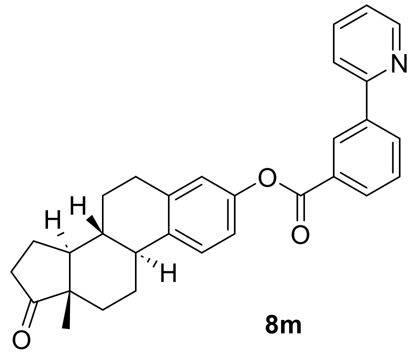

8m

Compound **8m** was prepared to a previously reported procedure with data in accordance.[5]

^{1}H NMR: (400 MHz, $CDCl_3$)

8.79 (t, J = 1.6 Hz, 1H), 8.76 – 8.70 (m, 1H), 8.34 – 8.28 (m, 1H), 8.27 – 8.20 (m, 1H), 7.86 – 7.77 (m, 2H), 7.63 (t, J = 7.8 Hz, 1H), 7.35 (d, J = 8.4 Hz, 1H), 7.29 (ddd, J = 6.8, 4.8, 2.0 Hz, 1H), 7.01 (dd, J = 8.4, 2.5 Hz, 1H), 6.97 (d, J = 2.4 Hz, 1H), 3.00 – 2.90 (m, 2H), 2.52 (dd, J = 18.9, 8.6 Hz, 1H), 2.48 – 2.41 (m, 1H), 2.38 – 2.29 (m, 1H), 2.21 – 1.95 (m, 4H), 1.70 – 1.59 (m, 3H), 1.56 – 1.42 (m, 3H), 0.93 (s, 3H).

^{13}C NMR: (101 MHz, $CDCl_3$)

220.9, 165.5, 156.4, 150.0, 149.0, 140.1, 138.2, 137.6, 137.1, 132.1, 130.7, 130.4, 129.2, 128.6, 126.6, 122.8, 121.9, 120.8, 119.0, 50.6, 48.1, 44.3, 38.2, 36.0, 31.7, 29.6, 26.5, 25.9, 21.7, 14.0.

Preparation of Starting Material 8s

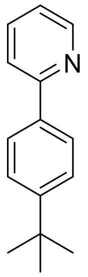

8s

Compound **8s** was prepared to a previously reported procedure with data in accordance.[17]

^{1}H NMR: (400 MHz, $CDCl_3$)

8.68 (d, *J* = 4.6 Hz, 1H), 7.94 (d, *J* = 8.4 Hz, 2H), 7.78 – 7.68 (m, 2H), 7.51 (d, *J* = 8.6 Hz, 2H), 7.24 – 7.16 (m, 1H), 1.37 (s, 9H).

^{13}C NMR: (101 MHz, $CDCl_3$)

157.5, 152.3, 149.7, 136.8, 136.7, 126.7, 125.9, 121.9, 120.5, 34.8, 31.4.

Preparation of Starting Material 8t

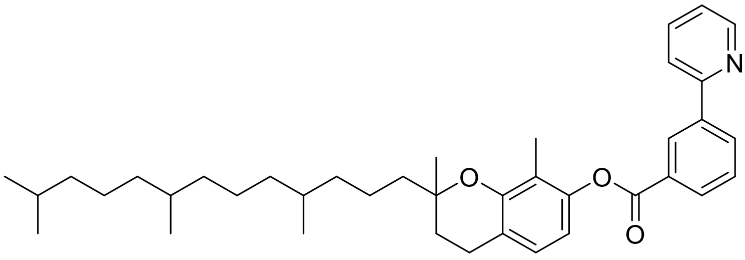

8t

Compound **8t** was prepared to a previously reported procedure with data in accordance.[5]

^{1}H NMR: (400 MHz, CDCl3)

8.77 (app. t, J = 1.6 Hz, 1H), 8.75 – 8.72 (m, 1H), 8.32 – 8.29 (m, 1H), 8.25 – 8.21 (m, 1H), 7.84 – 7.76 (m, 2H), 7.62 (t, J = 7.8 Hz, 1H), 7.28 (ddd, J = 6.8, 4.8, 1.8 Hz, 1H), 6.83 (d, J = 2.5 Hz, 1H), 6.78 (d, J = 2.7 Hz, 1H), 2.83 – 2.73 (m, 2H), 2.19 (s, 3H), 1.89 – 1.71 (m, 2H), 1.65 – 1.33 (m, 9H), 1.33 – 1.01 (m, 15H), 0.93 – 0.82 (m, 12H).

^{13}C NMR: (101 MHz, $CDCl_3$)

165.7, 156.3, 149.9, 149.9, 142.7, 139.9, 136.9, 131.8, 130.5, 129.0, 128.4, 127.4, 122.6, 121.3, 121.1, 120.7, 119.2, 77.3, 76.2, 40.2, 39.4, 37.5, 37.5, 37.3, 32.8, 32.7, 31.1, 28.0, 24.8, 24.5, 24.3, 22.8, 22.7, 22.5, 21.0, 19.8, 19.7, 16.2.

General Procedure E: RuAqua Catalysed Methylation of Arenes with Anilinium Salt 16

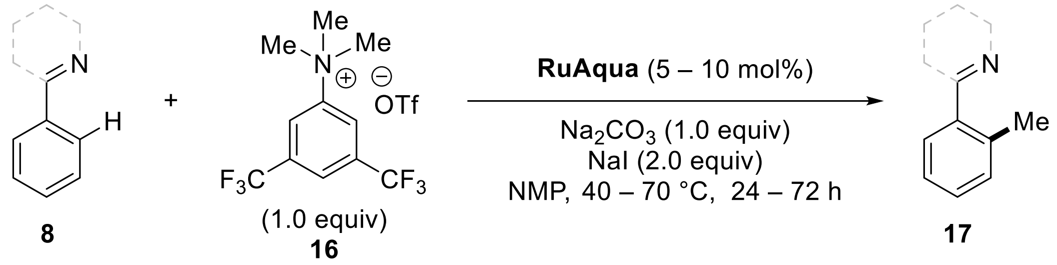

Reaction set up outside of the glovebox using Schlenk technique: Reaction conditions adapted from previously reported procedure[5]. To an oven-dried 10 mL Schlenk tube equipped with a magnetic stirring bar was added **RuAqua** (5–10 mol%), NaI (90 mg, 0.6 mmol, 2.0 equiv.), anilinium salt **16** (56 mg, 0.3 mmol, 1.0–2.0 equiv.), Na_2CO_3 (32 mg, 0.3 mmol, 1.0 equiv.) and the appropriate DG-containing arene **8** (0.3 mmol, 1.0 equiv.) and NMP (1.5 mL, 0.2 M with respect to **8**) first adding all solids to the reaction vessel before performing 3 x 5 min evac-refill cycles with subsequent addition of any degassed oils/solvents. The reaction mixture was stirred at the stated temperature for the indicated time. Upon completion, the crude reaction mixture was diluted with water (20 mL) and extracted with Et_2O (3 x 20 mL) before being loaded onto a silica gel column and purified by flash column chromatography under the conditions noted to afford pure product.

Anilinium salt **16** was prepared according to previously reported procedure. [5]

Preparation of Starting Material 16

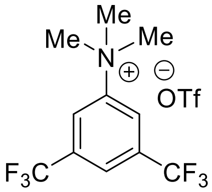

16

Starting Material **16** was prepared according to previously reported procedures.[5]

^{1}H NMR: (400 MHz, d_6-Acetone)

8.84 (s, 2H), 8.40 (s, 1H), 4.10 (s, 9H).

^{13}C NMR: (126 MHz, d_6-Acetone)

148.7, 133.1 (q, J = 34.5 Hz), 124.6 (hept, J = 3.5 Hz), 122.7 (q, J = 2.5 Hz), 122.7 (q, J = 273.3 Hz), 122.2 (q, J = 321.6 Hz), 57.2.

^{19}F NMR: (471 MHz, d_6-Acetone)

–63.2, –79.0.

Synthesis of 2-(2-fluoro-6-methylphenyl)pyrimidine 17a

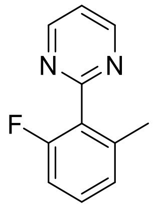

17a

Reaction set up outside of the glovebox using Schlenk technique: The title compound was synthesized as outlined in general procedure E, using 2-(*o*-fluroro)pyrimidine **8k** (52 mg, 0.3 mmol, 1.0 equiv.) and **RuAqua** (10.6 mg, 0.015 mmol, 5 mol%) for 24 hours. Quantitative ^{1}H NMR using nitromethane as an internal standard indicated conversion of 80%. Column chromatography eluting with 5–15% EtOAc in hexane (50 g silica gel cartridge, gradient over 15 CVs, 40 mL / min flowrate) gave the product **17a** as a cloudy oil (37 mg, 66%).

^{1}H NMR: (500 MHz, $CDCl_3$)

8.85 (d, *J* = 4.9 Hz, 2H), 7.29 – 7.21 (m, 2H), 7.03 (d, *J* = 7.6 Hz, 1H), 6.97 (t, 1H, *J* = 9.0 Hz), 2.20 (s, 3H).

^{13}C NMR: (126 MHz, $CDCl_3$)

164.1, 160.5 (d, *J* = 247.5 Hz), 157.3, 138.8 (d, *J* = 2.4 Hz), 130.1 (d, *J* = 8.8 Hz), 127.5 (d, *J* = 14.7 Hz), 126.1, 119.5, 113.3 (d, *J* = 22.0 Hz), 19.6 (d, *J* = 2.9 Hz).

^{19}F NMR: (376 MHz, $CDCl_3$)

–117.6.

IR: V_{max} (neat/cm^{-1})

3036, 2963, 1618, 1578, 1409, 1255, 779.

Spectroscopic data matched those previously reported[5].

Synthesis of 2-(2-methyl-5-(trifluoromethyl)phenyl)pyridine 17b

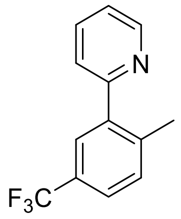

17b

Reaction set up outside of the glovebox using Schlenk technique: The title compound was synthesized as outlined in general procedure E, using 2-(3-(trifluoromethyl)phenyl)pyridine **8l** (71 mg, 0.3 mmol, 1.0 equiv.) and **RuAqua** (10.6 mg, 15 μmol, 5 mol%) for 24 hours. Quantitative ^{1}H NMR using nitromethane as an internal standard indicated conversion of 98%. Column chromatography eluting with 0–10% EtOAc in hexane (50 g silica gel cartridge, gradient over 15 CVs, 40 mL / min flowrate) gave the product **17b** as an cloudy oil (53 mg, 75%).

^{1}H NMR: (400 MHz, $CDCl_3$)

8.72 (d, *J* = 4.9 Hz, 1H), 7.78 (td, *J* = 7.8, 1.9 Hz, 2H), 7.66 (s, 1H), 7.55 (dd, *J* = 8.1, 2.3 Hz, 2H), 7.40 (t, *J* = 7.4 Hz, 2H), 7.35 – 7.25 (m, 1H), 2.42 (s, 3H).

^{13}C NMR: (101 MHz, $CDCl_3$)

158.7, 149.6, 141.1, 140.2 (q, *J* = 1.4 Hz), 136.6, 131.3, 128.5 (q, *J* = 32.5 Hz), 126.7 (q, *J* = 3.8 Hz), 125.0 (q, *J* = 3.7 Hz), 124.4 (q, *J* = 272.2 Hz), 124.2, 122.4, 20.5.

^{19}F NMR: (376 MHz, $CDCl_3$)

-62.3.

IR: V_{max} (neat/cm^{-1})

2929, 1620, 1335, 1118, 1076, 1060, 748.

Spectroscopic data matched those previously reported[5].

Synthesis of 1-(*o*-tolyl)isoquinoline 17c

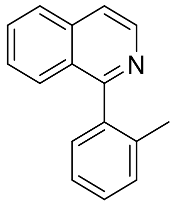

17c

a) Reaction set up outside of the glovebox using Schlenk technique: The title compound was synthesized as outlined in general procedure E, using 1-phenylisoquinoline **8g** (62 mg, 0.3 mmol, 1.0 equiv.) and **RuAqua** (10.6 mg, 15 μmol, 5 mol%) for 24 hours. After this time, quantitative ^{1}H NMR using nitromethane as internal standard indicated conversion to product **17c** of 82%. Column chromatography eluting with 0–10% EtOAc in hexane (50 g silica gel cartridge, gradient over 15 CVs, 40 mL / min flowrate) gave the product **17c** as a yellow oil (48 mg, 73%).

b) Reaction was set up in a microwave vial in an argon filled glovebox: The general procedure E was applied using $RuCl_3$ (3.6 mg, 0.02 mmol, 5 mol%) in place of **RuAqua** and 1-phenylisoquinoline **8g** (62 mg, 0.3 mmol, 1.0 equiv.) stirring for 24 hours. The crude reaction mixture was analysed by quantitative ^{1}H NMR using nitromethane as an internal standard with no product **17c** observed.

c) Reaction was set up in a microwave vial in an argon filled glovebox: The general procedure E was applied using $[Ru(p\text{-cymene})Cl_2]_2$ (4.7 mg, 0.01 mmol, 5 mol% with respect to Ru) in place of **RuAqua** and 1-phenylisoquinoline **8g** (62 mg, 0.3 mmol, 1.0 equiv.) stirring for 24 hours. The crude reaction mixture was analysed by quantitative ^{1}H NMR using nitromethane as an internal standard indicating ~1% conversion to the product **17c**.

d) Reaction set up outside of the glovebox using Schlenk technique: The general procedure E was applied using $[(C_6H_4CH_2NMe_2)Ru(MeCN)_4]PF_6$ **4** (10.9 mg, 0.02 mmol, 5 mol%) in place of **RuAqua** and 1-phenylisoquinoline **8g** (62 mg, 0.3 mmol, 1.0 equiv.) stirring for 24 hours. The crude reaction mixture was analysed by quantitative ^{1}H NMR using 1,3,5-trimethoxybenzene as an internal standard indicating no formation of the desired product 17c.

e) Reaction set up outside of the glovebox using Schlenk technique: The general procedure E was applied using $[Ru(PPy)(MeCN)_4]PF_6$ **4** (11.3 mg, 0.02 mmol, 5 mol%) in place of **RuAqua** and 1-phenylisoquinoline **8g** (62 mg, 0.3 mmol, 1.0 equiv.) stirring for 24 hours. The crude reaction mixture was analysed by quantitative ^{1}H NMR using 1,3,5-trimethoxybenzene as an internal standard indicating <1% conversion to the product **17c**.

^{1}H NMR: (400 MHz, $CDCl_3$)

8.62 (d, *J* = 5.7 Hz, 1H), 7.89 (d, *J* = 8.4 Hz, 1H), 7.74 – 7.62 (m, 3H), 7.48 (ddd, *J* = 8.2, 6.9, 1.4 Hz, 1H), 7.42 – 7.29 (m, 4H), 2.07 (s, 3H).

^{13}C NMR: (101 MHz, $CDCl_3$)

161.5, 142.3, 139.0, 136.4 (2 x resonances), 130.3, 130.1, 129.6, 128.4, 127.5 (2 x resonances), 127.2, 126.9, 125.6, 119.9, 19.8.

IR: V_{max} (neat/cm^{-1})

3048, 2952, 2921, 1556, 1382, 824, 726, 679.

Spectroscopic data matched those previously reported[5].

Synthesis of 7-chloro-1-methyl-5-(*o*-tolyl)-1,3-dihydro-2H-benzo[e][1,4]diazepin-2-one 17d from diazepam

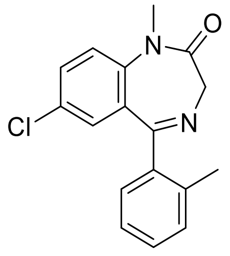

17d

Reaction set up outside of the glovebox using Schlenk technique: The title compound was synthesized as outlined in general procedure E, using diazepam **8b** (57 mg, 0.2 mmol, 1.0 equiv.) and **RuAqua** (21.2 mg, 0.03 mmol, 10 mol%). After this time, quantitative ^{1}H NMR using nitromethane as internal standard indicated conversion to product **17d** of 77%. Purification using flash column chromatography eluting with 25–35% EtOAc in hexane (50 g silica gel cartridge, gradient over 15 CVs) afforded the product **17d** as a colourless amorphous solid (45 mg, 75 %).

^{1}H NMR: (400 MHz, $CDCl_3$)

7.47 (dd, *J* = 8.7, 2.5 Hz, 1H), 7.39 – 7.31 (m, 2H), 7.30 – 7.23 (m, 2H), 7.18 (d, *J* = 7.1 Hz, 1H), 7.05 (d, *J* = 2.6 Hz, 1H), 4.86 (d, *J* = 11.0 Hz, 1H), 3.81 (d, *J* = 11.0 Hz, 1H), 3.43 (s, 3H), 1.98 (s, 3H).

^{13}C NMR: (101 MHz, $CDCl_3$)

170.9, 170.0, 141.8, 138.8, 136.4, 131.9, 131.6, 131.5, 131.0, 129.9, 129.8, 129.1, 126.1, 122.7, 56.9, 34.9, 20.1.

IR: V_{max} (neat/cm^{-1})

3019, 2974, 1670, 1614, 763, 722, 696.

m.p: 126–128 °C (EtOAc)

Spectroscopic data matched those previously reported[5].

Synthesis of (8*R*,9*S*,13*S*,14*S*)-13-methyl-17-oxo-7,8,9,11,12,13,14,15,16,17-decahydro-6H-cyclopenta[a]phenanthren-3-yl 4-methyl-3-(pyridin-2-yl)benzoate 17e from estrone derivative

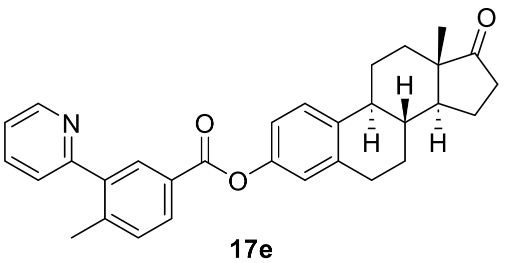

17e

Reaction set up outside of the glovebox using Schlenk technique: The title compound was synthesized as outlined in general procedure E, estrone derivative **8m** (136 mg, 0.3 mmol, 1.0 equiv.) and **RuAqua** (10.6 mg, 0.015 mmol, 5 mol%) were employed. The reaction was allowed to stir for 24 hours. After this time, quantitative ^{1}H NMR using nitromethane as internal standard indicated conversion to product **17e** of 92%. Purification using flash column chromatography eluting with 15–25% (50 g silica gel cartridge, gradient over 15 CVs, 40 mL / min flowrate) EtOAc in hexane afforded the product **17e** as colourless needles (113 mg, 81%).

^{1}H NMR: (400 MHz, $CDCl_3$)

8.74 – 8.71 (m, 1H), 8.22 (d, *J* = 2.0 Hz, 1H), 8.11 (dd, *J* = 7.9, 2.0 Hz, 1H), 7.79 (td, *J* = 7.7, 1.8 Hz, 1H), 7.46 (dt, *J* = 7.8, 1.1 Hz, 1H), 7.42 (d, *J* = 7.9 Hz, 1H), 7.35 – 7.27 (m, 2H), 6.98 (dd, *J* = 8.8, 2.9 Hz, 1H), 6.94 (d, *J* = 2.6 Hz, 1H), 2.97 – 2.89 (m, 2H), 2.56 – 2.39 (m, 5H), 2.37 – 2.27 (m, 1H), 2.21– 1.94 (m, 4H), 1.70 –1.41 (m, 6H), 0.93 (s, 3H).

^{13}C NMR: (101 MHz, $CDCl_3$)

220.1, 165.5, 159.1, 149.5, 149.0, 142.5, 141.0, 138.2, 137.5, 136.6, 131.5, 131.3, 130.0, 127.6, 126.6, 124.3, 122.3, 121.9, 119.1, 50.6, 48.1, 44.3, 38.2, 36.0, 31.7, 29.6, 26.5, 25.9, 21.8, 20.8, 14.0.

IR: V_{max} (neat/cm^{-1})

3058, 2930, 2879, 2854, 1731, 1725, 1370, 1153, 752.

m.p: 159–162 °C (EtOAc)

Spectroscopic data matched those previously reported[5].

Further Examples of RuAqua Catalysed C–H Methylation

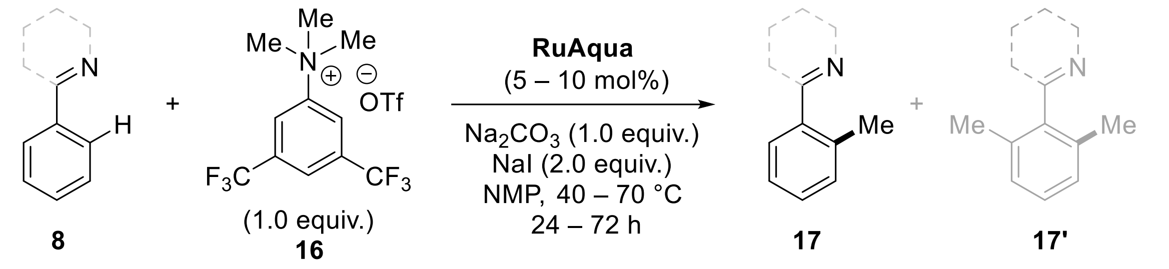

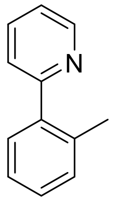

87% **17f**
5% **17f'**

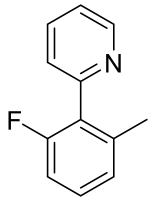

73% **17g**

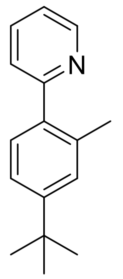

64% **17h**
7% **17h'**

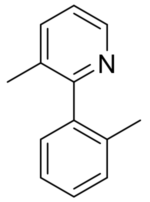

62% **17i**
5% **17i'**

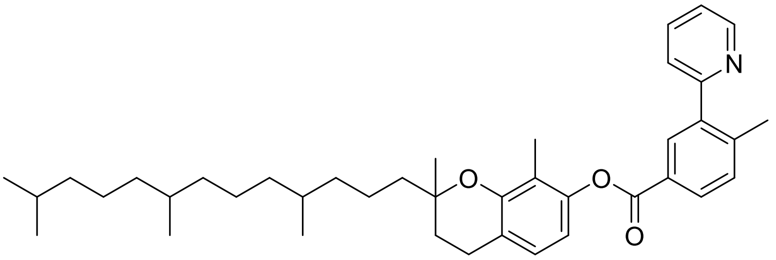

56% **17j**

Synthesis of 2-(*o*-tolyl)pyridine 17f and 2-(2,6-dimethylphenyl)pyridine 17f'

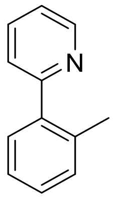

17f

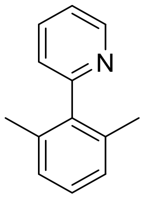

17f'

Reaction set up outside of the glovebox using Schlenk technique: The title compound was synthesized as outlined in general procedure E, using 2-phenylpyridine **8n** (47 mg, 0.3 mmol, 1.0 equiv.) and **RuAqua** (10.6 mg, 15 μmol) for 24 hours. Quantitative ^{1}H NMR using nitromethane as an internal standard indicated conversion of 91% to product **17f**. Purification using flash column chromatography eluting with 5–15% EtOAc in hexane (50 g silica gel cartridge, gradient over 15 CVs, 40 mL / min flowrate) afforded the product **17f** as a pale-yellow oil (36 mg, 87 %) and by-product **17f'** as a dark yellow oil (3 mg, 5%).

2-(*o*-tolyl)pyridine 17f

^{1}H NMR: (400 MHz, $CDCl_3$)

8.67 (m, 1H), 7.71 (td, *J* = 7.7, 1.9 Hz, 1H), 7.40 – 7.33 (m, 2H), 7.29 – 7.18 (m, 4H), 2.34 (s, 3H).

^{13}C NMR: (101 MHz, $CDCl_3$)

160.4, 149.5, 140.8, 137.0, 136.1, 131.0, 129.9, 128.6, 126.2, 124.4, 121.9, 20.6.

IR: V_{max} (neat/cm^{-1})

3058, 3016, 2956, 2924, 1606, 1468, 1423, 1024, 746.

2-(2,6-dimethylphenyl)pyridine 17f'

^{1}H NMR: (400 MHz, $CDCl_3$)

8.72 (m, 1H), 7.76 (app. td, *J* = 7.7, 1.9 Hz, 1H), 7.31 – 7.16 (m, 3H), 7.10 (d, *J* = 7.9 Hz, 2H), 2.04 (s, 6H).

^{13}C NMR: (101 MHz, $CDCl_3$)

160.3, 150.1, 140.8, 136.6, 136.1, 128.2, 127.9, 124.8, 122.0, 20.5.

Spectroscopic data matched those previously reported[5].

Synthesis of 2-(2-fluoro-6-methylphenyl)pyridine 17g

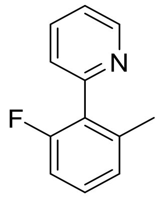

17g

Reaction set up outside of the glovebox using Schlenk technique: The title compound was synthesized as outlined in general procedure E, using 2-(2-fluorophenyl)pyridine **8j** (52 mg, 0.3 mmol, 1.0 equiv.) and **RuAqua** (10.6 mg, 0.015 mmol, 5 mol%). The reaction was allowed to stir for 24 hours. After this time, quantitative ^{1}H NMR using nitromethane as internal standard indicated conversion to product **17g** of 88%. Purification using flash column chromatography eluting with 0–10% EtOAc in hexane (50 g silica gel cartridge, gradient over 15 CVs, 40 mL / min flowrate) afforded the product **17g** as a yellow oil (34 mg, 73 %).

^{1}H NMR: (400 MHz, $CDCl_3$)

8.73 (m, 1H), 7.76 (td, *J* = 7.7, 1.8 Hz, 1H), 7.40 – 7.32 (m, 1H), 7.32 – 7.19 (m, 2H), 7.07 (d, *J* = 7.6 Hz, 1H), 6.98 (t, *J* = 8.8 Hz, 1H), 2.21 (s, 3H).

^{13}C NMR: (101 MHz, $CDCl_3$)

160.2 (d, *J* = 245.0 Hz), 154.4, 149.6, 139.0 (d, *J* = 2.4 Hz), 136.2, 129.4 (d, *J* = 8.8 Hz), 128.4 (d, *J* = 15.2 Hz), 125.9 (d, *J* = 3.4 Hz), 125.5 (d, *J* = 2.4 Hz), 122.3, 113.0 (d, *J* = 22.5 Hz), 19.7 (d, *J* = 2.4 Hz).

^{19}F NMR: (376 MHz, $CDCl_3$)

–117.2.

IR: V_{max} (neat/cm^{-1})

3049, 2926, 1615, 1483, 1460, 1252, 777, 747, 731.

Spectroscopic data matched those previously reported[5].

Synthesis of 2-(4-(*tert*-butyl)-2-methylphenyl)pyridine 17h and 2-(4-(*tert*-butyl)-2,6-dimethylphenyl)pyridine 17h'

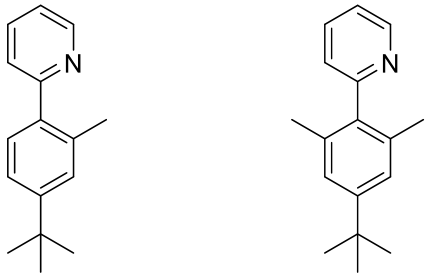

17h **17h'**

Reaction set up outside of the glovebox using Schlenk technique: The title compound was synthesized as outlined in general procedure E, using 2-(4-(*tert*-butyl)phenyl)pyridine **8s** (63 mg, 0.3 mmol, 1.0 equiv.) and **RuAqua** (10.6 mg, 15 μmol, 5 mol%). The reaction was allowed to stir for 24 hours. After this time, quantitative ^{1}H NMR using nitromethane as internal standard indicated conversion to product **17h** of 72%. Purification using flash column chromatography eluting with 0–10% EtOAc in hexane (50 g silica gel cartridge, gradient over 15 CVs, 40 mL / min flowrate) afforded the product **17h** as a yellow oil (43 mg, 64 %) and by-product **17h'** as a yellow oil (5 mg, 7% yield).

2-(4-(*tert*-butyl)-2-methylphenyl)pyridine 17h

^{1}H NMR: (400 MHz, $CDCl_3$)

8.59 – 8.51 (m, 1H), 7.59 (td, *J* = 7.7, 1.8 Hz, 1H), 7.27 (m, 1H), 7.22 (d, *J* = 8.7 Hz, 1H), 7.17 (d, *J* = 2.8 Hz, 2H), 7.08 (m, 1H), 2.25 (s, 3H), 1.22 (s, 9H).

^{13}C NMR: (101 MHz, $CDCl_3$)

160.1, 151.2, 149.2, 137.7, 136.0, 135.2, 129.4, 127.8, 124.0, 122.9, 121.4, 34.5, 31.4, 20.6.

IR: V_{max} (neat/cm^{-1})

3048, 2960, 2866, 1610, 1465, 1426, 787, 746.

2-(4-(*tert*-butyl)-2,6-dimethylphenyl)pyridine 17h'

^{1}H NMR: (400 MHz, $CDCl_3$)

8.70 (m, 1H), 7.74 (td, *J* = 7.7, 1.8 Hz, 1H), 7.26 – 7.20 (m, 2H), 7.14 – 7.08 (m, 2H), 2.05 (s, 6H), 1.33 (s, 9H).

^{13}C NMR: (101 MHz, $CDCl_3$)

160.2, 150.6, 149.7, 137.7, 136.1, 135.3, 124.6, 121.5, 34.4, 31.4, 20.5.

IR: V_{max} (neat/cm^{-1})

3047, 2960, 2924, 2866, 1589, 1464, 792, 748.

Spectroscopic data matched those previously reported[5].

Synthesis of 3-methyl-2-(*o*-tolyl)pyridine 17i

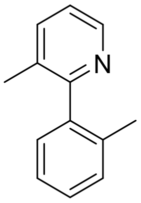

17i

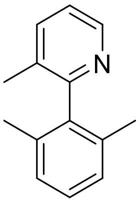

17i'

Reaction set up outside of the glovebox using Schlenk technique: The compound was synthesized as outlined in general procedure E, using 3-methyl-2-phenylpyridine **8r** (51 mg, 0.3 mmol, 1.0 equiv.) and **RuAqua** (10.6 mg, 15 μmol, 5 mol%) for 24 hours. After this time, quantitative ^{1}H NMR using nitromethane as internal standard indicated conversion to product **17i** of 80%. Column chromatography eluting with 0–10% EtOAc in hexane (50 g silica gel cartridge, gradient over 15 CVs, 40 mL / min flowrate) gave the product **17i** as a pale-yellow oil (34 mg, 62%) with small amounts of bis product (**17i'**, 5%).

^{1}H NMR: (400 MHz, $CDCl_3$)

8.51 (dd, *J* = 4.8, 1.8 Hz, 1H), 7.61 – 7.55 (m, 1H), 7.31 – 7.22 (m, 3H), 7.21 – 7.13 (m, 2H), 2.11 (s, 3H), 2.08 (s, 3H).

^{13}C NMR: (101 MHz, $CDCl_3$)

159.8, 146.9, 140.4, 137.8, 135.6, 131.6, 130.3, 128.6, 128.0, 125.8, 122.3, 19.5, 19.1.

IR: V_{max} (neat/cm^{-1})

3048, 2954, 1492, 1443, 754, 728.

Spectroscopic data matched those previously reported[5].

Synthesis of 2,8-dimethyl-2-(4,8,12-trimethyltridecyl)chroman-7-yl-4-methyl-3-(pyridin-2-yl)benzoate 17j from δ-tocopherol derivative

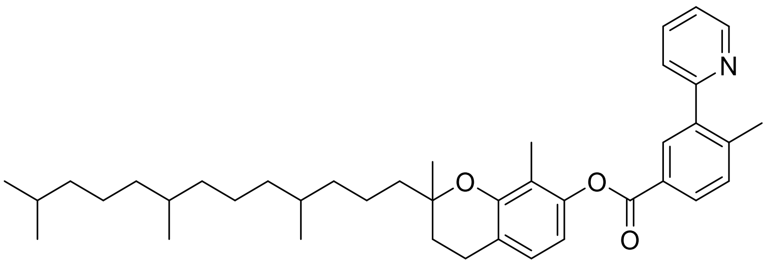

17j

Reaction set up outside of the glovebox using Schlenk technique: The title compound was synthesized as outlined in general procedure E, using the δ-tocopherol-derivative **8t** (175 mg, 0.3 mmol, 1.0 equiv.) and **RuAqua** (10.6 mg, 0.015 mmol, 5 mol%). The reaction was allowed to stir for 24 hours. After this time, quantitative ^{1}H NMR using nitromethane as internal standard indicated conversion to product **17j** of 78%. Purification using flash column chromatography eluting with 0–10% EtOAc in hexane (50 g silica gel cartridge, gradient over 15 CVs, 40 mL / min flowrate) afforded the product **17j** as a colourless oil (91 mg, 56%).

^{1}H NMR: (400 MHz, $CDCl_3$)

8.75 – 8.71 (m, 1H), 8.22 (d, *J* = 1.9 Hz, 1H), 8.11 (dd, *J* = 7.9, 1.9 Hz, 1H), 7.78 (td, *J* = 7.7, 1.8 Hz, 1H), 7.45 (dt, *J* = 7.8, 1.1 Hz, 1H), 7.41 (d, *J* = 8.0 Hz, 1H), 7.28 (ddd, *J* = 7.5, 4.9, 1.1 Hz, 1H), 6.81 (dd, *J* = 2.8, 0.9 Hz, 1H), 6.75 (d, *J* = 2.8 Hz, 1H), 2.82 – 2.69 (m, 2H), 2.46 (s, 3H), 2.18 (s, 3H), 1.89 – 1.69 (m, 2H), 1.67 – 0.99 (m, 24H), 0.87 (m, 12H).

^{13}C NMR: (101 MHz, $CDCl_3$)

165.8, 159.1, 149.9, 149.4, 142.8, 142.2, 140.8, 136.5, 131.4, 131.2, 129.9, 127.8, 127.4, 124.2, 122.2, 121.4, 121.1, 119.3, 76.2, 40.2, 39.5, 37.6, 37.5, 37.4, 32.9, 32.8, 31.1, 28.1, 24.9, 24.6, 24.3, 22.8, 22.7, 22.6, 21.1, 20.7, 19.9, 19.8, 16.3.

IR: V_{max} (neat/cm^{-1})

2924, 2865, 1733, 1473, 1215, 1079, 751.

Spectroscopic data matched those previously reported[5].

Meta-Selective C(sp^2)–H Functionalisation Reactions

Attainment of Starting Materials

Directing group arenes:

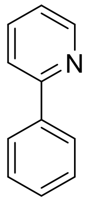

8n

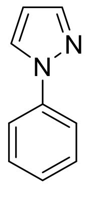

8h

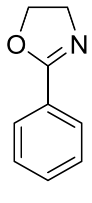

8o

Compounds **8n**, **8h** and **8o** were commercially available.

8n was purified prior to use via column chromatography eluting with 0–10% EtOAc in hexane.

Electrophiles:

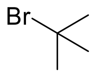

18a

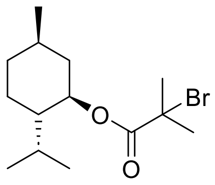

18b

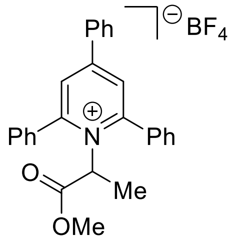

20

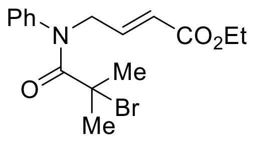

21

18a is commercially available.

18b[18], **20**[19] and **21**[20] were made according to previously established protocols in the literature.

Preparation of Starting Material 18b

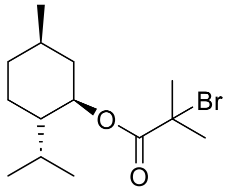

18b

Compound **18b** was prepared to a previously reported procedure with data in accordance.[18]

^{1}H NMR: (400 MHz, $CDCl_3$)

4.68 (td, *J* = 10.9, 4.4 Hz, 1H), 2.10 – 1.94 (m, 2H), 1.91 (s, 6H), 1.68 (dq, *J* = 13.8, 3.1 Hz, 2H), 1.55 – 1.41 (m, 2H), 1.13 – 0.95 (m, 2H), 0.90 (dd, *J* = 6.8, 4.4 Hz, 7H), 0.75 (d, *J* = 7.0 Hz, 3H).

^{13}C NMR: (101 MHz, $CDCl_3$)

171.3, 76.1, 56.4, 47.1, 40.2, 34.3, 31.5, 30.9, 30.8, 26.2, 23.4, 22.1, 20.9, 16.2.

Preparation of Starting Material 20

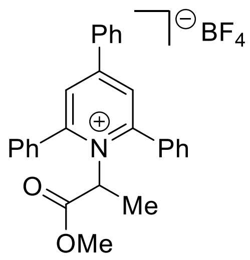

20

Compound **20** was prepared to a previously reported procedure with data in accordance.[19]

^{1}H NMR: (500 MHz, $CDCl_3$)

7.92 (s, 2H), 7.85 – 7.65 (m, 5H), 7.62 – 7.48 (m, 10H), 5.55 (q, *J* = 7.2 Hz, 1H), 3.67 (s, 3H), 1.51 (s, 3H).

^{13}C NMR: (101 MHz, $CDCl_3$)

169.3, 157.1, 156.8, 134.1, 132.9, 132.4, 131.5, 129.8, 129.2 (x2), 128.6, 128.0, 64.8, 53.8, 17.3.

^{19}F NMR: (376 MHz, $CDCl_3$)

–153.2.

Preparation of Starting Material 21

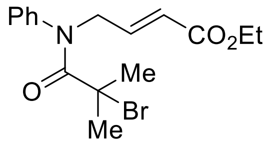

21

Compound **21** was prepared to a previously reported procedure with data in accordance.[20]

^{1}H NMR: (400 MHz, $CDCl_3$)

7.45 – 7.31 (m, 5H), 6.94 (dt, *J* = 15.7, 5.9 Hz, 1H), 5.96 – 5.84 (m, 1H), 4.40 (dd, *J* = 5.9, 1.6 Hz, 2H), 4.22 – 4.11 (m, 2H), 1.70 (s, 6H), 1.27 (t, *J* = 7.1 Hz, 3H).

^{13}C NMR: (101 MHz, $CDCl_3$)

170.0, 165.9, 142.4, 141.9, 129.6, 129.3, 128.7, 123.5, 60.5, 57.8, 54.7, 33.3, 14.3.

General Procedure F: *Meta*-Functionalisation with RuAqua

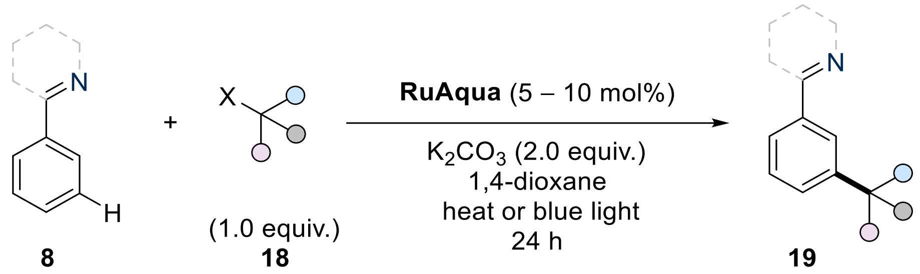

Reaction set up outside of the glovebox: Solid reagents were weighed into a microwave vial equipped with a magnetic stirrer bar before capping the reaction vessel and performing 3 x 5 min evac-refill cycles with N_2. Liquid reagents and solvents were then added before purging the vessel with N_2 for 30 seconds. Products were isolated *via* column chromatography on a manual column (silica 230–400 mesh) using the indicated conditions.

Synthesis of 2-(3-(*tert*-butyl)phenyl)pyridine 19a

a) *Light promoted procedure:*

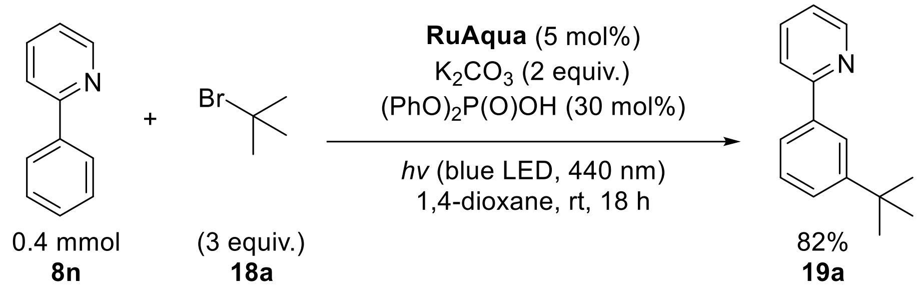

Reaction set up outside of the glovebox: Reaction conditions adapted from previously reported procedure[20]. The reaction was performed according to general procedure F using 2-phenylpyridine **8n** (62 mg, 0.4 mmol) ^{t}BuBr **18a** (135 μL, 1.2 mmol, 3 equiv.), **RuAqua** (14.2 mg, 0.02 mmol, 5 mol%), diphenyl phosphonate (30 mg, 0.12 mmol, 30 mol%), K_2CO_3 (111 mg, 0.8 mmol, 2 equiv.) and 1,4-dioxane (2.0 mL, 0.2 M with respect to **8n**). After stirring under blue light irradiation (Kessil lamp, 440 nm) for 18 h, the reaction mixture was purified via column chromatography eluting with 5–15% EtOAc in hexane giving the product **19a** as a yellow oil (69 mg, 82%).

b) *Heat promoted procedure:*

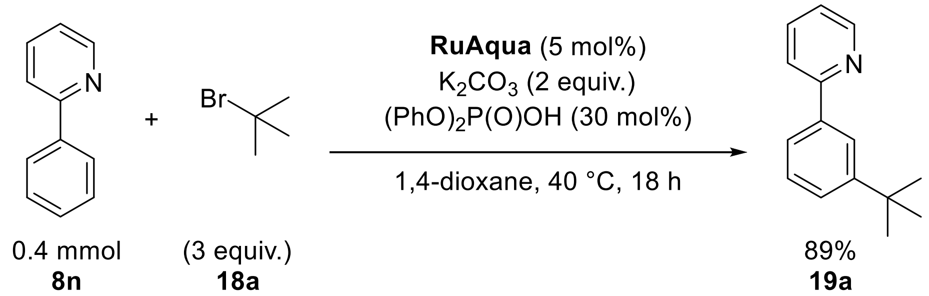

Reaction set up outside of the glovebox: Reaction conditions adapted from previously reported procedure using 40°C instead of light[20]. The reaction was performed according to general procedure F using 2-phenylpyridine **8n** (62 mg, 0.4 mmol) ^{t}BuBr **18a** (135 μL, 1.2 mmol, 3 equiv.), **RuAqua** (14 mg, 0.02 mmol, 5 mol%), diphenyl phosphonate (30 mg, 0.12 mmol, 30 mol%), K_2CO_3 (111 mg, 0.8 mmol, 2 equiv.) and 1,4-dioxane (2.0 mL, 0.2 M with respect to **8n**). After stirring for 18 h, the reaction mixture was purified via column chromatography eluting with 5–15% EtOAc in hexane giving the product **19a** as a yellow oil (75 mg, 89%).

^{1}H NMR: (500 MHz, $CDCl_3$)

8.71 (dt, *J* = 5.0, 1.4 Hz, 1H), 8.05 (t, *J* = 1.9 Hz, 1H), 7.85 – 7.64 (m, 3H), 7.48 – 7.47 (m, 1H), 7.42 (t, *J* = 7.7 Hz, 1H), 7.23 (ddd, *J* = 6.7, 4.8, 1.9 Hz, 1H), 1.40 (s, 9H).

^{13}C NMR: (126 MHz, $CDCl_3$)

158.2, 151.8, 149.7, 139.3, 136.9, 128.6, 126.2, 124.3, 124.1, 122.1, 120.9, 35.0, 31.5.

Spectroscopic data matched those previously reported[20].

Synthesis of (1*S*,2*R*,5*S*)-2-isopropyl-5-methylcyclohexyl 2-methyl-2-(3-(pyridin-2-yl)phenyl)propanoate 19b

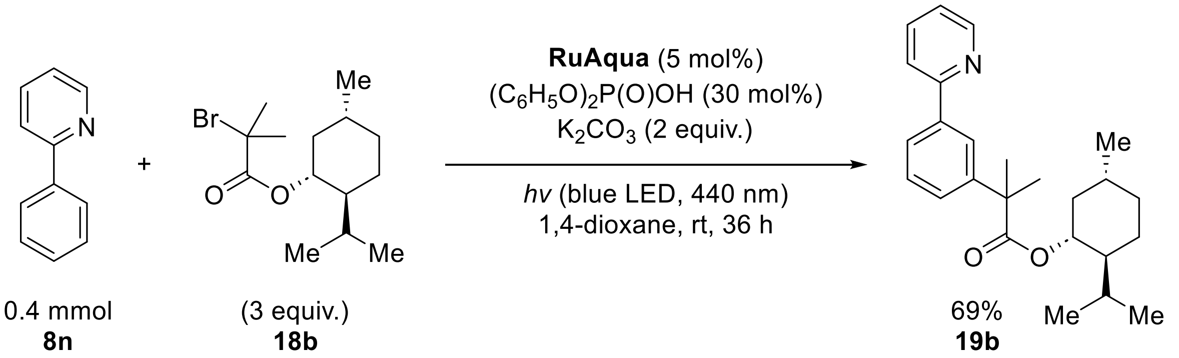

Reaction set up outside of the glovebox: Reaction conditions adapted from previously reported procedure[20]. The reaction was performed according to general procedure F using 2-phenylpyridine **8n** (62 mg, 0.4 mmol) electrophile **18b** (366 mg, 1.2 mmol, 3 equiv.), **RuAqua** (14.2 mg, 0.02 mmol, 5 mol%), diphenyl phosphonate (30 mg, 0.12 mmol, 30 mol%), K_2CO_3 (111 mg, 0.8 mmol, 2 equiv.) and 1,4-dioxane (2.0 mL, 0.2 M with respect to **8n**) which were reacted under blue light irradiation (Kessil, 440 nm) for 36 h. The reaction mixture was then purified via column chromatography eluting with EtOAc in hexane (5–15%) to give the product **19b** as a pale-yellow oil (105 mg, 69%).

^{1}H NMR: (400 MHz, $CDCl_3$)

8.69 (ddd, *J* = 4.8, 1.8, 1.0 Hz, 1H), 8.06 – 7.93 (m, 1H), 7.84 (dt, *J* = 7.3, 1.6 Hz, 1H), 7.78 – 7.66 (m, 2H), 7.49 – 7.34 (m, 2H), 7.21 (ddd, *J* = 6.7, 4.8, 1.7 Hz, 1H), 4.64 (td, *J* = 10.9, 4.4 Hz, 1H), 2.10 – 1.88 (m, 1H), 1.63 (s, 6H), 1.60 – 1.55 (m, 3H), 1.50 – 1.37 (m, 1H), 1.36 – 1.16 (m, 1H), 1.10 – 0.93 (m, 1H), 0.91 – 0.81 (m, 5H), 0.69 (d, *J* = 7.0 Hz, 3H), 0.59 (d, *J* = 7.0 Hz, 3H).

^{13}C NMR: (101 MHz, $CDCl_3$)

176.4, 157.6, 149.7, 145.5, 139.5, 136.8, 128.7, 126.5, 125.3, 124.5, 122.2, 120.7, 74.7, 46.9 (2 x resonances), 40.5, 34.3, 31.4, 26.7, 26.5, 25.8, 23.2, 22.1, 20.9, 15.9.

IR: V_{max} (thin film/cm^2)

2927, 1718, 1584, 1432, 1386, 1252, 1143, 772.

Spectroscopic data matched those previously reported[20].

Synthesis of 1-(3-(*tert*-butyl)phenyl)-1H-pyrazole 19c

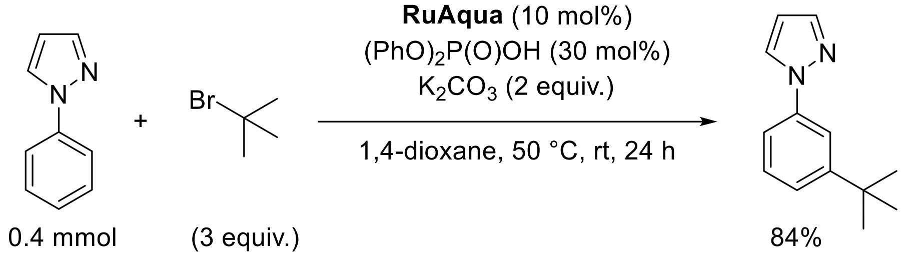

0.4 mmol **8h** (3 equiv.) **18a** 84% **19c**

a) Reaction set up outside of the glovebox: Reaction conditions adapted from previously reported procedure using 50 °C instead of light[20]. The reaction was carried out according to general procedure F using 1-phenylpyrazole **8h** (58 mg, 0.4 mmol), ^{t}BuBr **18a** (135 μL, 1.2 mmol, 3 equiv.), **RuAqua** (28.4 mg, 0.04 mmol, 10 mol%), diphenyl phosphonate (30 mg, 0.12 mmol, 30 mol%), K_2CO_3 (111 mg, 0.8 mmol, 2 equiv.) and 1,4-dioxane (2 mL, 0.2 M with respect to **8h**) allowing the reaction mixture to stir at 50 °C for 24 h. The crude reaction mixture was then purified *via* column chromatography eluting with EtOAc in hexane (0–15%) to give the title compound as a yellow oil (68 mg, 84%).
b) Reaction set up outside of the glovebox: The reaction was carried out according to general procedure F using 1-phenylpyrazole **8h** (58 mg, 0.4 mmol), ^{t}BuBr **18a** (135 μL, 1.2 mmol, 3 equiv.), $[Ru(p\text{-cymene})Cl_2]_2$ (12.2 mg, 0.04 mmol, 10 mol%), diphenyl phosphonate (30 mg, 0.12 mmol, 30 mol%), K_2CO_3 (111 mg, 0.8 mmol, 2 equiv.) and 1,4-dioxane (2 mL, 0.2 M with respect to **8h**) allowing the reaction mixture to stir at 50 °C for 24 h. The desired product **19c** was not observed by NMR.
c) Reaction set up outside of the glovebox: The reaction was carried out according to general procedure F using 1-phenylpyrazole **8h** (58 mg, 0.4 mmol), ^{t}BuBr **18a** (135 μL, 1.2 mmol, 3 equiv.), $RuCl_3$ (8.2 mg, 0.04 mmol, 10 mol%), diphenyl phosphonate (30 mg, 0.12 mmol, 30 mol%), K_2CO_3 (111 mg, 0.8 mmol, 2 equiv.) and 1,4-dioxane (2 mL, 0.2 M with respect to **8h**) allowing the reaction mixture to stir at 50 °C for 24 h. The desired product **19c** was not observed by NMR.

^{1}H NMR: (400 MHz, $CDCl_3$)

7.92 (dd, J = 2.4, 0.7 Hz, 1H), 7.76 (t, J = 2.0 Hz, 1H), 7.74 (d, J = 1.8 Hz, 1H), 7.45 (ddd, J = 7.7, 2.2, 1.4 Hz, 1H), 7.38 (t, J = 7.7 Hz, 1H), 7.33 (dt, J = 7.8, 1.6 Hz, 1H), 6.46 (dd, J = 2.4, 1.8 Hz, 1H), 1.38 (s, 9H).

^{13}C NMR: (101 MHz, $CDCl_3$)

153.1, 141.0, 140.2, 129.1, 127.1, 123.8, 116.9, 116.6, 107.5, 35.1, 31.4.

Spectroscopic data matched those previously reported[20].

Synthesis of 2-(3-(*tert*-butyl)phenyl)-4,5-dihydrooxazole 19d

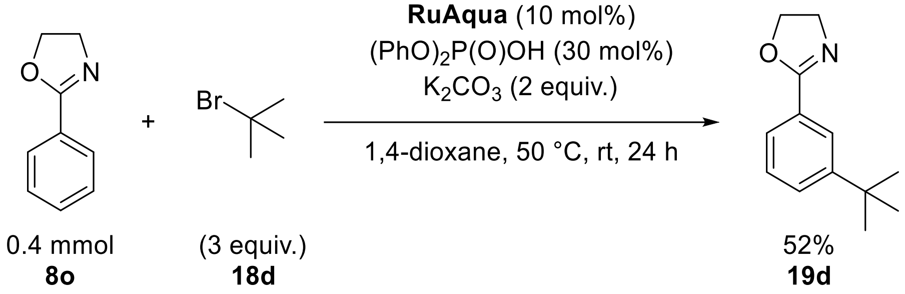

Reaction set up outside of the glovebox: Reaction conditions adapted from previously reported procedure using 50 °C instead of light[20]. The reaction was performed according to general procedure F using 2-phenyl-4,5-dihydrooxazole **8o** (59 mg, 0.4 mmol), ^{t}BuBr **18a** (135 μL, 1.2 mmol, 3 equiv.), **RuAqua** (28.4 mg, 0.04 mmol, 10 mol%), diphenyl phosphonate (30 mg, 0.12 mmol, 30 mol%), K_2CO_3 (111, 0.8 mmol, 2 equiv.) and 1,4-dioxane (2 mL, 0.2 M with respect to **8o**) allowing the reaction mixture to stir at 50 °C for 24 h. After this time the crude reaction mixture was purified via column chromatography eluting with EtOAc in hexane (5–15%) giving the product **19d** as a brown oil (42 mg, 52%).

^{1}H NMR: (400 MHz, $CDCl_3$)

8.00 (t, *J* = 1.9 Hz, 1H), 7.75 (dt, *J* = 7.6, 1.4 Hz, 1H), 7.51 (ddd, *J* = 7.9, 2.1, 1.2 Hz, 1H), 7.34 (t, *J* = 7.8 Hz, 1H), 4.43 (t, *J* = 9.3 Hz, 2H), 4.06 (t, *J* = 9.4 Hz, 2H), 1.34 (s, 9H).

^{13}C NMR: (101 MHz, $CDCl_3$)

165.2, 151.5, 128.6, 128.2, 127.4, 125.5, 125.3, 67.7, 54.9, 34.9, 31.4.

IR: V_{max} (thin film/cm^2)

2962, 1649, 1480, 1363, 1243, 1067, 949, 711.

Spectroscopic data matched those previously reported[20].

Synthesis of methyl 2-(3-(pyridin-2-yl)phenyl)propanoate 19e

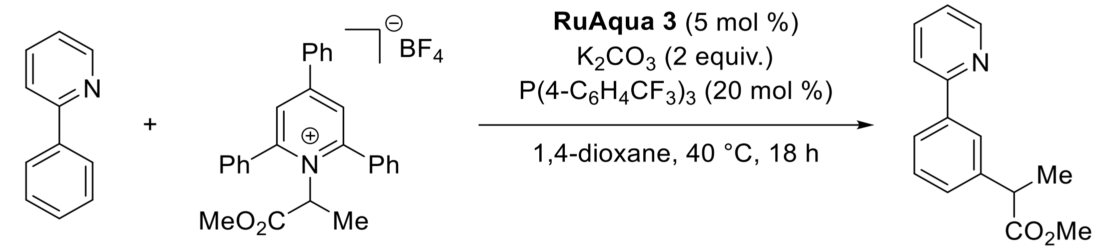

0.2 mmol **8n** (2 equiv.) **20** 56% **19e**

Reaction set up outside of the glovebox: Reaction conditions adapted from previously reported procedure reducing the temperature from 100 °C to 40 °C[21]. The reaction was carried out according to general procedure F using 2-phenylpyridine **8n** (31 mg, 0.2 mmol), Katritzky salt **20** (193 mg, 0.4 mmol, 2 equiv.), **RuAqua** (7.1 mg, 0.01 mmol, 5 mol%), K_2CO_3 (55 mg, 0.4 mmol, 2 equiv.), P(4-$C_6H_4CF_3)_3$ (19 mg, 0.04 mmol, 20 mol%) and 1,4-dioxane (2 mL, 0.1 M with respect to **8n**) allowing the resulting mixture to stir at 40 °C for 18 h. After this time the crude reaction mixture was purified via column chromatography eluting with EtOAc in hexane (5–15%) to give the title compound **19e** as a brown oil (28 mg, 56%).

^{1}H NMR: (400 MHz, $CDCl_3$)

8.70 (d, *J* = 4.8 Hz, 1H), 7.94 (s, 1H), 7.87 (d, *J* = 7.7 Hz, 1H), 7.81 – 7.67 (m, 2H), 7.44 (t, *J* = 7.6 Hz, 1H), 7.37 (d, *J* = 7.6 Hz, 1H), 7.23 (ddd, *J* = 6.7, 5.0, 1.8 Hz, 1H), 3.84 (t, *J* = 7.2 Hz, 1H), 3.67 (s, 3H), 1.56 (d, *J* = 7.2 Hz, 3H).

^{13}C NMR: (101 MHz, $CDCl_3$)

175.1, 157.4, 149.7, 141.2, 139.8, 136.9, 129.2, 128.1, 126.5, 125.9, 122.3, 120.8, 52.2, 45.6, 18.8.

IR: V_{max} (thin film/cm^2)

2979, 1729, 1583, 1375, 1163, 743.

Spectroscopic data matched those previously reported[21].

Synthesis of ethyl 2-(4,4-dimethyl-5-oxo-1-phenylpyrrolidin-3-yl)-2-(3-(pyridin-2-yl)phenyl)acetate 19f

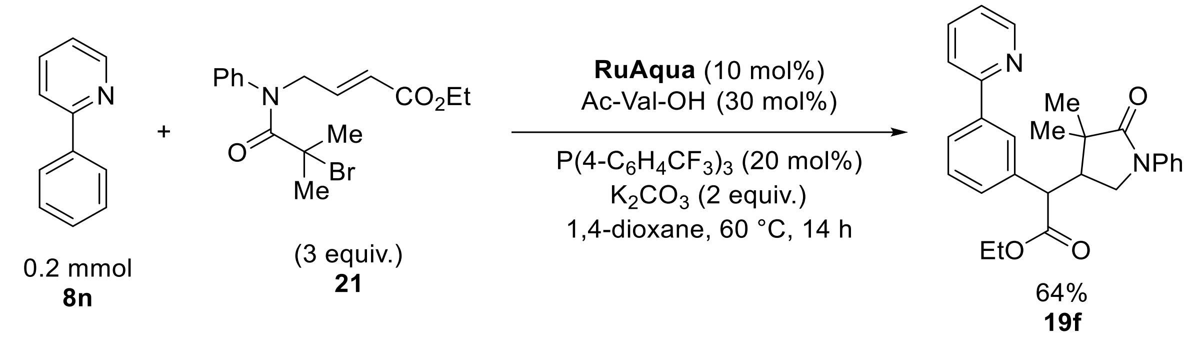

Reaction set up outside of the glovebox: Reaction conditions adapted from previously reported procedures reducing the temperature from 80 °C to 60 °C[22]. The reaction was performed according to general procedure F using 2-phenylpyridine **8n** (31 mg, 0.2 mmol), electrophile **21** (213 mg, 0.6 mmol, 3 equiv.), **RuAqua** (14.2 mg, 0.02 mmol, 10 mol%), Ac-Val-OH (10 mg, 0.06 mmol, 30 mol%), P(4-$C_6H_4CF_3)_3$ (19 mg, 0.04 mmol, 20 mol%) and 1,4-dioxane (2.0 mL, 0.1 M with respect to **8n**). After stirring at 60 °C for 14 h, the product was purified *via* column chromatography eluting with EtOAc in hexane (5–15%) giving the title compound **19f** as a colourless oil (54 mg, 64%).

^{1}H NMR: (400 MHz, $CDCl_3$)

8.71 (dd, *J* = 4.9, 1.4 Hz, 1H), 8.09 (d, *J* = 1.9 Hz, 1H), 7.94 (dt, *J* = 7.4, 1.6 Hz, 1H), 7.84 – 7.72 (m, 2H), 7.69 – 7.59 (m, 1H), 7.57 – 7.42 (m, 3H), 7.36 (ddd, *J* = 8.6, 7.4, 5.8 Hz, 1H), 7.31 – 7.22 (m, 2H), 7.19 – 7.01 (m, 1H), 4.35 – 4.15 (m, 1H), 4.14 – 4.05 (m, 1H), 4.00 – 3.58 (m, 2H), 3.29 – 3.22 (m, 1H), 3.14 (ddd, *J* = 11.6, 10.2, 7.6 Hz, 1H), 1.35 (s, 2H), 1.29 – 1.22 (m, 5H), 1.12 (s, 1H), 0.74 (s, 1H).

^{13}C NMR: (101 MHz, $CDCl_3$)

178.3, 172.7, 156.8, 149.8, 139.4, 137.1, 129.6, 129.3, 128.9, 128.8, 127.6, 127.3, 126.8, 124.6, 122.6, 120.9, 119.7, 61.4, 51.8, 49.2, 45.7, 44.7, 24.8, 19.0, 14.2.

IR: V_{max} (thin film/cm^2)

2937, 1729, 1698, 1461, 1398, 1270.

Spectroscopic data matched those previously reported[22].

RuAqua Catalysed H / D Exchange

Attainment of Starting Materials

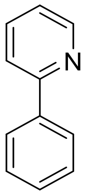

8n

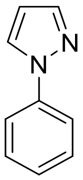

8h

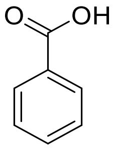

22

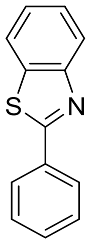

8p

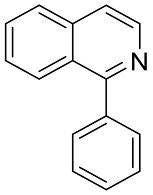

8g

Compounds **8n**, **8h**, **22**, **8p** and **8g** were commercially available. **8n** was purified via column chromatography eluting with 0–10% EtOAc in hexane prior to use.

General Procedure G: RuAqua catalysed H / D exchange

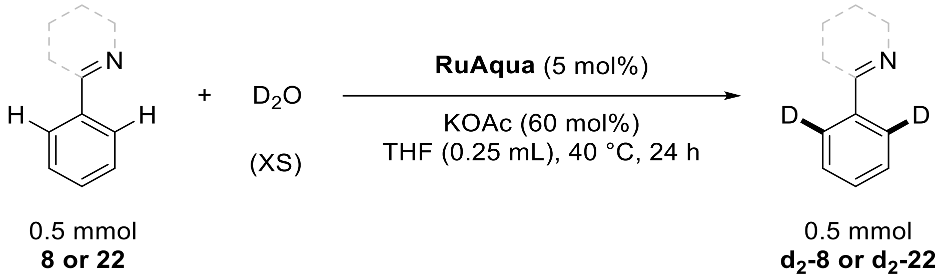

Reaction set up outside of the glovebox: Reactions were carried out without any optimisation performed. To a microwave vial equipped with a magnetic stirrer bar was added **RuAqua** (17.7 mg, 0.03 mmol, 5 mol%) and KOAc (29 mg, 0.3 mmol, 60 mol%) before purging with N_2 for 10 minutes. Solid substrates **8** (0.5 mmol, 1 equiv.) were also added before degassing took place while oil/liquid reagents were degassed with N_2 for 10 minutes and added *via* microsyringe. N_2-degassed D_2O (0.25 mL) and THF (0.25 mL) were then added and the resulting mixture was left to stir at 40 °C for 24 h. After this time the reaction was extracted with Et_2O (3 x 3 mL) and filtered through a small plug of silica to give the desired product.

Synthesis of 2-(Phenyl-2,6-d_2)pyridine-6-d (d_2-8n)

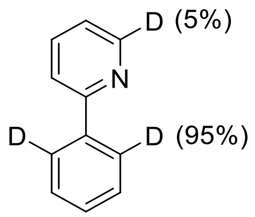

d_2-8n

Reaction set up outside of the glovebox: General procedure G was applied using 2-phenylpyridine **8n** (71 µL, 0.5 mmol) as a substrate. The organic phase was extracted using Et_2O (3 x 2 mL), dried over Na_2SO_4, filtered and the solvent removed *in vacuo*. The crude mixture was filtered (SiO_2, 5 g, diethylether) to give **d_2-8n** (74 mg, 95%) as a colourless oil.

^{1}H NMR: (500 MHz, $CDCl_3$)

8.80 – 8.66 (m, **0.95H**), 8.01 – 7.98 (m, **0.1H**), 7.84 – 7.68 (m, 2H), 7.52 (d, J = 6.8 Hz, 2H), 7.49 – 7.42 (m, 1H), 7.27 (ddd, J = 6.6, 4.8, 1.8 Hz, 1H).

^{2}D NMR: (77 MHz, $CHCl_3$)

8.05 (s, 2D).

^{13}C NMR: (126 MHz, $CDCl_3$)

157.6, 149.8, 139.4, 136.9, 129.1 128.9, 128.8, 127.0, 126.7 (t, J = 24.0 Hz), 122.2, 120.7.

Spectroscopic data matched those previously reported[23].

Synthesis of 1-(phenyl-2,6-d$_2$)-1H-pyrazole (d$_2$-8h)

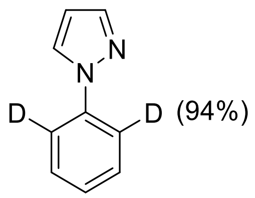

d$_2$-8h

a) Reaction set up outside of the glovebox: General procedure G was applied using 1-phenylpyrazole **8h** (72 mg, 0.5 mmol, 1 equiv.) as a substrate. The organic phase was extracted using Et_2O (3 x 2 mL), dried over Na_2SO_4, filtered and the solvent removed *in vacuo*. The crude mixture was filtered (SiO_2, 5 g, diethylether) to give **d$_2$-8h** (74 mg, 94%) as pale purple oil.
b) Reaction set up outside of the glovebox: General procedure G was applied using 1-phenylpyrazole **8h** (72 mg, 0.5 mmol, 1 equiv.) as a substrate with $RuCl_3$ (5.2 mg, 25 μmol, 5 mol%) in place of **RuAqua**. The organic phase was extracted using Et_2O (3 x 2 mL), dried over Na_2SO_4, filtered and the solvent removed *in vacuo*. Quantitative ^{1}H NMR using 1,3,5-trimethoxybenzene as an internal standard indicated 2% incorporation of deuterium.
c) Reaction set up outside of the glovebox: General procedure G was applied using 1-phenylpyrazole (72 mg, 0.5 mmol, 1 equiv.) as a substrate with [Ru(*p*-cymene)Cl_2]$_2$ (7.7 mg, 25 μmol, 5 mol% with respect to Ru) in place of **RuAqua**. The organic phase was extracted using Et_2O (3 x 2 mL), dried over Na_2SO_4, filtered and the solvent removed *in vacuo*. Quantitative ^{1}H NMR using 1,3,5-trimethoxybenzene as an internal standard indicated 5% incorporation of deuterium.

^{1}H NMR: (400 MHz, $CDCl_3$)

7.93 (d, *J* = 2.5 Hz, **0.97H**), 7.76 – 7.67 (m, **1.12H**), 7.49 – 7.40 (m, 2H), 7.29 (t, *J* = 7.4 Hz, 1H), 6.47 (t, *J* = 2.1 Hz, 1H).

^{2}D NMR: (77 MHz, $CHCl_3$)

7.75 (s, 2D).

^{13}C NMR: (126 MHz, $CDCl_3$)

160.9, 142.4, 139.6, 137.0, 130.1, 130.0, 129.8 (t, *J* = 24.5 Hz), 128.7, 128.5, 128.4, 127.7, 127.3, 127.1, 126.9, 120.0.

Spectroscopic data matched those previously reported[23].

Synthesis of benzoic-2,6-d_2 acid-d (d_2-22)

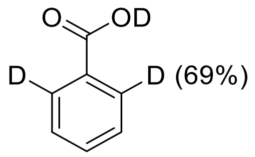

d_2-22

Reaction set up outside of the glovebox: General procedure G was applied using benzoic acid **22** (61 mg, 0.5 mmol, 1 equiv.) as a substrate. The organic phase was extracted using Et_2O (3 x 2 mL), dried over Na_2SO_4, filtered and the solvent removed *in vacuo*. The crude mixture was filtered (SiO_2, 5 g, diethylether) to give the product **d_2-22** (55 mg, 69%) as an off-white amorphous solid.

^{1}H NMR: (400 MHz, $CDCl_3$)

8.11 (dd, *J* = 8.0, 1.3 Hz, **0.62H**), 7.61 (td, *J* = 7.5, 1.0 Hz, 1H), 7.52 – 7.40 (m, 2H).

^{2}D NMR: (77 MHz, $CHCl_3$)

8.18 (s, 2D).

^{13}C NMR: (101 MHz, $CDCl_3$)

172.6, 134.0, 130.3 (2 x resonances), 130.3 – 129.8 (t, *J* = 25.1 Hz), 129.5, 129.4, 129.3, 128.3, 128.5.

Spectroscopic data matched those previously reported[23].

Synthesis of 2-(phenyl-2,6-d2)benzo[d]thiazole (d_2-8p)

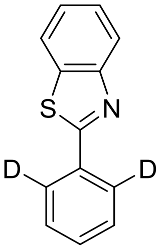

D (42%)

d_2-8p

Reaction set up outside of the glovebox: General procedure G was applied using 2-phenylbenzo[d]thiazole **8p** (106 mg, 0.5 mmol) as a substrate. The organic phase was extracted using Et_2O (3 x 2 mL), dried over Na_2SO_4, filtered and the solvent removed *in vacuo*. The crude mixture was filtered (SiO_2, 5 g, diethylether) to give the product **d_2-8p** (105 mg, 42%) as white solid needles.

^{1}H NMR: (500 MHz, $CDCl_3$)

8.13 – 8.04 (m, **2.16H**), 7.91 (d, *J* = 8.0 Hz, 1H), 7.54 – 7.44 (m, 4H), 7.43 – 7.36 (m, 1H).

^{2}D NMR: (77 MHz, $CHCl_3$)

8.15 (s, 2D).

^{13}C NMR: (126 MHz, $CDCl_3$)

168.1, 154.2, 135.1, 133.7, 133.6, 133.5, 131.0, 129.1, 128.9, 127.6, 127.3 (t, *J* = 24.2 Hz), 126.3, 125.2, 123.2, 121.7.

Spectroscopic data matched those previously reported[24].

Synthesis of 1-(phenyl-2,6-d_2)isoquinoline-3-d (d_2-8g, Additional Example)

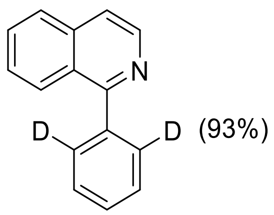

d_2-8g

Reaction set up outside of the glovebox: General procedure G was applied using 1-phenylisoquinoline **8g** (103 mg, 0.5 mmol, 1 equiv.) as a substrate. The organic phase was extracted using Et_2O (3 x 2 mL), dried over Na_2SO_4, filtered and the solvent removed *in vacuo*. The crude mixture was filtered (SiO_2, 5 g, diethylether) to give the product **d_2-8g** (101 mg, 93%) as a light brown amorphous solid.

^{1}H NMR: (400 MHz, $CDCl_3$)

8.62 (d, *J* = 5.7 Hz, **0.96H**), 8.12 (dq, *J* = 8.5, 1.0 Hz, 1H), 7.89 (dt, *J* = 8.2, 1.0 Hz, 1H), 7.74 – 7.62 (m, **2.18H**), 7.58 – 7.47 (m, 4H).

^{2}D NMR: (77 MHz, $CHCl_3$)

7.76 (s, 2D).

^{13}C NMR: (126 MHz, $CDCl_3$)

160.5, 142.0, 139.2, 136.7, 129.8, 129.7, 129.4 (t, *J* = 24.6 Hz), 128.4, 128.2, 128.0, 127.4, 127.0, 126.8, 126.5.

RuAqua Catalysed Alkene Isomerisation

Synthesis of (*E*)-2-methoxy-4-(prop-1-en-1-yl)phenol 24

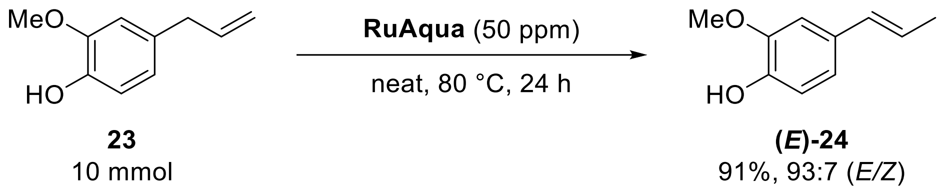

Reaction conditions adapted from previously reported procedure lowering the temperature from 150 °C to 80 °C[25].

a) Reaction set up outside of the glovebox: Eugenol was commercially available and used directly without further purification. A stock solution of **RuAqua** was prepared in acetone (17.1 mg in a 50 mL volumetric flask) and 1.25 mL was added to a microwave vial before removing acetone under reduced pressure. The microwave vial was purged with argon for 10 mins before the addition of eugenol **23** (1.5 mL, 10 mmol, 1 equiv.). The reaction was then stirred at 80 °C for 24 hours. The reaction was diluted in EtOAc and filtered through a small pad of silica gel giving product **(*E*)-24** as a yellow oil (1.50 g, 91% yield, 93:7 (*E/Z*)).
b) Reaction set up outside of the glovebox: Reaction was carried out as above using $RuCl_3$ (50 ppm, added as stock solution in MeOH before removal of MeOH under reduced pressure) in place of **RuAqua**. Quantitative ^{1}H NMR using nitromethane as an internal standard indicated no formation of product **(*E*)-24**.
c) Reaction set up outside of the glovebox: Reaction was carried out as above using [Ru(*p*-cymene)Cl_2]$_2$ (50 ppm with respect to Ru, added as stock solution in MeOH before removal of MeOH under reduced pressure) in place of **RuAqua**. Quantitative ^{1}H NMR using nitromethane as an internal standard indicated 76% conversion to the product **(*E*)-24**, 92:8 (*E*/*Z*).

^{1}H NMR: (500 MHz, $CDCl_3$)

6.89 – 6.79 (m, 3H), 6.32 (dq, *J* = 15.7, 1.8 Hz, 1H), 6.08 (dq, *J* = 15.7, 6.6 Hz, 1H), 5.55 (s, 1H), 3.90 (s, 3H), 1.86 (dd, *J* = 6.6, 1.7 Hz, 3H).

^{13}C NMR: (126 MHz, $CDCl_3$)

146.7, 144.9, 130.9, 130.8, 123.6, 119.4, 114.5, 108.0, 56.0, 18.5.

IR: V_{max} (neat/cm^{-1})

3424 (br), 3015, 2960, 2934, 2915, 2730, 1676, 1596, 1509, 1261, 1030, 960, 854, 803.

Spectroscopic data matched those previously reported.[25]

RuAqua Catalysed Hydroalkynylation

Synthesis of (*E*)-But-1-en-3-yne-1,4-diyldibenzene 26

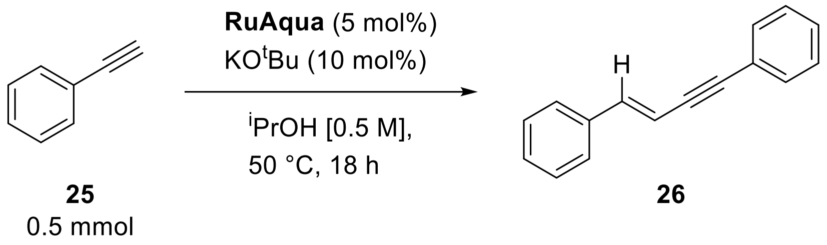

Phenylacetylene is commercially available and was used without further purification. Reaction was discovered while testing various possible reactions and no specific optimisation was performed.

a) Reaction set up outside of the glovebox: Phenylacetylene (55 µL, 0.5 mmol), potassium *tert*-butoxide (6 mg, 50 µmol, 10 mol%) and **RuAqua** (18 mg, 25 µmol, 5 mol%) were reacted in isopropanol (1.0 mL, 0.5 M with respect to **25**) in a microwave vial which had been sparged with N_2 for 10 minutes at 50 °C for 18 hours. The solvent was removed under reduced pressure and the crude reaction product purified by flash column chromatography eluting with EtOAc in hexane (0–10%) to give the product **26** (96:4 *E*/*Z*, 19 mg, 93 µmol, 37%) as a colourless amorphous solid.
b) Reaction set up outside of the glovebox: The reaction was carried out as indicated above using $RuCl_3$ (5.2 mg, 25 µmol, 5 mol%) in place of **RuAqua**. The desired product **26** was not observed by NMR.
c) Reaction set up outside of the glovebox: The reaction was carried out as indicated above using $[Ru(p\text{-cymene})Cl_2]_2$ (7.7 mg, 25 µmol, 5 mol%) in place of **RuAqua**. Quantitative ^{1}H NMR using 1,3,5-trimethoxybenzene as an internal standard indicated 9% conversion to the desired product **26**.

^{1}H NMR: (500 MHz, $CDCl_3$)

7.55 – 7.47 (m, 2H), 7.46 – 7.41 (m, 2H), 7.40 – 7.27 (m, 6H), 7.07 (d, *J* = 16.2 Hz, 1H), 6.41 (d, *J* = 16.2 Hz, 1H).

^{13}C NMR: (126 MHz, $CDCl_3$)

141.3, 136.4, 131.6 (2 x resonances), 128.8, 128.7, 128.4, 128.2, 126.4, 123.5, 91.8, 88.9.

m.p: 92–94°C (EtOAc)

Spectroscopic data matched those previously reported[26].

RuAqua Catalysed C(sp^3)–H Oxidation

Synthesis of Adamantan-1-ol 28a and adamantane-1,3-diol 28b

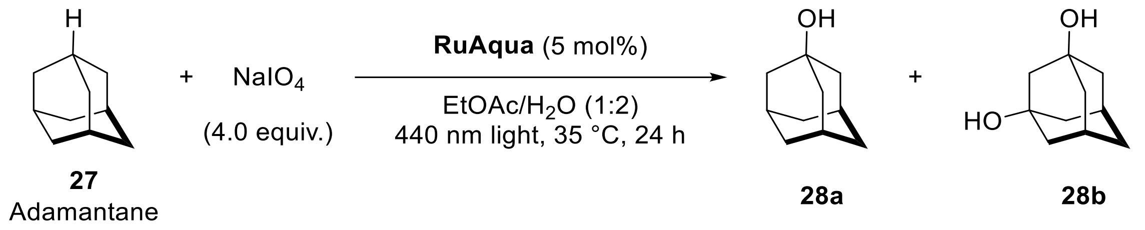

Adamantane is commercially available and was used without further purification. Reaction conditions were adapted from previously published procedures[27].

a) Reaction set up outside of the glovebox: Adamantane **27** (136 mg, 1.0 mmol), sodium periodate (856 mg, 4.0 mmol, 4.0 equiv.) and **RuAqua** (35.4 mg, 50 µmol, 5 mol%) were reacted under blue light irradiation (Kessil, 440 nm) in ethyl acetate/water (2:1, 3 mL) under an ambient atmosphere for 18 hours. The reaction mixture was diluted with saturated aqueous sodium thiosulfate (10 mL) and the organic products extracted using dichloromethane (4 x 10 mL). The organic extracts were dried over Na_2SO_4, filtered, the solvent removed under reduced pressure and the products triturated with diethylether (30 mL) – adamantan-1-ol soluble in diethyl ether, adamantan-1,3-diol insoluble. Adamantan-1,3-diol **28b** (30 mg, 0.18 mmol, 18%) was collected by vacuum filtration and the impure mixture containing adamantan-1-ol purified by flash column chromatography eluting with EtOAc in hexane (0–30%) to give adamantan-1-ol **28a** (73 mg, 48%) as a colourless amorphous solid.

b) Reaction set up outside of the glovebox under an ambient atmosphere: The reaction was carried out as indicated above using $RuCl_3$ (5.2 mg, 25 µmol, 5 mol%) in place of **RuAqua**. Quantitative ^{1}H NMR using 1,3,5-trimethoxybenzene as an internal standard indicated 50% conversion to the desired product (14:86 **28a:28b**).

c) Reaction set up outside of the glovebox under an ambient atmosphere: The reaction was carried out as indicated above using [Ru(*p*-cymene)Cl_2]$_2$ (7.7 mg, 25 µmol, 5 mol%) in place of **RuAqua**. Quantitative ^{1}H NMR using 1,3,5-trimethoxybenzene as an internal standard indicated 58% conversion to the desired product (40:60 **28a:28b**).

Characterisation for Adamantan-1-ol 28a

^{1}H NMR: (500 MHz, $CDCl_3$)

2.18 – 2.10 (m, 3H), 1.71 (d, *J* = 2.9 Hz, 6H), 1.68 – 1.54 (m, 7H).

^{13}C NMR: (101 MHz, $CDCl_3$)

68.3, 45.4, 36.1, 30.7.

IR: (*neat*, cm^{-1})

3348 (br), 2915, 2855, 1718, 907, 727.

m.p: 190–192 °C (EtOAc)

Spectroscopic data matched those previously reported[28].

Characterisation for Adamantan-1,3-diol 28b:

^{1}H NMR: (500 MHz, d_4-MeOD)

2.28 – 2.19 (m, 2H), 1.67 (s, 2H), 1.63 (d, *J* = 3.1 Hz, 6H), 1.52 – 1.47 (m, 2H).

^{13}C NMR: (126 MHz, d_4-MeOD)

70.8, 53.3, 44.7, 35.9, 32.6.

IR: (*neat*, cm^{-1})

3213 (br), 2930, 2850, 1298, 1133, 1026.

m.p: 248–250 °C (MeOH)

Spectroscopic data matched those previously reported[28].

RuAqua Catalysed Curtius Rearrangement

Preparation of Starting Material 29

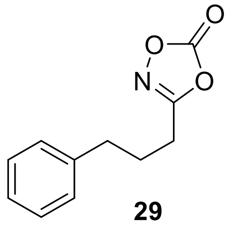

29

Compound **29** was prepared according to a previously reported procedure with data in accordance.[29]

^{1}H NMR: (500 MHz, $CDCl_3$)

7.32 (t, *J* = 7.4 Hz, 2H), 7.29 – 7.21 (m, 1H), 7.19 (d, *J* = 7.9 Hz, 2H), 2.75 (t, *J* = 7.4 Hz, 2H), 2.62 (t, *J* = 7.5 Hz, 2H), 2.07 (p, *J* = 7.4 Hz, 2H).

^{13}C NMR: (126 MHz, $CDCl_3$)

166.6, 154.2, 139.9, 128.8, 128.6, 126.7, 34.7, 26.0, 24.1.

Synthesis of (3-isocyanatopropyl)benzene 30

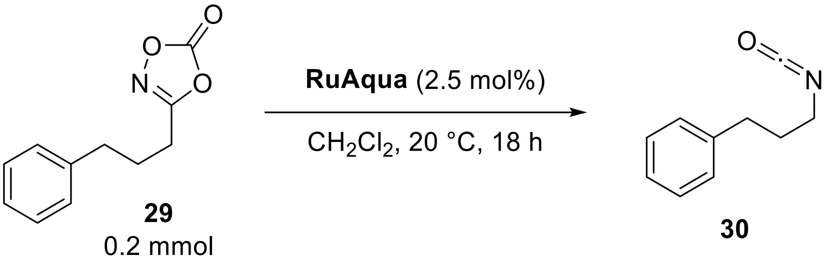

Reaction was discovered while testing various possible reactions and no specific optimisation was performed.

a) Reaction set up outside of the glovebox: To an oven dried microwave vial equipped with a magnetic stirrer bar was added dioxazolone **29** (41 mg, 0.2 mmol, 1 equiv.) and **RuAqua** (3.5 mg, 50 mmol, 2.5 mol%) before sealing the vessel and performing 3 x 5 min evac-refill cycles. Dry CH_2Cl_2 (2 mL, 0.1 M with respect to **29**) was added to the tube under the flow of nitrogen. The reaction mixture was then stirred at ambient temperature for 18 h. After this time, the reaction mixture was concentrated under *vacuum* and purified by flash column chromatography eluting with CH_2Cl_2 on silica gel to afford the product **30** as a colourless viscous oil (26 mg, 82%).
b) Reaction set up outside of the glovebox: The reaction was carried out as indicated above using $RuCl_3$ (1.0 mg, 25 μmol, 5 mol%) in place of **RuAqua**. ^{1}H NMR indicated no conversion to the desired product **30**.
c) Reaction set up outside of the glovebox: The reaction was carried out as indicated above using $[Ru(p\text{-cymene})Cl_2]_2$ (1.5 mg, 25 μmol, 5 mol%) in place of **RuAqua**. Quantitative ^{1}H NMR using 1,3,5-trimethoxybenzene as an internal standard indicated 74% conversion to the desired product **30**.

^{1}H NMR: (500 MHz, $CDCl_3$)

7.31 (dd, *J* = 8.2, 6.8 Hz, 2H), 7.25 – 7.17 (m, 3H), 3.32 (t, *J* = 6.6 Hz, 2H), 2.73 (t, *J* = 7.5 Hz, 2H), 1.99 – 1.89 (m, 2H).

^{13}C NMR: (126 MHz, $CDCl_3$)

140.7, 128.7, 128.6, 126.3, 42.3, 32.7 (2 x resonances).

Spectroscopic data matched those previously reported[29].

RuAqua Catalysed Oxidative Alkene Cleavage

Attainment of Starting Materials

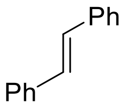

31a

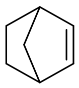

31b

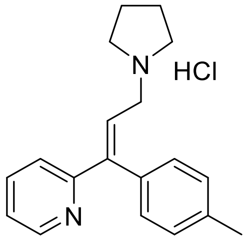

31c
Tripolidine

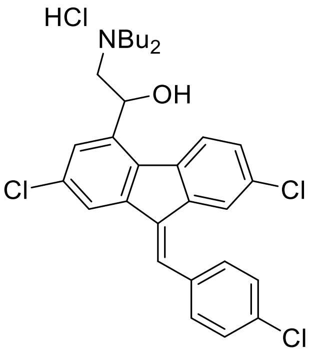

31d
Lumefantrine

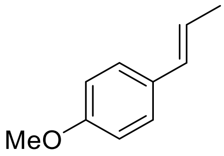

31e
Anethole

Starting materials **31a**, **31b**, **31c**, **31d** and **31e** were commercially available and were used without further purification.

General Procedure H: Oxidative Cleavage of Alkenes

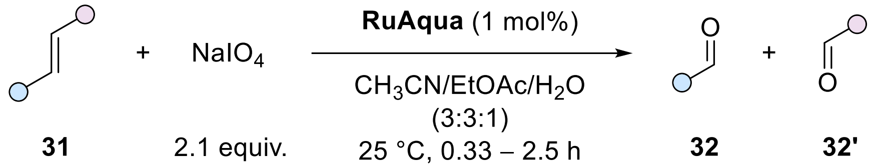

Carried out under ambient atmosphere. Reaction conditions were adapted from previously reported procedures lowering the equivalents of $NaIO_4$ from 4.0 to 2.1[30]. Reactions were carried out under atmospheric conditions. To a 50 mL round bottom flask equipped with a magnetic stirrer bar was added $NaIO_4$ (439 mg, 2.1 mmol, 2.1 equiv.), **RuAqua** (7.1 mg, 0.01 mmol, 1 mol%) and water (1.0 mL). This was stirred at room temperature for 10 mins producing a yellow solution to which was added MeCN (3.0 mL), and EtOAc (3.0 mL). After stirring the resulting solution for 2 mins the indicated substrate **31** was added (1 mmol, 1 equiv.). This was further stirred at 25 °C for the indicated time before being quenched with a mixture of saturated $Na_2S_2O_3$ (10.0 mL) and saturated $NaHCO_3$ (7.5 mL) solutions. The resulting mixture was then diluted with water (20 mL) and extracted with ethyl acetate (3 x 20 mL) before being dried over $MgSO_4$ and concentrated under reduced pressure. Products were isolated via column chromatography using the noted conditions.

Synthesis of benzaldehyde 32a

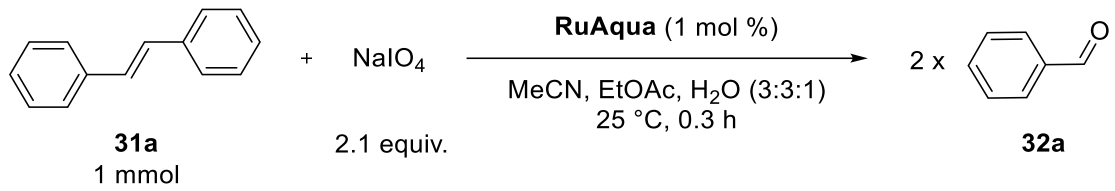

a) Carried out under ambient atmosphere: was carried out according to general procedure H using trans-stilbene **31a** (180 mg, 1 mmol) as a substrate allowing the reaction mixture to stir for 20 mins. After workup the reaction mixture was purified via column chromatography eluting with EtOAc in hexane (0–5%) giving the product **32a** as a yellow oil (177 mg, 83%).

b) Carried out under ambient atmosphere: Reaction was carried out according to general procedure H using $RuCl_3$ (2.0 mg, 10 μmol, 1 mol%) in place of **RuAqua** with trans-stilbene **31a** (180 mg, 1 mmol) as a substrate allowing the reaction mixture to stir for 20 mins. After workup, quantitative ^{1}H NMR using 1,3,5-trimethoxy benzene as an internal standard indicated conversion of 69% to the product **32a**.

c) Carried out under ambient atmosphere: Reaction was carried out according to general procedure H using $[Ru(p\text{-cymene})Cl_2]_2$ (3.1 mg, 5 μmol, 1 mol% with respect to Ru) in place of **RuAqua** with trans-stilbene **31a** (180 mg, 1 mmol) as a substrate allowing the reaction mixture to stir for 20 mins. After workup, quantitative ^{1}H NMR using 1,3,5-trimethoxy benzene as an internal standard indicated conversion of 61% to the product **32a**.

^{1}H NMR: (400 MHz, $CDCl_3$)

10.03 (s, 1H), 7.92 – 7.86 (m, 2H), 7.67 – 7.61 (m, 1H), 7.54 (t, J = 7.5 Hz, 2H).

^{13}C NMR: (101 MHz, $CDCl_3$)

192.5, 136.6, 134.6, 129.9, 129.2.

IR: V_{max} (neat/cm^{-1})

3070, 2830, 1672, 1323, 703.

Spectroscopic data matched those previously reported[31].

Synthesis of cyclopentane-1,3-dicarbaldehyde 32b

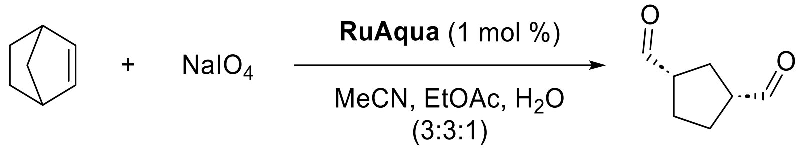

31b 1 mmol   2.1 equiv.   **32b** 68%

a) Carried out under ambient atmosphere: The reaction was carried out according to general procedure H using norbornene **31b** (97 µL, 1 mmol) as a substrate allowing the reaction mixture to stir for 30 mins at room temperature. After workup, the reaction mixture was purified via column chromatography eluting with EtOAc in hexane (0–10%) to give the product **32b** as a colourless oil (86 mg, 68%).

b) Carried out under ambient atmosphere: was carried out according to general procedure H using $RuCl_3$ (2.0 mg, 10 µmol, 1 mol%) in place of **RuAqua** with norbornene **31b** (97 µL, 1 mmol) as a substrate allowing the reaction mixture to stir for 30 mins. After workup, quantitative ^{1}H NMR using 1,3,5-trimethoxy benzene as an internal standard indicated conversion of 44% to the product **32b**.

c) Carried out under ambient atmosphere: Reaction was carried out according to general procedure H using $[Ru(p\text{-cymene})Cl_2]_2$ (3.1 mg, 5 µmol, 1 mol% with respect to Ru) in place of **RuAqua** with norbornene **31b** (97 µL, 1 mmol) as a substrate allowing the reaction mixture to stir for 30 mins. After workup, quantitative ^{1}H NMR using 1,3,5-trimethoxy benzene as an internal standard indicated conversion of 16% to the product **32b**.

^{1}H NMR: (400 MHz, $CDCl_3$)

9.68 – 9.64 (m, 2H), 2.96 – 2.82 (m, 1H), 2.36 – 2.18 (m, 1H), 2.21 – 1.99 (m, 2H), 1.96 – 1.87 (m, 2H).

^{13}C NMR: (101 MHz, $CDCl_3$)

202.6, 51.5, 26.4, 26.1.

IR: V_{max} (neat/cm^{-1})

2955, 2873, 1716, 1450, 1076, 945.

Spectroscopic data matched those previously reported[32].

Synthesis of pyridin-2-yl(*p*-tolyl)methanone 32c

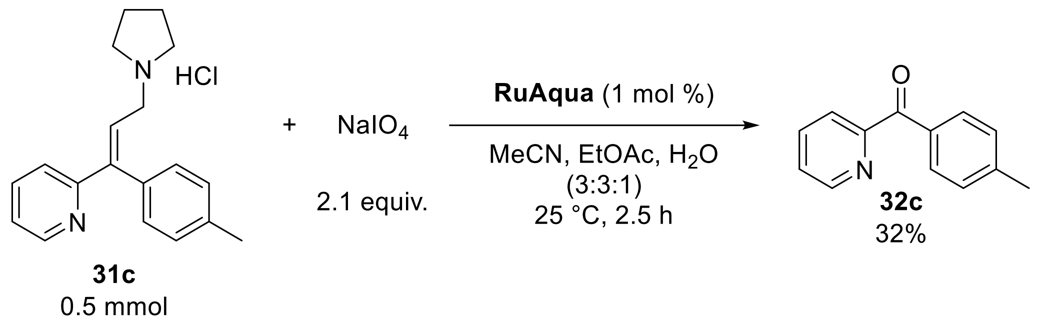

Carried out under ambient atmosphere: The reaction was carried out according to general procedure H using triprolidine HCl salt **31c** as a substrate (157 mg, 0.5 mmol, 1 equiv.), $NaIO_4$ (219 mg, 1 mmol, 1.0 equiv.), **RuAqua** (3.6 mg 5 µmol, 1 mol%), H_2O (0.5 mL), MeCN (1.5 mL) and EtOAc (1.5 mL) allowing the reaction mixture to stir for 2.5 h at room temperature. After workup, the reaction mixture was purified via column chromatography eluting with EtOAc in hexane (0–10%) to give the product **32c** (31 mg, 32%) as a pale-yellow oil.

^{1}H NMR: (400 MHz, $CDCl_3$)

8.72 (d, *J* = 4.5 Hz, 1H), 8.06 – 7.93 (m, 3H), 7.89 (td, *J* = 7.8, 1.8 Hz, 1H), 7.52 – 7.42 (m, 1H), 7.29 (d, *J* = 8.2 Hz, 2H), 2.43 (s, 3H).

^{13}C NMR: (126 MHz, $CDCl_3$)

193.7, 155.5, 148.6, 144.0, 137.2, 133.8, 132.1, 129.9, 126.1, 124.7, 21.9.

IR: V_{max} (neat/cm^{-1})

2920, 1657, 1604, 1314, 931, 744, 639.

Spectroscopic data matched those previously reported[33].

Synthesis of 2,7-dichloro-4-(2-(dibutylamino)-1-hydroxyethyl)-9H-fluoren-9-one 32d and 4-chlorobenzaldehyde 32d' from Lumefantrine

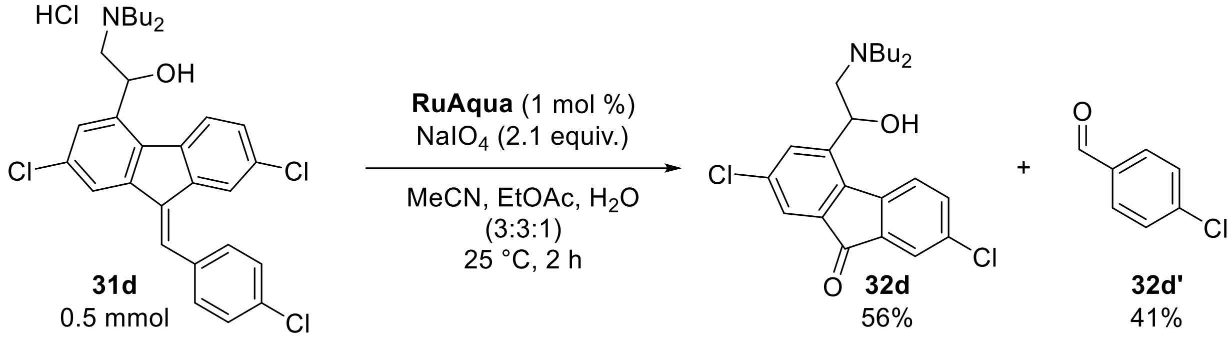

Carried out under ambient atmosphere: The reaction was carried out according to general procedure H using Lumefantrine HCl salt **31d** (265 mg, 0.5 mmol, 1 equiv.), $NaIO_4$ (219 mg, 1 mmol, 1.0 equiv.), **RuAqua** (3.6 mg, 5 μmol, 1 mol%), H_2O (0.5 mL), MeCN (1.5 mL) and EtOAc (1.5 mL) allowing the reaction mixture to stir for 2 h at room temperature. After workup, the reaction mixture was purified via column chromatography eluting with EtOAc in hexane (0–10%) to give the products **32d** (yellow oil, 118 mg, 56% yield) and **32d'** (yellow amorphous solid, 28 mg, 41% yield).

Characterisation for 32d

^{1}H NMR: (400 MHz, $CDCl_3$)

7.81 (d, *J* = 2.1 Hz, 1H), 7.66 (t, *J* = 1.3 Hz, 1H), 7.56 (d, *J* = 2.0 Hz, 1H), 7.50 – 7.43 (m, 2H), 5.18 (d, *J* = 9.7 Hz, 1H), 4.56 (s, 1H), 2.82 (dd, *J* = 13.0, 3.6 Hz, 1H), 2.68 (dq, *J* = 15.8, 7.8 Hz, 2H), 2.49 (dd, *J* = 27.6, 14.9 Hz, 3H), 1.55 – 1.23 (m, 8H), 0.97 (t, *J* = 7.3 Hz, 6H).

^{13}C NMR: (101 MHz, $CDCl_3$)

191.2, 141.9, 140.9, 138.1, 136.1 (2 x resonances), 136.0, 135.2, 134.5, 132.4, 125.2, 124.9, 123.9, 65.6, 60.0, 53.5, 29.1, 20.7, 14.2.

IR: V_{max} (neat/cm^{-1})

3405 (br), 2956, 2931, 2862, 1722, 1598, 1489, 905, 729.

Characterisation for 32d'

^{1}H NMR: (500 MHz, $CDCl_3$)

9.99 (s, 1H), 7.83 (d, *J* = 8.2 Hz, 2H), 7.52 (d, *J* = 8.2 Hz, 2H).

^{13}C NMR: (126 MHz, $CDCl_3$)

191.0, 141.1, 134.9, 131.1, 129.6.

IR: V_{max} (neat/cm^{-1})

3088, 2961, 2858, 1690, 1586, 1573, 1205, 1010, 813.

m.p: 40–43 °C (EtOAc)

Spectroscopic data matched those previously reported[33].

Synthesis of 4-methoxybenzaldehyde 32e

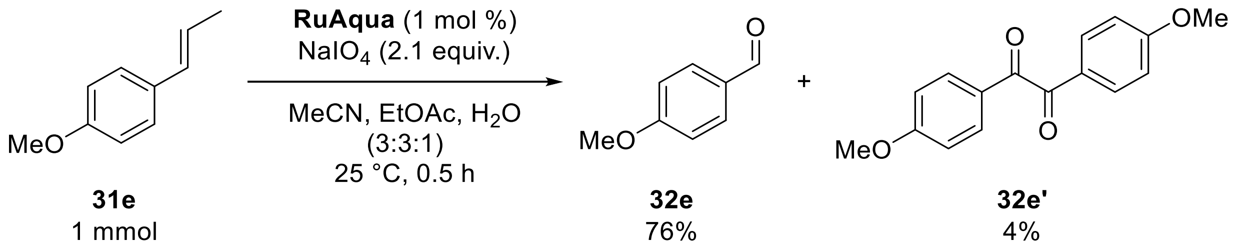

Carried out under ambient atmosphere: Reaction was carried out according to general procedure H using anethole **31e** (150 μL, 1 mmol) as a substrate allowing the reaction mixture to stir for 30 mins. After workup, the reaction mixture was purified via column chromatography eluting with EtOAc in hexane (0–5%) giving the product **32e** as a colourless oil (114 mg, 76%) containing byproduct **32e'** as an impurity (9% of sample).

^{1}H NMR: (400 MHz, $CDCl_3$)

9.89 (s, 1H), 7.87 – 7.80 (m, 2H), 7.04 – 6.98 (m, 2H), 3.89 (s, 3H).

^{13}C NMR: (101 MHz, $CDCl_3$)

191.0, 164.8, 132.2, 130.1, 114.4, 55.7.

IR: V_{max} (neat/cm^{-1})

2938, 2841, 1922, 1805, 1736, 1600, 1506, 1257, 1158, 1019, 841, 693.

Spectroscopic data matched those previously reported[34]. For dimerised byproduct[35].

RuAqua Catalysed Transfer Hydrogenation

Attainment of Starting Materials

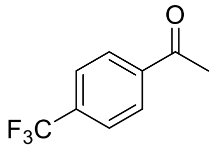

33a

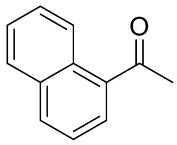

33b

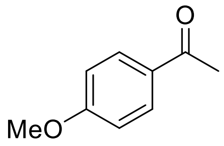

33c

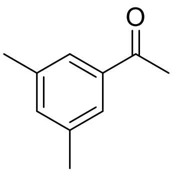

33d

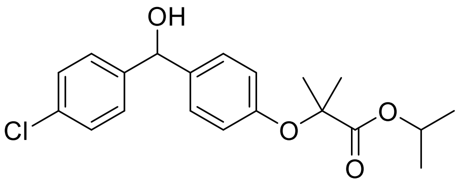

33e
fenofibrate

Starting materials **33a**, **33b**, **33c**, **33d** and **33e** were commercially available and used without further purification.

General Procedure I: RuAqua Catalysed Transfer Hydrogenation

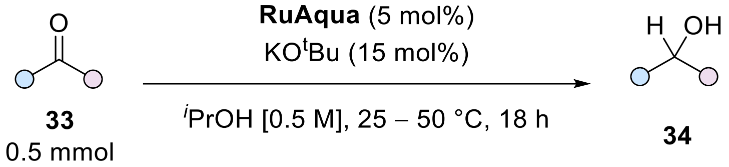

Reaction set up outside of the glovebox: Conditions adapted from previously reported procedures with no specific optimisation for **RuAqua** performed[36]. The indicated ketone **33** (0.5 mmol) was added to a mixture of potassium *tert*-butoxide (9 mg, 75 µmol, 15 mol%) and **RuAqua** (17.5 mg, 15 µmol, 5 mol%) in a 7 mL microwave vial equipped with a magnetic stirrer bar. The headspace was purged with N_2 for 5 minutes before the addition of N_2-degassed isopropanol (0.5 mL). The reaction mixture was stirred at either 25 °C or 50 °C for 18 hours. The reaction mixture was cooled to room temperature and the crude reaction mixture directly purified by flash column chromatography using the indicated conditions to give product **34**.

Synthesis of 1-(4-(trifluoromethyl)phenyl)ethan-1-ol 34a

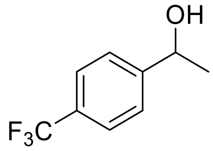

34a

a) Reaction set up outside of the glovebox: The reaction was carried out according to general procedure I using 4-trifluoromethylacetophenone **33a** (94 mg, 0.5 mmol), potassium *tert*-butoxide (9 mg, 75 µmol, 15 mol%) and **RuAqua** (17.5 mg, 15 µmol, 5 mol%) which were stirred in isopropanol (1.0 mL) at 25 °C for 18 hours. The solvent was removed under reduced pressure and the crude reaction product purified by flash column chromatography eluting with ethyl acetate in hexane (0–30%) to give the product **34a** (83 mg, 87%) as a colourless oil.

b) Reaction set up outside of the glovebox: The reaction was carried out according to general procedure I using 4-trifluoromethylacetophenone **33a** (94 mg, 0.5 mmol), potassium *tert*-butoxide (9 mg, 75 µmol, 15 mol%) and **RuAqua** (17.5 mg, 15 µmol, 5 mol%) which were stirred in isopropanol (1.0 mL) at 25 °C for 8 hours. After this time, quantitative ^{1}H NMR using 1,3,5-trimethoxybenzene as an internal standard indicated 79% conversion to **34a**.

c) Reaction set up outside of the glovebox: The reaction was carried out according to general procedure I using 4-trifluoromethylacetophenone **33a** (94 mg, 0.5 mmol), potassium *tert*-butoxide (9 mg, 75 µmol, 15 mol%) and $RuCl_3$ (5.0 mg, 15 µmol, 5 mol%) which were stirred in isopropanol (1.0 mL) at 25 °C for 8 hours. After this time, quantitative ^{1}H NMR using 1,3,5-trimethoxybenzene as an internal standard indicated 2% conversion to **34a**.

d) Reaction set up outside of the glovebox: The reaction was carried out according to general procedure I using 4-trifluoromethylacetophenone **33a** (94 mg, 0.5 mmol), potassium *tert*-butoxide (9 mg, 75 µmol, 15 mol%) and [Ru(*p*-cymene)Cl_2]$_2$ **1** (7.5 mg, 15 µmol, 5 mol%) which were stirred in isopropanol (1.0 mL) at 25 °C for 8 hours. After this time, quantitative ^{1}H NMR using 1,3,5-trimethoxybenzene as an internal standard indicated 10% conversion to **34a**.

^{1}H NMR: (400 MHz, $CDCl_3$)

7.61 (d, *J* = 8.1 Hz, 2H), 7.49 (d, *J* = 7.6 Hz, 2H), 4.98 (q, *J* = 6.5 Hz, 1H), 1.82 (bs, 1H), 1.51 (d, *J* = 6.5 Hz, 3H).

^{13}C NMR: (101 MHz, $CDCl_3$)

149.8, 129.7 (q, *J* = 32.3 Hz), 125.8, 125.6 (q, *J* = 4.0 Hz), 124.3 (q, *J* = 272.0 Hz), 69.9, 25.5.

^{19}F NMR: (376 MHz, $CDCl_3$)

-62.4.

IR: (*neat*, cm^{-1})

3332 (OH), 2978, 1415, 1322, 1065.

Spectroscopic data matched those previously reported[37].

Synthesis of 1-(Naphthalen-1-yl)ethan-1-ol 34b

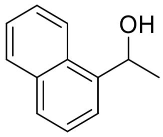

34b

a) Reaction set up outside of the glovebox: The reaction was carried out following general procedure I using 1-(naphthalen-1-yl)ethan-1-one **33b** (85 mg, 0.5 mmol), potassium *tert*-butoxide (9 mg, 75 µmol, 15 mol%) and **RuAqua** (17.5 mg, 15 µmol, 5 mol%) were reacted in isopropanol (1.0 mL) at 50 °C for 18 hours. The solvent was removed under reduced pressure and the crude reaction product purified by flash column chromatography eluting with EtOAc in hexane (0–20%) to give the product **34b** (73 mg, 85%) as a colourless oil.

b) Reaction set up outside of the glovebox: The reaction was carried out following general procedure I using 1-(naphthalen-1-yl)ethan-1-one **33b** (85 mg, 0.5 mmol), potassium *tert*-butoxide (9 mg, 75 µmol, 15 mol%) and $RuCl_3$ (5.0 mg, 15 µmol, 5 mol%) were reacted in isopropanol (1.0 mL) at 50 °C for 18 hours. Quantitative ^{1}H NMR using 1,3,5-trimethoxybenzene as an internal standard indicated conversion of >95% to the desired product **34b**. Reproducibility of this result was low with yields massively varying between 10% and >95% conversion. This is a common problem when employing $RuCl_3$ due to its hygroscopic nature and the different morphologies between batches.

c) Reaction set up outside of the glovebox: The reaction was carried out following general procedure I using 1-(naphthalen-1-yl)ethan-1-one **33b** (85 mg, 0.5 mmol), potassium *tert*-butoxide (9 mg, 75 µmol, 15 mol%) and [Ru(*p*-cymene)Cl_2]$_2$ **1** (7.5 mg, 15 µmol, 5 mol%) were reacted in isopropanol (1.0 mL) at 50 °C for 18 hours. Quantitative ^{1}H NMR using 1,3,5-trimethoxybenzene as an internal standard indicated conversion of >95% to the desired product **34b**.

^{1}H NMR: (500 MHz, $CDCl_3$)

8.19 – 8.07 (m, 1H), 7.95 – 7.84 (m, 1H), 7.79 (d, *J* = 8.2 Hz, 1H), 7.69 (dt, *J* = 7.1, 1.1 Hz, 1H), 7.55 – 7.46 (m, 3H), 5.69 (q, *J* = 6.5 Hz, 1H), 1.94 (bs, 1H), 1.68 (d, *J* = 6.4 Hz, 3H).

^{13}C NMR: (126 MHz, $CDCl_3$)

141.5, 134.0, 130.4, 129.0, 128.1, 126.2, 125.7 (2 x resonances), 123.3, 122.1, 67.3, 24.5.

IR: (*neat*, cm^{-1})

3338 (br), 2973, 776.

Spectroscopic data matched those previously reported[38].

Synthesis of 1-(4-Methoxyphenyl)ethan-1-ol 34c

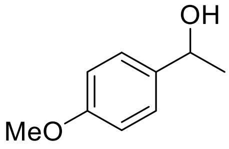

34c

Reaction set up outside of the glovebox: The reaction was carried out following general procedure I using a mixture of 1-(4-methoxyphenyl)ethan-1-one **33c** (75 mg, 0.5 mmol), potassium *tert*-butoxide (9 mg, 75 µmol, 15 mol%) and **RuAqua** (17.5 mg, 15 µmol, 5 mol%) in isopropanol (1.0 mL) at 50 °C for 18 hours. The solvent was removed under reduced pressure and the crude reaction product purified by flash column chromatography eluting with EtOAc in hexane (0–30%) to give the product **34c** (42 mg, 55%) as a colourless oil.

^{1}H NMR: (500 MHz, $CDCl_3$)

7.34 – 7.27 (m, 2H), 6.93 – 6.81 (m, 2H), 4.85 (q, *J* = 6.4 Hz, 1H), 3.80 (s, 3H), 1.84 (bs, 1H), 1.47 (d, *J* = 6.4 Hz, 3H).

^{13}C NMR: (126 MHz, $CDCl_3$)

159.0, 138.0, 126.7, 113.9, 70.0, 55.3, 25.0.

IR: (*neat*, cm^{-1})

3420 (br), 2968, 1511, 1303, 1088.

Spectroscopic data matched those previously reported[39].

Synthesis of 1-(3,5-Dimethylphenyl)ethan-1-ol 34d

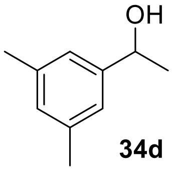

34d

Reaction set up outside of the glovebox: The reaction was carried out following general procedure I using 1-(3,5-dimethylphenyl)ethan-1-one **34d** (75 mg, 0.5 mmol), potassium *tert*-butoxide (9 mg, 75 µmol, 15 mol%) and **RuAqua** (17.5 mg, 15 µmol, 5 mol%) in isopropanol (1.0 mL) at 50 °C for 18 hours. The solvent was removed under reduced pressure and the crude reaction product purified by flash column chromatography eluting with EtOAc in hexane (0–30%) to give the product **34d** (47 mg, 63%) as a colourless oil.

^{1}H NMR: (500 MHz, $CDCl_3$)

7.00 (s, 2H), 6.93 (s, 1H), 4.83 (q, *J* = 6.5 Hz, 1H), 2.33 (s, 6H), 1.86 (bs, 1H), 1.49 (d, *J* = 6.5 Hz, 3H).

^{13}C NMR: (126 MHz, $CDCl_3$)

145.9, 138.2, 129.2, 123.3, 70.6, 25.2, 21.5.

IR: (*neat*, cm^{-1})

3346 (br), 3011, 1607, 1450, 847.

Spectroscopic data matched those previously reported[40].

Synthesis of Isopropyl 2-{4-[(4-chlorophenyl)(hydroxy)methyl]phenoxy}-2-methylpropanoate 34e

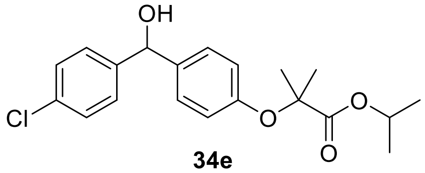

34e

Reaction set up outside of the glovebox: The reaction was carried out following general procedure I using a mixture of fenofibrate **33e** (181 mg, 0.5 mmol), potassium *tert*-butoxide (9 mg, 75 µmol, 5 mol%) and **RuAqua** (17.5 mg, 15 µmol, 5 mol%) in isopropanol (1.0 mL) at 50 °C for 18 hours. The solvent was removed under reduced pressure and the crude reaction product purified by flash column chromatography eluting with EtOAc in hexane (0–30%) to give the product **34e** (54 mg, 30%) as a colourless oil.

^{1}H NMR: (500 MHz, $CDCl_3$)

7.28 – 7.23 (m, 4H), 7.18 – 7.09 (m, 2H), 6.81 – 6.70 (m, 2H), 5.72 (s, 1H), 5.04 (hept, *J* = 6.3 Hz, 1H), 1.54 (s, 6H), 1.18 (d, *J* = 6.2 Hz, 6H).

^{13}C NMR: (126 MHz, $CDCl_3$)

173.8, 155.3, 142.5, 137.0, 133.3, 128.6, 127.9, 127.6, 119.0, 79.3, 77.4, 75.2, 69.1, 25.5, 21.7.

IR: (*neat*, cm^{-1})

3471 (OH), 2982, 1726, 1489, 1100.

Spectroscopic data matched those previously reported[41].

Photocatalyst Synthesis via Mixer Mill

Attainment of Starting Materials

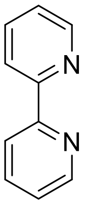

L1

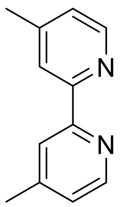

L2

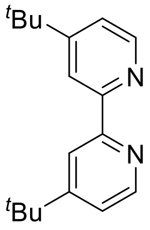

L3

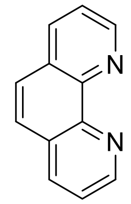

L10

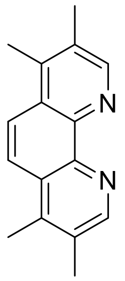

L12

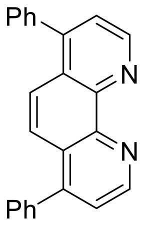

L13

Starting Materials **L1**, **L2**, **L3**, **L10**, **L12** and **L13** were commercially available and were used in the reaction without further purification.

General Procedure J: Synthesis of Tris(bipyridine)ruthenium Derivatives

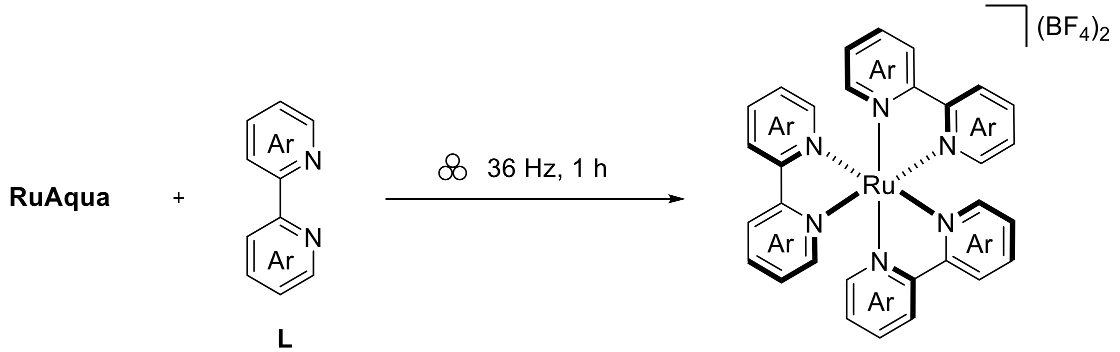

L
(3 equiv.)

35 – **40**

Reactions were carried out under an ambient atmosphere with no need for inertion. Previously reported conditions often rely on harsh conditions and temperatures of up to 250 °C[42]. Reaction was discovered while testing various possible reactions and no specific optimisation was performed. The use of 4 × 7 mm ball bearings was found to perform better than 2 × 7 mm ball bearings.

Experimental Procedure:

A 15 mL milling jar was loaded with **RuAqua** (15 mg, 21 μmol, 1 equiv.), the indicated ligand (63 μmol, 3 equiv.) and 4 x 7 mm grinding balls. The vessel was sealed before ball milling at 36 Hz for 1 hour. After this time, the reaction crude was washed with MeOH, filtered and concentrated under reduced pressure. The product was then washed with hexane (3 x 20 mL) and either EtOAc (3 x 20 mL) or Et_2O (3 x 20 mL) before being dissolved in MeOH, filtered and concentrated to give the desired product.

Synthesis of Tris(2,2′-bipyridine)ruthenium(II) tetrafluoroborate 35

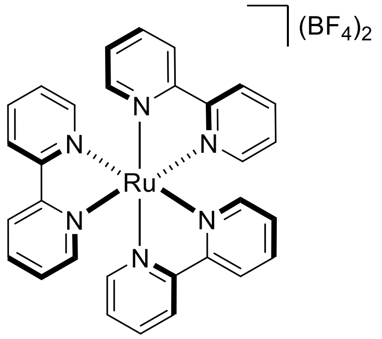

35

a) Carried out under ambient atmosphere: Prepared according to general procedure J using 2,2-bipyridine **L1** (10 mg, 63 μmol, 3 equiv.) as a ligand. Solid was washed with hexane and EtOAc to give the product **35** as a red amorphous solid (12 mg, 80%).

b) Carried out under ambient atmosphere: Prepared according to general procedure J using $RuCl_3$ (4.4 mg, 21 μmol, 1 equiv.) in place of **RuAqua** and 2,2-bipyridine **L1** (10 mg, 63 μmol, 3 equiv.) as a ligand. Quantitative ^1H NMR using 1,3,5-trimethoxybenzene as an internal standard indicated conversion of 22% to the desired product **35**.

c) Carried out under ambient atmosphere: Prepared according to general procedure J using $[Ru(p\text{-cymene})Cl_2]_2$ **1** (6.5 mg, 11 μmol, 1 equiv. with respect to Ru) in place of **RuAqua** and 2,2-bipyridine **L1** (10 mg, 63 μmol, 3 equiv.) as a ligand. Quantitative ^1H NMR using 1,3,5-trimethoxybenzene as an internal standard indicated conversion of 2% to the desired product **35**.

^{1}H NMR: (400 MHz, d_6-DMSO)

8.84 (d, *J* = 7.6 Hz, 6H), 8.17 (t, *J* = 6.7 Hz, 6H), 7.72 (d, *J* = 4.5 Hz, 6H), 7.52 (t, *J* = 5.8 Hz, 6H).

^{13}C NMR: (126 MHz, d_6-DMSO)

156.6, 151.2, 138.0, 127.9, 124.6.

Spectroscopic data matched those previously reported[42].

Synthesis of Tris(*p*-Me-2,2'-bipyridine)ruthenium(II) tetrafluoroborate 36

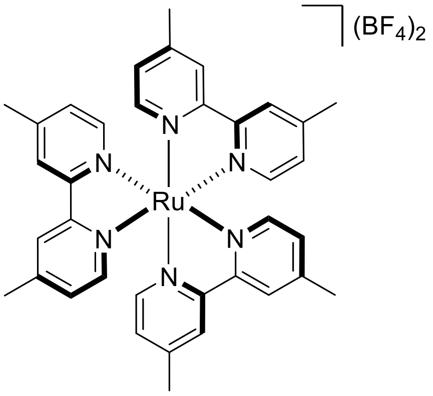

36

Carried out under ambient atmosphere: Prepared according to general procedure J using *p*-Me-2,2'-bipyridine **L2** (12 mg, 63 μmol, 3 equiv.) as a ligand. Solid was washed with hexane and EtOAc to give the product **36** as a red amorphous solid (13 mg, 87%).

^{1}H NMR: (500 MHz, d_6-DMSO)

8.69 (s, 6H), 7.54 (d, *J* = 5.8 Hz, 6H), 7.35 (d, *J* = 6.0 Hz, 6H).

^{13}C NMR: (126 MHz, d_6-DMSO)

156.7, 150.7, 149.8, 128.9, 125.4, 21.2.

Spectroscopic data matched those previously reported[42].

Synthesis of Tris(*p*-^{t}Bu-2,2'-bipyridine)ruthenium(II) tetrafluoroborate 37

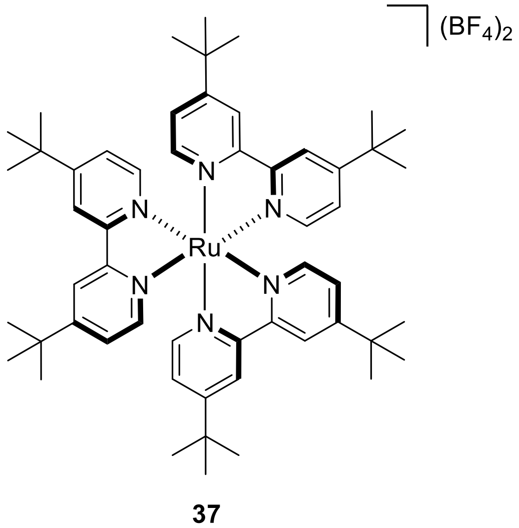

37

Carried out under ambient atmosphere: Prepared according to general procedure J using *p*-^{t}Bu-2,2'-bipyridine **L3** (17 mg, 63 μmol, 3 equiv.) as a ligand. Solid was washed with hexane and Et_2O to give the product **37** as a red amorphous solid (23 mg, 86%).

^{1}H NMR: (400 MHz, d_6-DMSO)

8.84 (s, 6H), 7.56 (dd, *J* = 6.1, 2.1 Hz, 6H), 7.51 (d, *J* = 6.0 Hz, 6H), 1.39 (s, 54H).

^{13}C NMR: (101 MHz, d_6-DMSO)

161.6, 156.3, 150.4, 124.7, 122.8, 35.5, 30.0.

Spectroscopic data matched those previously reported[42].

Synthesis of Tris(1,10-phenanthroline)ruthenium(II) tetrafluoroborate 38

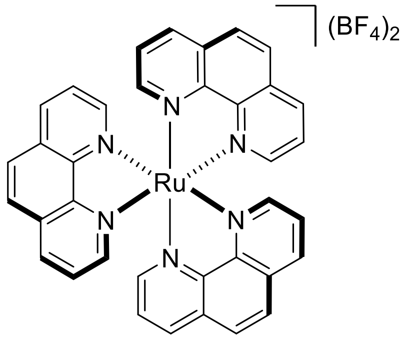

38

Carried out under ambient atmosphere: Prepared according to general procedure J using 1,10-phenanthroline **L10** (10 mg, 63 μmol, 3 equiv.) as a ligand. Solid was washed with hexane and EtOAc to give the product **38** as a red amorphous solid (15 mg, 77%).

^{1}H NMR: (500 MHz, d_6-DMSO)

8.78 (d, *J* = 9.5 Hz, 1H), 8.39 (s, 1H), 8.08 (d, *J* = 6.4 Hz, 1H), 7.77 (dd, *J* = 8.2, 5.2 Hz, 1H).

^{13}C NMR: (126 MHz, d_6-DMSO)

152.8, 147.2, 136.8, 130.4, 128.0, 126.3.

Spectroscopic data matched those previously reported[42].

Synthesis of Tris(3,4,7,8-tetramethyl phenanthroline)ruthenium(II) tetrafluoroborate 39

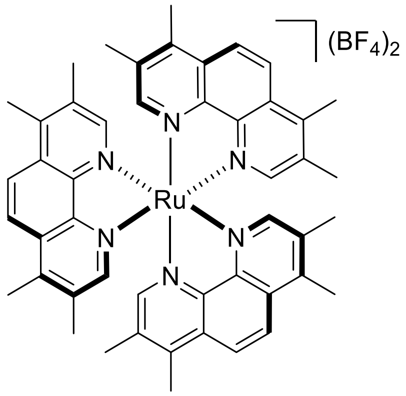

39

Carried out under ambient atmosphere: Prepared according to general procedure J using 3,4,7,8-tetramethyl phenanthroline **L12** (15 mg, 63 μmol, 3 equiv.) as a ligand. Solid was washed with hexane and Et_2O to give the product **39** as a red amorphous solid (16 mg, 78%).

^{1}H NMR: (400 MHz, d_6-DMSO)

8.48 (s, 6H), 7.68 (s, 6H), 2.77 (s, 18H), 2.22 (s, 18H).

^{13}C NMR: (101 MHz, d_6-DMSO)

151.9, 146.0, 144.1, 134.4, 128.7, 124.3, 17.6, 14.5.

Spectroscopic data matched those previously reported[42].

Synthesis of Tris(bathophenanthroline)ruthenium(II) tetrafluoroborate 40

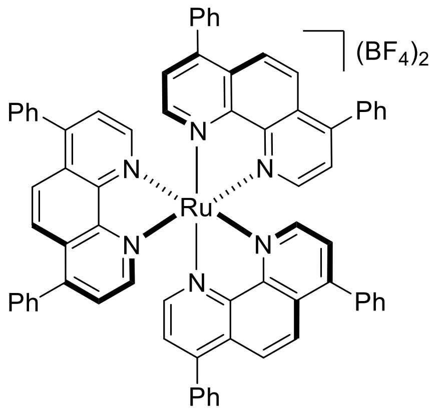

Carried out under ambient atmosphere: Prepared according to general procedure J on a 42 μmol scale split equally over two milling vessels using bathophenanthroline **L13** (42 mg, 126 μmol, 3 equiv.) as a ligand. Solid was washed with hexane and Et_2O to give the product **40** as a red amorphous solid (44 mg, 82%).

^{1}H NMR: (400 MHz, d_6-DMSO)

8.37 (s, 6H), 8.30 (s, 6H), 7.86 (s, 6H), 7.75 – 7.58 (m, 30H).

^{13}C NMR: (101 MHz, d_6-DMSO)

152.4, 148.0, 135.4, 129.9, 129.7, 129.2, 128.1, 126.5, 126.1.

^{19}F NMR: (471 MHz, d_4-MeOD)

–154.8

MS: HRMS (ESI^+)

Calculated for C_{72} $H_{48}N_6Ru$: 549.1486. Found: 549.1498

IR: V_{max} (neat, cm^{-1})

3057, 1621, 1594, 1556, 1416, 1398, 1050, 847, 736, 700.

m.p: Decomposition point > 250 °C (Et_2O).

Data consistent with analogous species in the literature[43].

RuAqua as a Platform pre-Catalyst for the Discovery of New Reactions

Simulated High-Throughput Reaction Discovery Using RuAqua

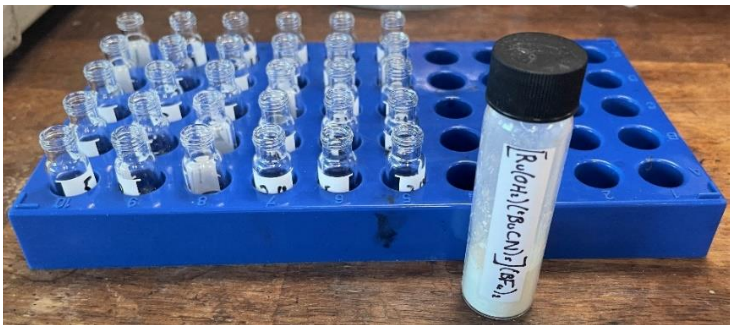

Figure 12 Set up of High-Throughput Reaction discovery with **RuAqua**

All substrates were commercially available and were used in the reaction without further purification. Reactions set up in a microwave vial in an argon filled glovebox: **RuAqua** (9 mg, 13 µmol, 5 mol%), phenylsilane (61 µL, 0.5 mmol, 2.0 equiv.) or pinacolborane (73 µL, 0.5 mmol, 2.0 equiv.) and a reducible substrate were loaded into a 2 mL HPLC vial with a screwcap lid. The reaction mixtures were stirred at room temperature for 24 hours before the addition of 1,3,5-trimethoxybenzene (1 mL, 0.2 M in $CDCl_3$) as an internal standard. The conversions and yields for the reactions were determined by integration of residual starting material and any product resonances with respect to 1,3,5-trimethoxybenzene.

Substrate Reduction using Phenylsilane

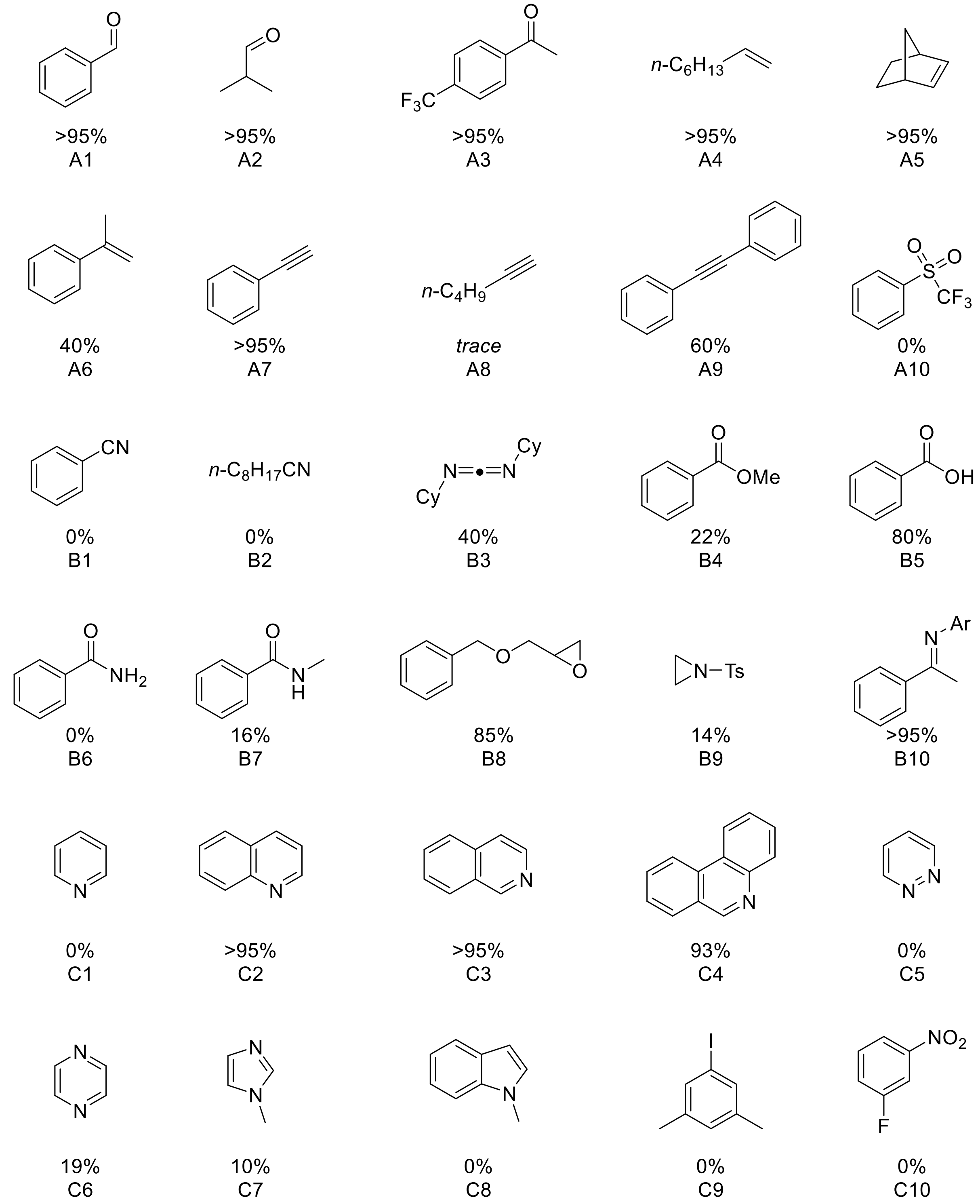

Figure 13 Substrate pool for reduction with phenylsilane

Substrate Reduction using Pinacolborane

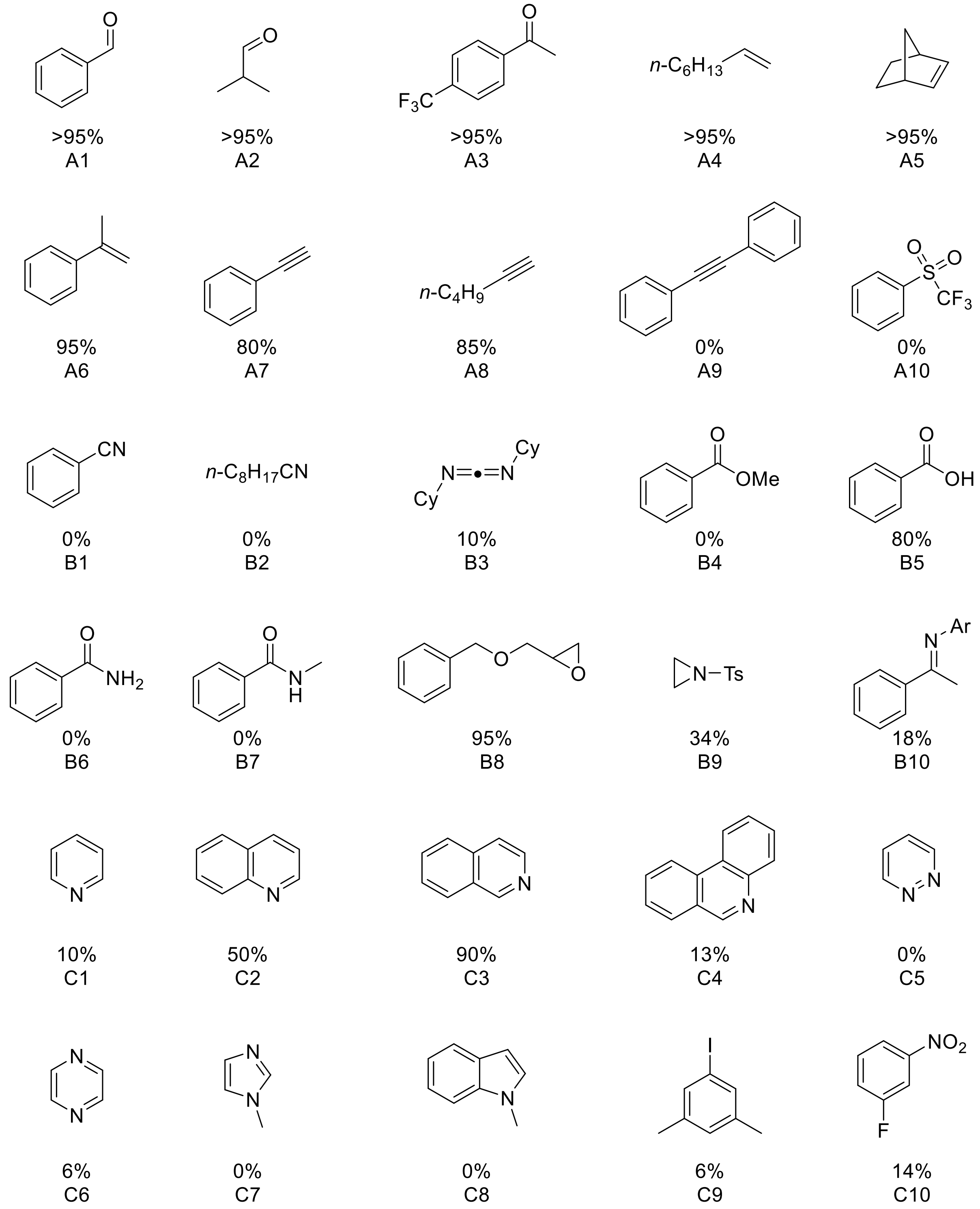

Figure 14 Substrate pool for reduction with pinacolborane

General Procedure K: Validation of RuAqua Catalysed Hydrosilylation

Reducable substrate (1 mmol) + H_3SiPh (2 equiv) —**RuAqua** (5 mol%), neat, rt, 24 h→ reduced product

Reactions set up in the glovebox: To an oven-dried microwave vial equipped with a magnetic stirrer bar was added **RuAqua** (35.4 mg, 0.05 mmol, 5 mol%), phenylsilane (249 μL, 2 mmol, 2 equiv.) and the indicated substrate. The resulting mixture was capped and allowed to stir at room temperature for 24 hours. ***Warning: These conditions have been known to undergo a redistribution reaction leading to formation of pyrophoric gas which can undergo spontaneous combustion***. After this time, the cap was removed inside the glovebox and the microwave vial was allowed to vent for 30 minutes while stirring at room temperature. The microwave vial was then removed from the glovebox and purified using flash column chromatography using the conditions indicated.

Synthesis of octyl(phenyl)silane 43

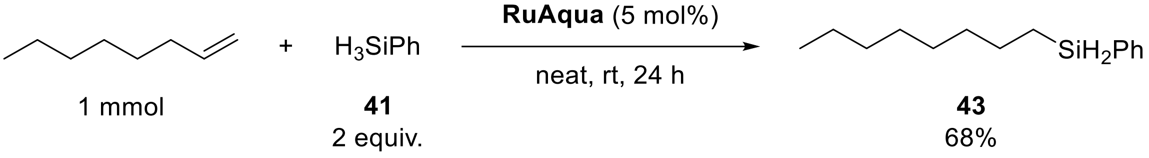

a) Reaction set up in an argon filled glovebox: Compound **43** was prepared according to general procedure K using 1-octene **A4** (158 µL, 1 mmol, 1 equiv.) as a substrate. The substrate was then purified using flash column chromatography on silica gel eluting with *n*-hexane giving the product **43** as a colourless oil (149 mg, 68%).

b) Reaction set up in an argon filled glovebox: Reaction performed according to general procedure K using 1-octene **A4** (158 µL, 1 mmol, 1 equiv.) as a substrate and $RuCl_3$ (10.4 mg, 0.05 mmol, 5 mol%) in place of **RuAqua**. Quantitative ^{1}H NMR using 1,3,5-trimethoxybenzene as an internal standard indicated 4% conversion to product **43**.

c) Reaction set up in an argon filled glovebox: Reaction performed according to general procedure K using 1-octene **A4** (158 µL, 1 mmol, 1 equiv.) as a substrate and **1** (15.3 mg, 0.05 mmol, 5 mol% with respect to Ru) in place of **RuAqua**. Quantitative ^{1}H NMR using 1,3,5-trimethoxybenzene as an internal standard indicated 9% conversion to product **43**.

^{1}H NMR: (400 MHz, $CDCl_3$)

7.64 – 7.51 (m, 2H), 7.44 – 7.31 (m, 3H), 4.28 (t, 2H, *J* = 3.7), 1.51 – 1.40 (m, 2H), 1.40 – 1.31 (m, 2H), 1.27 (d, *J* = 11.9 Hz, 8H), 1.06 – 0.71 (m, 5H).

^{13}C NMR: (126 MHz, $CDCl_3$)

135.4, 133.0, 129.6, 128.1, 33.0, 32.0, 29.4 (2 x resonances) 25.2, 22.9, 14.3, 10.2.

IR: V_{max} (neat/cm^{-1})

3052, 2956, 2853, 2130, 1485, 1463, 1116, 935, 840.

Spectroscopic data matched those previously reported[44].

Synthesis of phenylmethanol 44

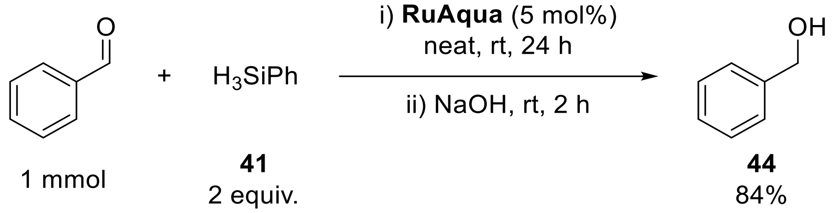

a) Reaction set up in an argon filled glovebox: Compound **44** was prepared according to general procedure K using benzaldehyde **A1** (102 μL, 1 mmol, 1 equiv.) as a substrate. After venting the reaction mixture and removing it from the glovebox, NaOH (1 M, 5 mL) was added and the reaction was stirred for 2 h before aqueous workup. The substrate was then purified using flash column chromatography on silica gel eluting with EtOAc in hexane (0–10%) giving the product **44** as a yellow oil (90 mg, 84%).
b) Reaction set up in an argon filled glovebox: Reaction performed according to general procedure K using benzaldehyde **A1** (102 μL, 1 mmol, 1 equiv.) as a substrate and $RuCl_3$ (10.4 mg, 0.05 mmol, 5 mol%) in place of **RuAqua**. Quantitative ^{1}H NMR using 1,3,5-trimethoxybenzene as an internal standard indicated 72% conversion to product **44**.
c) Reaction set up in an argon filled glovebox: Reaction performed according to general procedure K using benzaldehyde **A1** (102 μL, 1 mmol, 1 equiv.) as a substrate and **1** (15.3 mg, 0.05 mmol, 5 mol% with respect to Ru) in place of **RuAqua**. Quantitative ^{1}H NMR using 1,3,5-trimethoxybenzene as an internal standard indicated 86% conversion to product **44**.

^{1}H NMR: (500 MHz, d_6-Acetone)

7.38 – 7.27 (m, 4H), 7.25 – 7.20 (m, 1H), 4.62 (d, *J* = 4.7 Hz, 2H), 4.23 (t, *J* = 5.6 Hz, 1H).

^{13}C NMR: (126 MHz, $CDCl_3$)

141.0, 128.7, 127.7, 127.1, 65.4.

IR: V_{max} (neat/cm^{-1})

3316 (OH), 3069, 3029, 2980, 2873, 1495, 1129, 731, 694.

Spectroscopic data matched those previously reported[45].

Synthesis of 1-(4-(trifluoromethyl)phenyl)ethan-1-ol 34a

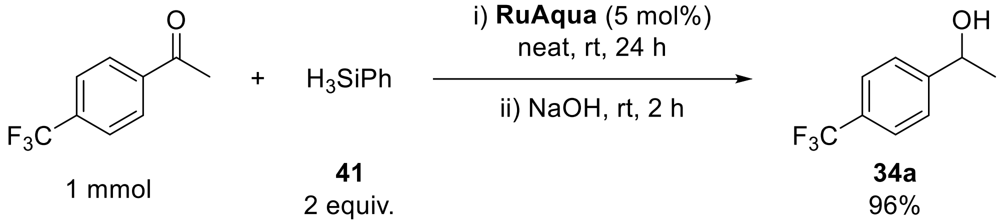

a) Reaction set up in an argon filled glovebox: Compound **34a** was prepared according to general procedure K using 1-(4-(trifluoromethyl)phenyl)ethan-1-one **A3** (188 mg, 1 mmol, 1 equiv.) as a substrate. After venting the reaction mixture and removing it from the glovebox, NaOH (1 M, 5 mL) was added and the reaction was stirred for 2 h before aqueous workup. The substrate was then purified using flash column chromatography on silica gel eluting with EtOAc in *n*-hexane (0–10%) giving the product **34a** as a yellow oil (183 mg, 96%).

b) Reaction set up in an argon filled glovebox: Reaction performed according to general procedure K using 1-(4-(trifluoromethyl)phenyl)ethan-1-one **A3** (188 mg, 1 mmol, 1 equiv.) as a substrate and $RuCl_3$ (10.4 mg, 0.05 mmol, 5 mol%) in place of **RuAqua**. Quantitative ^{1}H NMR using 1,3,5-trimethoxybenzene as an internal standard indicated 58% conversion to product **34a**.

c) Reaction set up in an argon filled glovebox: Reaction performed according to general procedure K using 1-(4-(trifluoromethyl)phenyl)ethan-1-one **A3** (188 mg, 1 mmol, 1 equiv.) as a substrate and **1** (15.3 mg, 0.05 mmol, 5 mol% with respect to Ru) in place of **RuAqua**. Quantitative ^{1}H NMR using 1,3,5-trimethoxybenzene as an internal standard indicated 47% conversion to product **44**.

^{1}H NMR: (500 MHz, $CDCl_3$)

7.61 (d, *J* = 7.9 Hz, 2H), 7.52 – 7.46 (m, 2H), 4.96 (q, *J* = 6.5 Hz, 1H), 1.96 (s, 1H), 1.50 (d, *J* = 6.5 Hz, 3H).

^{13}C NMR: (126 MHz, $CDCl_3$)

149.8 (d, *J* = 1.4 Hz), 129.8 (q, *J* = 32.3 Hz), 125.8, 125.8–125.5 (m), 123.2, 70.0, 25.6.

IR: V_{max} (neat/cm^{-1})

3346 (br), 2979, 1323, 1162, 1065, 1014, 839, 737, 698.

Spectroscopic data matched those previously reported[37].

General Procedure L: Validation of RuAqua Catalysed Hydroboration

Reducible substrate (1 mmol) + HBPin **42** (2 equiv.) → (**RuAqua** (5 mol%), neat, rt, 24 h) reduced product **45** – **46**

Reaction set up outside of the glovebox: A 7 mL microwave vial fitted with a magnetic stirrer bead was charged with **RuAqua** (35.4 mg, 0.05 mmol, 5 mol%). The vial was then capped and purged with N_2 for 10 mins. The substrate to be reduced was then added (1 mmol, 1 equiv.) before adding HBPin **42** (2 mmol, 2 equiv., 0.3 mL). The resulting mixture was allowed to stir at room temperature (25 °C) for 24 hours. After this time the reaction mixture was diluted with water (20 mL) and extracted with EtOAc (3 x 20 mL), dried over $MgSO_4$ and concentrated under vacuum. Products were then isolated via flash column chromatography using the indicated conditions.

Synthesis of 4,4,5,5-tetramethyl-2-(2-phenylpropyl)-1,3,2-dioxaborolane 45

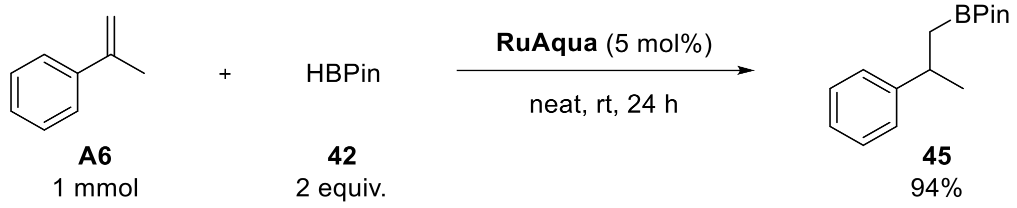

a) Reaction set up outside of the glovebox: Compound **45** was prepared following general procedure L employing α-methyl styrene **A6** (130 μL, 1 mmol, 1 equiv.) as a substrate. After work-up the product was isolated using column chromatography on silica gel eluting with EtOAc in hexane (0–10%) to give the product **45** as a colourless oil (231 mg, 94%).

b) Reaction set up outside of the glovebox: Reaction performed according to general procedure L employing α-methyl styrene **A6** (130 μL, 1 mmol, 1 equiv.) as a substrate and $RuCl_3$ (10.4 mg, 0.05 mmol, 5 mol%) in place of **RuAqua**. Quantitative ^{1}H NMR using 1,3,5-trimethoxybenzene as an internal standard indicated 54% conversion to product **45**.

c) Reaction set up outside of the glovebox: Reaction performed according to general procedure L employing α-methyl styrene **A6** (130 μL, 1 mmol, 1 equiv.) as a substrate and **1** (15.3 mg, 0.05 mmol, 5 mol% with respect to Ru) in place of **RuAqua**. Quantitative ^{1}H NMR using 1,3,5-trimethoxybenzene as an internal standard indicated 78% conversion to product **45**.

^{1}H NMR: (400 MHz, $CDCl_3$)

7.23 – 7.13 (m, 4H), 7.12 – 7.03 (m, 1H), 2.96 (hept, *J* = 7.0 Hz, 1H), 1.20 (d, *J* = 6.9 Hz, 3H), 1.09 (s, 12H).

^{11}B NMR: (128 MHz, $CDCl_3$)

33.9.

^{13}C NMR: (101 MHz, $CDCl_3$)

149.4, 128.3, 126.8, 125.8, 83.1, 35.9, 25.0, 24.9, 24.8.

IR: V_{max} (neat/cm^{-1})

3061, 3027, 2977, 2926, 1365, 1319, 1143, 698.

Spectroscopic data matched those previously reported[46].

Synthesis of 1,2,3,4-tetrahydroquinoline 46

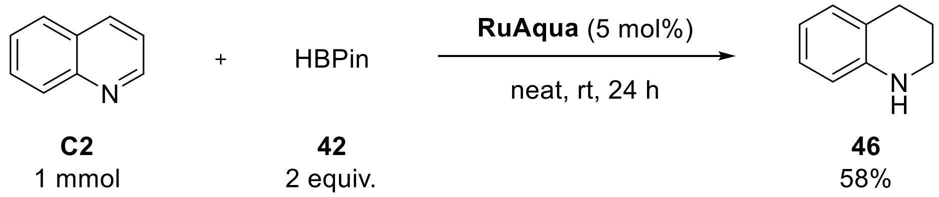

a) Reaction set up outside of the glovebox: Compound **46** was prepared following general procedure L employing quinoline **C2** (118 μL, 1 mmol, 1 equiv.) as a substrate. After work-up the product was isolated using column chromatography on silica gel eluting with EtOAc in hexane (0–10%) to give the product **46** as a colourless oil (77 mg, 58%).
b) Reaction set up outside of the glovebox: Reaction performed according to general procedure L employing quinoline **C2** (118 μL, 1 mmol, 1 equiv.) as a substrate and $RuCl_3$ (10.4 mg, 0.05 mmol, 5 mol%) in place of **RuAqua**. Quantitative ^{1}H NMR using 1,3,5-trimethoxybenzene as an internal standard indicated 7% conversion to product **46**.
c) Reaction set up outside of the glovebox: Reaction performed according to general procedure L employing quinoline **C2** (118 μL, 1 mmol, 1 equiv.) as a substrate and **1** (15.3 mg, 0.05 mmol, 5 mol% with respect to Ru) in place of **RuAqua**. Quantitative ^{1}H NMR using 1,3,5-trimethoxybenzene as an internal standard indicated 29% conversion to product **46**.

^{1}H NMR: (400 MHz, $CDCl_3$)

7.01 – 6.91 (m, 2H), 6.61 (td, *J* = 7.4, 1.3 Hz, 1H), 6.48 (d, *J* = 7.9 Hz, 1H), 3.71 (bs, 1H), 3.38 – 3.20 (m, 2H), 2.77 (t, *J* = 6.4 Hz, 2H), 2.03 – 1.88 (m, 2H).

^{13}C NMR: (101 MHz, $CDCl_3$)

144.9, 129.6, 126.9, 121.6, 117.1, 114.3, 42.1, 27.1, 22.3.

IR: V_{max} (neat/cm^{-1})

3404, 3051, 2924, 1604, 1495, 1308, 742.

Spectroscopic data matched those previously reported[47].

Synthesis of phenylmethanol 44

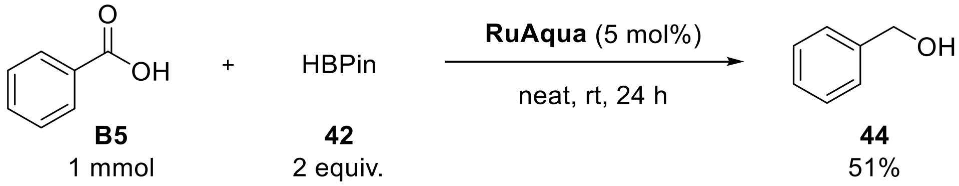

a) Reaction set up outside of the glovebox: Compound **44** was prepared following general procedure L employing benzoic acid **B5** (122 mg, 1 mmol, 1 equiv.) as a substrate adding all solids to the reaction vessel prior to purging. After stirring the reaction mixture for 24 h, NaOH (2 M, 1 mL) was added and the reaction was stirred for a further hour before diluting with water (20 mL) and extracting with EtOAc (3 x 20 mL). The Product was isolated using column chromatography on silica gel eluting with EtOAc in hexane (10–30%) to give the product **44** as a colourless oil (55 mg, 51%).

b) Reaction set up outside of the glovebox: Reaction performed according to general procedure L employing benzoic acid **B5** (122 mg, 1 mmol, 1 equiv.) as a substrate and $RuCl_3$ (10.4 mg, 0.05 mmol, 5 mol%) in place of **RuAqua**. Quantitative ^{1}H NMR using 1,3,5-trimethoxybenzene as an internal standard indicated 30% conversion to product **44**.

c) Reaction set up outside of the glovebox: Reaction performed according to general procedure L employing benzoic acid **B5** (122 mg, 1 mmol, 1 equiv.) as a substrate and **1** (15.3 mg, 0.05 mmol, 5 mol% with respect to Ru) in place of **RuAqua**. Quantitative ^{1}H NMR using 1,3,5-trimethoxybenzene as an internal standard indicated 30% conversion to product **44**.

^{1}H NMR: (500 MHz, $CDCl_3$)

7.37 (d, *J* = 4.4 Hz, 4H), 7.33 – 7.27 (m, 1H), 4.70 (s, 2H), 1.75 (s, 1H).

^{13}C NMR: (126 MHz, $CDCl_3$)

141.0, 128.7, 127.8, 127.1, 65.5.

IR: V_{max} (neat/cm^{-1})

3314 (OH), 3029, 2930, 2872, 1495, 1453, 1207, 1009, 732, 695.

Spectroscopic data matched those previously reported[45].

High Throughput Experimentation for Selection of Photocatalyst via *in situ* Generation

Formation and Evaluation of Photocatalysts Generated from RuAqua

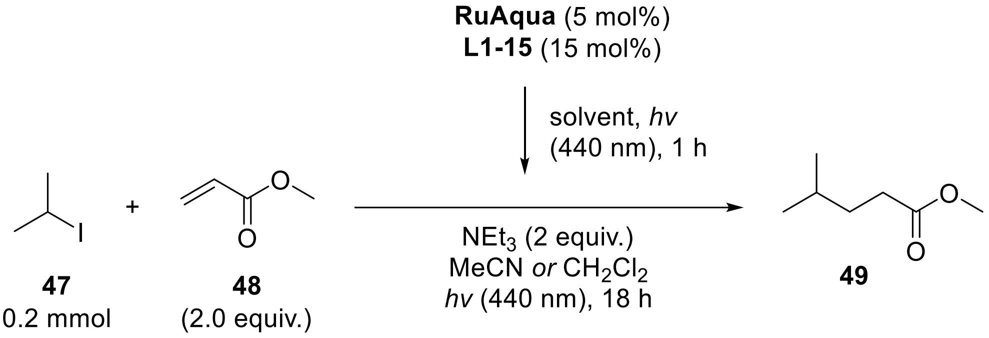

Reaction was adapted from methodology previously reported in the literature[43].

Reaction set up outside of the glovebox: Solvents were degassed prior to use via bubbling with N_2. **L1**–**L15** were commercially available and were used without further purification. To a 7 mL microwave vial equipped with a magnetic stirrer bar was added the indicated ligand (30 μmol, 15 mol%) before purging with N_2 for 2 mins. A stock solution of **RuAqua** (7.1 mg, 10 μmol, 5 mol%) in the indicated solvent (0.25 mL) was added and the reaction was stirred irradiating with blue light (440 nm, Kessil lamp) for 1 hour. After this time a stock solution of 2-iodo-propane **47** (34 mg, 0.2 mmol, 1 equiv.), methyl acrylate **48** (34.4 mg, 0.4 mmol, 2 equiv.), NEt_3 (41 mg, 0.4 mmol, 2 equiv.) and biphenyl (3 mg, 20 μmol, internal standard) in the indicated solvent (1.75 mL) was added and the mixture was stirred irradiating with blue light for a further 18 h. Reaction yields were analysed using GC-FID.

Table 5 Ligands used in HTE screening and corresponding results

Ligands:

no ligand (control)
MeCN:
4 h - 0%
18 h - 0%
DCM:
4 h - 0%
18 h - 0%

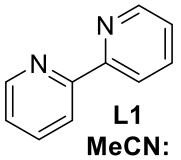

L1
MeCN:
4 h - 9%
18 h - 11%
DCM:
4 h - 7%
18 h - 8%

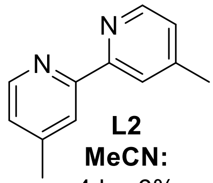

L2
MeCN:
4 h - 6%
18 h - 7%
DCM:
4 h - 4%
18 h - 6%

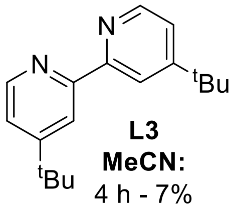

L3
MeCN:
4 h - 7%
18 h - 6 %
DCM:
4 h - 3%
18 h 4%

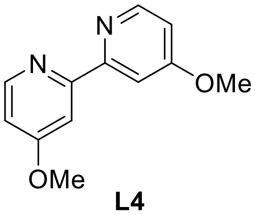

L4
MeCN:
4 h - 0%
18 h - 6%
DCM:
4 h - 1%
18 h - 4%

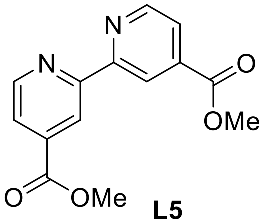

L5
MeCN:
4 h - 2%
18 h - 5%
DCM:
4 h - 1%
18 h - 3%

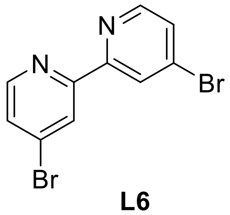

L6
MeCN:
4 h - 5%
18 h - 16%
DCM:
4 h - 4%
18 h - 5%

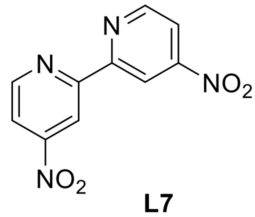

L7
MeCN:
4 h - 0%
18 h - 0%
DCM:
4 h - 0%
18 h - 1%

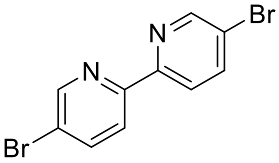

L8
MeCN:
4 h - 6%
18 h - 6%
DCM:
4 h - 1%
18 h - 1%

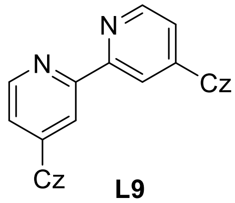

L9
MeCN:
4 h - 1%
18 h - 1%
DCM:
4 h - 1%
18 h - 3%

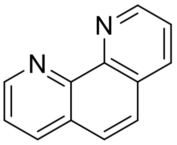

L10
MeCN:
4 h - 14%
18 h - 14%
DCM:
4 h - 11%
18 h - 18%

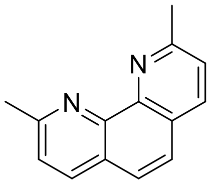

L11
MeCN:
4 h - 0%
18 h - 0%
DCM:
4 h - 0%
18 h - 0%

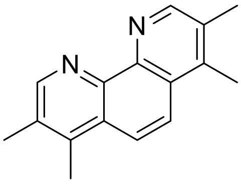

L12
MeCN:
4 h - 0%
18 h - 3%
DCM:
4 h - 0%
18 h - 1%

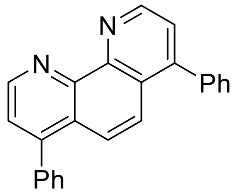

L13
MeCN:
4 h - 17%
18 h - 19%
DCM:
4 h - 9%
18 h - 16%

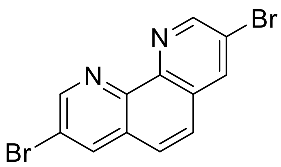

L14
MeCN:
4 h - 3%
18 h - 8%
DCM:
4 h - 3%
18 h - 4%

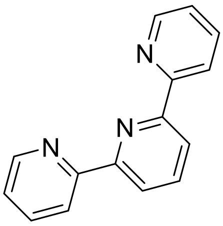

L15
MeCN:
4 h - 0%
18 h - 0%
DCM:
4 h - 0%
18 h - 0%

General Procedure M: Direct Employment of Photocatalysts in Giese 1,4-addition

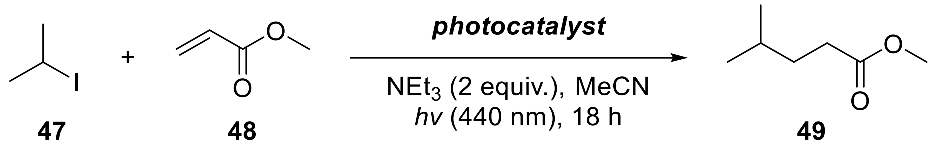

47 0.2 mmol; **48** (2.0 equiv.); **49**

Reaction was adapted from methodology previously reported in the literature[48].

Reaction set up outside of the glovebox: Solvents were degassed prior to use via bubbling with N_2. Photocatalysts were prepared via ball milling (see earlier section). To a 7 mL microwave vial equipped with a magnetic stirrer bar was added the indicated photocatalyst (10 μmol, 5 mol%) before performing 3 x 5 min evac-refill cycles with N_2. A stock solution of 2-iodo-propane **47** (34 mg, 0.2 mmol, 1 equiv.), methyl acrylate **48** (34.4 mg, 0.4 mmol, 2 equiv.), NEt_3 (41 mg, 0.4 mmol, 2 equiv.) and biphenyl (3 mg, 20 μmol, internal standard) in the indicated solvent (2.0 mL) was added and the mixture was stirred irradiating with blue light (Kessil lamp, 440 nm) for 18 h. Reaction yields were analysed using GC-FID.

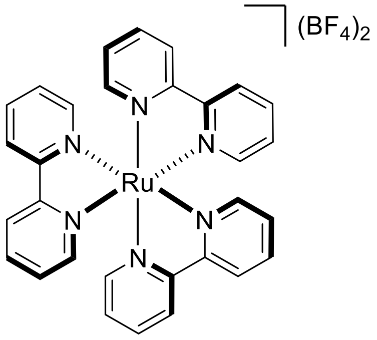

35

a) Reaction set up outside of the glovebox: Product **49** was prepared according to general procedure M using Tris(2,2′-bipyridine)ruthenium(II) tetrafluoroborate **35** as the photocatalyst. After irradiation for 18 h, GC-FID indicated 22% conversion to the desired product **49**.

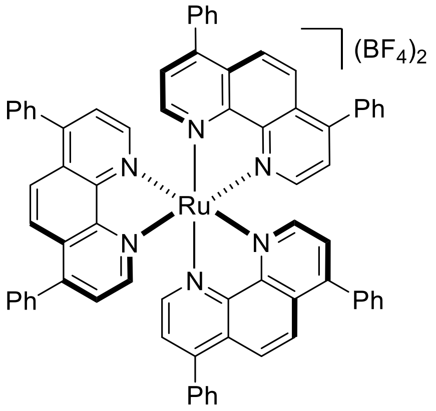

40

b) Reaction set up outside of the glovebox: Product **49** was prepared according to general procedure M using Tris(bathophenanthroline)ruthenium(II) tetrafluoroborate **40** as the photocatalyst. After irradiation for 18 h, GC-FID indicated 85% conversion to the desired product **49**.

NMR Spectra

^{1}H NMR (500 MHz, CD_2Cl_2) of $[Ru(OH_2)(^tBuCN)_5](BF_4)_2$ (RuAqua)

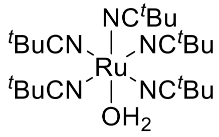

RuAqua

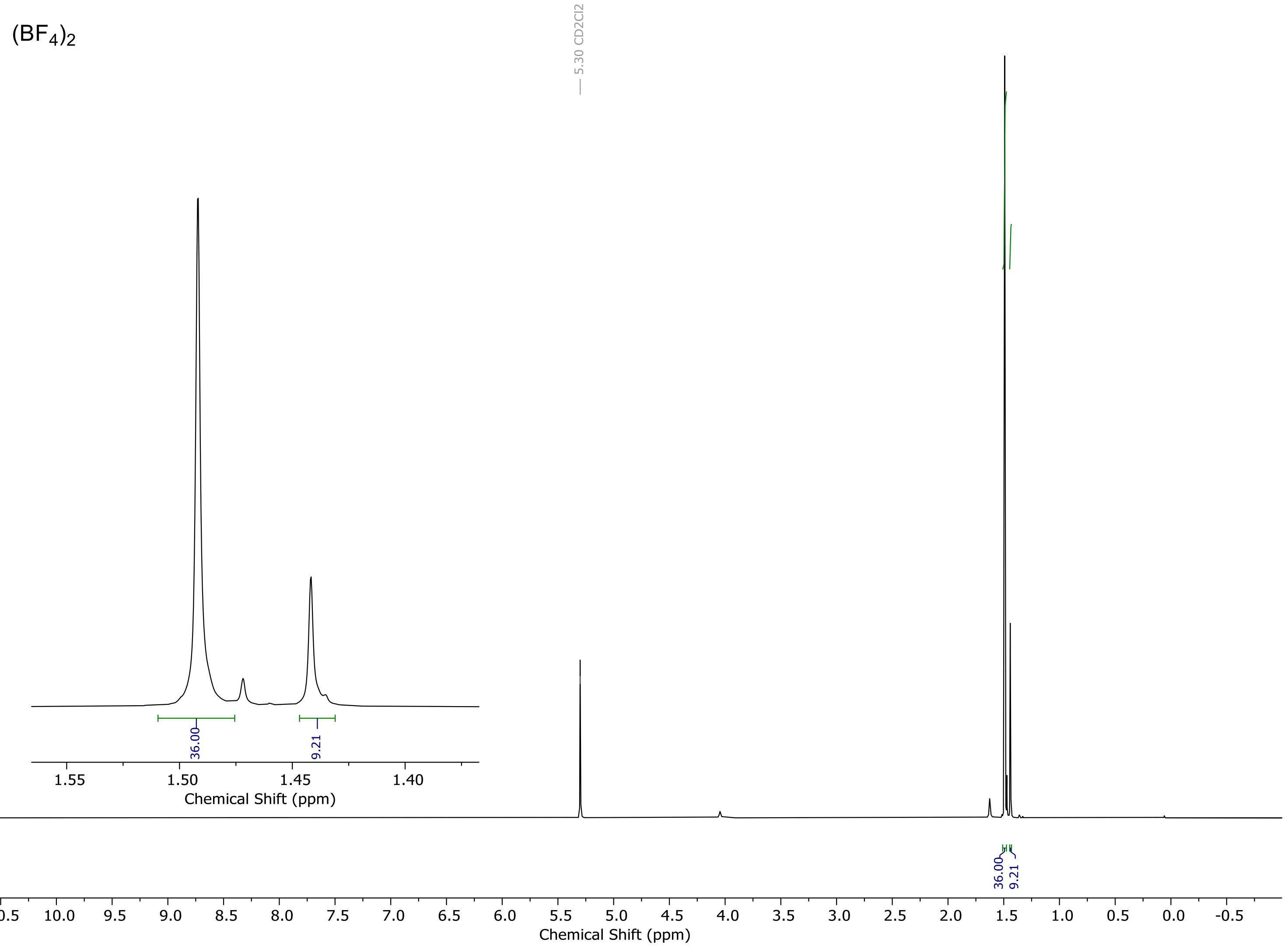

^{13}C NMR (101 MHz, CD_2Cl_2) of $[Ru(OH_2)(^tBuCN)_5](BF_4)_2$ (RuAqua)

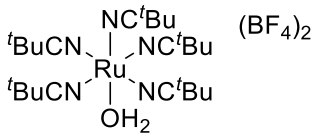

RuAqua

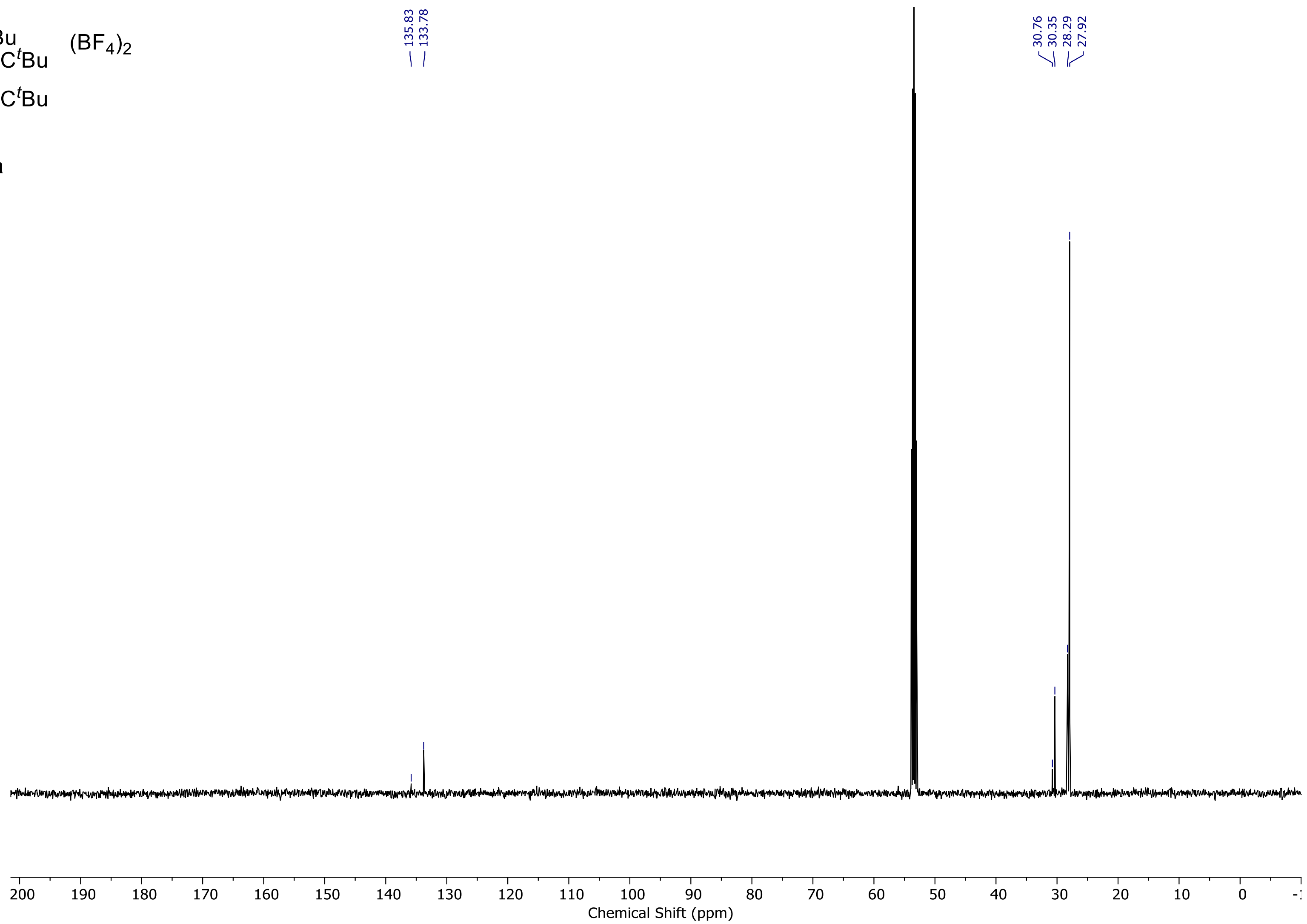

^{19}F NMR (376 MHz, CD_2Cl_2) of [Ru(OH_2)(^{t}BuCN)$_5$](BF_4)$_2$ (RuAqua)

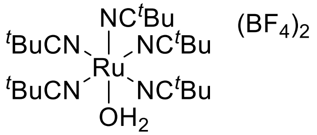

RuAqua

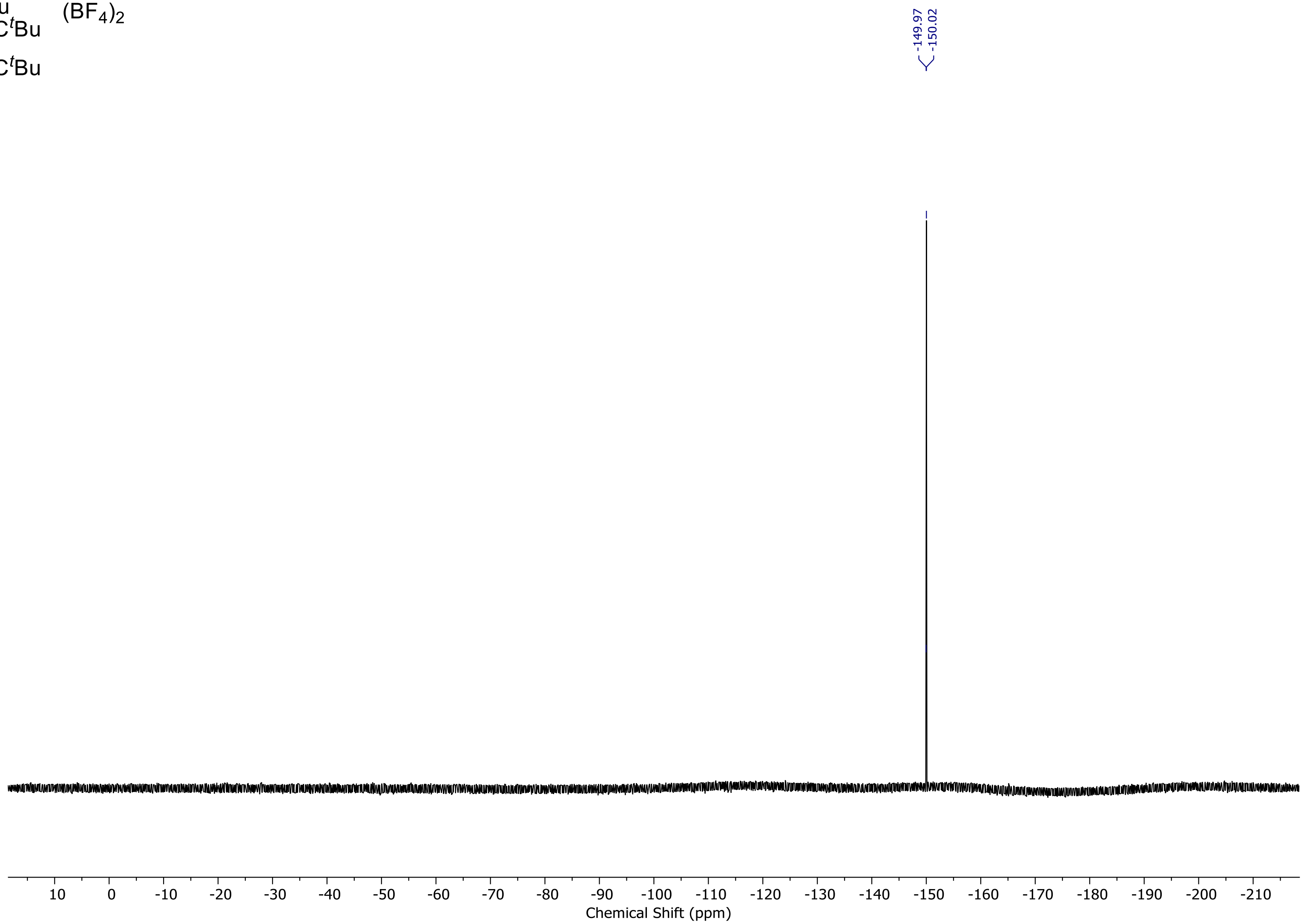

^{1}H NMR (400 MHz, CD_2Cl_2) of RuAqua-OTf

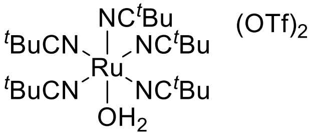

RuAqua-OTf

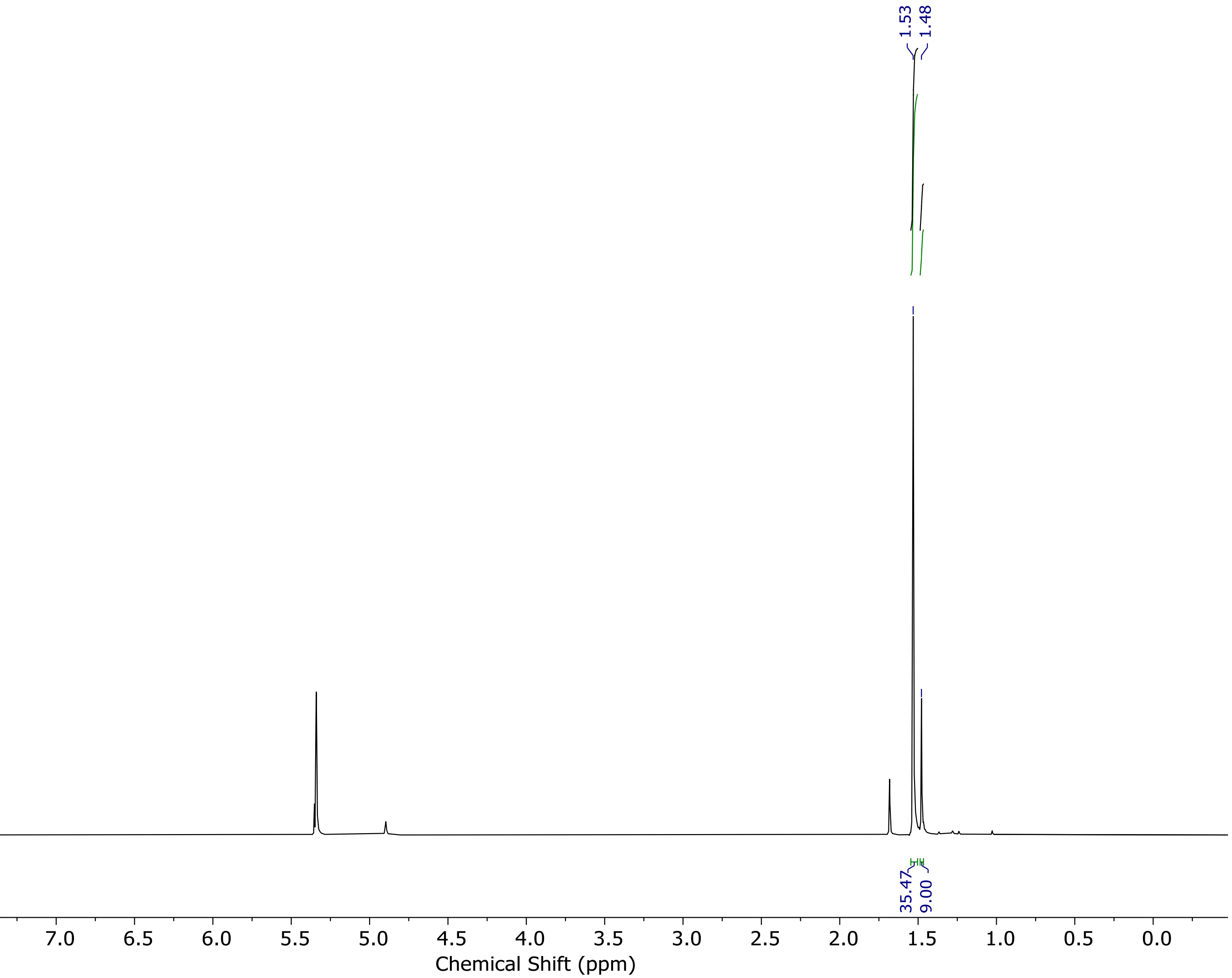

^{13}C NMR (101 MHz, CD_2Cl_2) of RuAqua-OTf

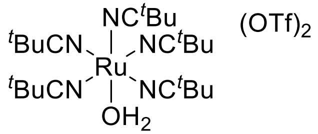

RuAqua-OTf

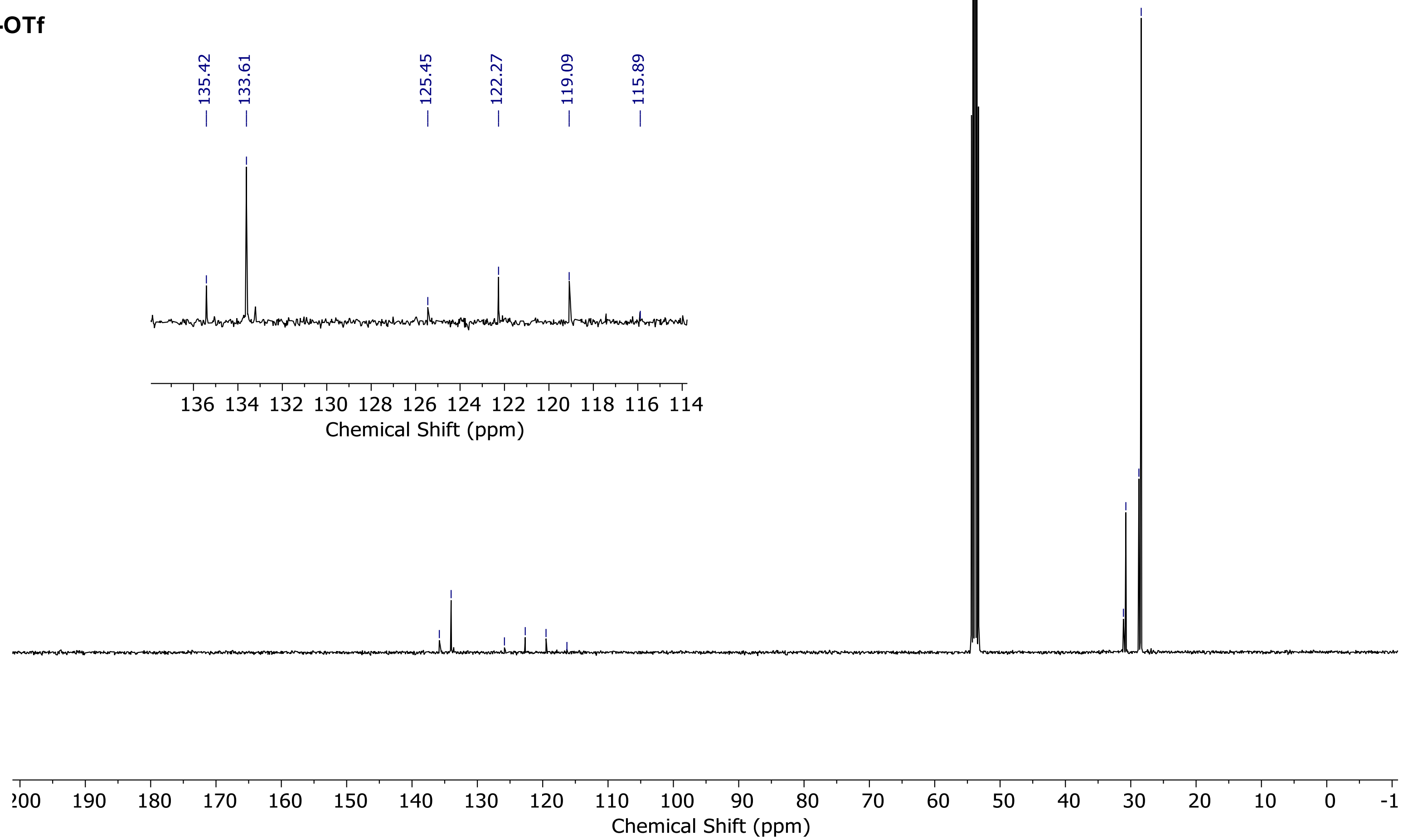

^{19}F NMR (376 MHz, CD_2Cl_2) of RuAqua-OTf

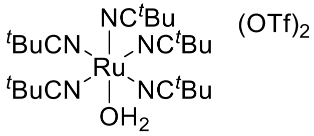

RuAqua-OTf

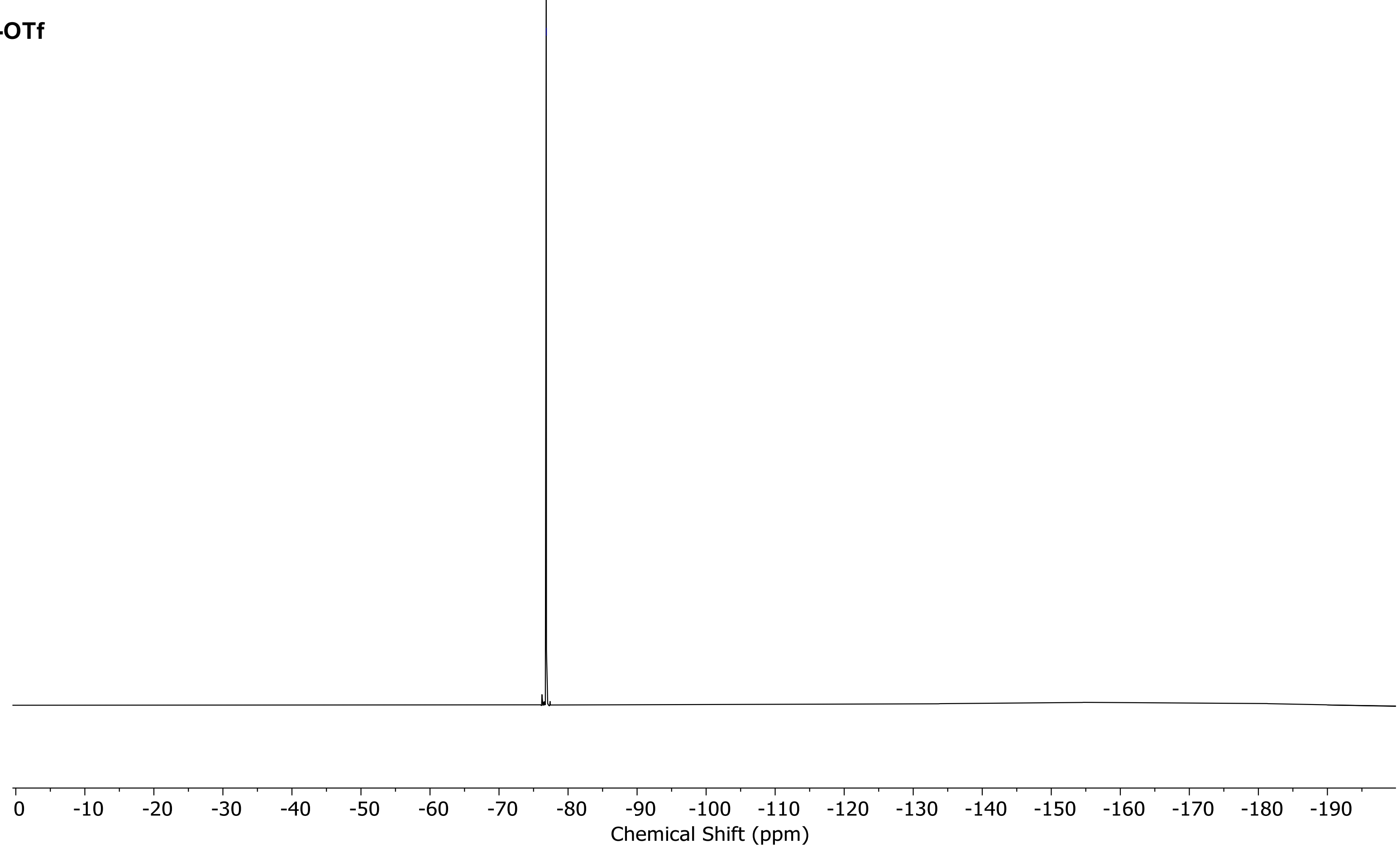

^{1}H NMR (500 MHz, $CDCl_3$) of 2-(4'-methoxy-3-methyl-[1,1'-biphenyl]-2-yl)pyridine 10b

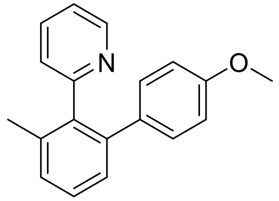

10b

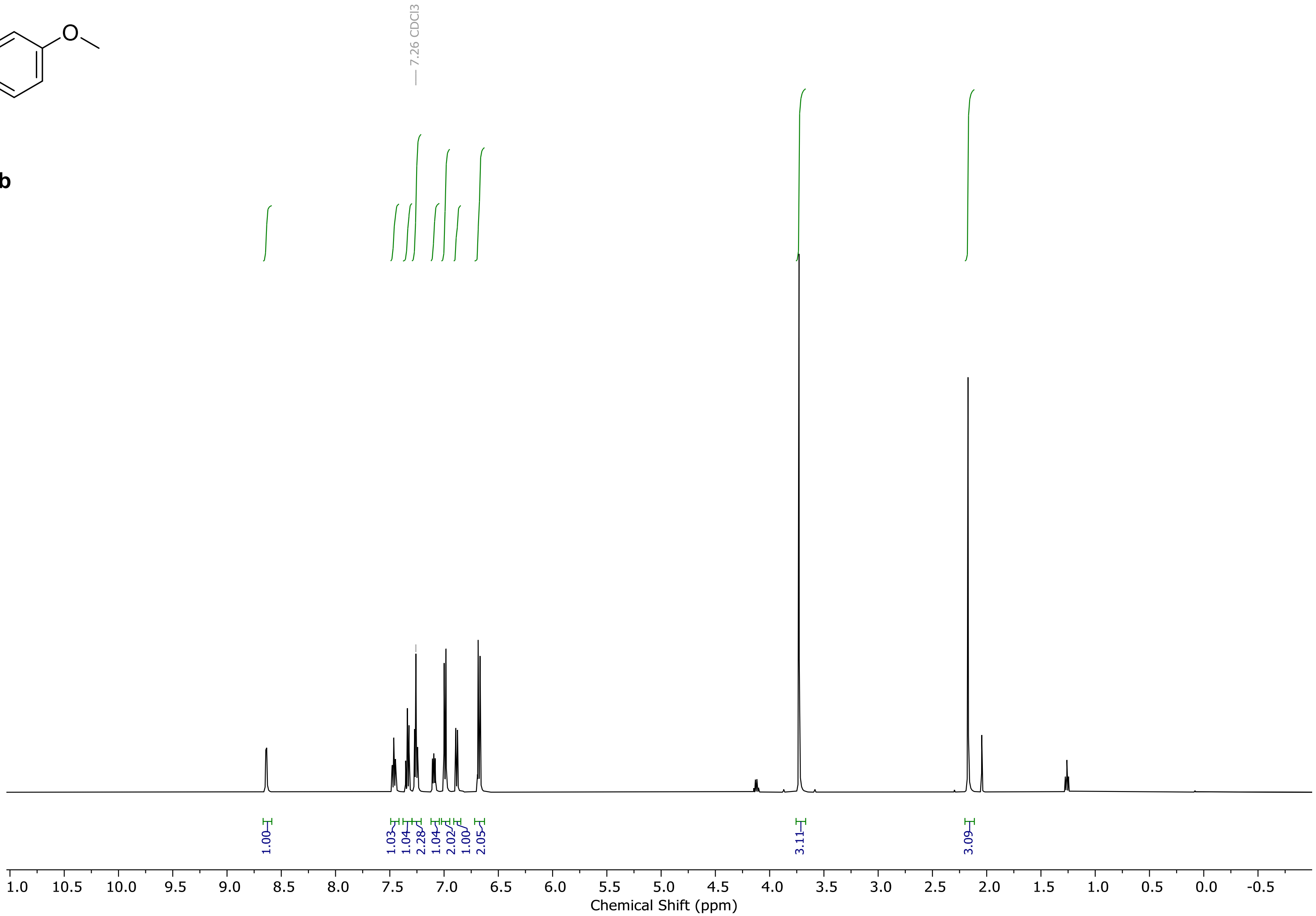

^{13}C NMR (126 MHz, $CDCl_3$) of 2-(4'-methoxy-3-methyl-[1,1'-biphenyl]-2-yl)pyridine 10b

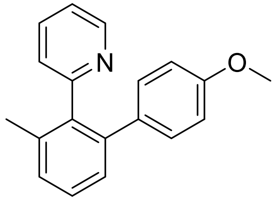

10b

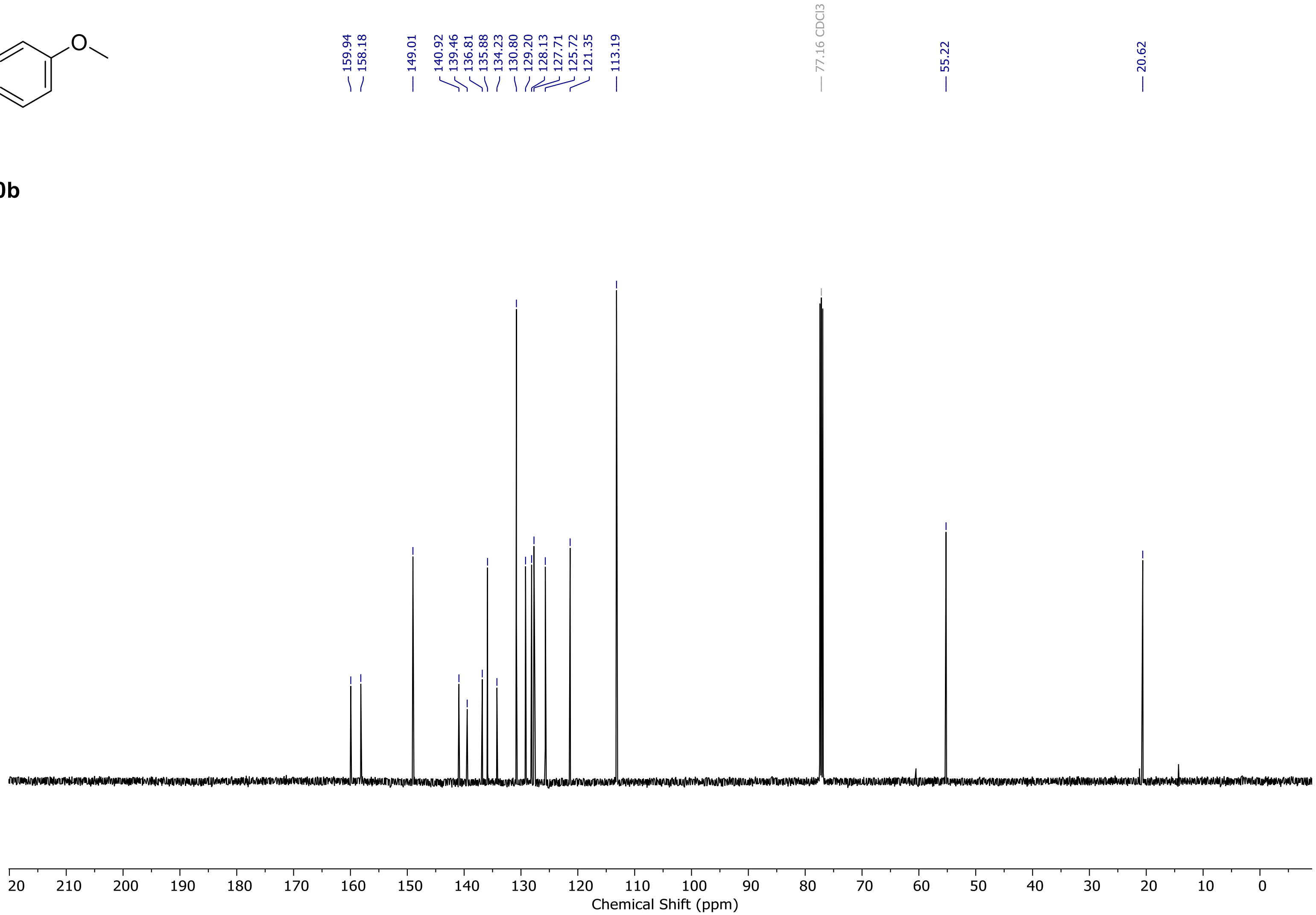

^{1}H NMR (400 MHz, $CDCl_3$) of Starting Material 8a

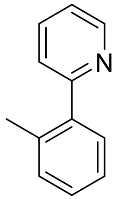

8a

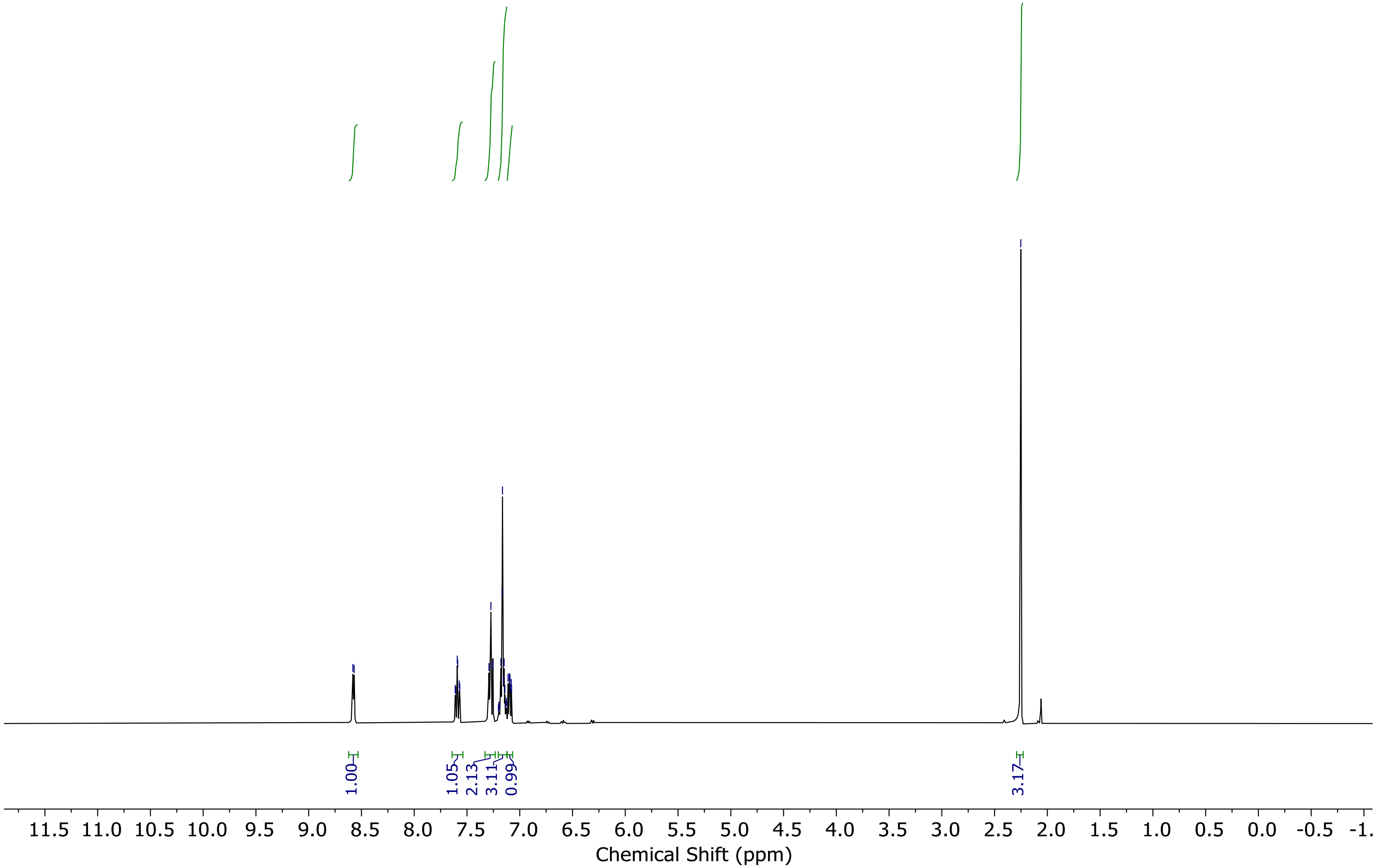

^{13}C NMR (126 MHz, $CDCl_3$) of Starting Material 8a

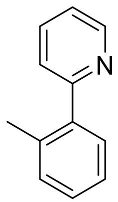

8a

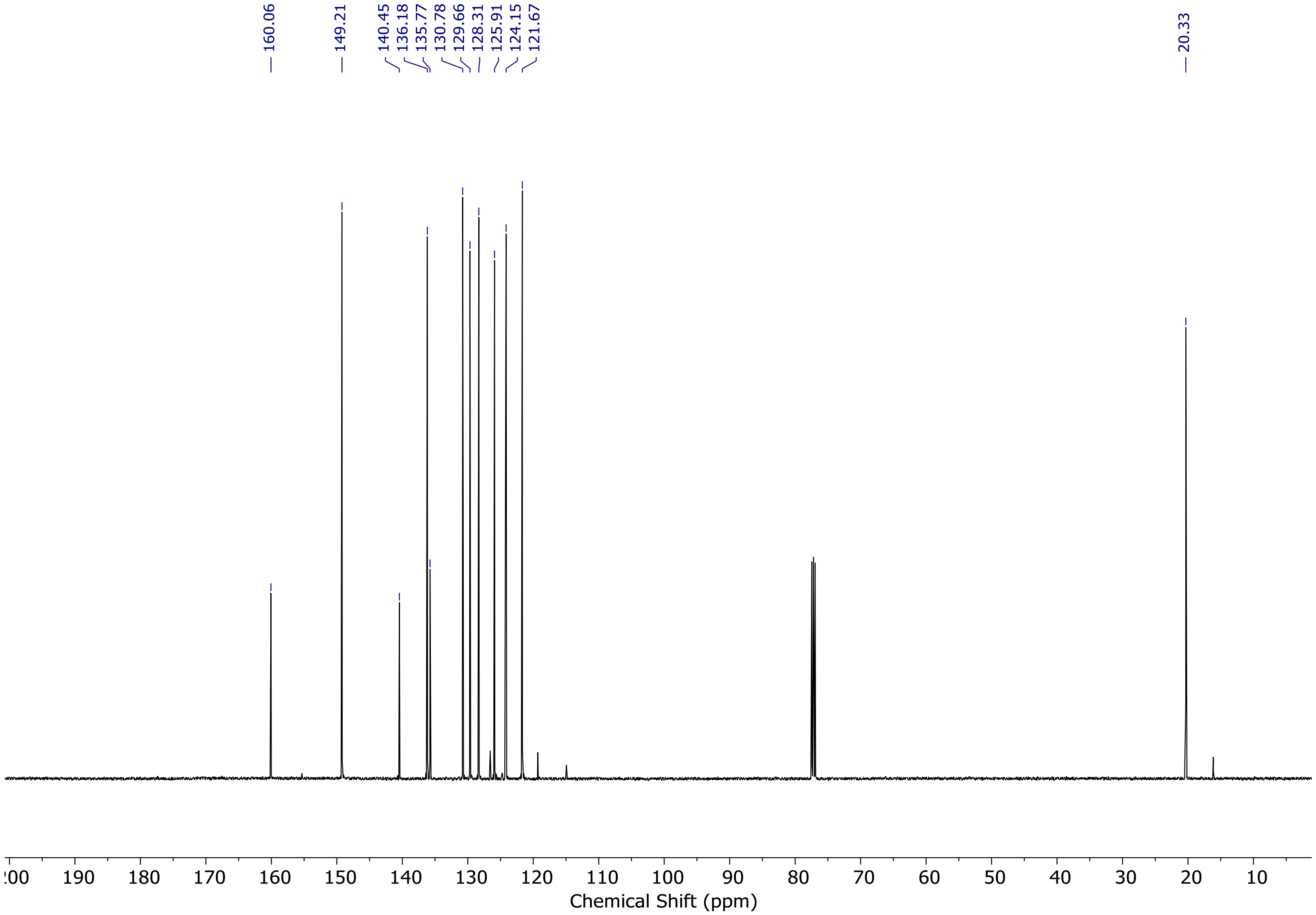

^{1}H NMR (500 MHz, $CDCl_3$) of Starting Material 8c

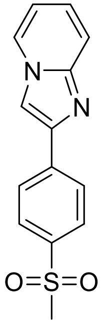

8c

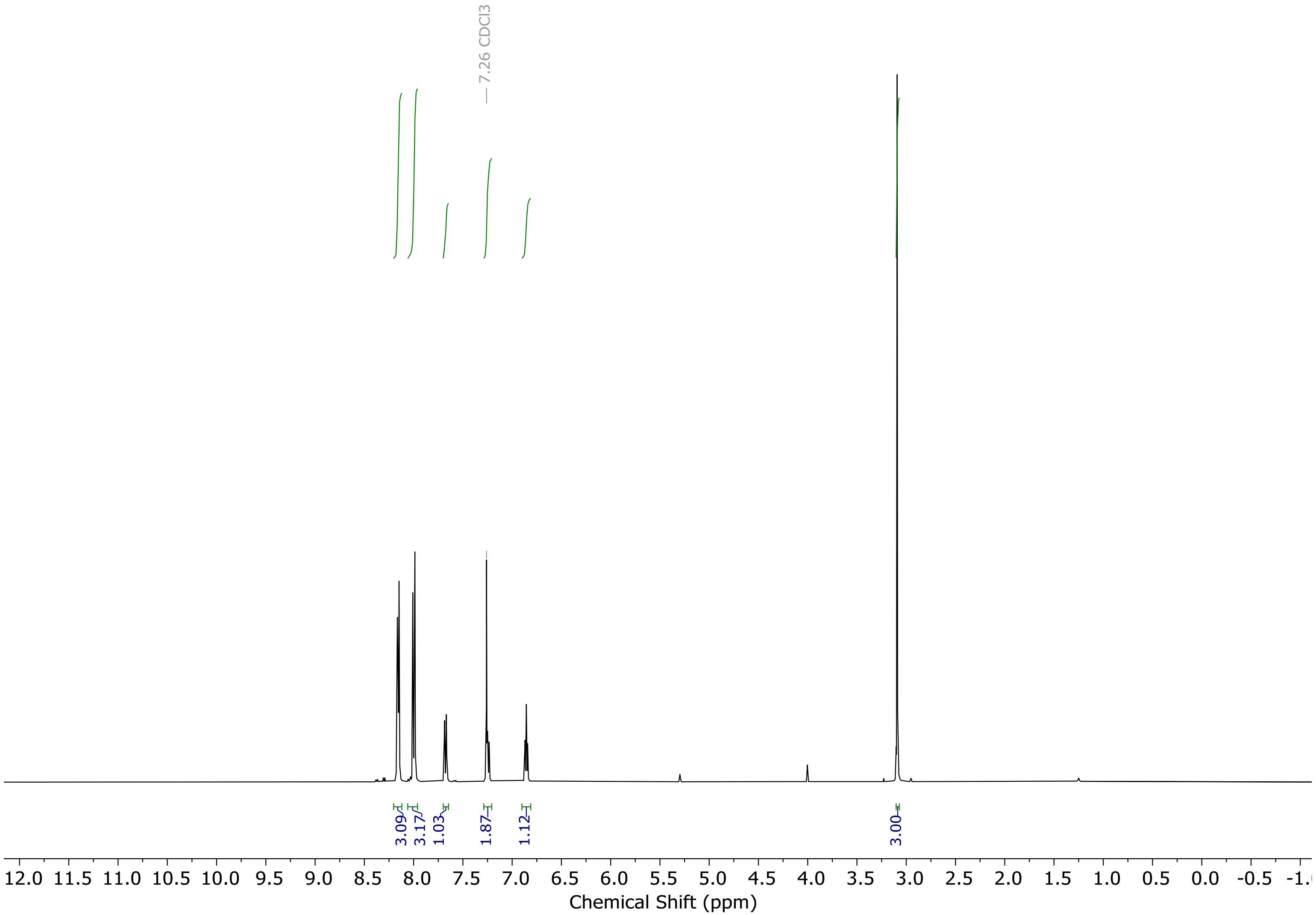

^{13}C NMR (126 MHz, $CDCl_3$) of Starting Material 8c

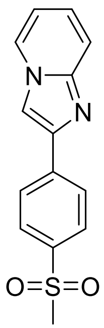

8c

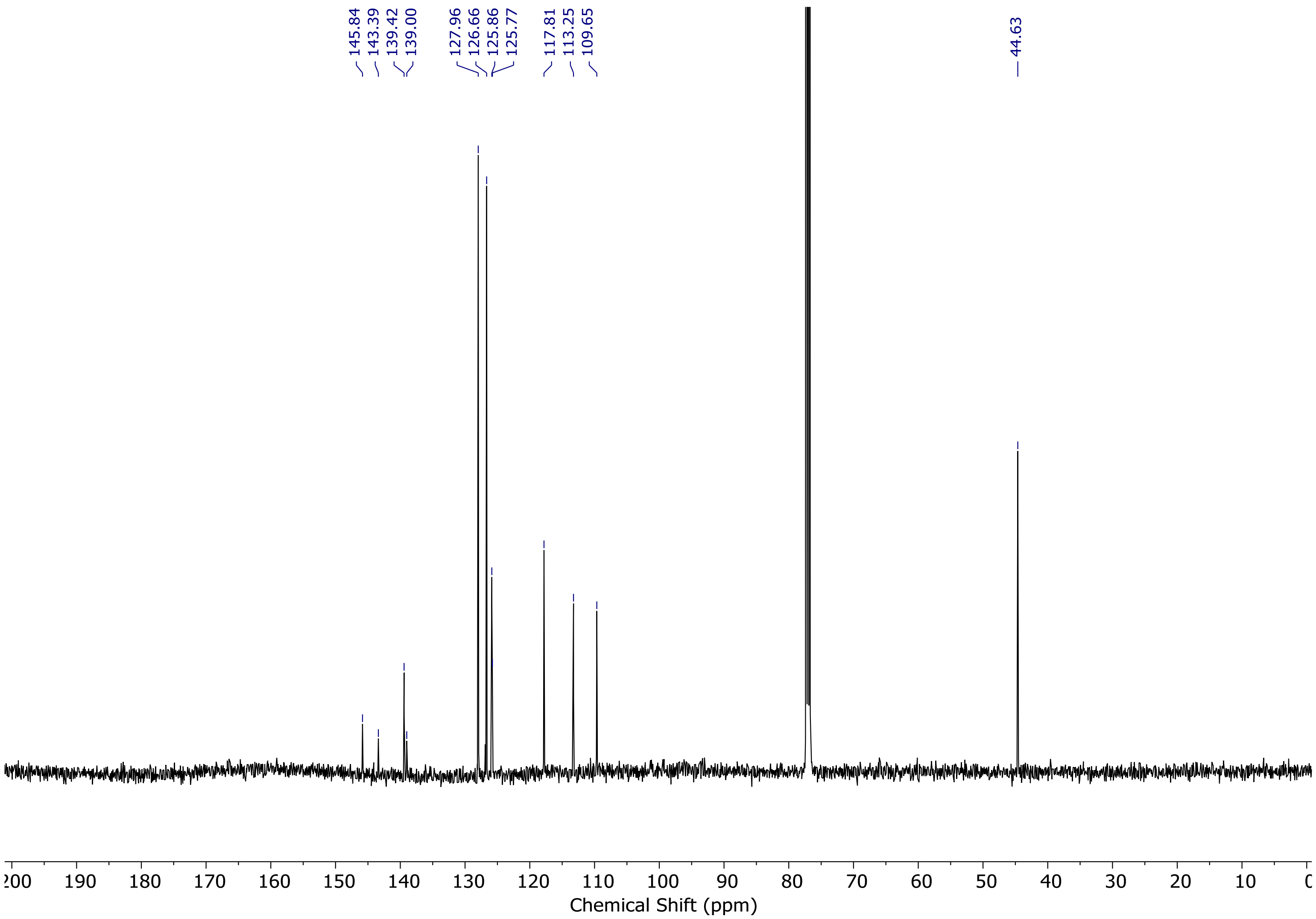

^{1}H NMR (400 MHz, $CDCl_3$) of Starting Material 8d

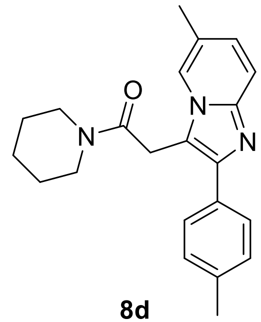

8d

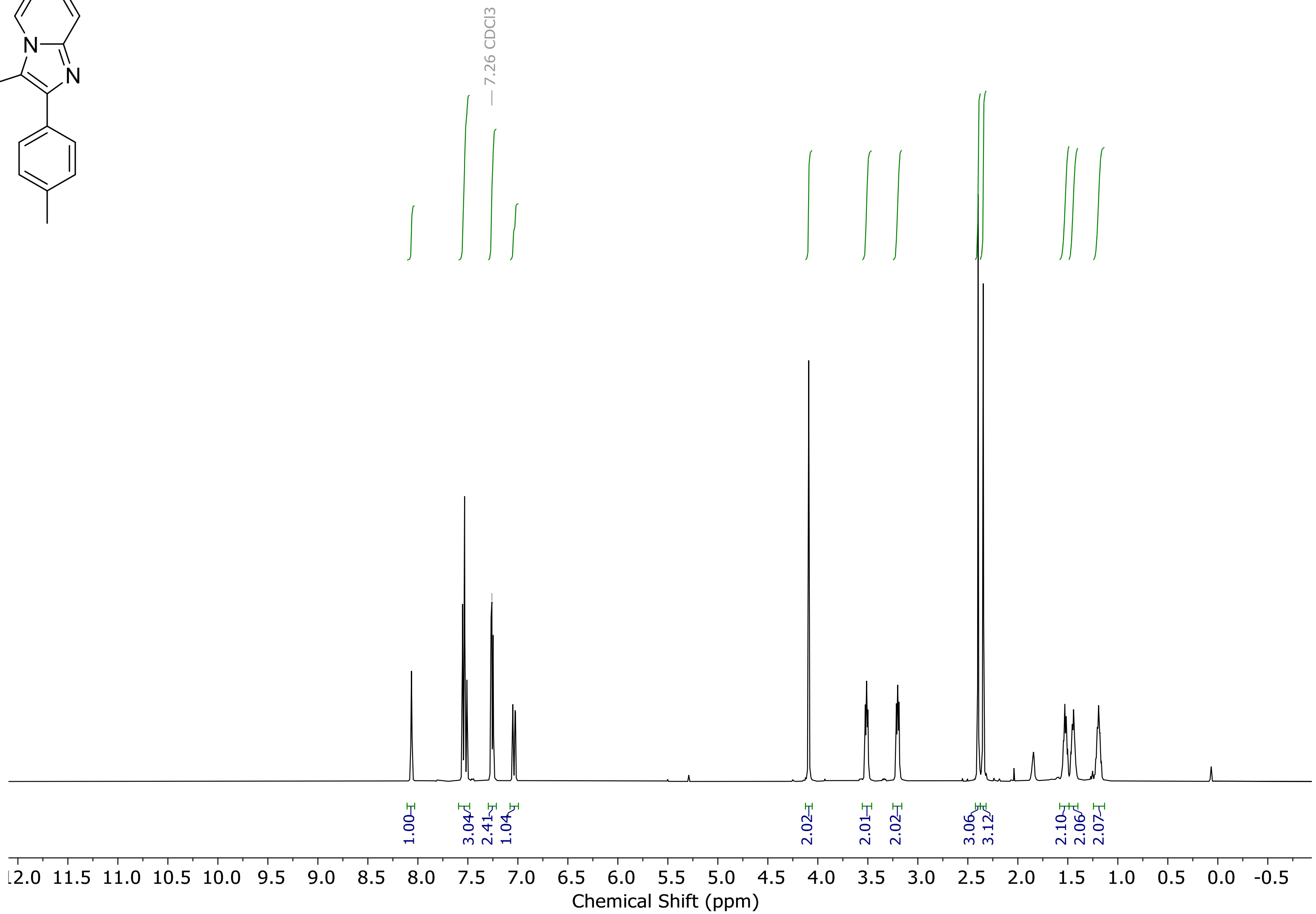

^{13}C NMR (101 MHz, CDCl$_3$) of Starting Material 8d

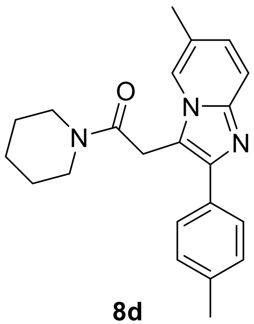

8d

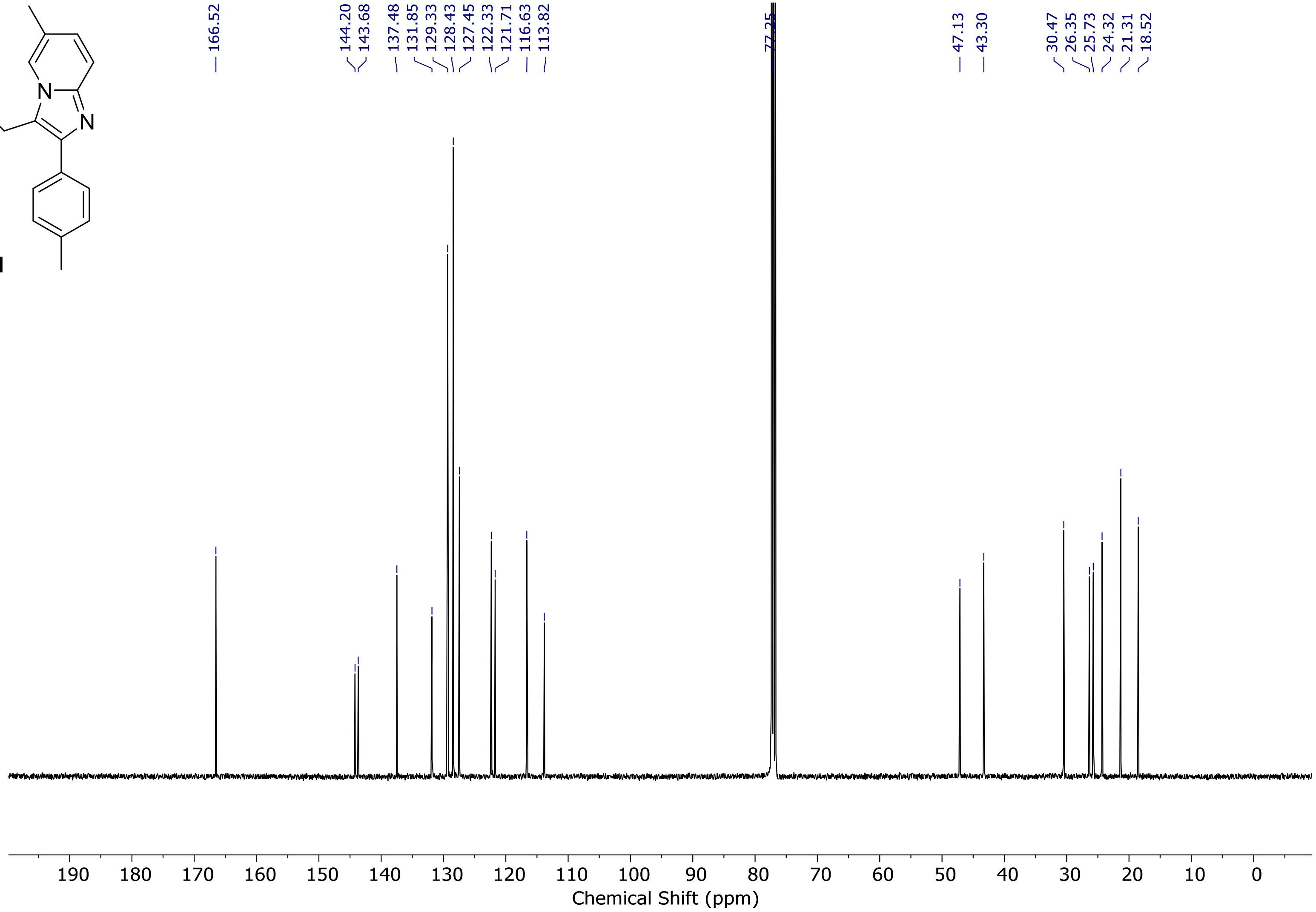

^{1}H NMR (400 MHz, CDCl$_3$) of Starting Material 9l

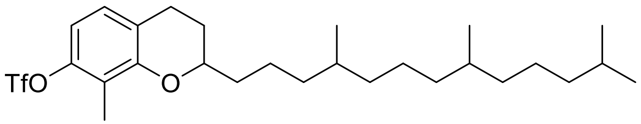

9l

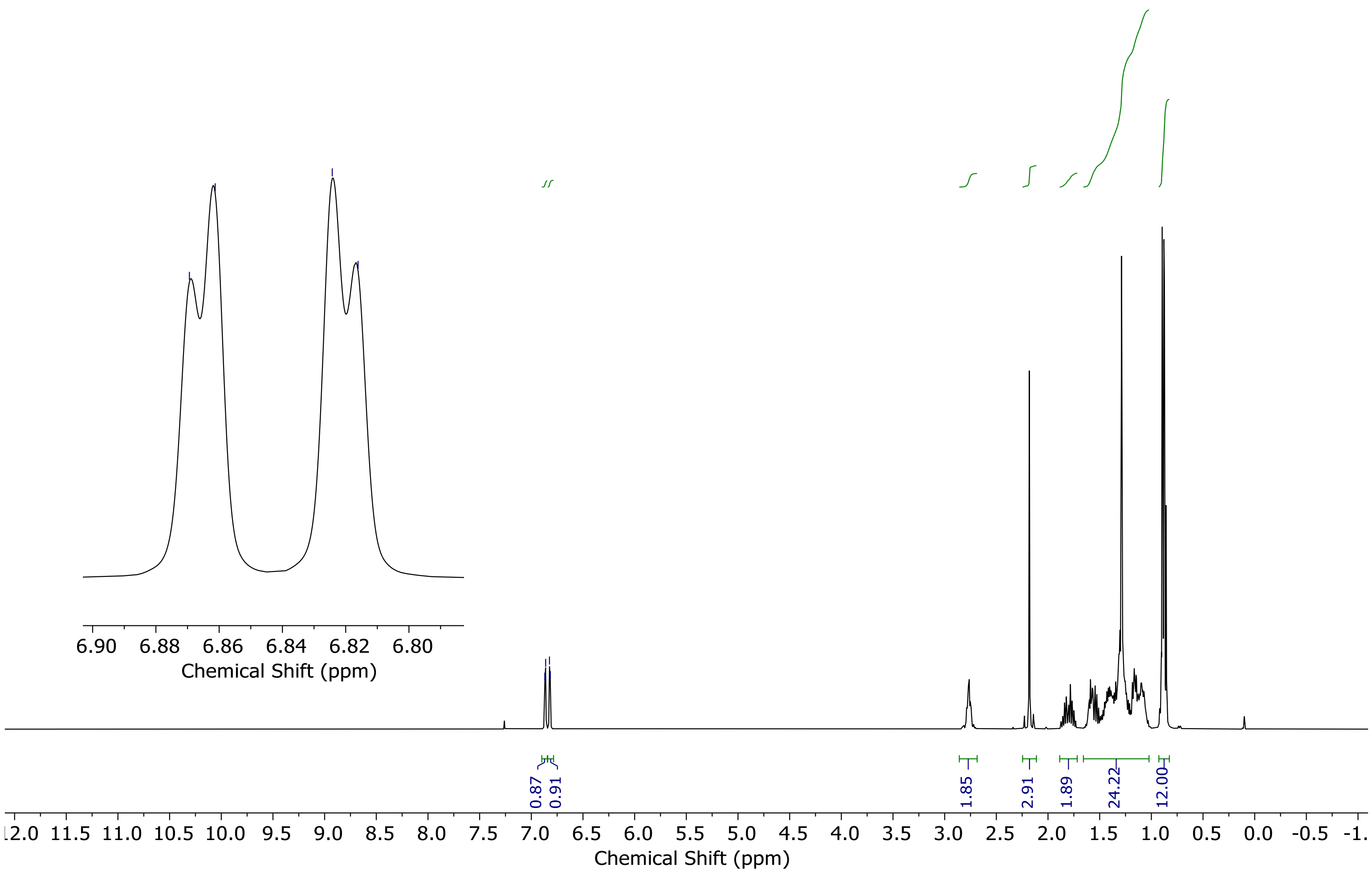

^{13}C NMR (101 MHz, $CDCl_3$) of Starting Material 9l

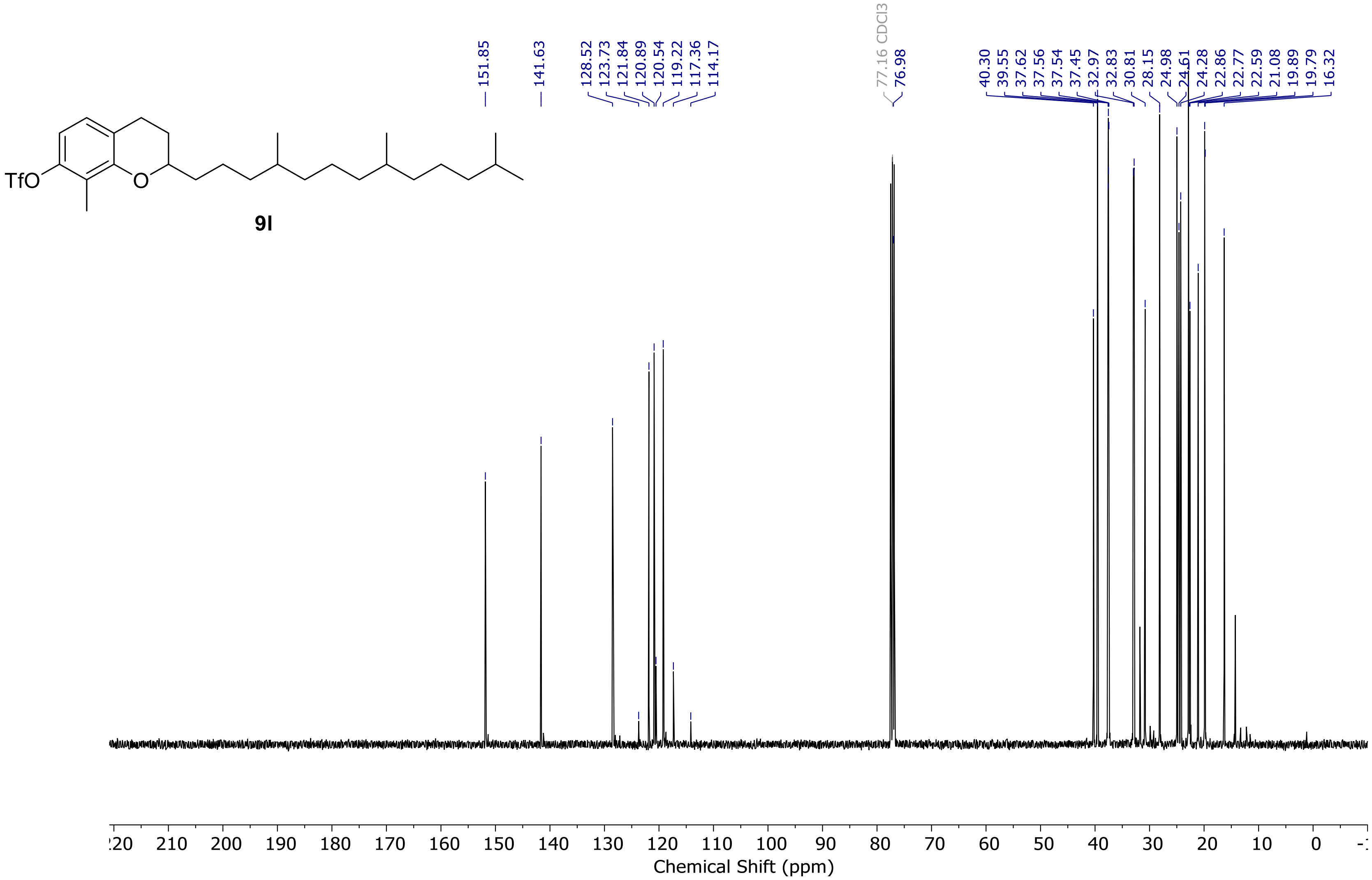

^{19}F NMR (376 MHz, $CDCl_3$) of Starting Material 9l

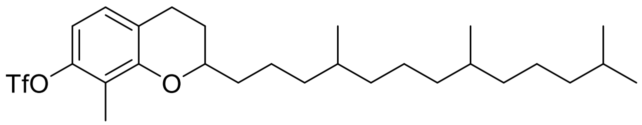

9l

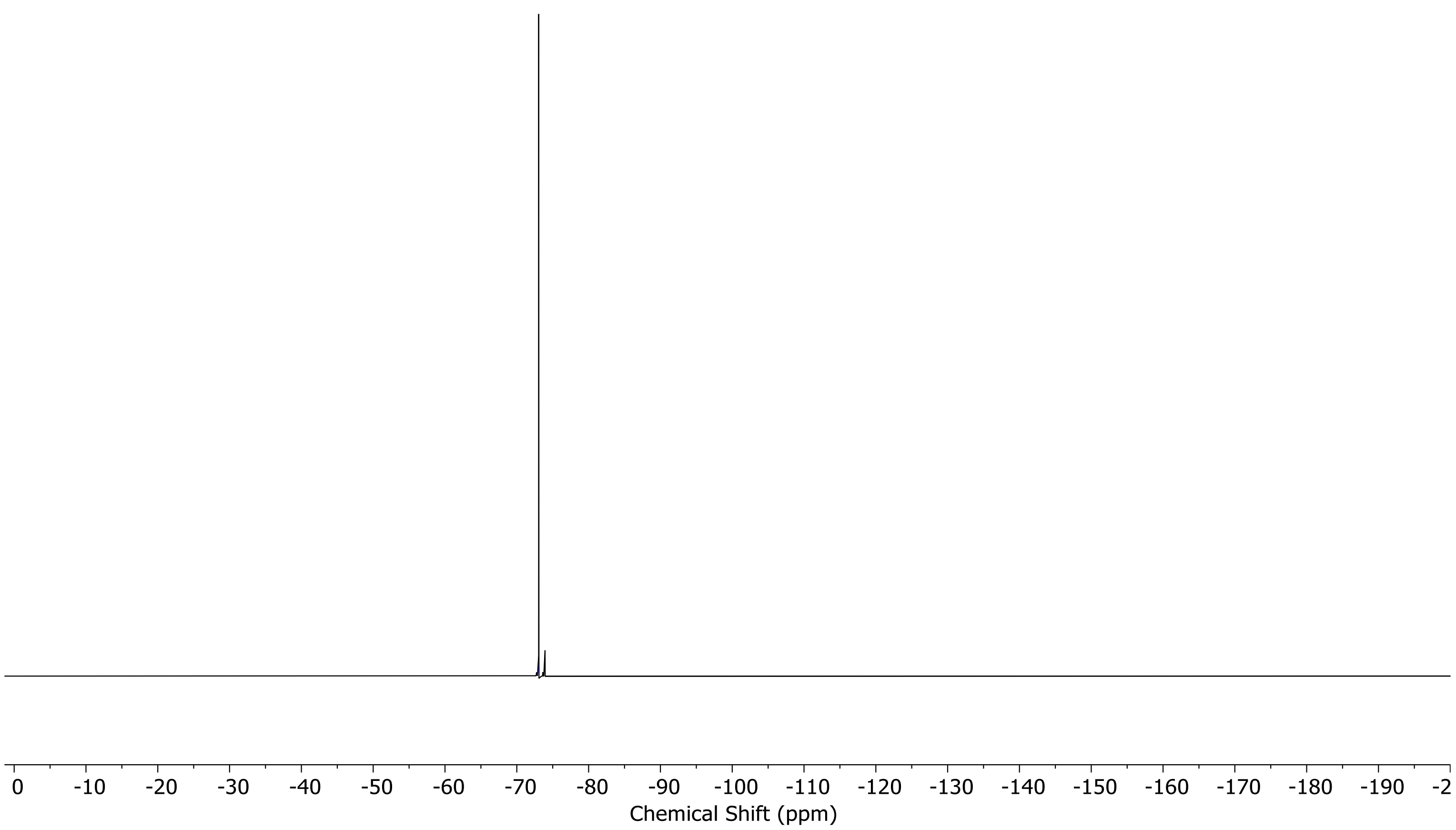

^{1}H NMR (400 MHz, $CDCl_3$) of 2-(3,3',5'-trimethyl-[1,1'-biphenyl]-2-yl)pyridine 10a

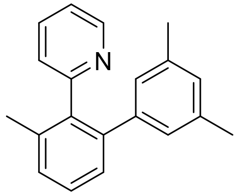

10a

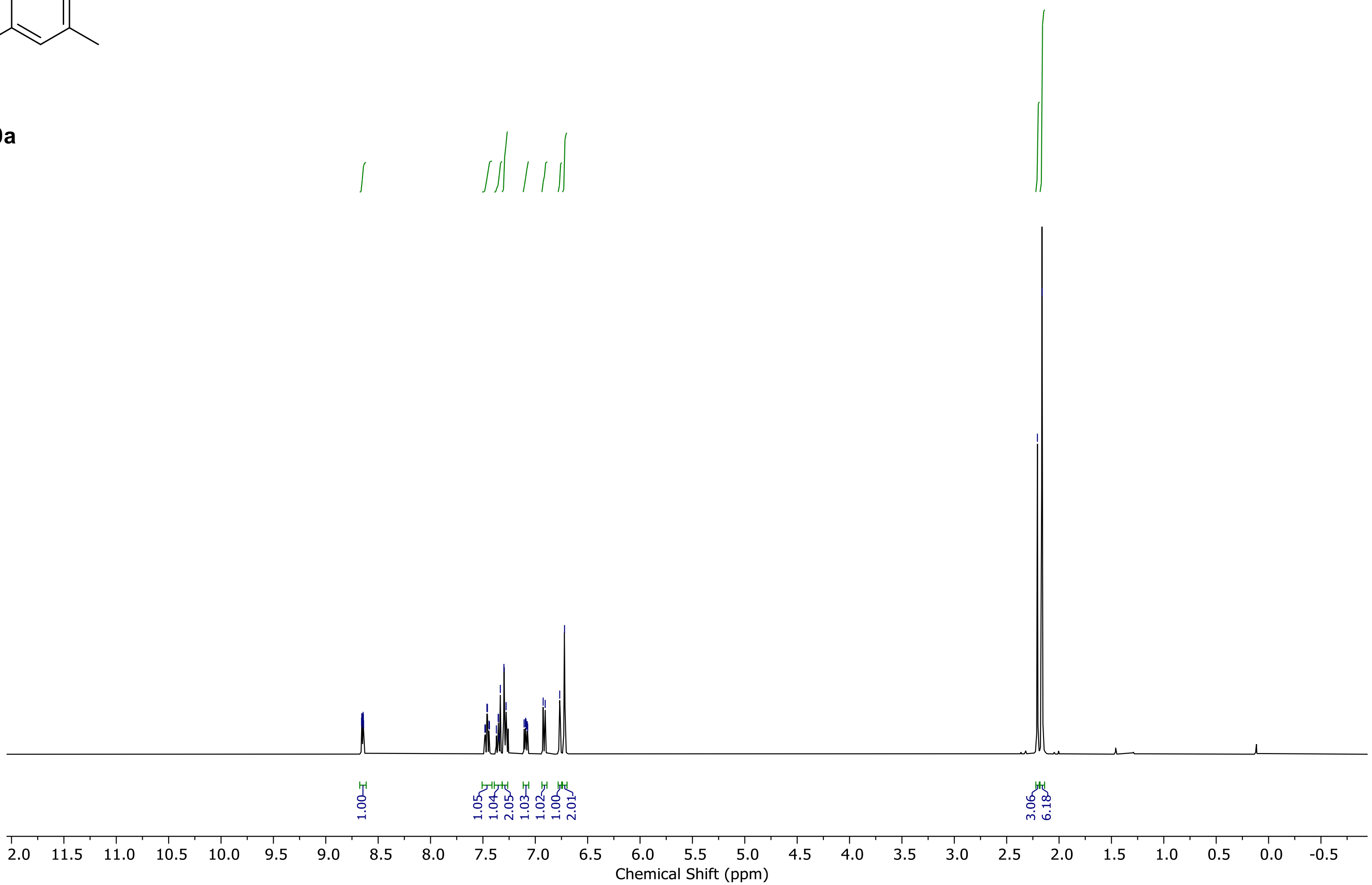

^{13}C NMR (101 MHz, $CDCl_3$) of 2-(3,3',5'-trimethyl-[1,1'-biphenyl]-2-yl)pyridine 10a

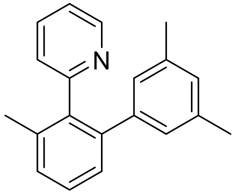

10a

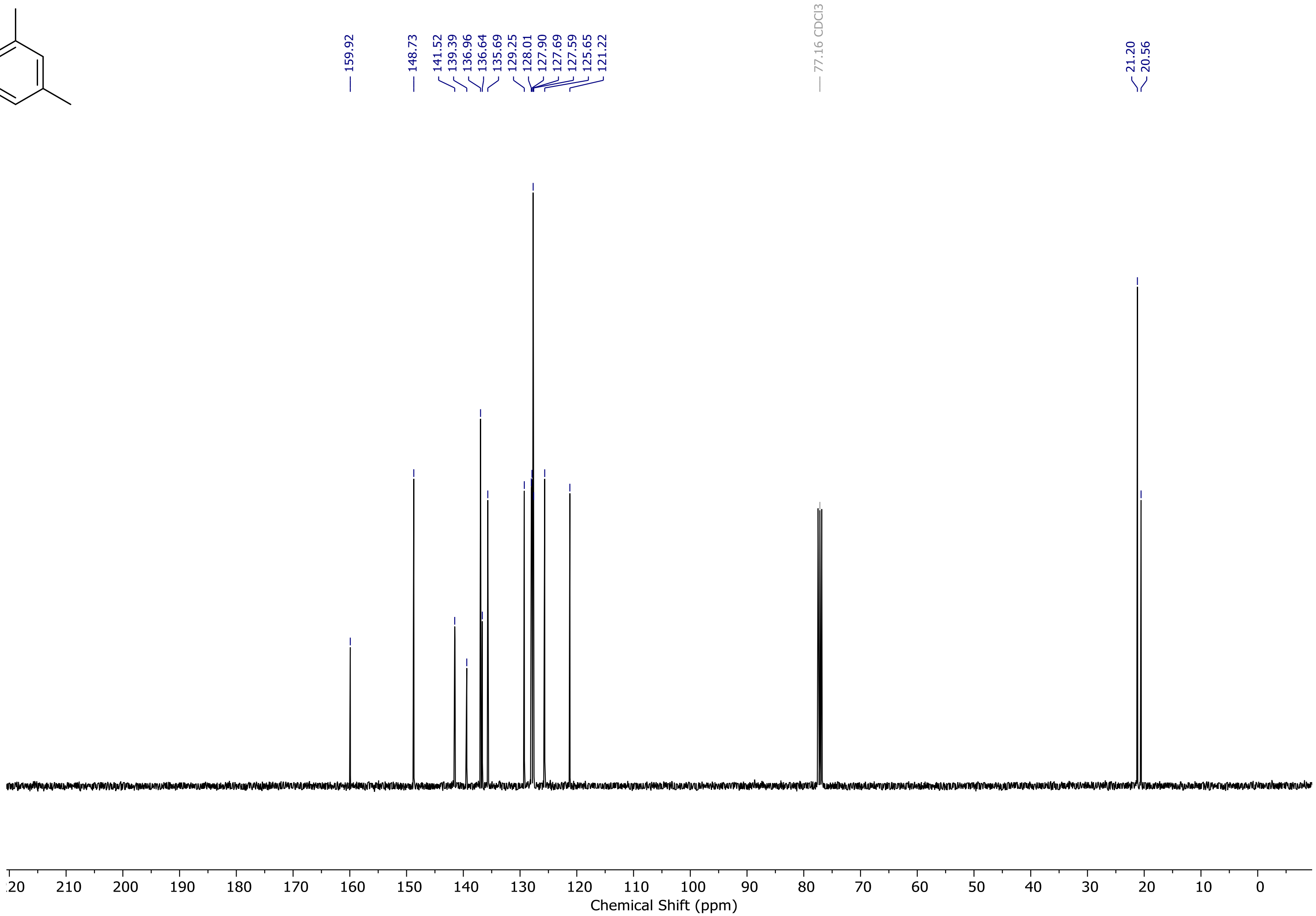

Large Scale Synthesis of 9b at AstraZeneca UHPLC MS (220 nm) spectra of the crude reaction mixture after 22 h.

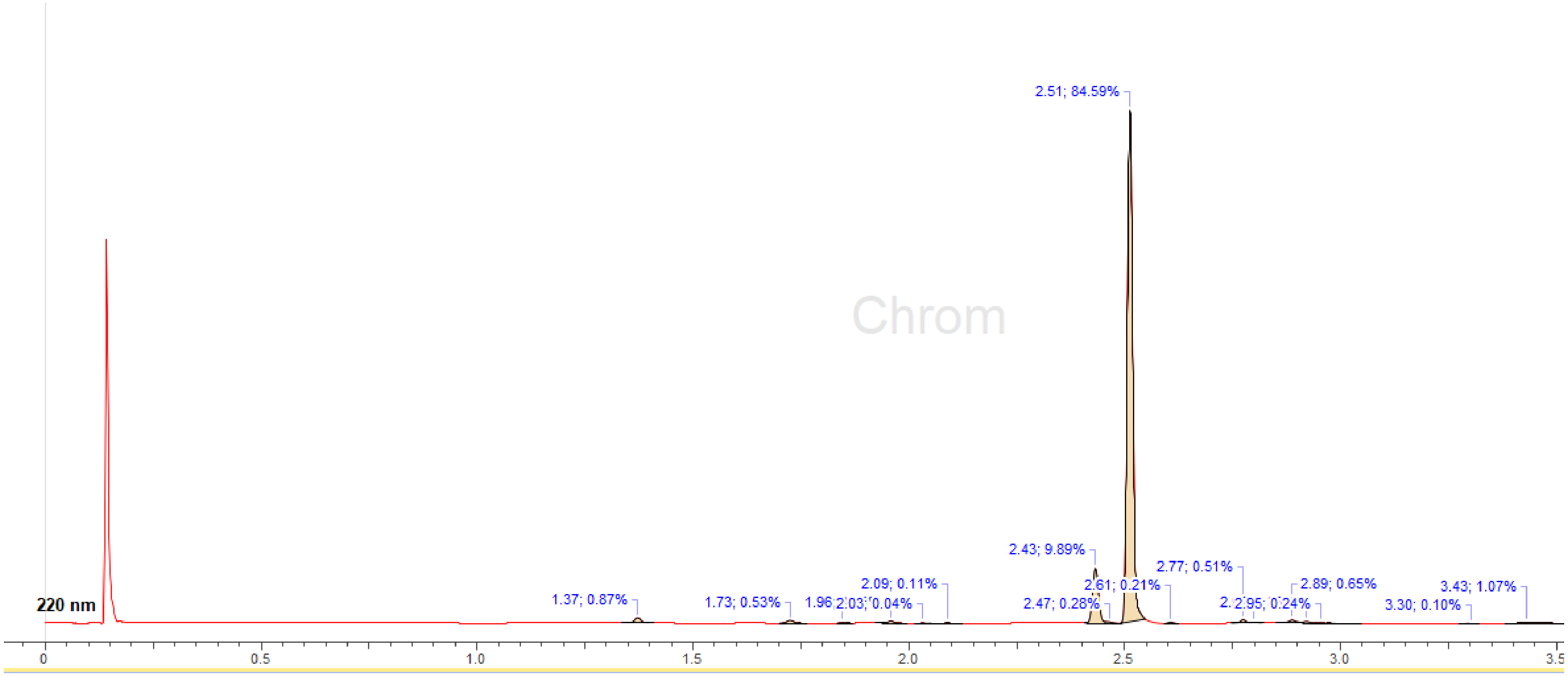

2-(o-tolyl)pyridine (1.96 min, <1 a/a%), 4-iodoanisole (2.43 min, 10 a/a%), 2-(4'-methoxy-3-methyl-[1,1'-biphenyl]-2-yl)pyridine (2.51 min, 85% a/a)

^{1}H NMR spectrum of the crude reaction product 10b for purity calculation.

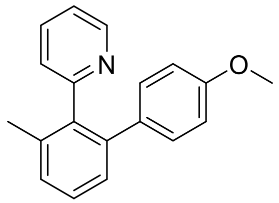

10b

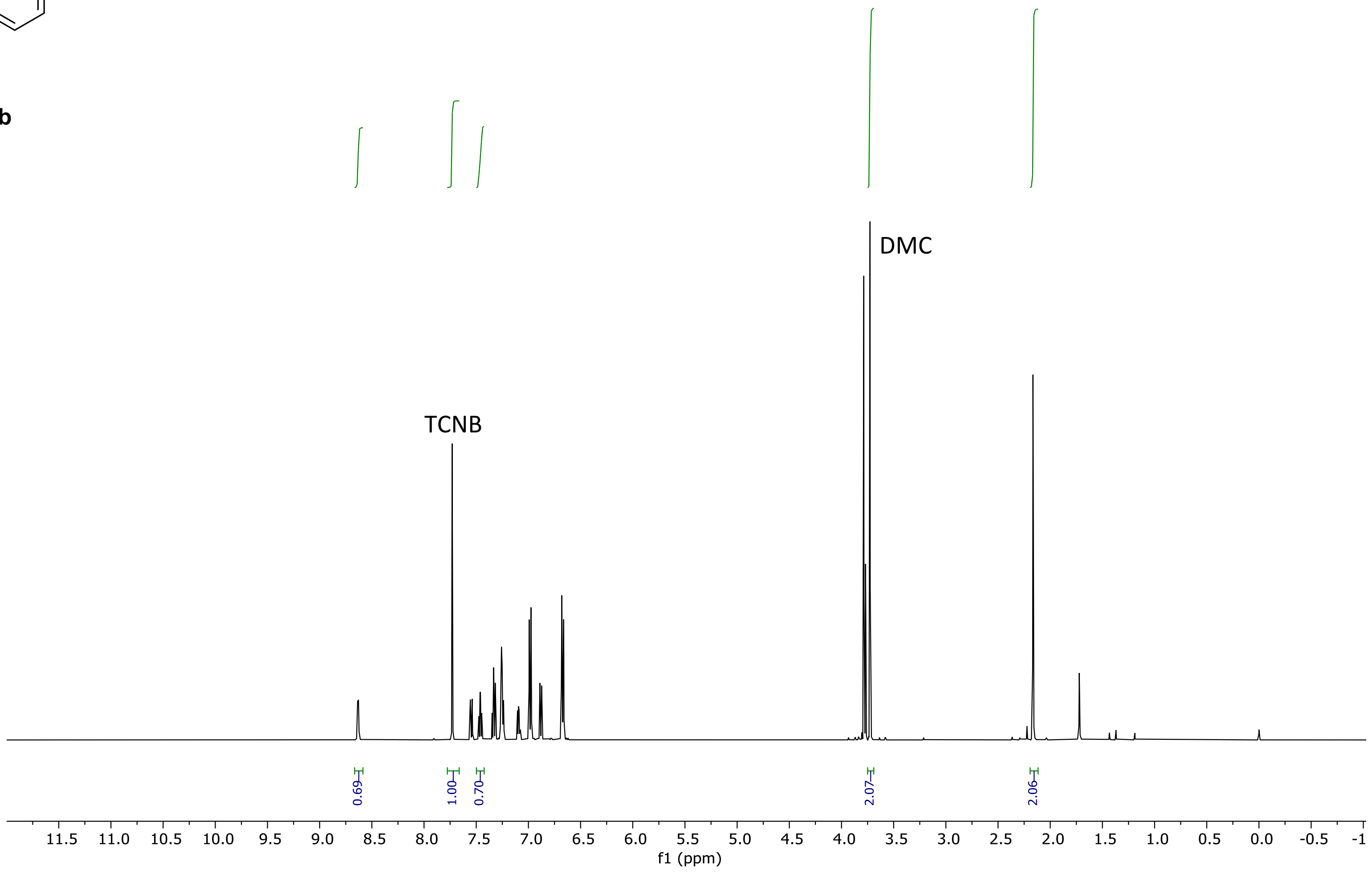

Average Purity = 67.8%

Assuming sample weight: 29.36 mg, and mol weight: 275.34

Using Reference Compound: 2-3-5-6-TCNB (27.29 mg, 99.8% purity, Mol Weight=260.89)

Sample Integral 1: 8.59 - 8.66 ppm, value = 0.69 (1 nuclides) - Purity = 67.5%

Sample Integral 2: 7.42 - 7.5 ppm, value = 0.7 (1 nuclides) - Purity = 69%

Sample Integral 3: 3.69 - 3.75 ppm, value = 2.07 (3 nuclides) - Purity = 67.5%

Sample Integral 4: 2.12 - 2.19 ppm, value = 2.06 (3 nuclides) - Purity = 67.2%

Reference Integral: 7.66 - 7.78 ppm, value = 1 (1 nuclides)

^{1}H NMR (500 MHz, CDCl$_3$) of [2-(*o*-tolyl)pyridine]–[haloperidol] 10c

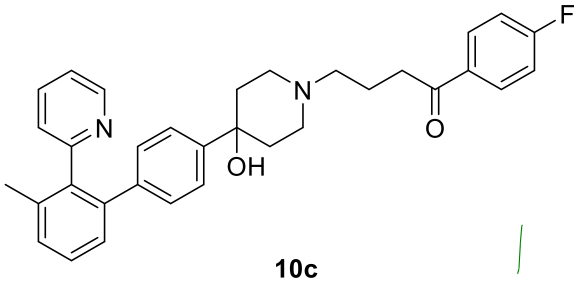

10c

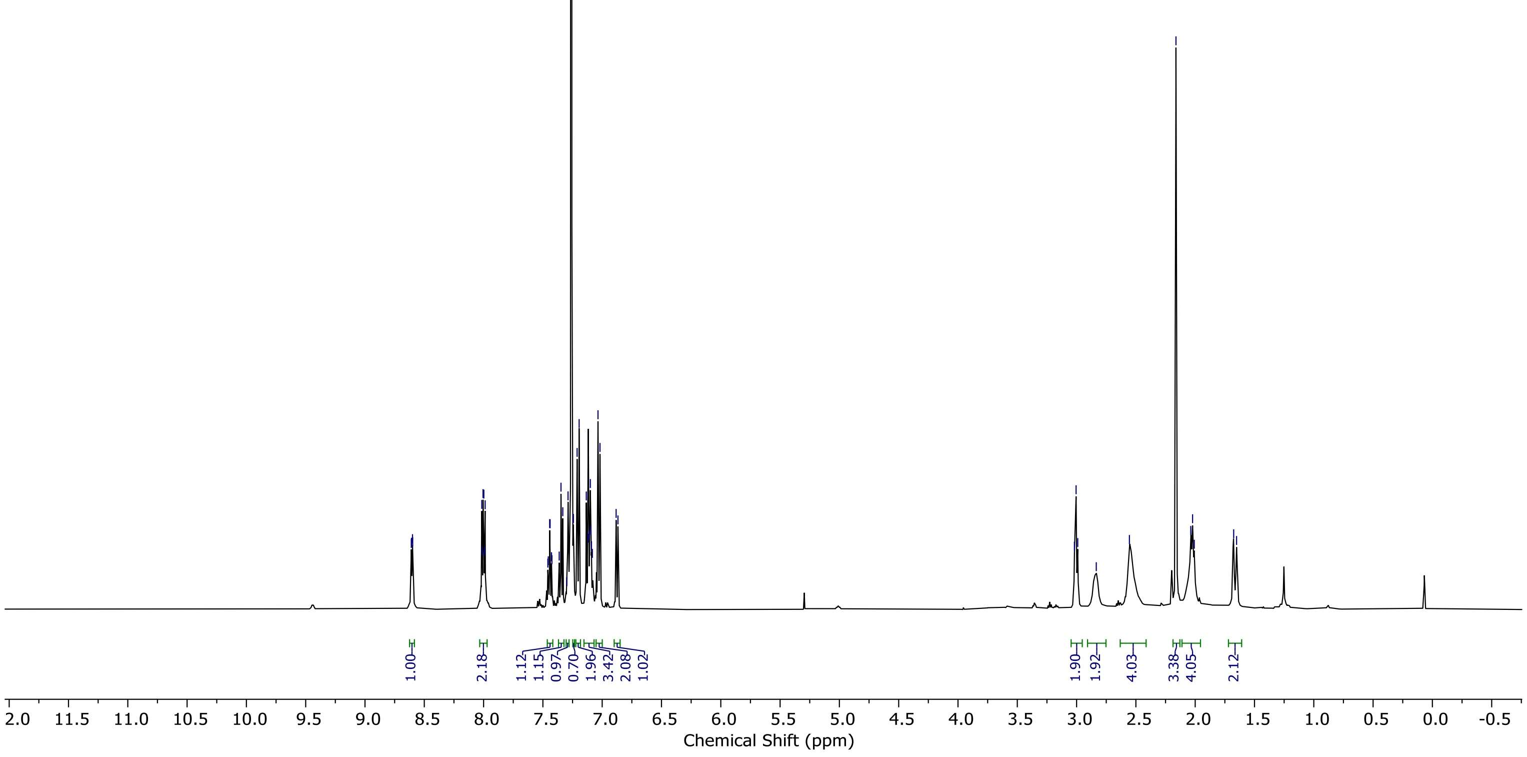

^{13}C NMR (126 MHz, $CDCl_3$) of [2-(*o*-tolyl)pyridine]–[haloperidol] 10c

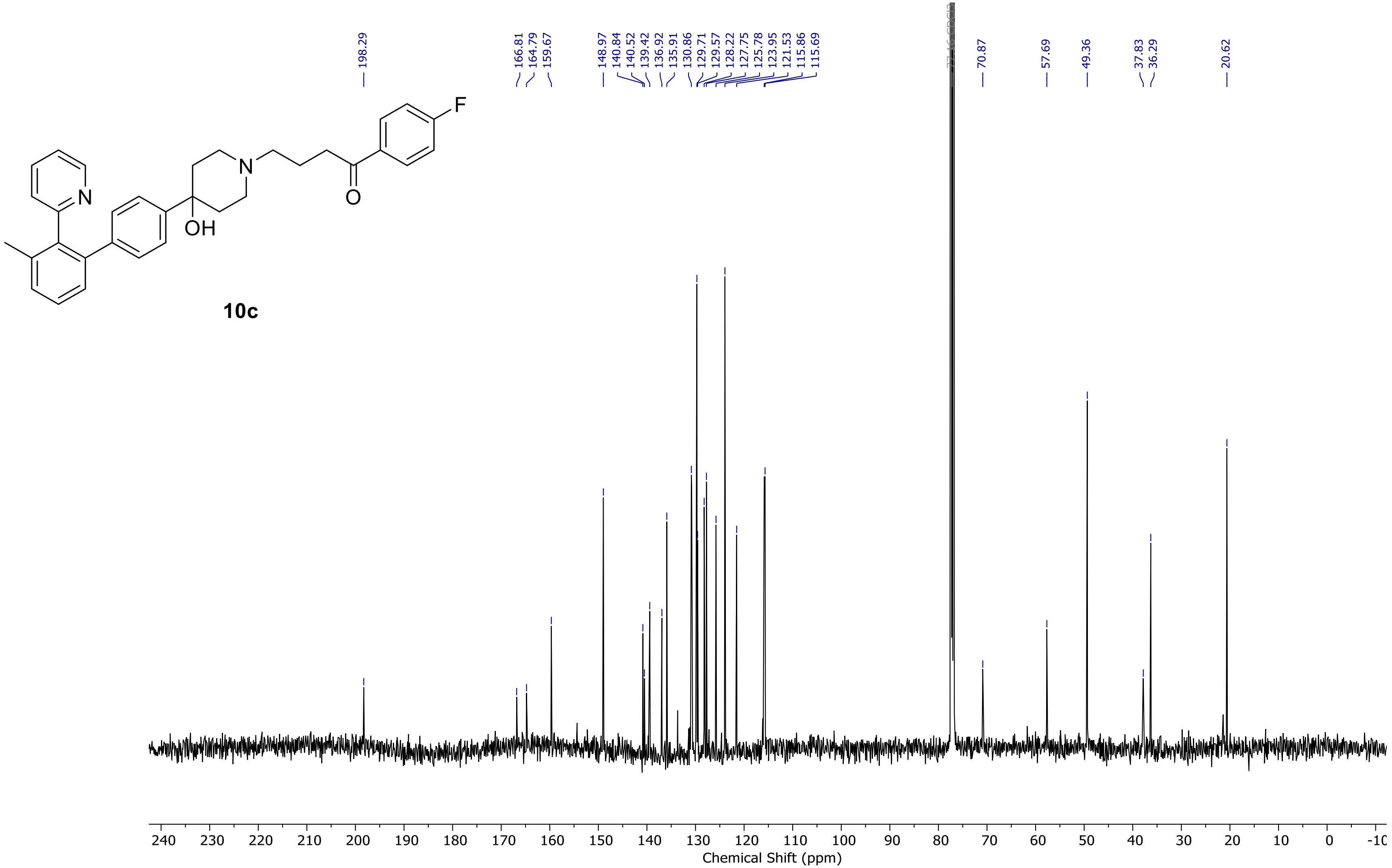

^{19}F NMR (376 MHz, $CDCl_3$) of [2-(*o*-tolyl)pyridine]–[haloperidol] 10c

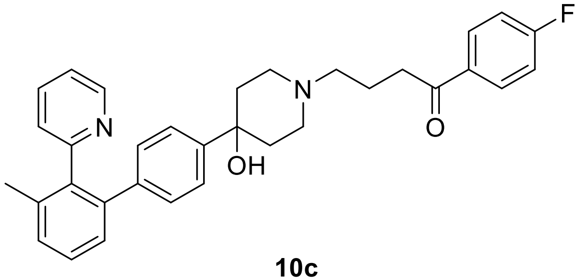

10c

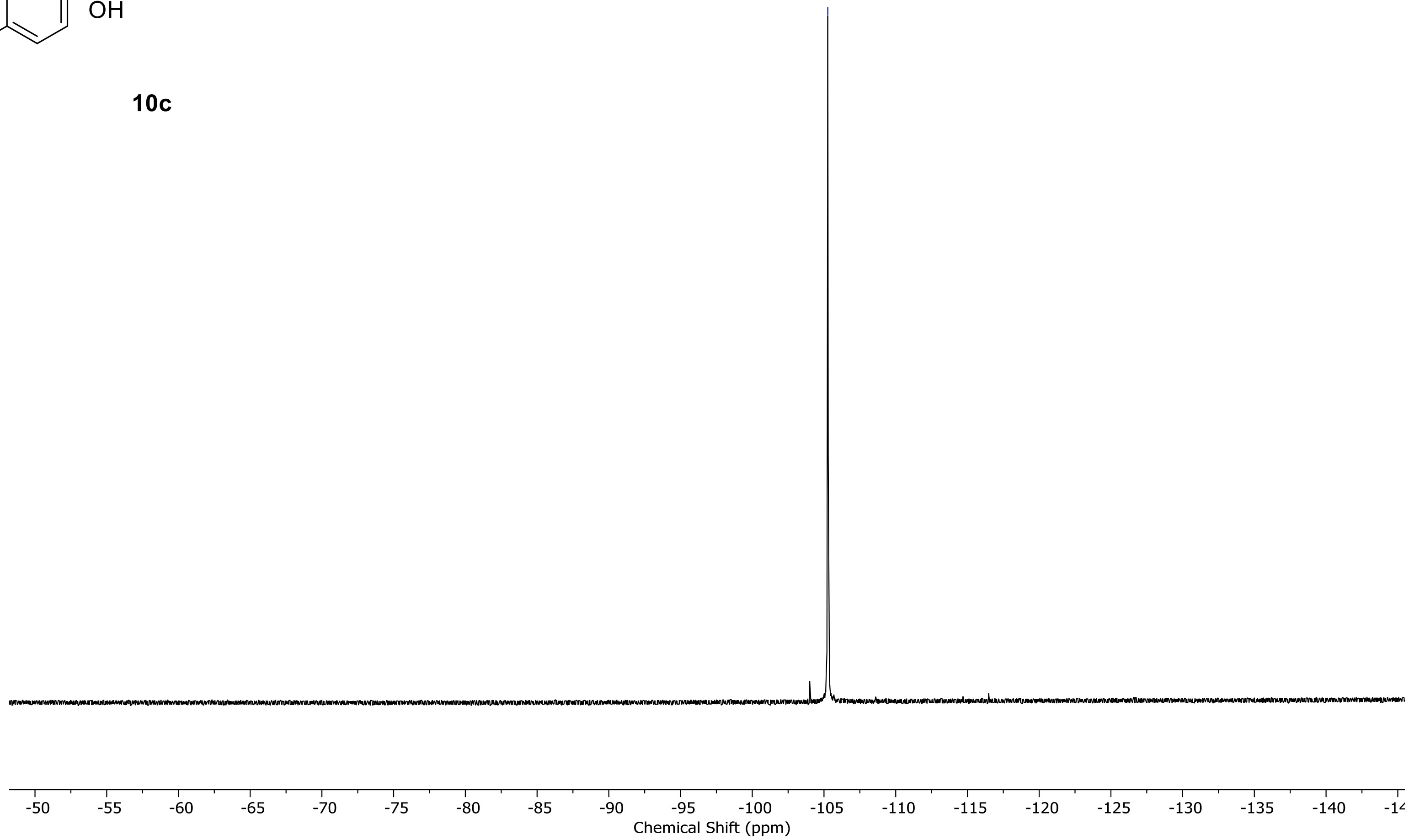

^{1}H NMR (400 MHz, $CDCl_3$) of [2-(*o*-tolyl)pyridine]–[chlormezanone] 10d

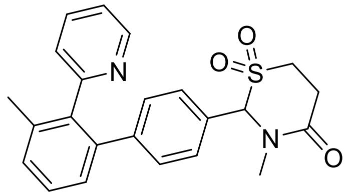

10d

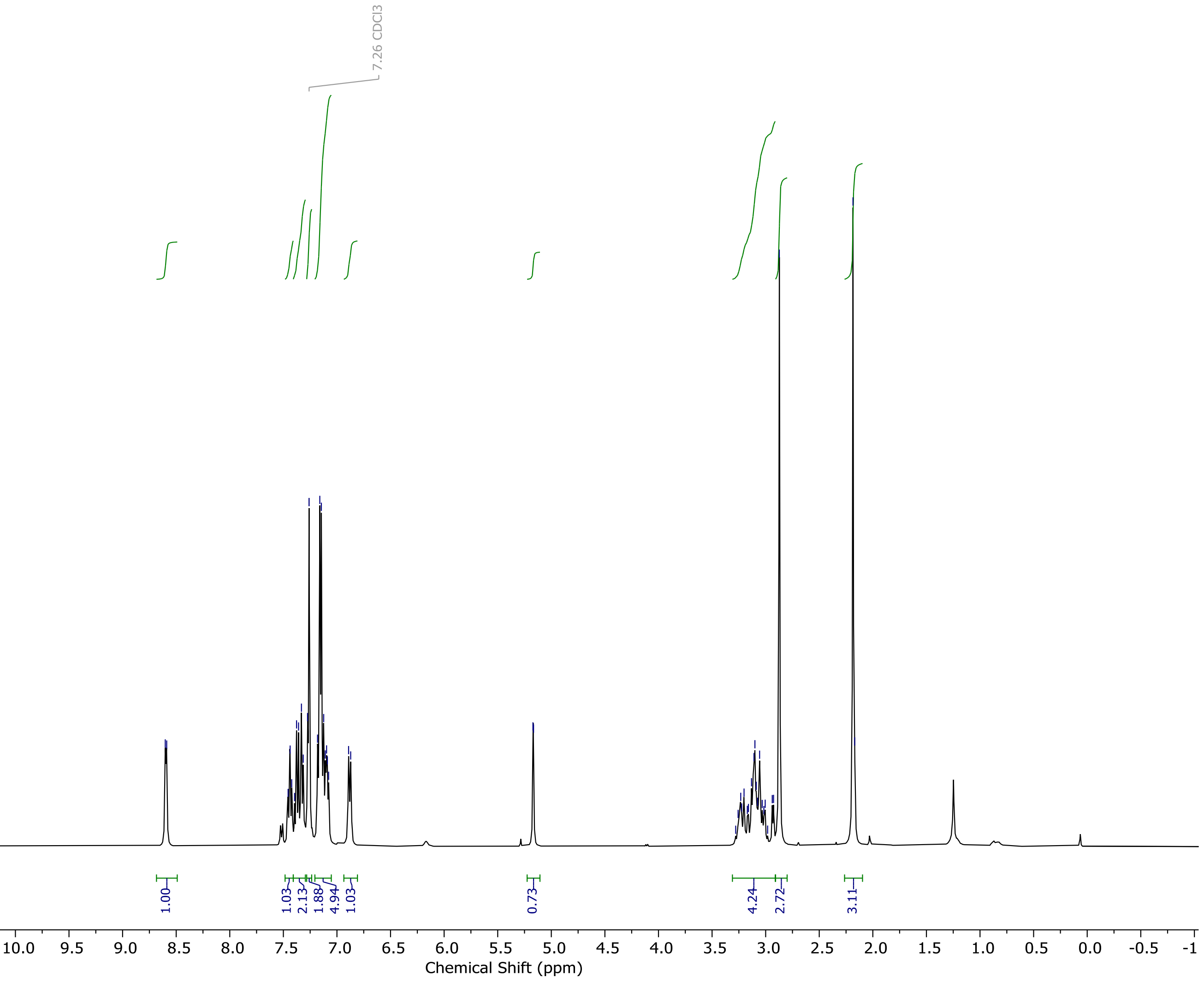

^{13}C NMR (101 MHz, CDCl$_3$) of [2-(*o*-tolyl)pyridine]–[chlormezanone] 10d

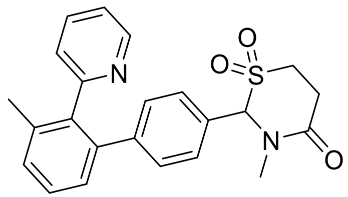

10d

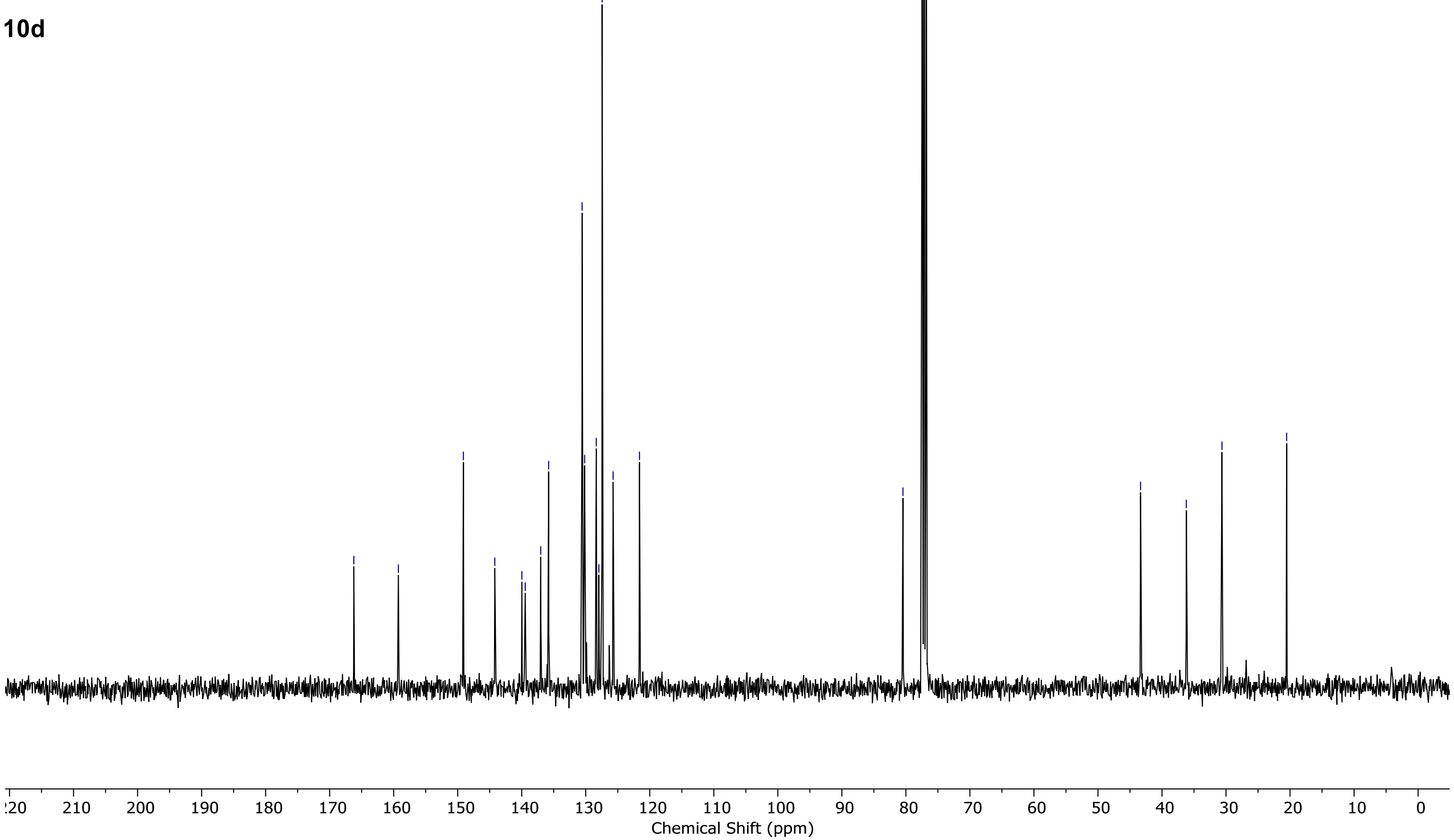

^{1}H NMR (400 MHz, $CDCl_3$) of [2-(*o*-tolyl)pyridine]–[chlorpropham] 10e

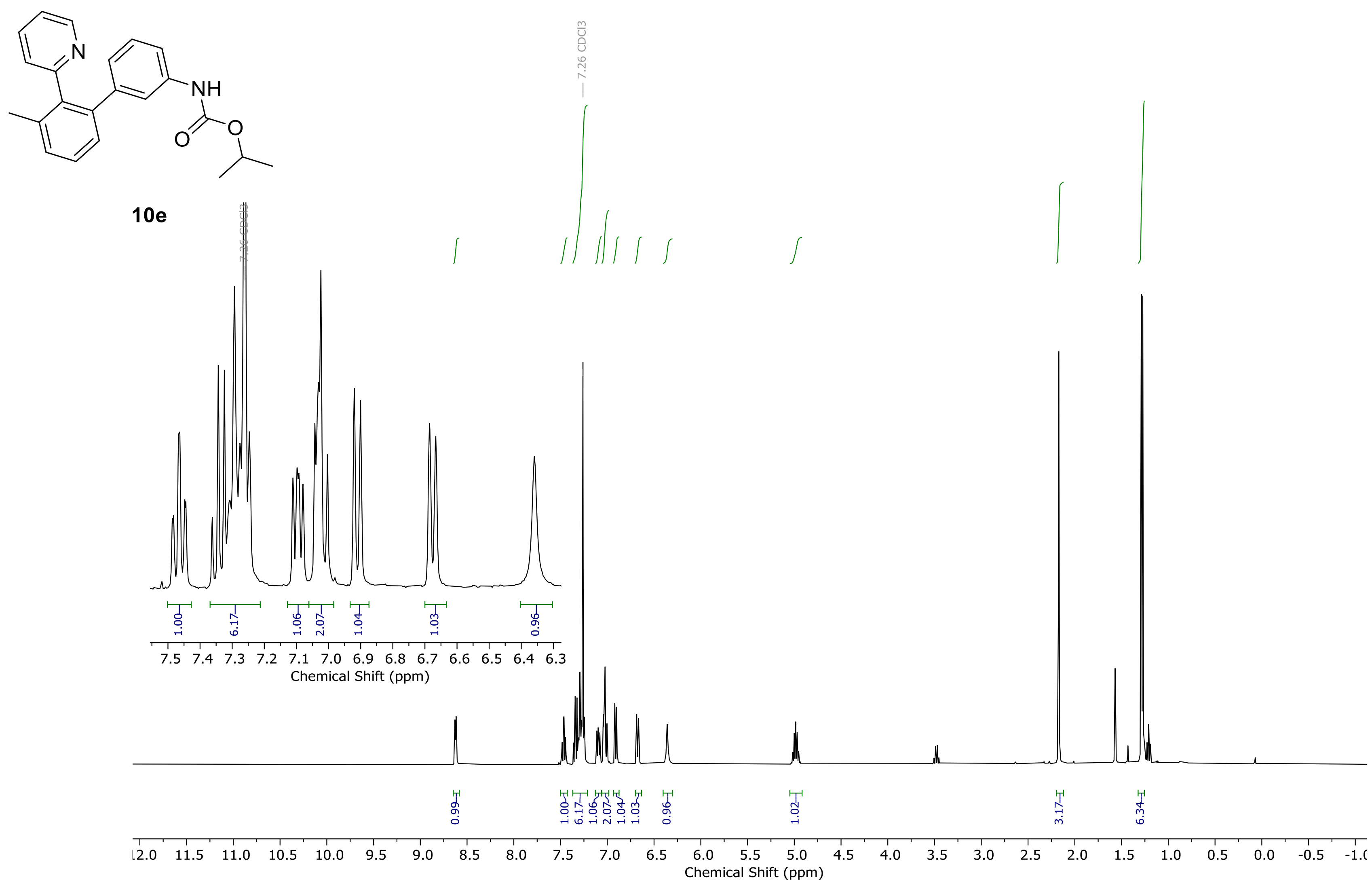

^{13}C NMR (126 MHz, CDCl$_3$) of [2-(*o*-tolyl)pyridine]–[chlorpropham] 10e

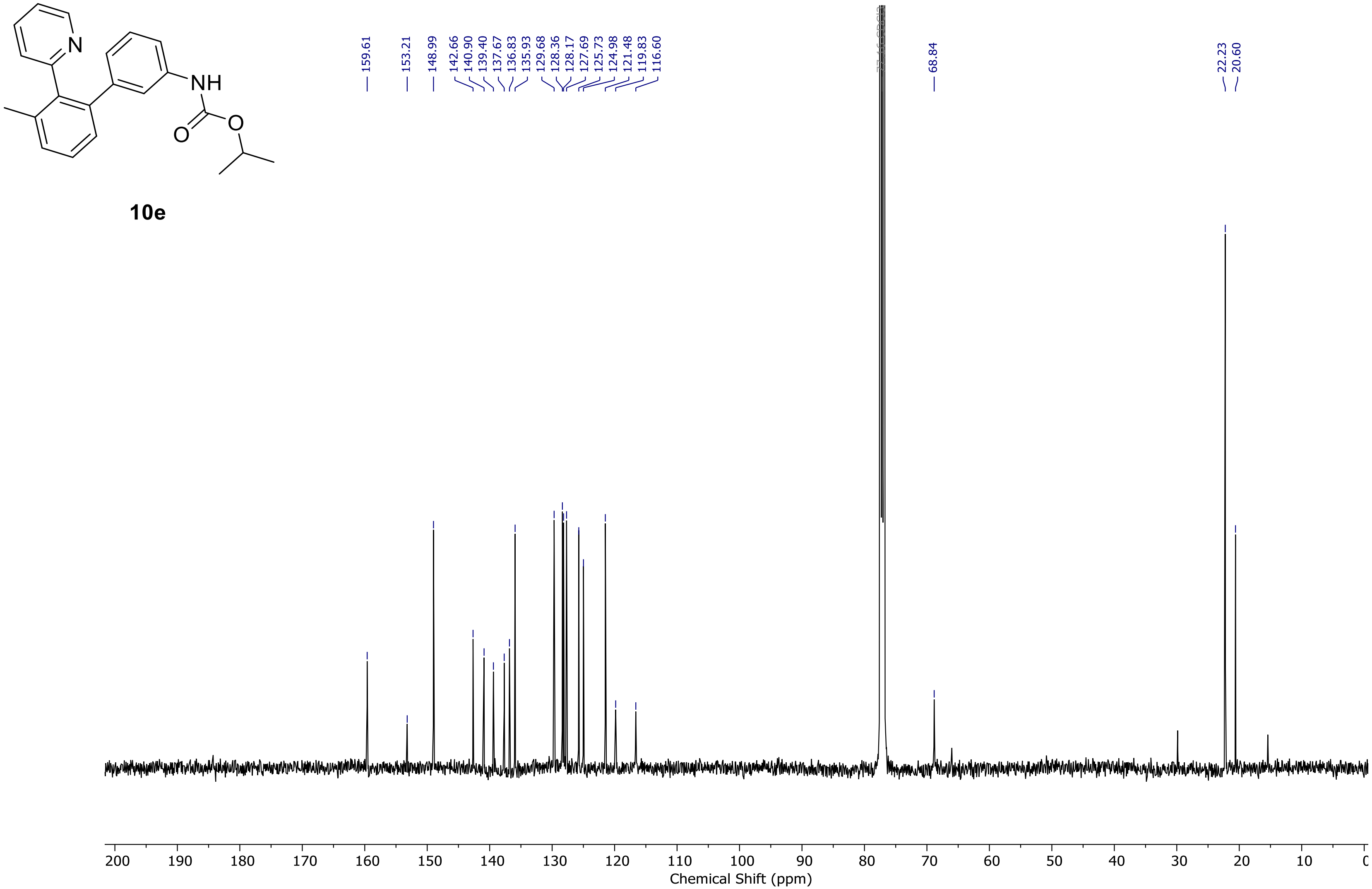

^{1}H NMR (500 MHz, $CDCl_3$) of [2-(*o*-tolyl)pyridine]–[ladasten] 10f

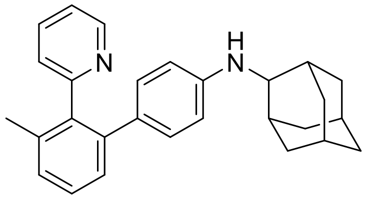

10f

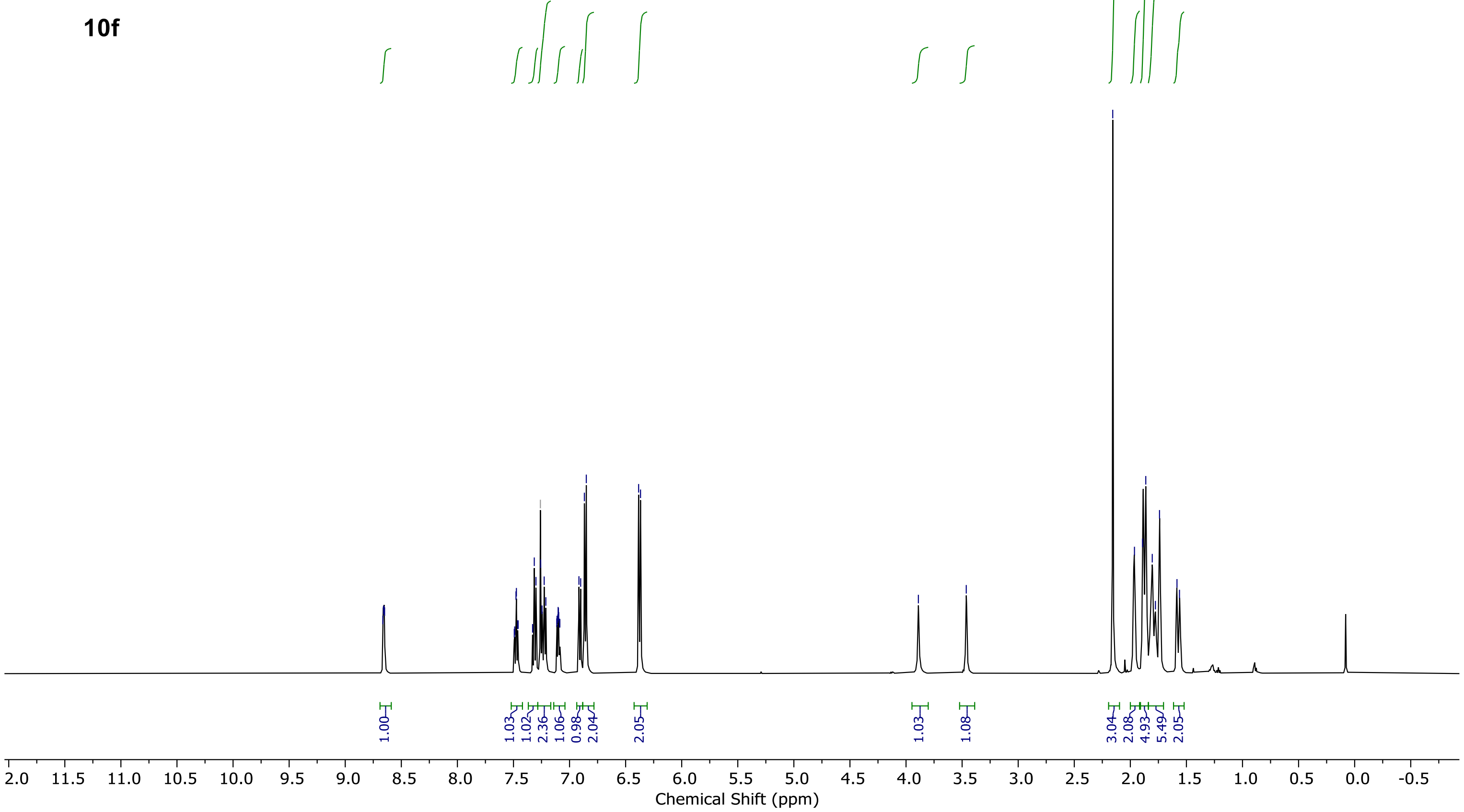

^{13}C NMR (126 MHz, $CDCl_3$) of [2-(*o*-tolyl)pyridine]–[ladasten] 10f

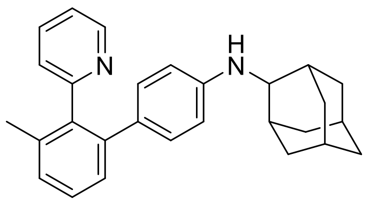

10f

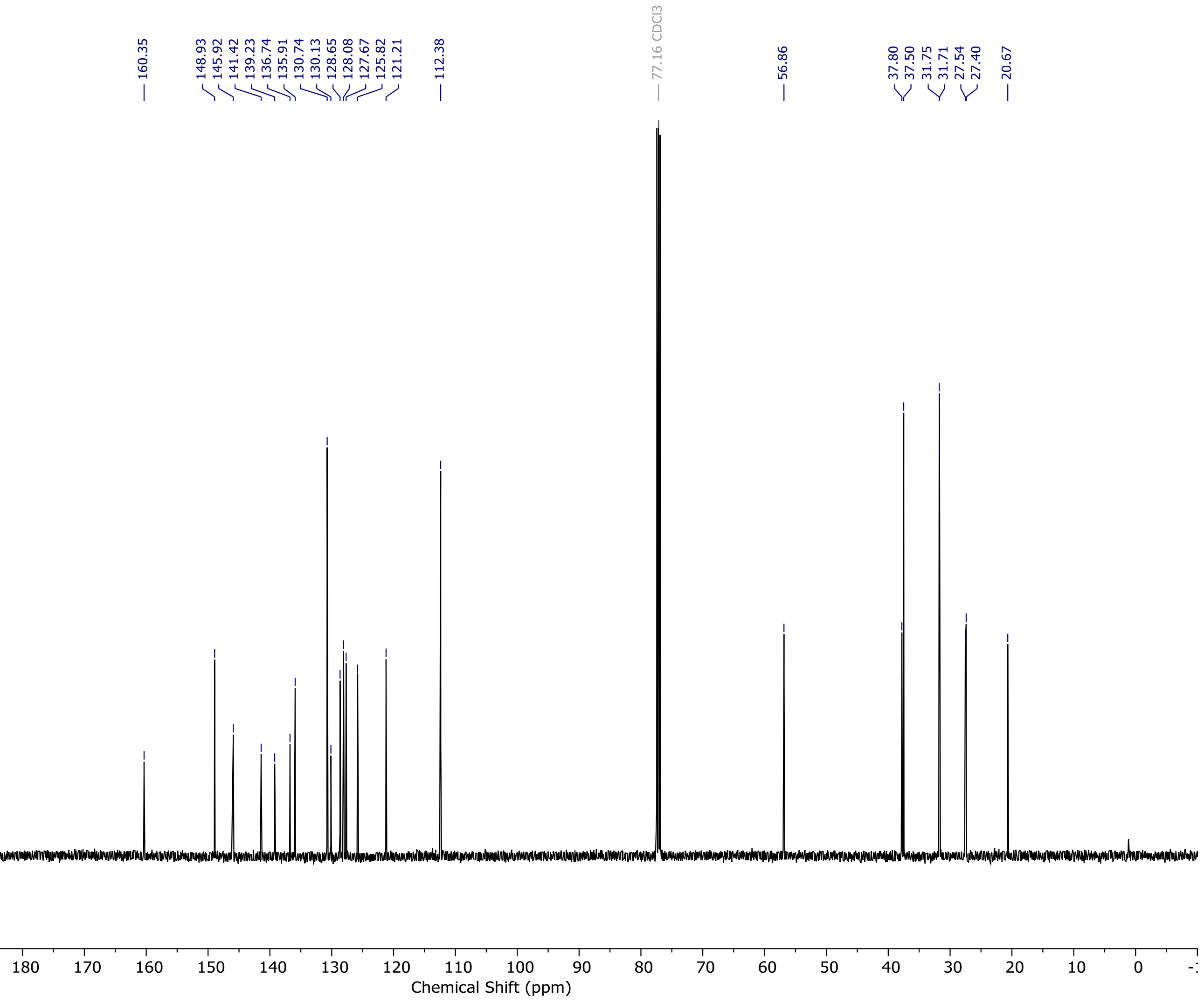

^{1}H NMR (400 MHz, $CDCl_3$) of [diazepam]-[5-*m*-xylene] 11g

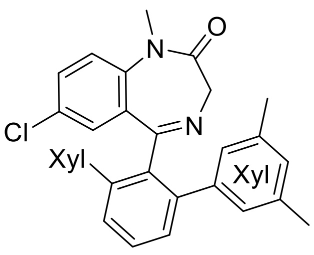

11g

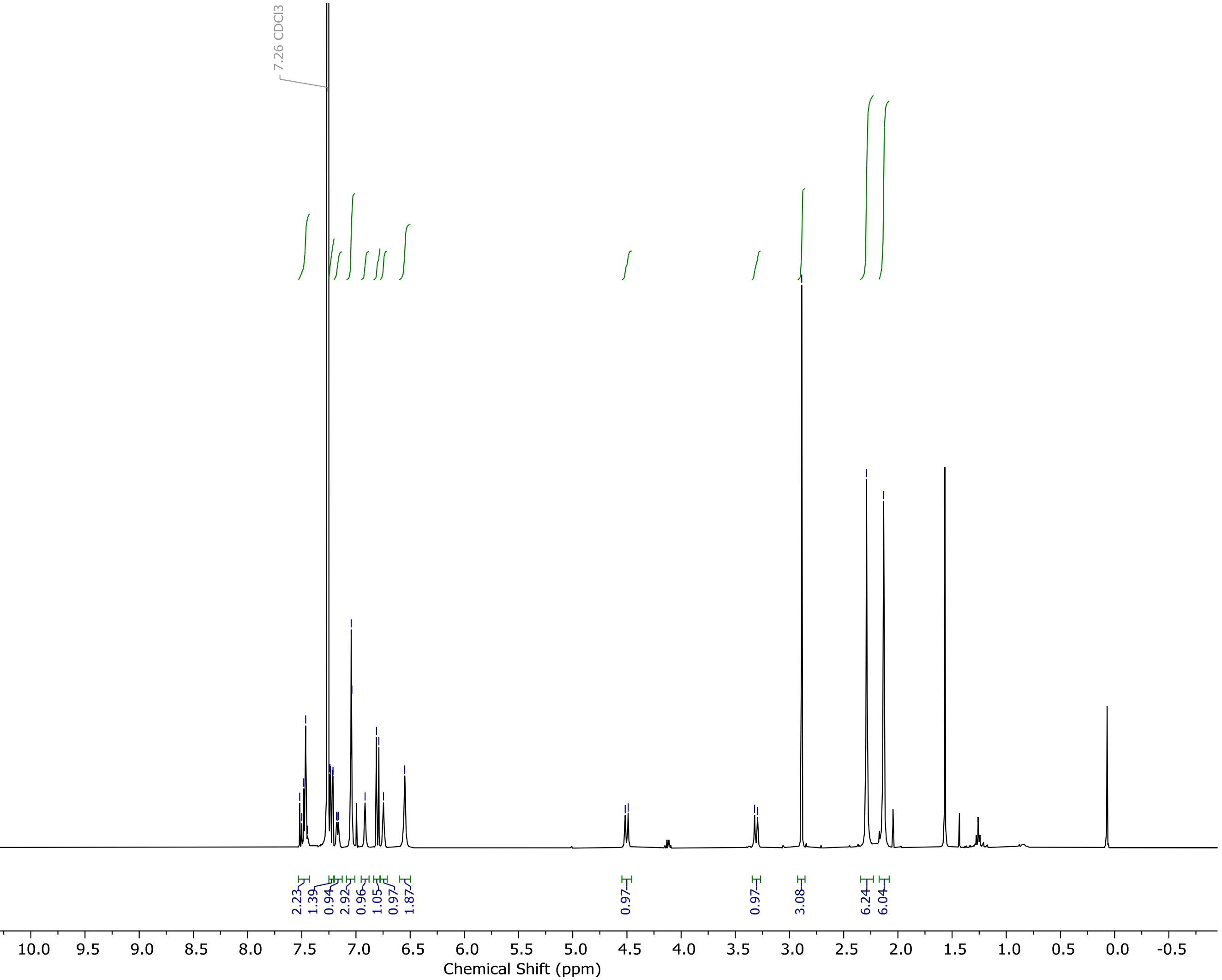

^{13}C NMR (101 MHz, $CDCl_3$) of [diazepam]-[5-*m*-xylene] 11g

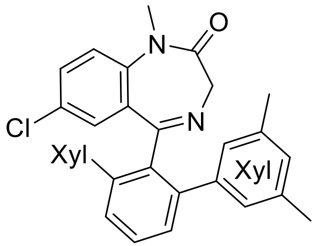

11g

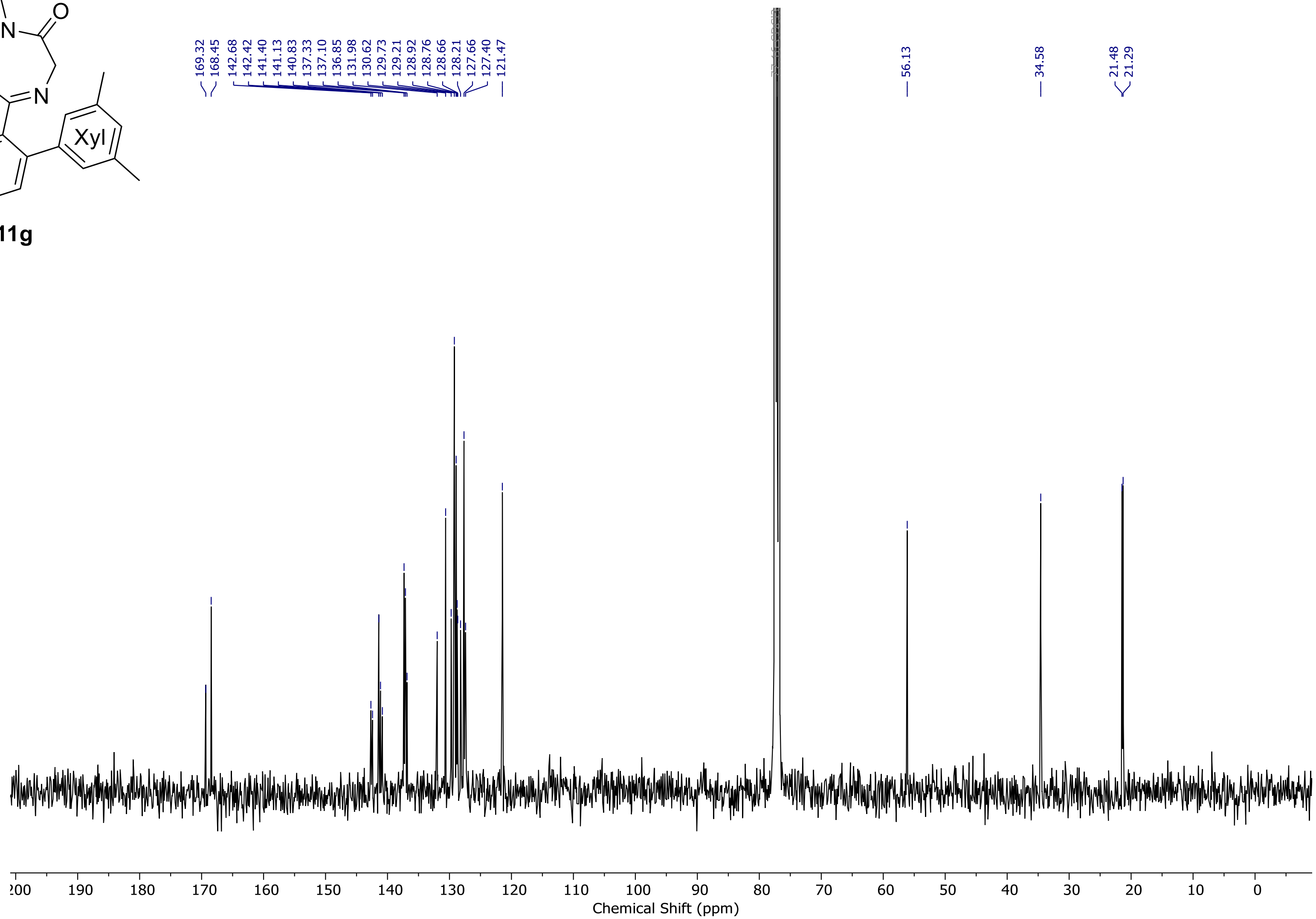

^{1}H NMR (500 MHz, $CDCl_3$) of [zolimidine]–[5-*m*-xylene] 11h

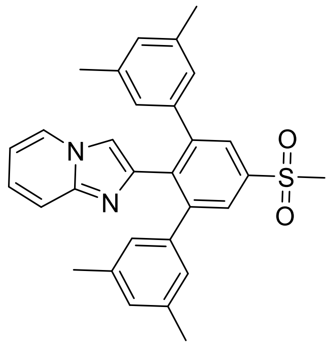

11h

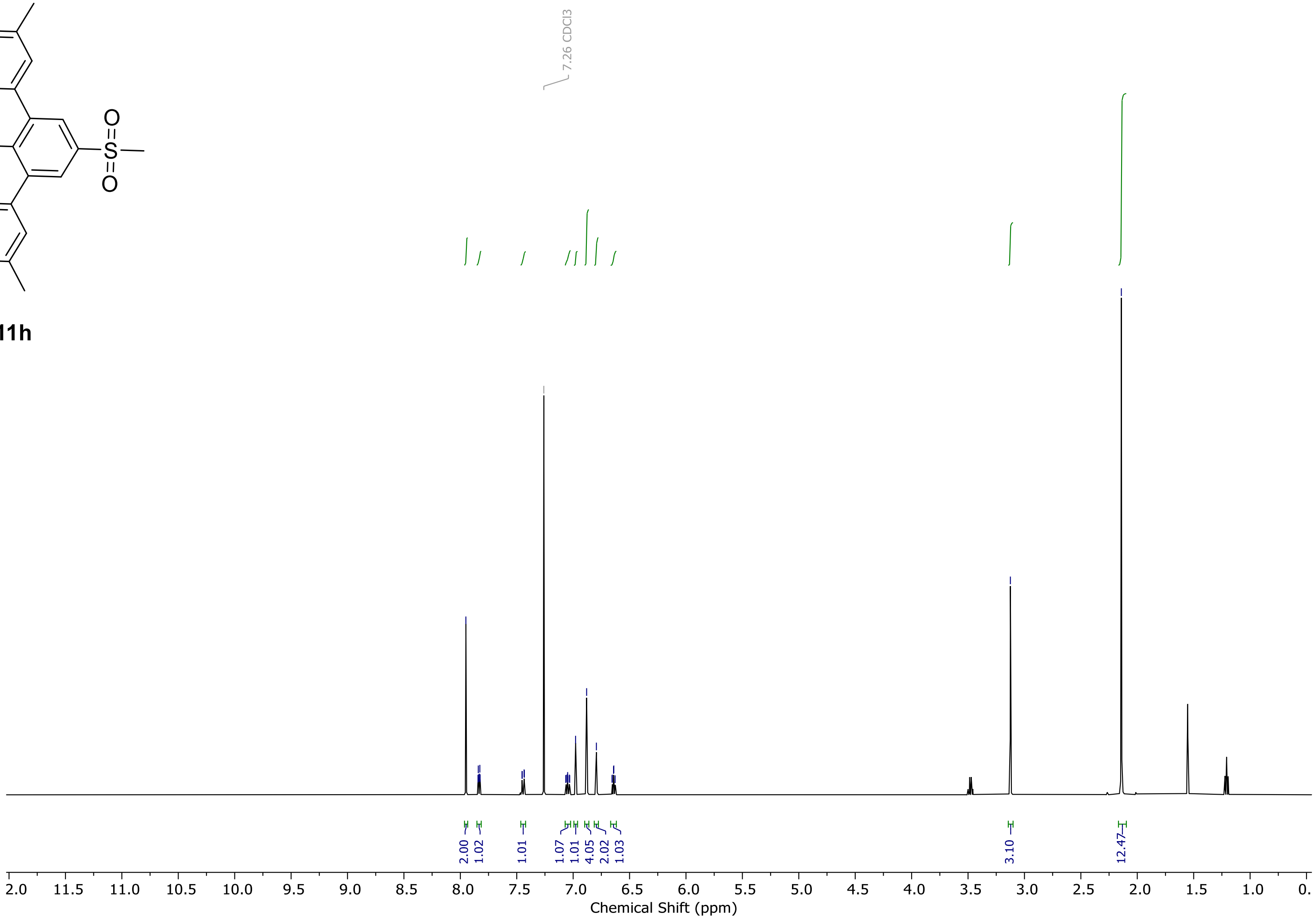

^{13}C NMR (126 MHz, $CDCl_3$) of [zolimidine]–[5-*m*-xylene] 11h

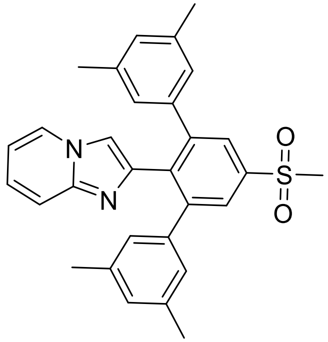

11h

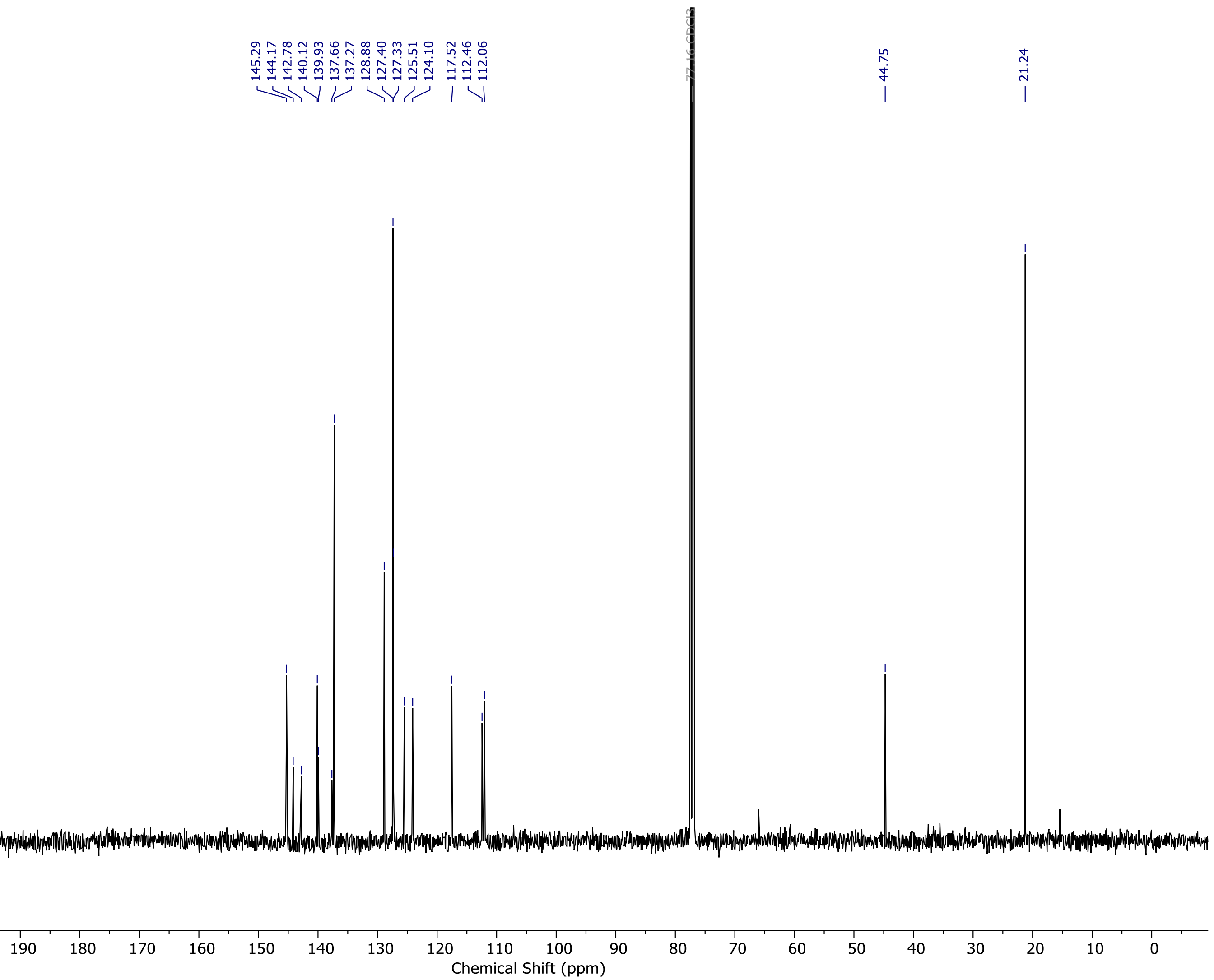

^{1}H NMR (400 MHz, CD_3OD) of [zolpidem derivative]-clozapine 10i

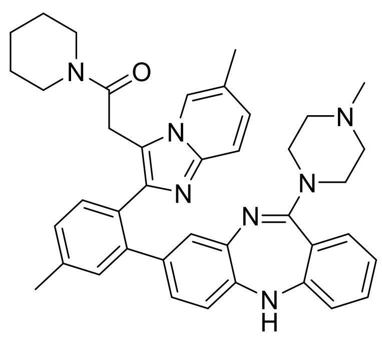

10i

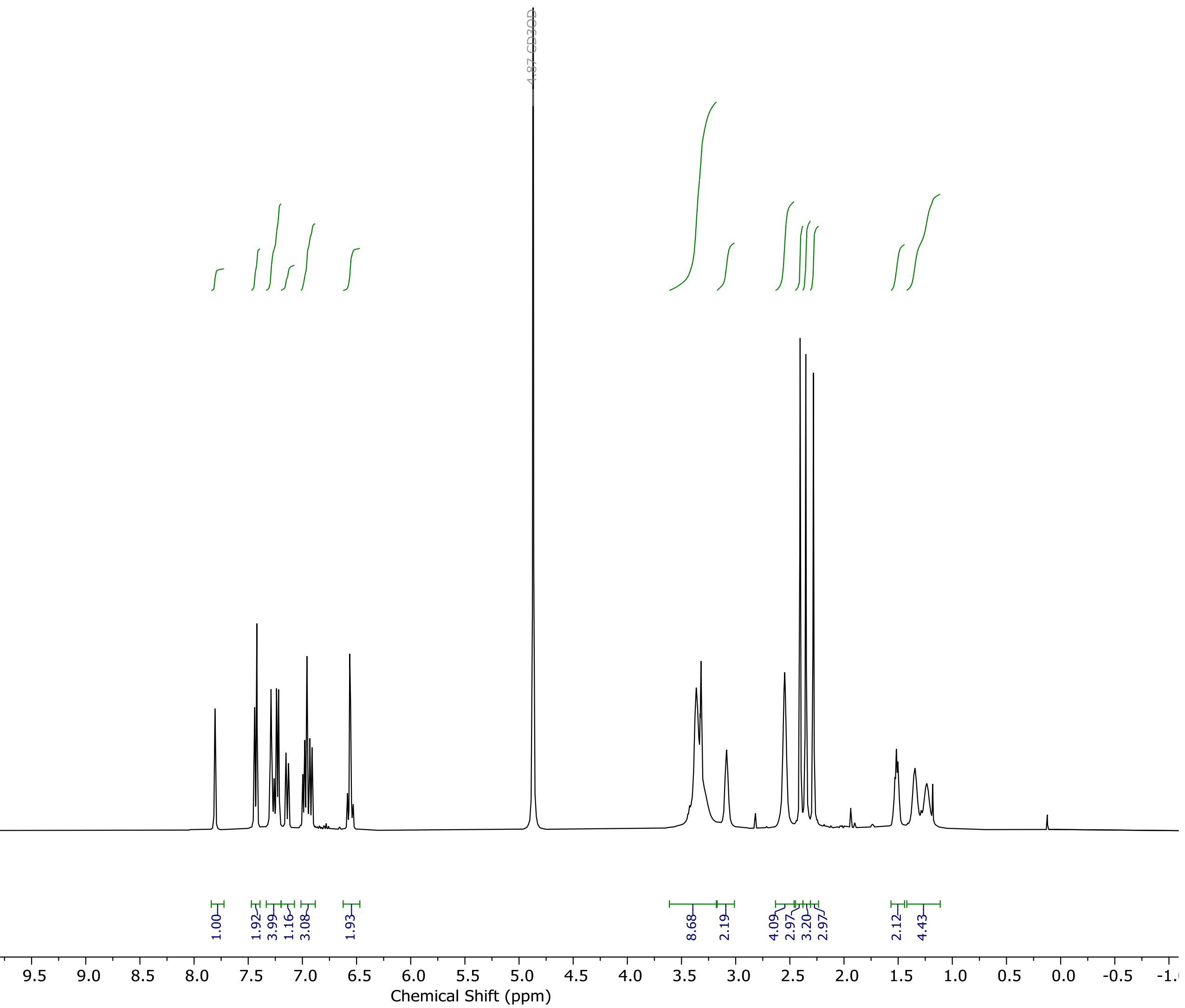

^{13}C NMR (01 MHz, CD_3OD) of [zolpidem derivative]-clozapine 10i

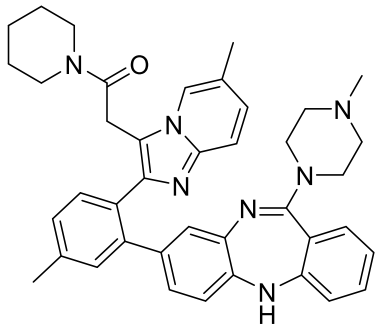

10i

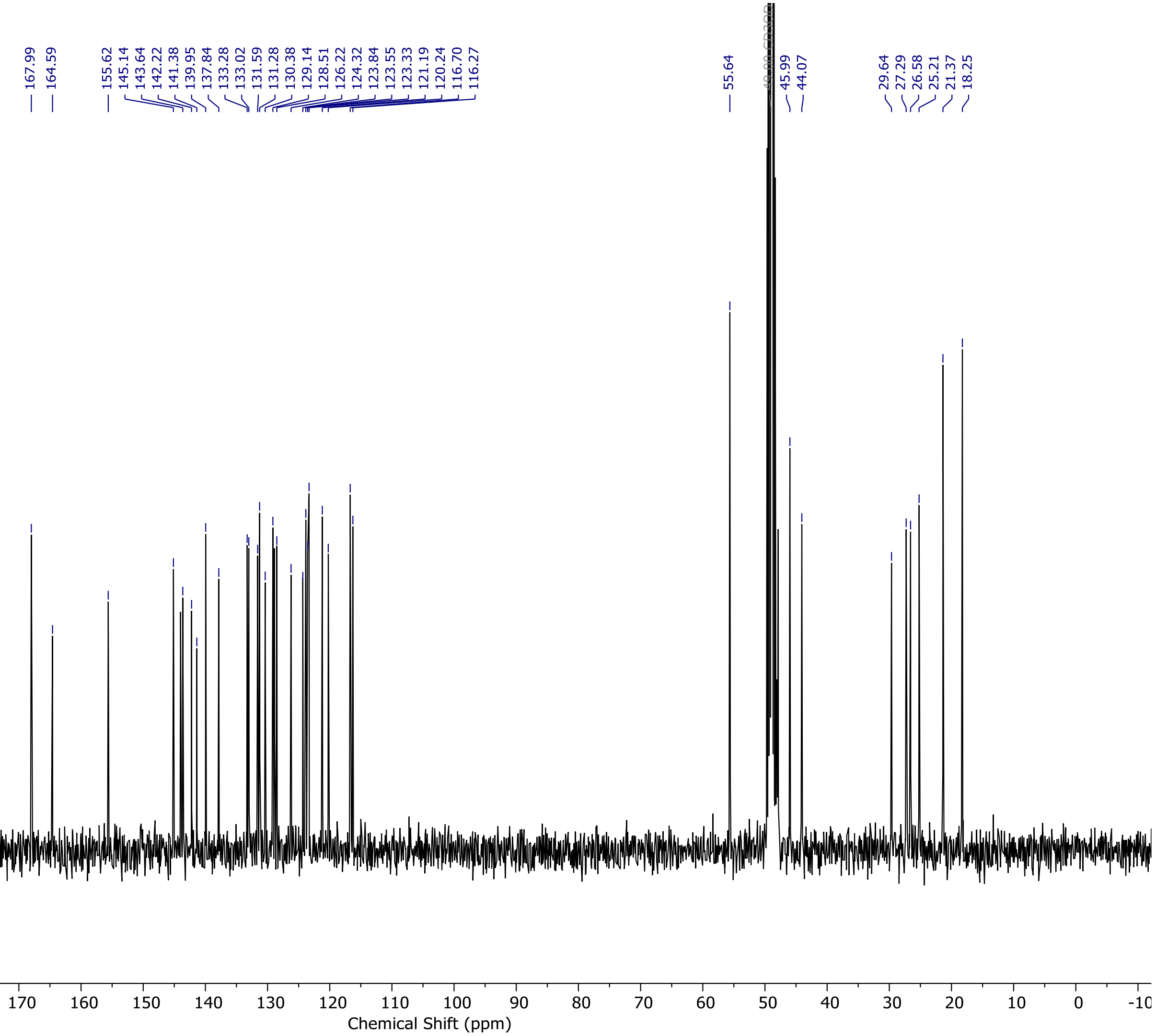

^{1}H NMR (500 MHz, CDCl$_3$) of [2-(*o*-tolyl)pyridine]–[trazodone] 10j

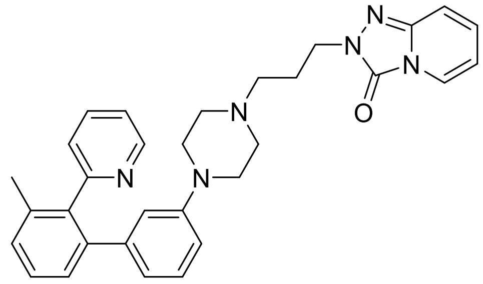

10j

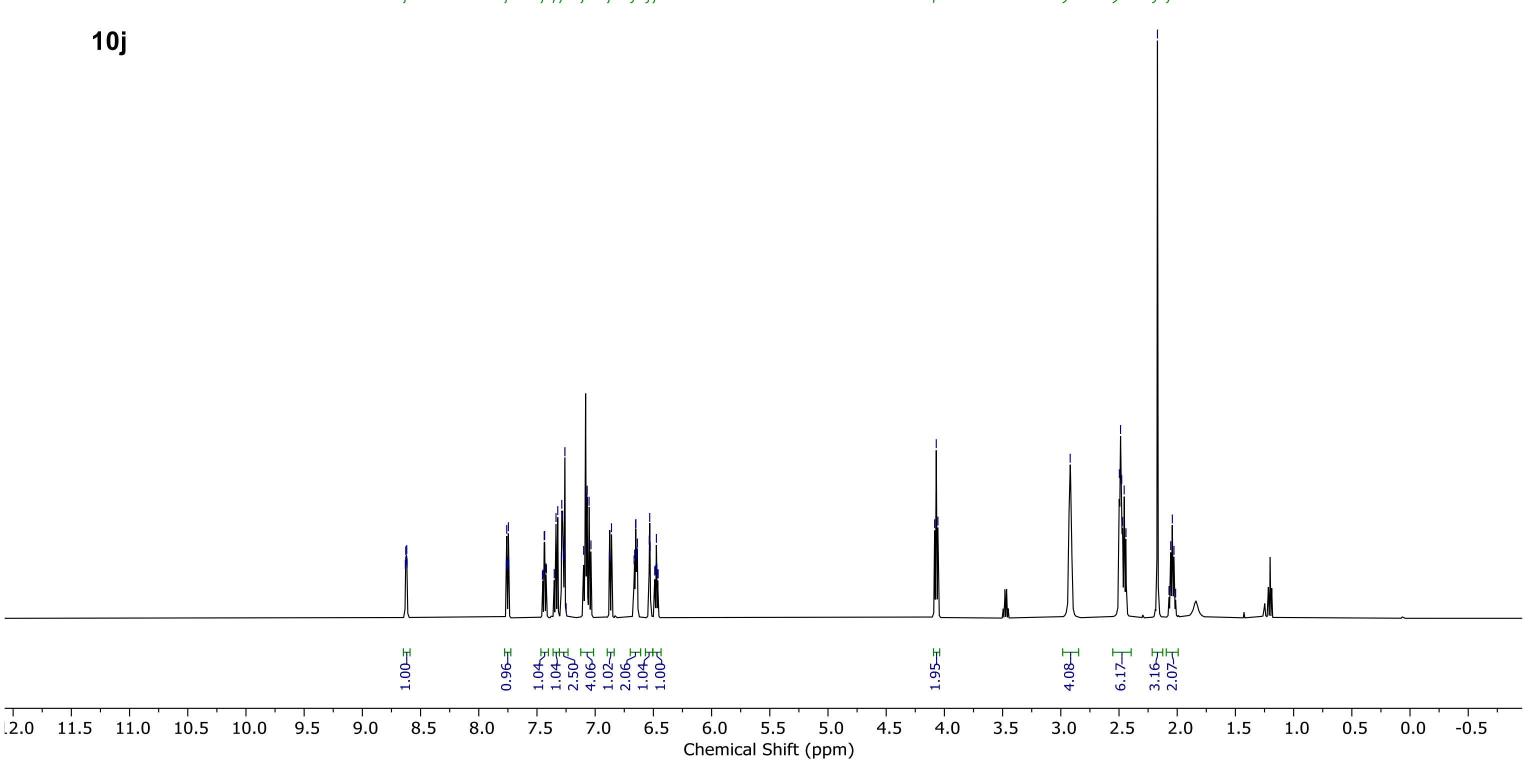

^{13}C NMR (126 MHz, $CDCl_3$) of [2-(*o*-tolyl)pyridine]–[trazodone] 10j

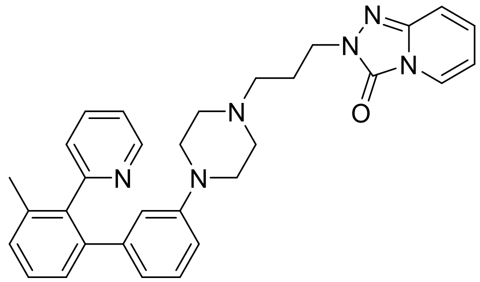

10j

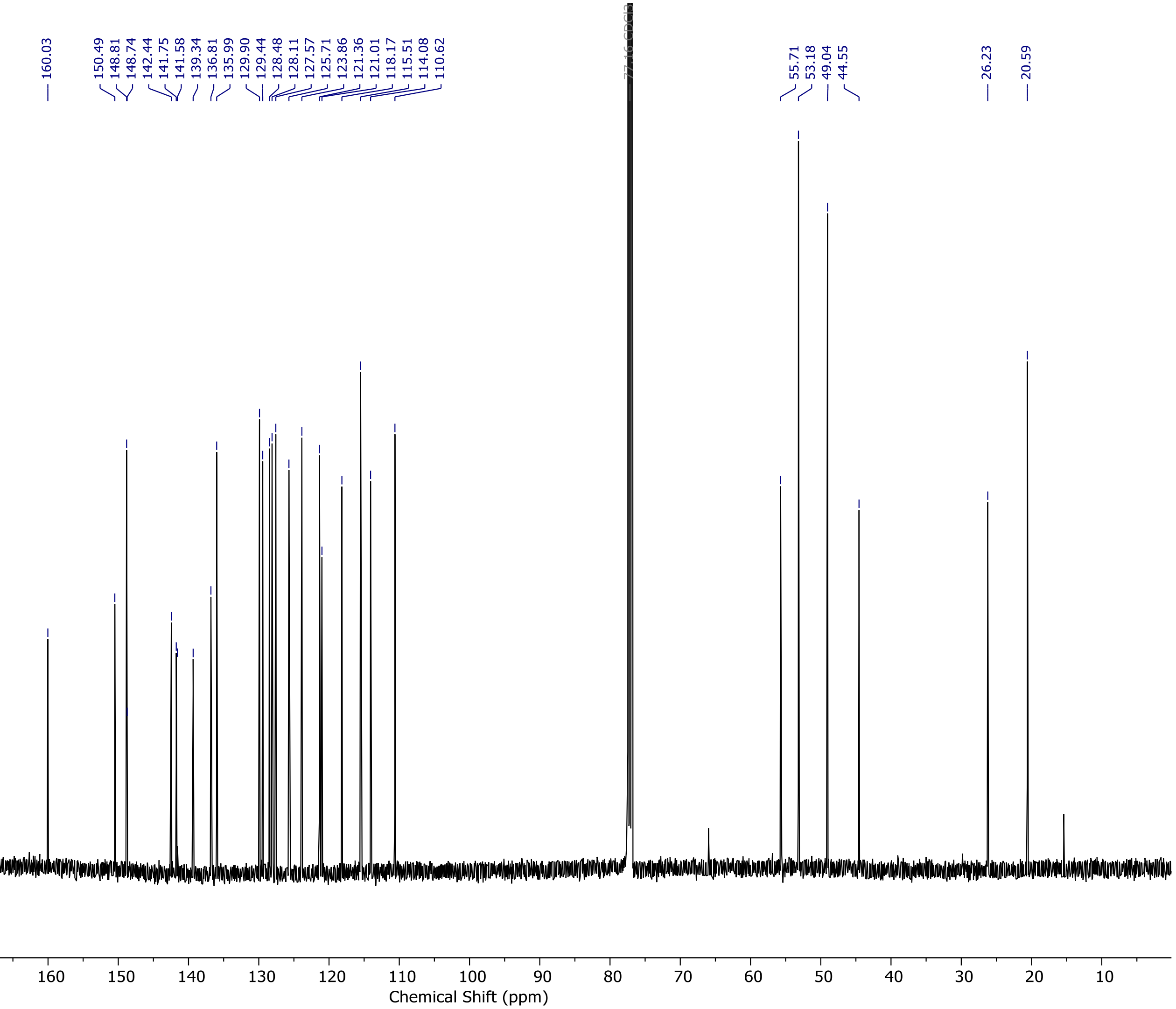

^{1}H NMR (400 MHz, $CDCl_3$) of [2-(*o*-tolyl)pyridine]–[meclizine] 10k

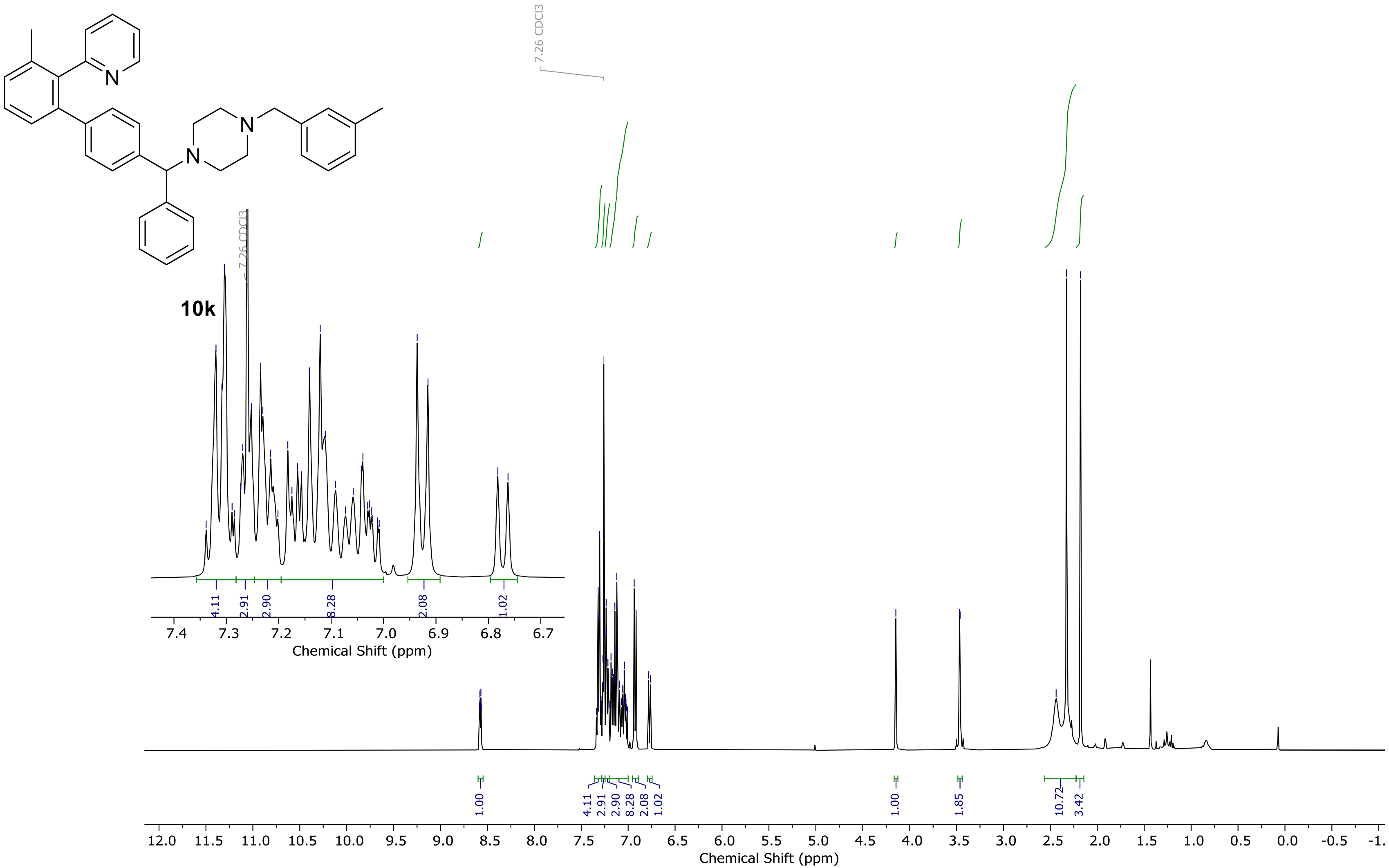

^{13}C NMR (101 MHz, CDCl$_3$) of [2-(*o*-tolyl)pyridine]–[meclizine] 10k

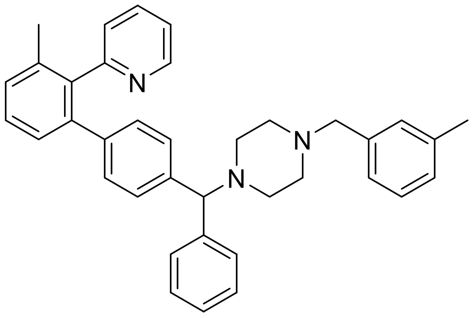

10k

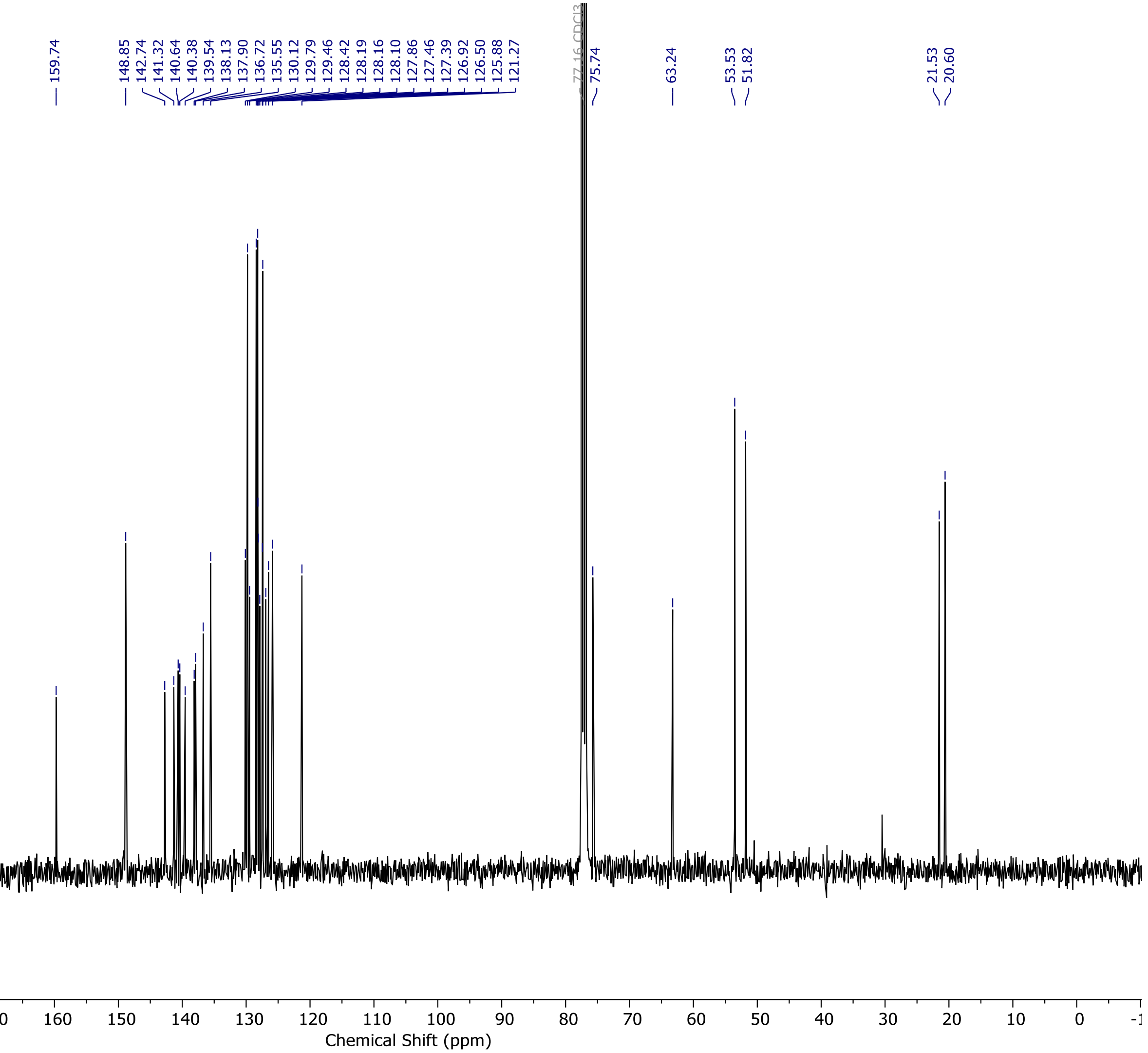

^{1}H NMR (400 MHz, CDCl$_3$) of [2-(*o*-tolyl)pyridine]–[fenofibrate] 10l

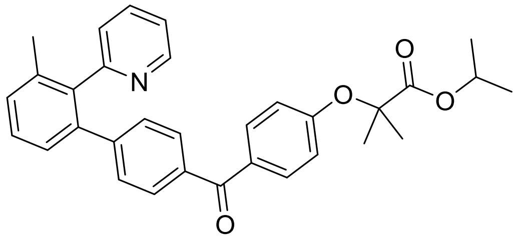

10l

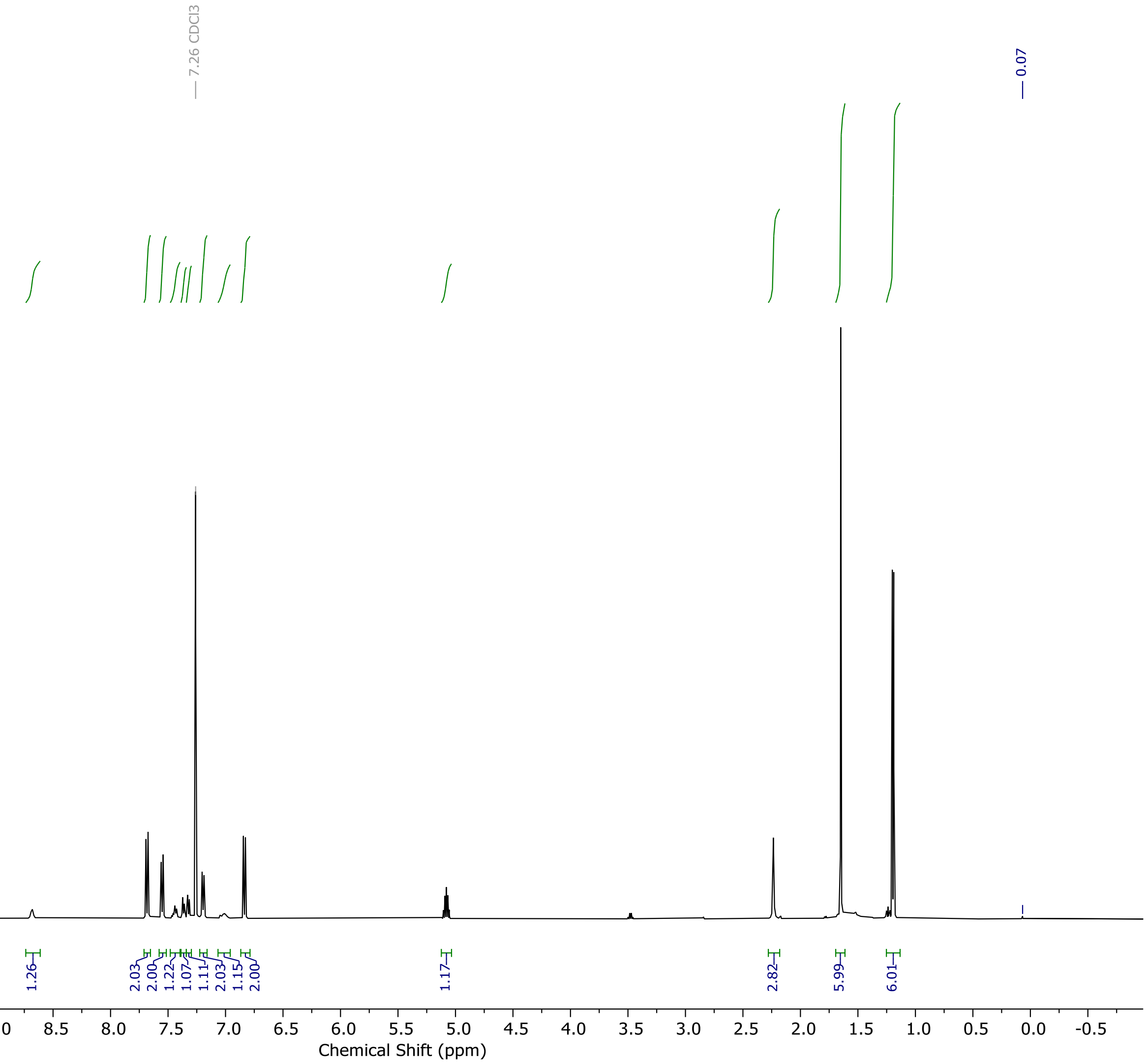

^{13}C NMR (101 MHz, CDCl$_3$) of [2-(*o*-tolyl)pyridine]–[fenofibrate] 10l

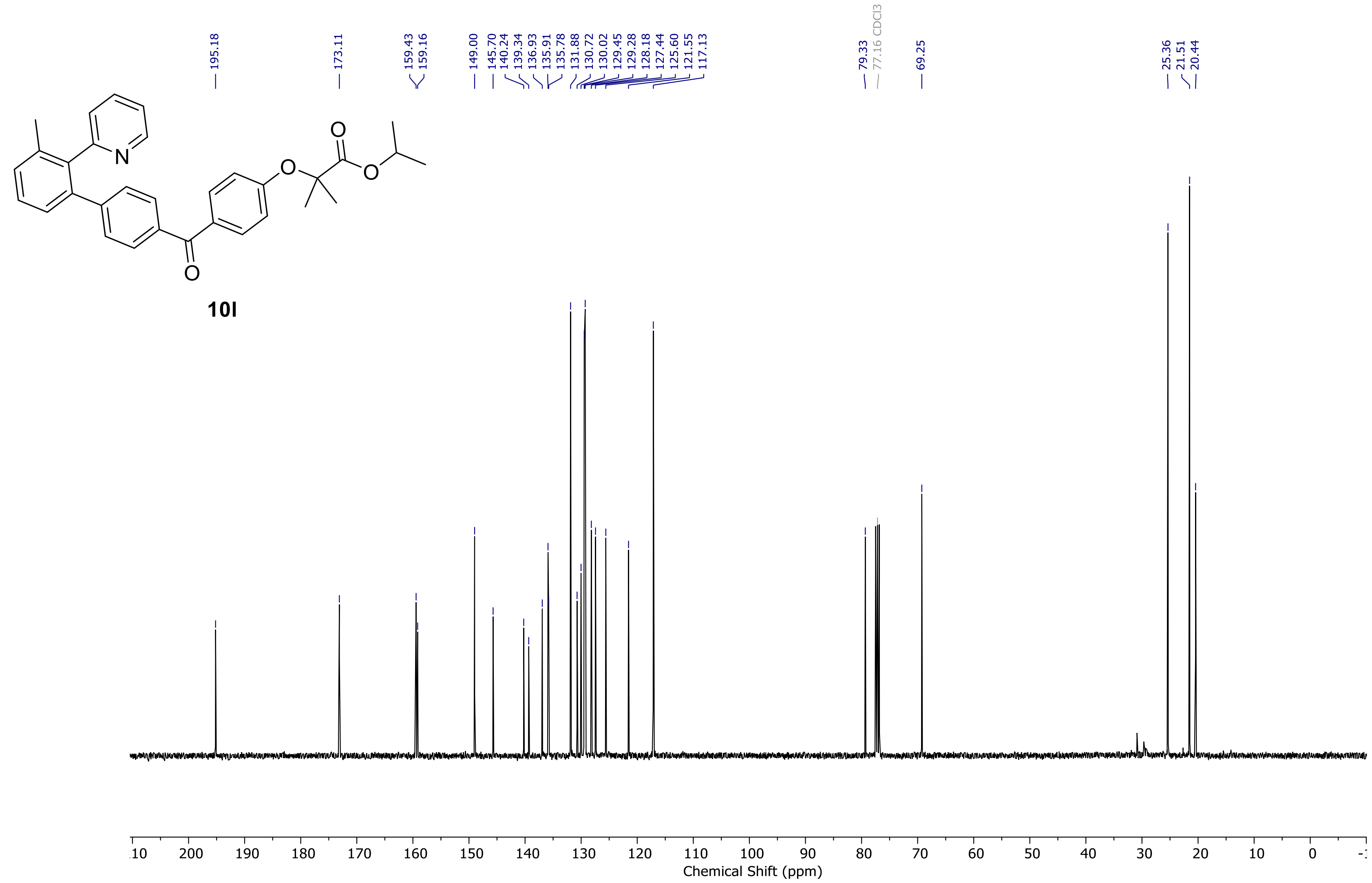

^{1}H NMR (400 MHz, $CDCl_3$) of [2-(*o*-tolyl)pyridine]–[δ-tocopherol] 10m

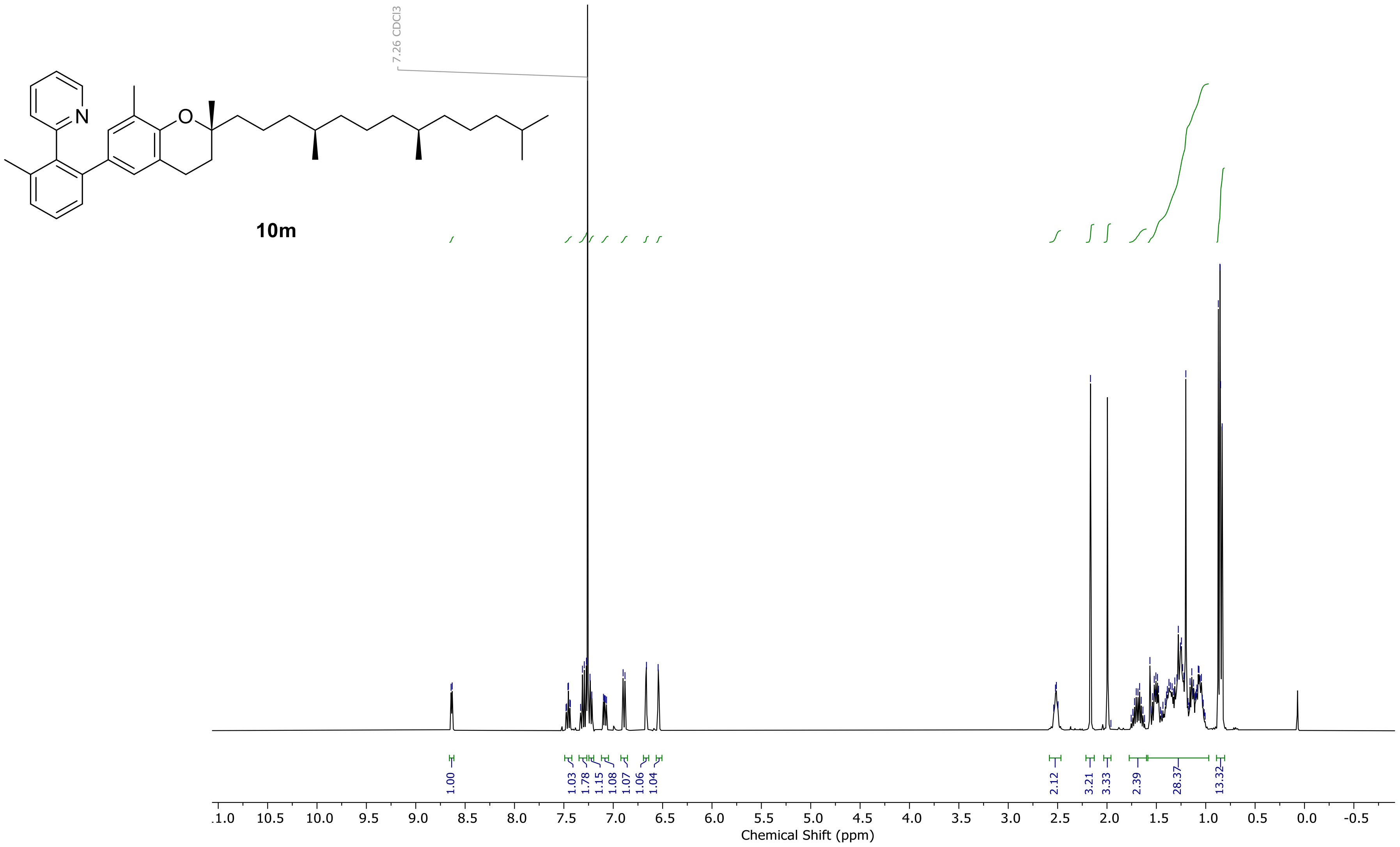

^{13}C NMR (101 MHz, CDCl$_3$) of [2-(*o*-tolyl)pyridine]–[δ-tocopherol] 10m

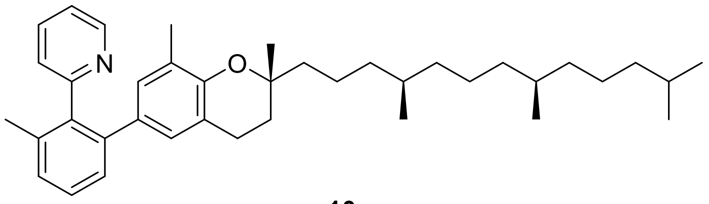

10m

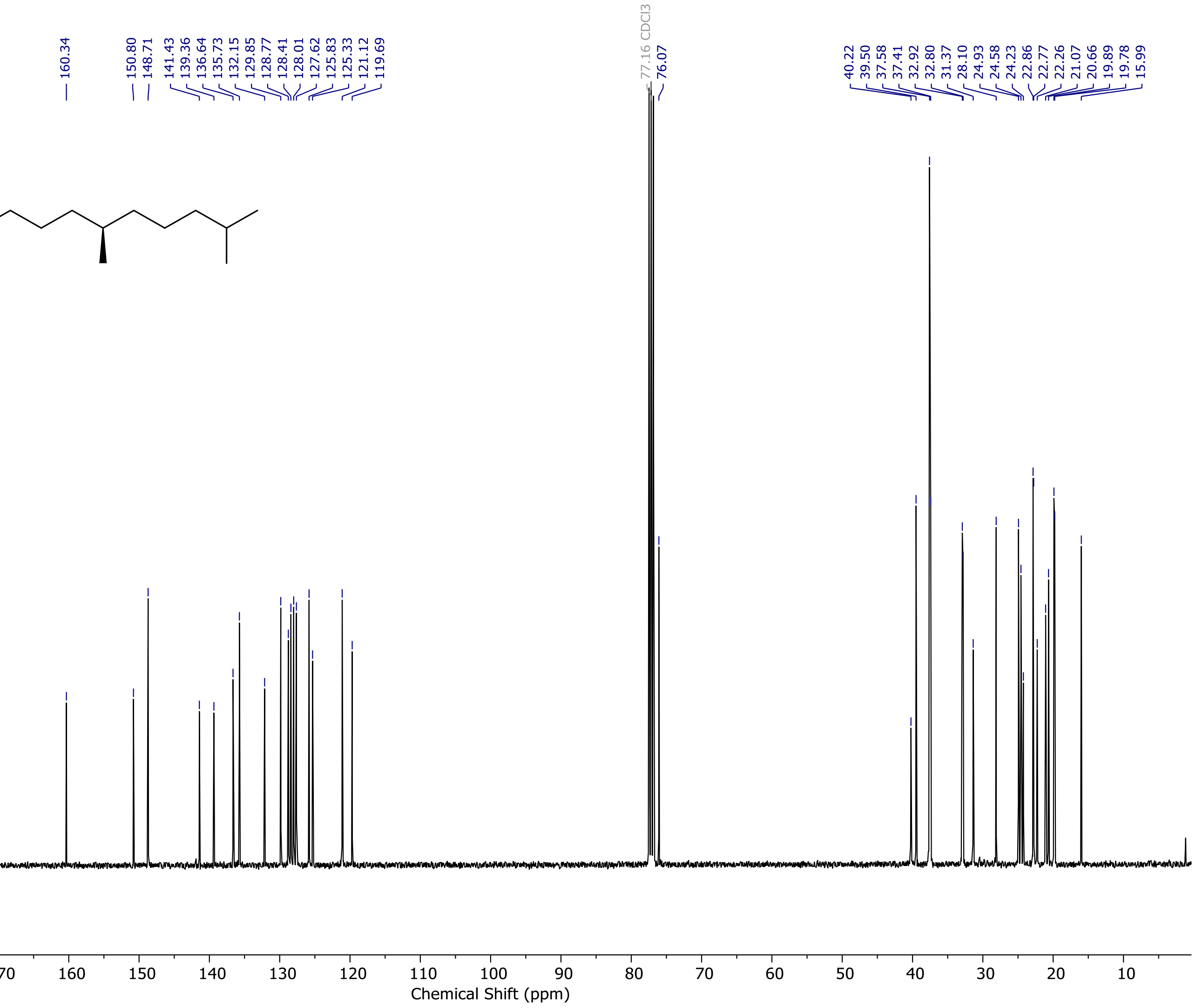

^{1}H NMR (400 MHz, $CDCl_3$) of Starting Material 8e

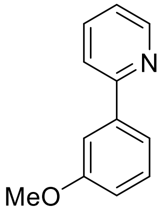

8e

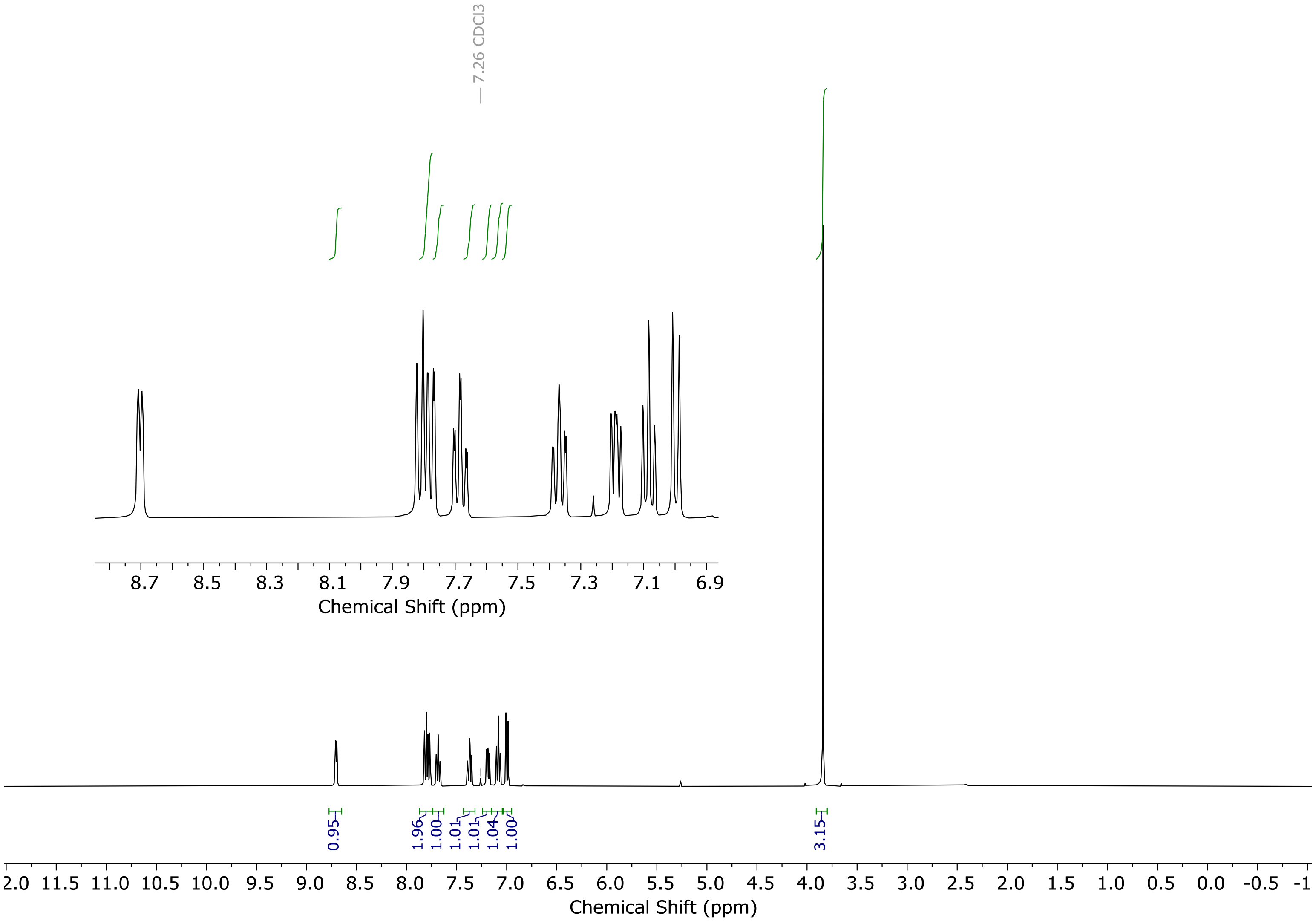

^{13}C NMR (126 MHz, $CDCl_3$) of Starting Material 8e

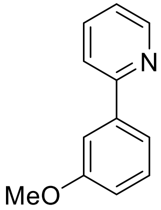

8e

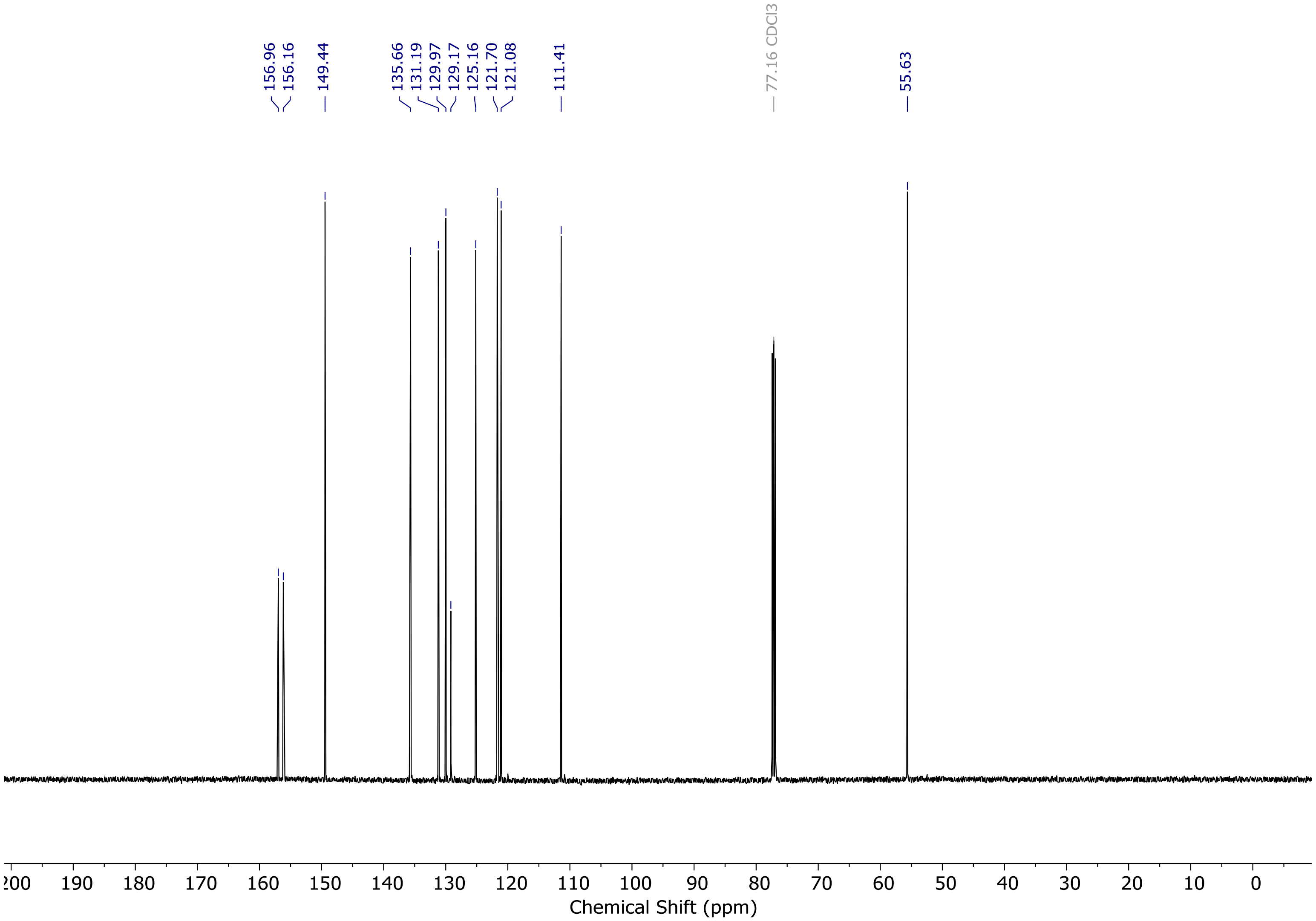

^{1}H NMR (400 MHz, $CDCl_3$) of Starting Material 8f

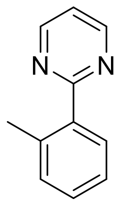

8f

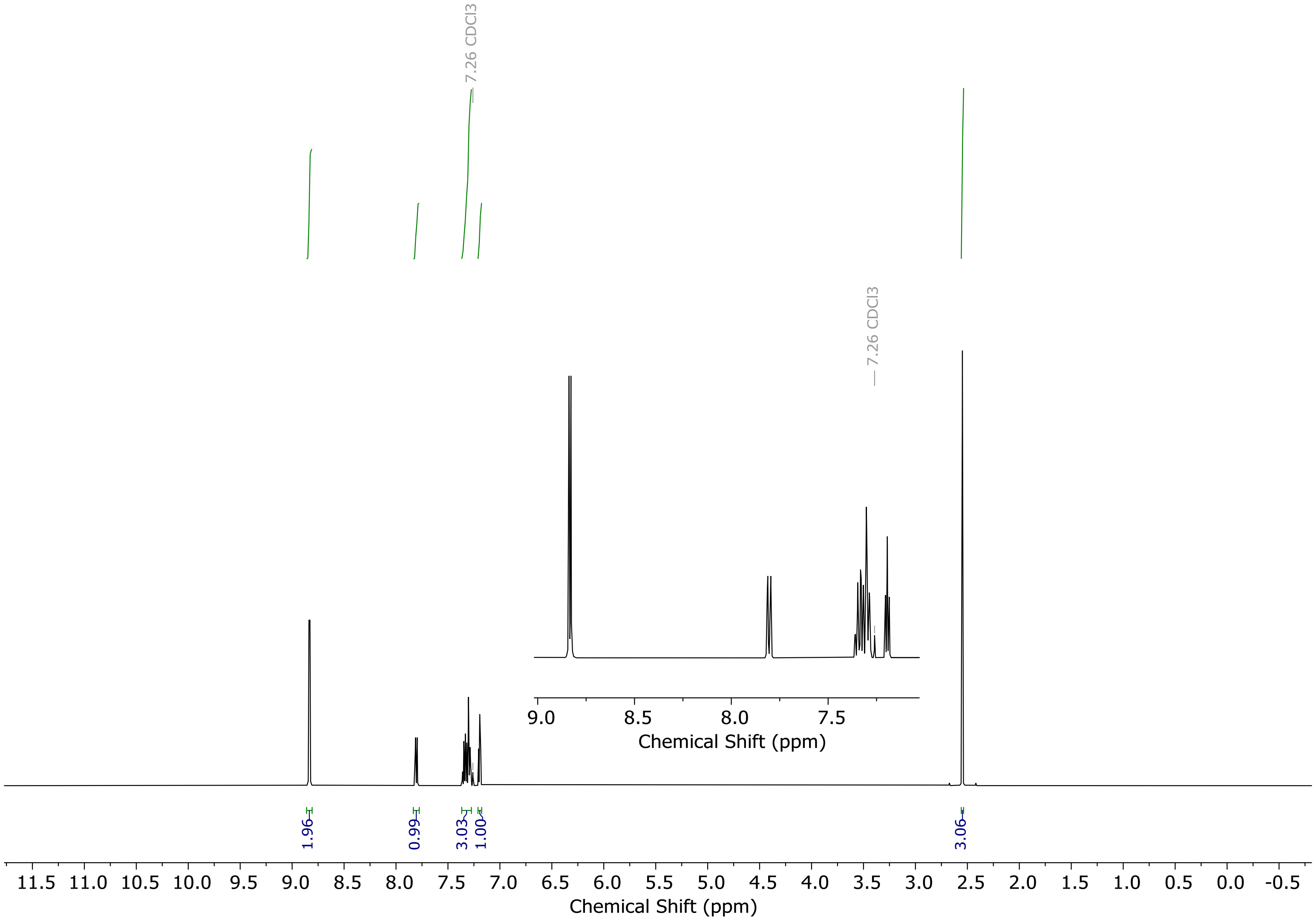

^{13}C NMR (126 MHz, $CDCl_3$) of Starting Material 8f

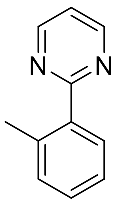

8f

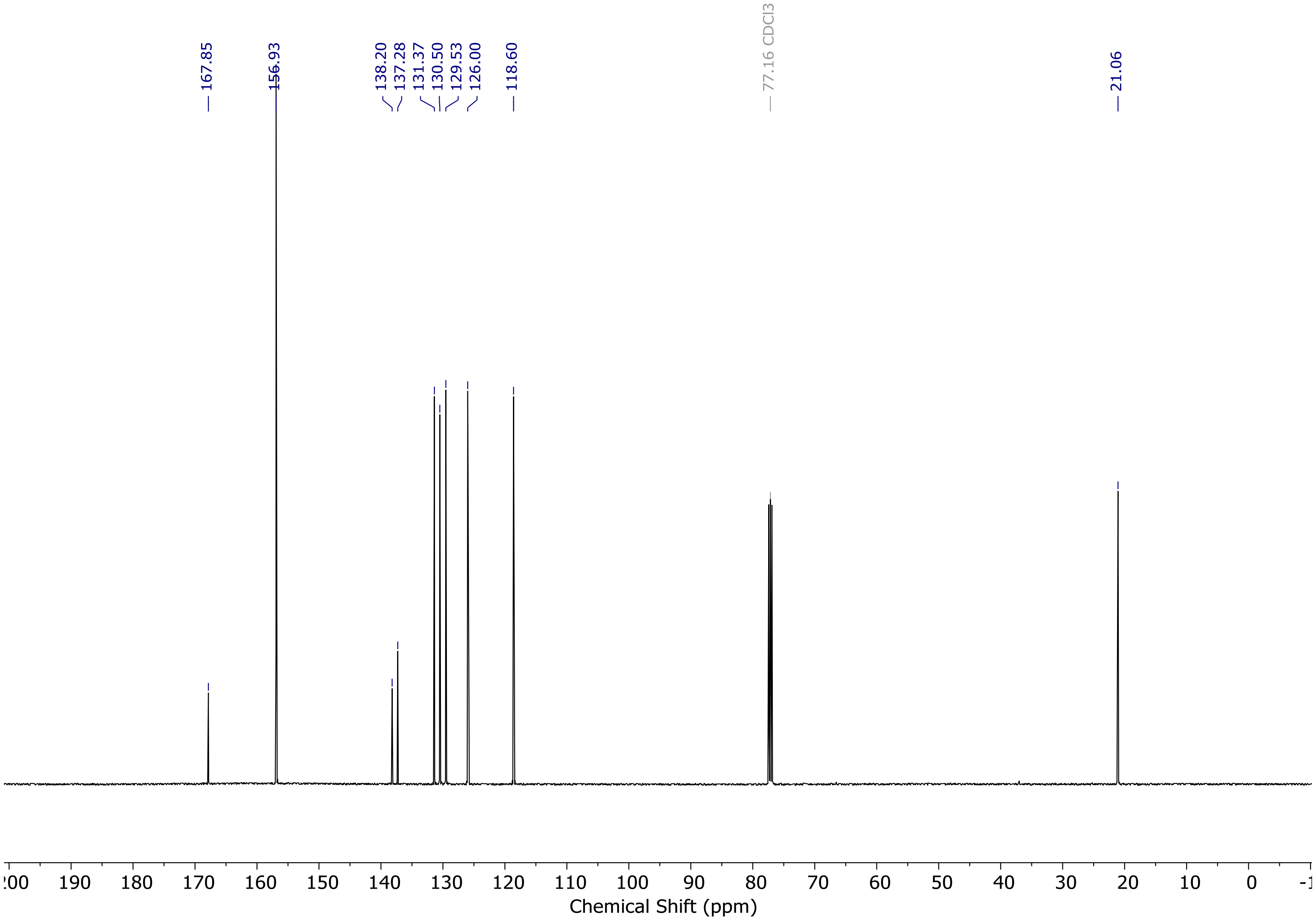

^{1}H NMR (400 MHz, $CDCl_3$) of Starting Material 8i

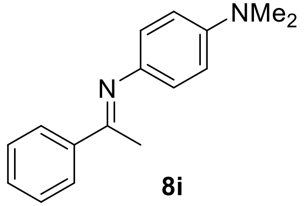

8i

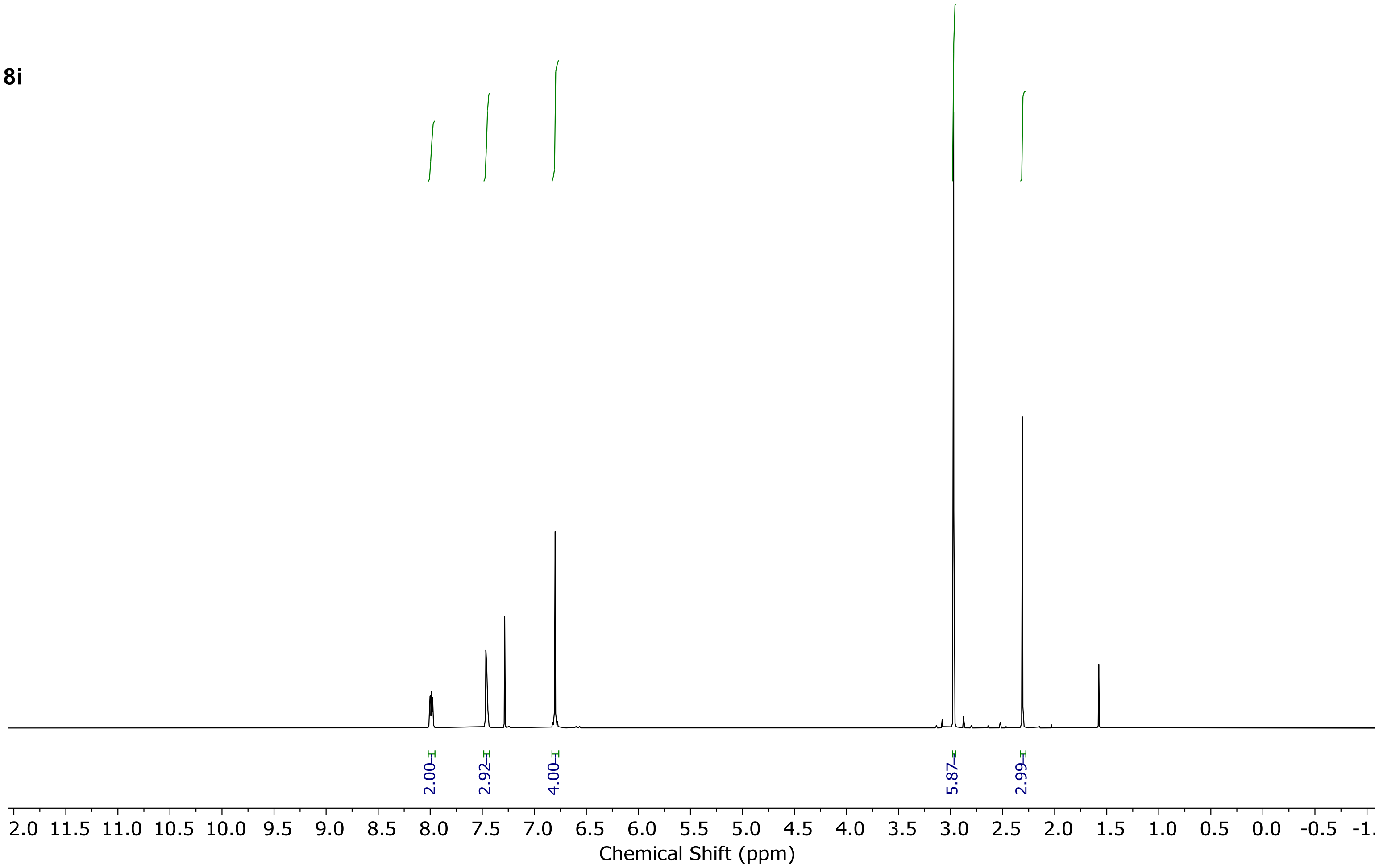

^{13}C NMR (126 MHz, $CDCl_3$) of Starting Material 8i

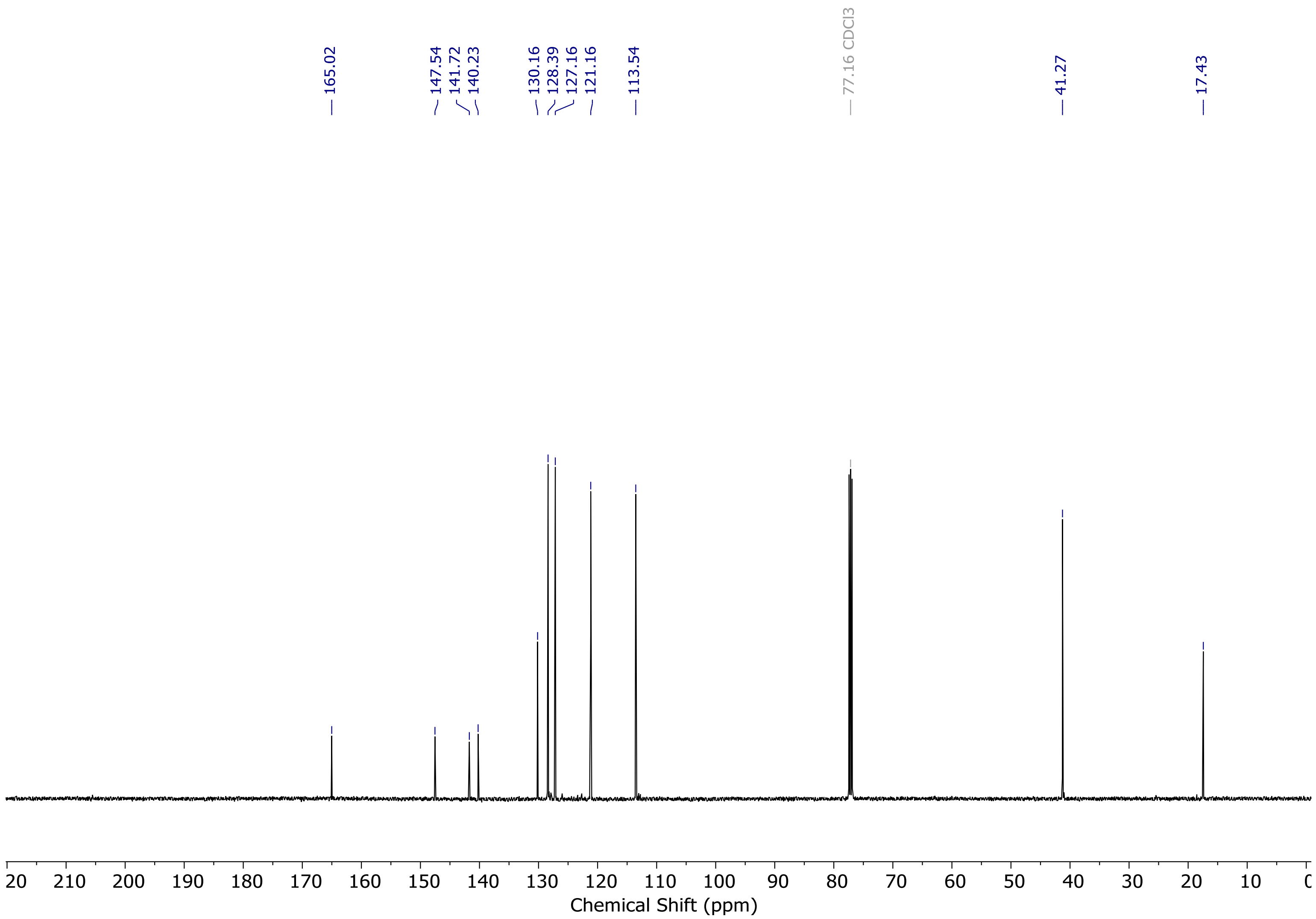

^{1}H NMR (500 MHz, $CDCl_3$) of Starting Material 8q

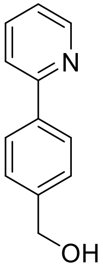

8q

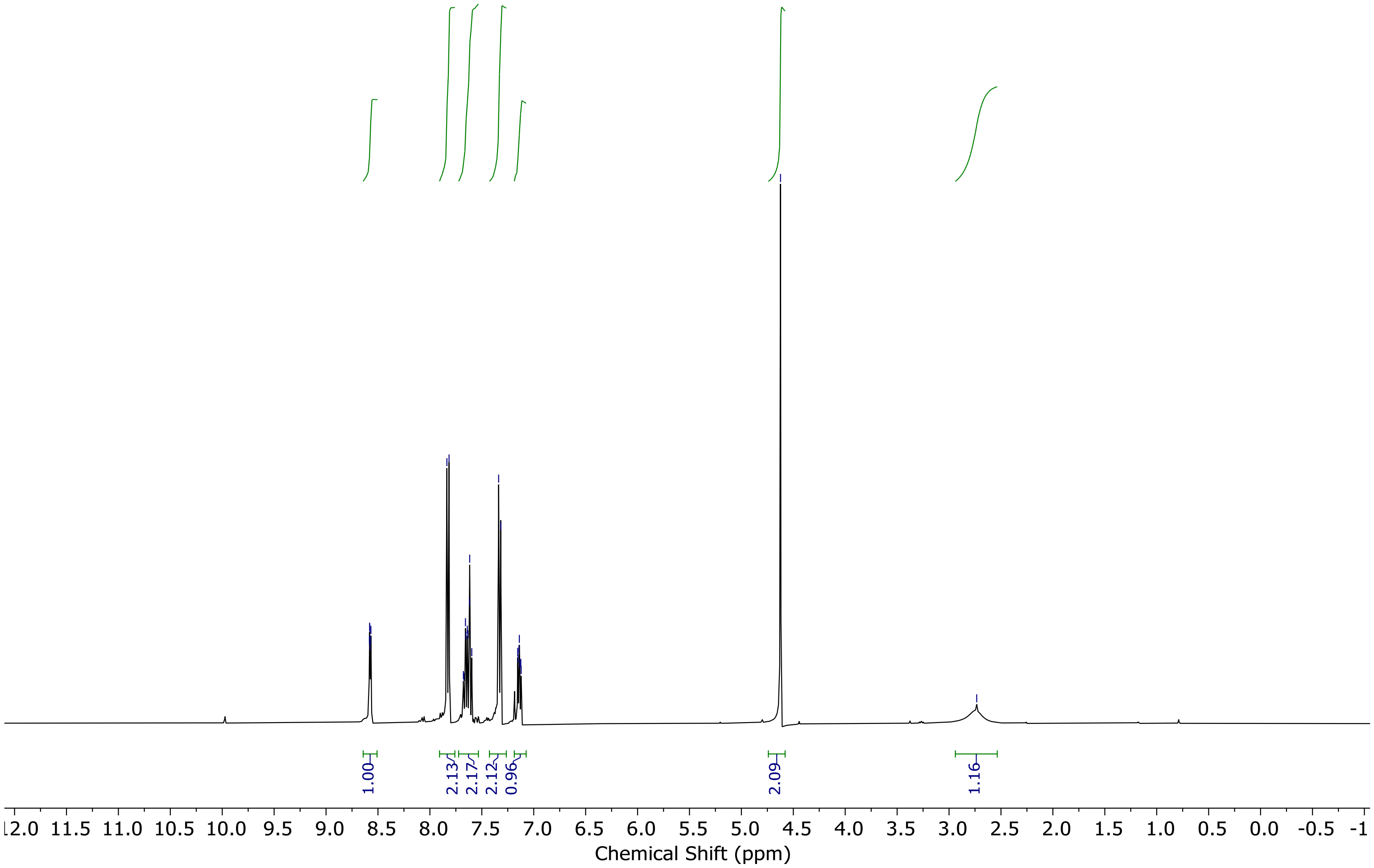

^{13}C NMR (126 MHz, $CDCl_3$) of Starting Material 8q

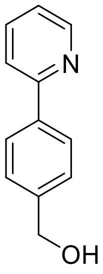

8q

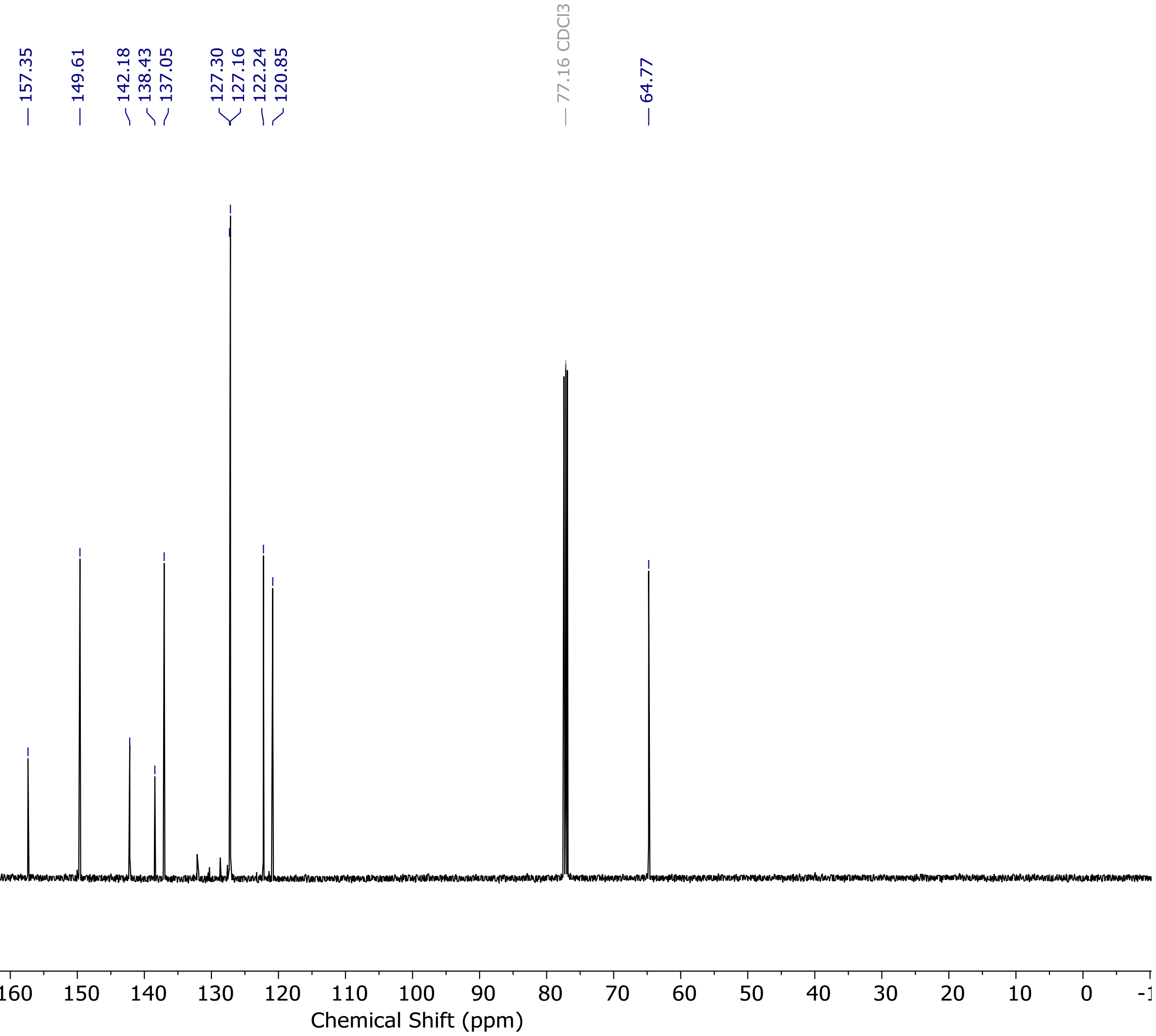

^{1}H NMR (500 MHz, $CDCl_3$) of Starting Material 12d

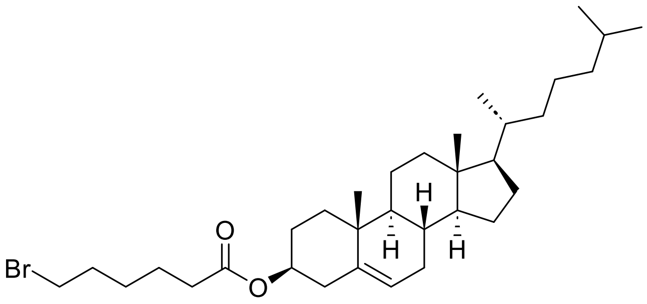

12d

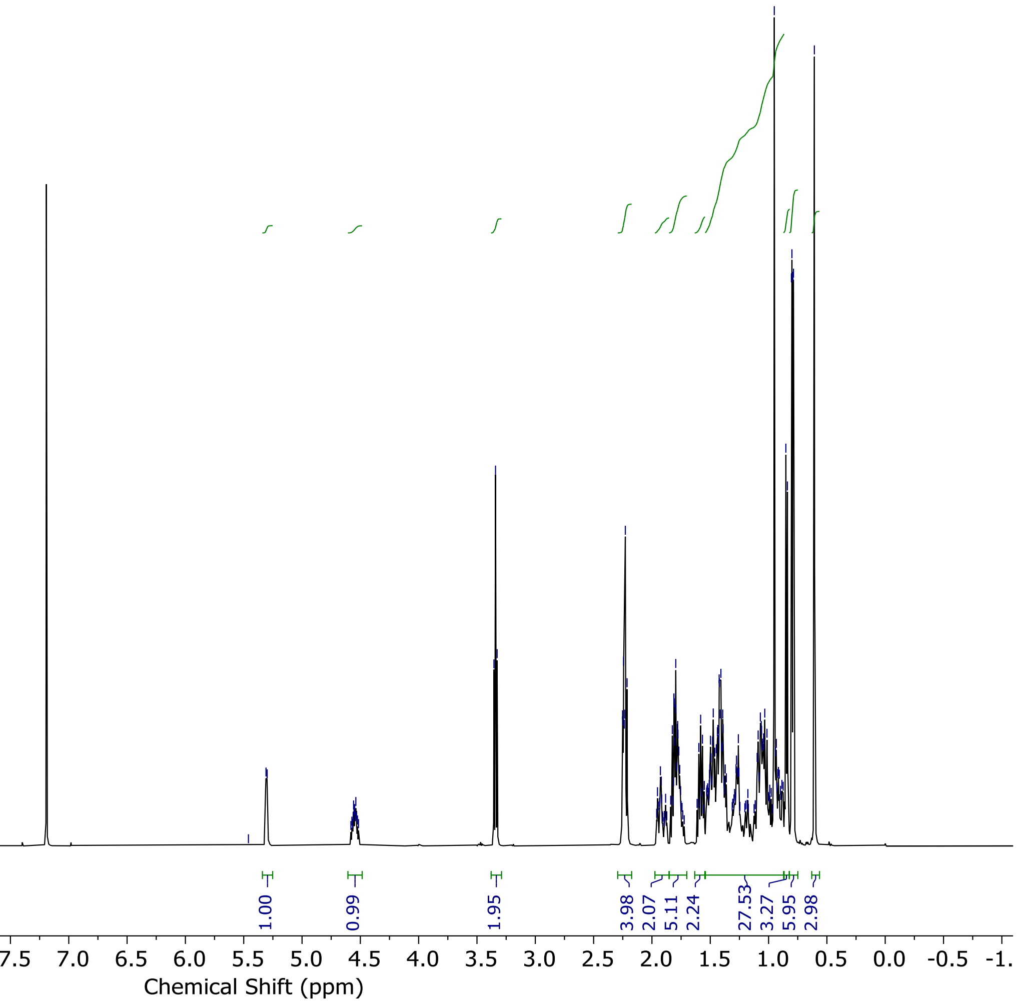

^{13}C NMR (126 MHz, $CDCl_3$) of Starting Material 12d

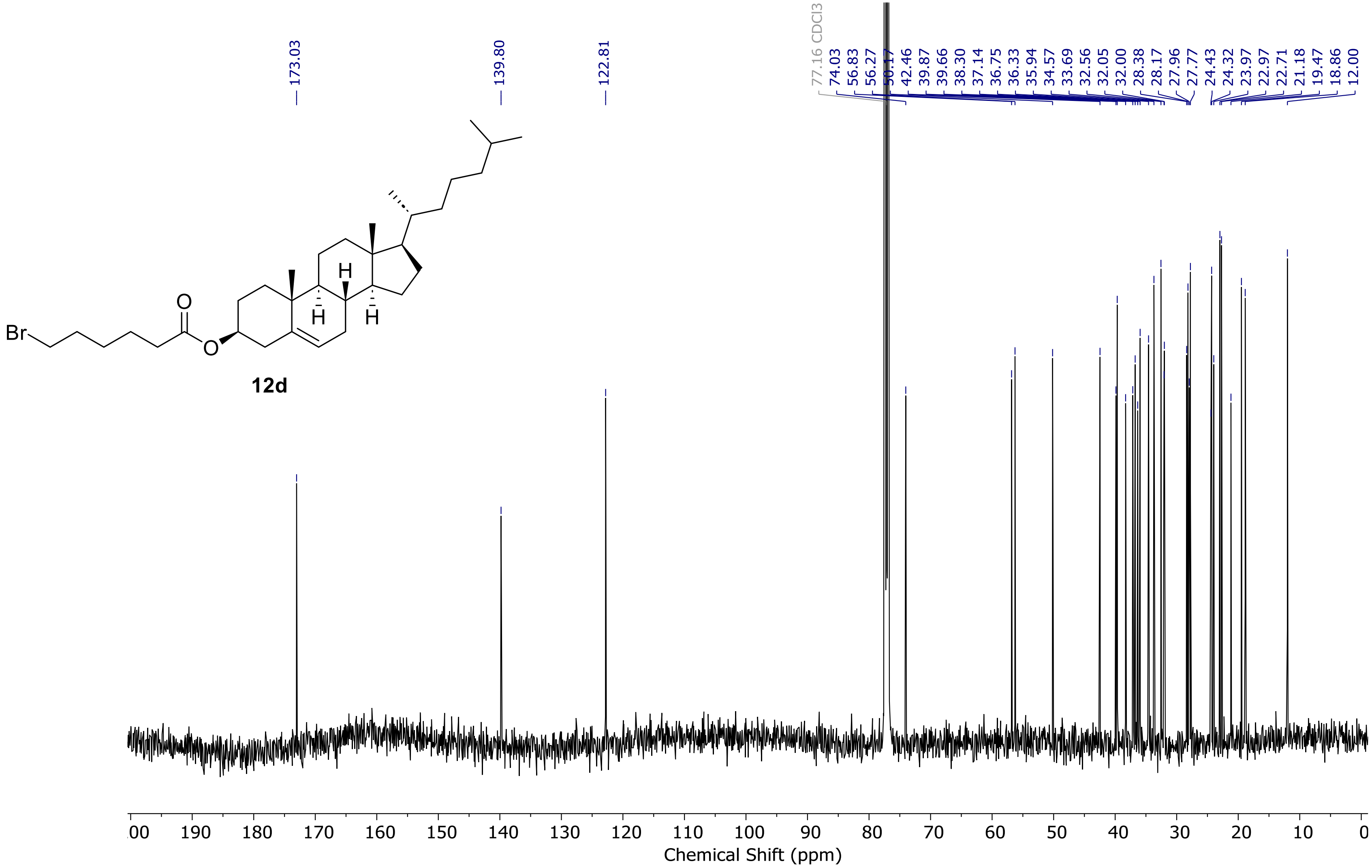

^{1}H NMR (500 MHz, $CDCl_3$) of 2-(5-methoxy-2-octylphenyl)pyridine 14a

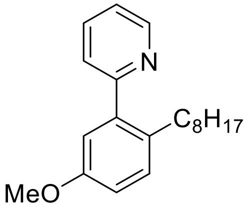

14a

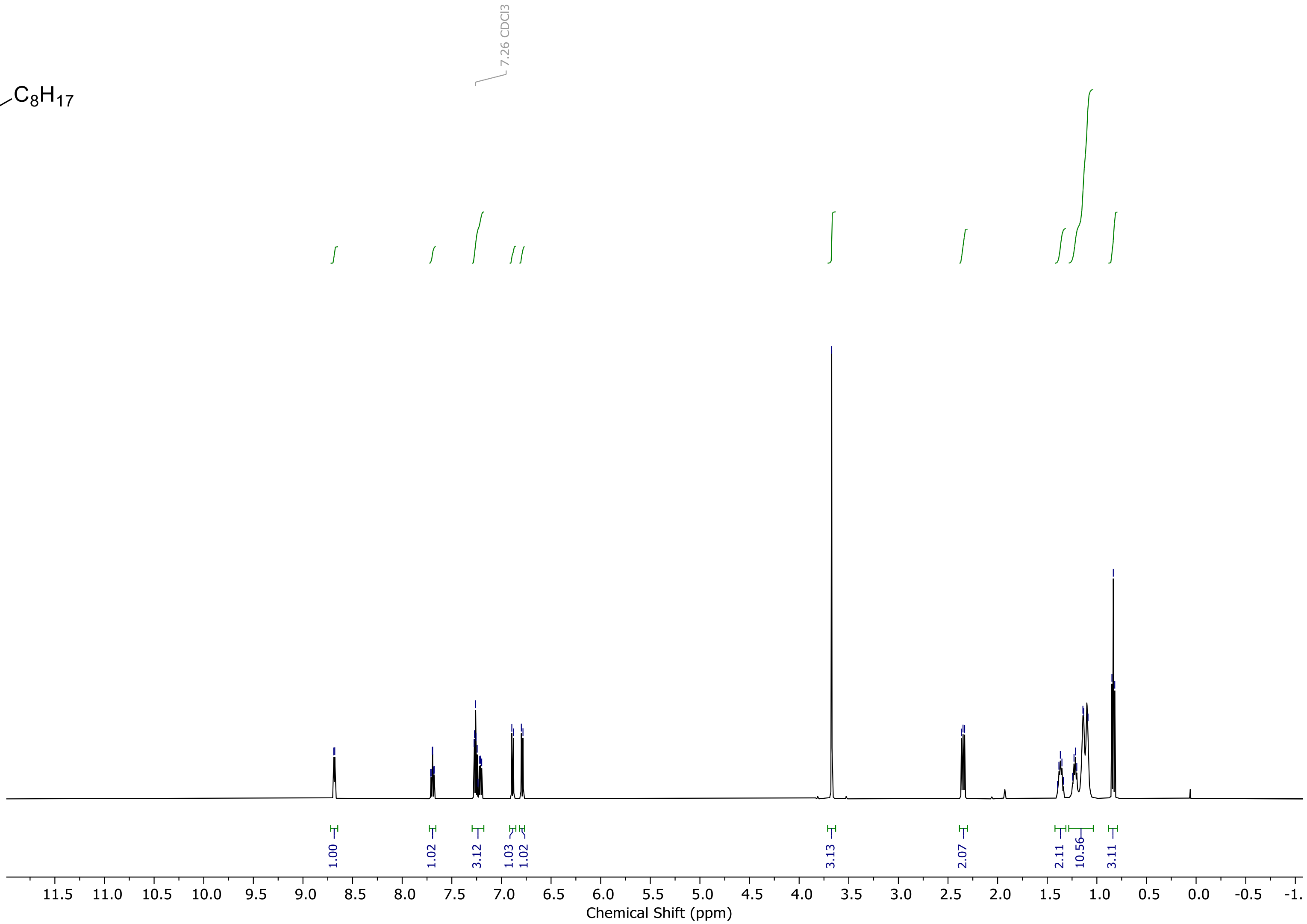

^{13}C NMR (126 MHz, $CDCl_3$) of 2-(5-methoxy-2-octylphenyl)pyridine 14a

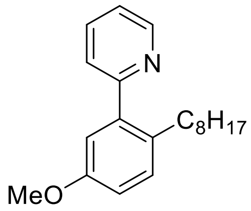

14a

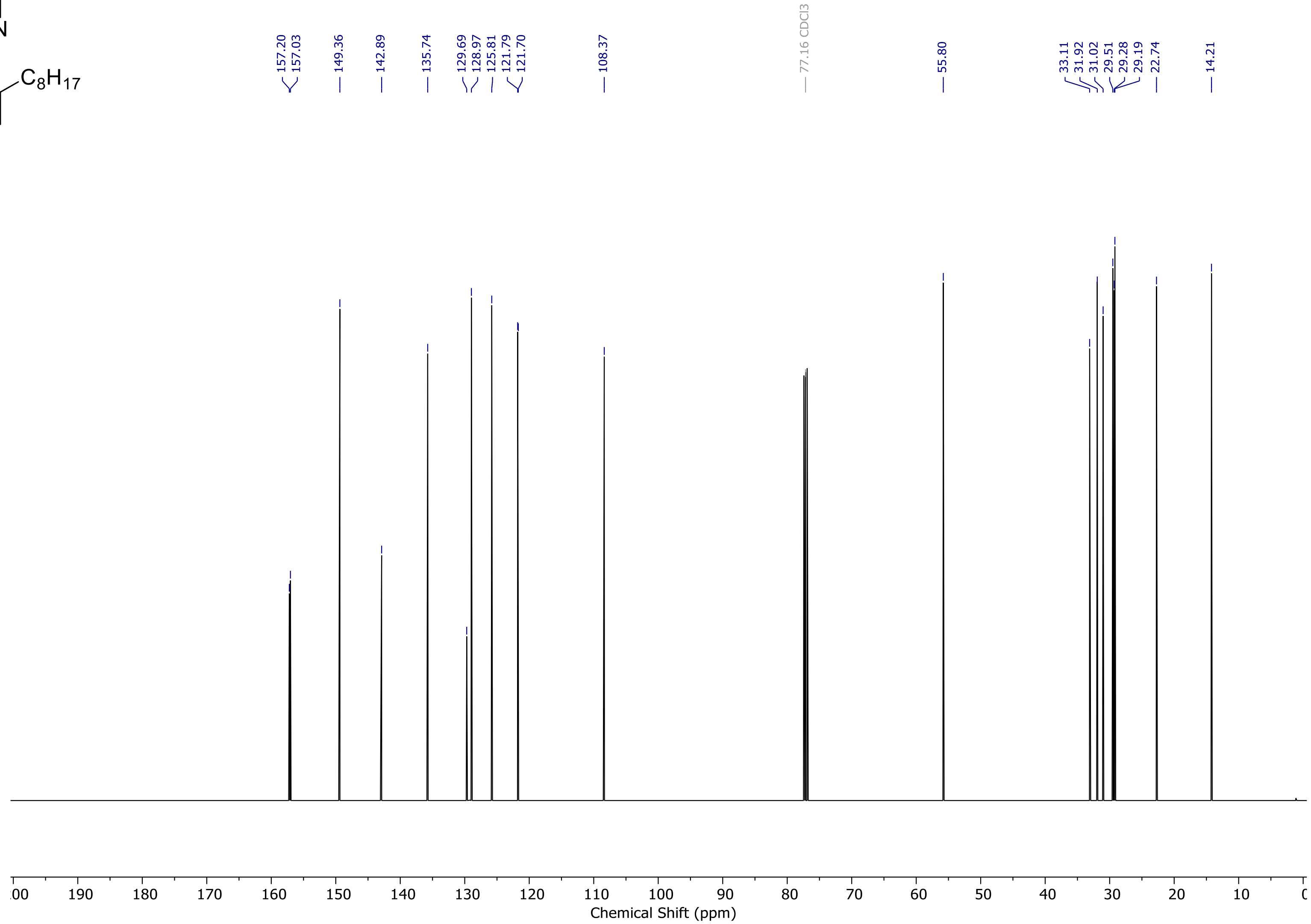

^{1}H NMR (500 MHz, CDCl₃) of 2-(2-methyl-6-octylphenyl)pyrimidine 14b

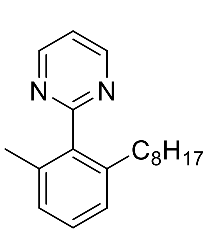

14b

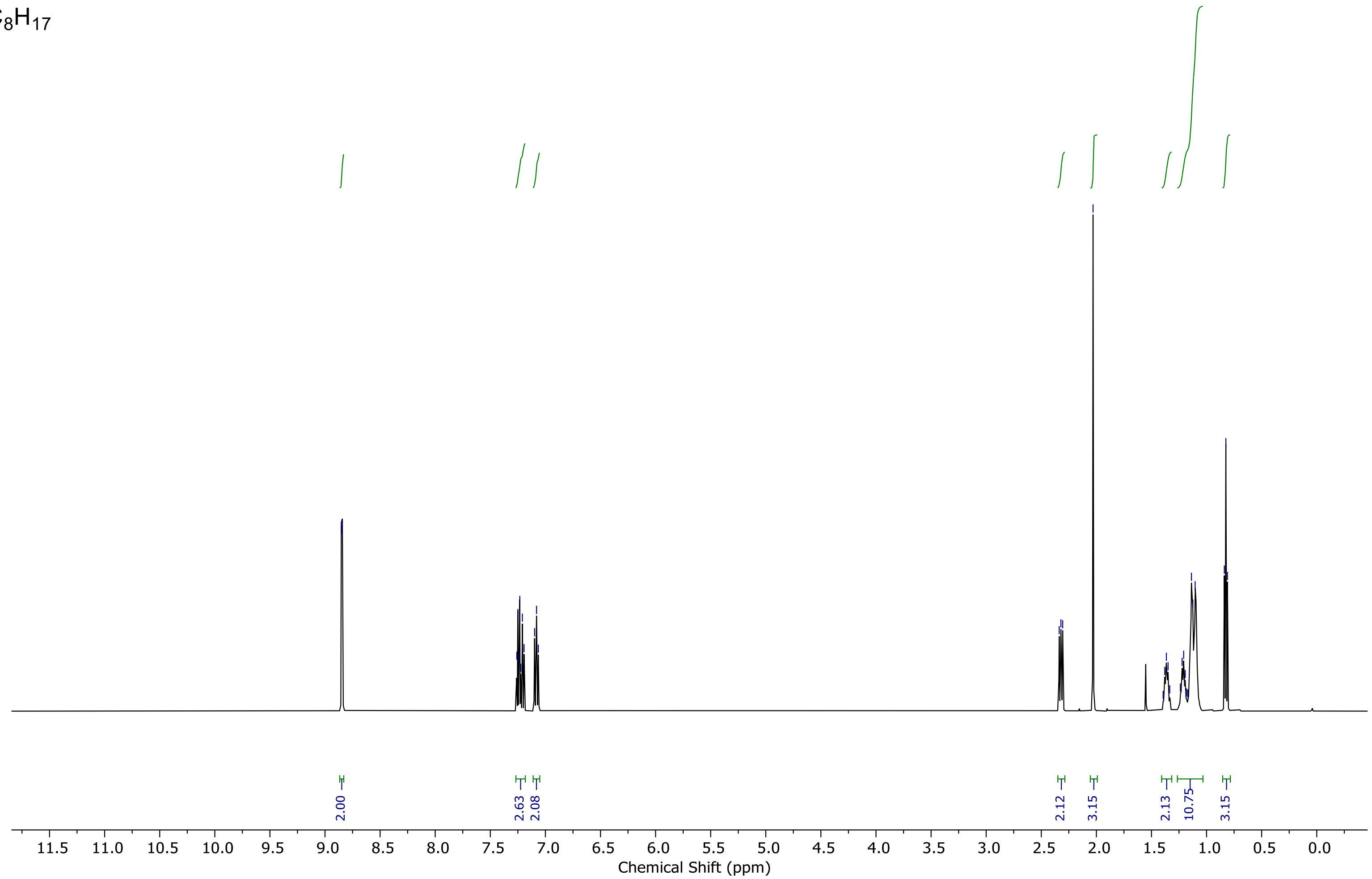

^{13}C NMR (126 MHz, CDCl$_3$) of 2-(2-methyl-6-octylphenyl)pyrimidine 14b

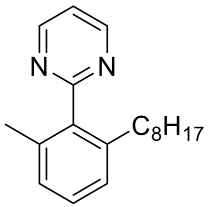

14b

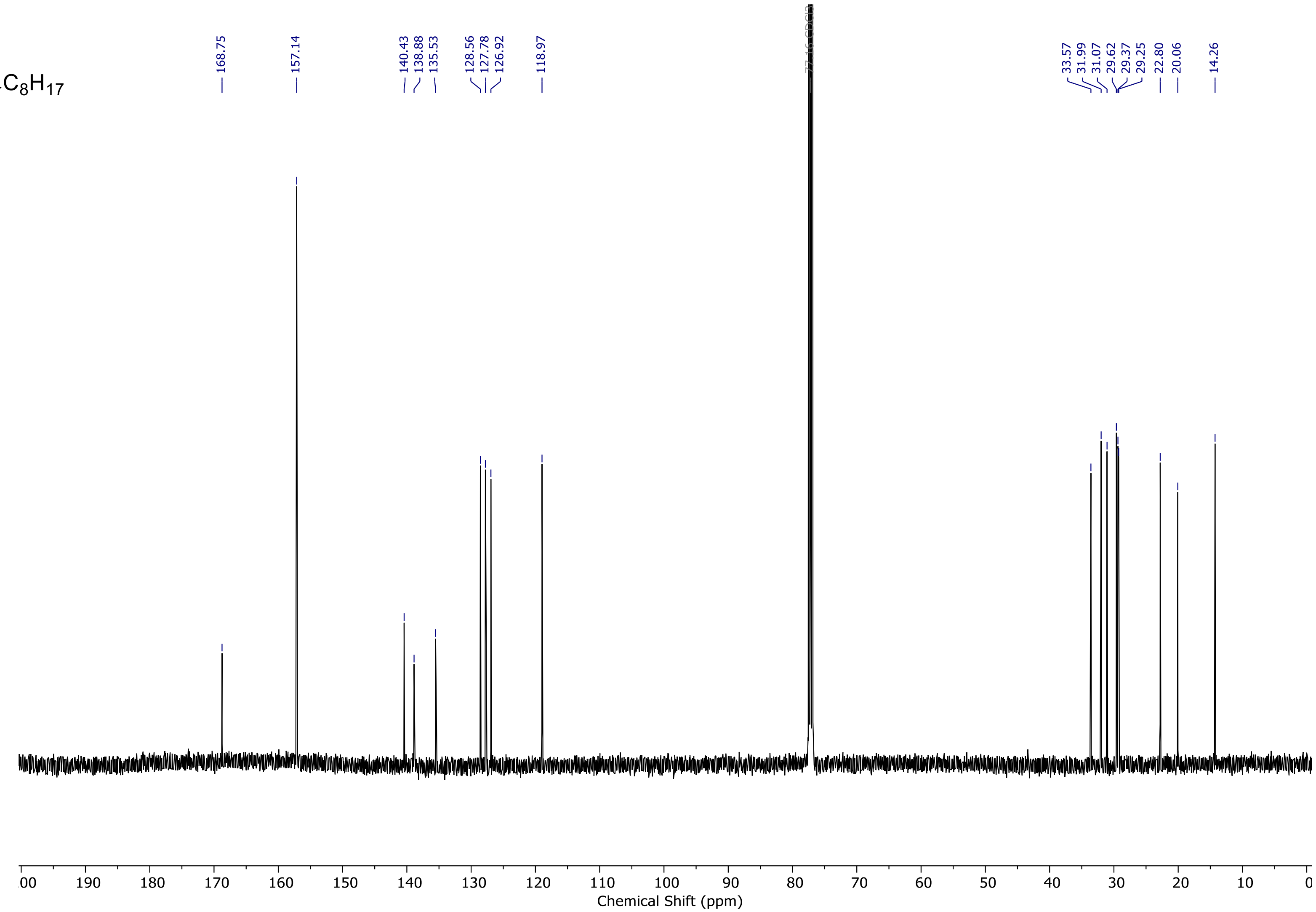

^{1}H NMR (400 MHz, $CDCl_3$) of 1-(2-octylphenyl)isoquinoline 14c

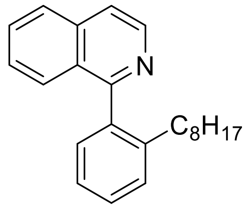

14c

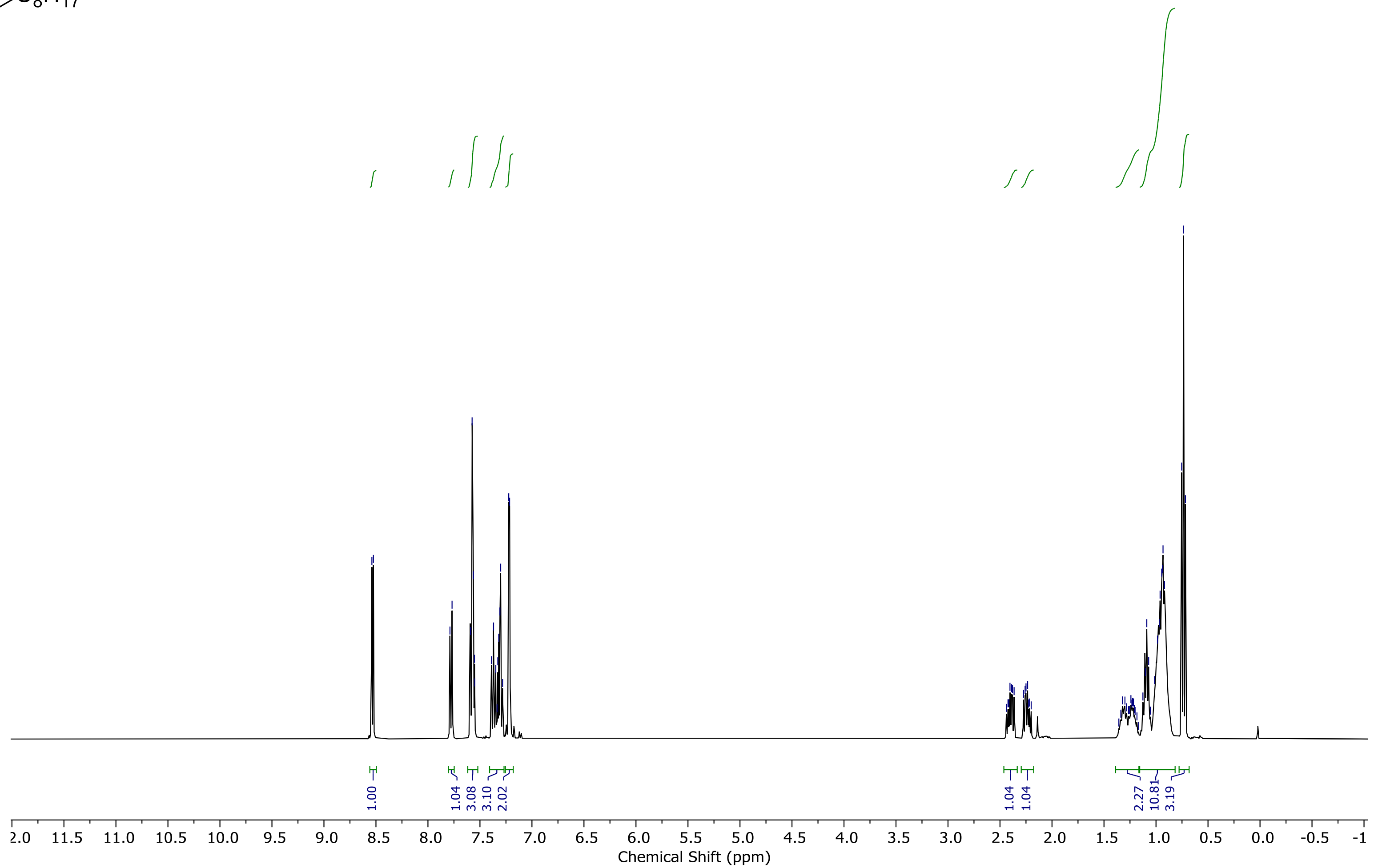

^{13}C NMR (101 MHz, CDCl$_3$) of 1-(2-octylphenyl)isoquinoline 14c

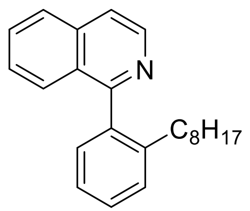

14c

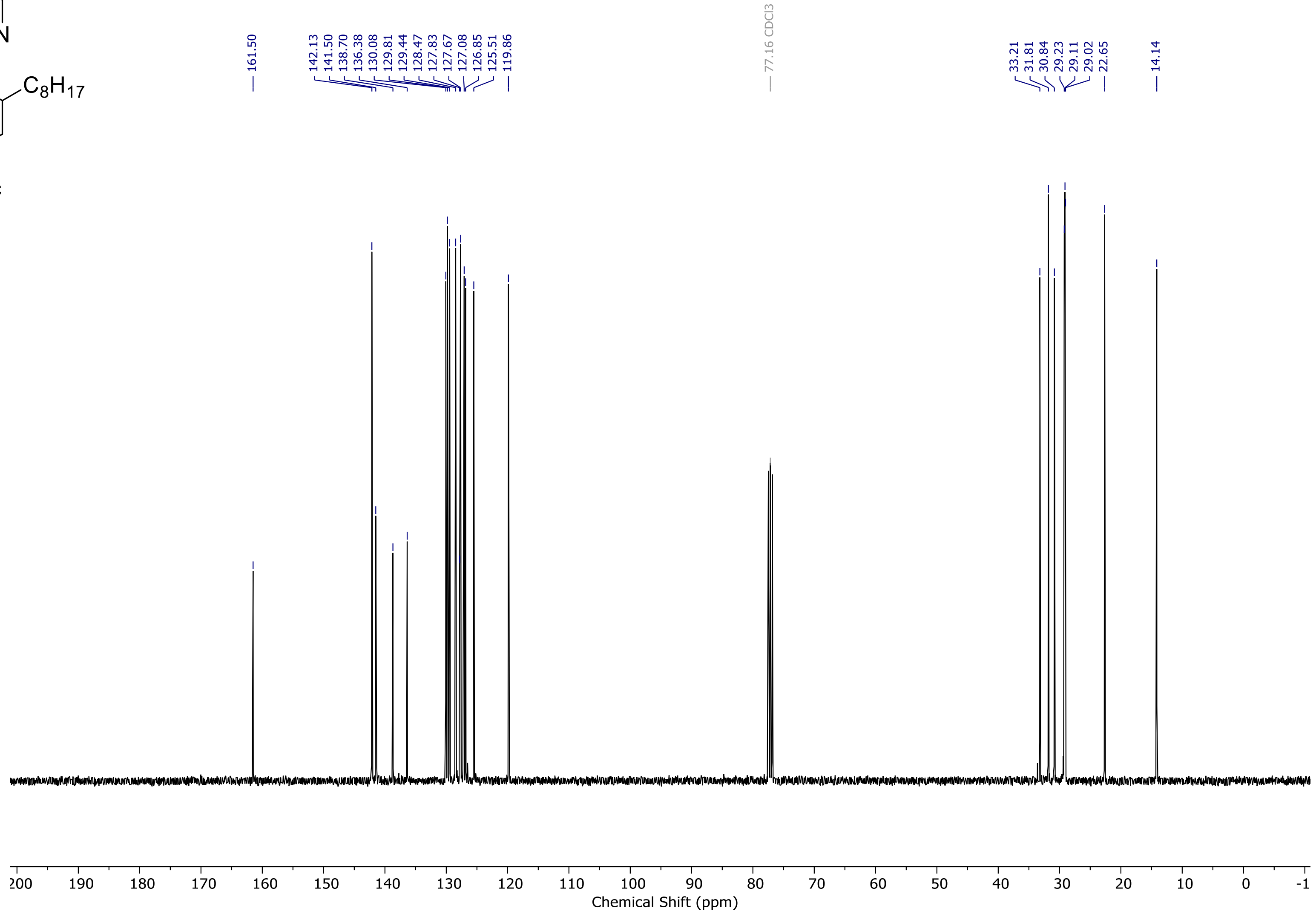

^{1}H NMR (400 MHz, CDCl$_3$) of 1-(2-octylphenyl)-1H-pyrazole 14d

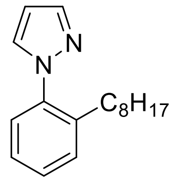

14d

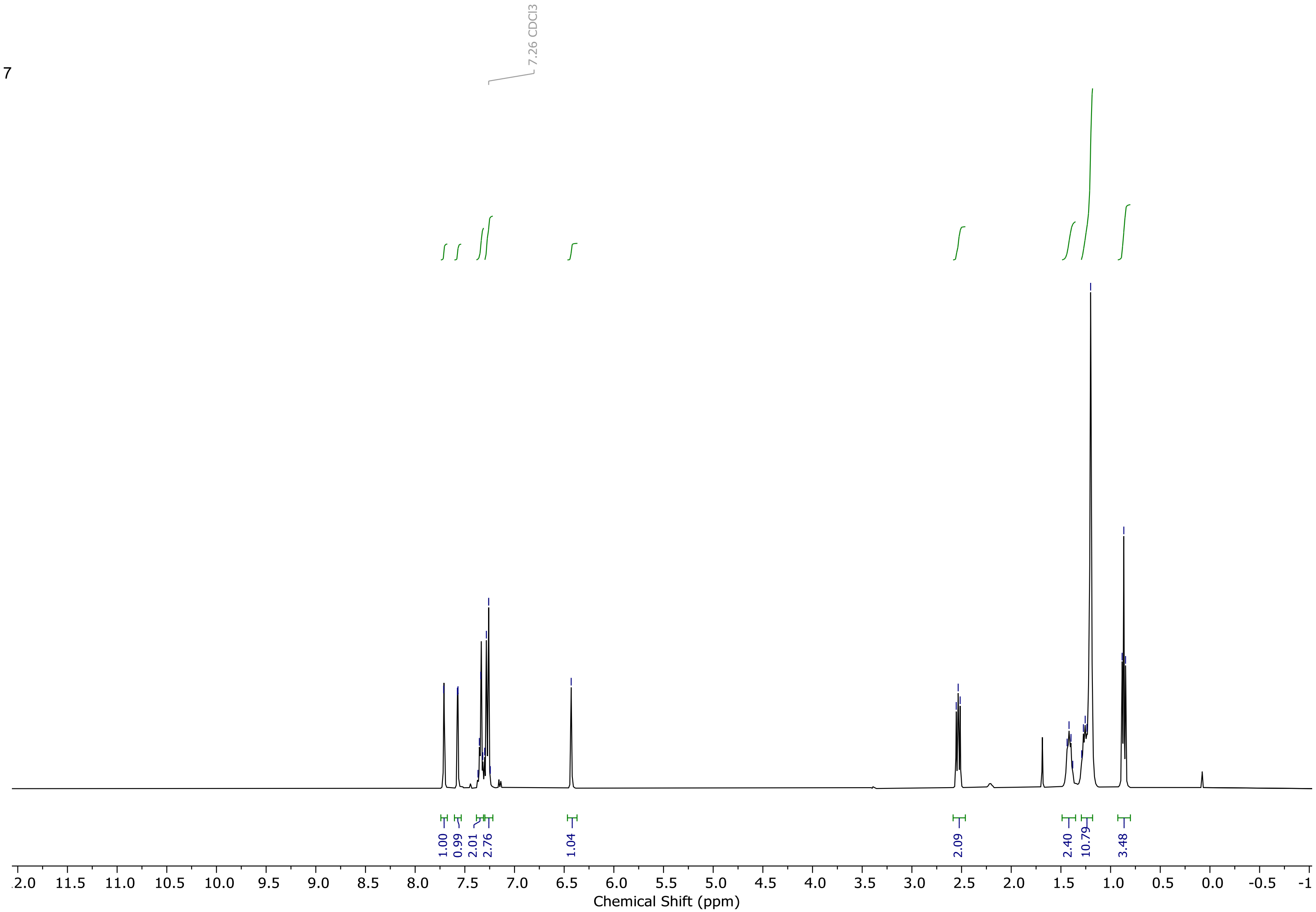

^{13}C NMR (101 MHz, $CDCl_3$) of 1-(2-octylphenyl)-1H-pyrazole 14d

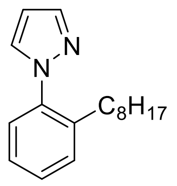

14d

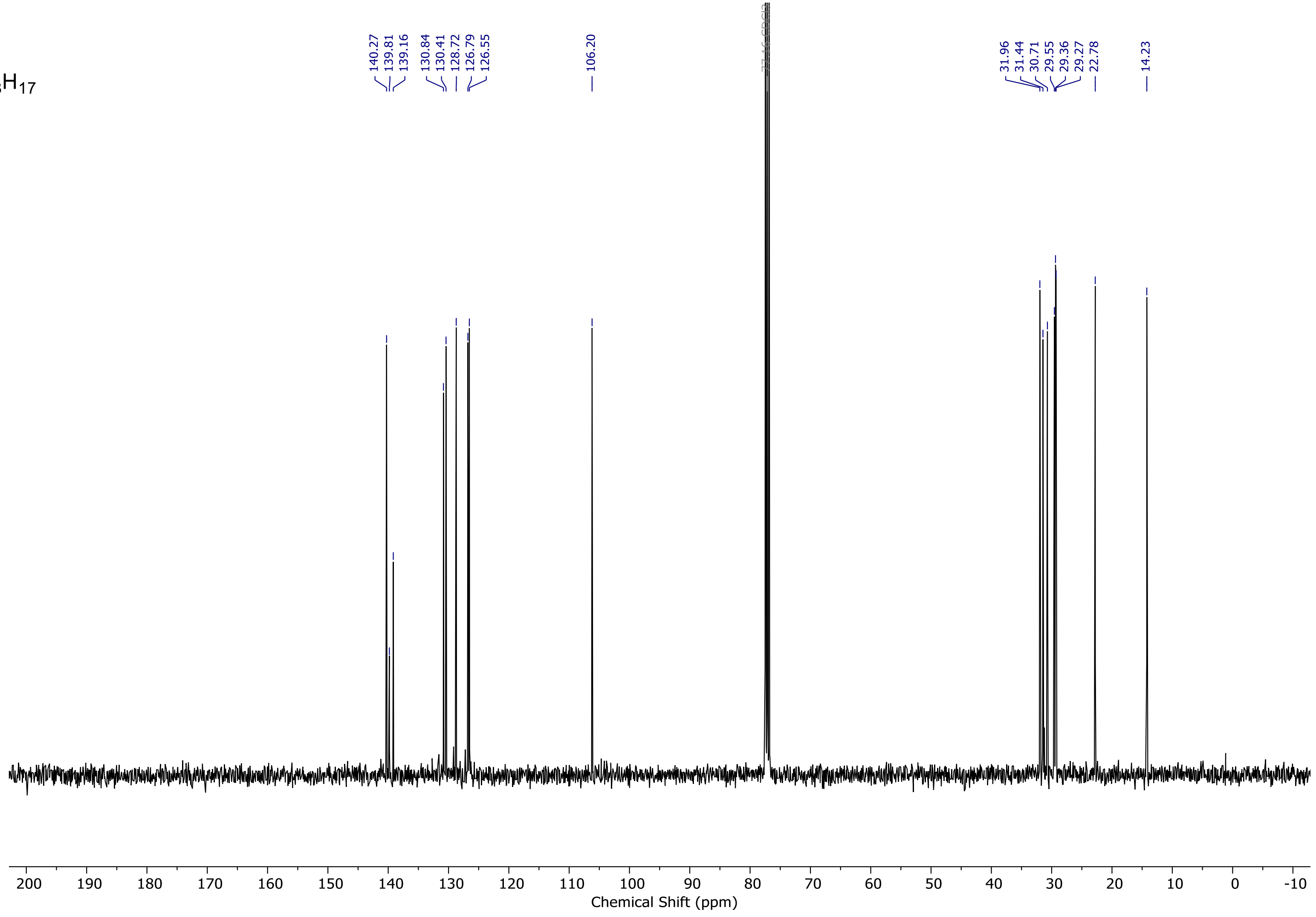

^{1}H NMR (500 MHz, $CDCl_3$) of [diazepam]-[*n*-octyl] 14e

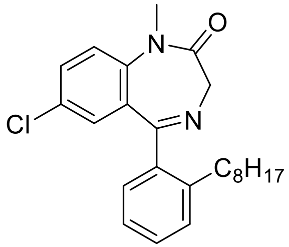

14e

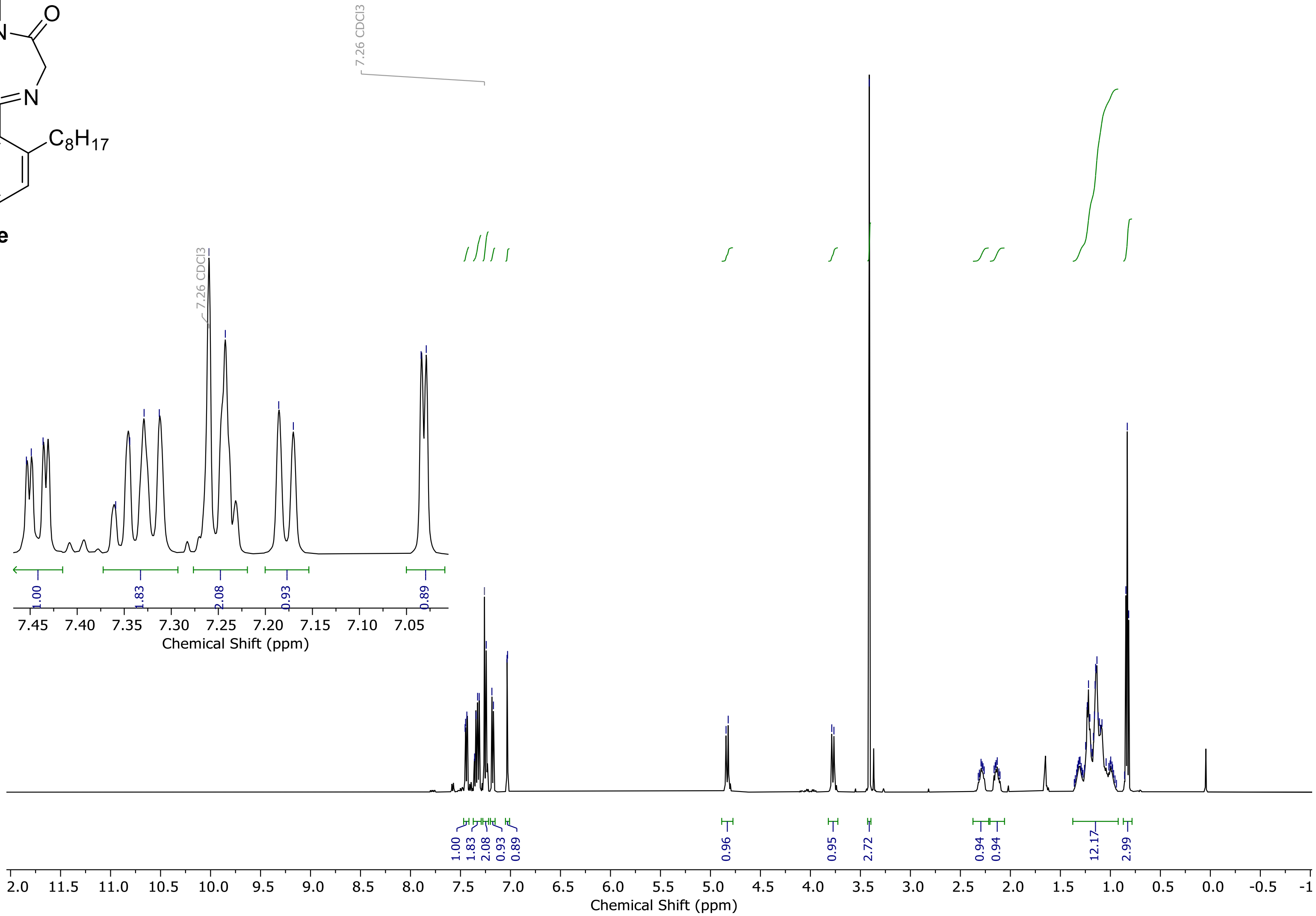

^{13}C NMR (126 MHz, CDCl$_3$) of [diazepam]-[*n*-octyl] 14e

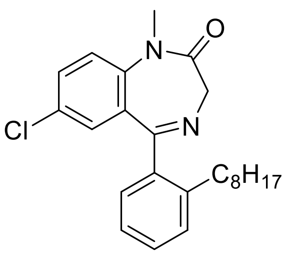

14e

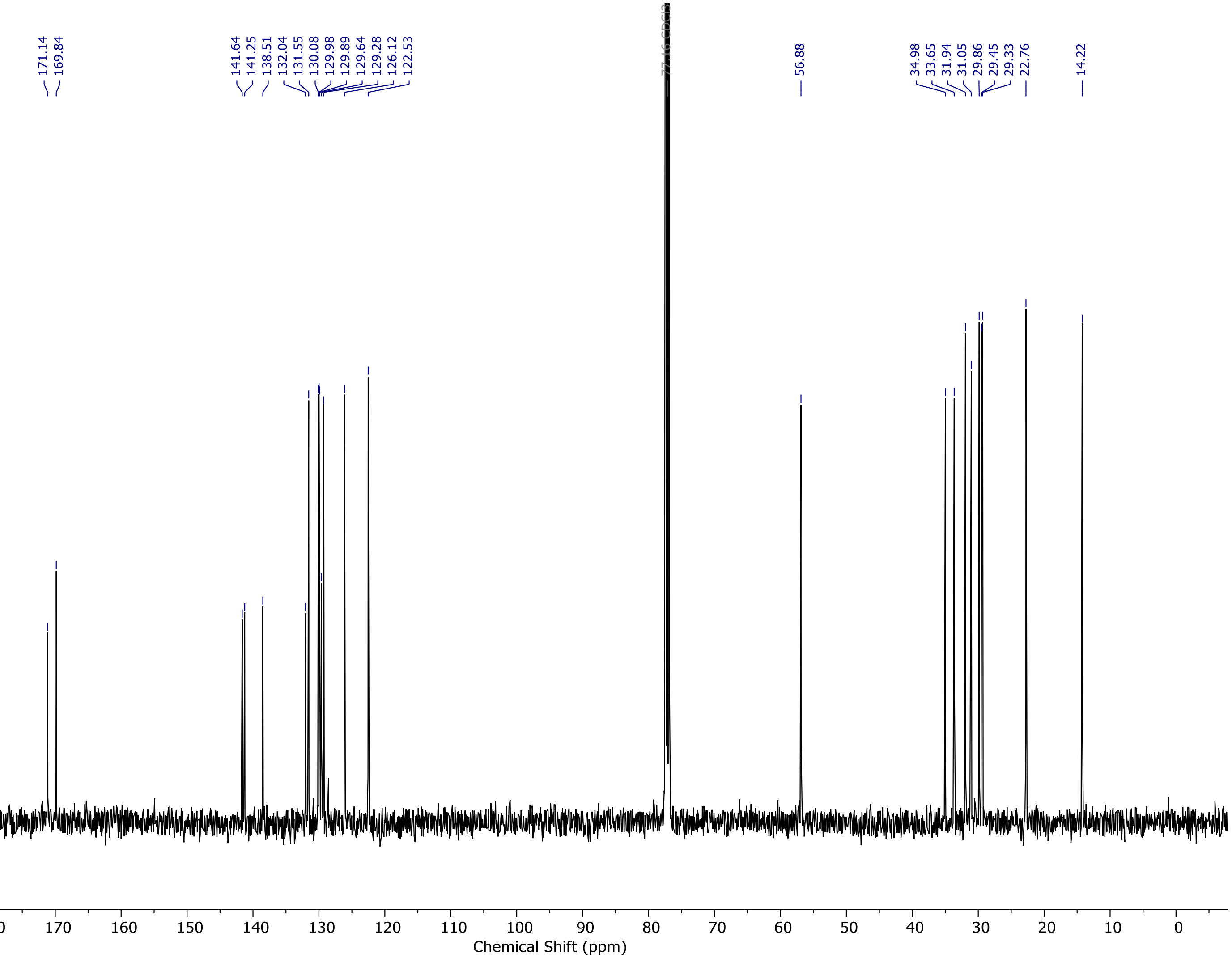

^{1}H NMR (500 MHz, $CDCl_3$) of 1-(2-octylphenyl)ethan-1-one 14f

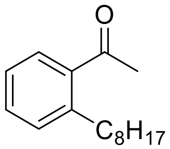

14f

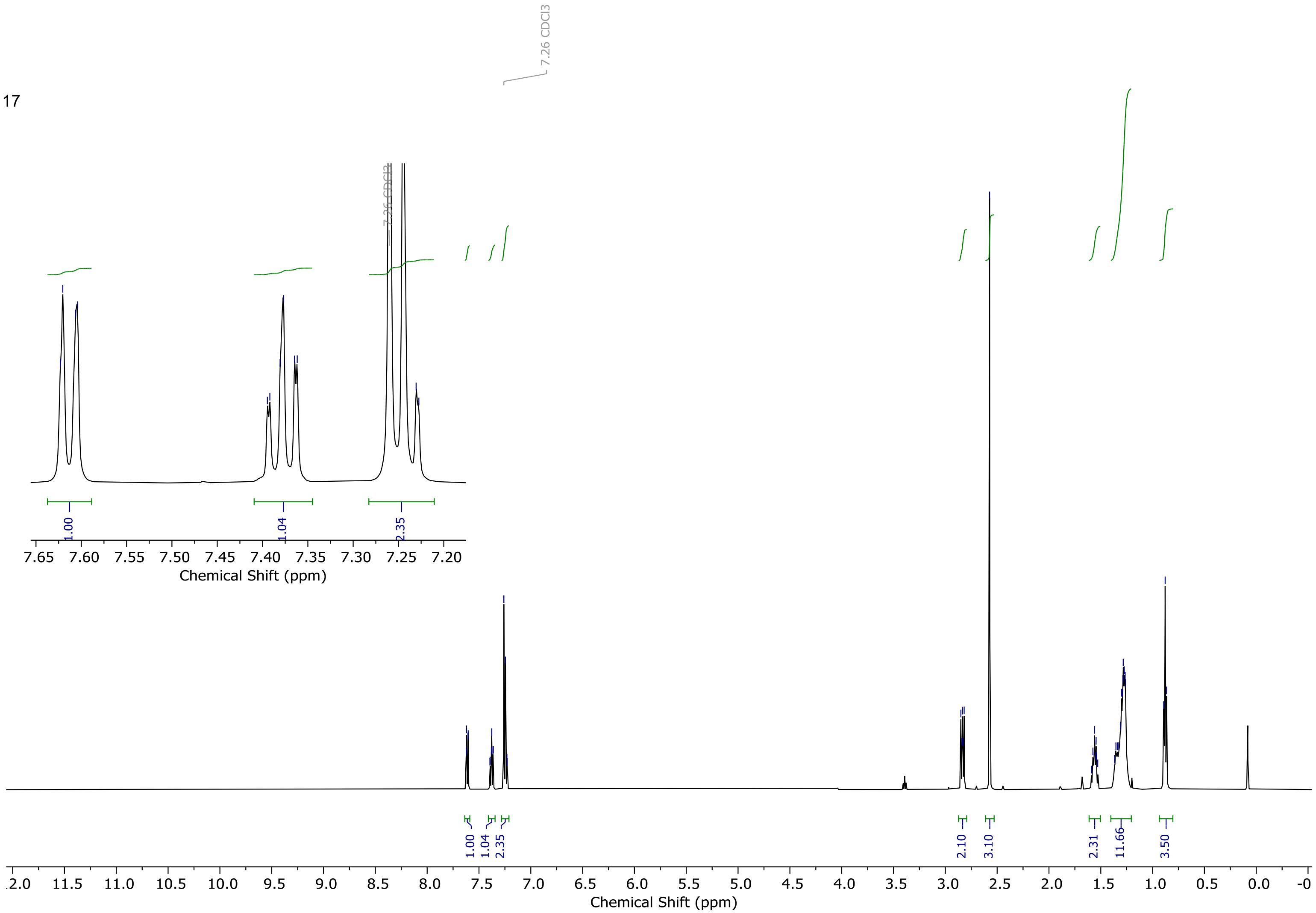

^{13}C NMR (101 MHz, $CDCl_3$) of 1-(2-octylphenyl)ethan-1-one 14f

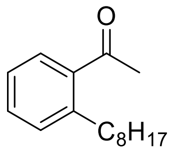

14f

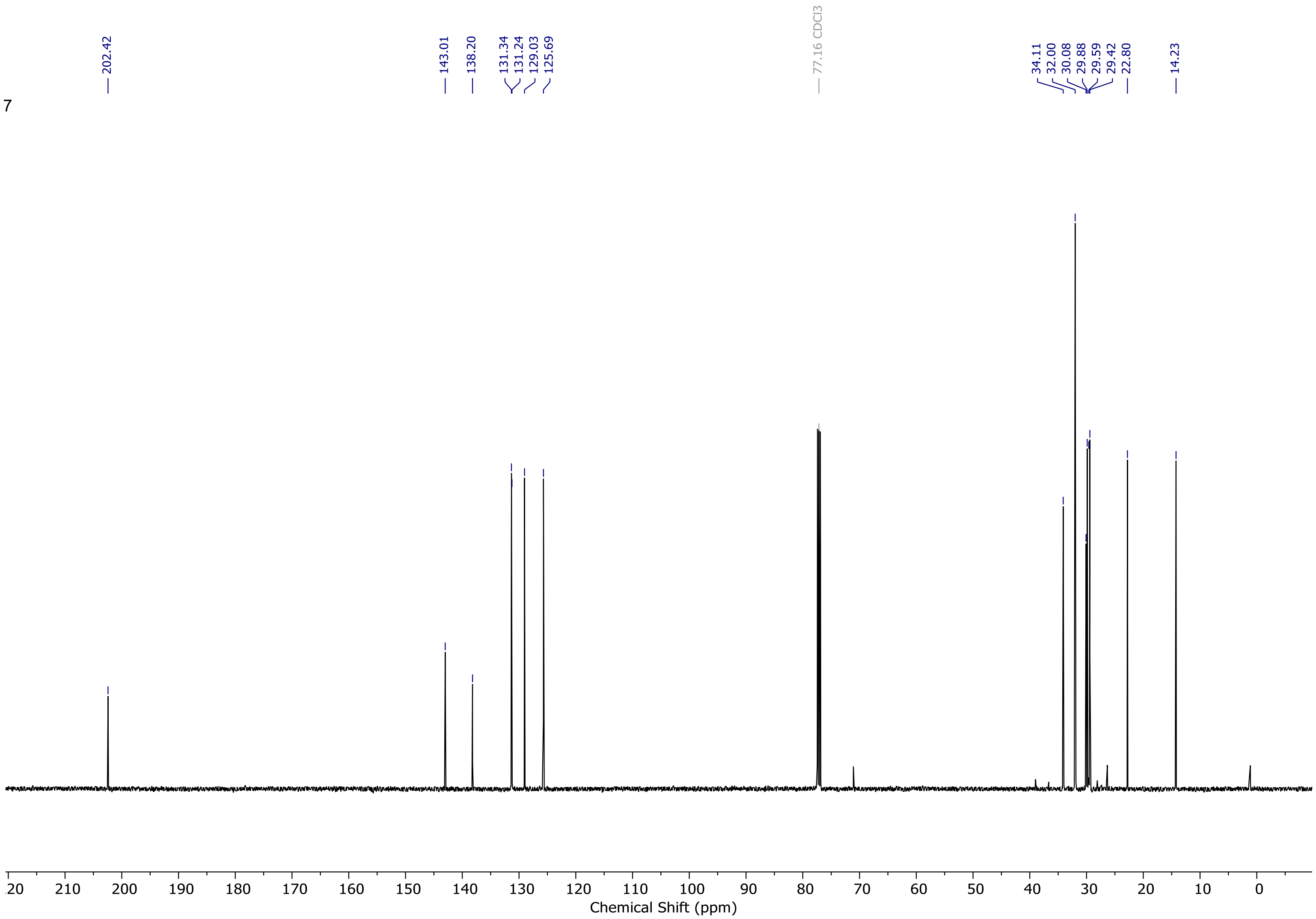

^{1}H NMR (400 MHz, $CDCl_3$) of 2-(2-methyl-6-octylphenyl)pyridine 14g

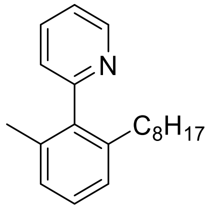

14g

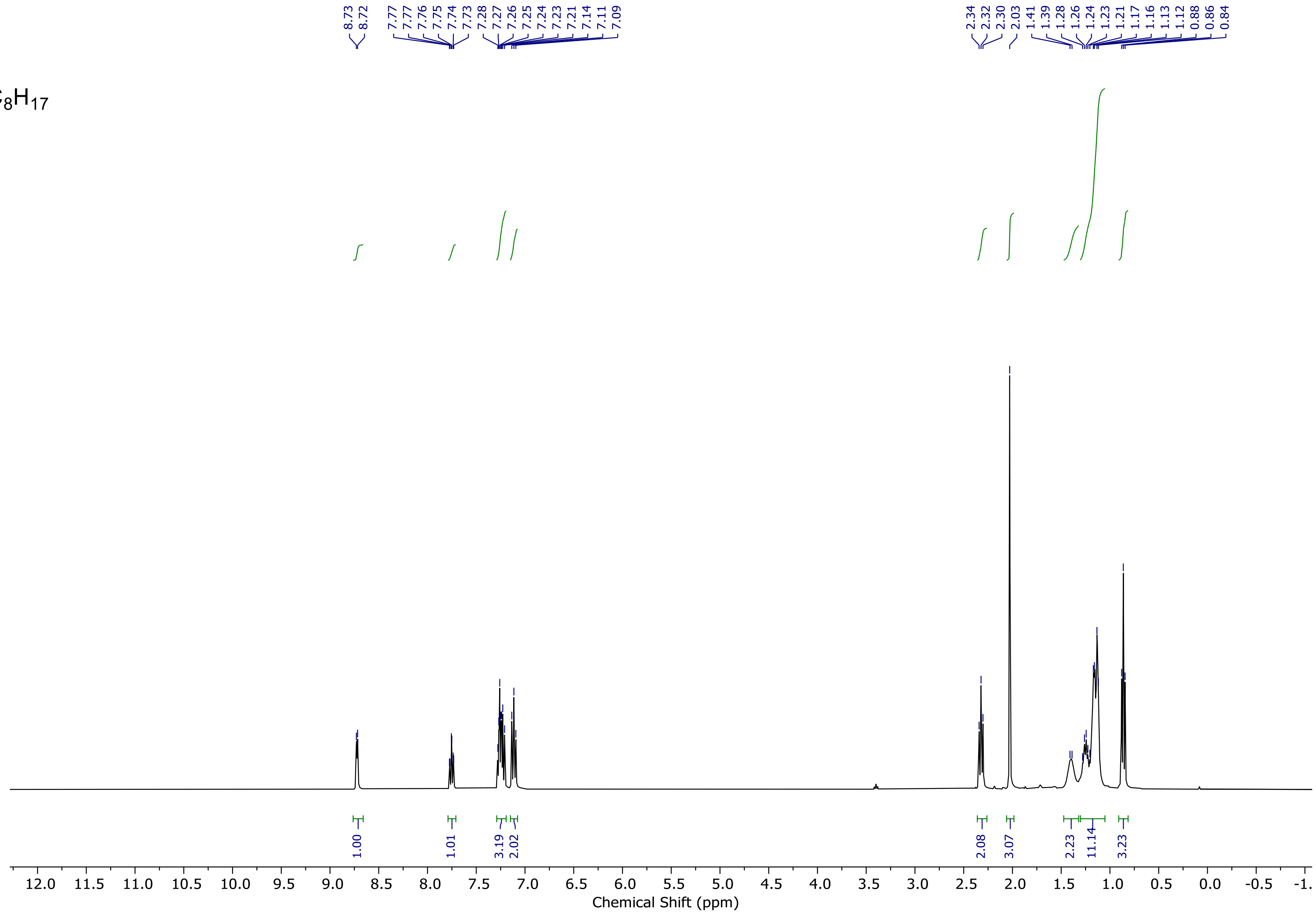

^{13}C NMR (101 MHz, CDCl₃) of 2-(2-methyl-6-octylphenyl)pyridine 14g

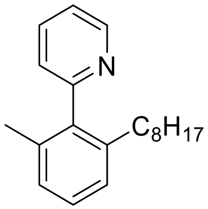

14g

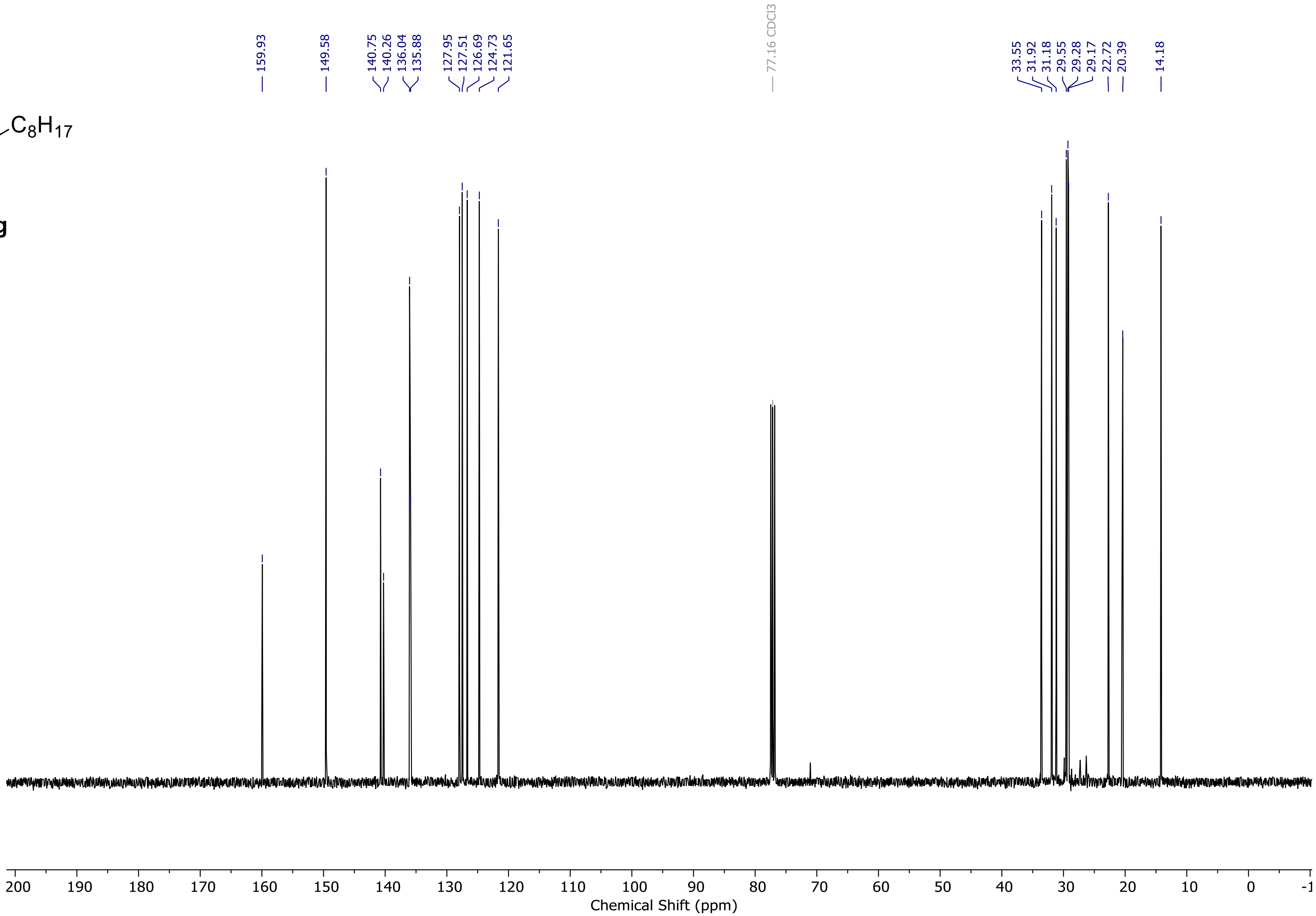

^{1}H NMR (500 MHz, $CDCl_3$) of ethanol--2-(2-methyl-6-octylphenyl)pyridine 14h

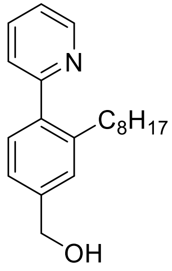

14h

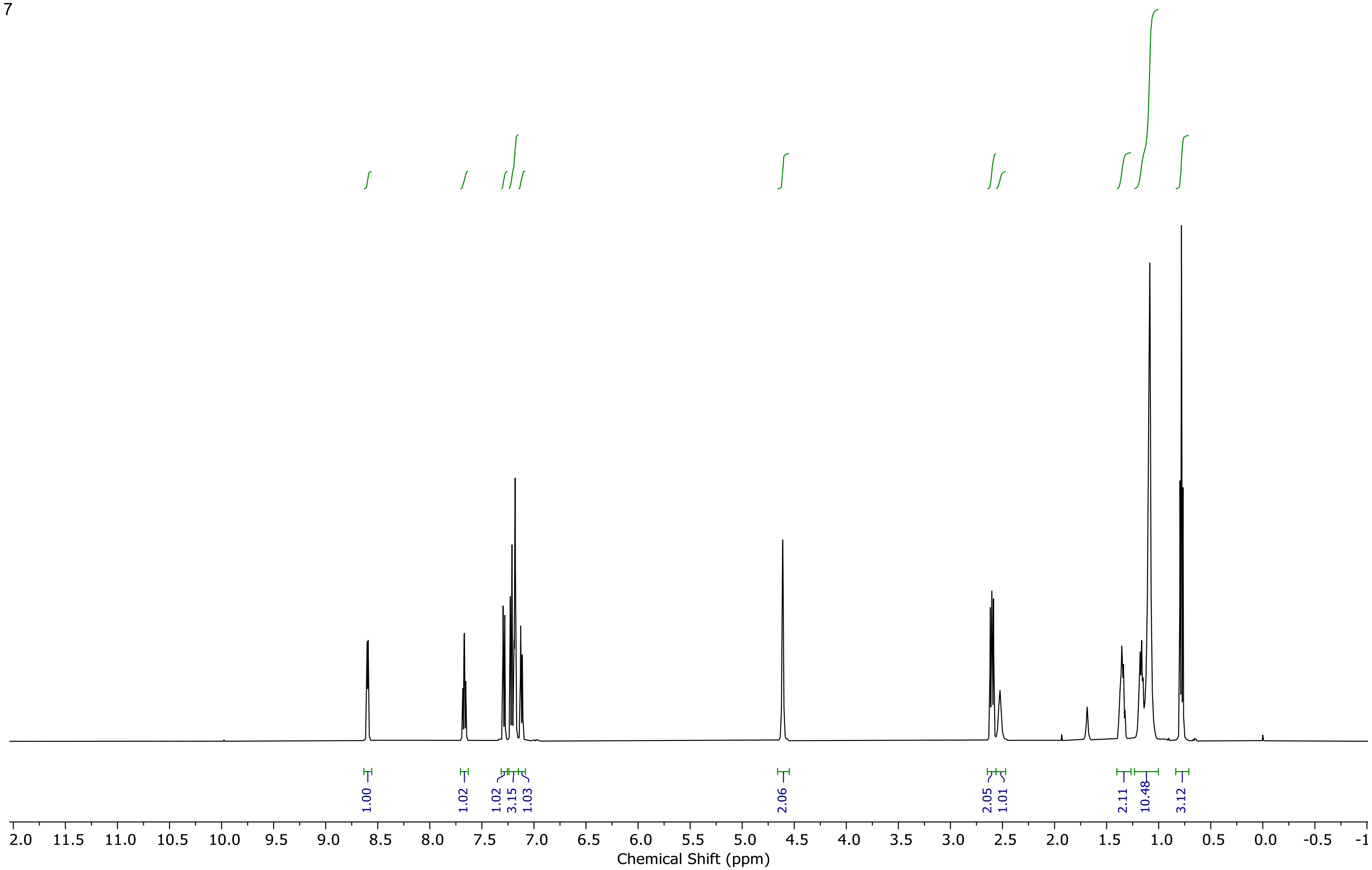

^{13}C NMR (126 MHz, CDCl₃) of (3-octyl-4-(pyridin-2-yl)phenyl)methanol--2-(2-methyl-6-octylphenyl)pyridine 14h

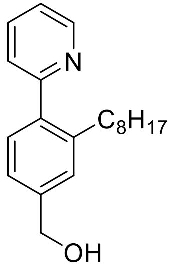

14h

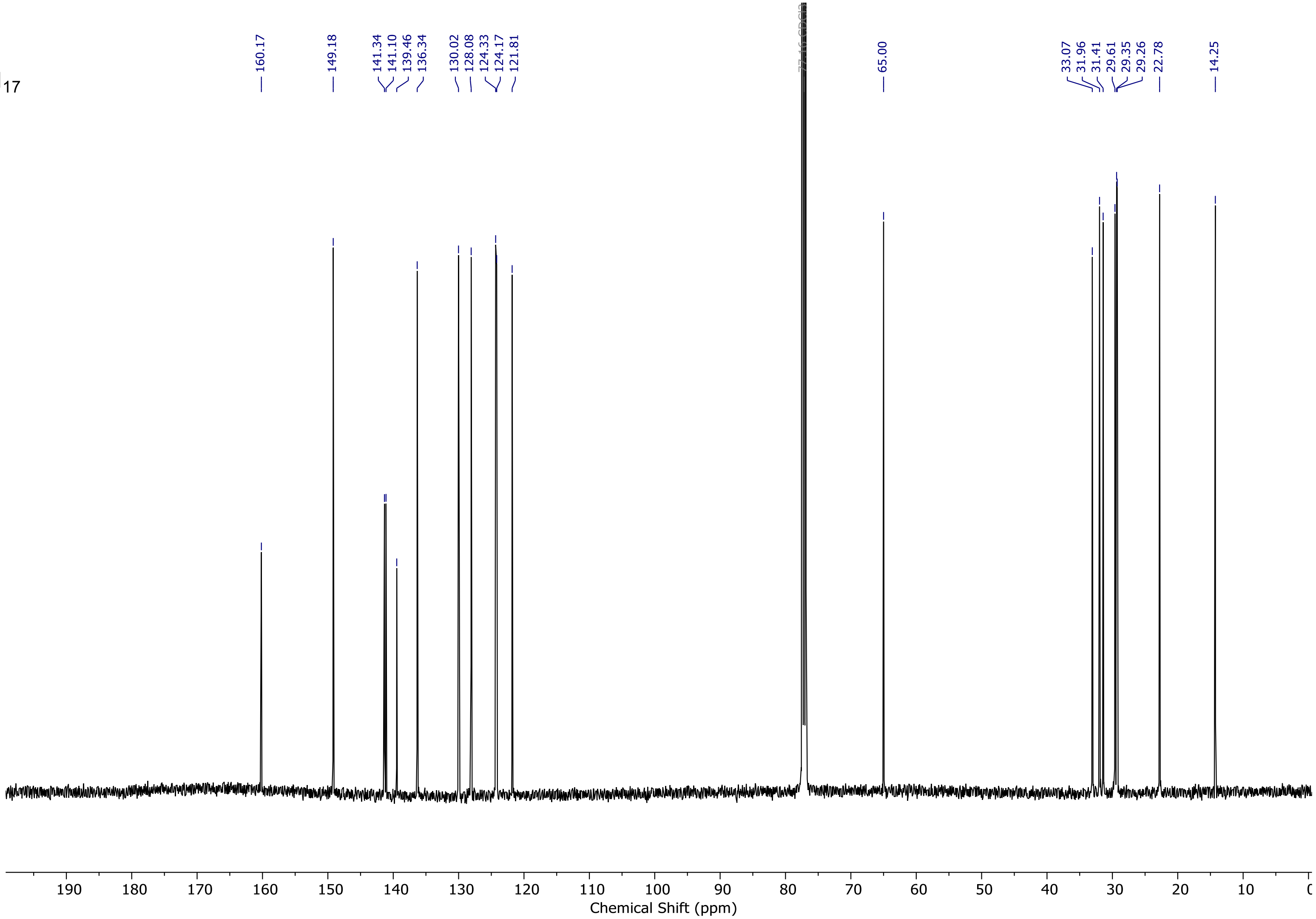

^{1}H NMR (500 MHz, $CDCl_3$) of 3-methyl-2-(2-octylphenyl)pyridine 14i

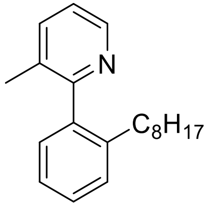

14i

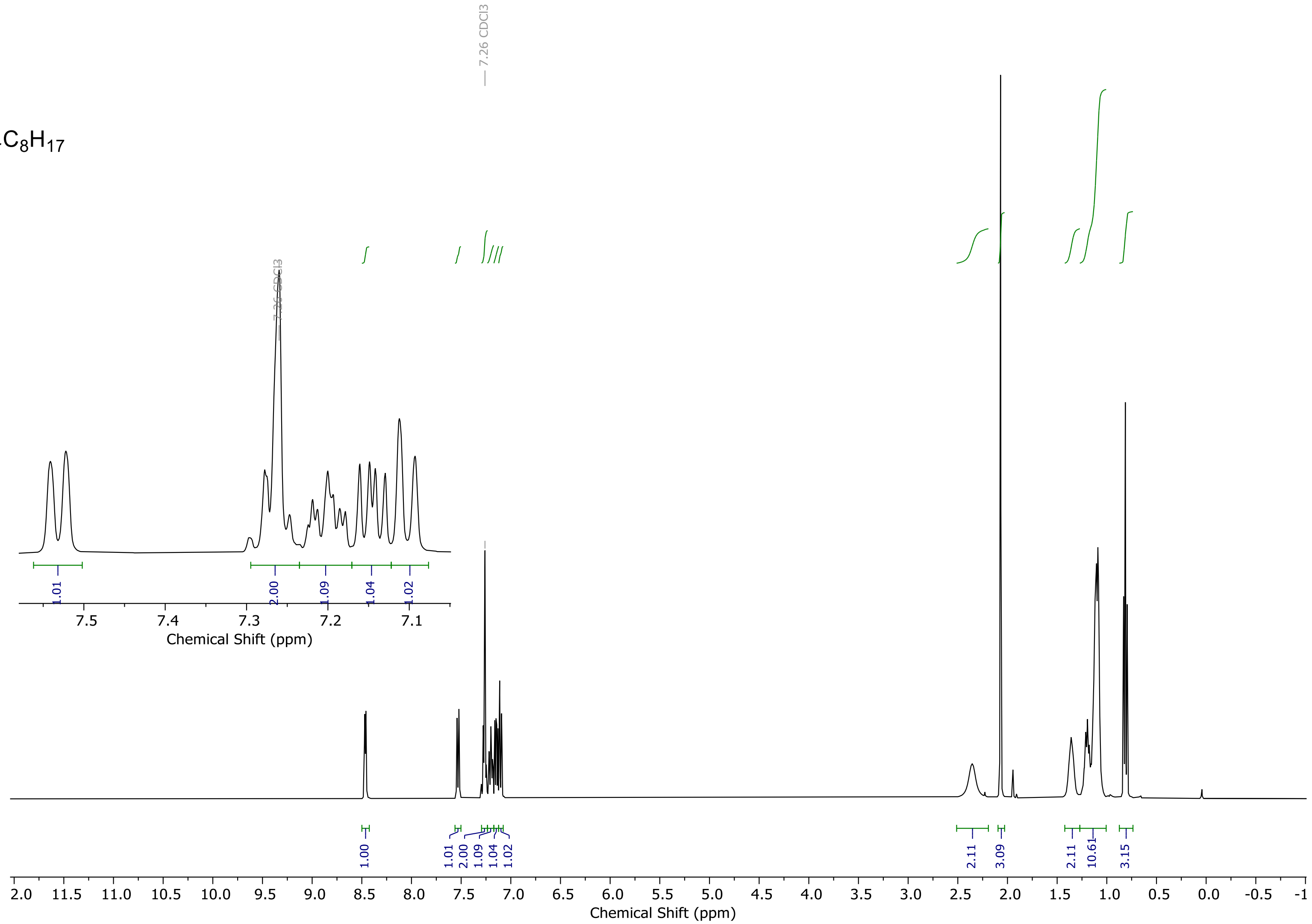

^{13}C NMR (101 MHz, CDCl₃) of 3-methyl-2-(2-octylphenyl)pyridine 14i

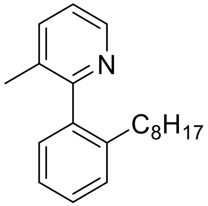

14i

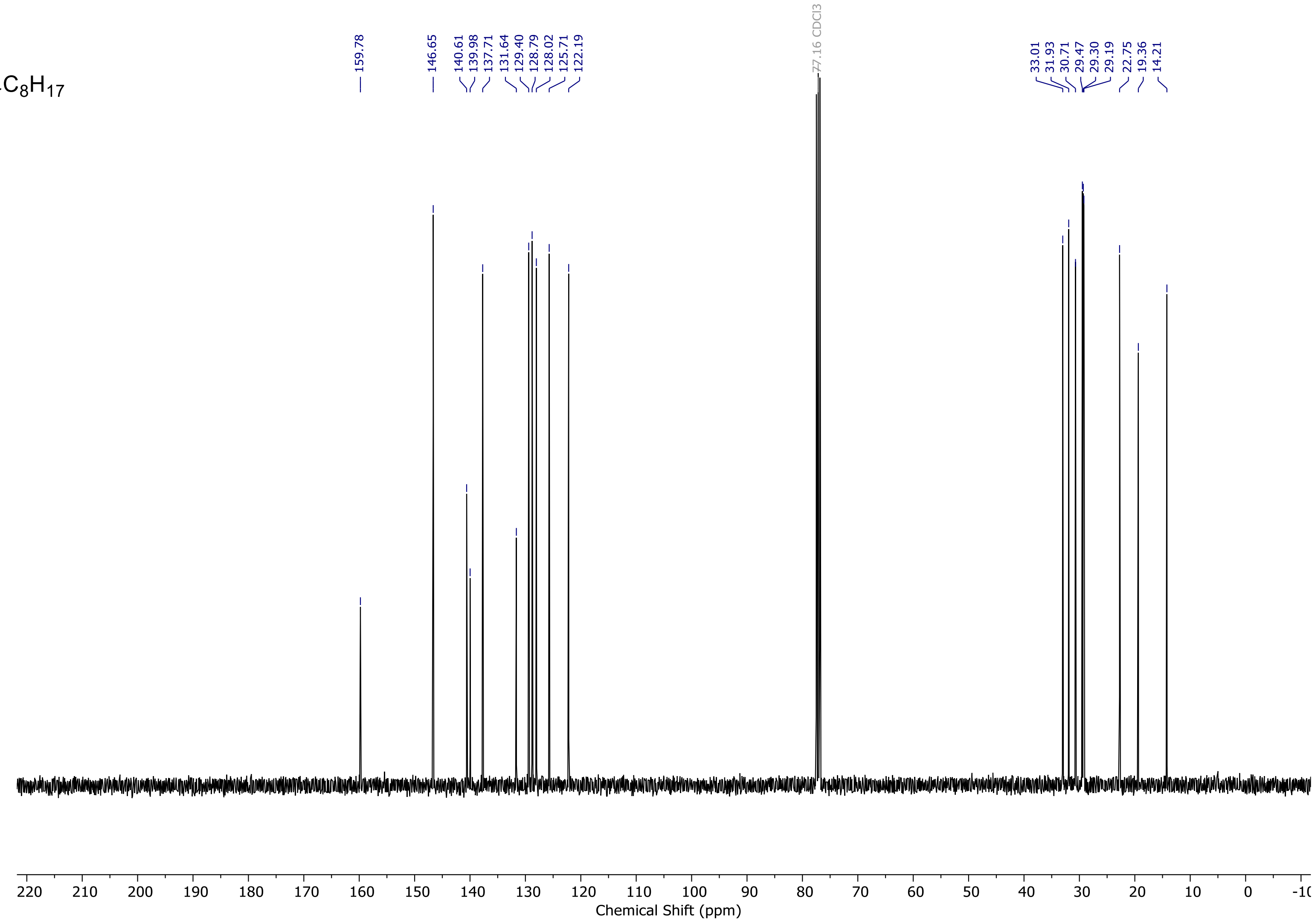

^{1}H NMR (500 MHz, $CDCl_3$) of 3-methyl-2-(2-(3-phenylpropyl)phenyl)pyridine 14j

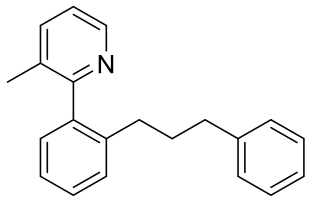

14j

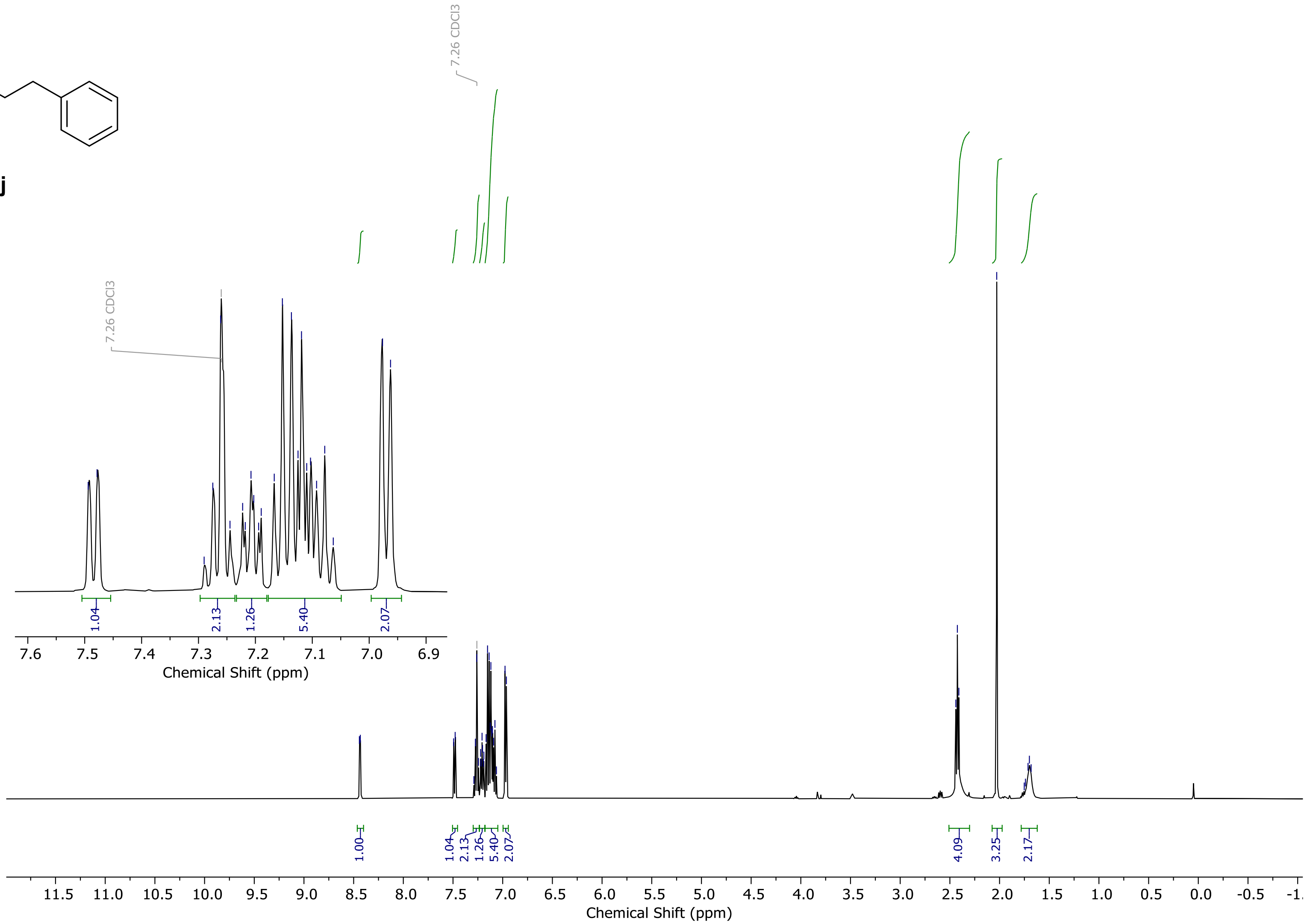

^{13}C NMR (126 MHz, $CDCl_3$) of 3-methyl-2-(2-(3-phenylpropyl)phenyl)pyridine 14j

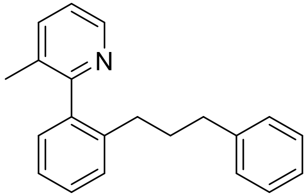

14j

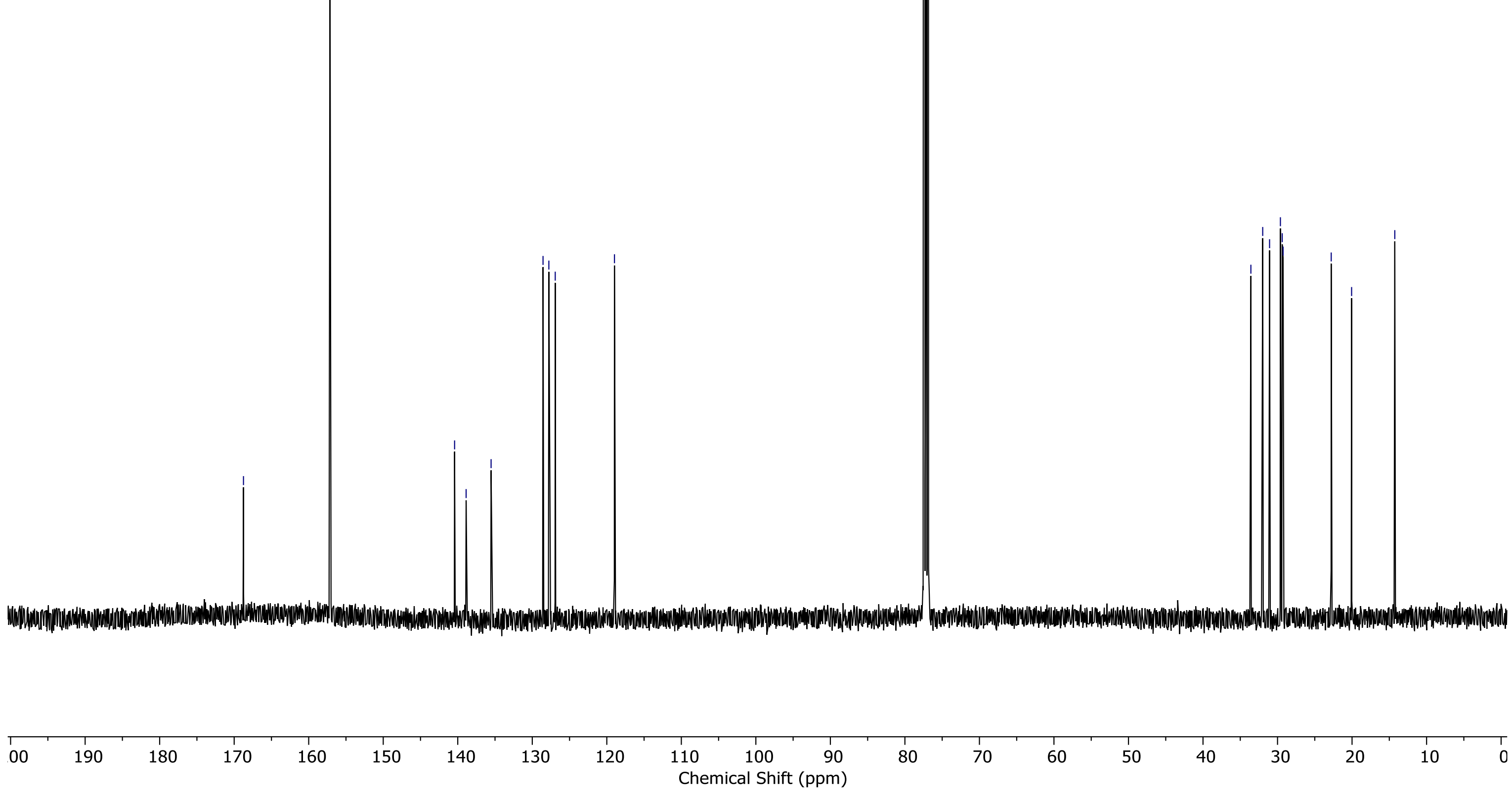

^{1}H NMR (400 MHz, $CDCl_3$) of *tert*-butyl 4-(2-(3-methylpyridin-2-yl)benzyl)piperidine-1-carboxylate 14k

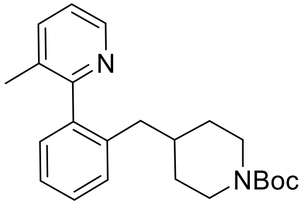

14k

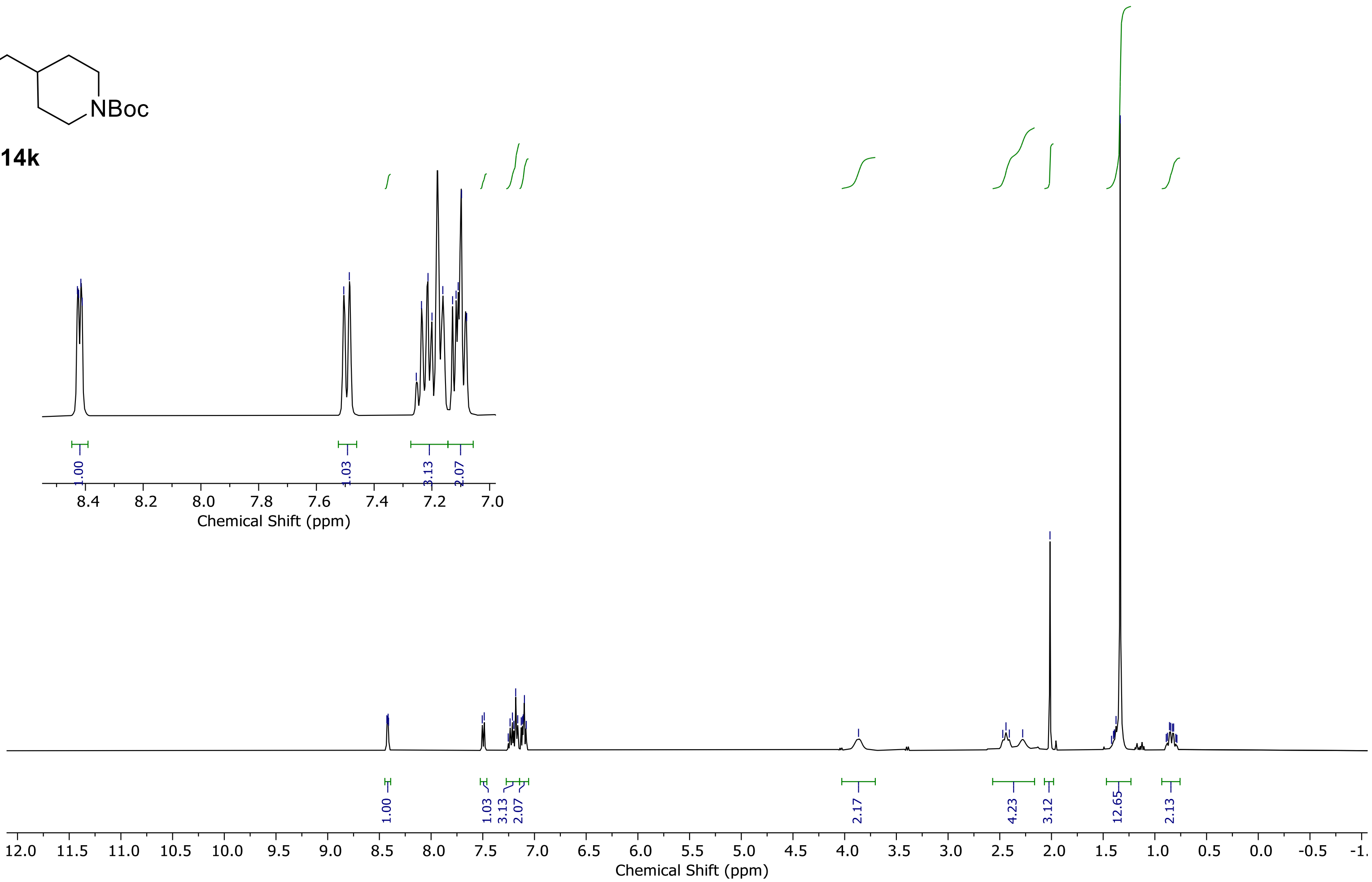

^{13}C NMR (101 MHz, $CDCl_3$) of *tert*-butyl 4-(2-(3-methylpyridin-2-yl)benzyl)piperidine-1-carboxylate 14k

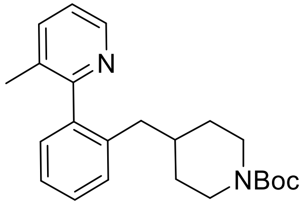

14k

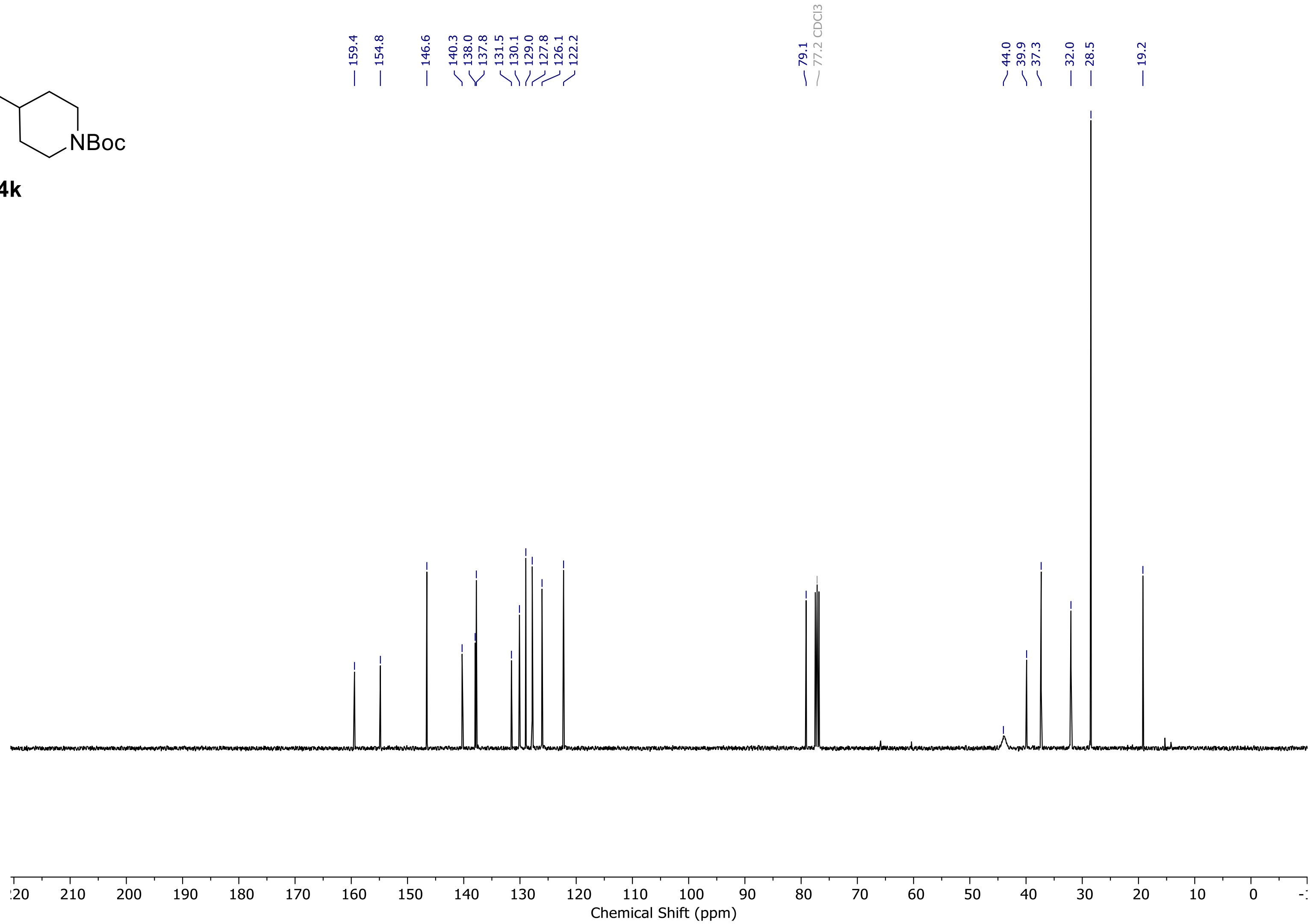

^{1}H NMR (400 MHz, CDCl$_3$) of [3-Me-2-Phenyl-pyridine]-[cholesterol derivative] 14l

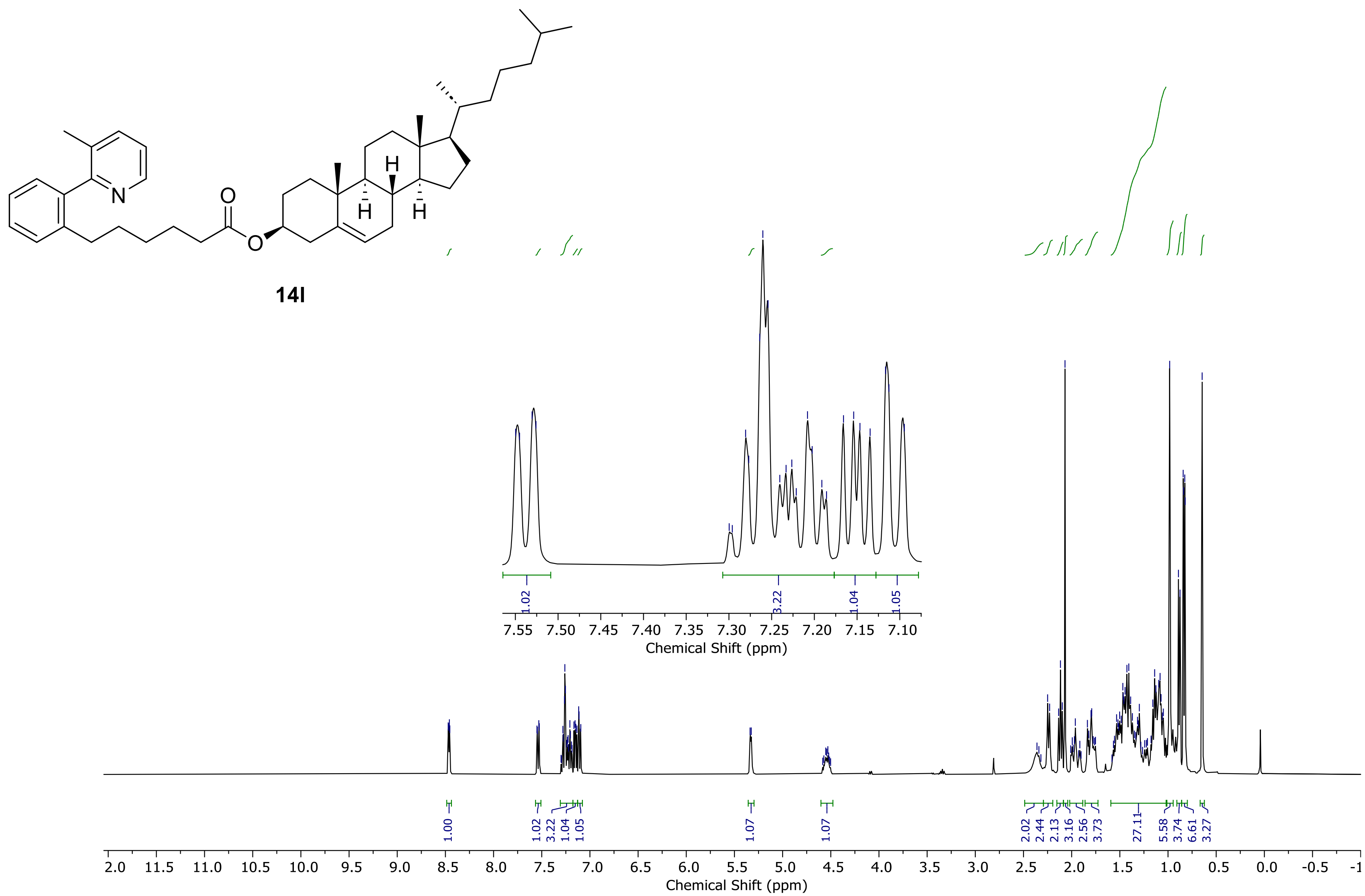

^{13}C NMR (101 MHz, CDCl$_3$) of [3-Me-2-Phenyl-pyridine]-[cholesterol derivative] 14l

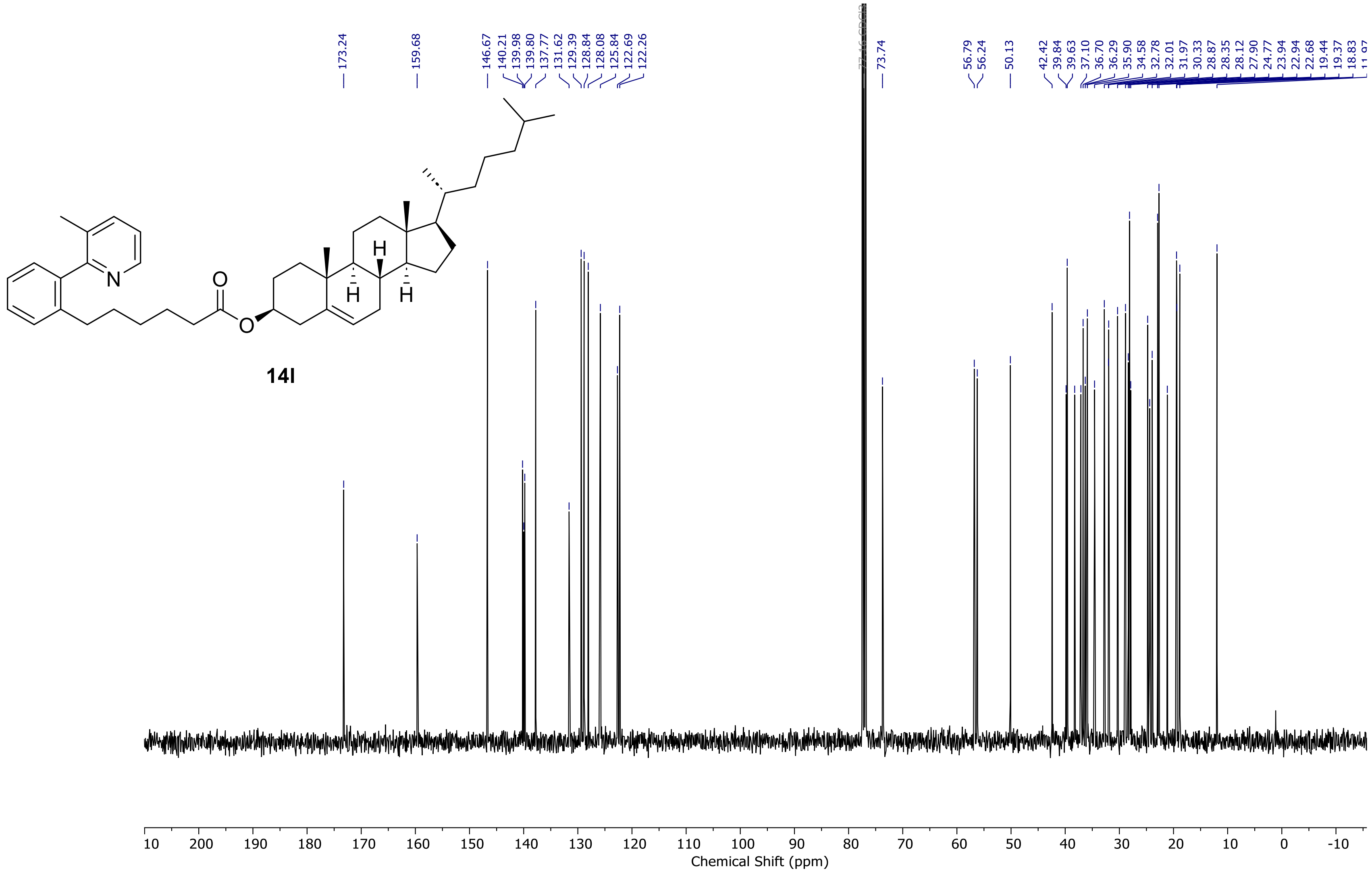

^{1}H NMR (400 MHz, CDCl$_3$) of Starting Material 8j

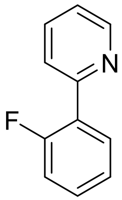

8j

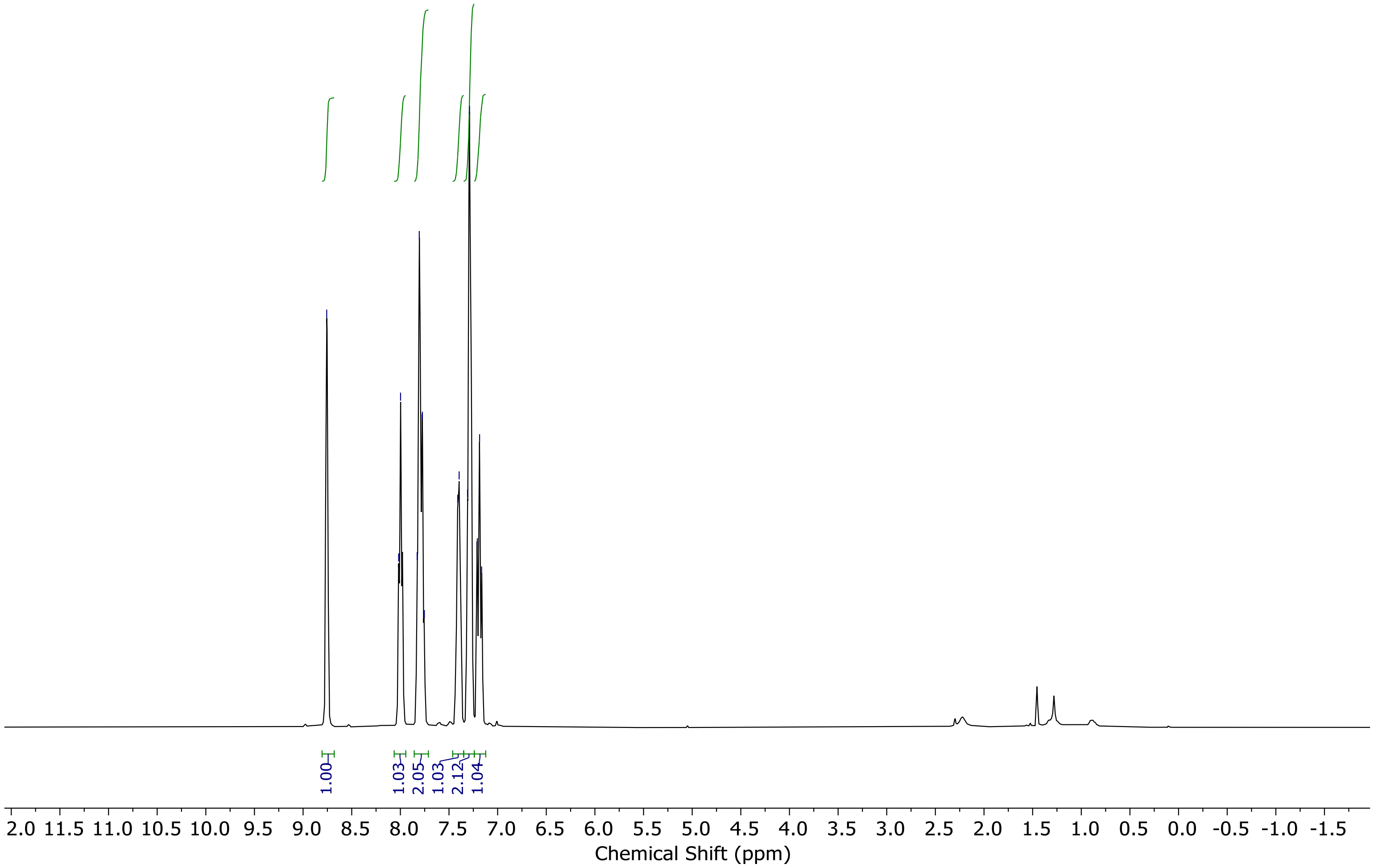

^{13}C NMR (101 MHz, CDCl$_3$) of Starting Material 8j

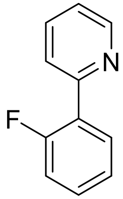

8j

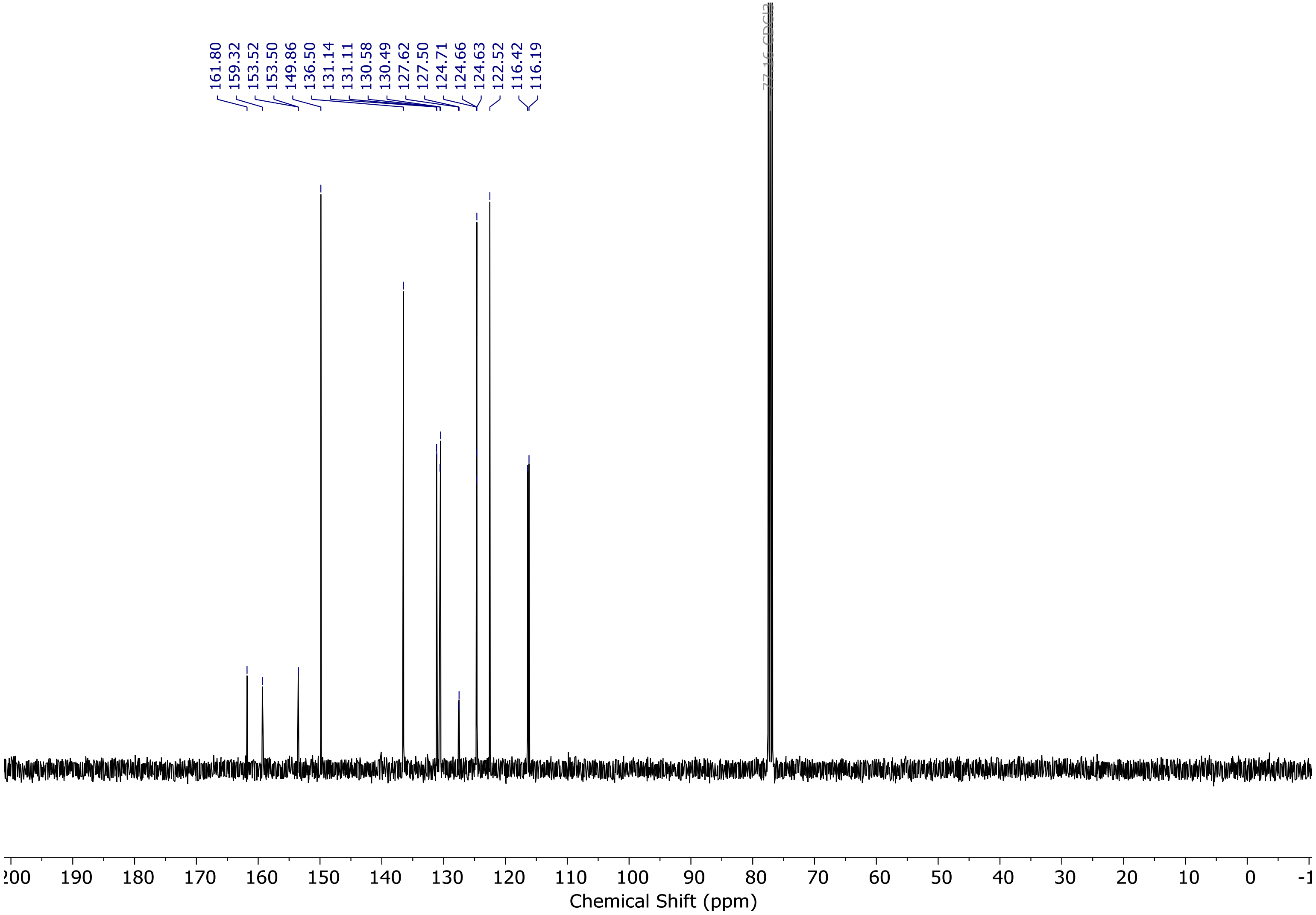

^{19}F NMR (376 MHz, CDCl$_3$) of Starting Material 8j

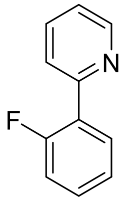

8j

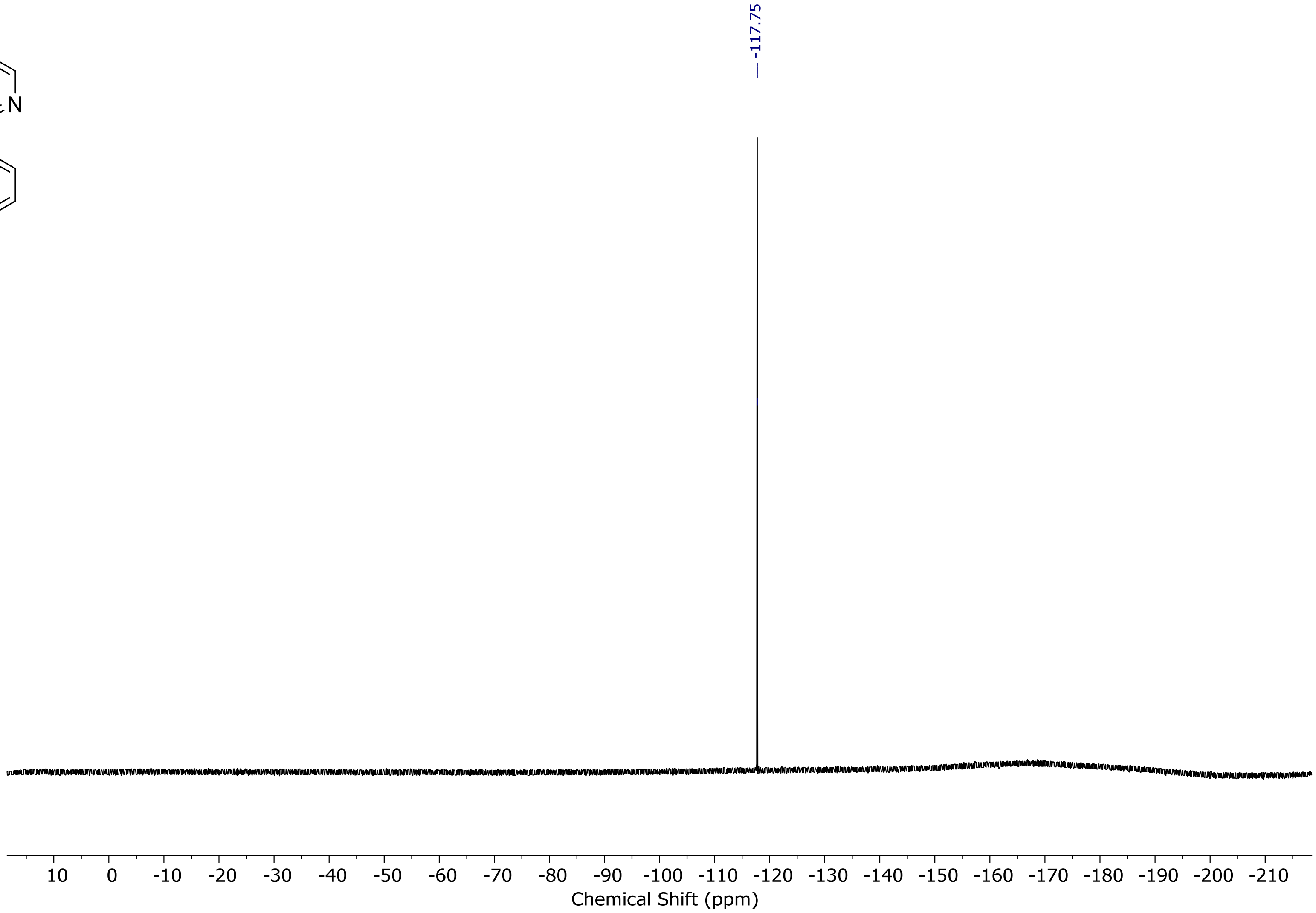

^{1}H NMR (400 MHz, $CDCl_3$) of Starting Material 13a

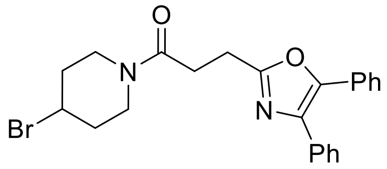

13a

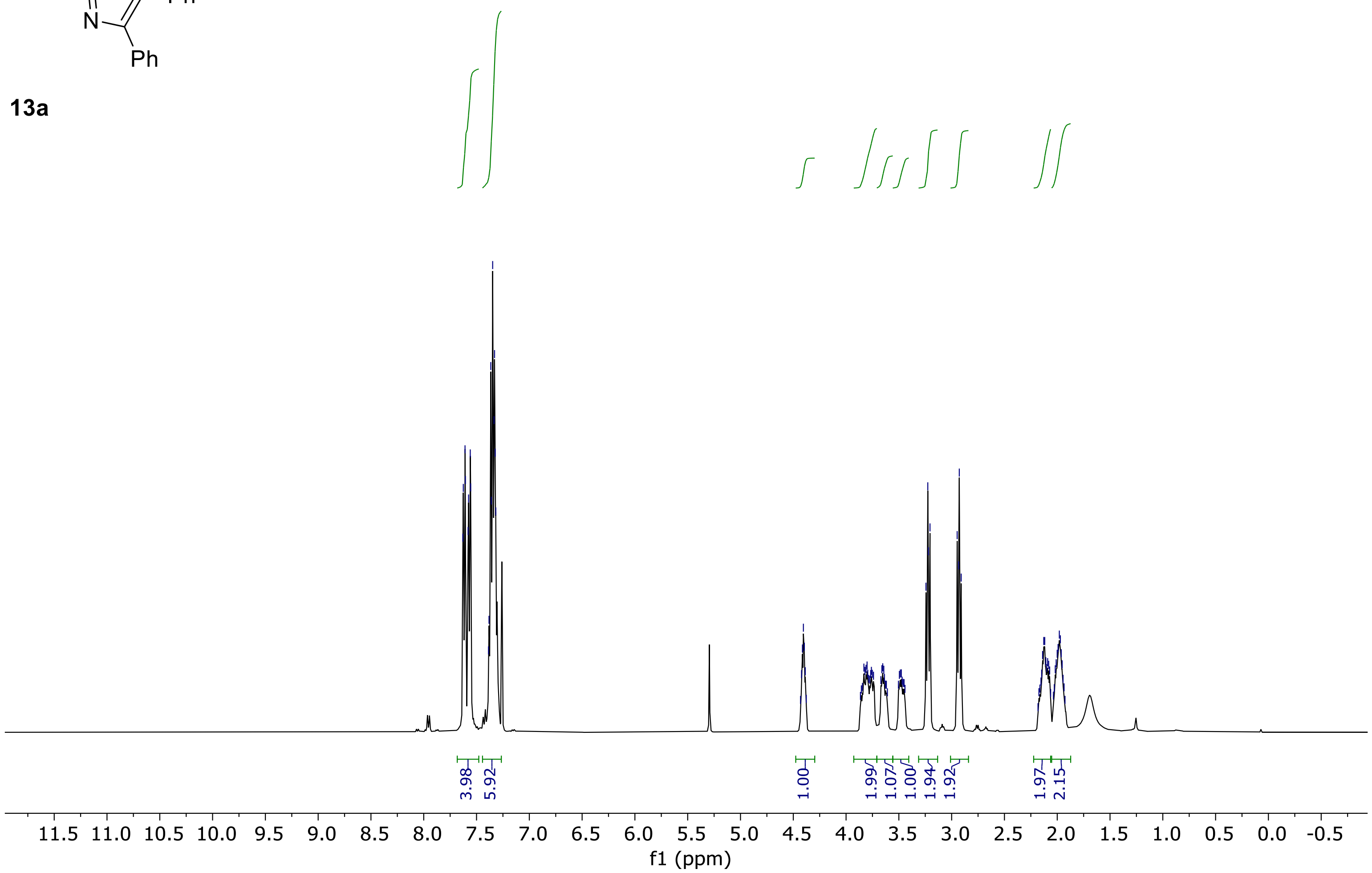

^{13}C NMR (101 MHz, CDCl$_3$) of Starting Material 13a

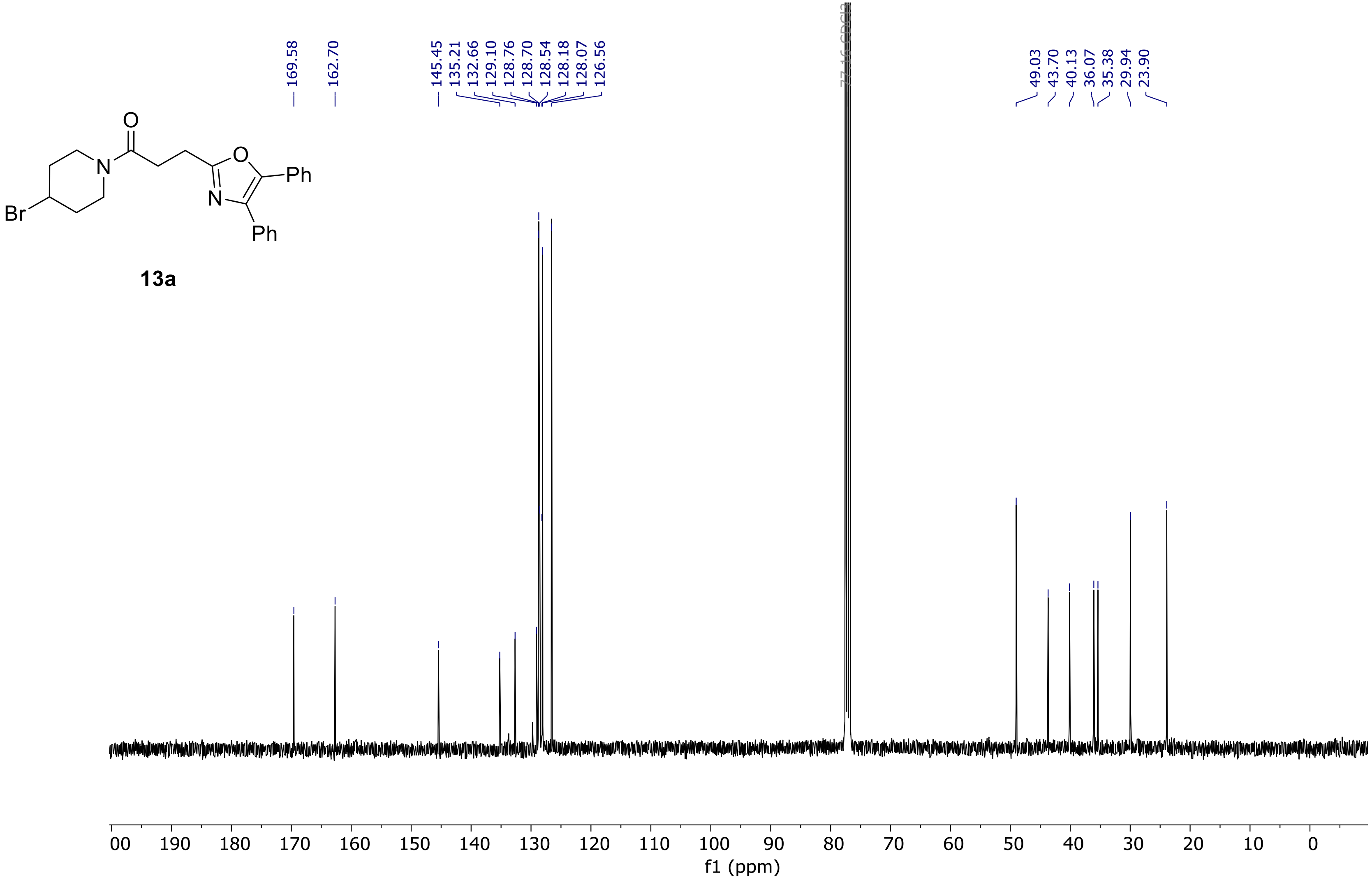

13a

^{1}H NMR (400 MHz, $CDCl_3$) of Starting Material 13c

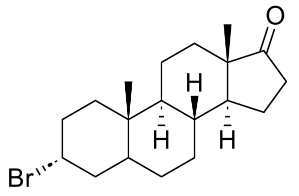

13c

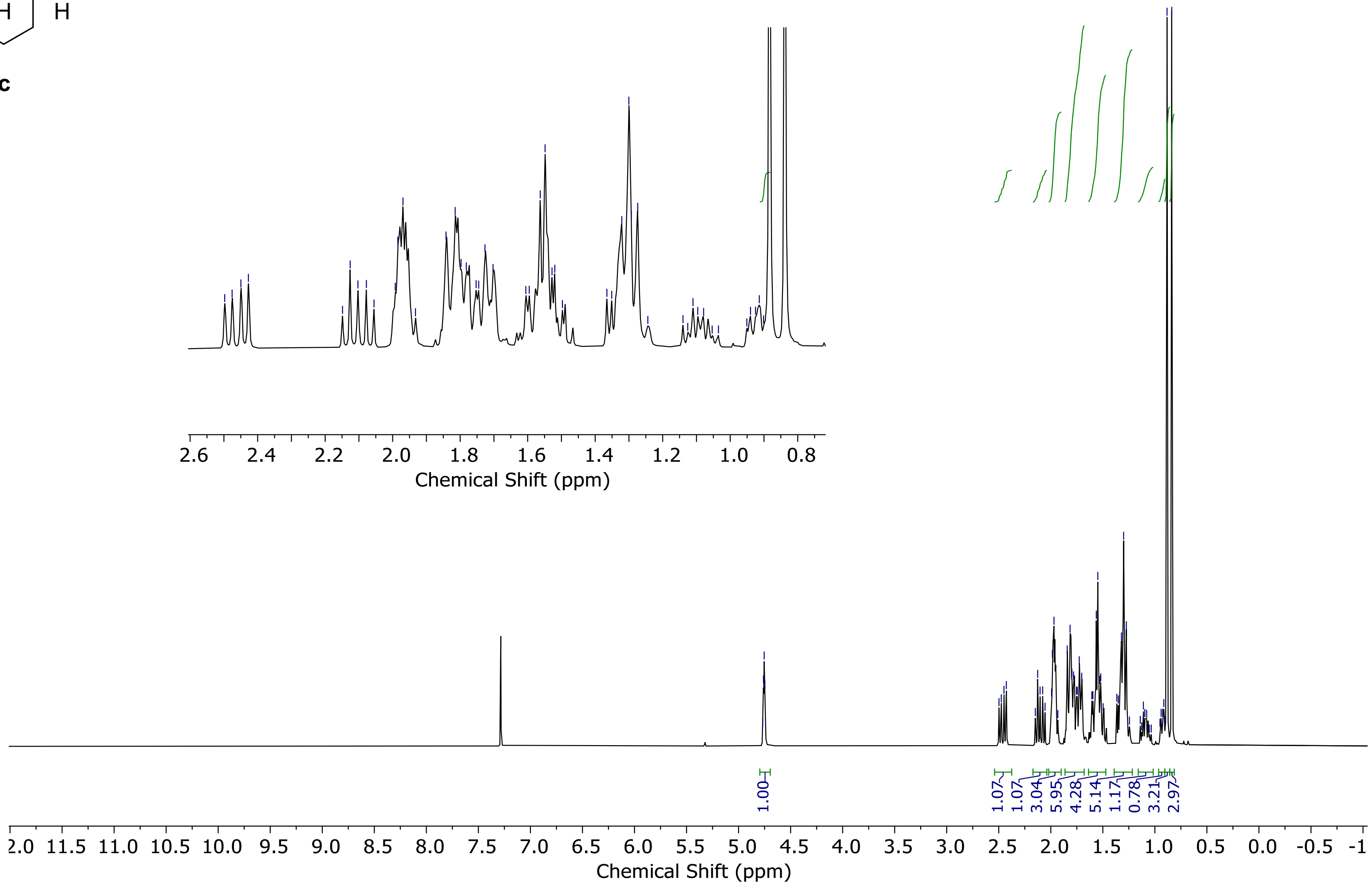

^{13}C NMR (101 MHz, CDCl$_3$) of Starting Material 13c

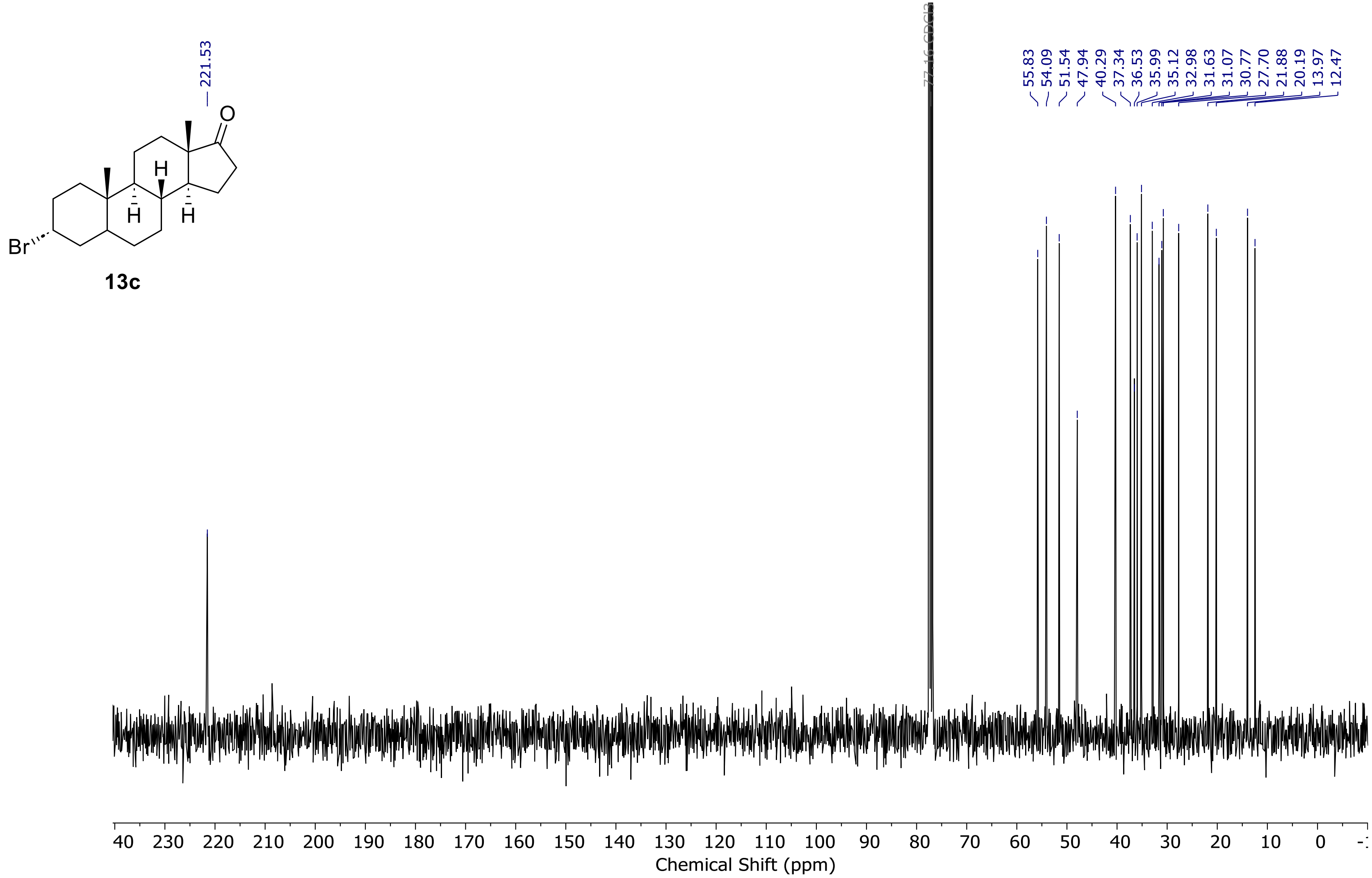

^{1}H NMR (400 MHz, $CDCl_3$) of Starting Material 13e

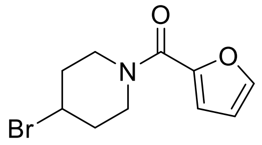

13e

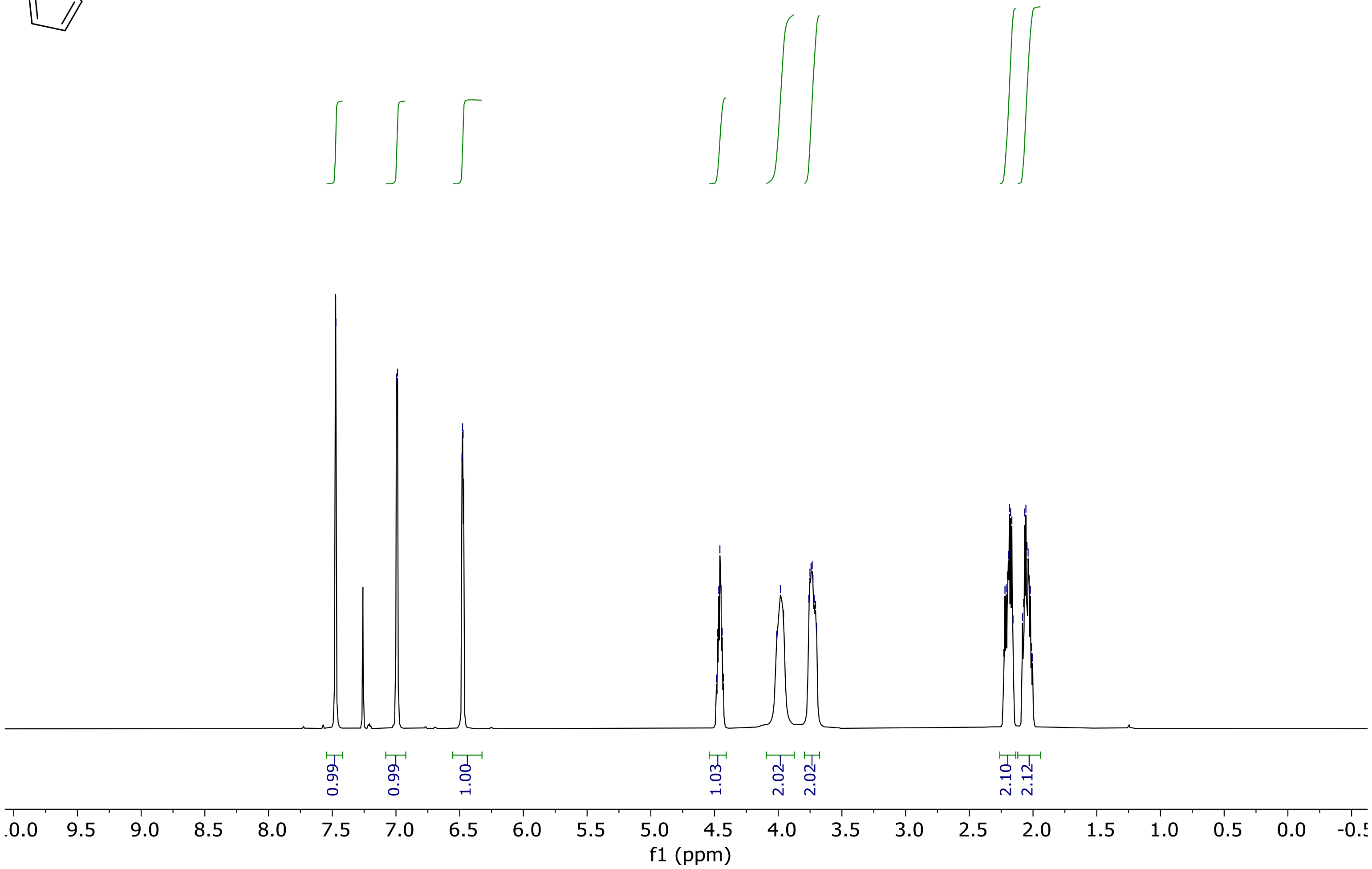

^{13}C NMR (101 MHz, CDCl$_3$) of Starting Material 13e

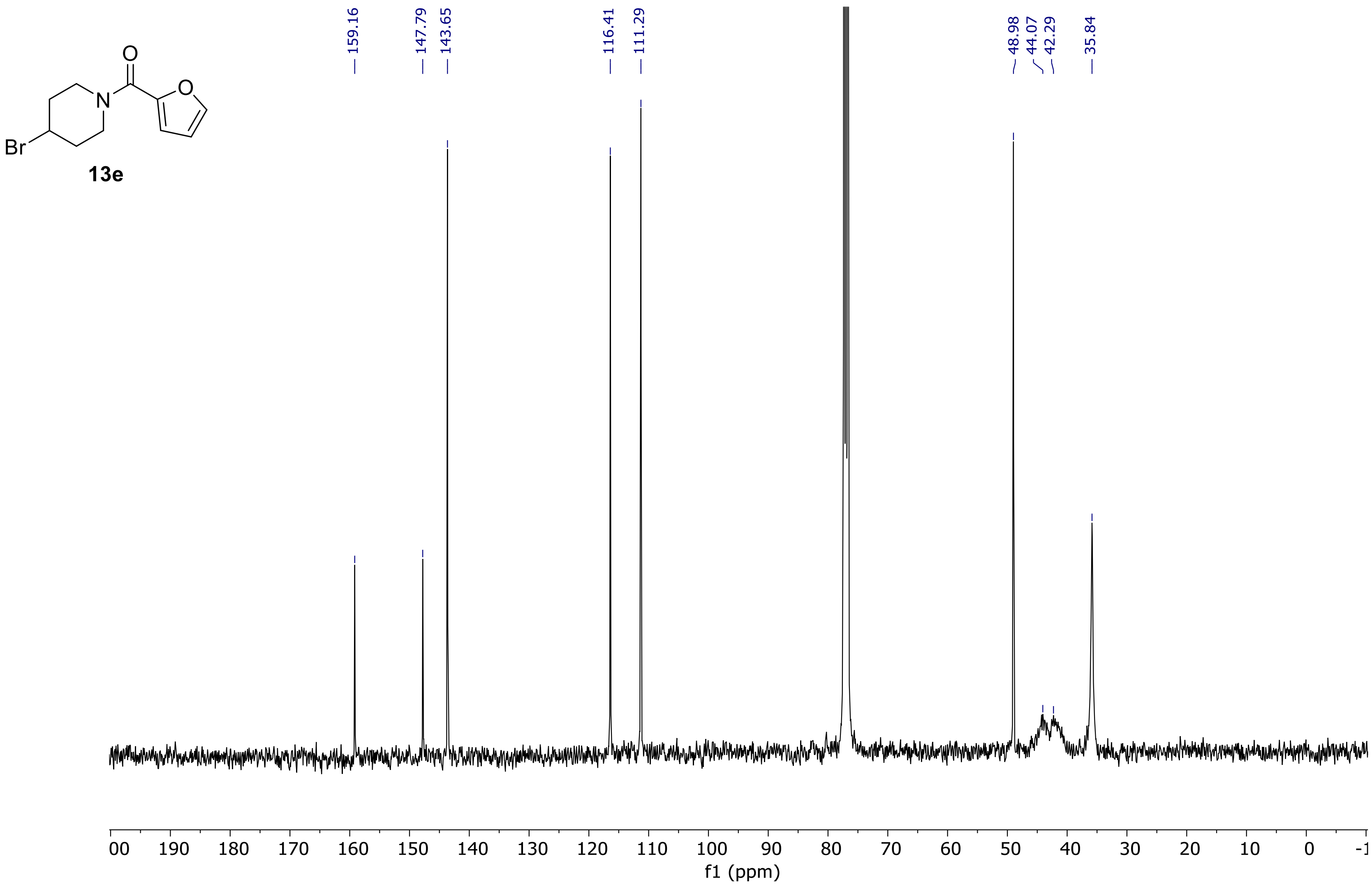

^{1}H NMR (400 MHz, $CDCl_3$) of 3-(4,5-diphenyloxazol-2-yl)-1-(4-(3-methyl-2-(pyridin-2-yl)phenyl)piperidin-1-yl)propan-1-one 15a

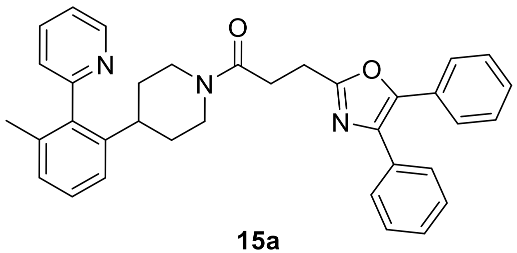

15a

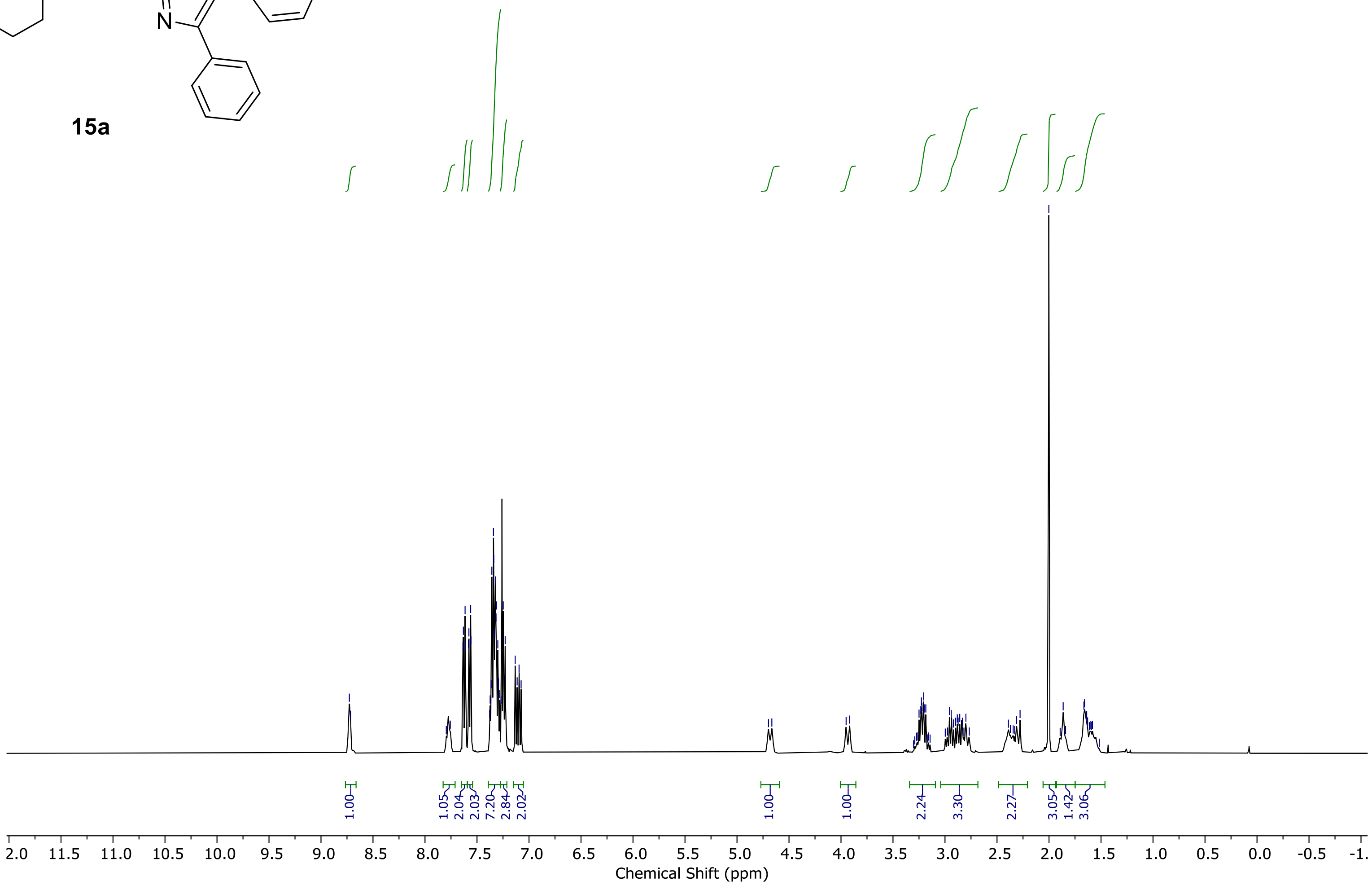

^{13}C NMR (101 MHz, CDCl₃) of 3-(4,5-diphenyloxazol-2-yl)-1-(4-(3-methyl-2-(pyridin-2-yl)phenyl)piperidin-1-yl)propan-1-one 15a

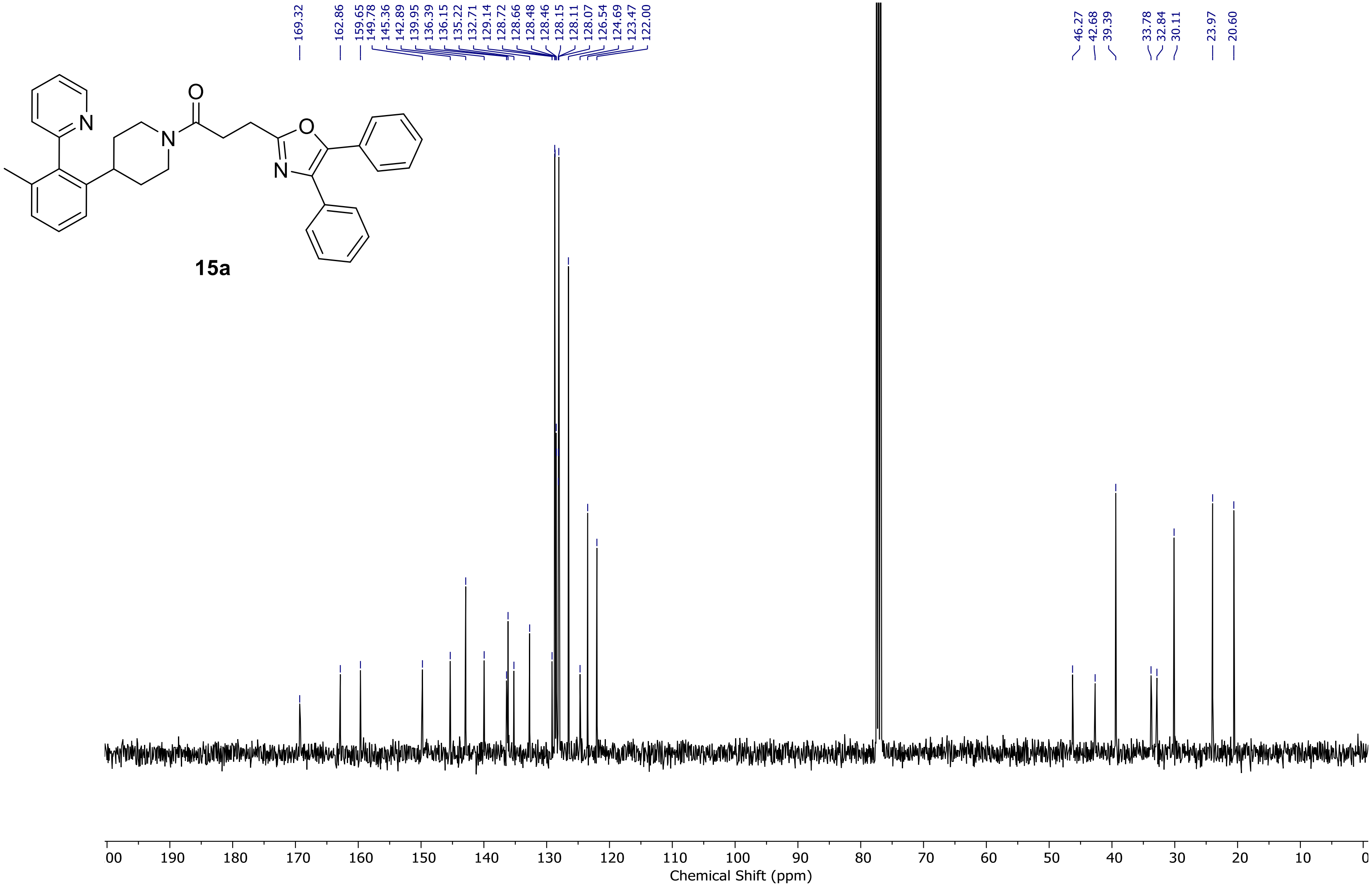

15a

^{1}H NMR (400 MHz, $CDCl_3$) of 2-(2-methyl-6-(tetrahydro-2H-pyran-4-yl)phenyl)pyrimidine 15b

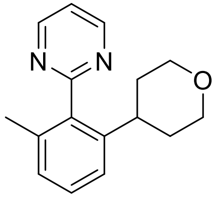

15b

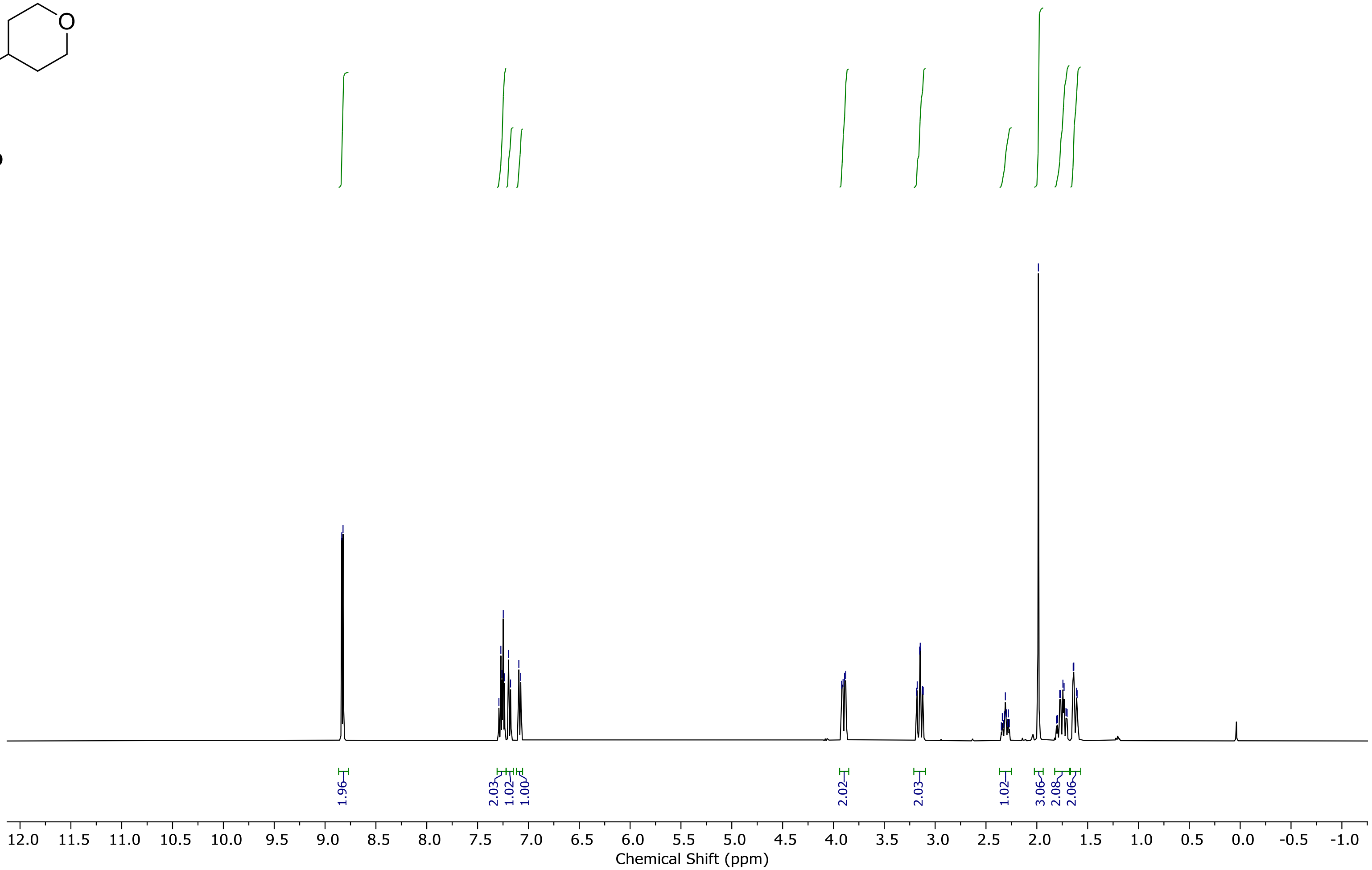

^{13}C NMR (101 MHz, $CDCl_3$) of 2-(2-methyl-6-(tetrahydro-2H-pyran-4-yl)phenyl)pyrimidine 15b

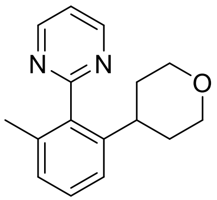

15b

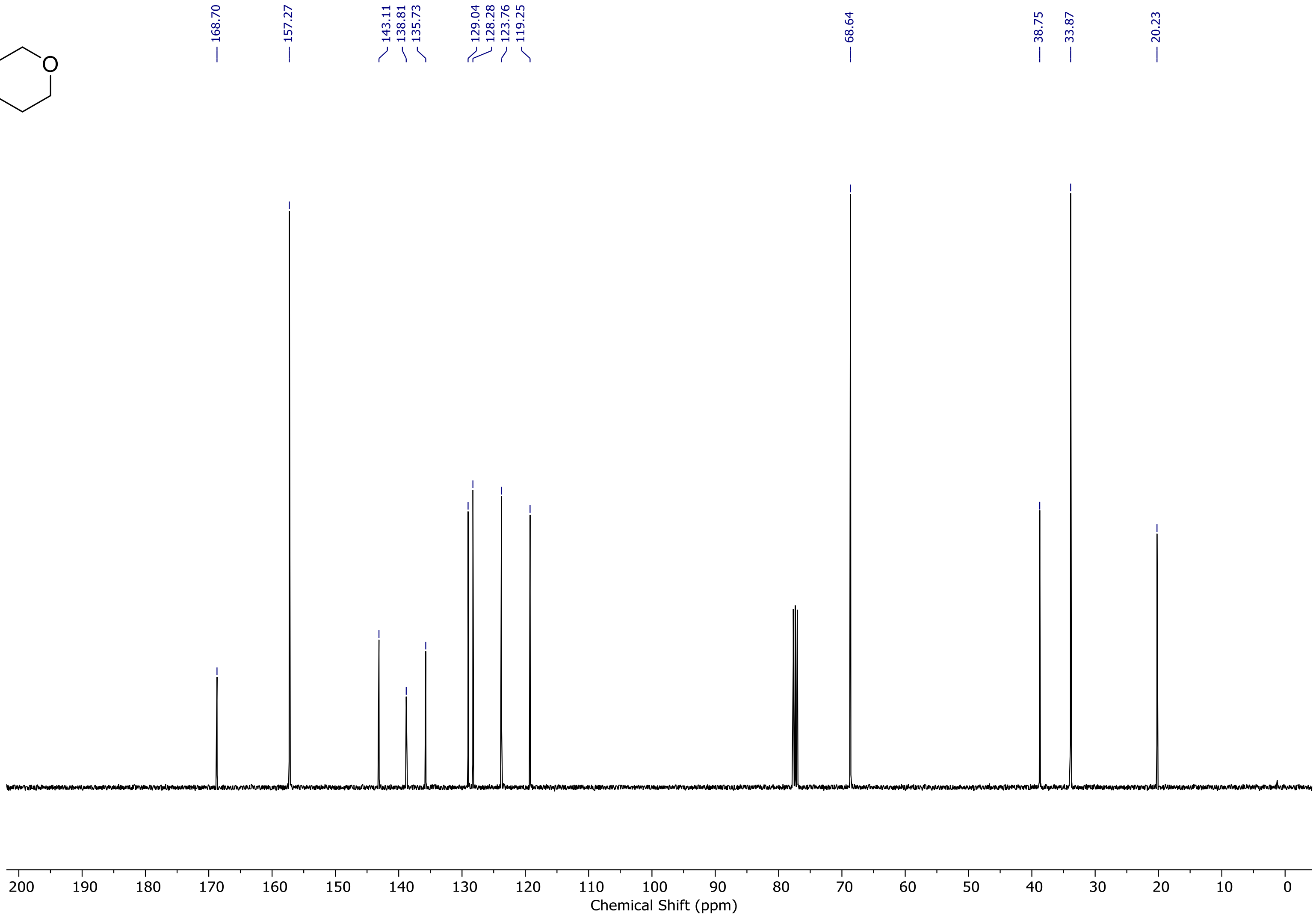

^{1}H NMR (500 MHz, $CDCl_3$) of 7-chloro-1-methyl-5-(2-(tetrahydro-2H-pyran-4-yl)phenyl)-1,3-dihydro-2H-benzo[e][1,4]diazepin-2-one 15c

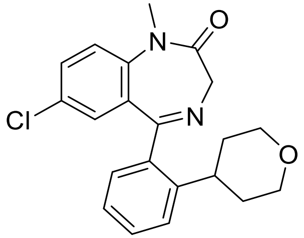

15c

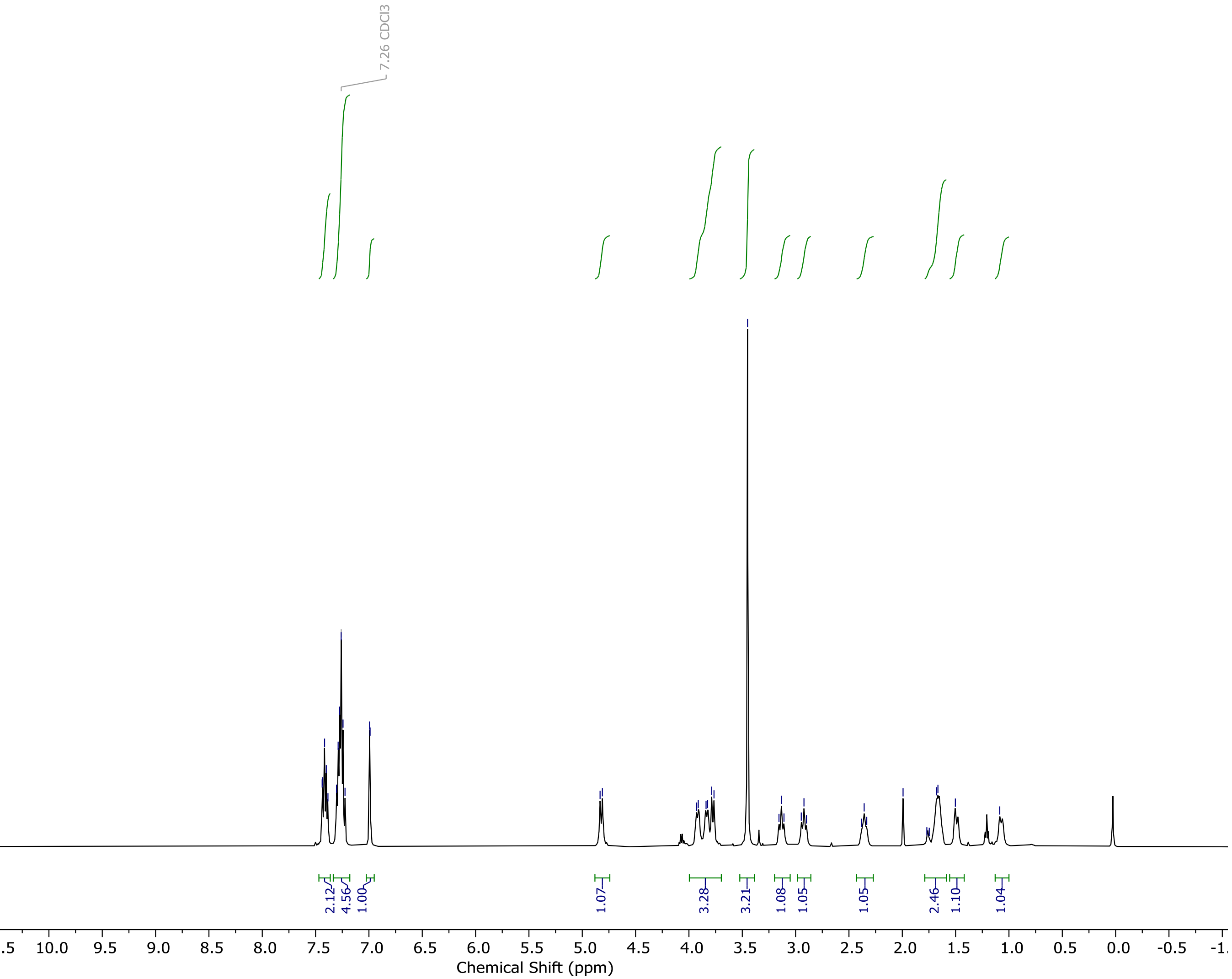

^{13}C NMR (126 MHz, $CDCl_3$) of 7-chloro-1-methyl-5-(2-(tetrahydro-2H-pyran-4-yl)phenyl)-1,3-dihydro-2H-benzo[e][1,4]diazepin-2-one 15c

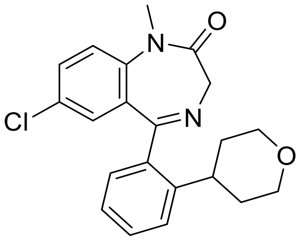

15c

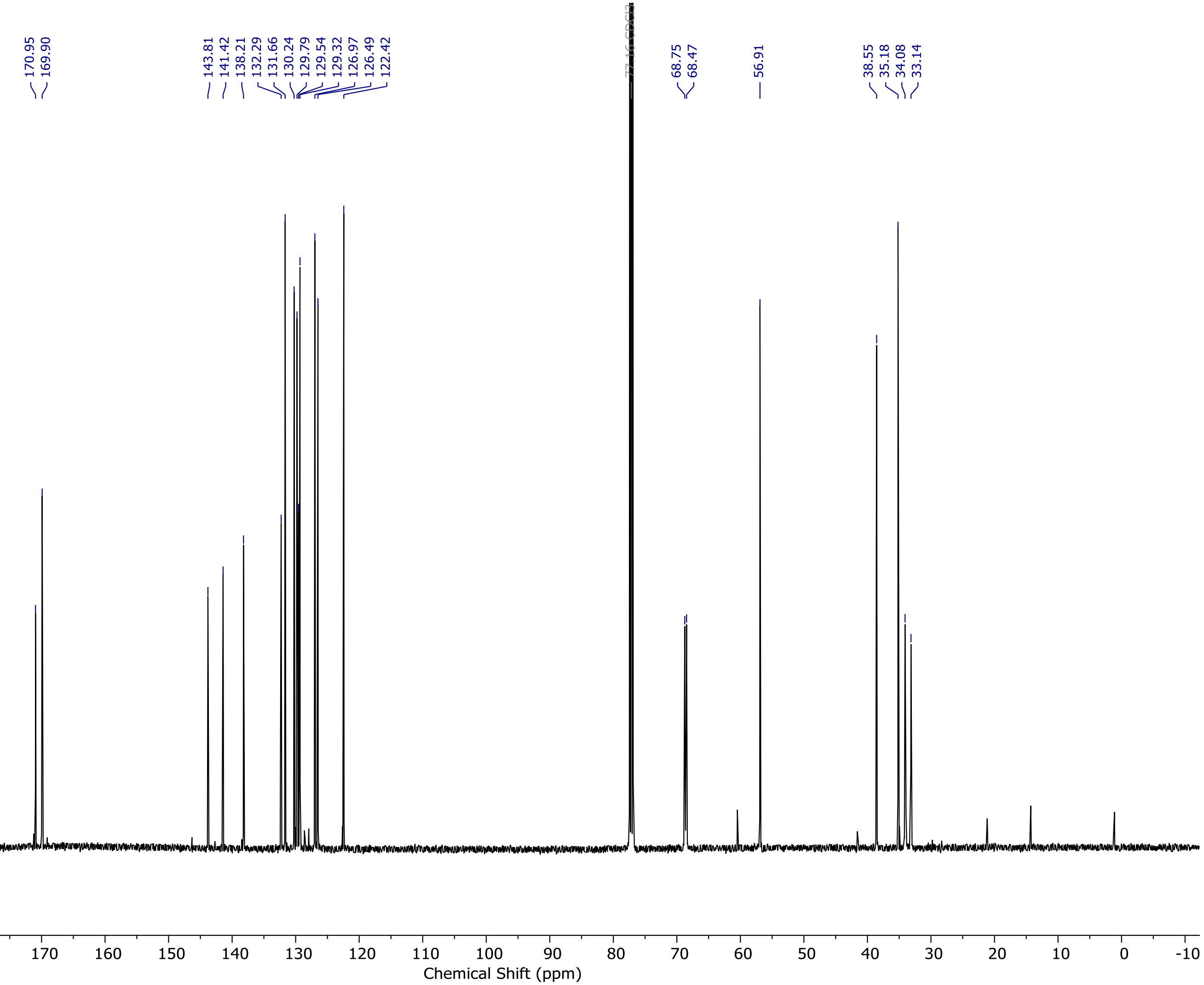

¹H NMR (500 MHz, CDCl$_3$) of (8R,9S,10S,13S,14S)-3-(3-fluoro-2-(pyridin-2-yl)phenyl)-10,13-dimethylhexadecahydro-17H-cyclopenta[a]phenanthren-17-one 15d

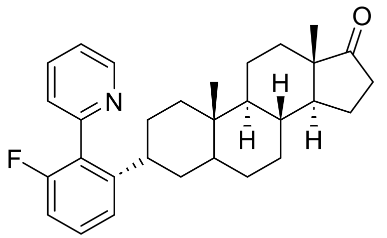

15d

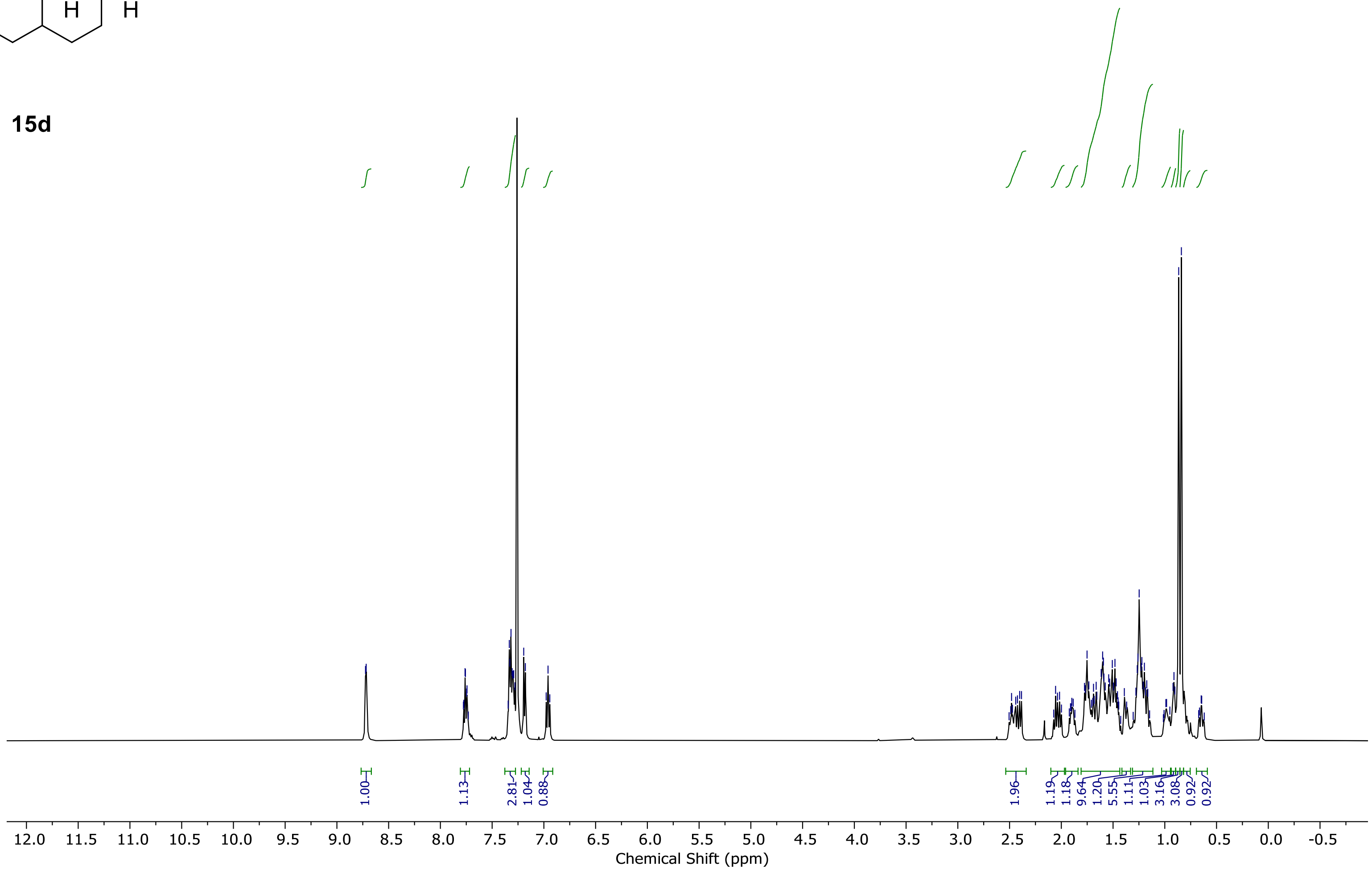

^{13}C NMR (126 MHz, CDCl$_3$) of (8*R*,9*S*,10*S*,13*S*,14*S*)-3-(3-fluoro-2-(pyridin-2-yl)phenyl)-10,13-dimethylhexadecahydro-17H-cyclopenta[a]phenanthren-17-one 15d

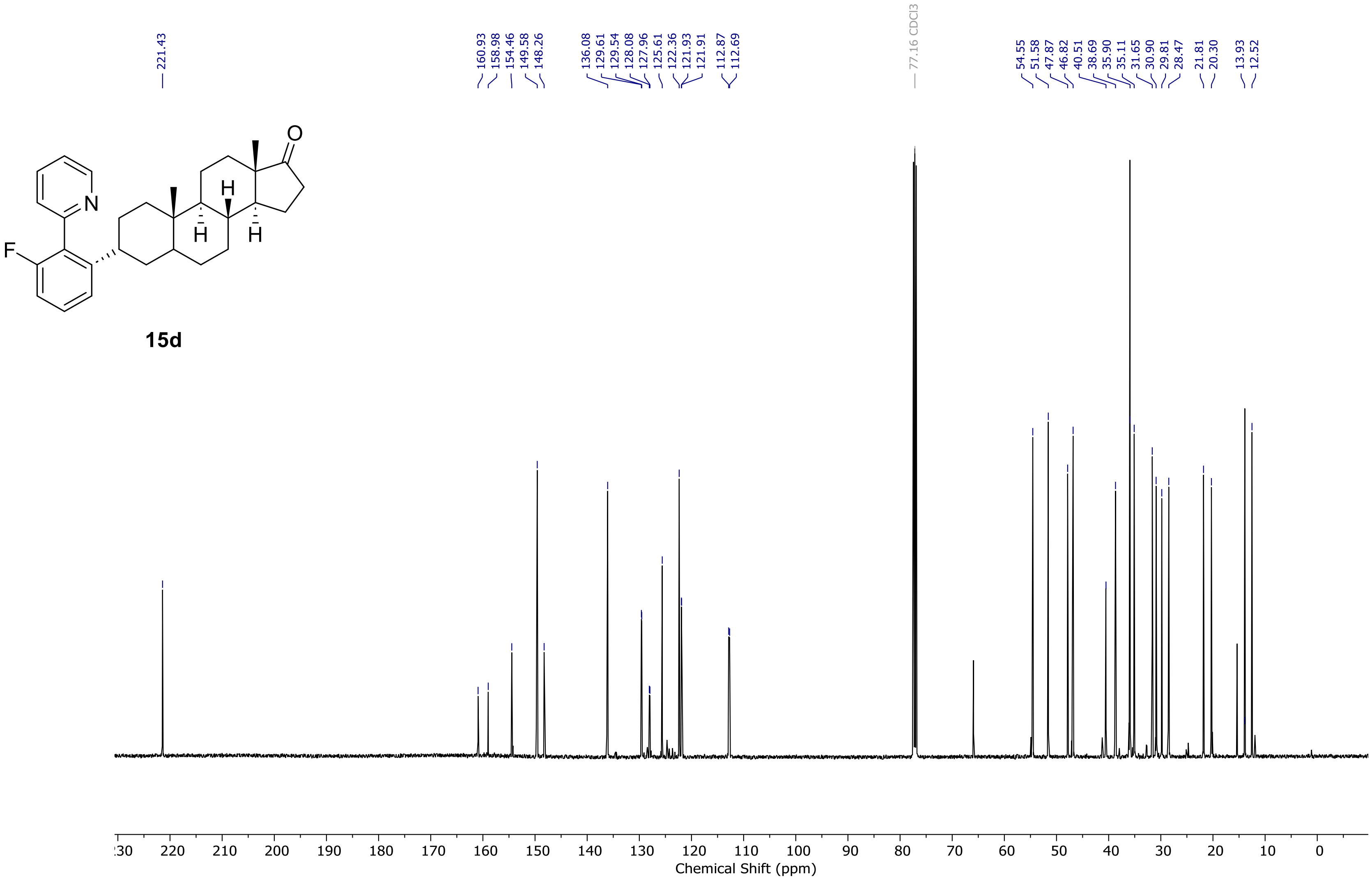

^{19}F NMR (376 MHz, $CDCl_3$) of (8*R*,9*S*,10*S*,13*S*,14*S*)-3-(3-fluoro-2-(pyridin-2-yl)phenyl)-10,13-dimethylhexadecahydro-17H-cyclopenta[a]phenanthren-17-one 15d

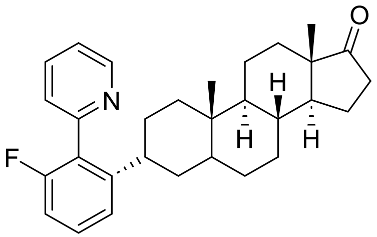

15d

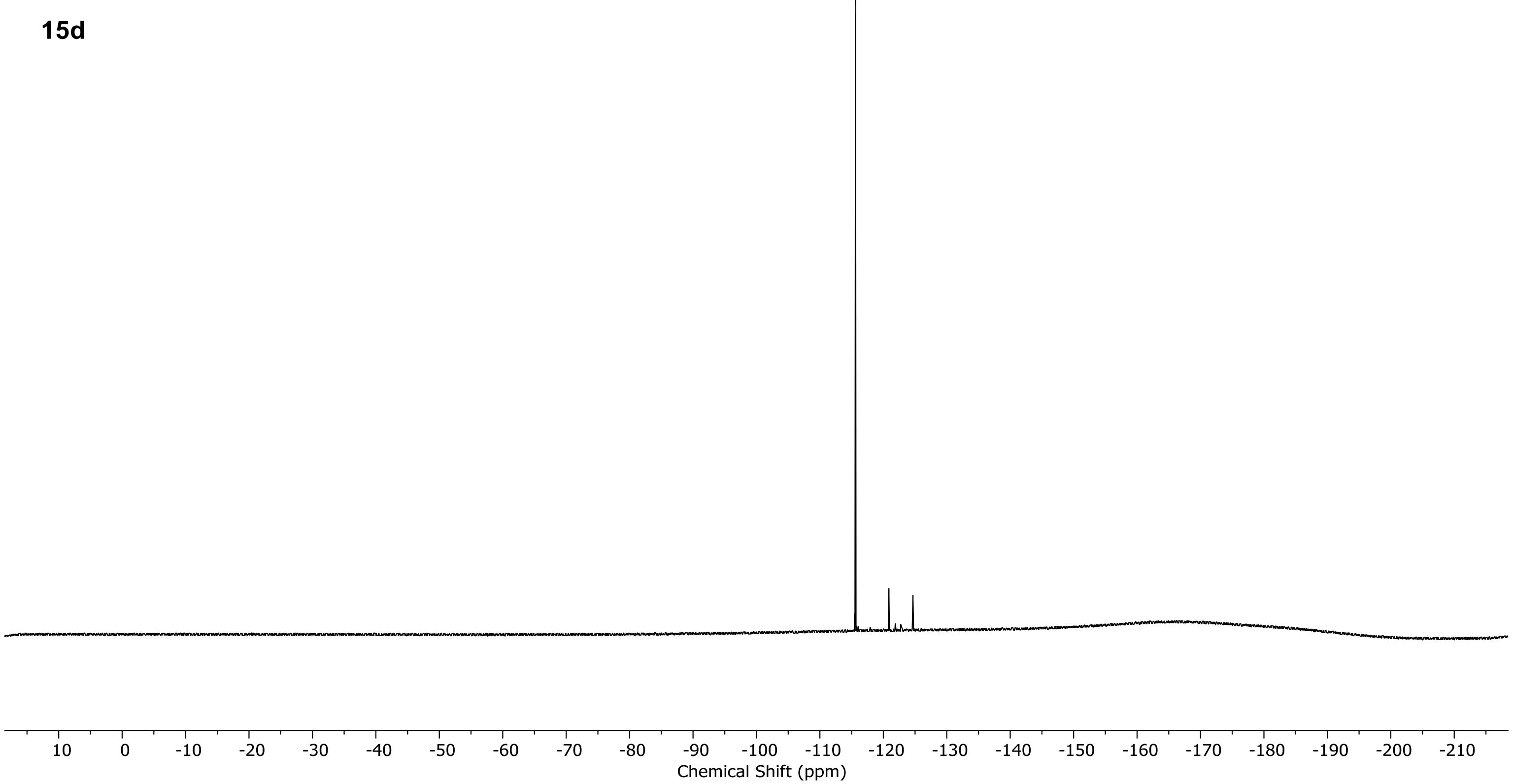

^{1}H NMR (400 MHz, $CDCl_3$) of 2-(2-methyl-6-(tetrahydro-2H-pyran-4-yl)phenyl)pyridine 15e

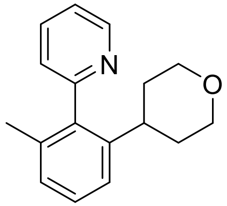

15e

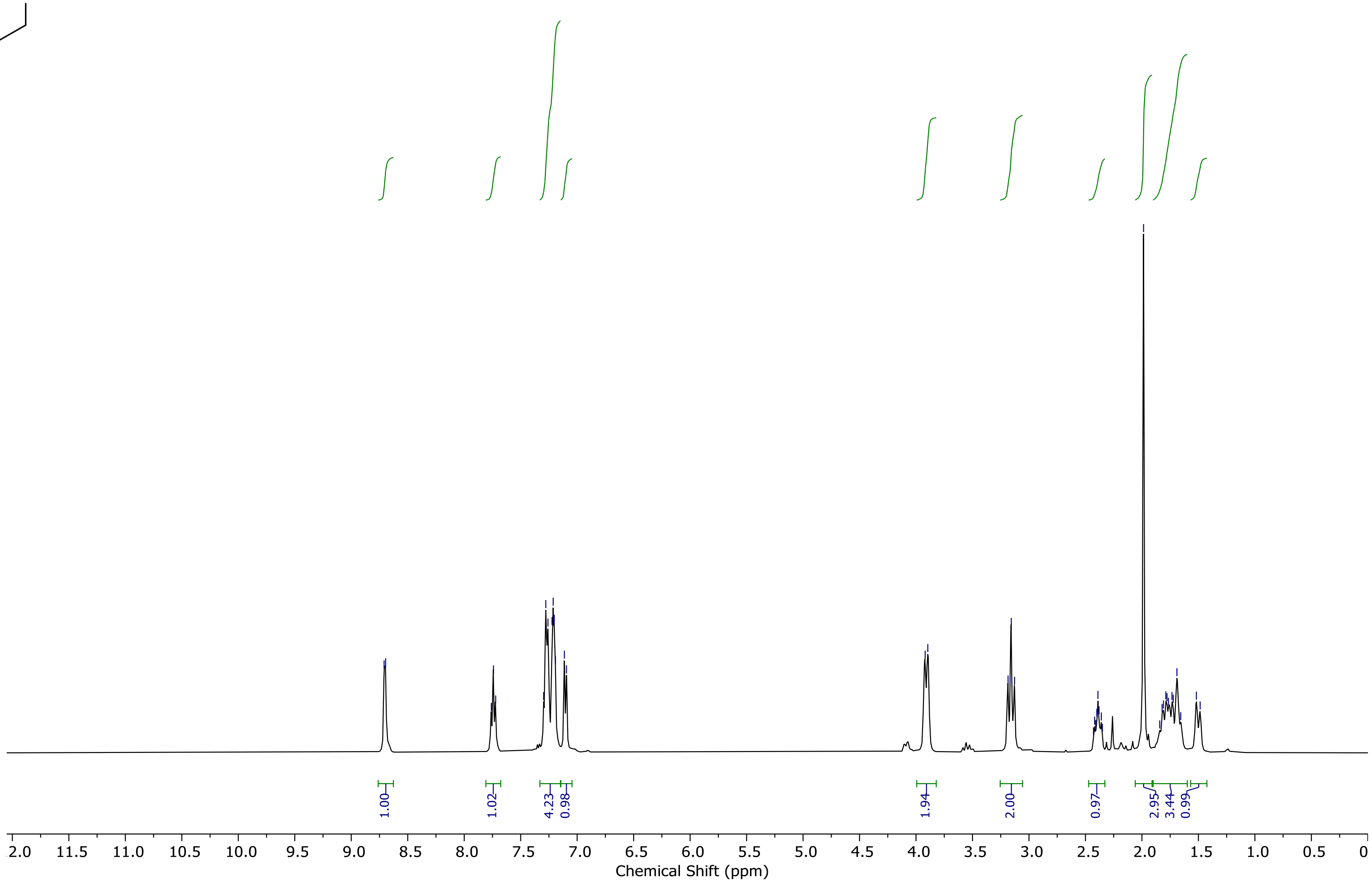

^{13}C NMR (101 MHz, $CDCl_3$) of 2-(2-methyl-6-(tetrahydro-2H-pyran-4-yl)phenyl)pyridine 15e

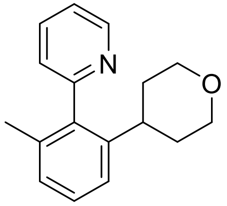

15e

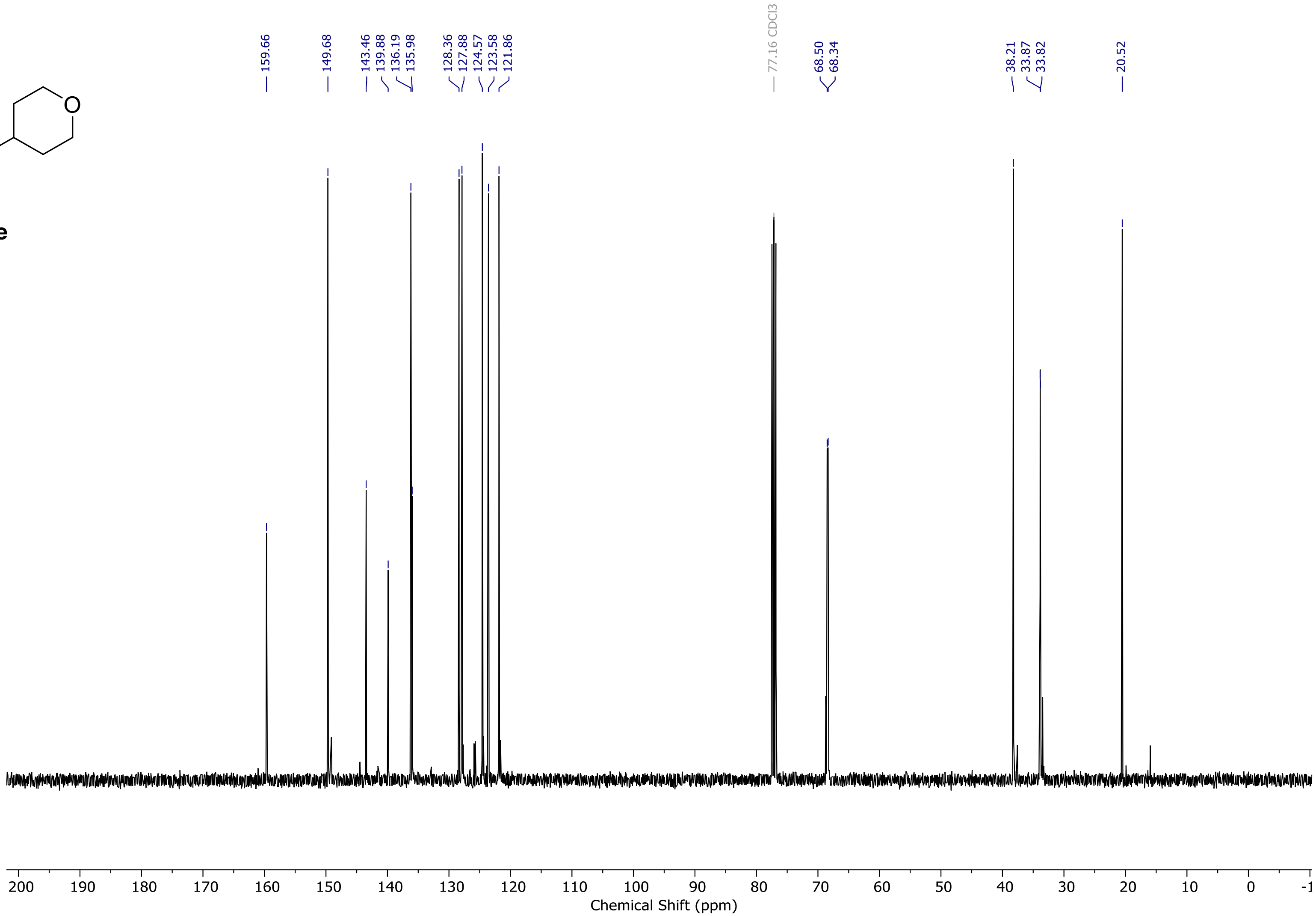

^{1}H NMR (400 MHz, $CDCl_3$) of 2-(2-fluoro-6-(tetrahydro-2H-pyran-4-yl)phenyl)pyridine 15f

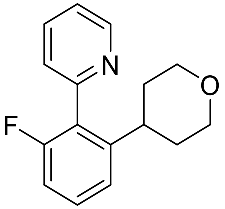

15f

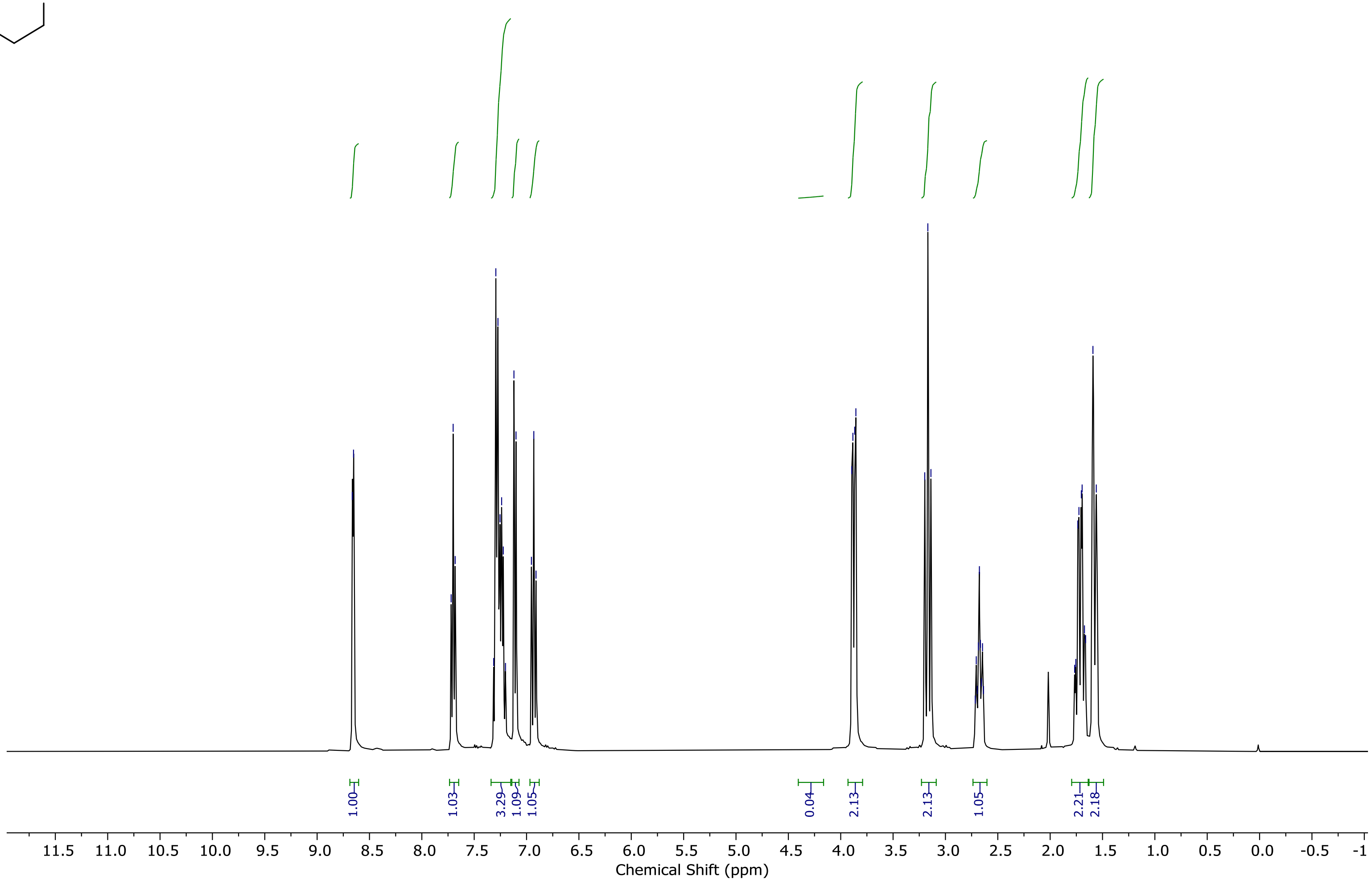

^{13}C NMR (101 MHz, $CDCl_3$) of 2-(2-fluoro-6-(tetrahydro-2H-pyran-4-yl)phenyl)pyridine 15f

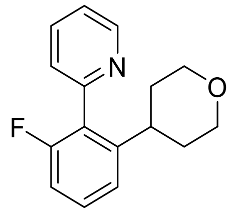

15f

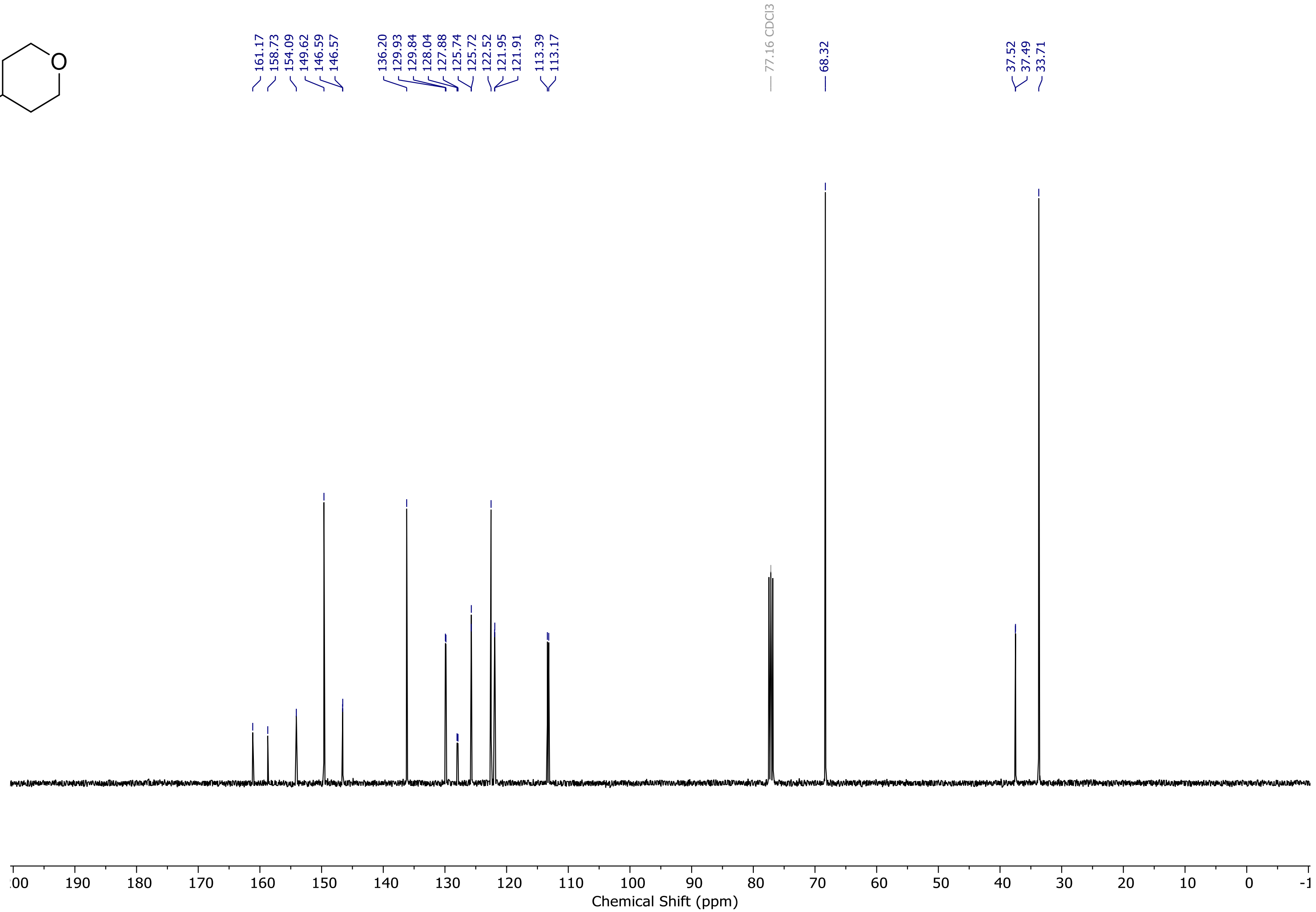

^{19}F NMR (376 MHz, $CDCl_3$) of 2-(2-fluoro-6-(tetrahydro-2H-pyran-4-yl)phenyl)pyridine 15f

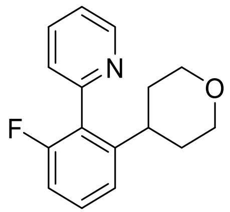

15f

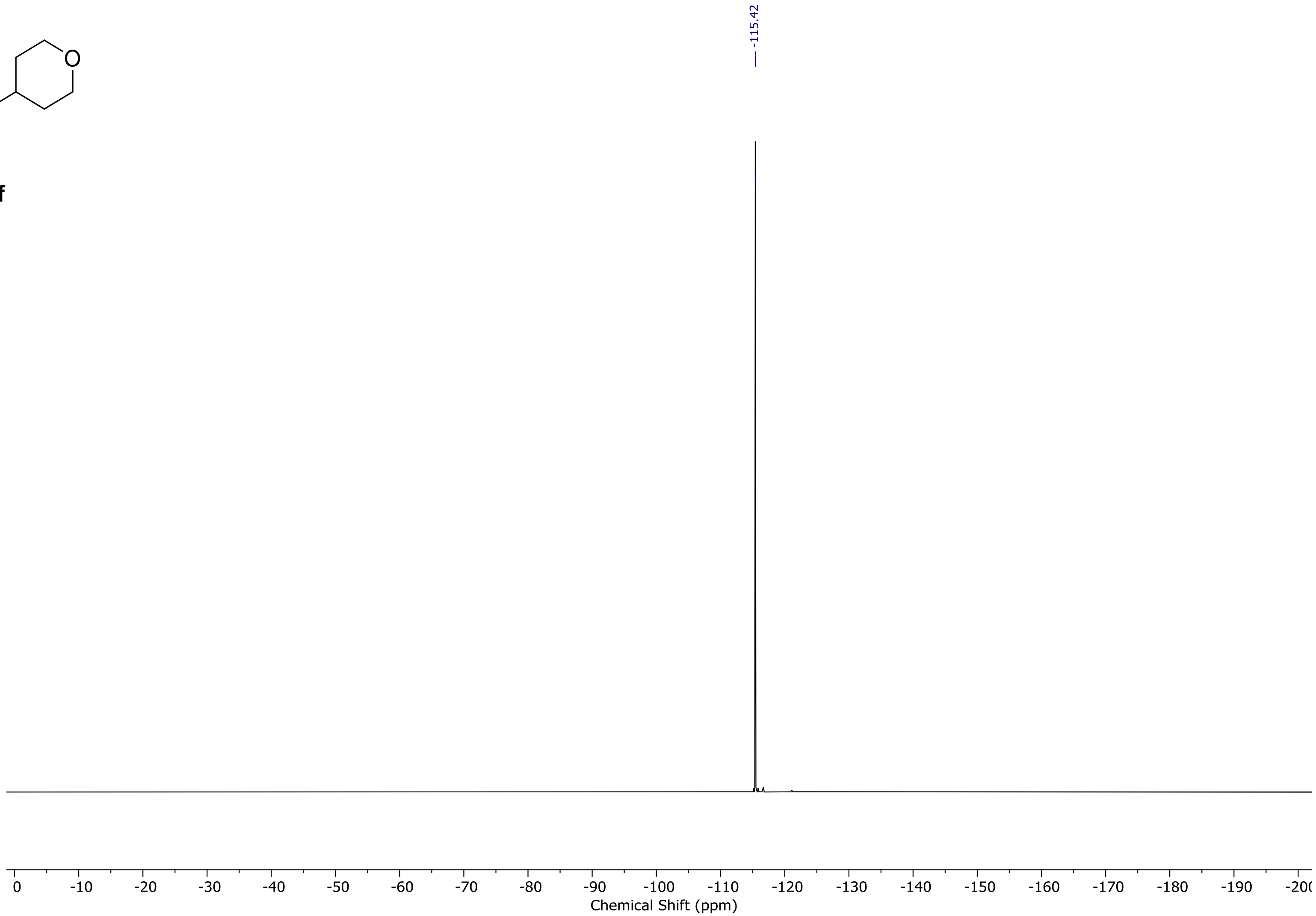

^{1}H NMR (500 MHz, $CDCl_3$) of 3-methyl-2-(2-(tetrahydro-2H-pyran-4-yl)phenyl)pyridine 15g

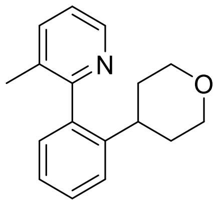

15g

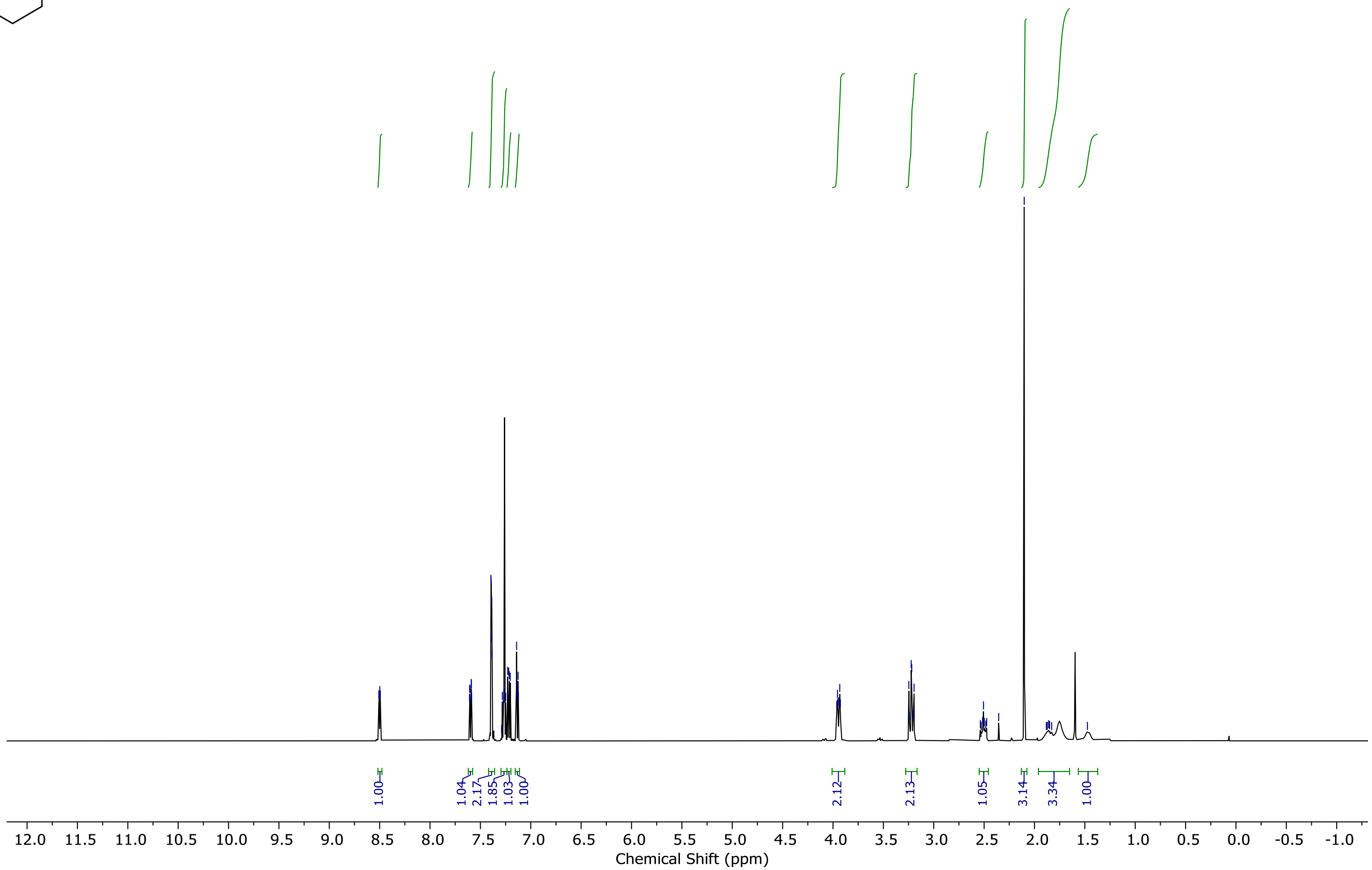

^{13}C NMR (126 MHz, CDCl$_3$) of 3-methyl-2-(2-(tetrahydro-2H-pyran-4-yl)phenyl)pyridine 15g

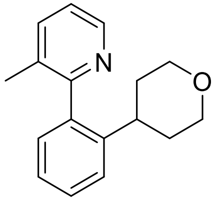

15g

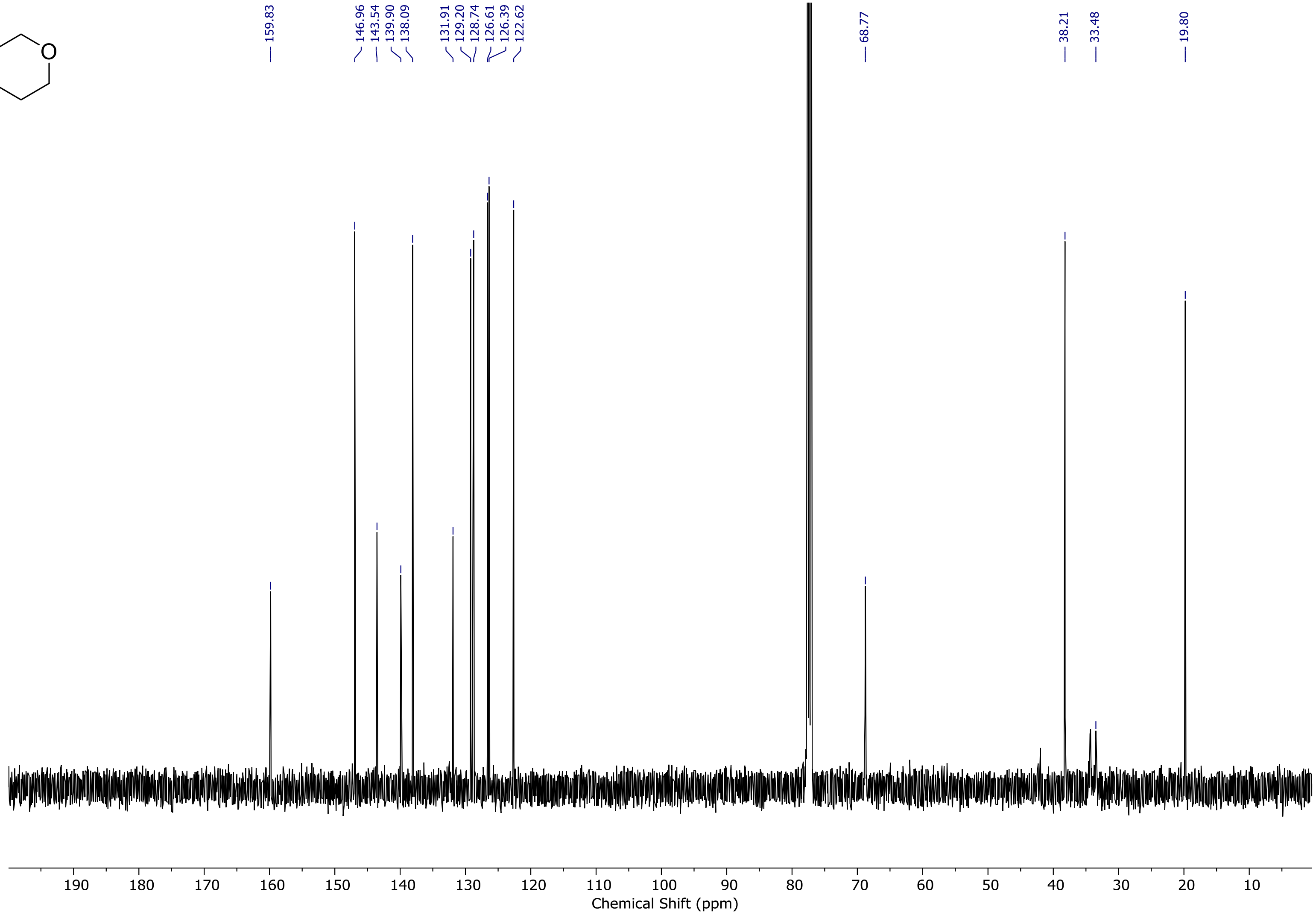

^{1}H NMR (400 MHz, CDCl$_3$) of *tert*-butyl 4-(3-methyl-2-(pyridin-2-yl)phenyl)piperidine-1-carboxylate 15h

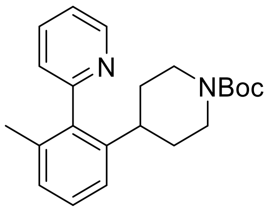

15h

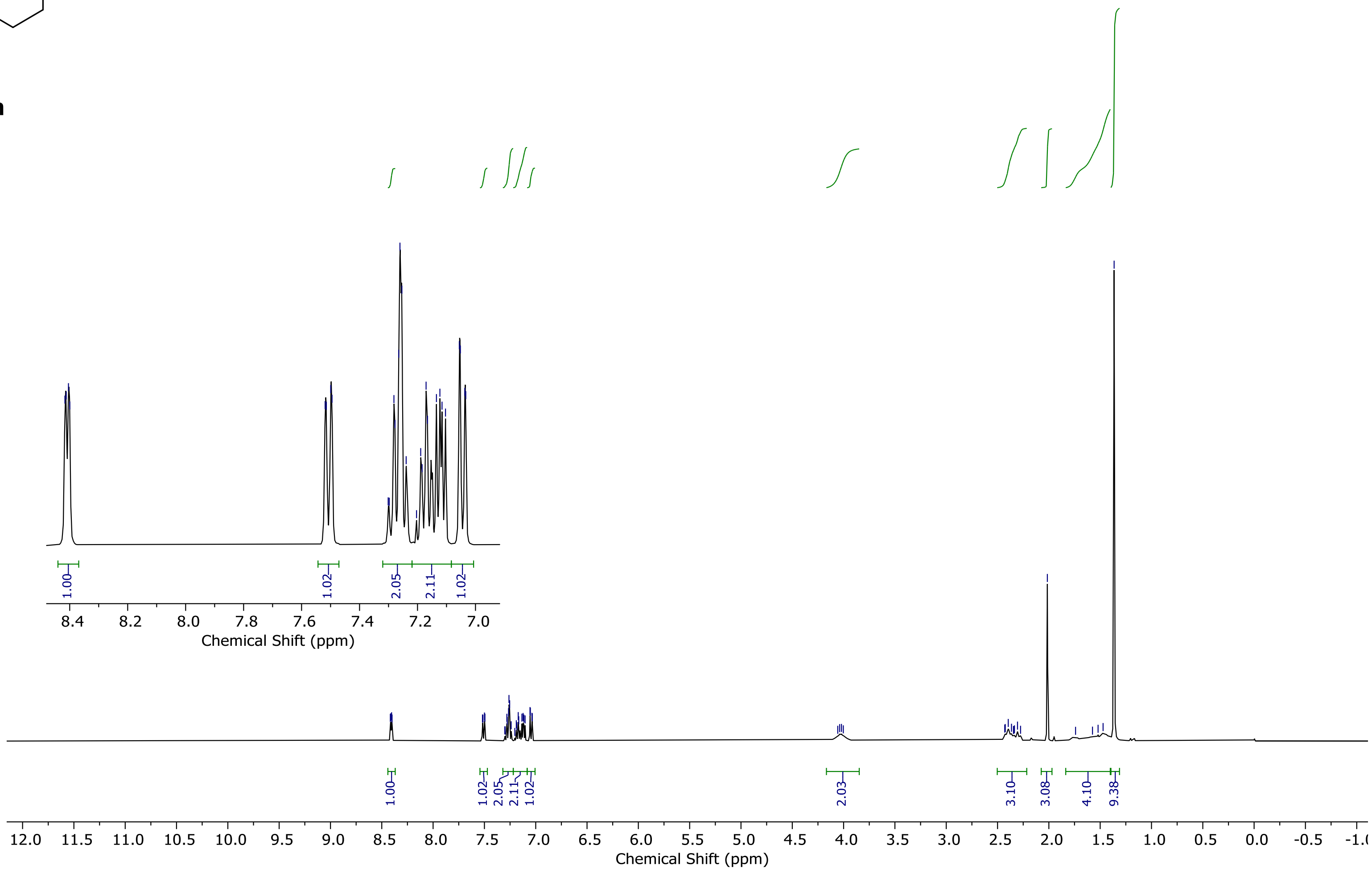

^{13}C NMR (101 MHz, CDCl$_3$) of *tert*-butyl 4-(3-methyl-2-(pyridin-2-yl)phenyl)piperidine-1-carboxylate 15h

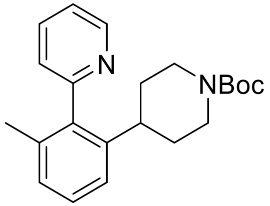

15h

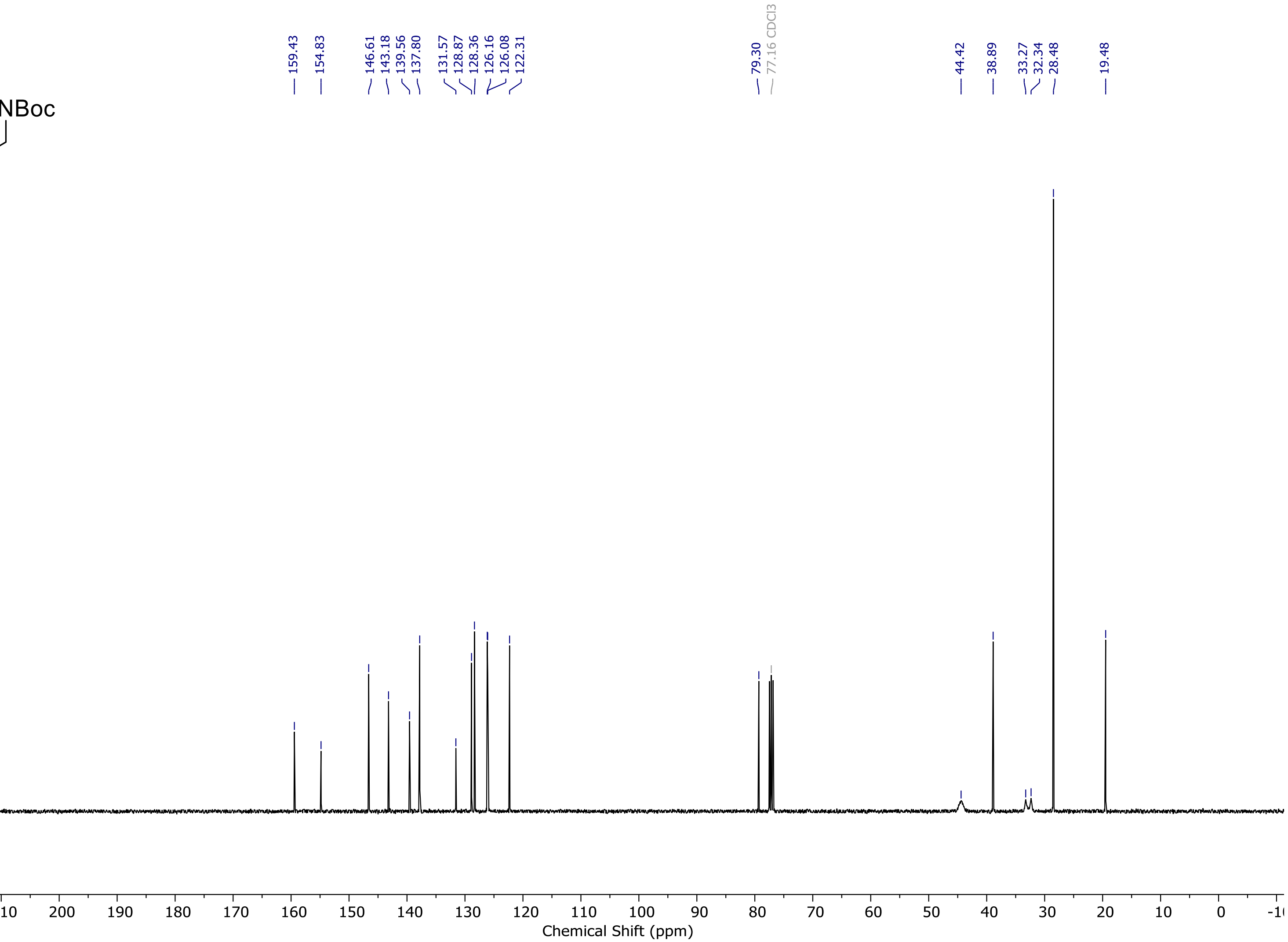

^{1}H NMR (500 MHz, $CDCl_3$) of furan-2-yl(4-(3-methyl-2-(pyridin-2-yl)phenyl)piperidin-1-yl)methanone 15i

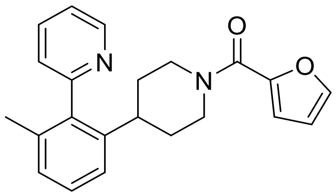

15i

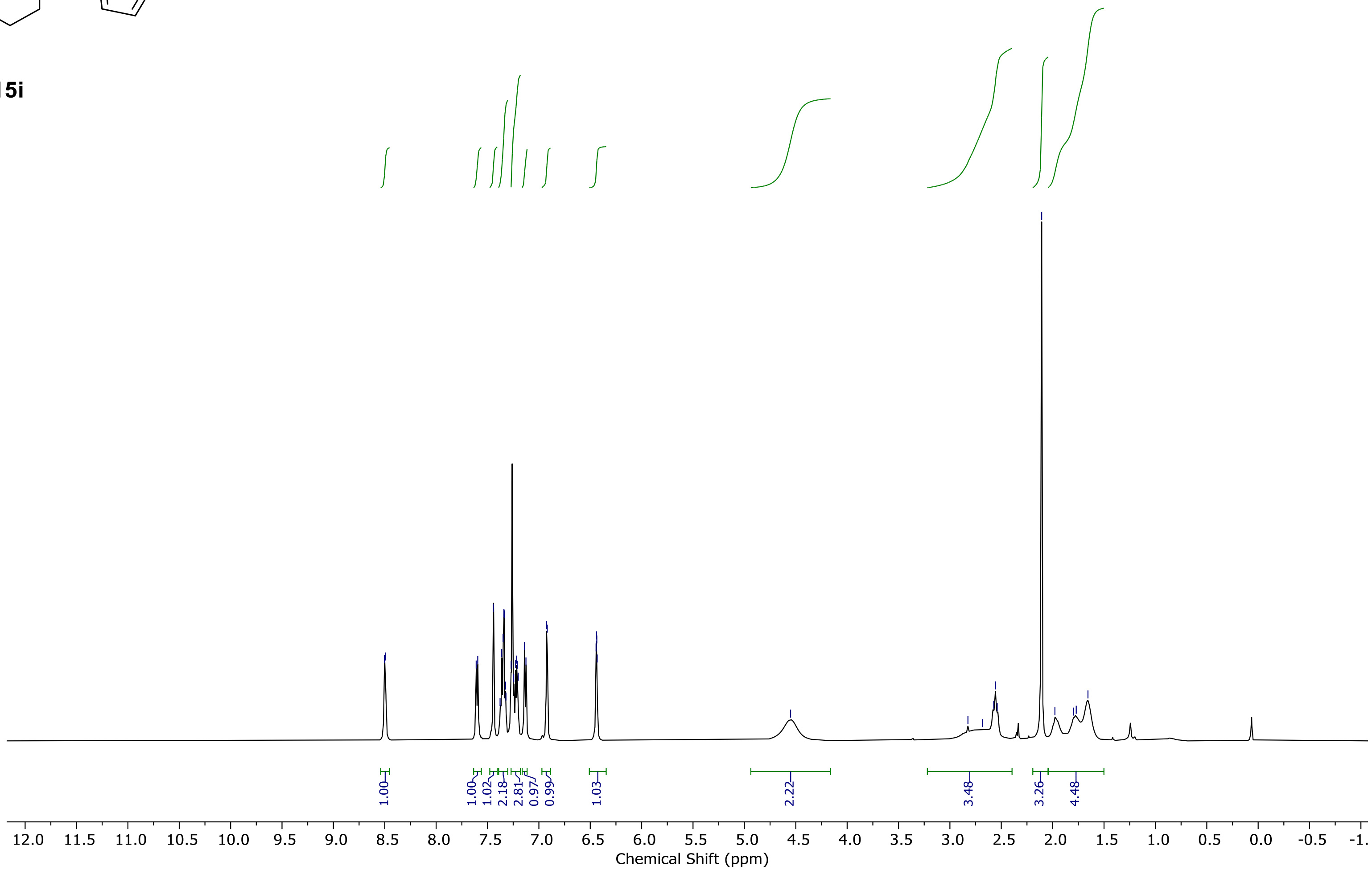

^{13}C NMR (101 MHz, $CDCl_3$) of furan-2-yl(4-(3-methyl-2-(pyridin-2-yl)phenyl)piperidin-1-yl)methanone 15i

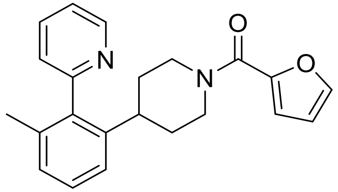

15i

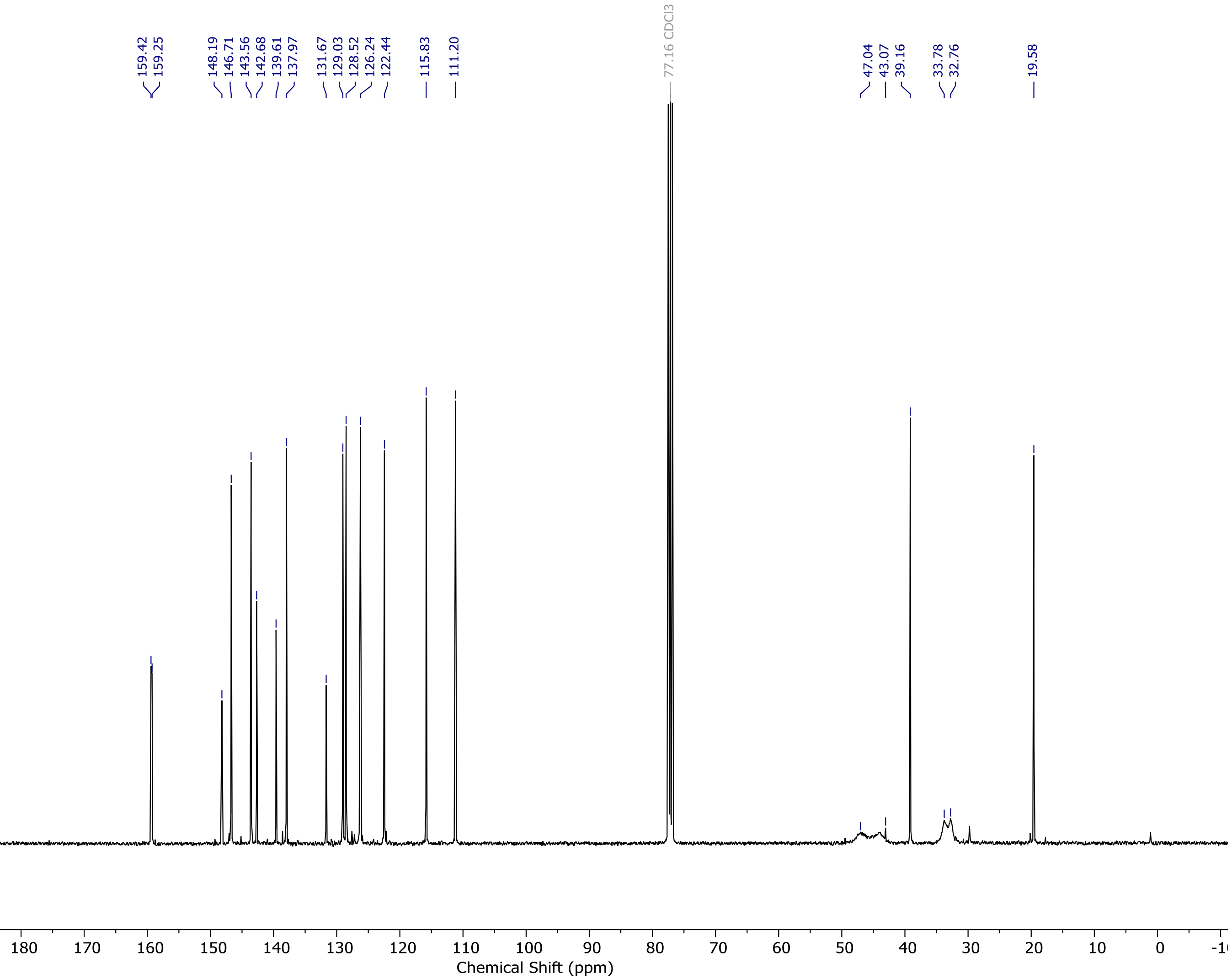

^{1}H NMR (400 MHz, $CDCl_3$) of 1-(2-(tetrahydro-2H-pyran-4-yl)phenyl)isoquinoline 15j

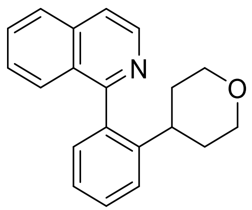

15j

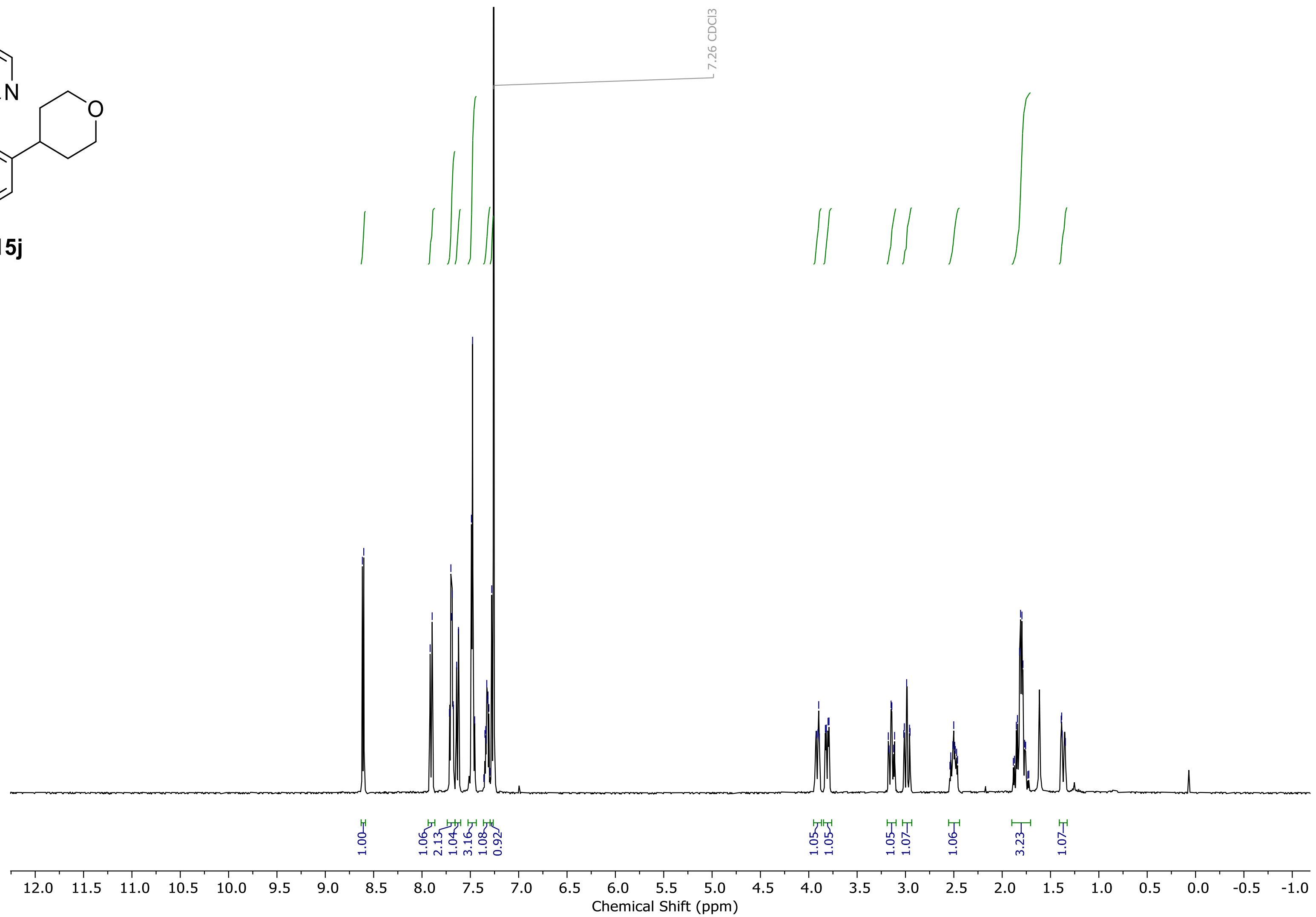

^{13}C NMR (400 MHz, $CDCl_3$) of 1-(2-(tetrahydro-2H-pyran-4-yl)phenyl)isoquinoline 15j

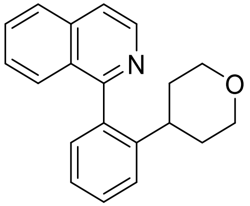

15j

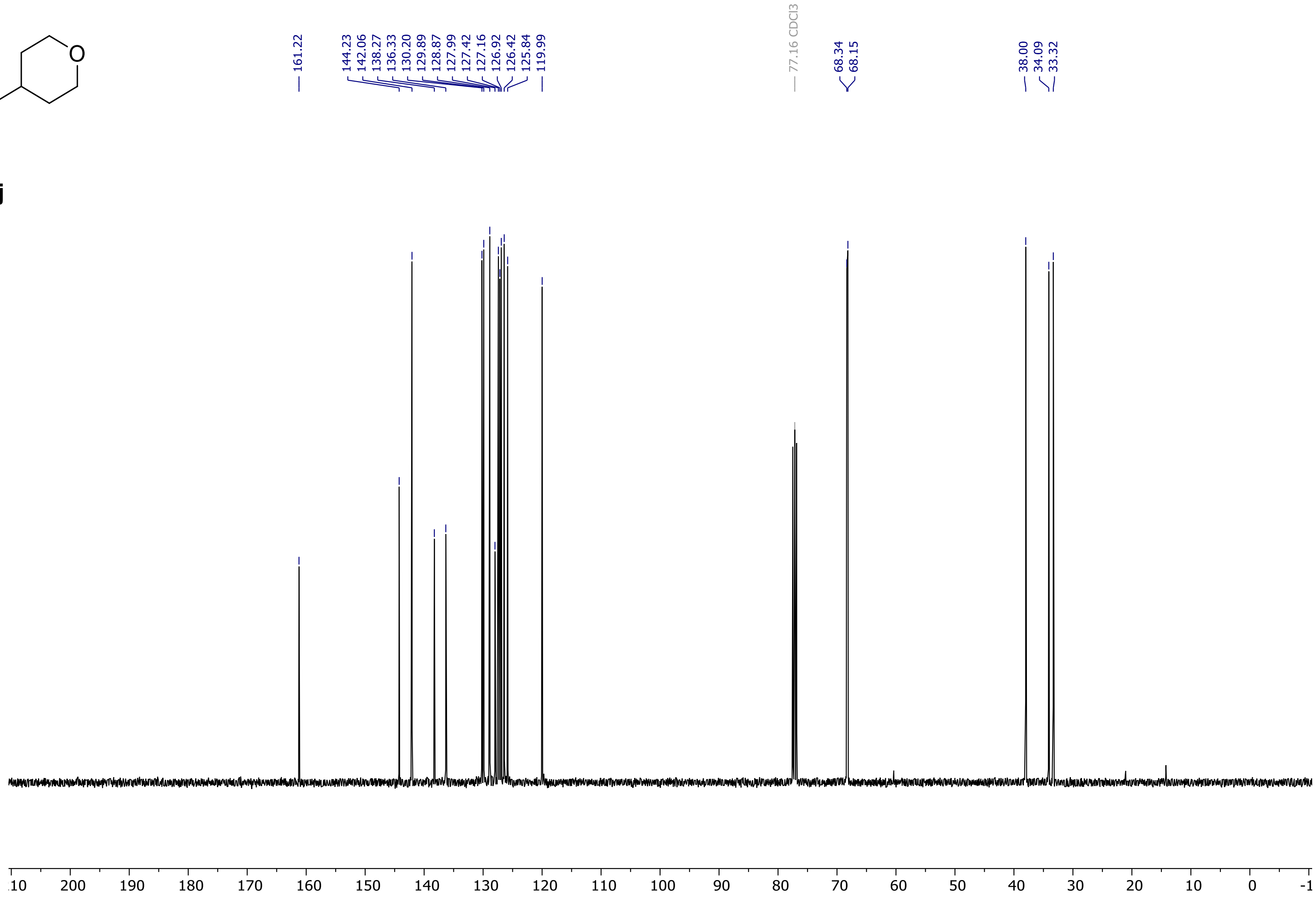

^{1}H NMR (500 MHz, $CDCl_3$) of Starting Material 8k

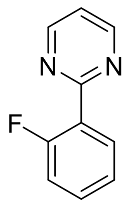

8k

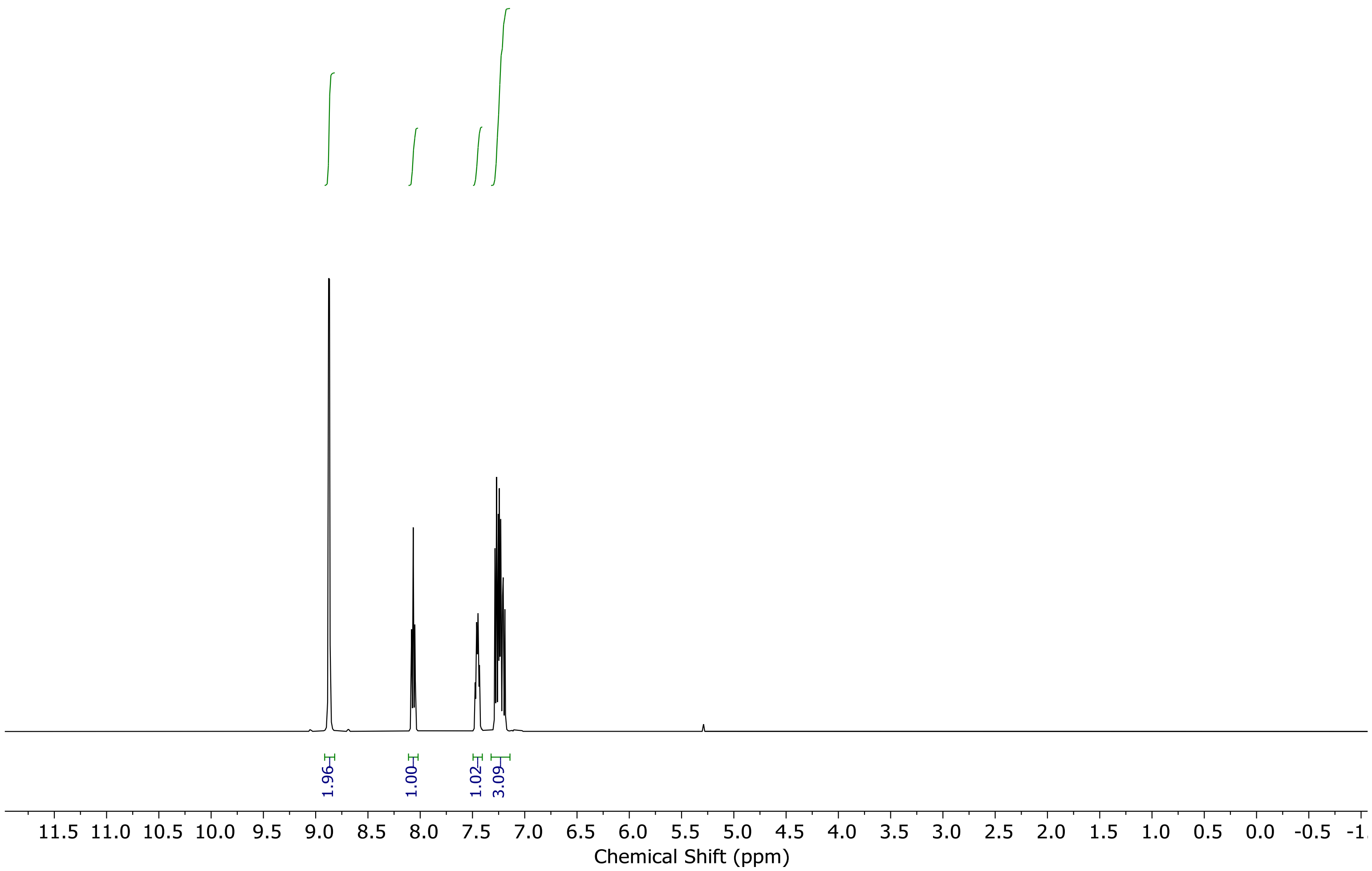

^{13}C NMR (126 MHz, $CDCl_3$) of Starting Material 8k

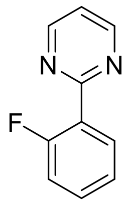

8k

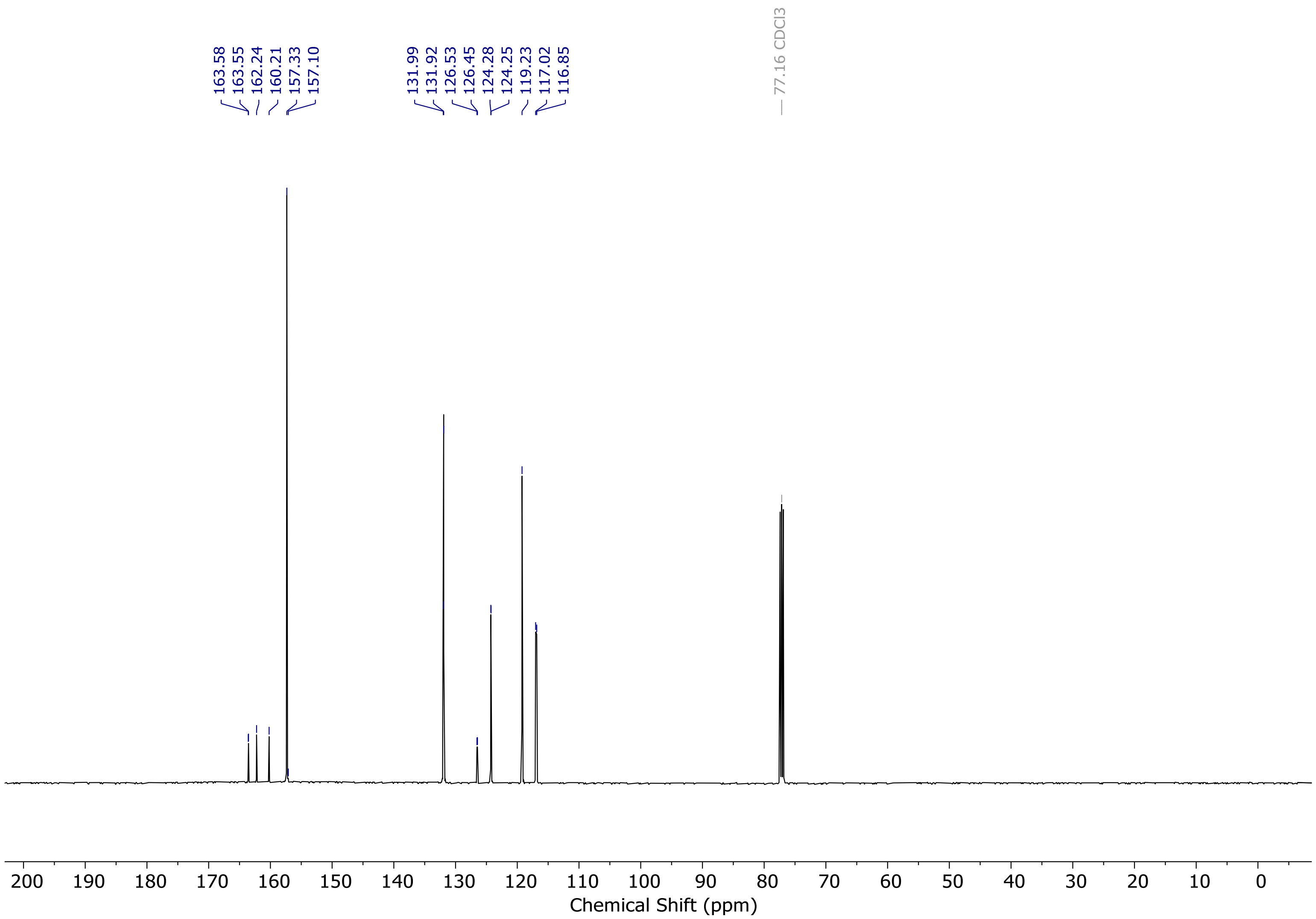

^{19}F NMR (471 MHz, CDCl₃) of Starting Material 8k

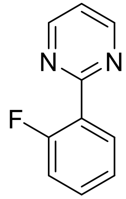

8k

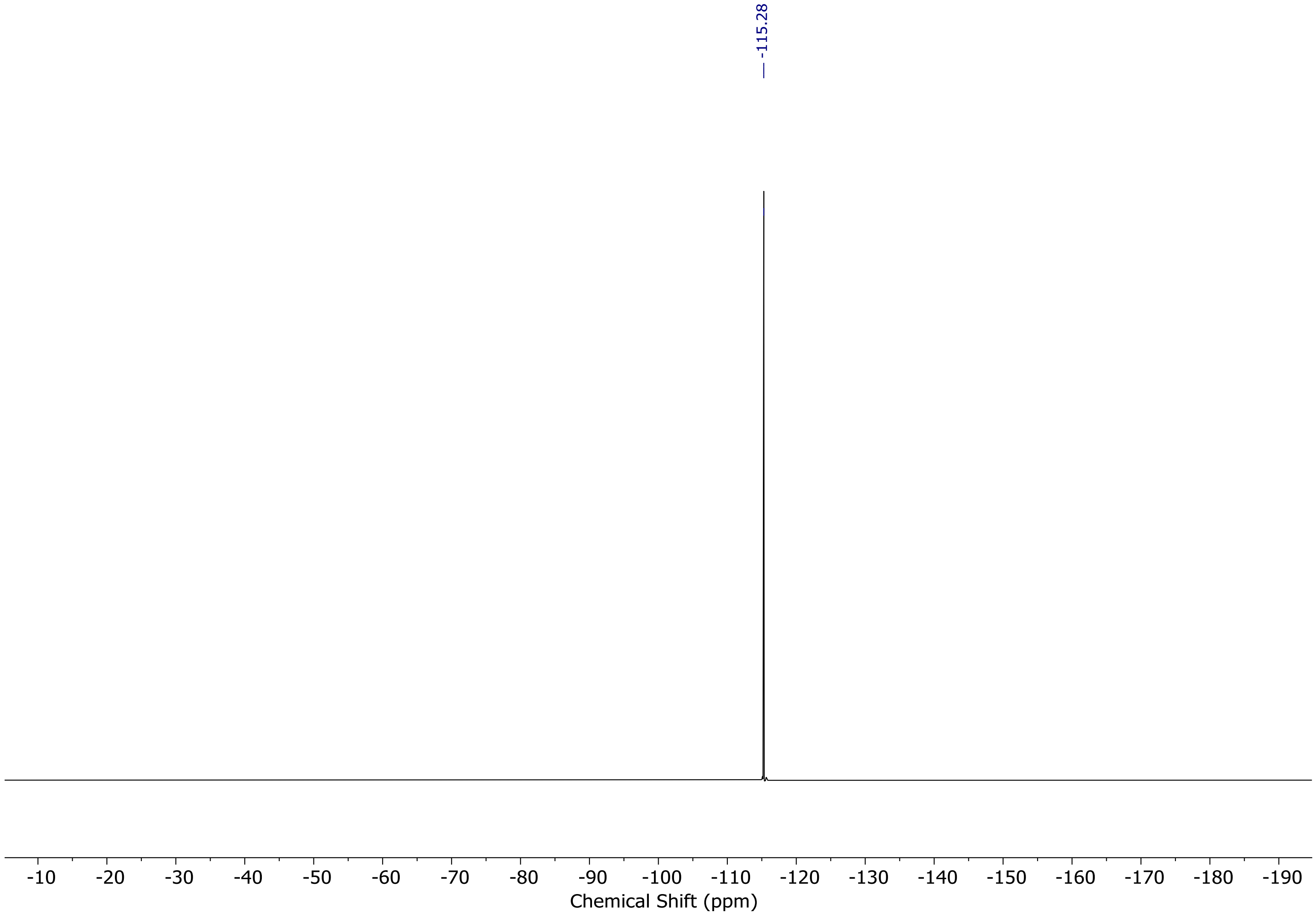

^{1}H NMR (400 MHz, $CDCl_3$) of Starting Material 8l

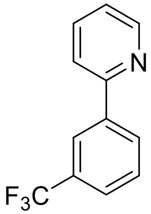

8l

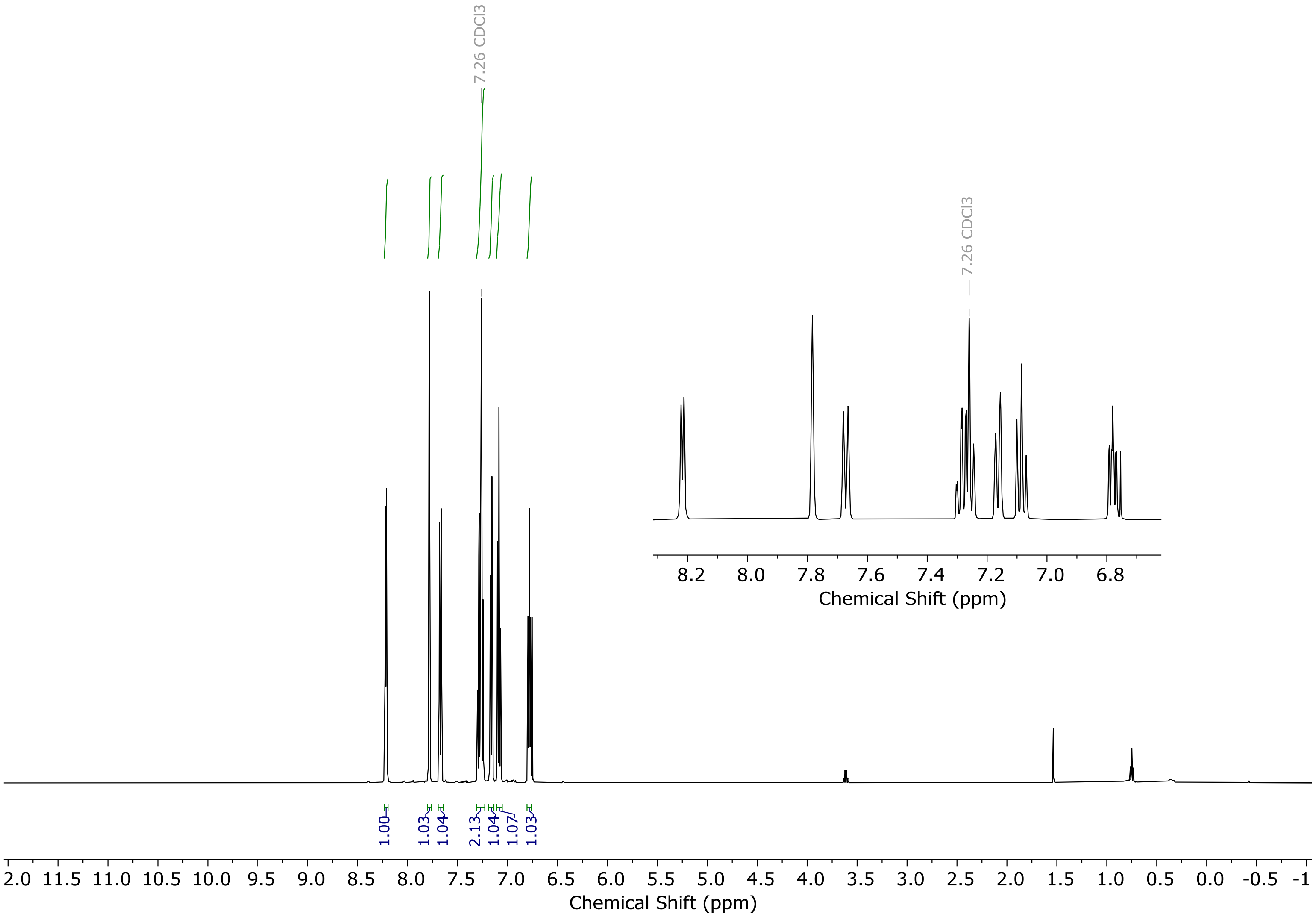

^{13}C NMR (126 MHz, CDCl₃) of Starting Material 8l

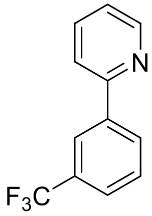

8l

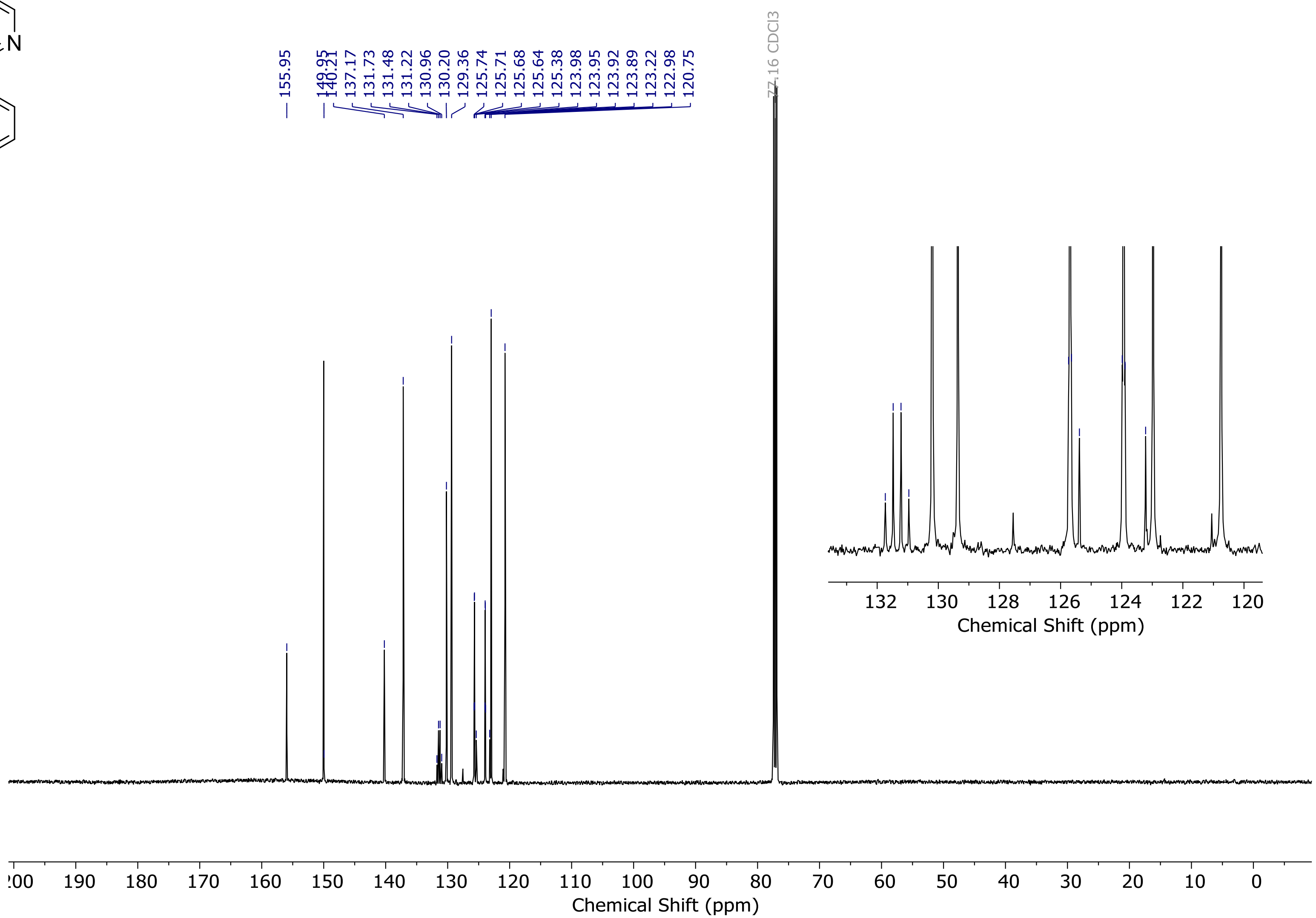

^{19}F NMR (471 MHz, $CDCl_3$) of Starting Material 8l

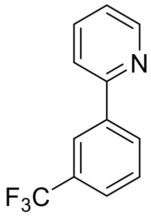

8l

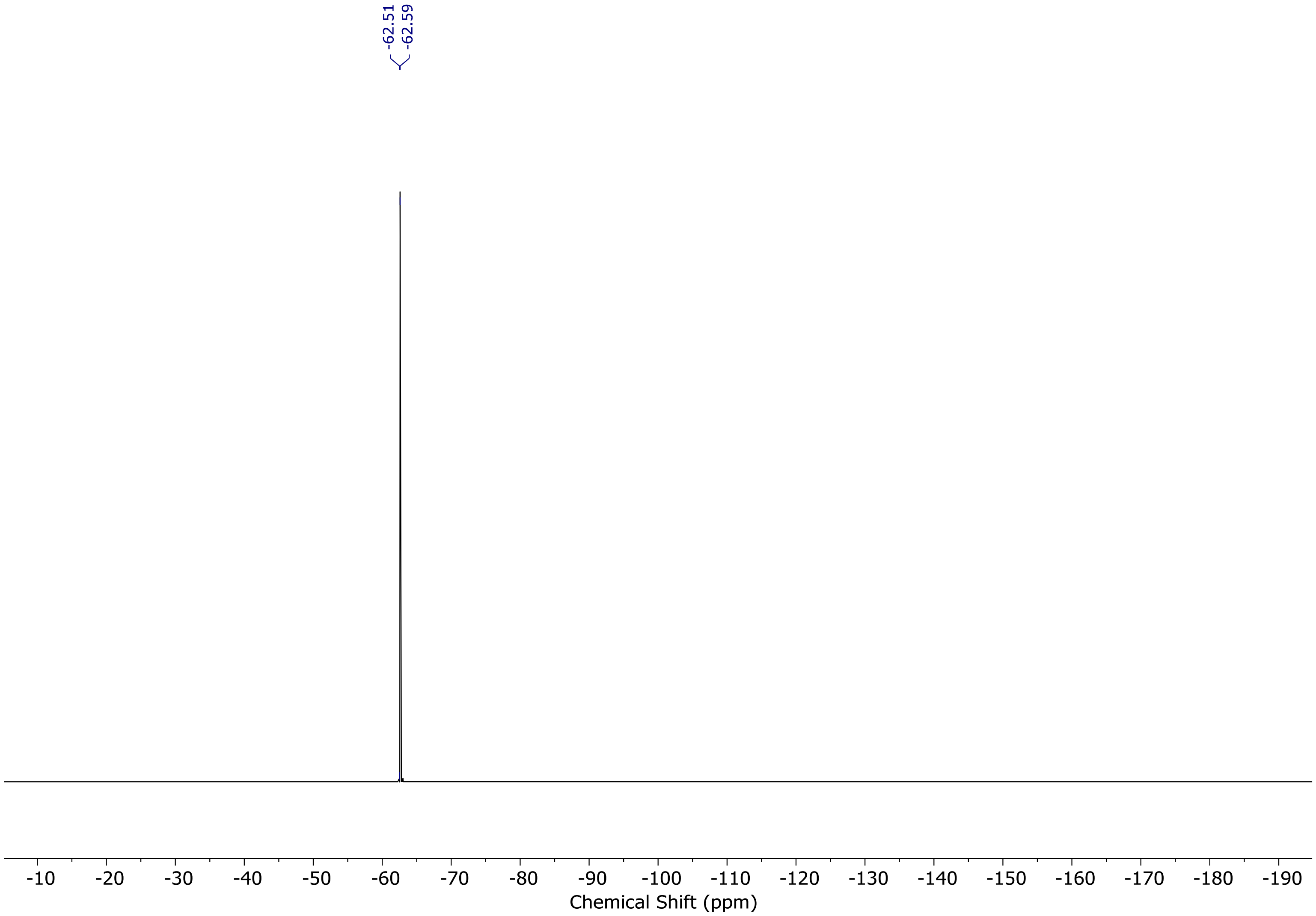

^{1}H NMR (400 MHz, $CDCl_3$) of Starting Material 8m

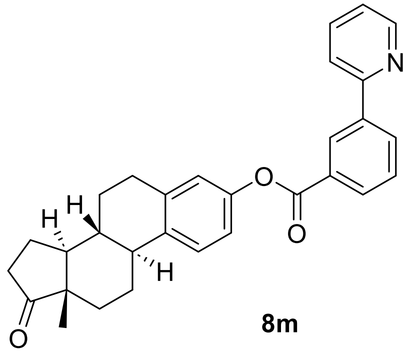

8m

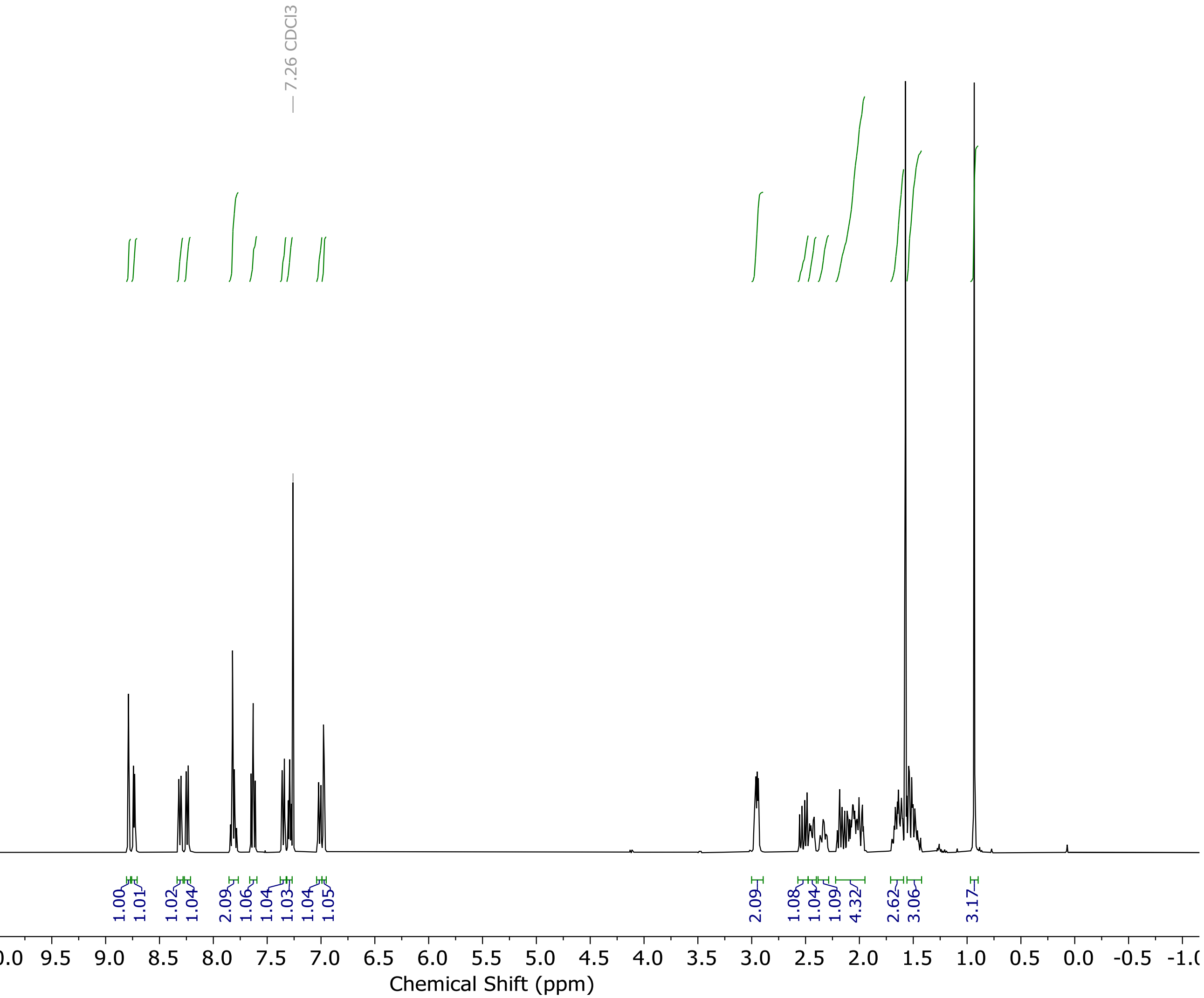

^{13}C NMR (126 MHz, CDCl₃) of Starting Material 8m

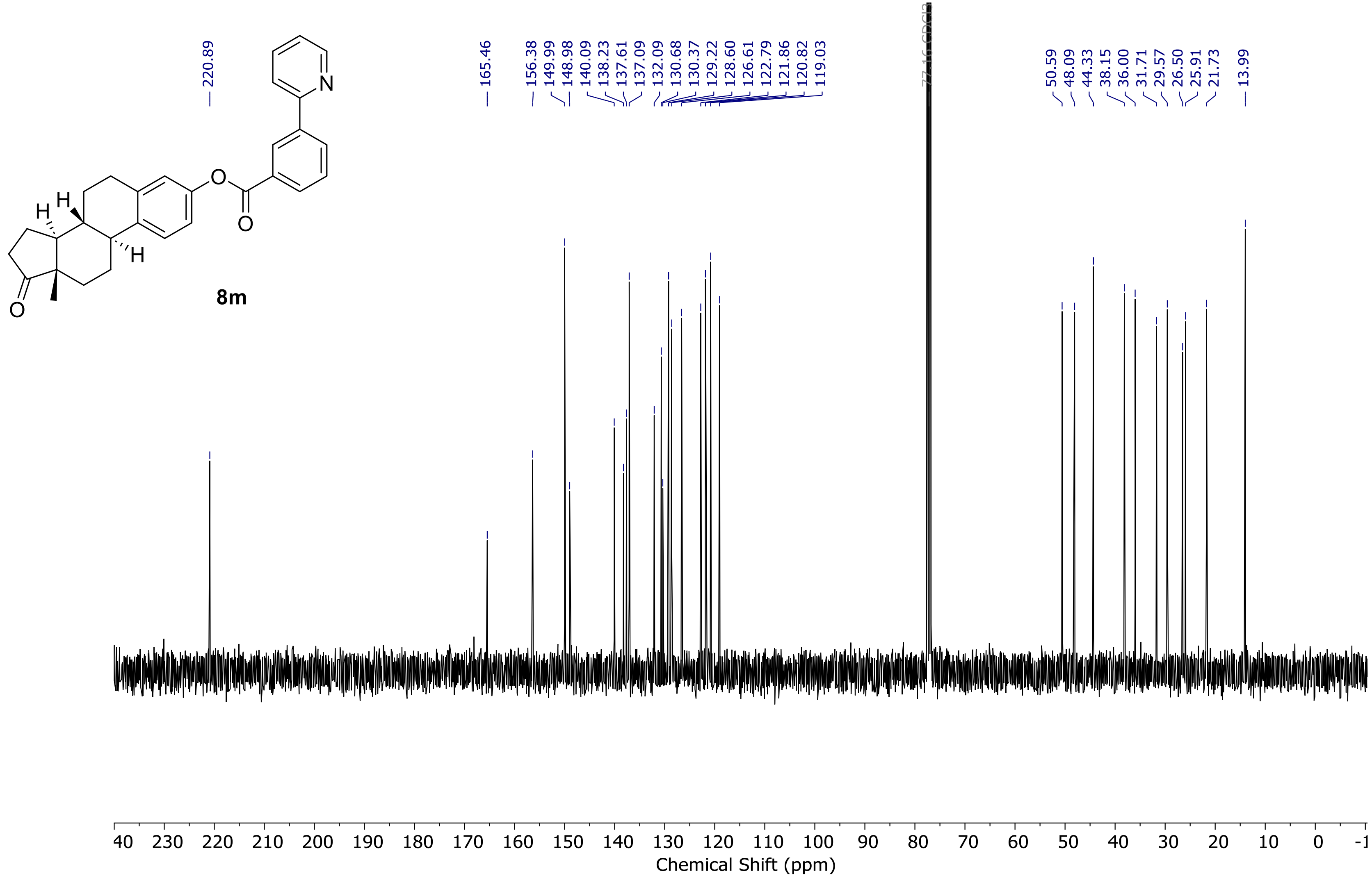

^{1}H NMR (400 MHz, $CDCl_3$) of Starting Material 8s

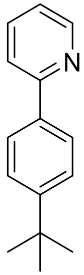

8s

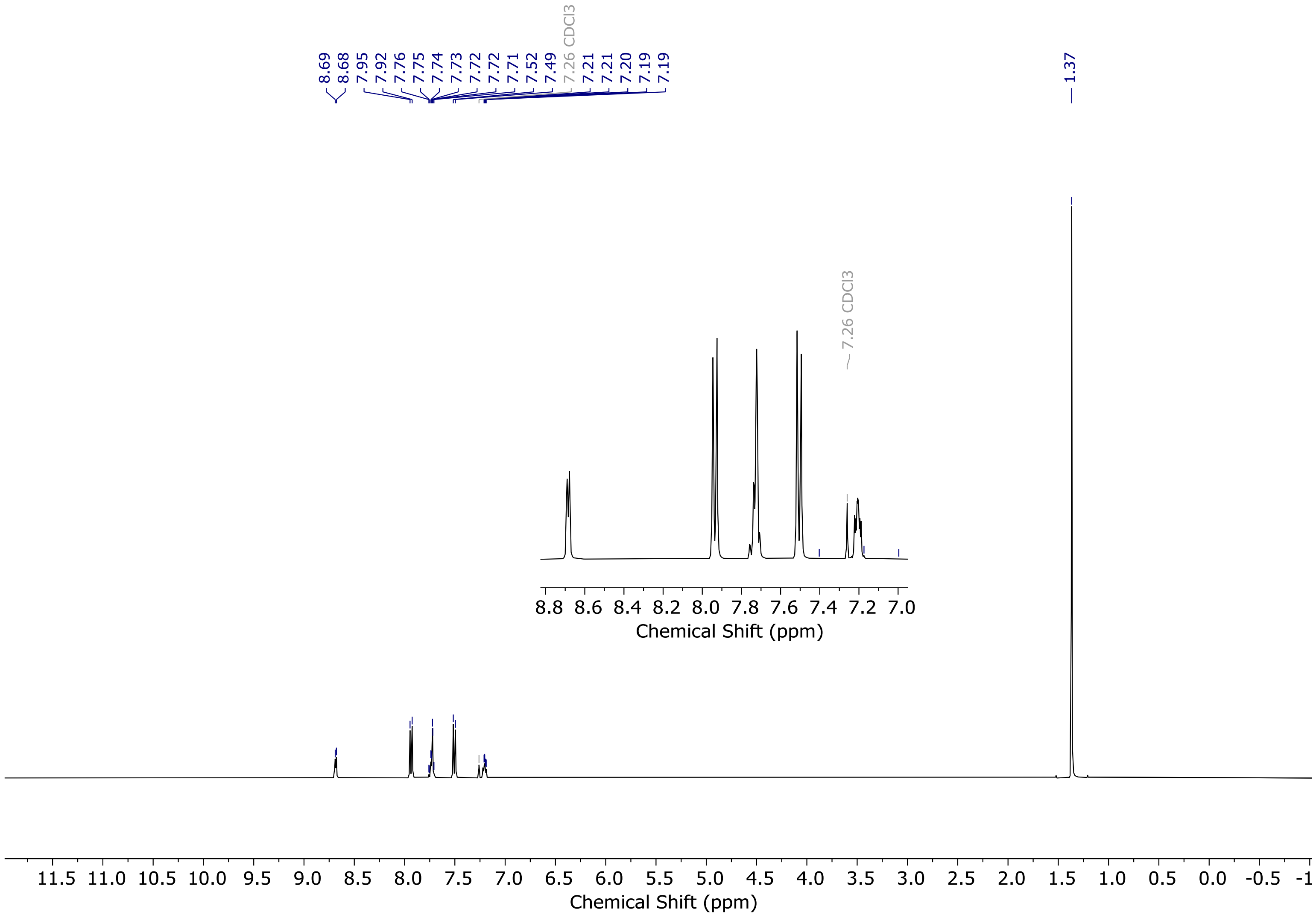

^{13}C NMR (126 MHz, CDCl3) of Starting Material 8s

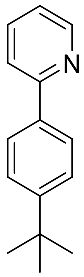

8s

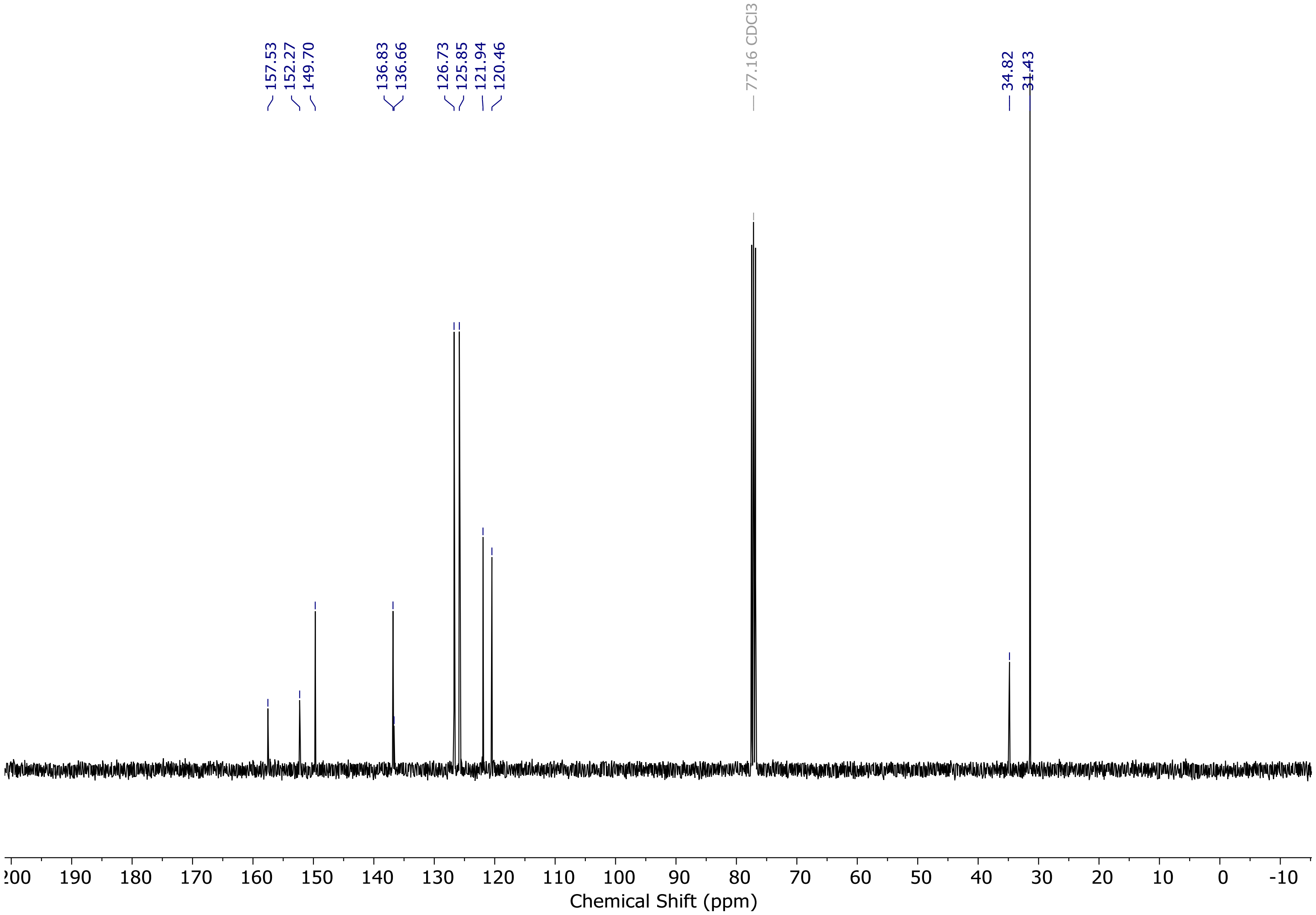

^{1}H NMR (400 MHz, $CDCl_3$) of Starting Material 8t

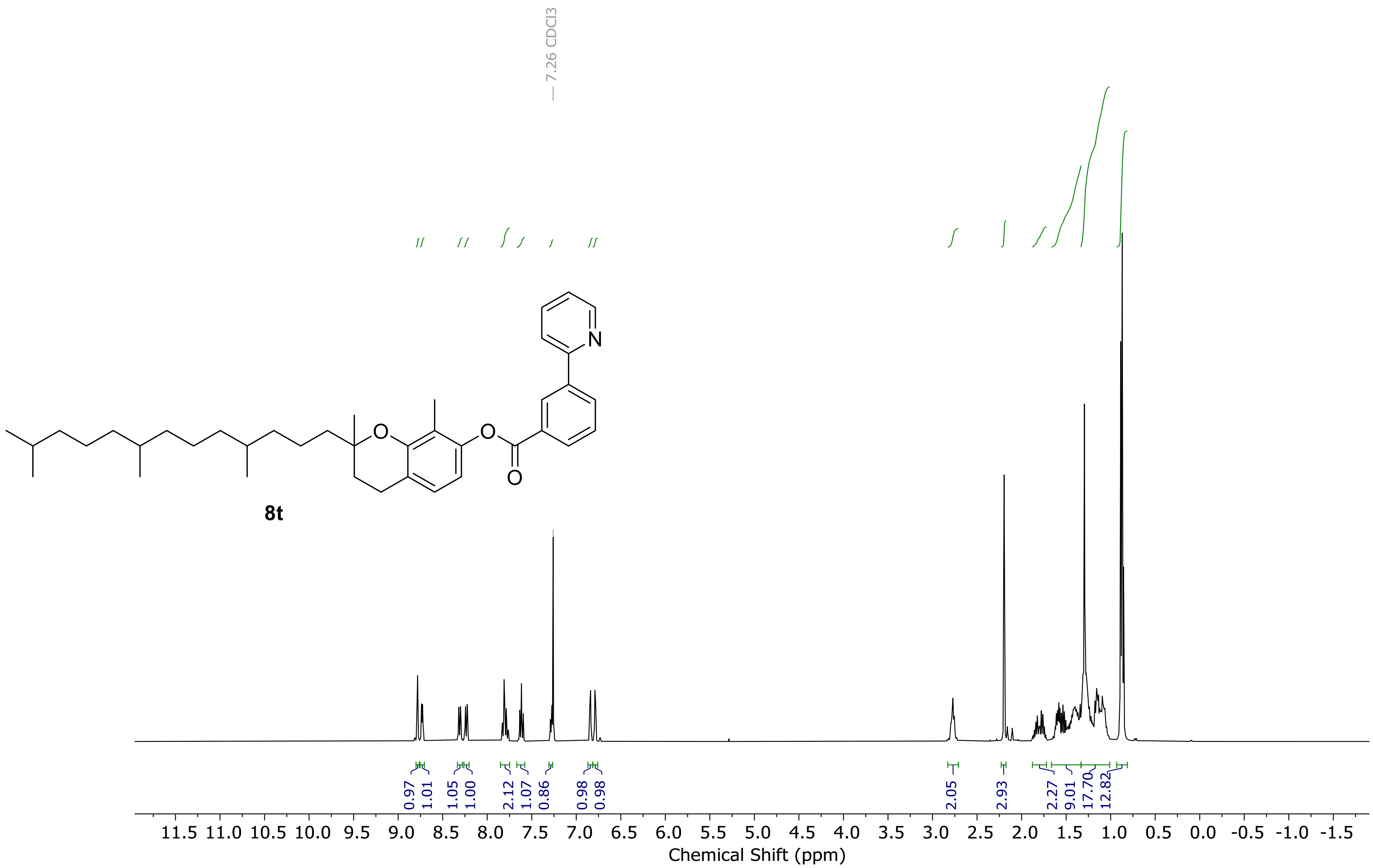

^{13}C NMR (126 MHz, CDCl3) of Starting Material 8t

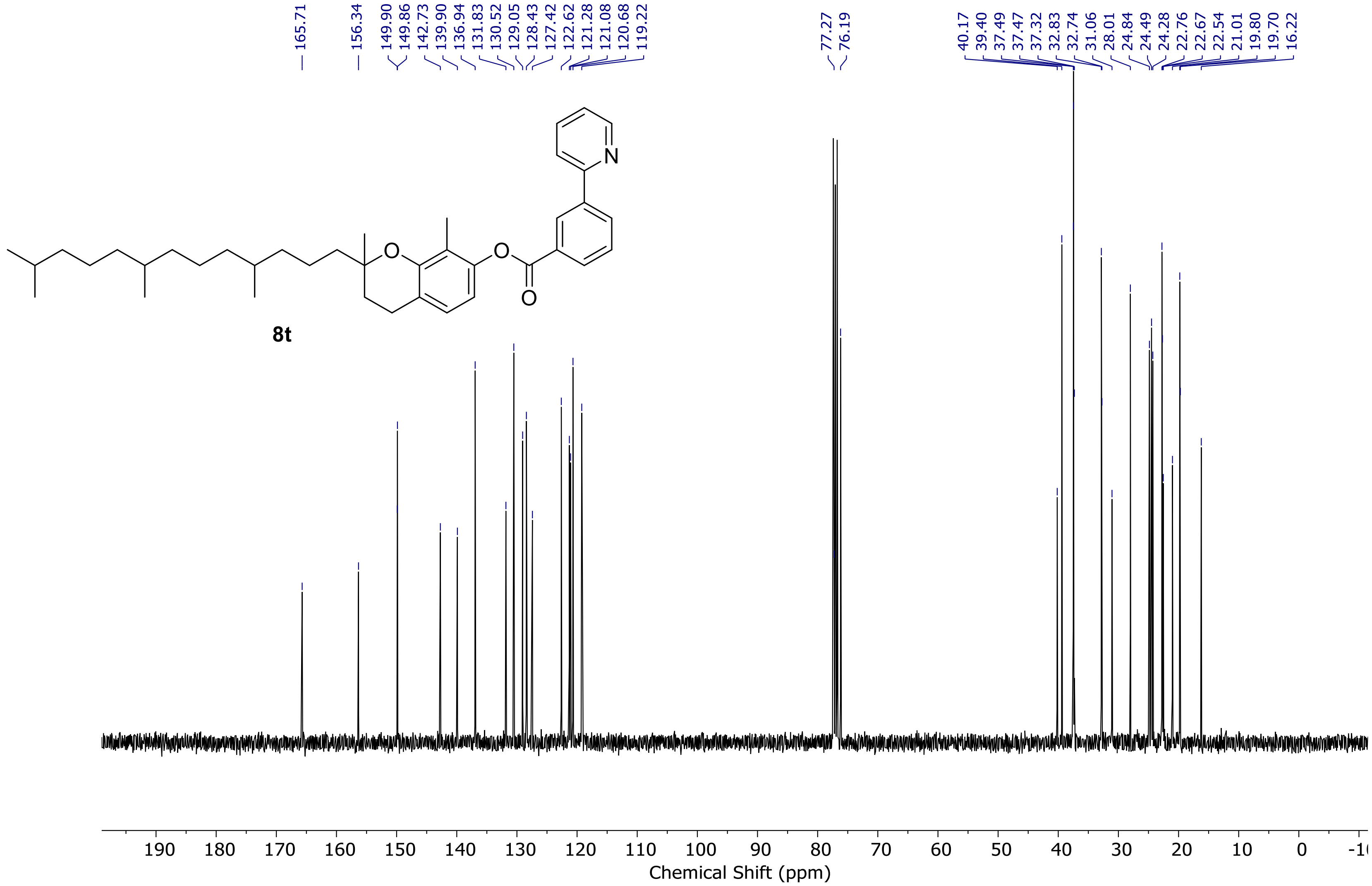

^{1}H NMR (400 MHz, d_6-Acetone) of Starting Material 16

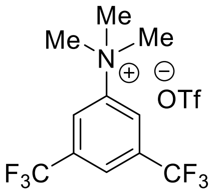

16

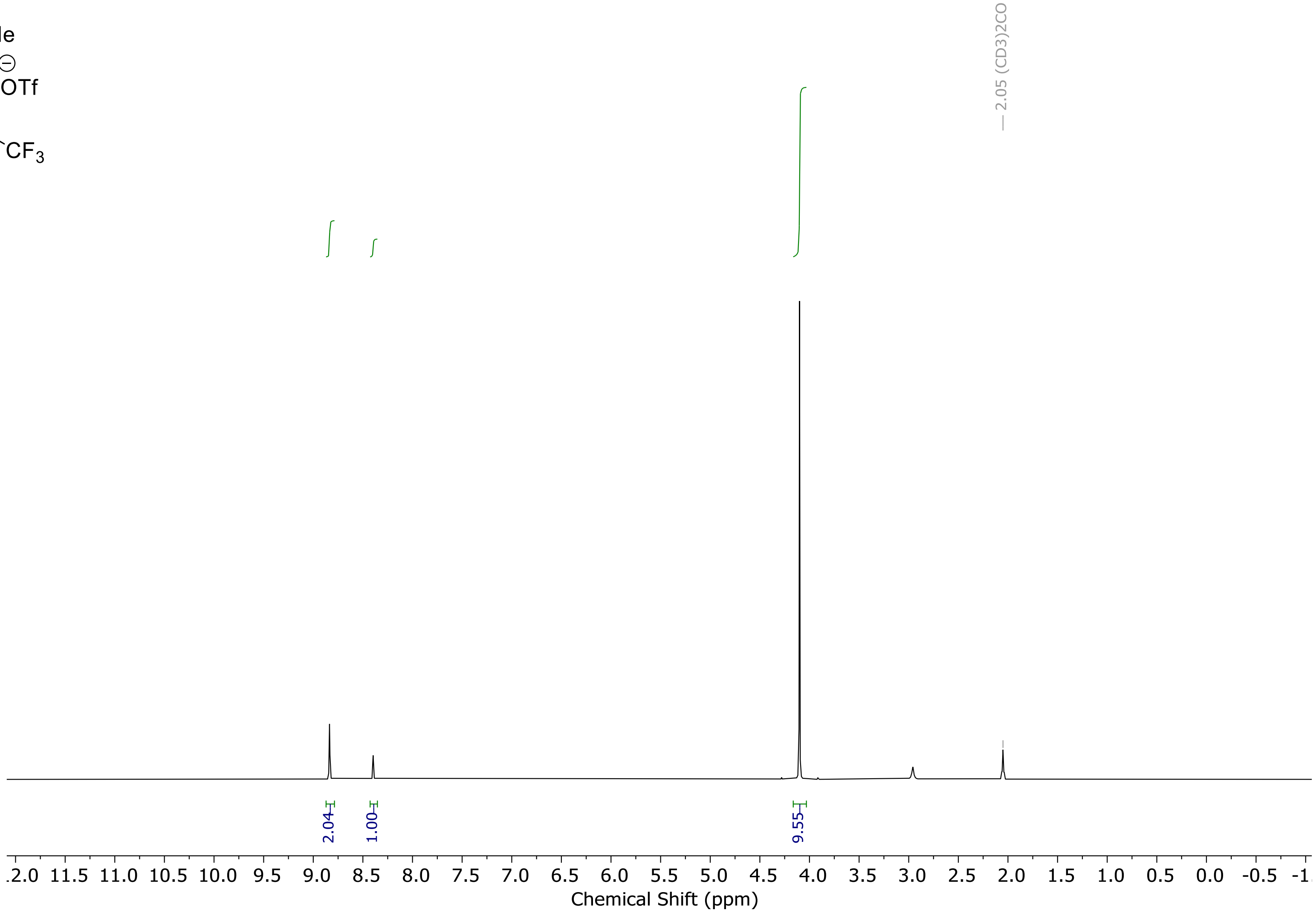

^{13}C NMR (126 MHz, d_6-Acetone) of Starting Material 16

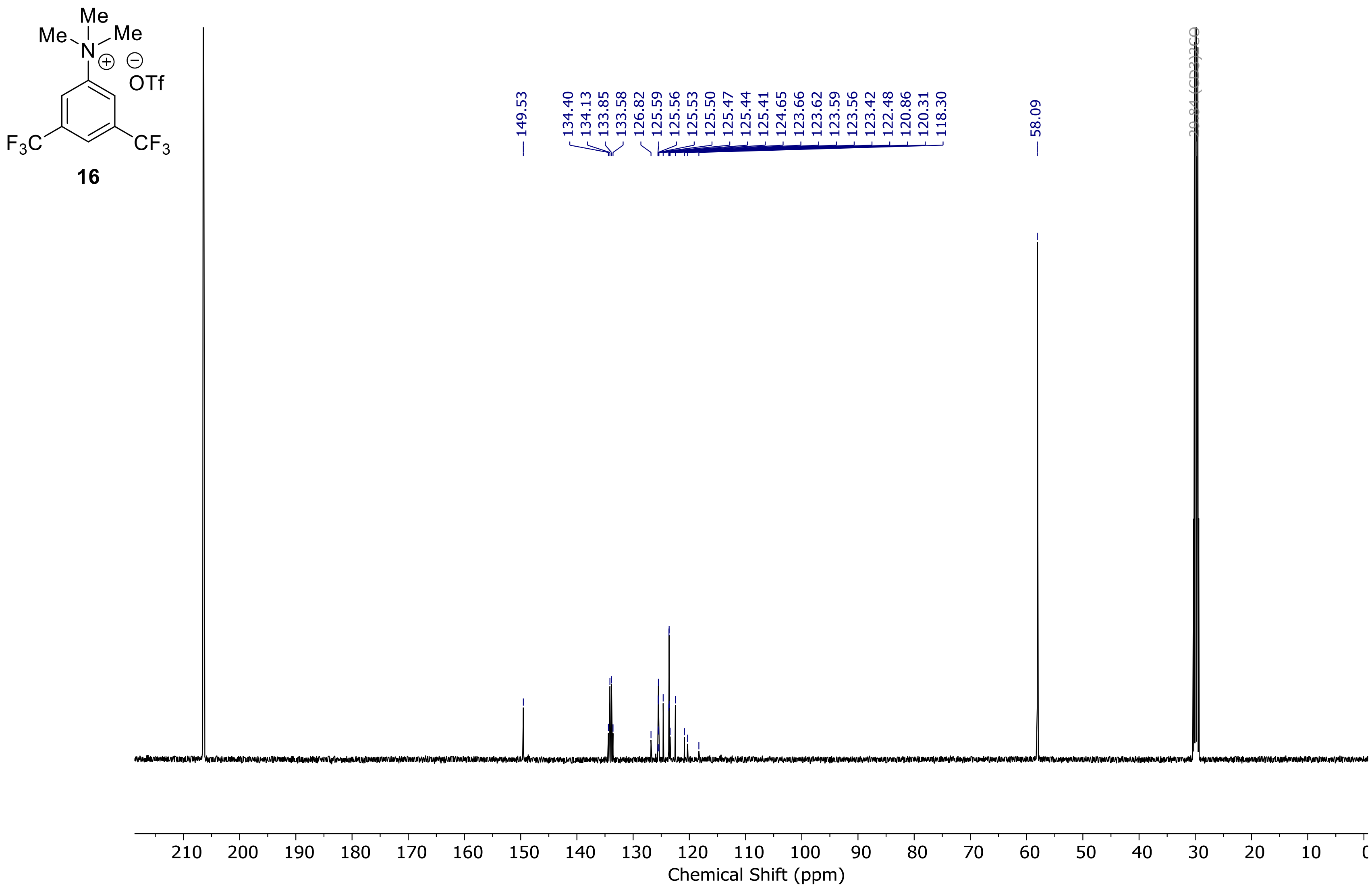

^{19}F NMR (XXX MHz, d_6-Acetone) of Starting Material 16

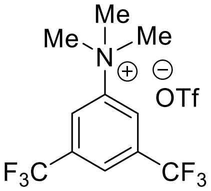

16

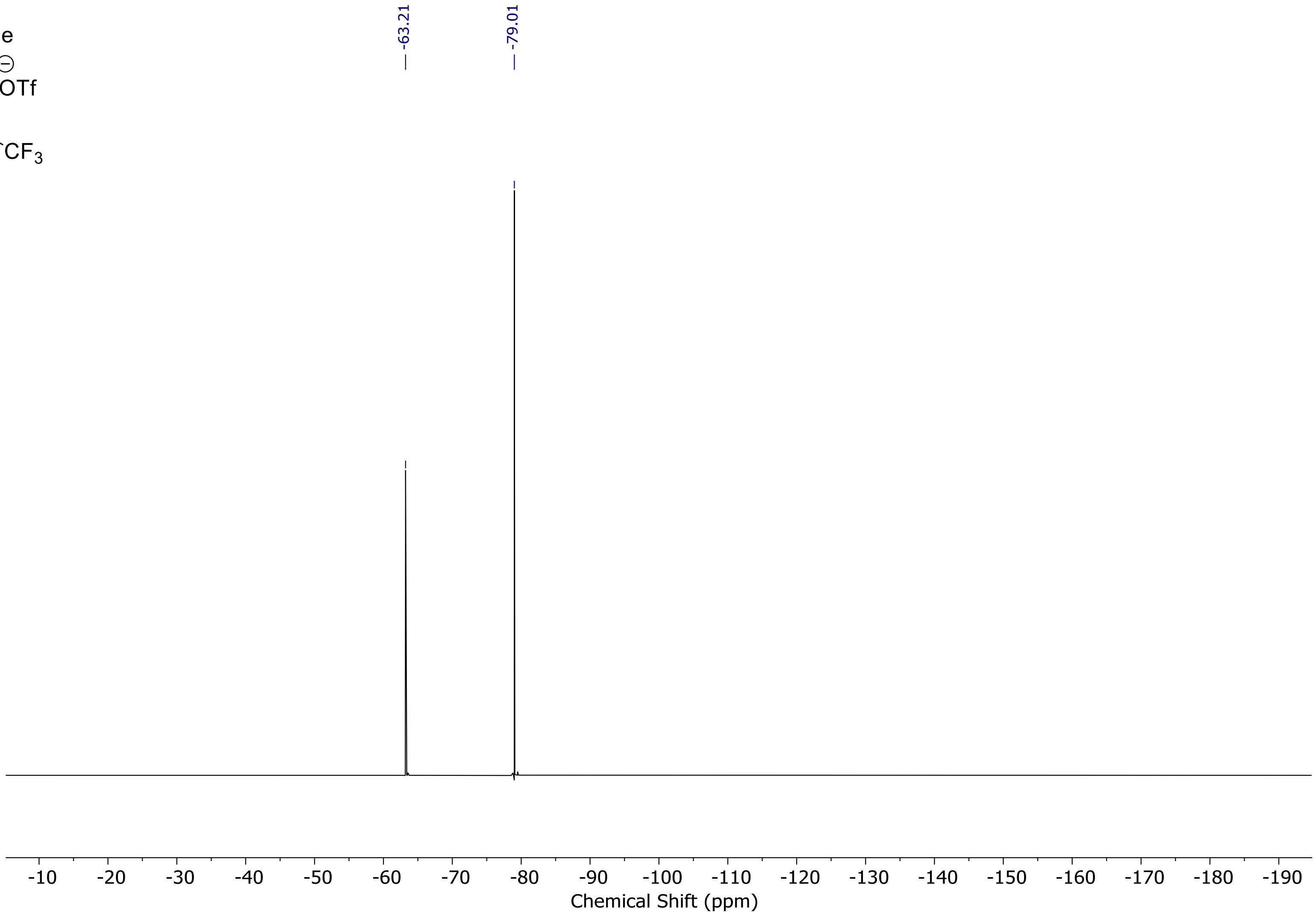

^{1}H NMR (400 MHz, $CDCl_3$) of 2-(2-fluoro-6-methylphenyl)pyrimidine 17a

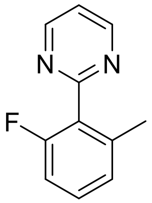

17a

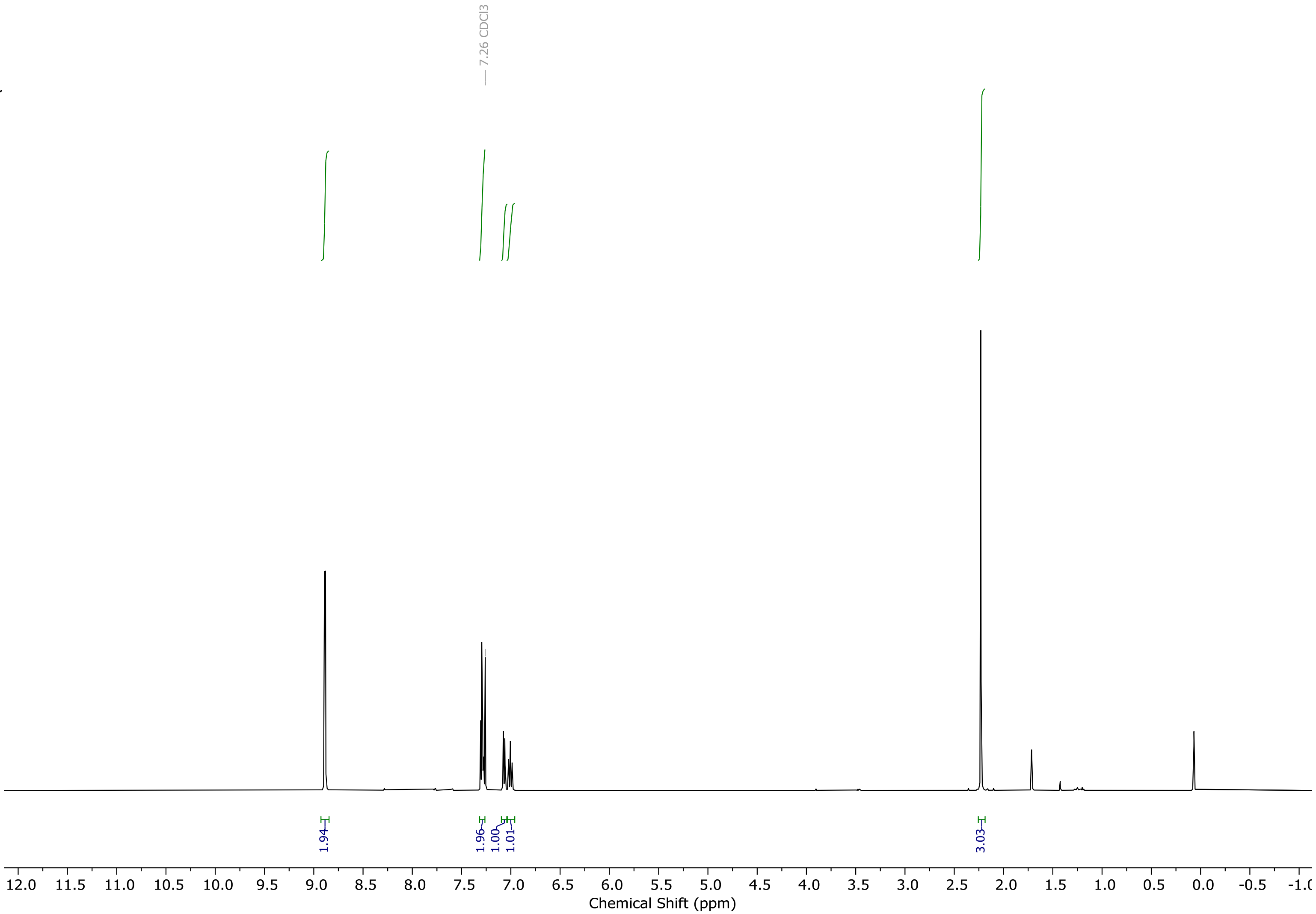

^{13}C NMR (126 MHz, $CDCl_3$) of 2-(2-fluoro-6-methylphenyl)pyrimidine 17a

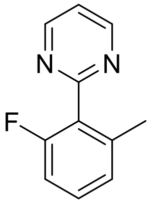

17a

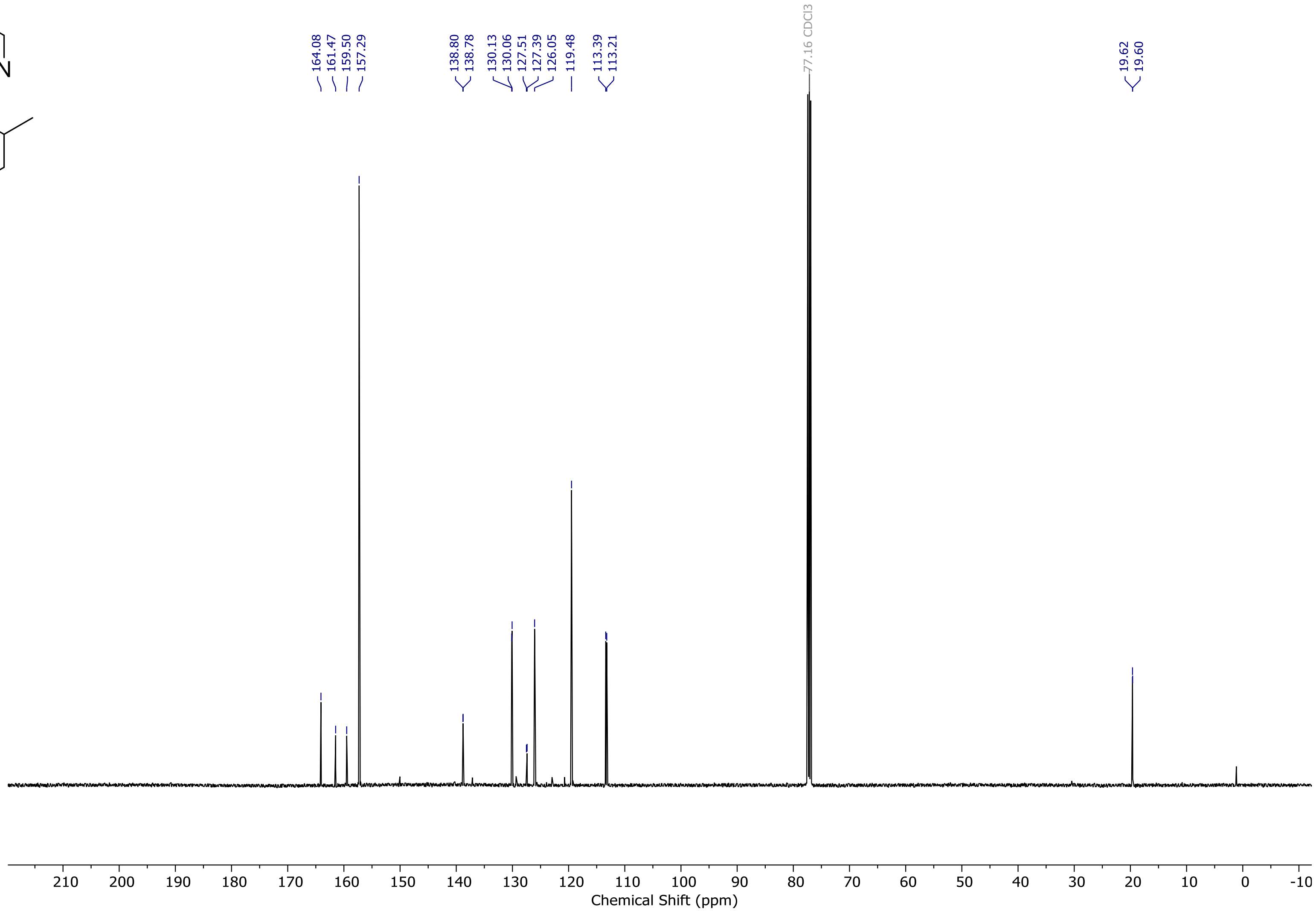

^{19}F NMR (376 MHz, $CDCl_3$) of 2-(2-fluoro-6-methylphenyl)pyrimidine 17a

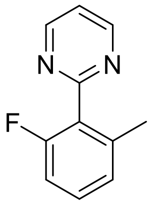

17a

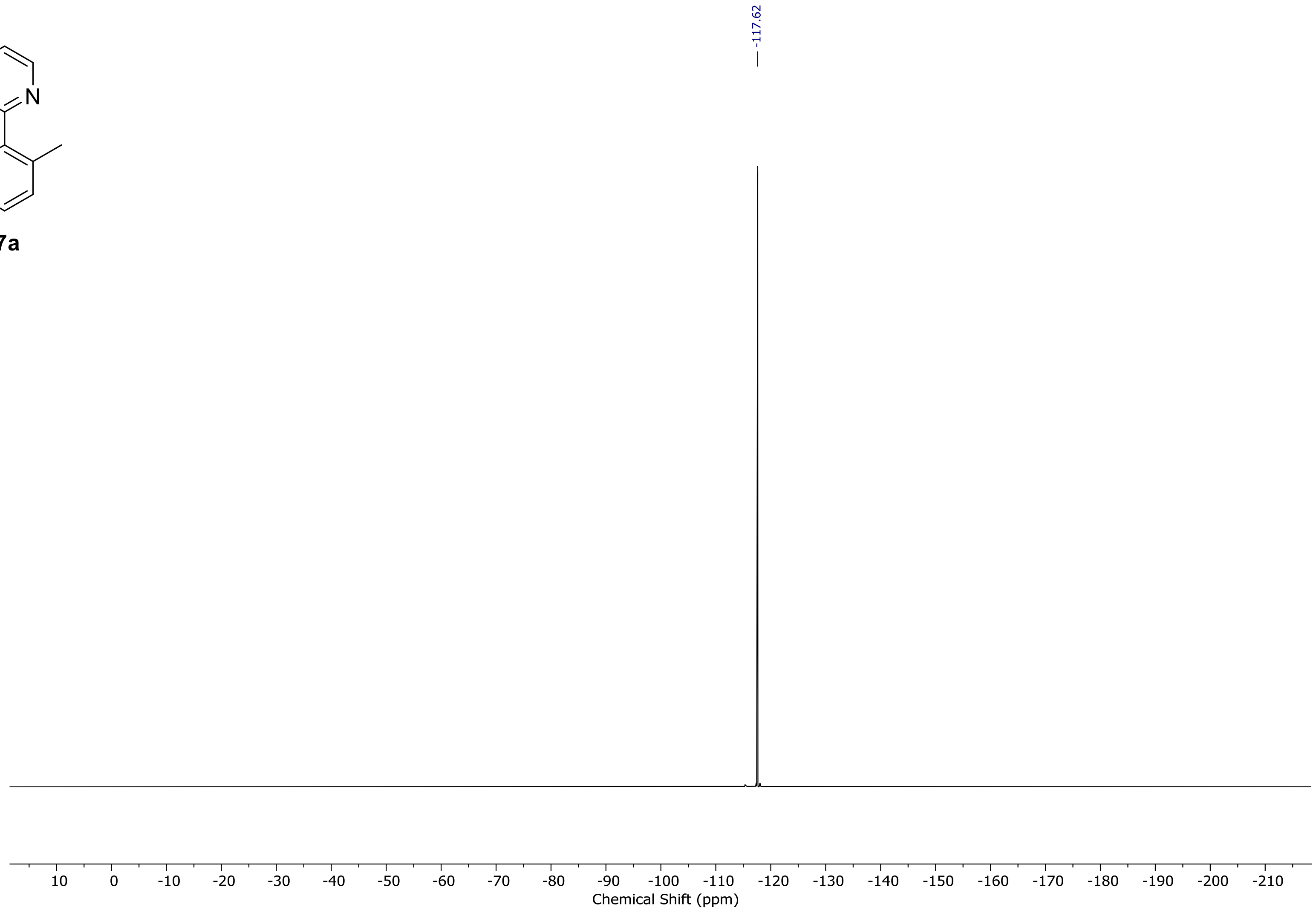

^{1}H NMR (400 MHz, $CDCl_3$) of 2-(2-methyl-5-(trifluoromethyl)phenyl)pyridine 17b

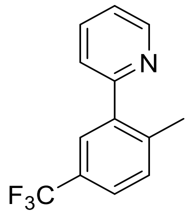

17b

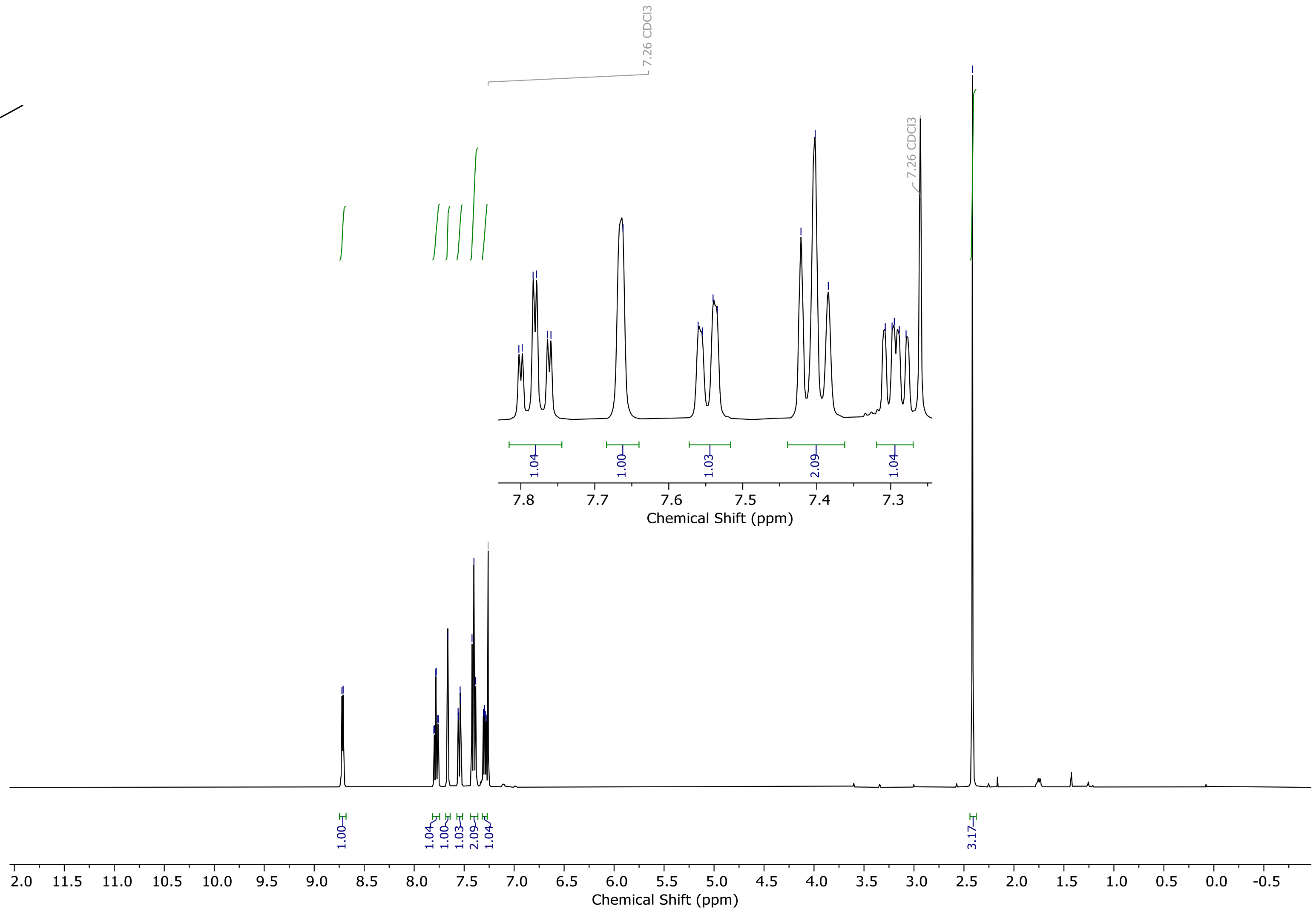

^{13}C NMR (101 MHz, $CDCl_3$) of 2-(2-methyl-5-(trifluoromethyl)phenyl)pyridine 17b

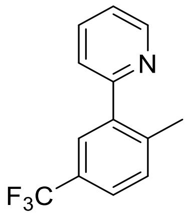

17b

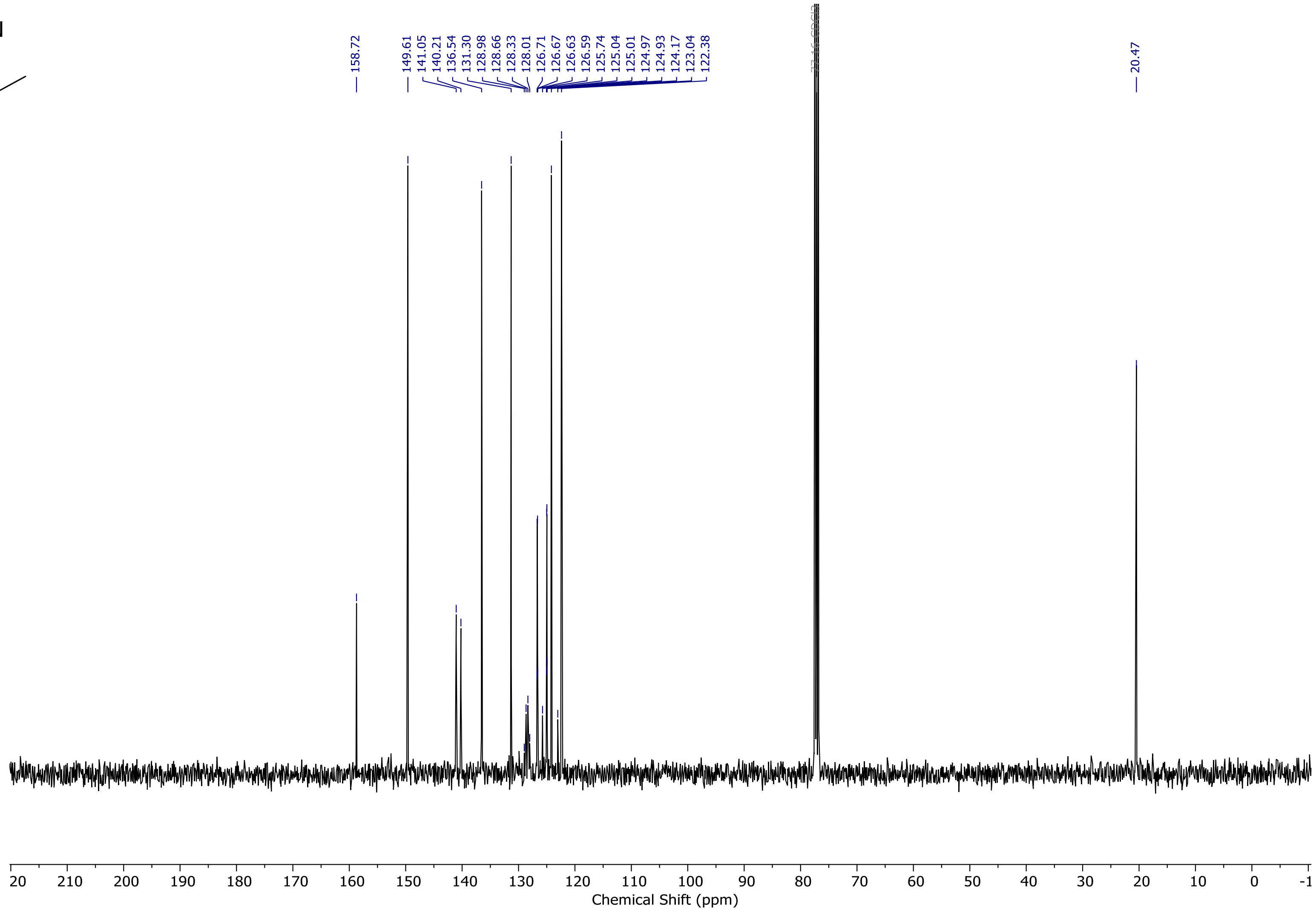

^{19}F NMR (376 MHz, $CDCl_3$) of 2-(2-methyl-5-(trifluoromethyl)phenyl)pyridine 17b

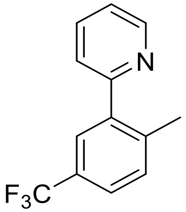

17b

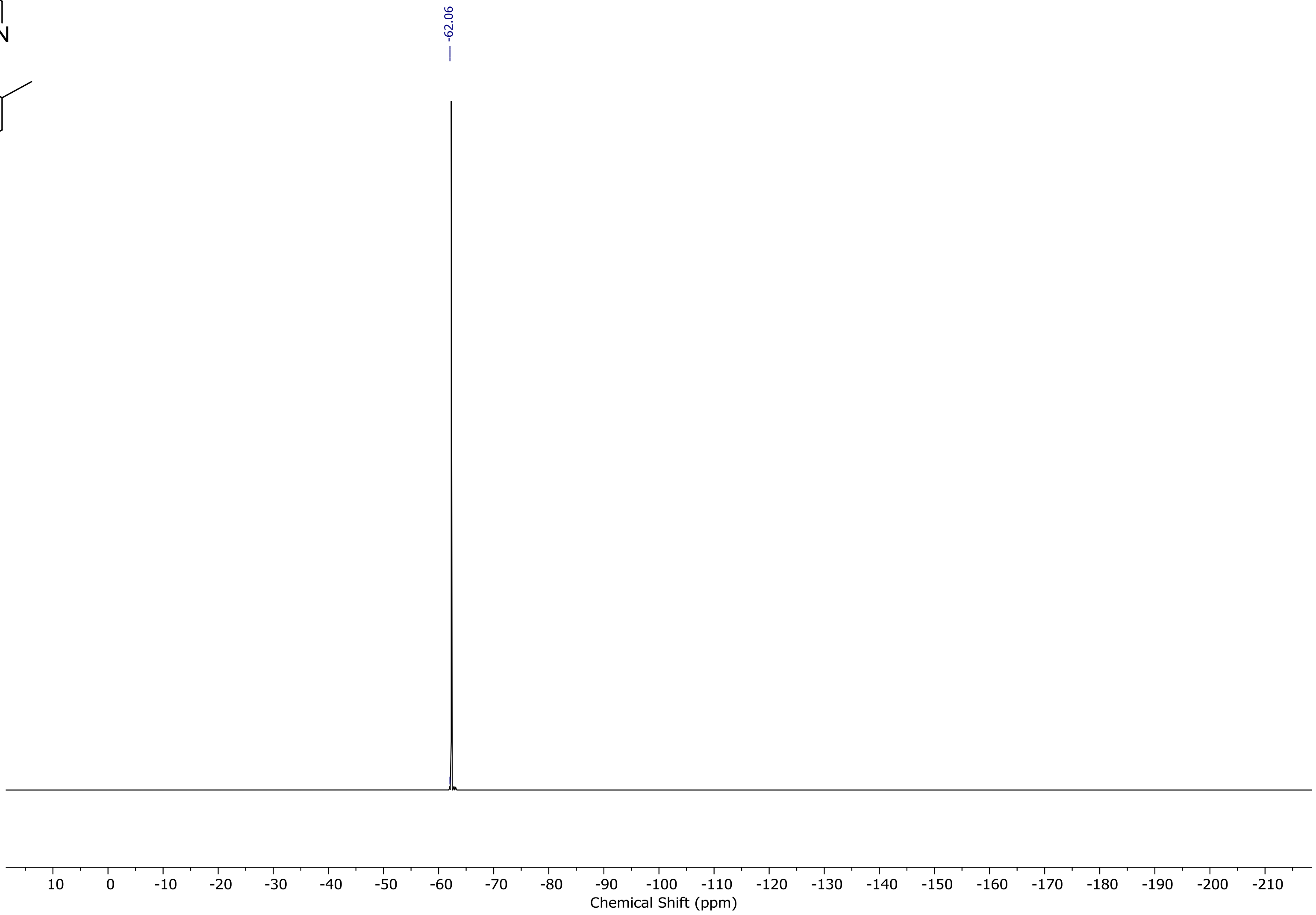

^{1}H NMR (400 MHz, $CDCl_3$) of 1-(*o*-tolyl)isoquinoline 17c

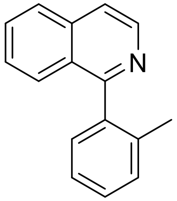

17c

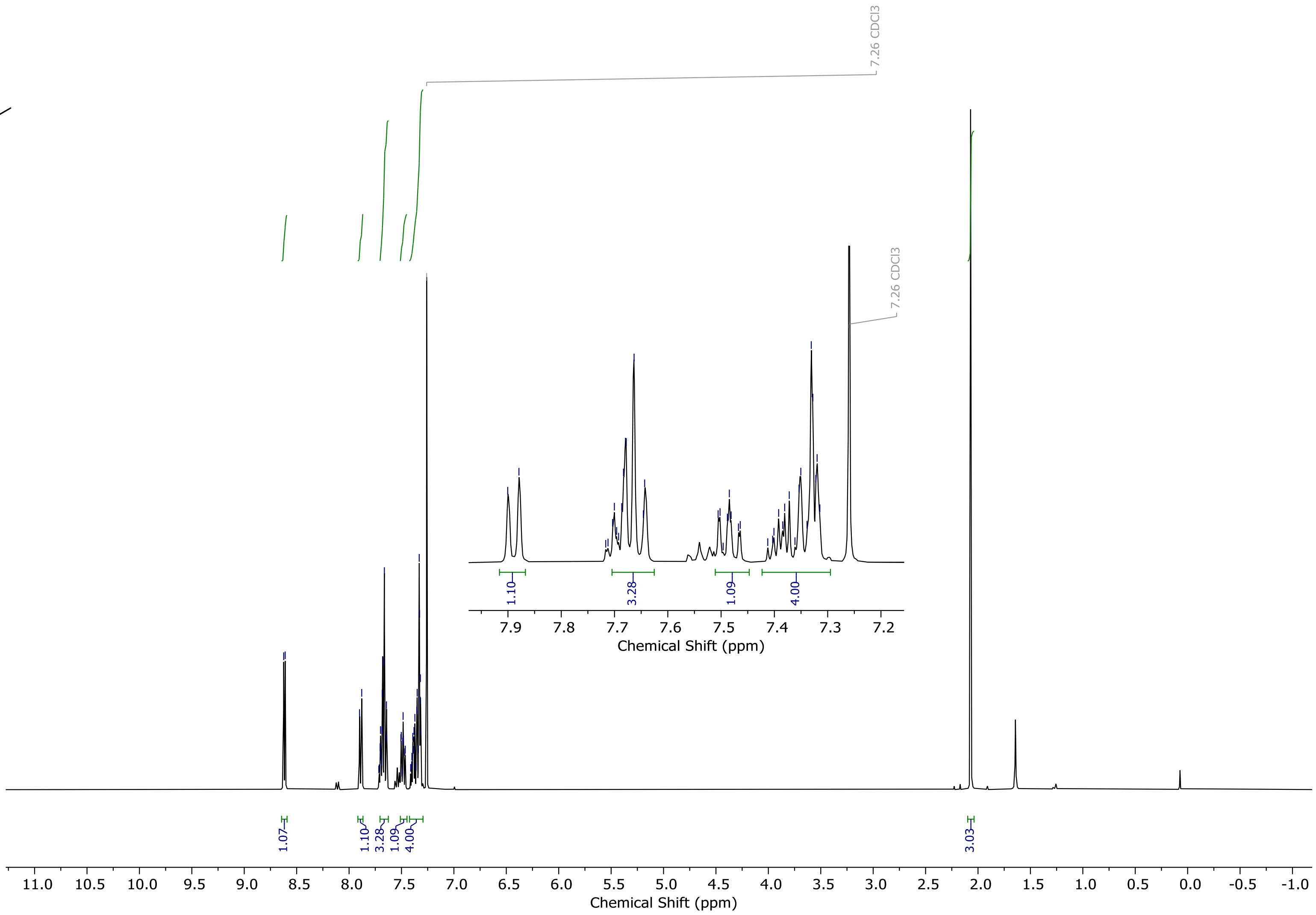

^{13}C NMR (101 MHz, $CDCl_3$) of 1-(*o*-tolyl)isoquinoline 17c

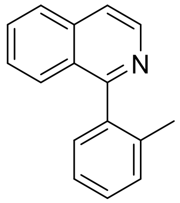

17c

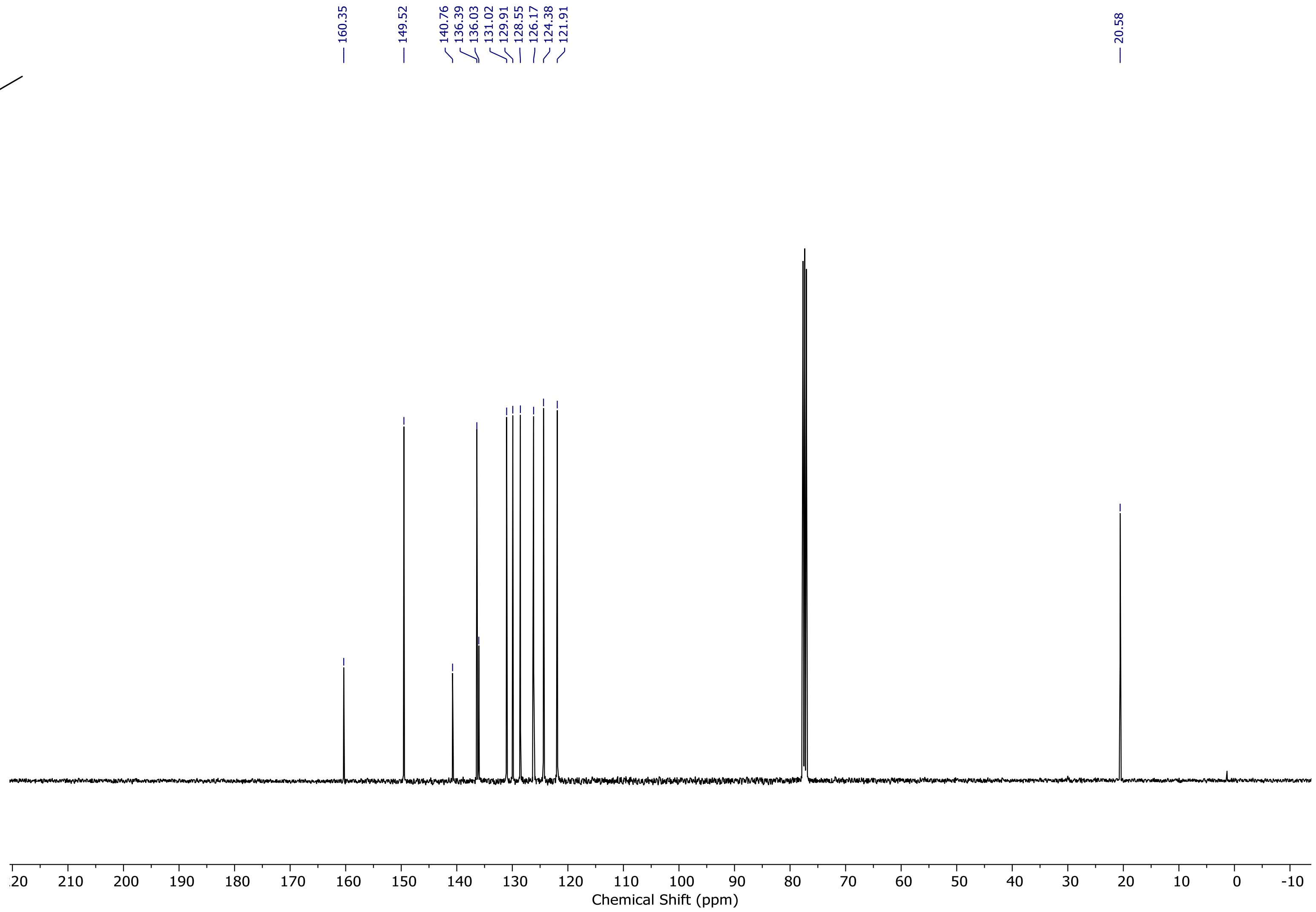

^{1}H NMR (400 MHz, $CDCl_3$) of 7-chloro-1-methyl-5-(o-tolyl)-1,3-dihydro-2H-benzo[e][1,4]diazepin-2-one 17d from diazepam

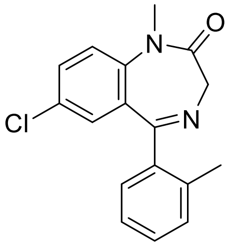

17d

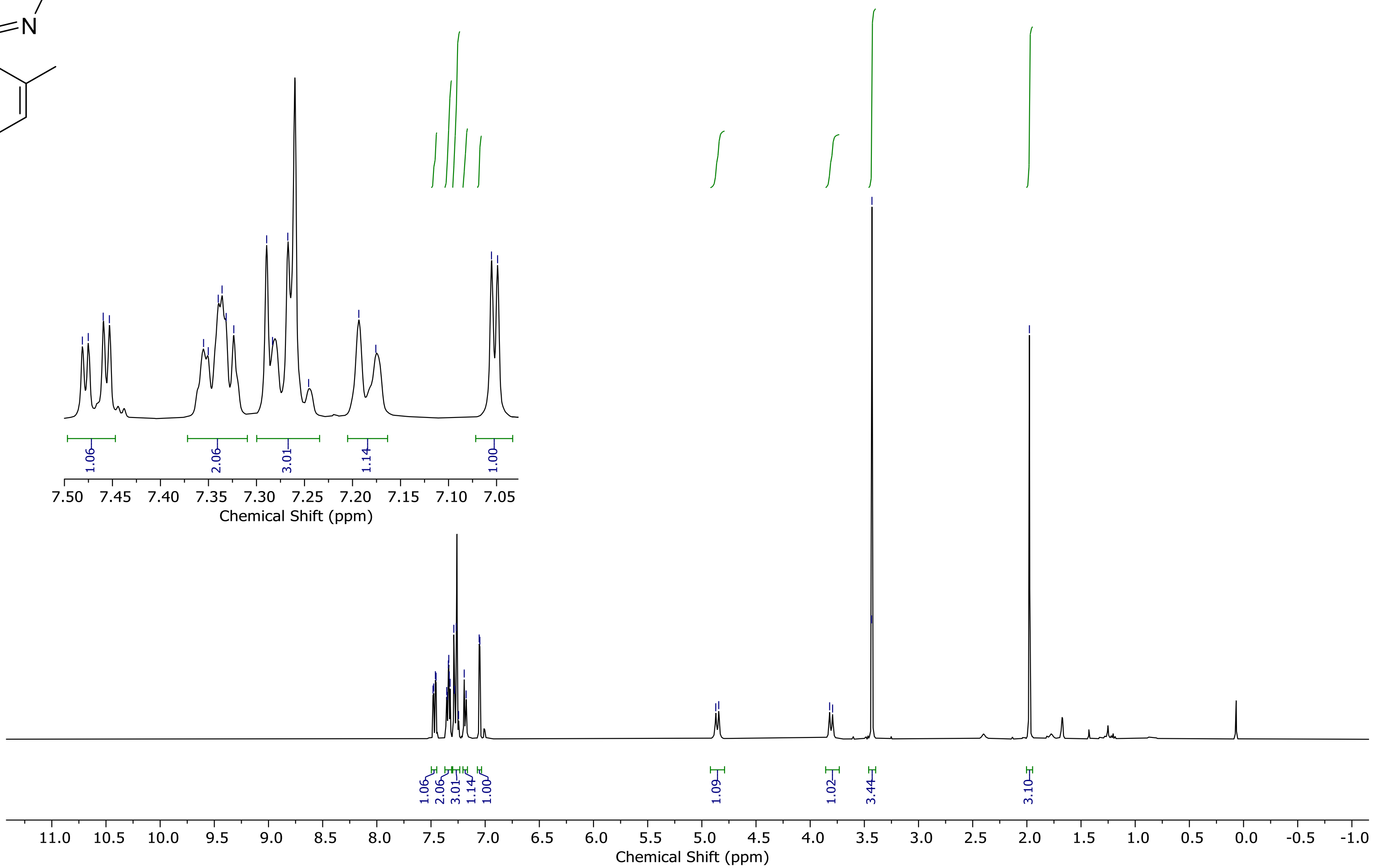

^{13}C NMR (101 MHz, CDCl$_3$) of 7-chloro-1-methyl-5-(o-tolyl)-1,3-dihydro-2H-benzo[e][1,4]diazepin-2-one 17d from diazepam

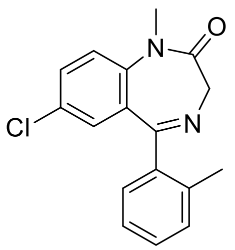

17d

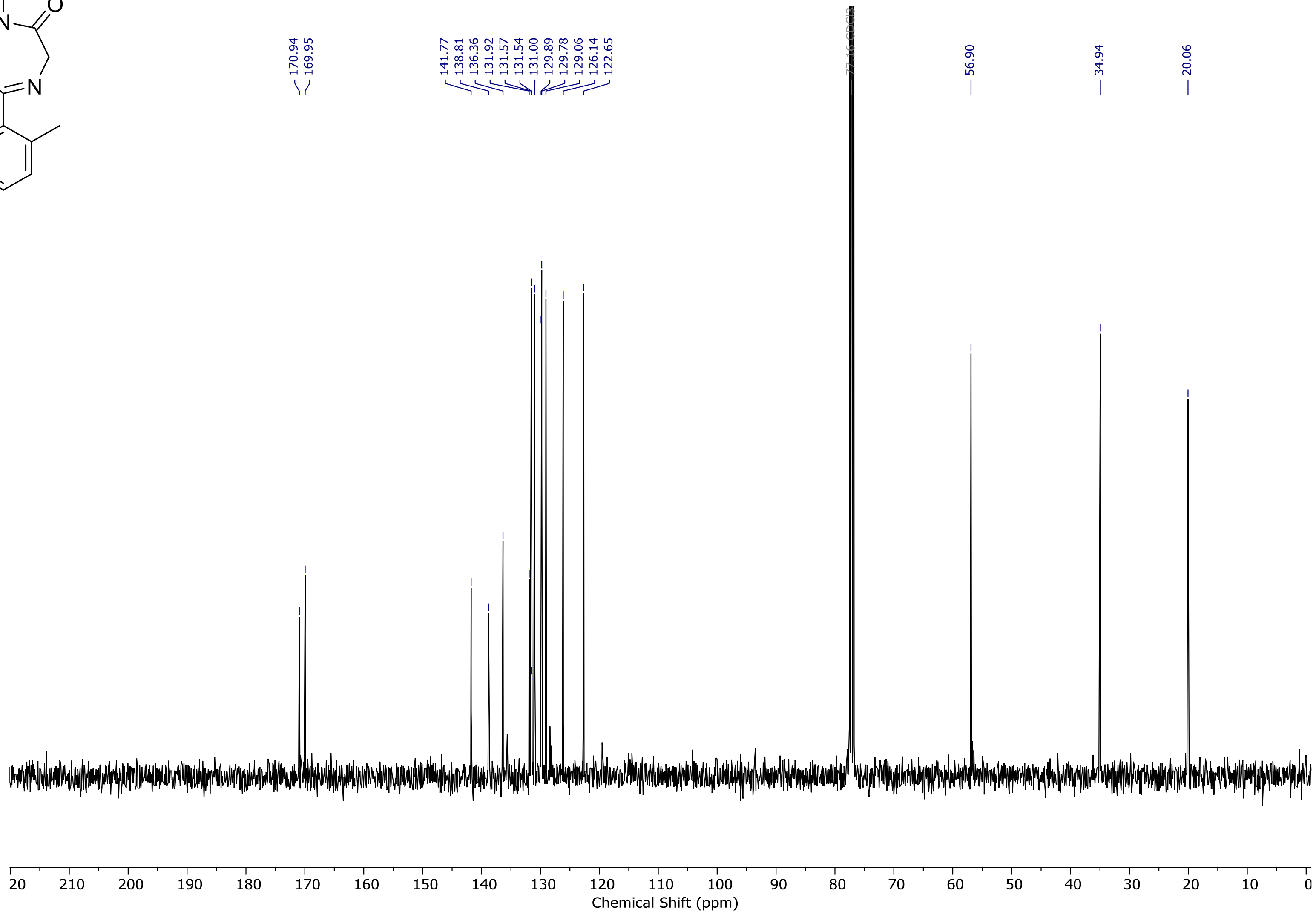

^{1}H NMR (400 MHz, CDCl$_3$) of (8*R*,9*S*,13*S*,14*S*)-13-methyl-17-oxo-7,8,9,11,12,13,14,15,16,17-decahydro-6H-cyclopenta[a]phenanthren-3-yl 4-methyl-3-(pyridin-2-yl)benzoate 17e from estrone derivative

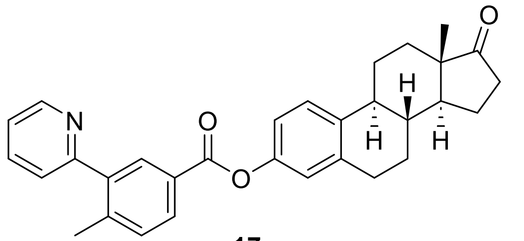

17e

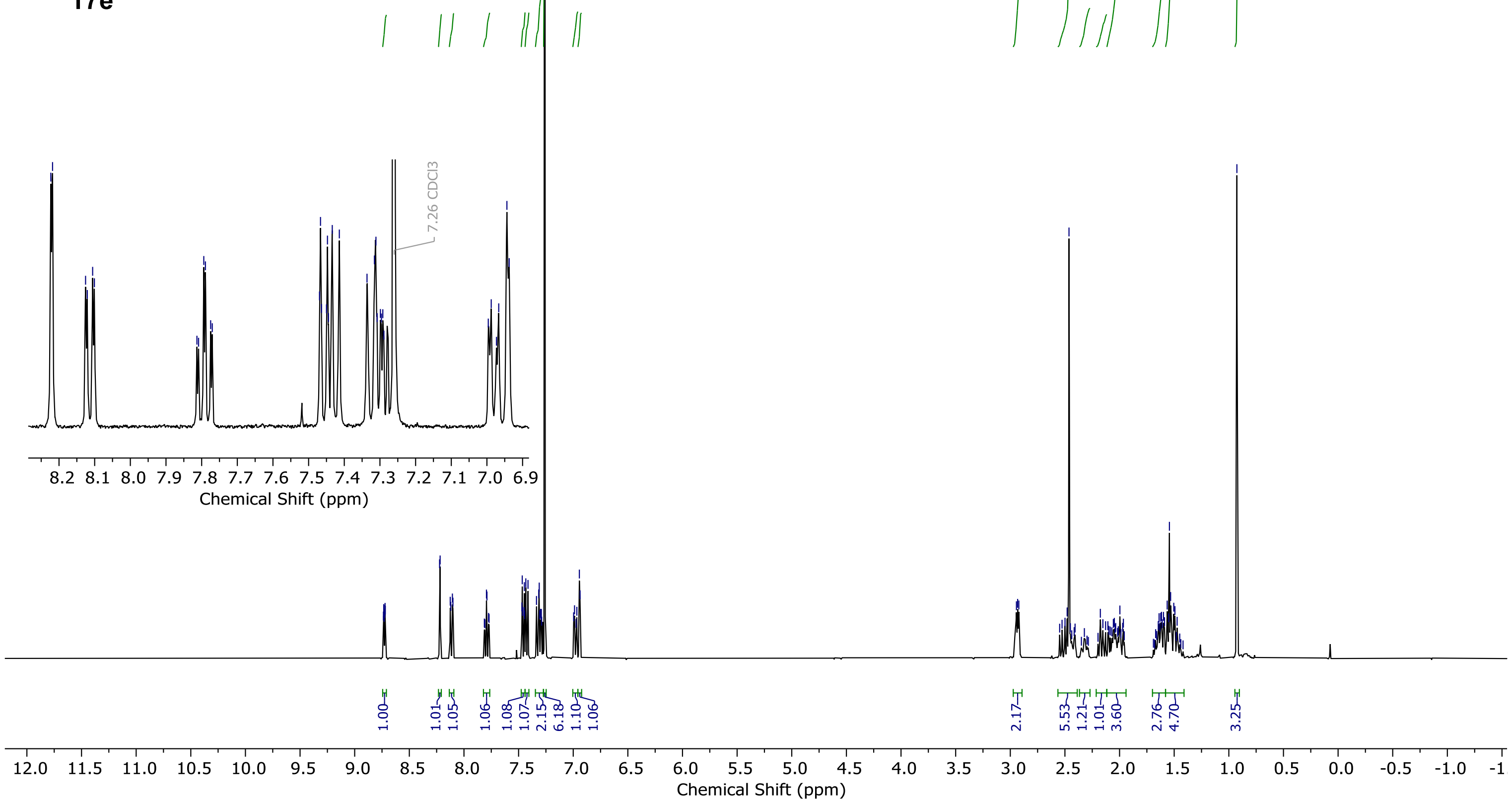

^{13}C NMR (101 MHz, $CDCl_3$) of (8R,9S,13S,14S)-13-methyl-17-oxo-7,8,9,11,12,13,14,15,16,17-decahydro-6H-cyclopenta[a]phenanthren-3-yl 4-methyl-3-(pyridin-2-yl)benzoate 17e from estrone derivative

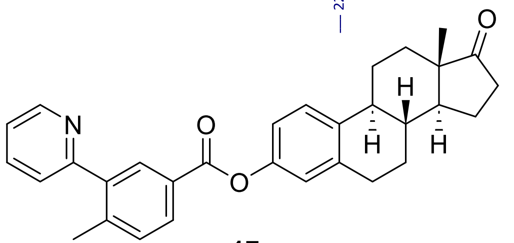

17e

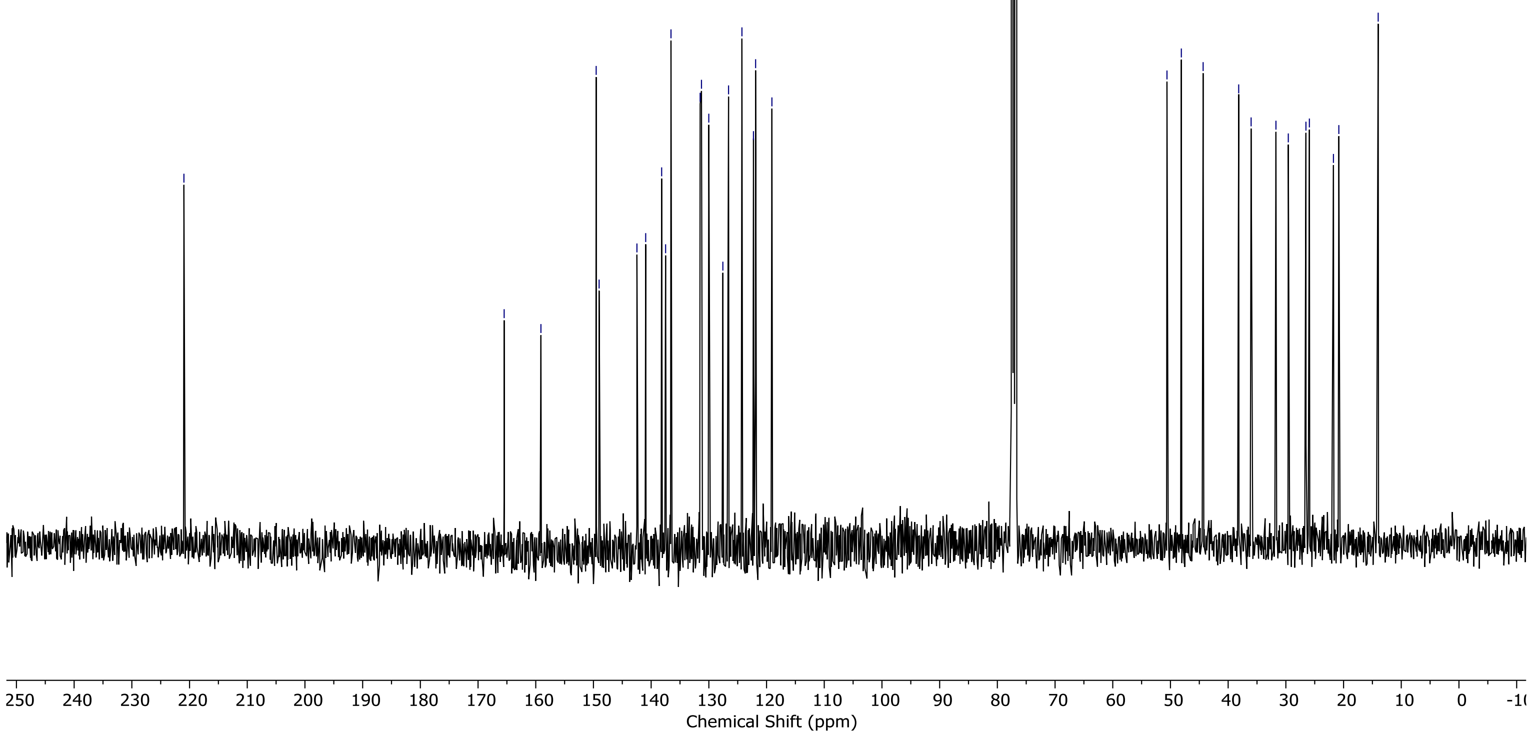

^{1}H NMR (400 MHz, $CDCl_3$) of 2-(*o*-tolyl)pyridine 17f

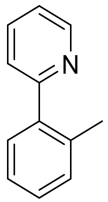

17f

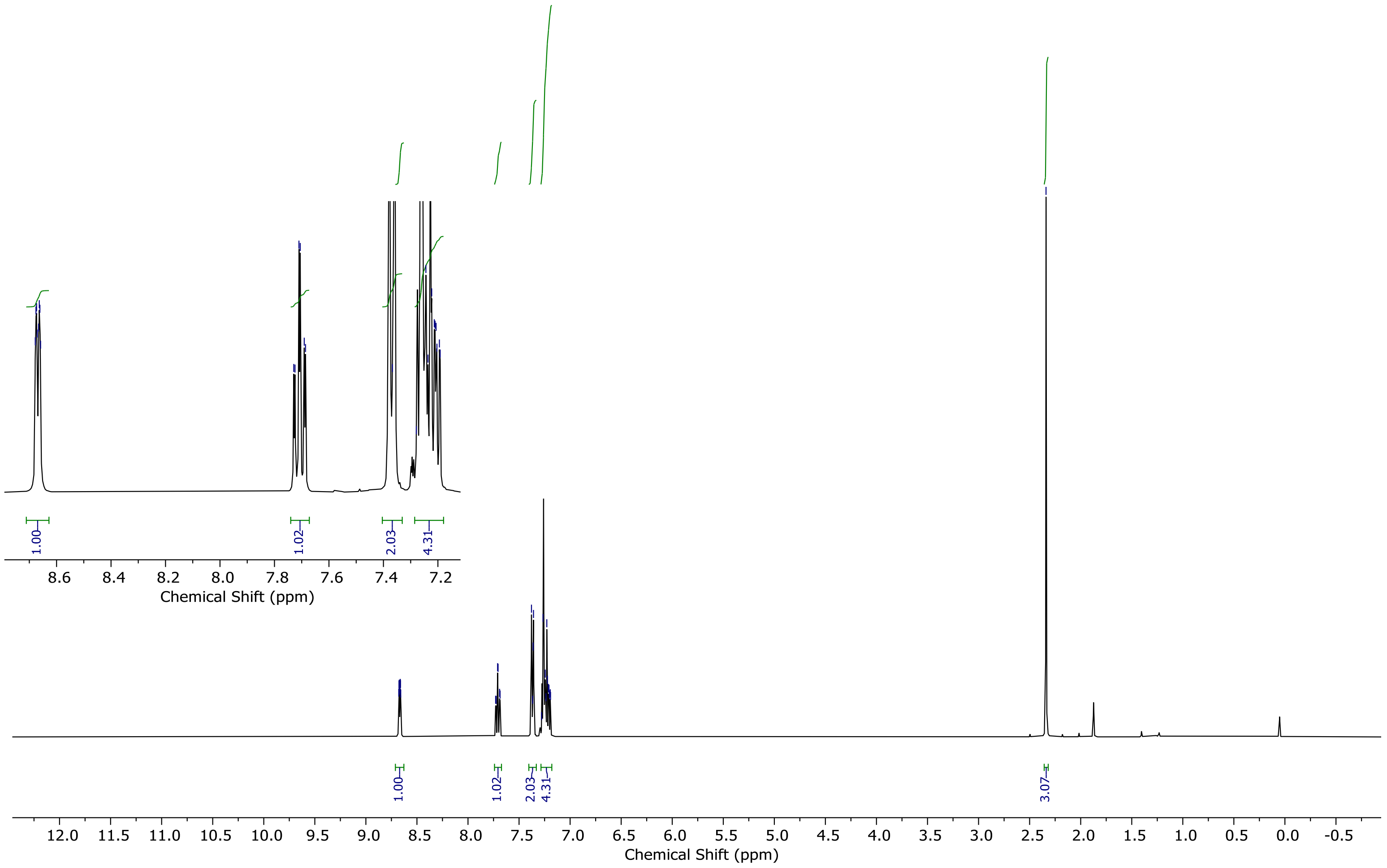

^{13}C NMR (101 MHz, $CDCl_3$) of 2-(*o*-tolyl)pyridine 17f

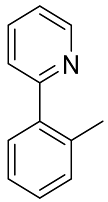

17f

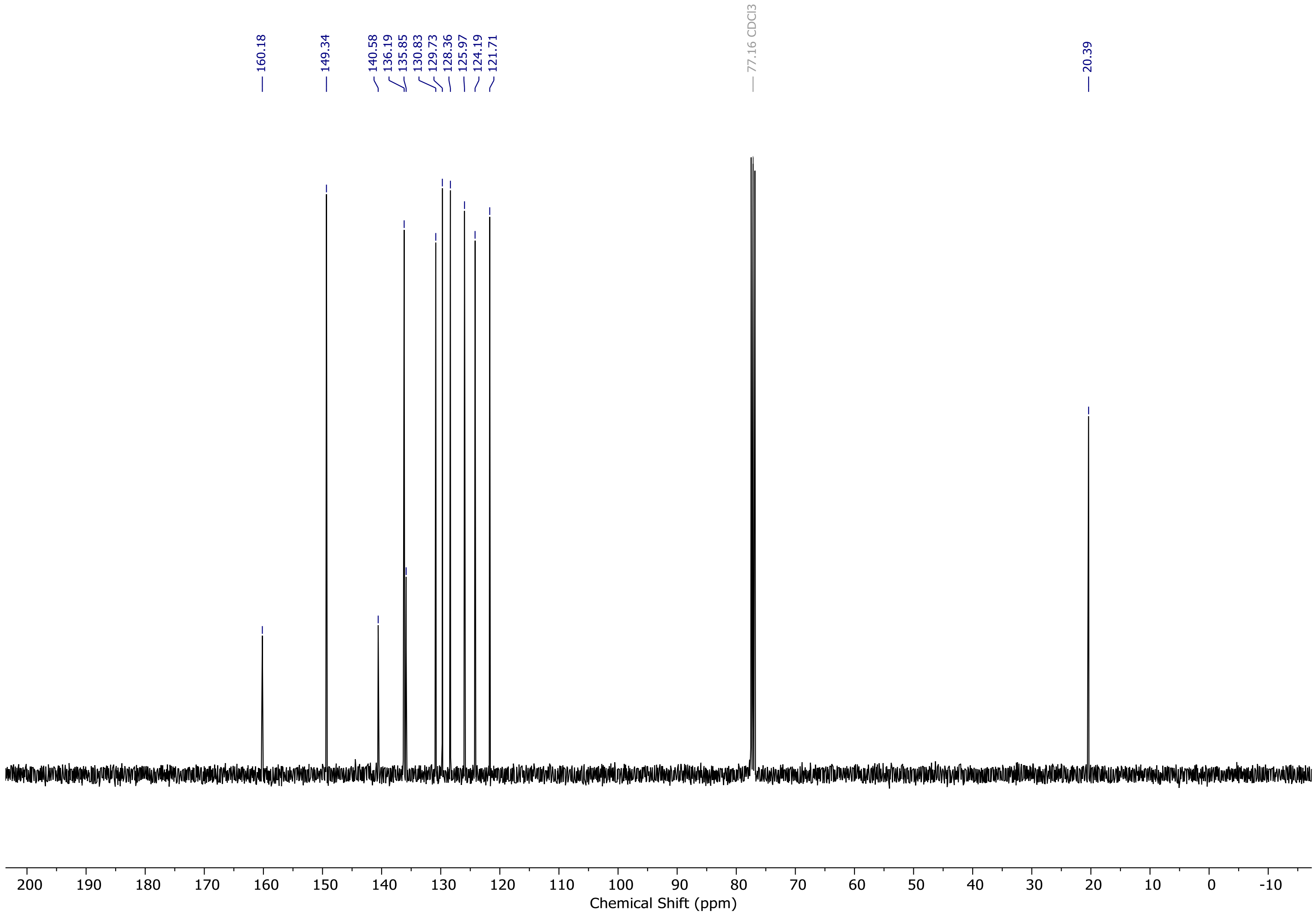

^{1}H NMR (400 MHz, $CDCl_3$) of 2-(2,6-dimethylphenyl)pyridine 17f'

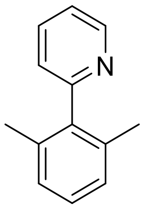

17f'

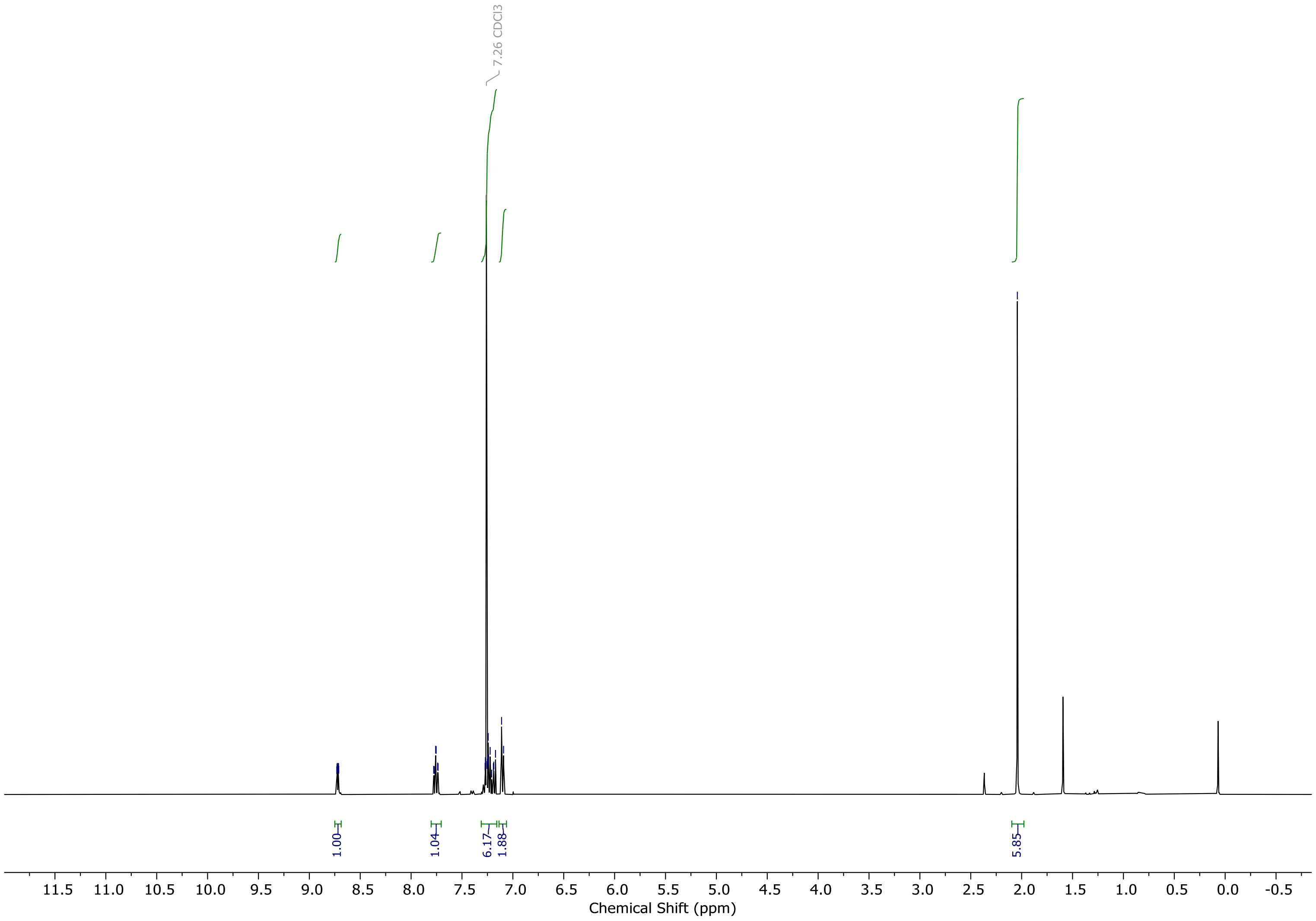

^{13}C NMR (101 MHz, $CDCl_3$) of 2-(2,6-dimethylphenyl)pyridine 17f'

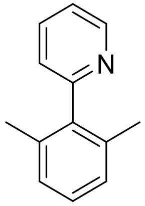

17f'

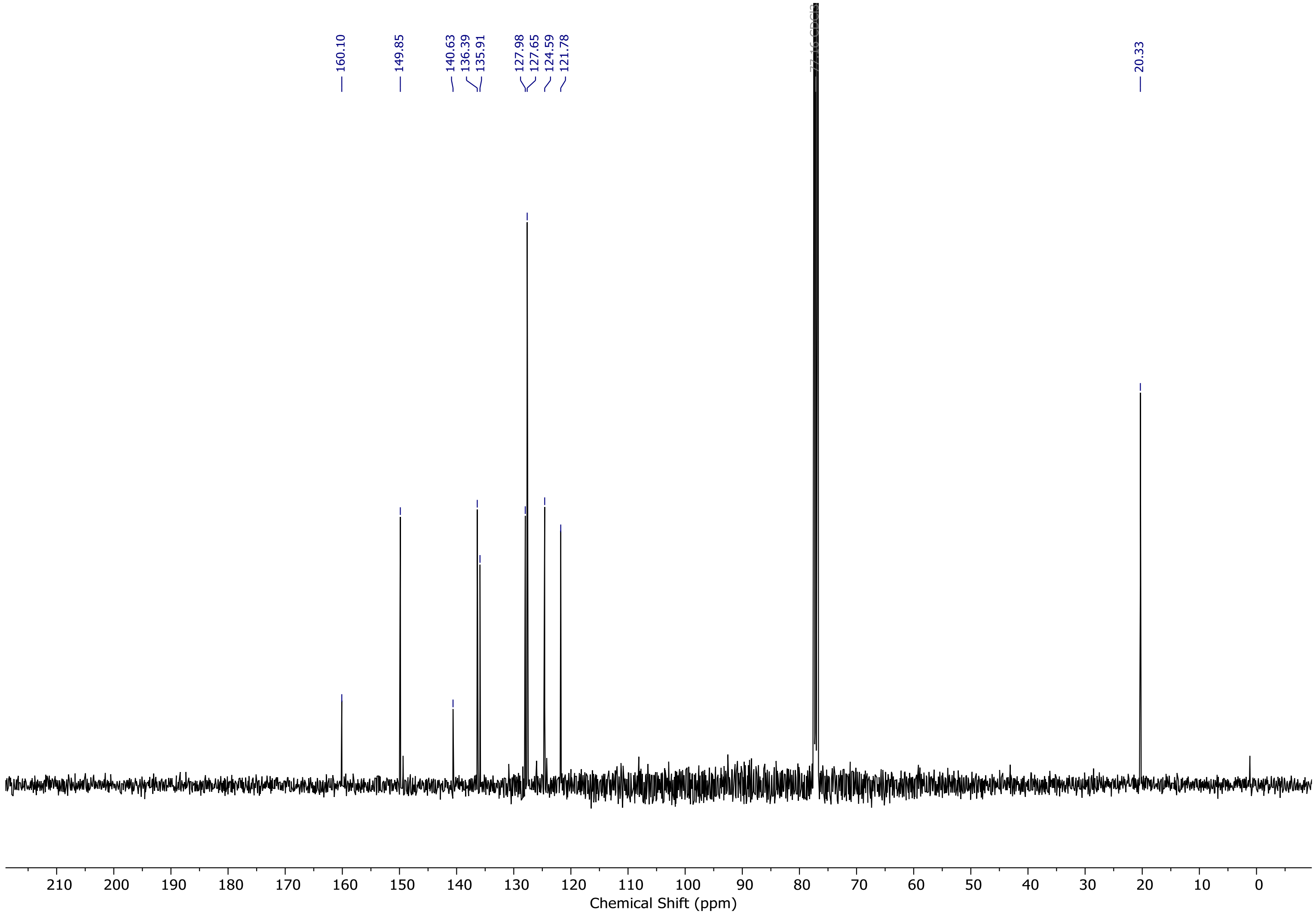

^{1}H NMR (400 MHz, CDCl₃) of 2-(2-fluoro-6-methylphenyl)pyridine 17g

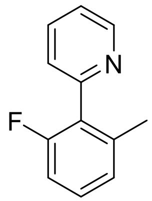

17g

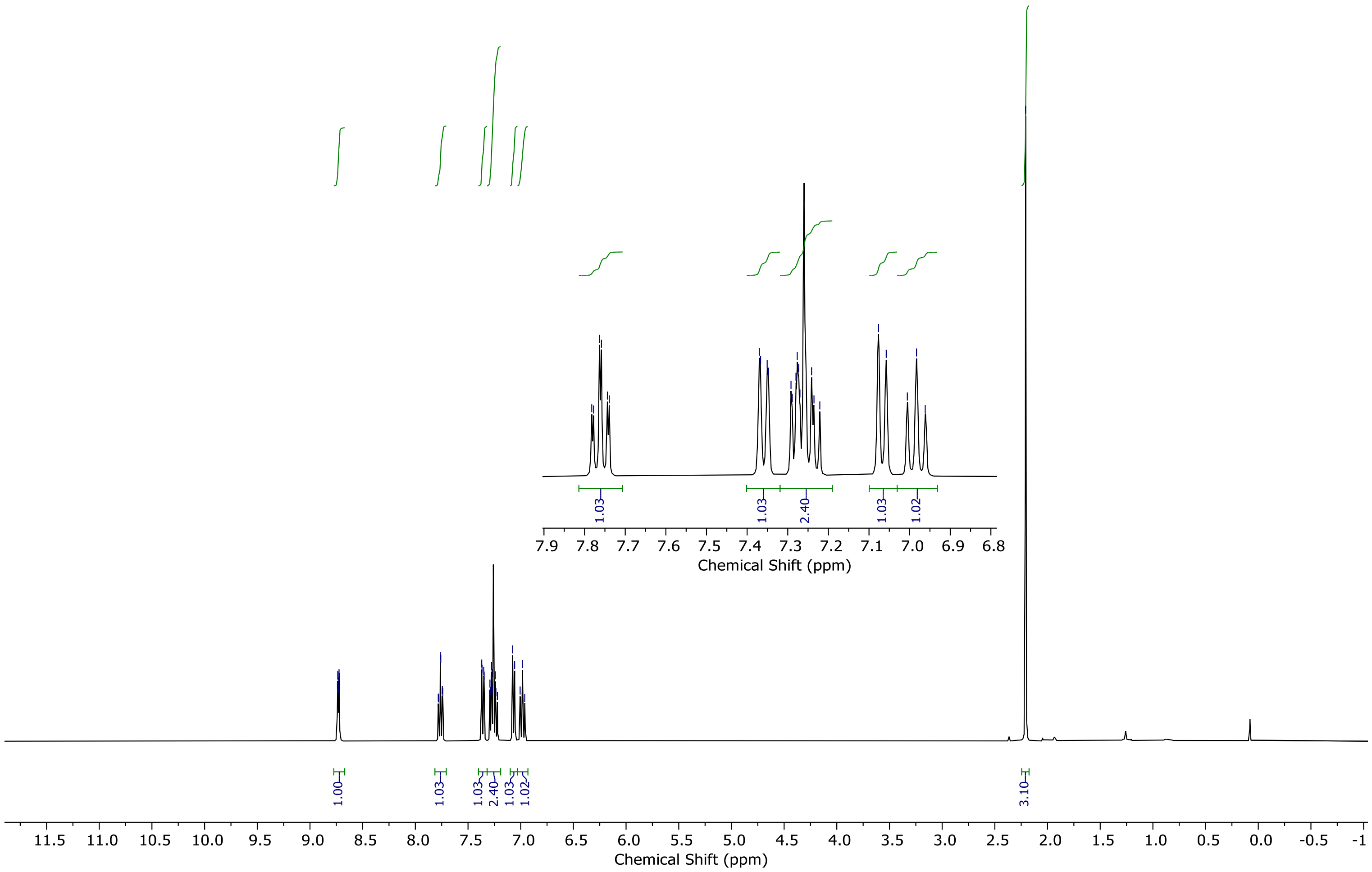

^{13}C NMR (101 MHz, CDCl3) of 2-(2-fluoro-6-methylphenyl)pyridine 17g

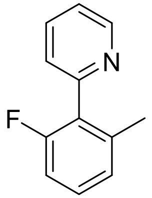
17g

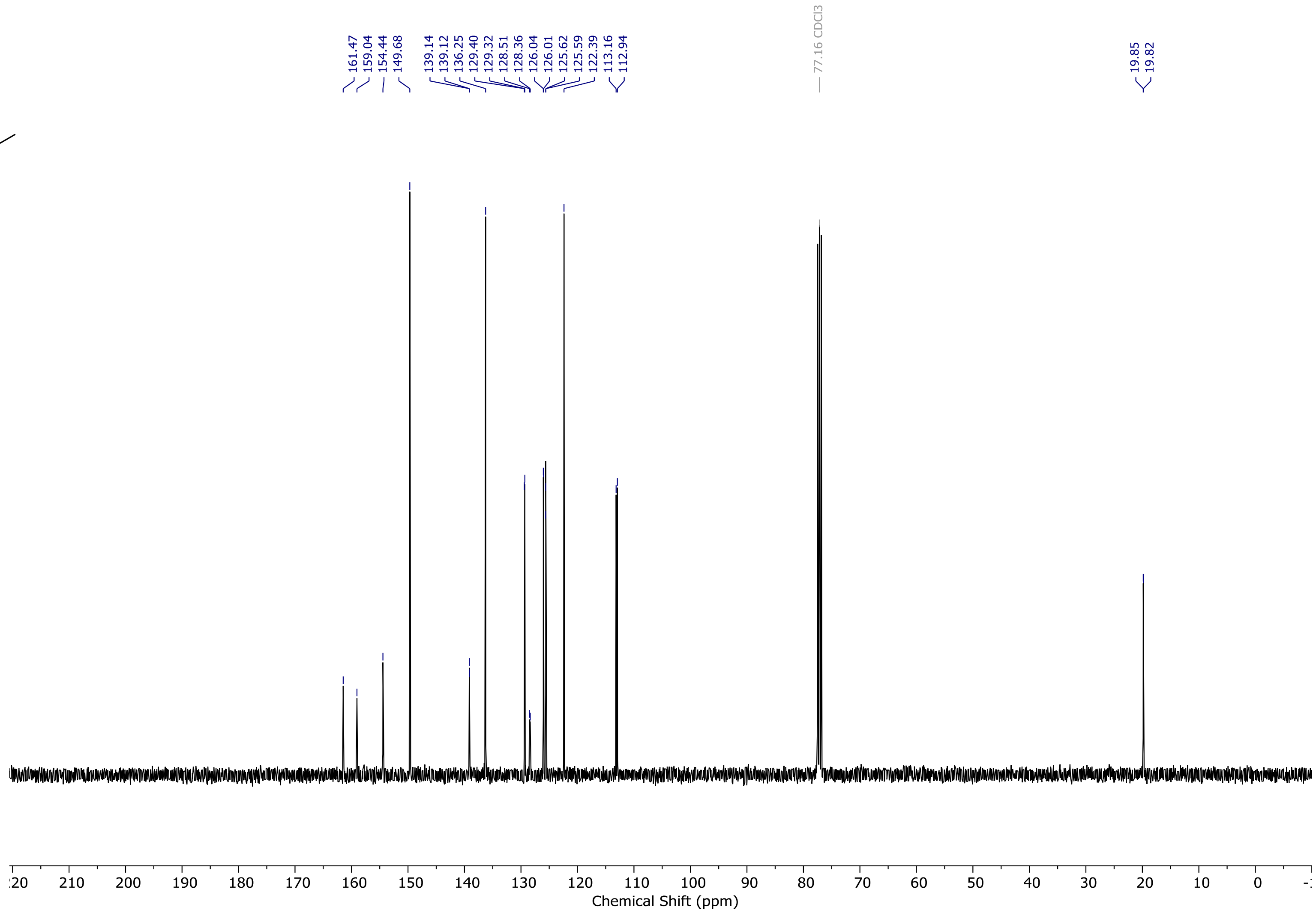

^{19}F NMR (376 MHz, $CDCl_3$) of 2-(2-fluoro-6-methylphenyl)pyridine 17g

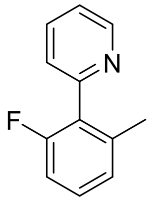

17g

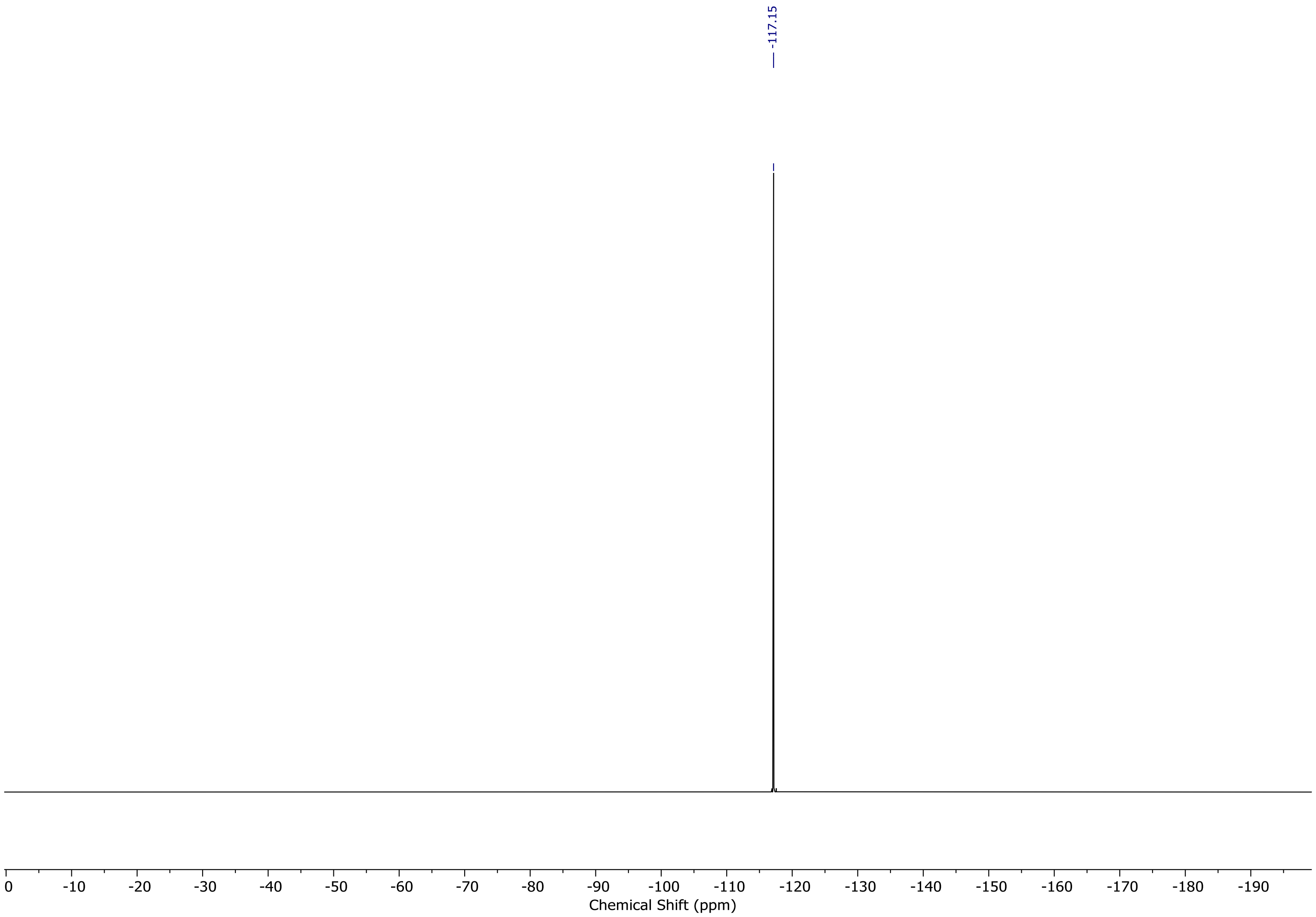

^{1}H NMR (400 MHz, CDCl₃) of 2-(4-(tert-butyl)-2-methylphenyl)pyridine 17h

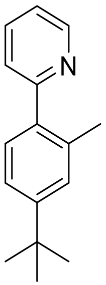

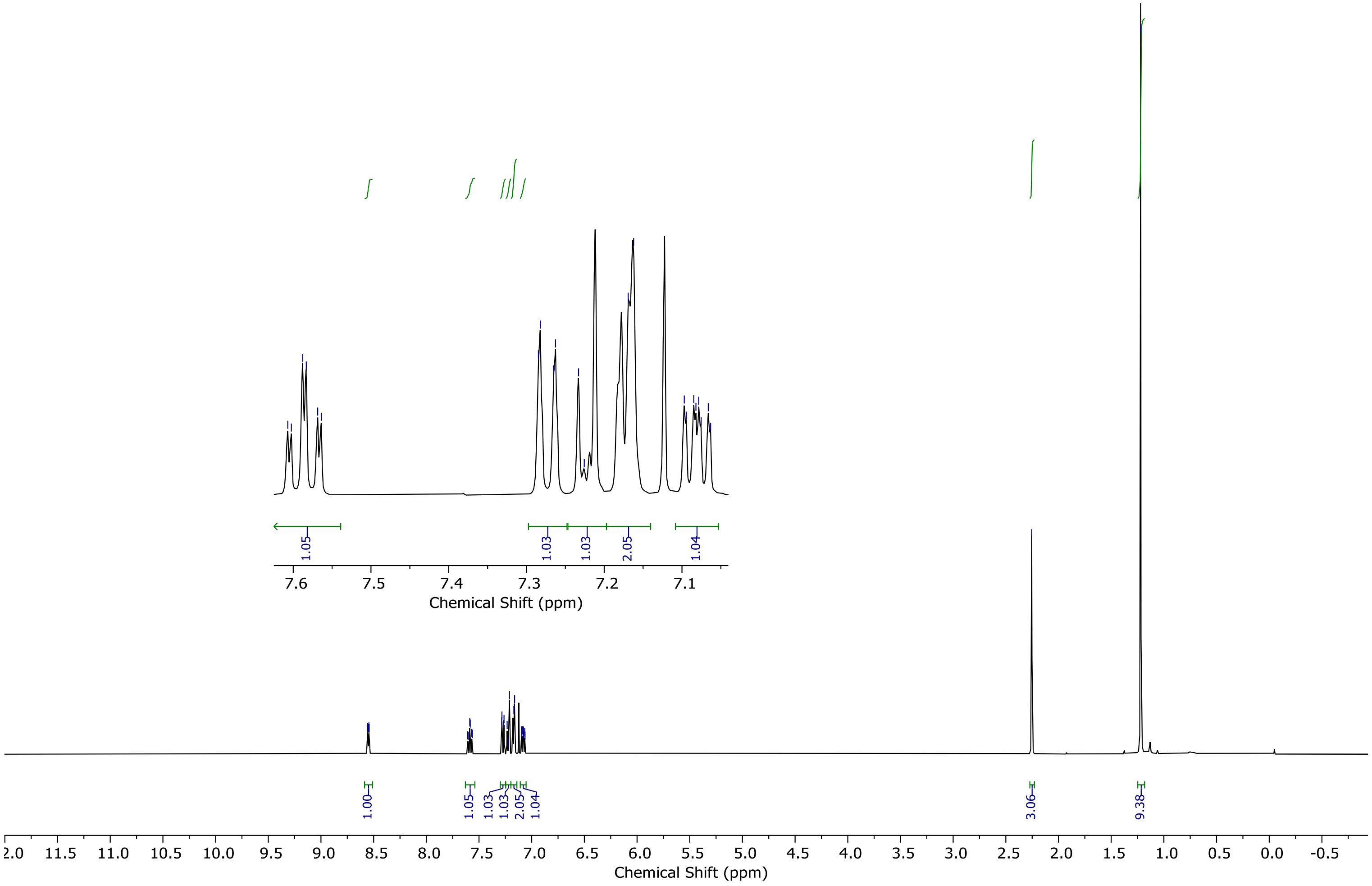

^{13}C NMR (101 MHz, $CDCl_3$) of 2-(4-(tert-butyl)-2-methylphenyl)pyridine 17h

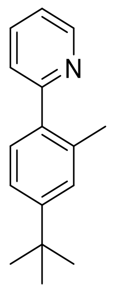

17h

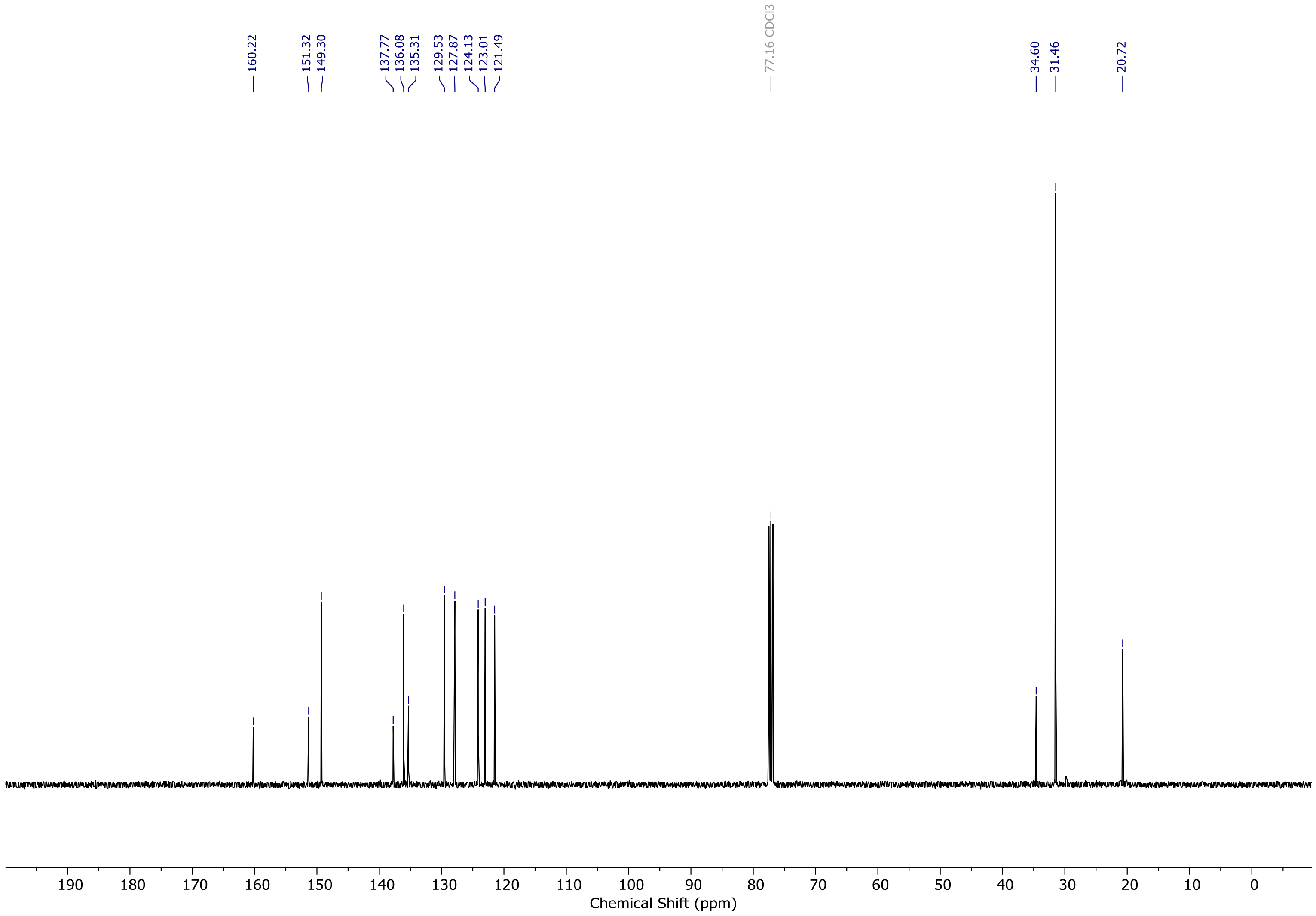

^{1}H NMR (400 MHz, CDCl$_3$) of 2-(4-(tert-butyl)-2,6-dimethylphenyl)pyridine 17h'

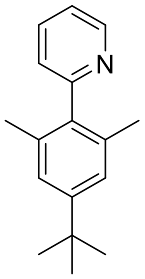

17h'

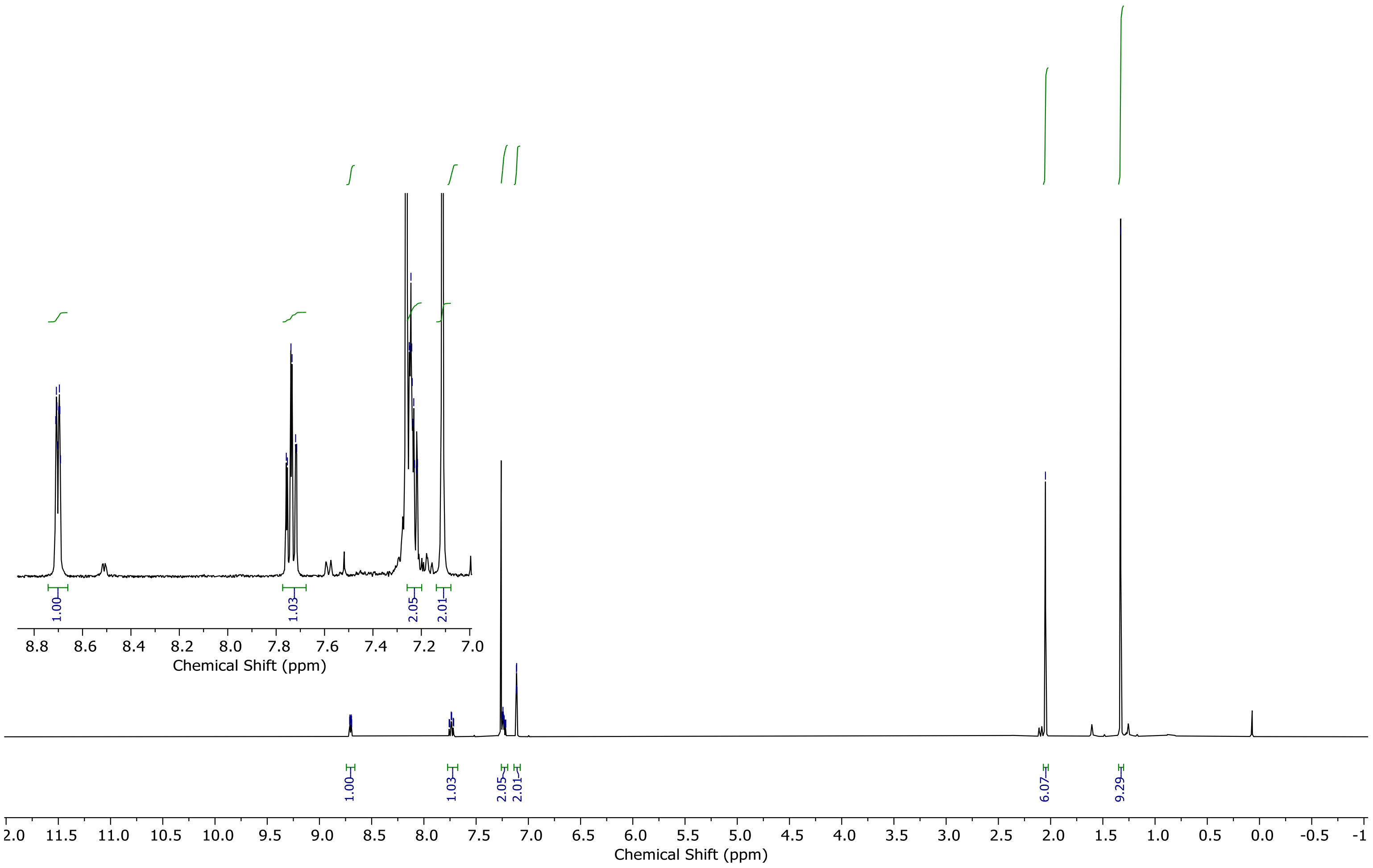

^{13}C NMR (101 MHz, CDCl₃) of 2-(4-(tert-butyl)-2,6-dimethylphenyl)pyridine 17h’

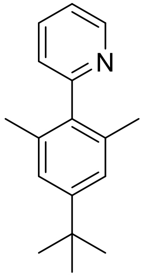

17h'

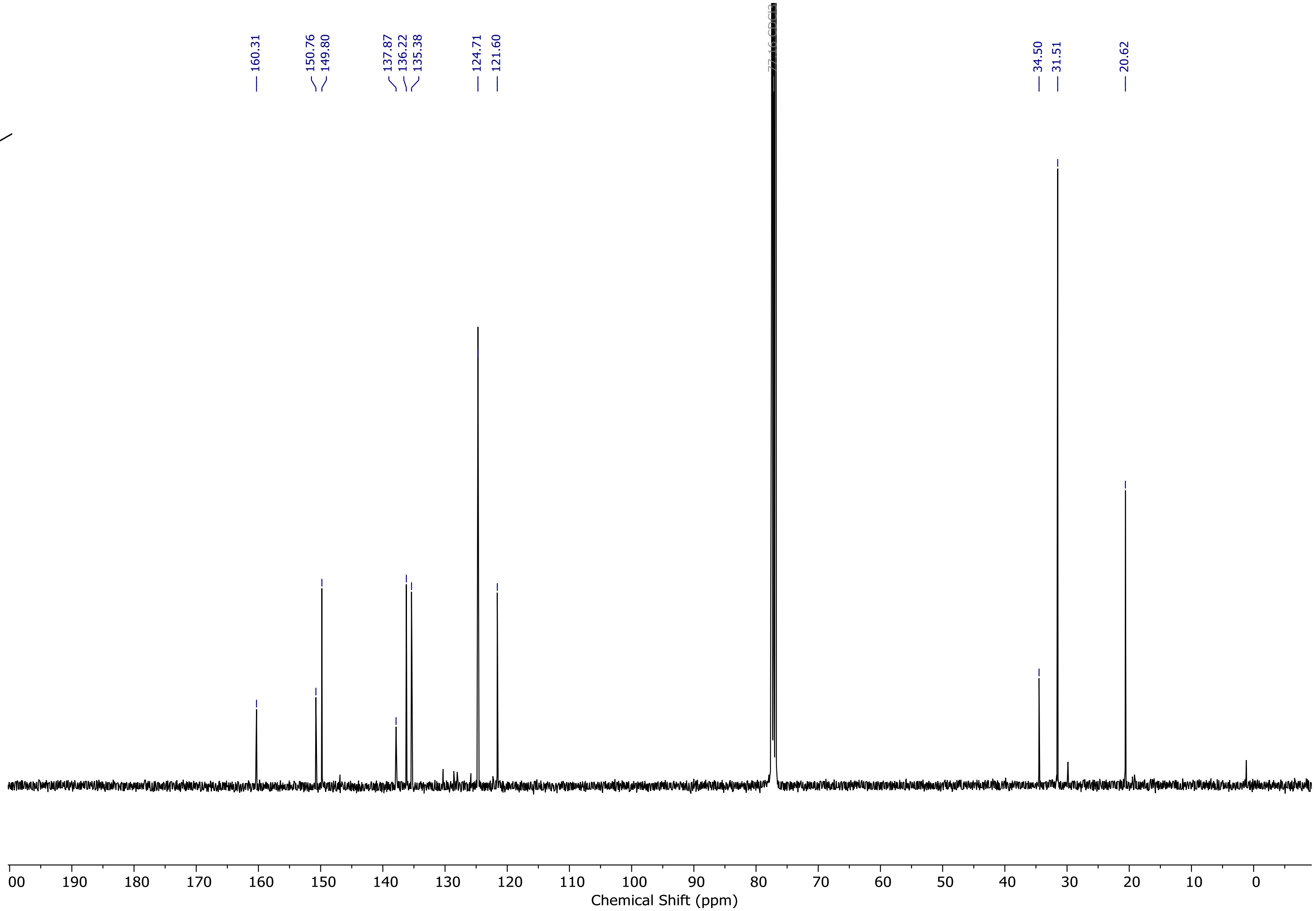

^{1}H NMR (400 MHz, $CDCl_3$) of 3-methyl-2-(*o*-tolyl)pyridine **17i**

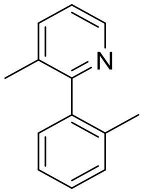

17i

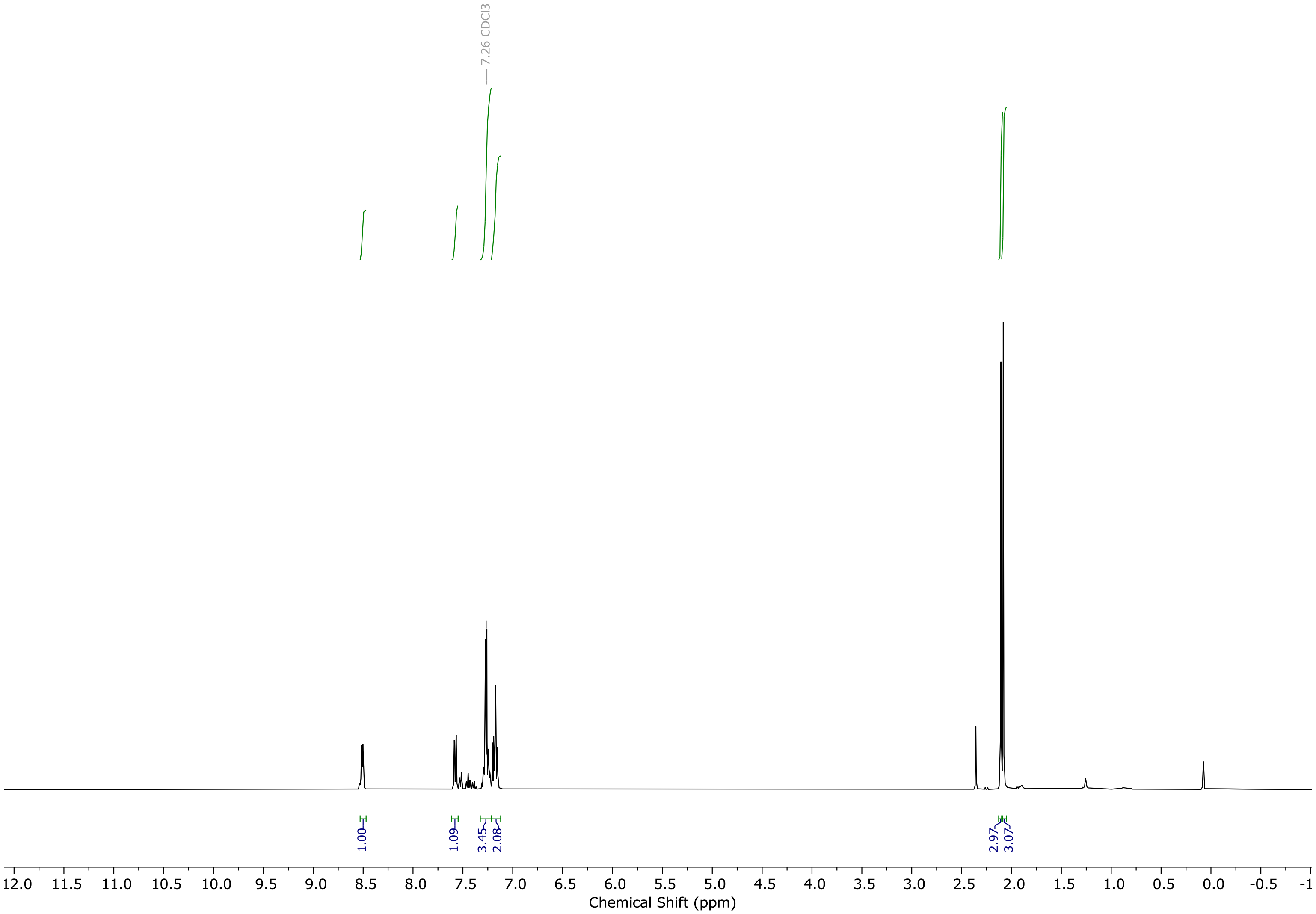

^{13}C NMR (101 MHz, CDCl$_3$) of 3-methyl-2-(*o*-tolyl)pyridine 17i

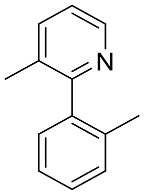

17i

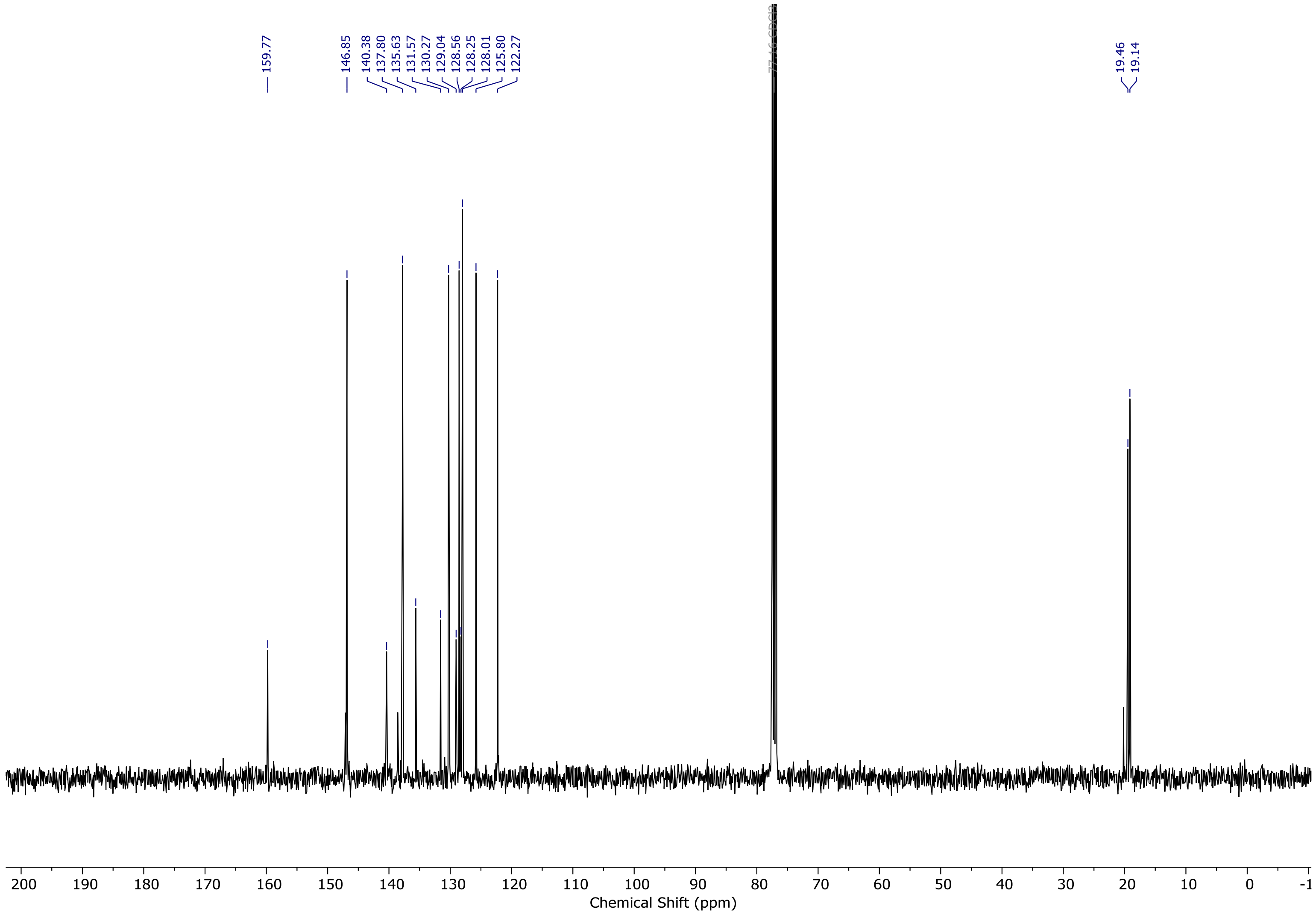

^{1}H NMR (400 MHz, CDCl₃) of 2,8-dimethyl-2-(4,8,12-trimethyltridecyl)chroman-7-yl-4-methyl-3-(pyridin-2-yl) 17j from δ-tocopherol derivative

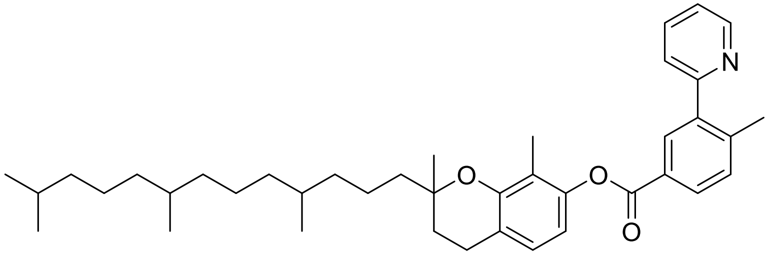

17j

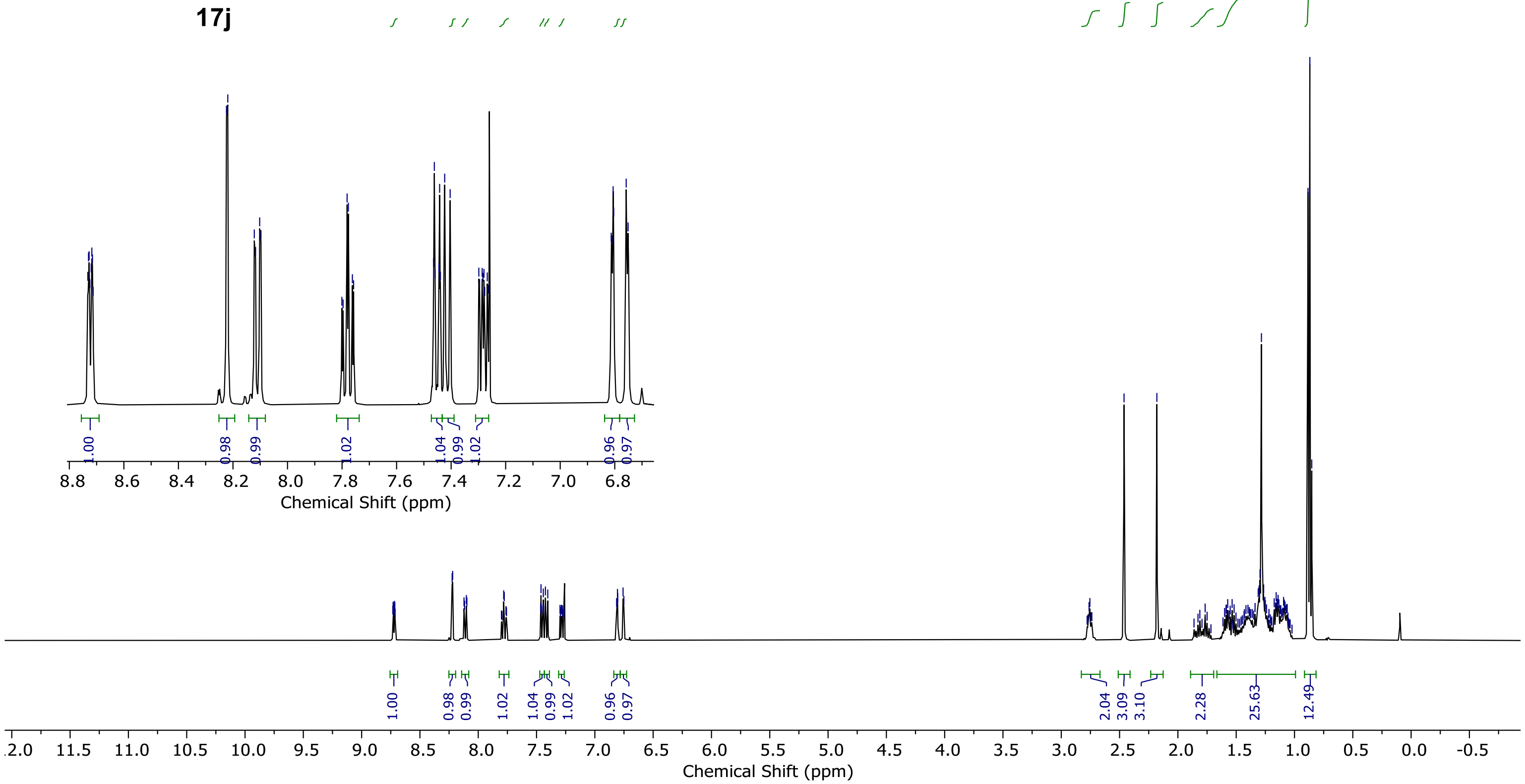

^{13}C NMR (101 MHz, $CDCl_3$) of 2,8-dimethyl-2-(4,8,12-trimethyltridecyl)chroman-7-yl-4-methyl-3-(pyridin-2-yl)benzoate 17j from δ-tocopherol derivative

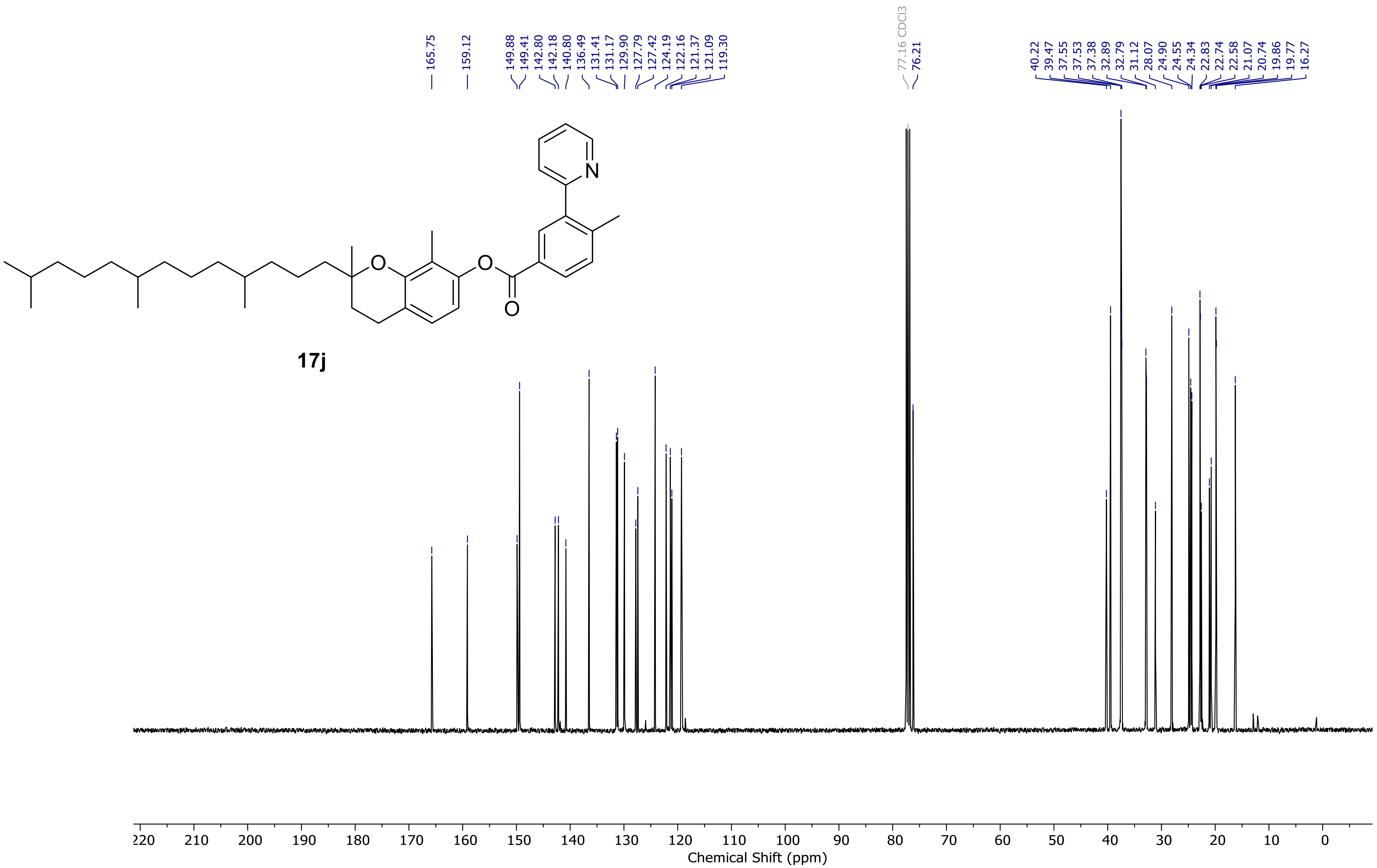

^{1}H NMR (400 MHz, $CDCl_3$) of 18b

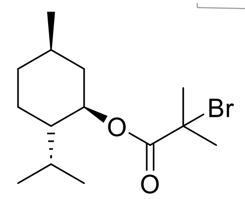

18b

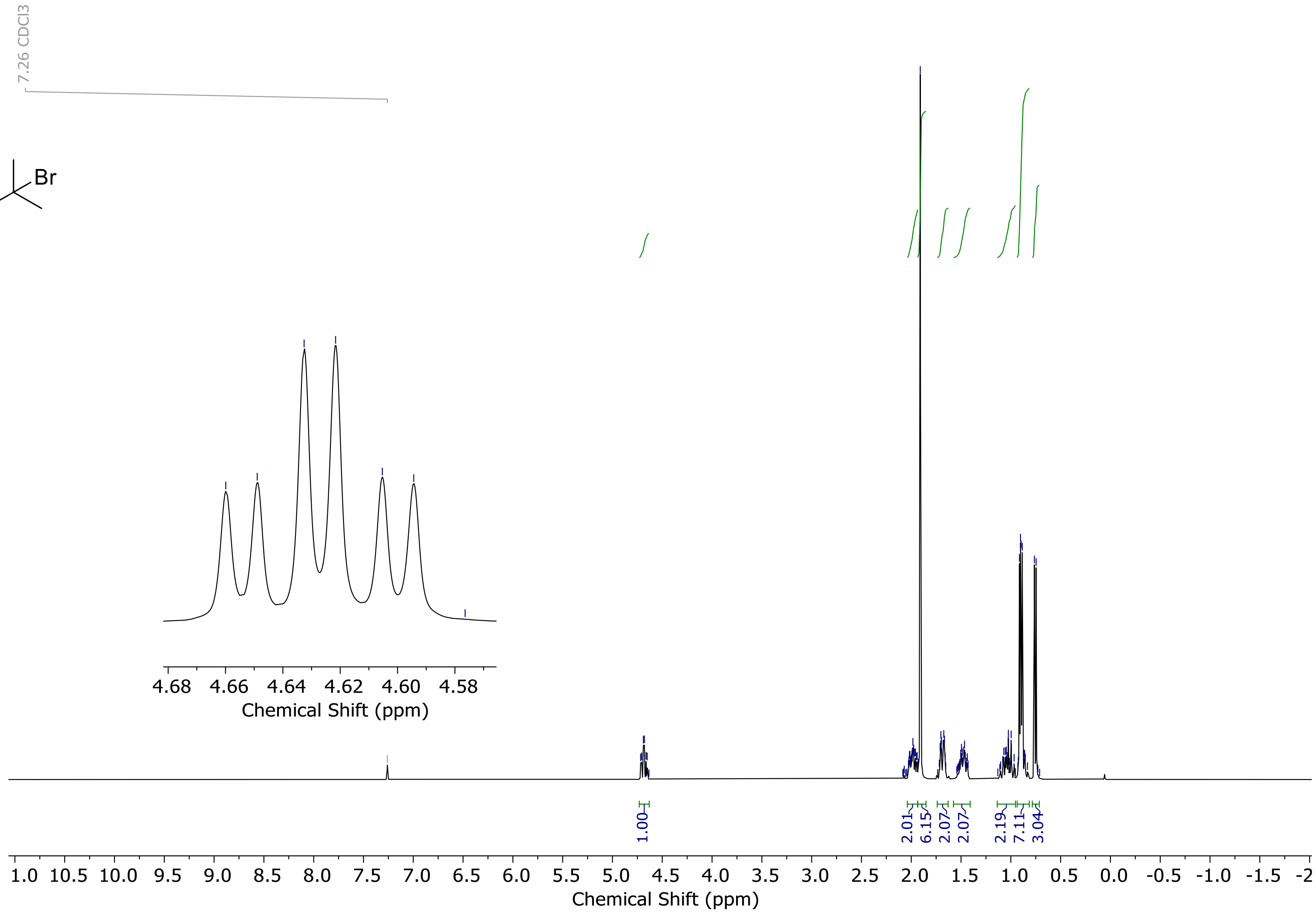

^{13}C NMR (126 MHz, $CDCl_3$) of 18b

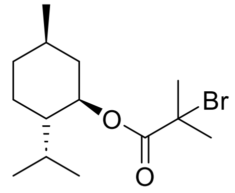

18b

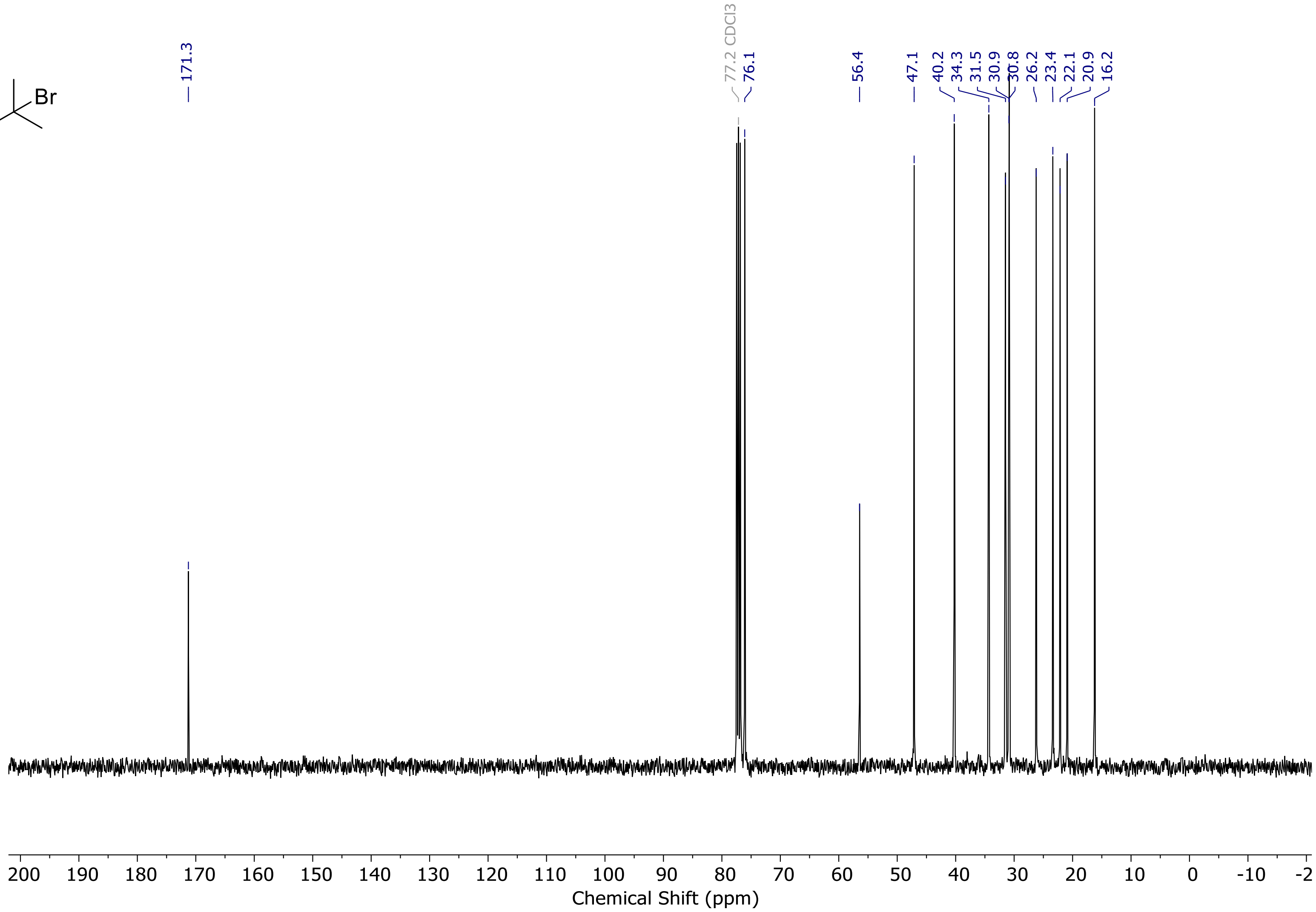

^{1}H NMR (500 MHz, $CDCl_3$) of 20

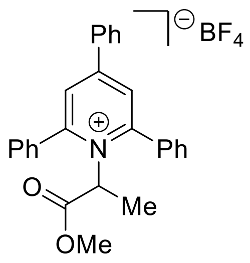

20

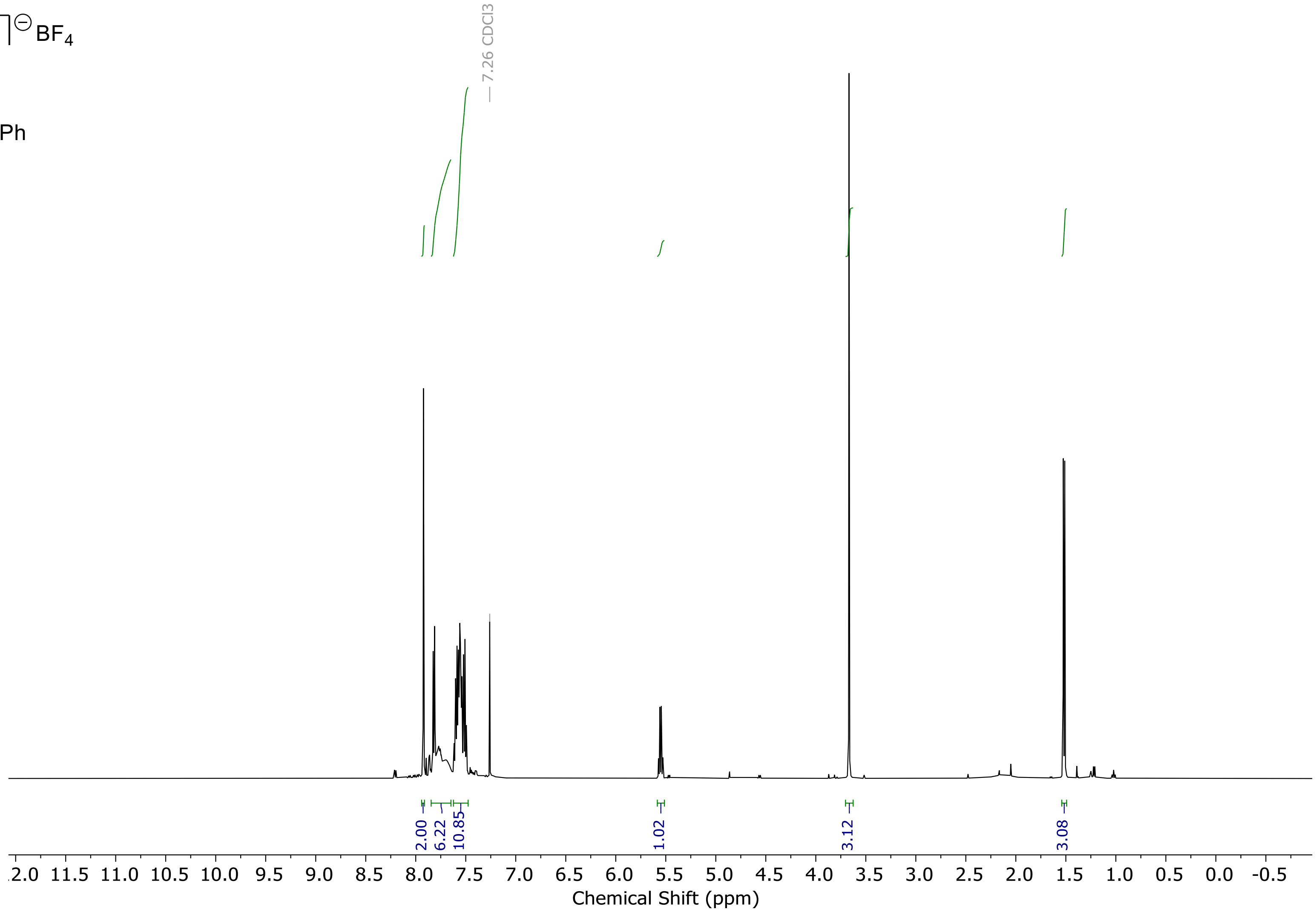

^{13}C NMR (126 MHz, CDCl$_3$) of 20

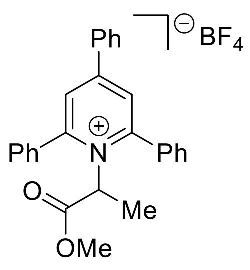

20

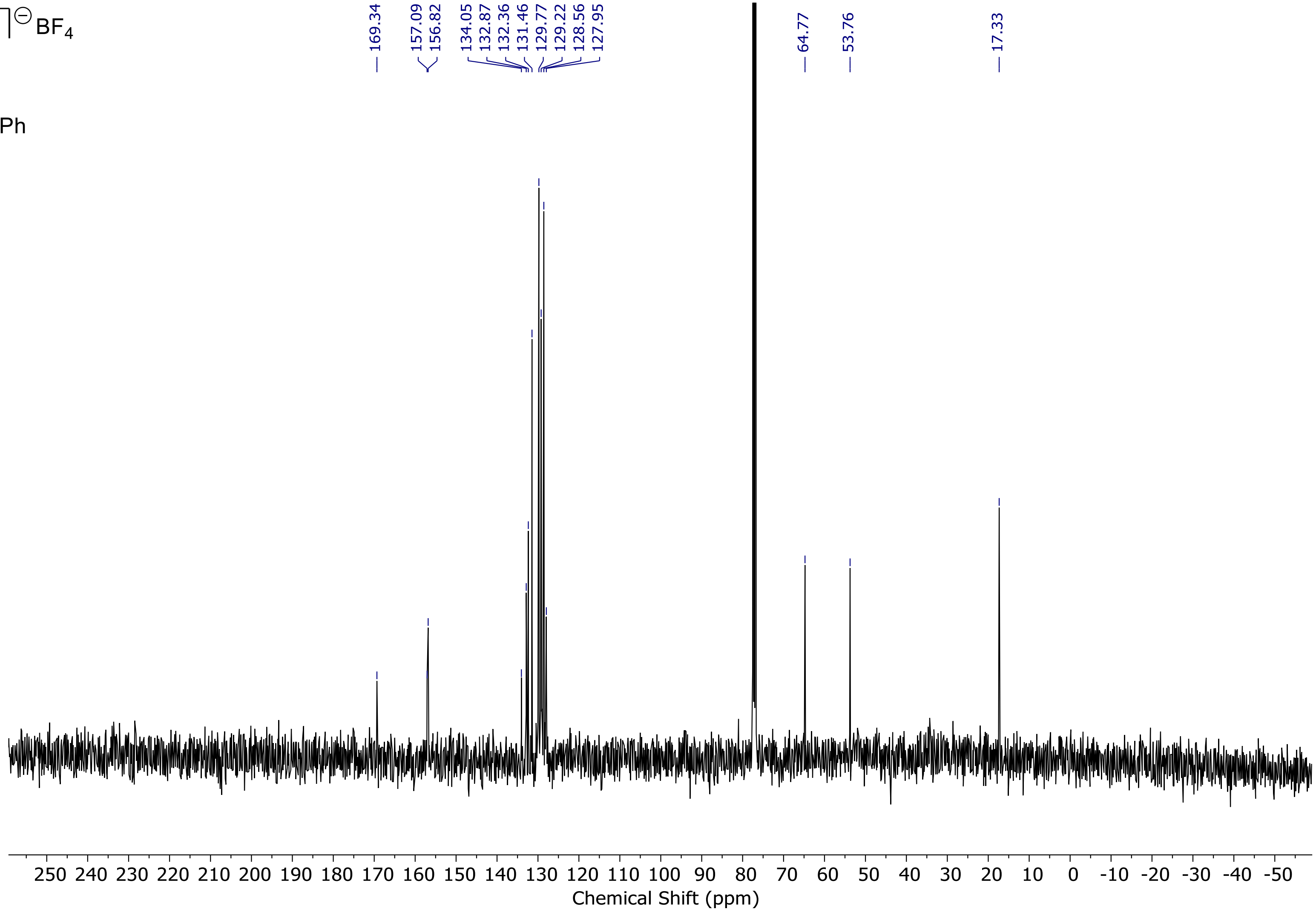

^{19}F NMR (376 MHz, CDCl$_3$) of 20

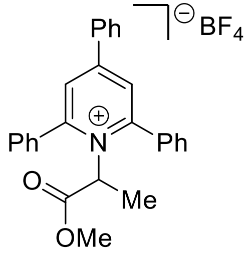

20

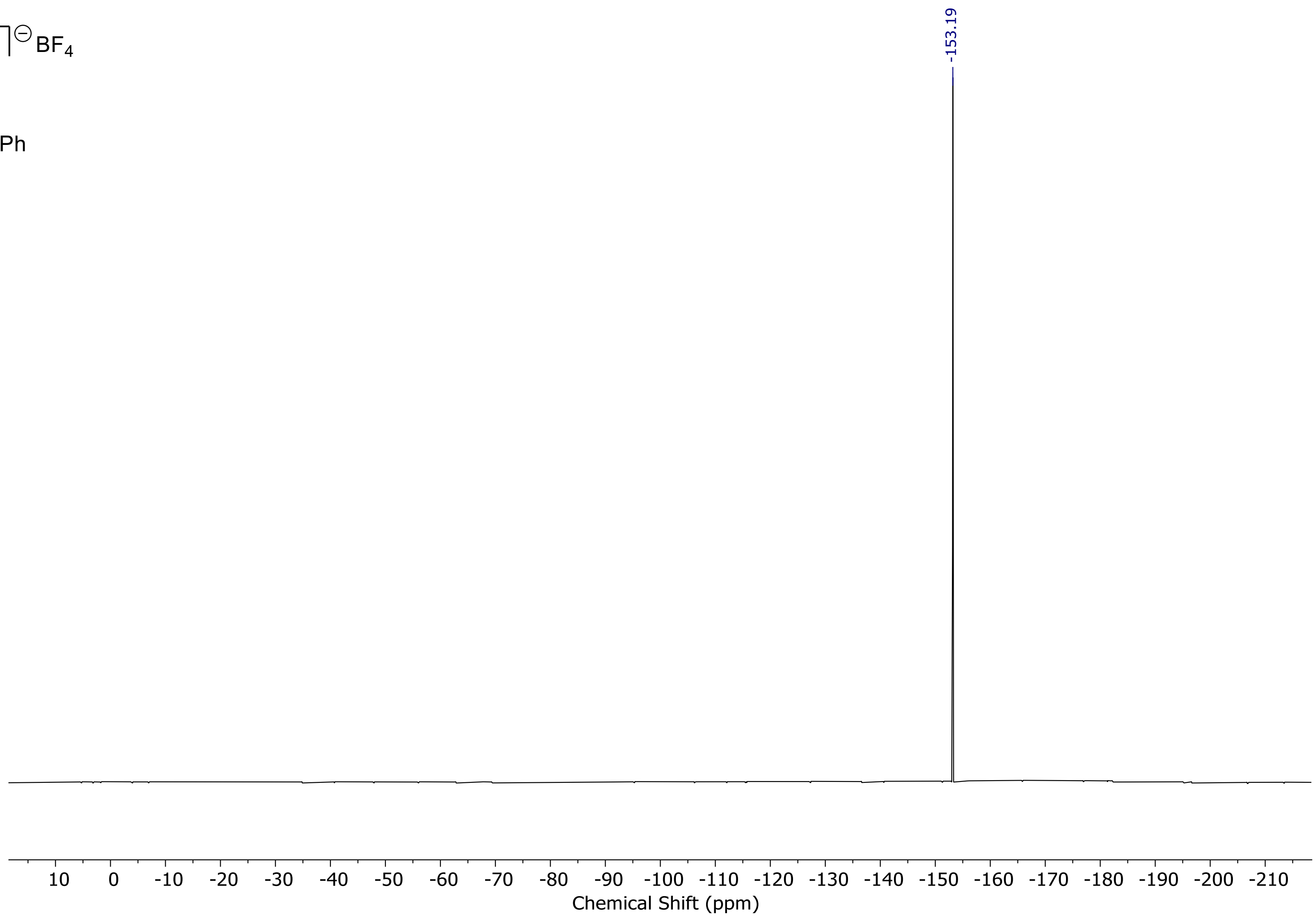

^{1}H NMR (500 MHz, $CDCl_3$) of 21

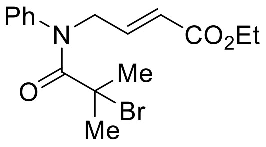

21

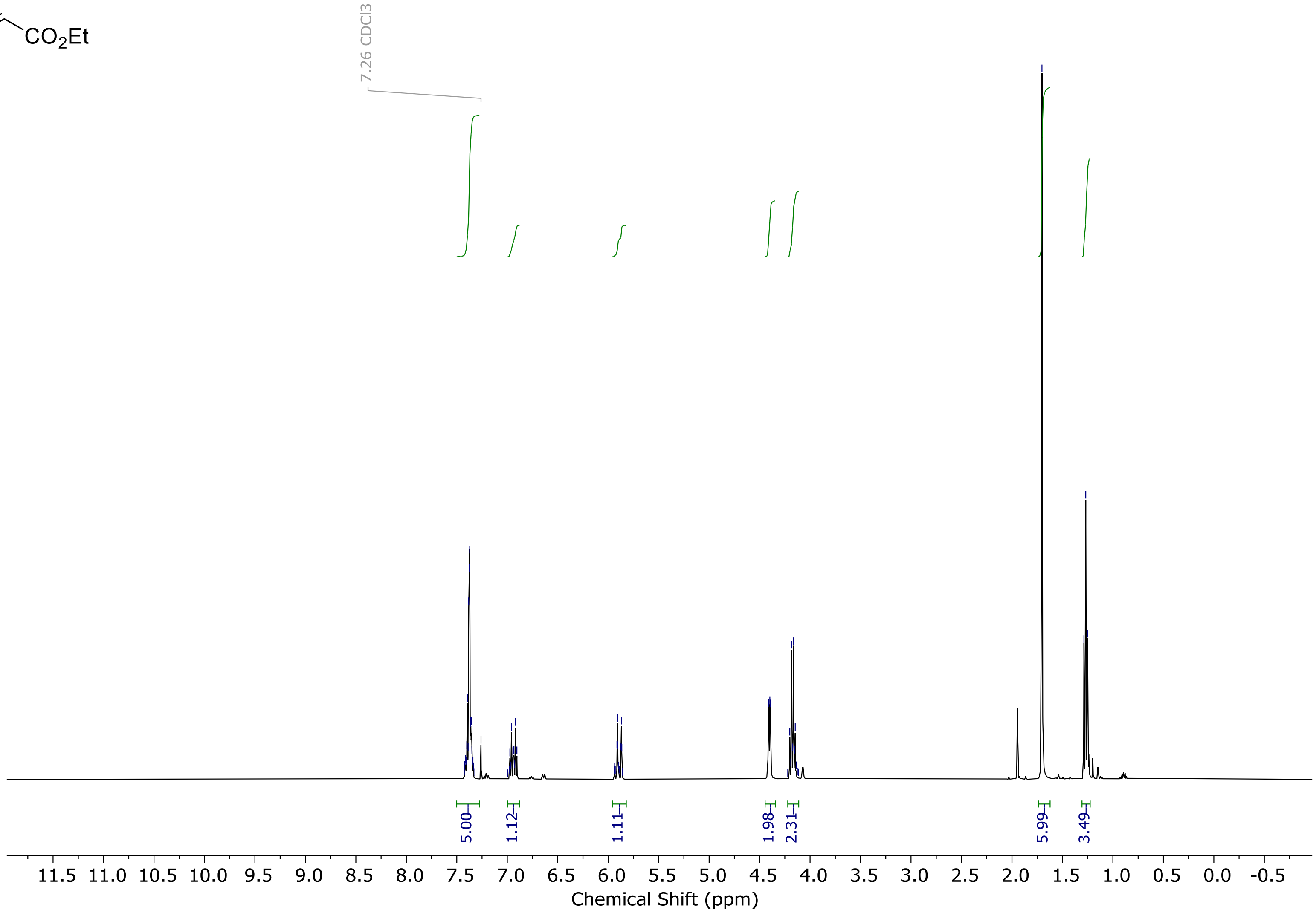

^{13}C NMR (101 MHz, $CDCl_3$) of 21

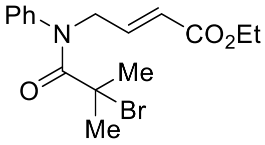

21

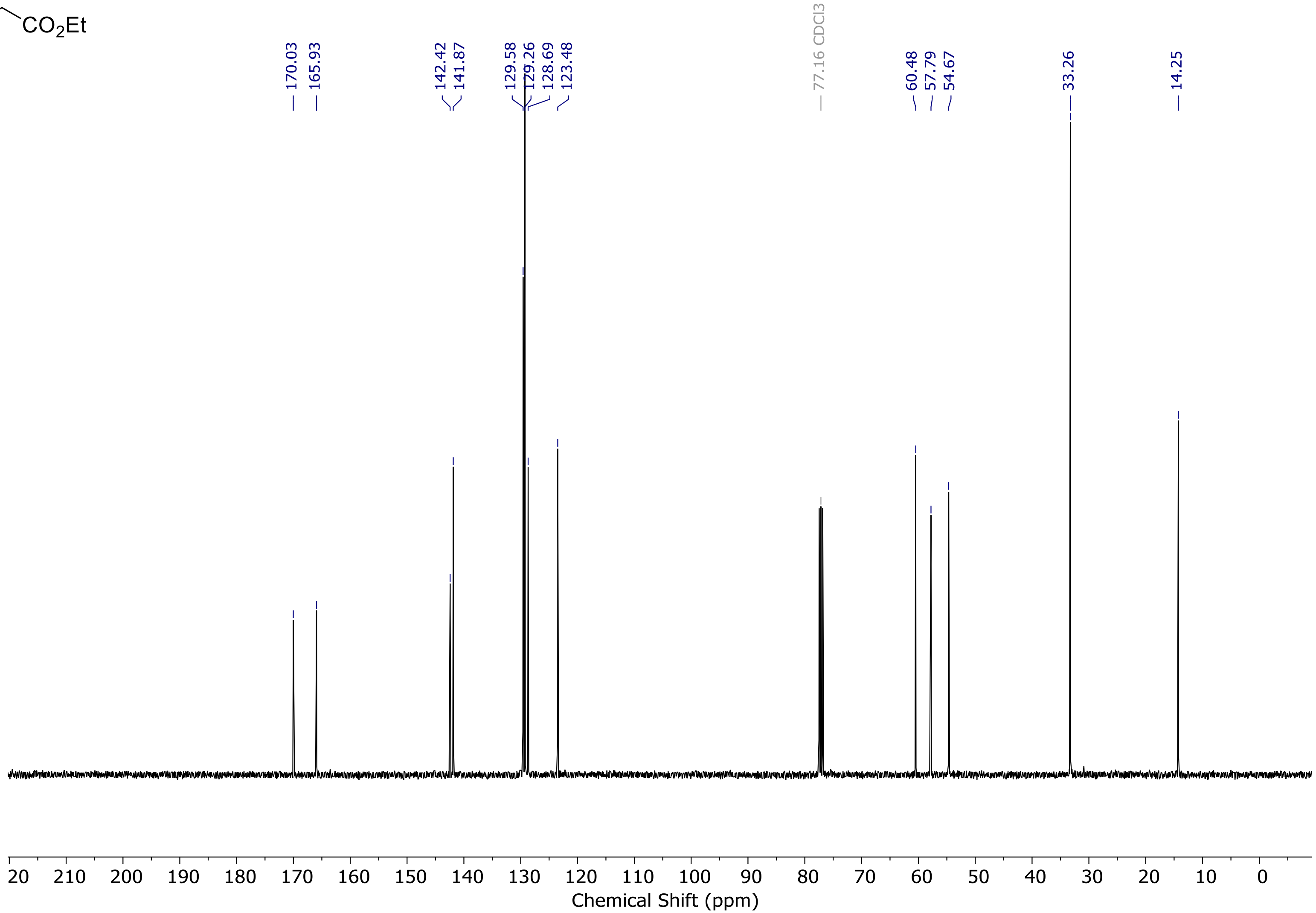

^{1}H NMR (500 MHz, $CDCl_3$) of 2-(3-(tert-butyl)phenyl)pyridine 19a

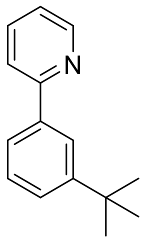

19a

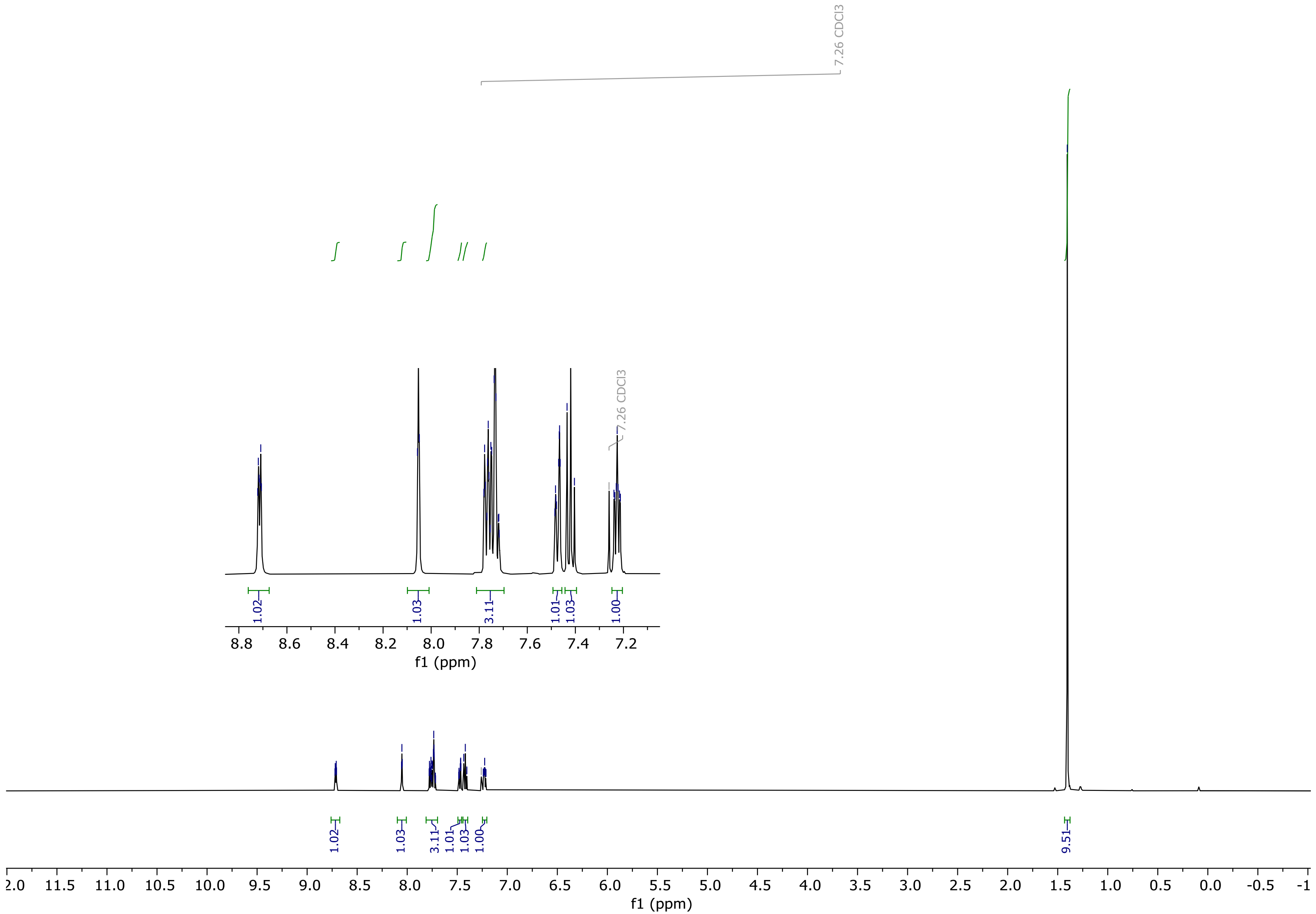

^{13}C NMR (126 MHz, $CDCl_3$) of 2-(3-(tert-butyl)phenyl)pyridine 19a

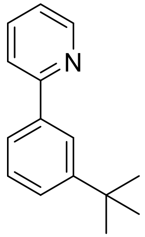

19a

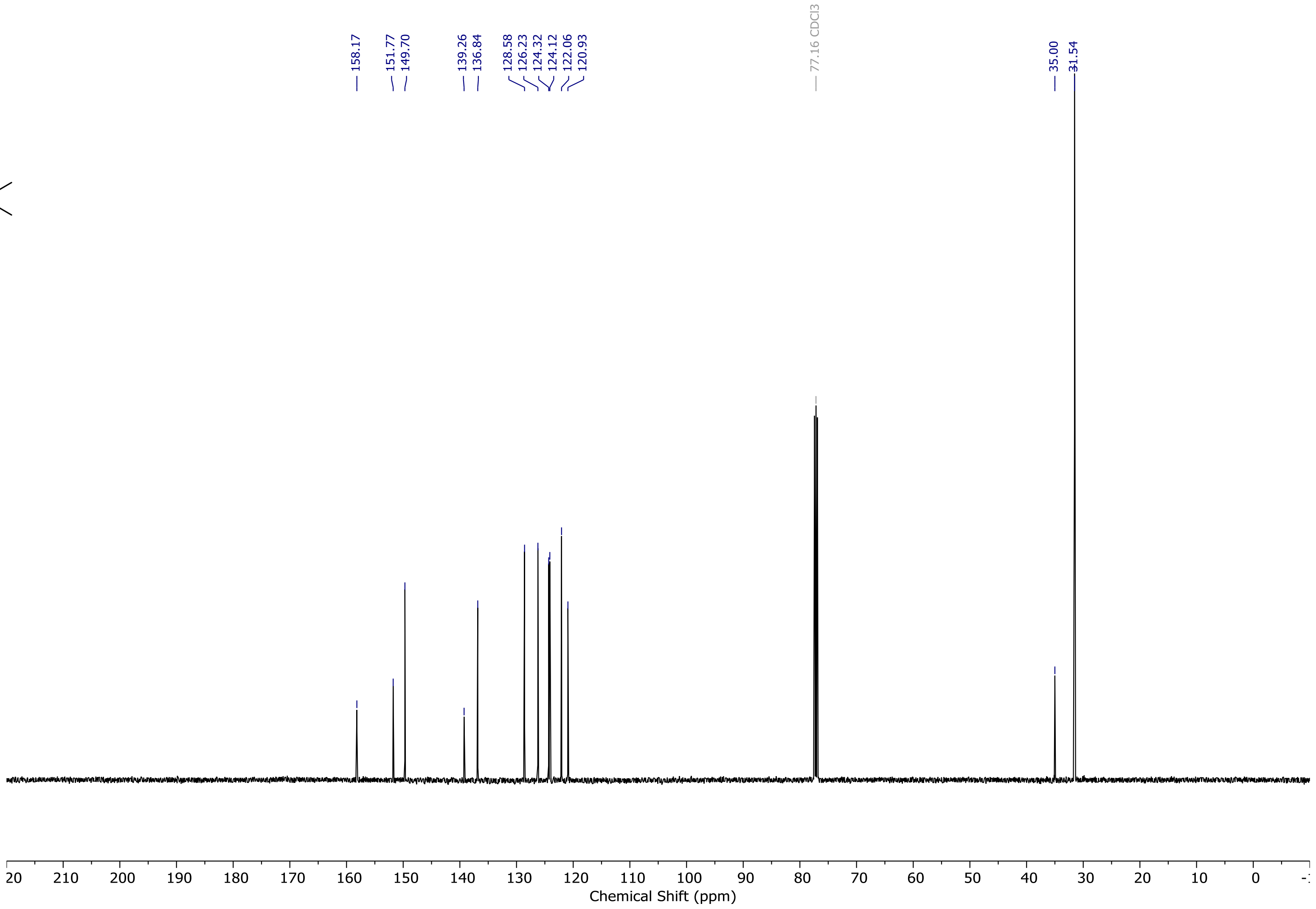

^{1}H NMR (400 MHz, $CDCl_3$) of (1S,2R,5S)-2-isopropyl-5-methylcyclohexyl 2-methyl-2-(3-(pyridin-2-yl)phenyl)propanoate 19b

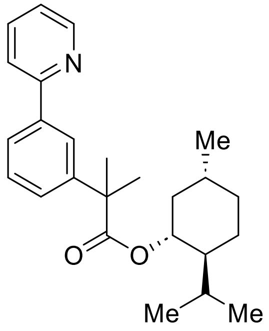

19b

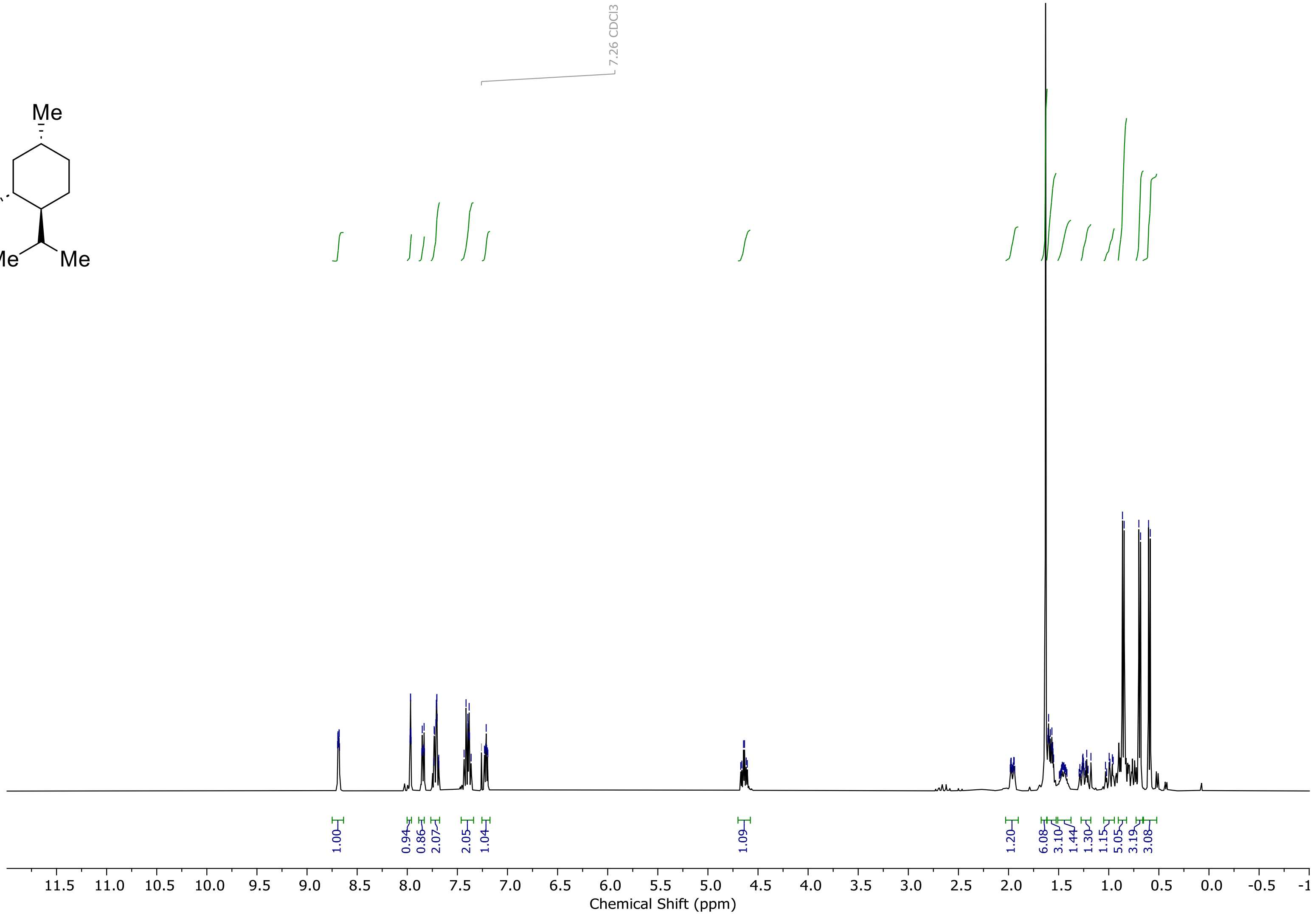

^{13}C NMR (101 MHz, CDCl₃) of (1S,2R,5S)-2-isopropyl-5-methylcyclohexyl 2-methyl-2-(3-(pyridin-2-yl)phenyl)propanoate 19b

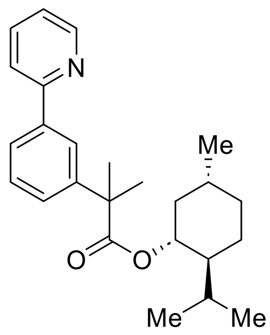

19b

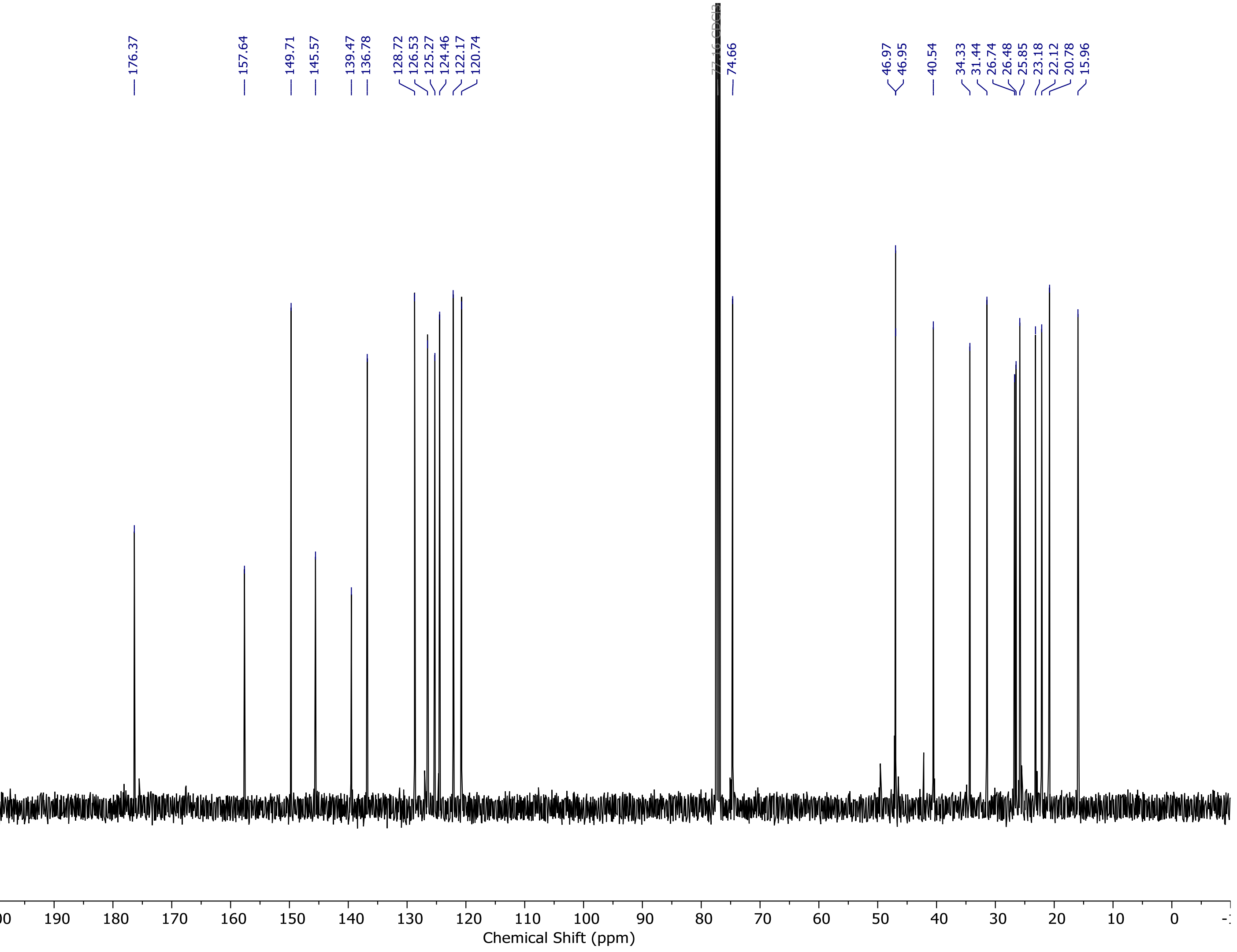

^{1}H NMR (400 MHz, $CDCl_3$) of 1-(3-(tert-butyl)phenyl)-1H-pyrazole 19c

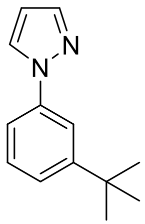

19c

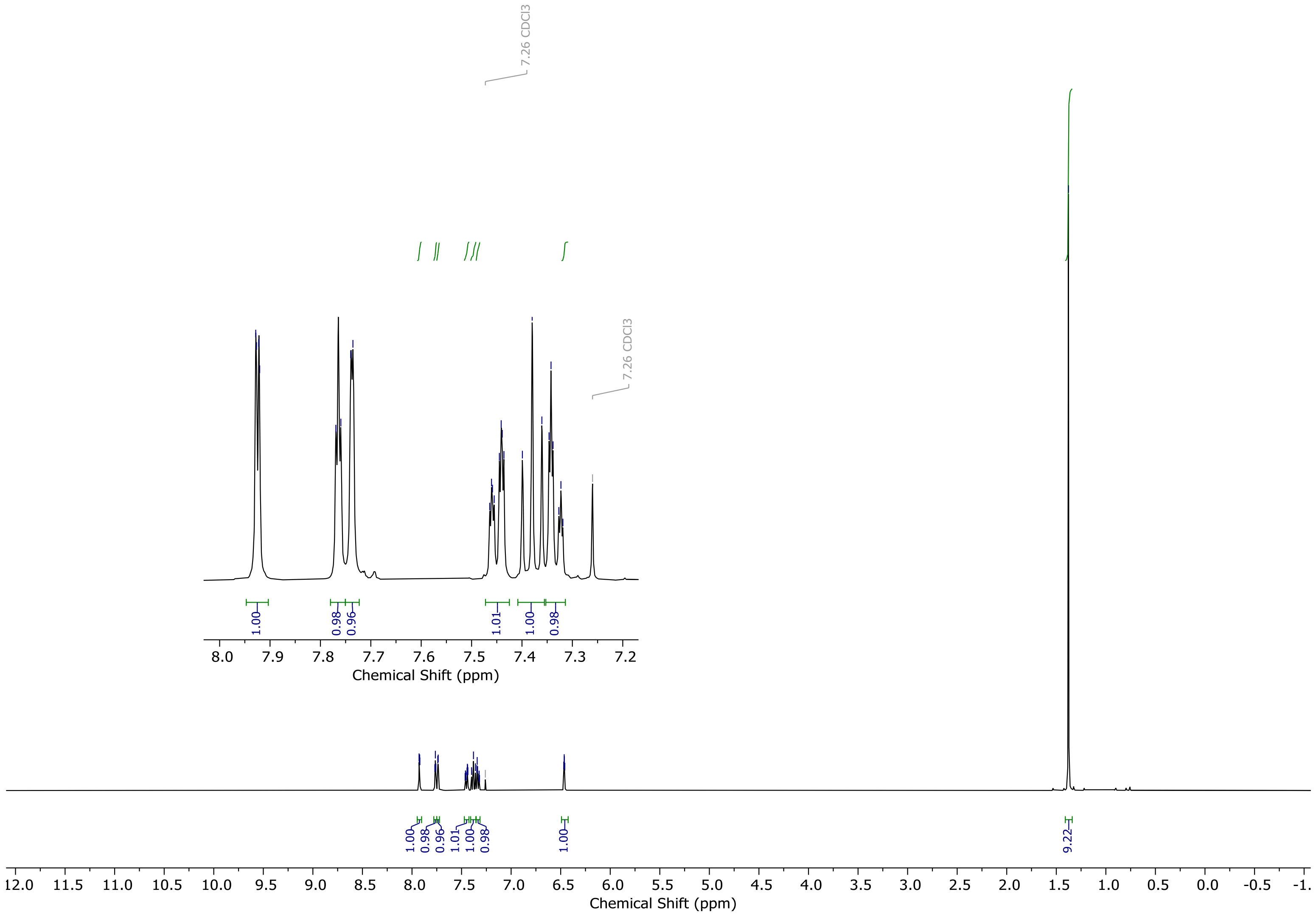

^{13}C NMR (101 MHz, $CDCl_3$) of 1-(3-(tert-butyl)phenyl)-1H-pyrazole 19c

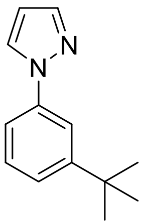

19c

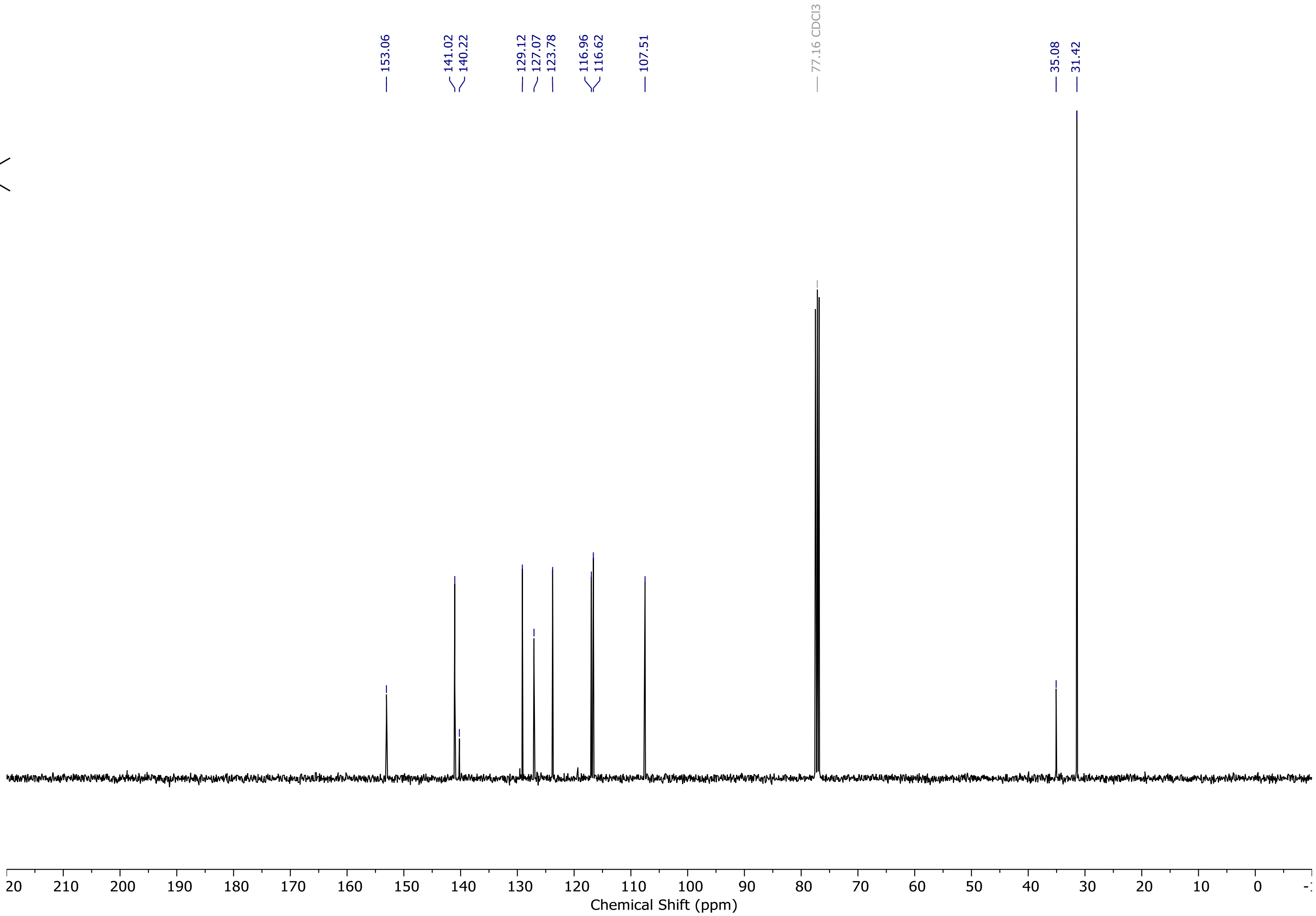

^{1}H NMR (400 MHz, $CDCl_3$) of 2-(3-(tert-butyl)phenyl)-4,5-dihydrooxazole 19d

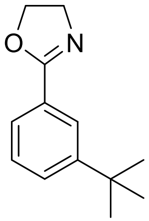

19d

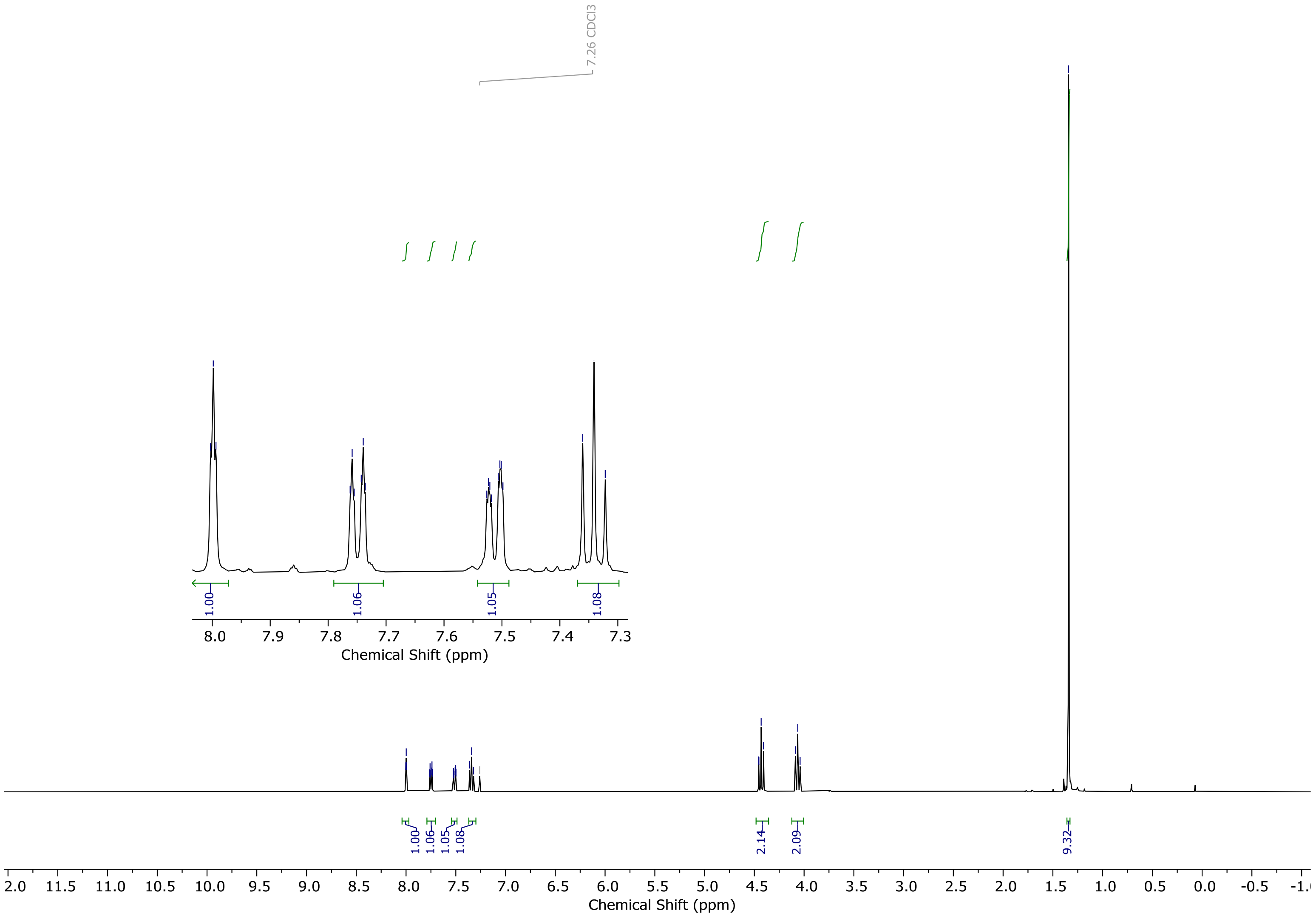

^{13}C NMR (101 MHz, $CDCl_3$) of 2-(3-(tert-butyl)phenyl)-4,5-dihydrooxazole 19d

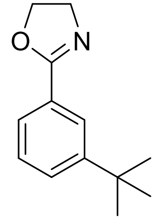

19d

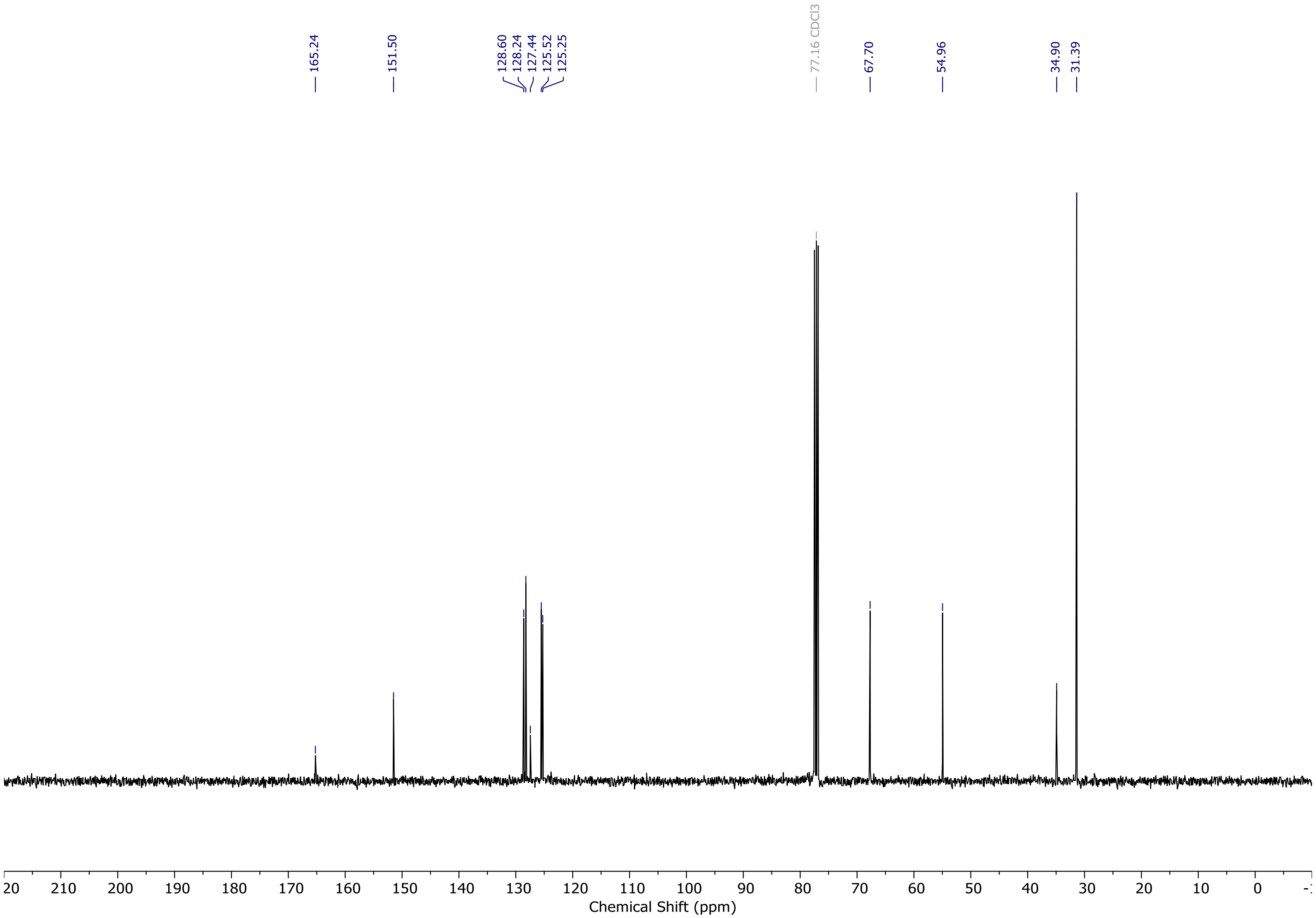

^{1}H NMR (400 MHz, $CDCl_3$) of methyl 2-(3-(pyridin-2-yl)phenyl)propanoate 19e

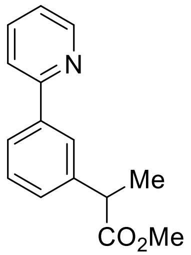

19e

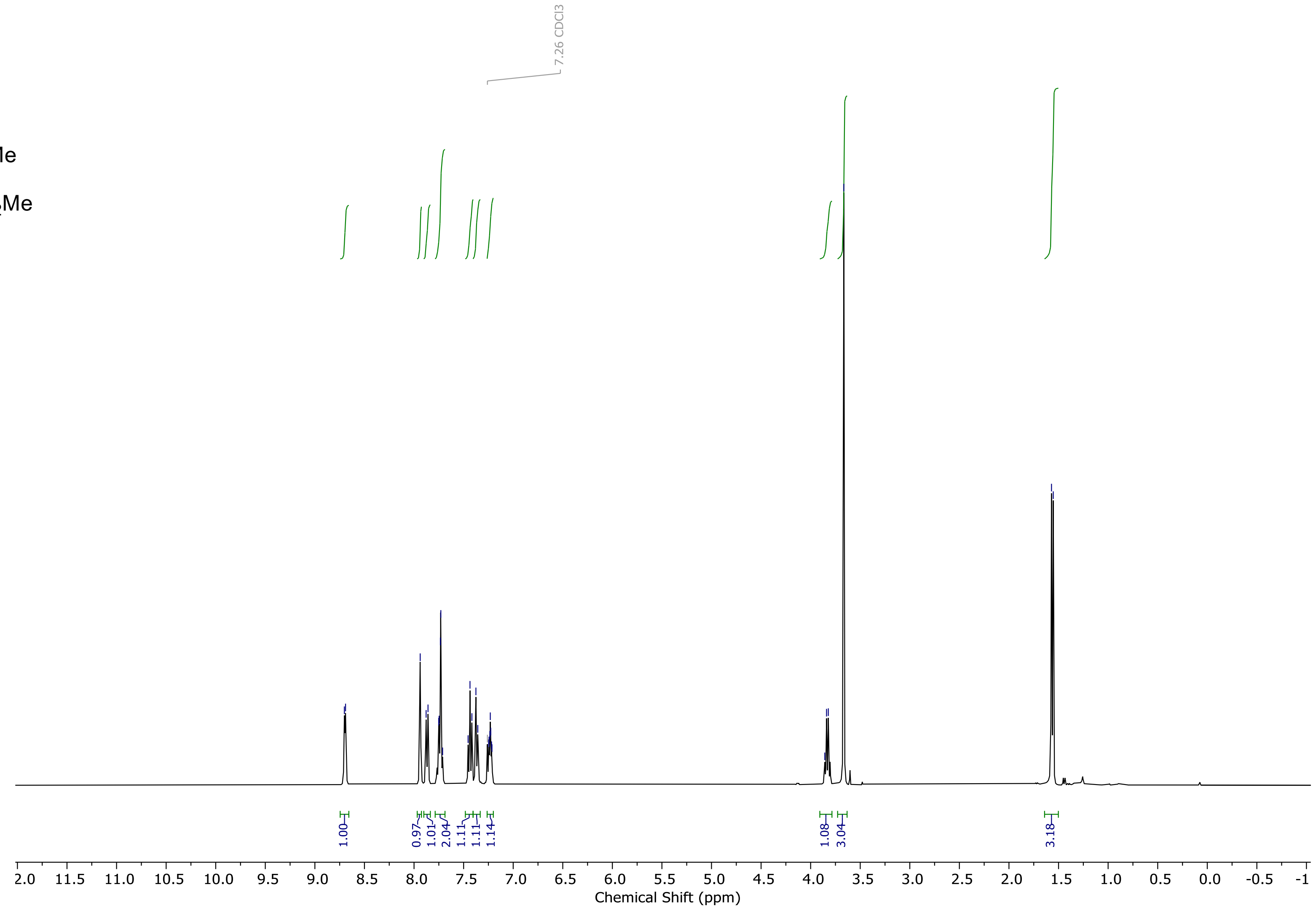

^{13}C NMR (101 MHz, $CDCl_3$) of methyl 2-(3-(pyridin-2-yl)phenyl)propanoate 19e

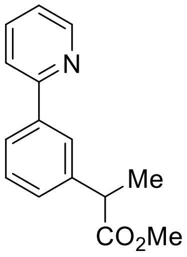

19e

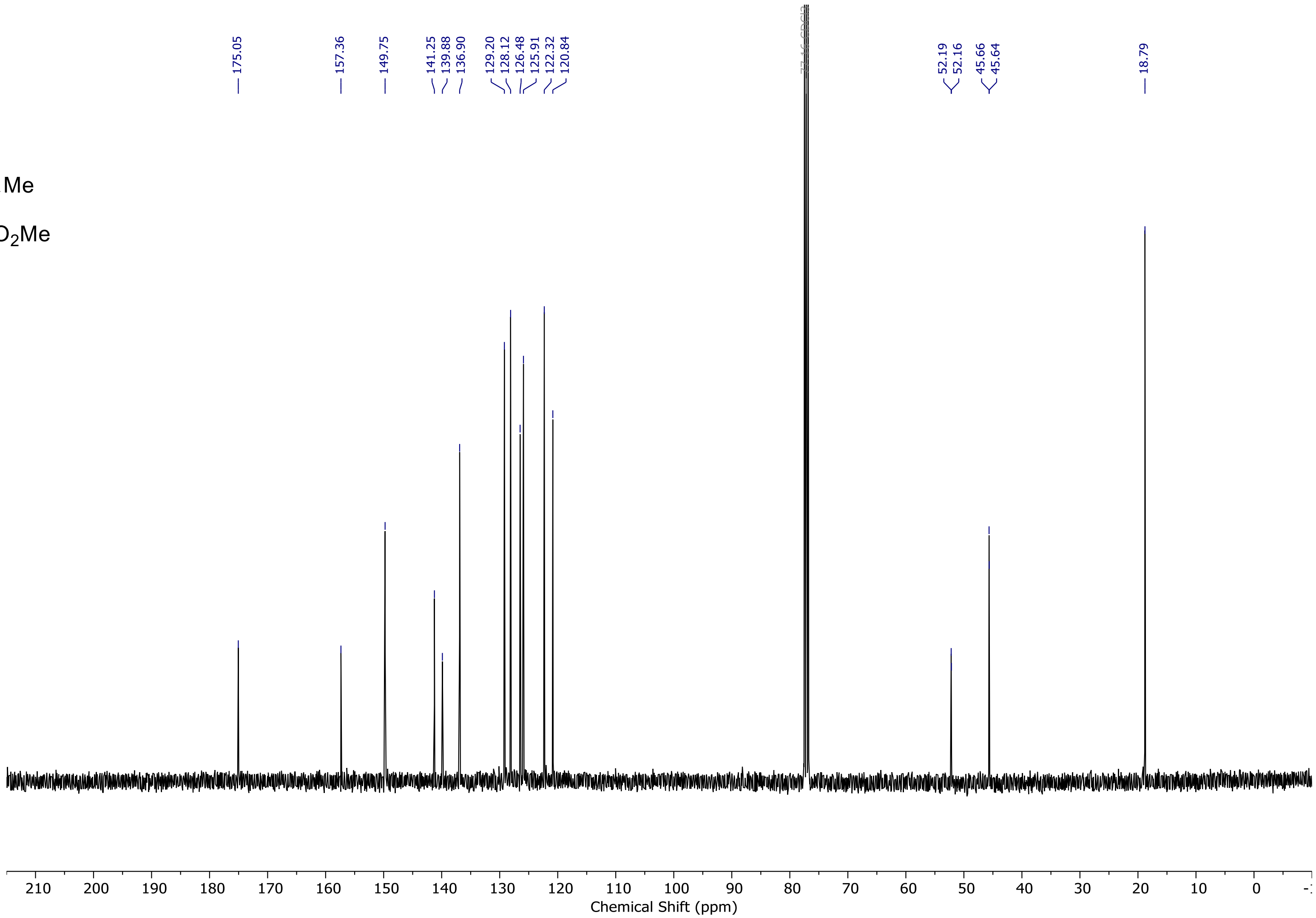

^{1}H NMR (400 MHz, $CDCl_3$) of ethyl 2-(4,4-dimethyl-5-oxo-1-phenylpyrrolidin-3-yl)-2-(3-(pyridin-2-yl)phenyl)acetate 19f

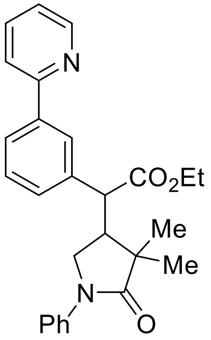

19f

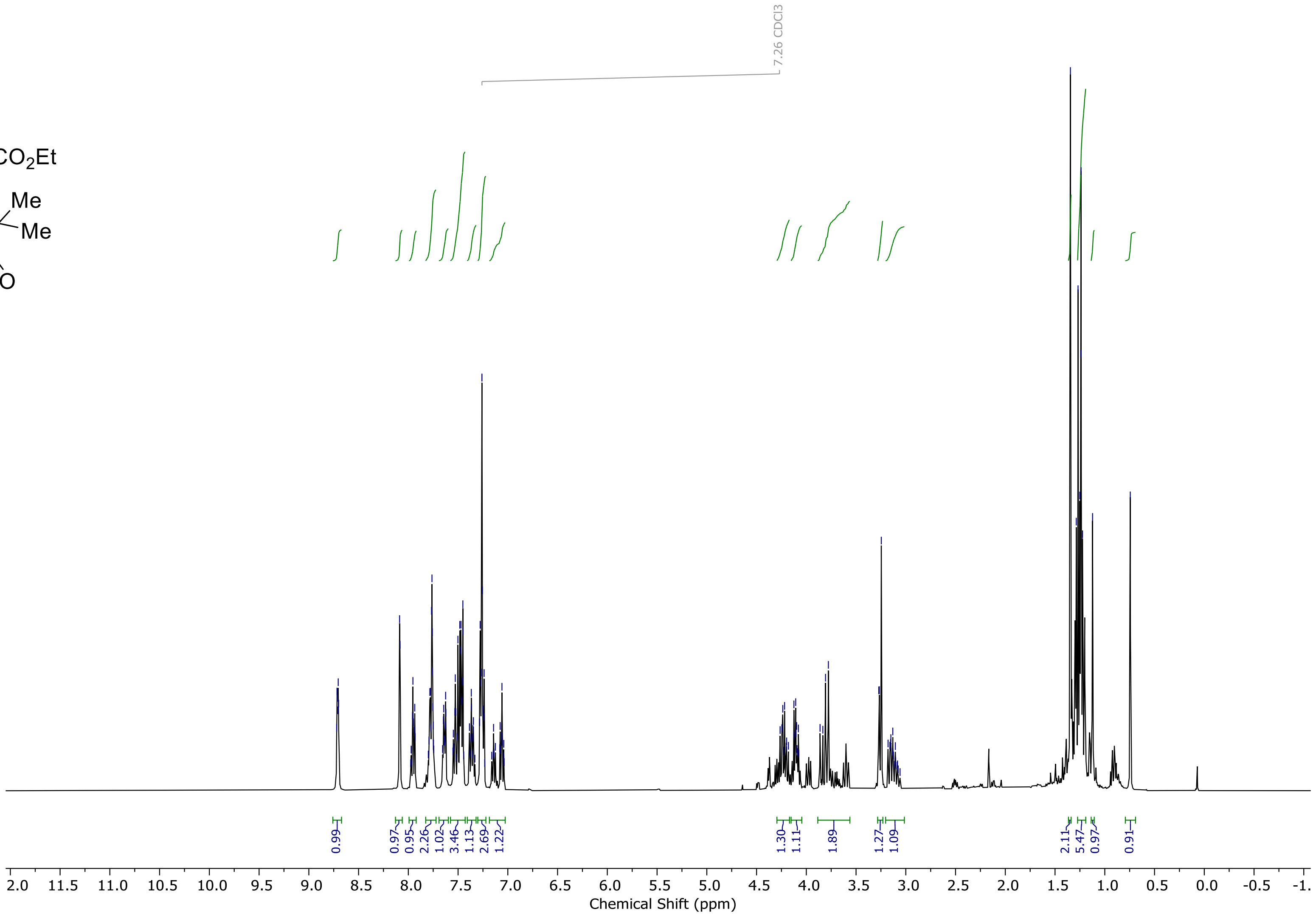

^{13}C NMR (101 MHz, CDCl$_3$) of ethyl 2-(4,4-dimethyl-5-oxo-1-phenylpyrrolidin-3-yl)-2-(3-(pyridin-2-yl)phenyl)acetate 19f

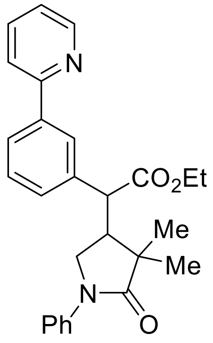

19f

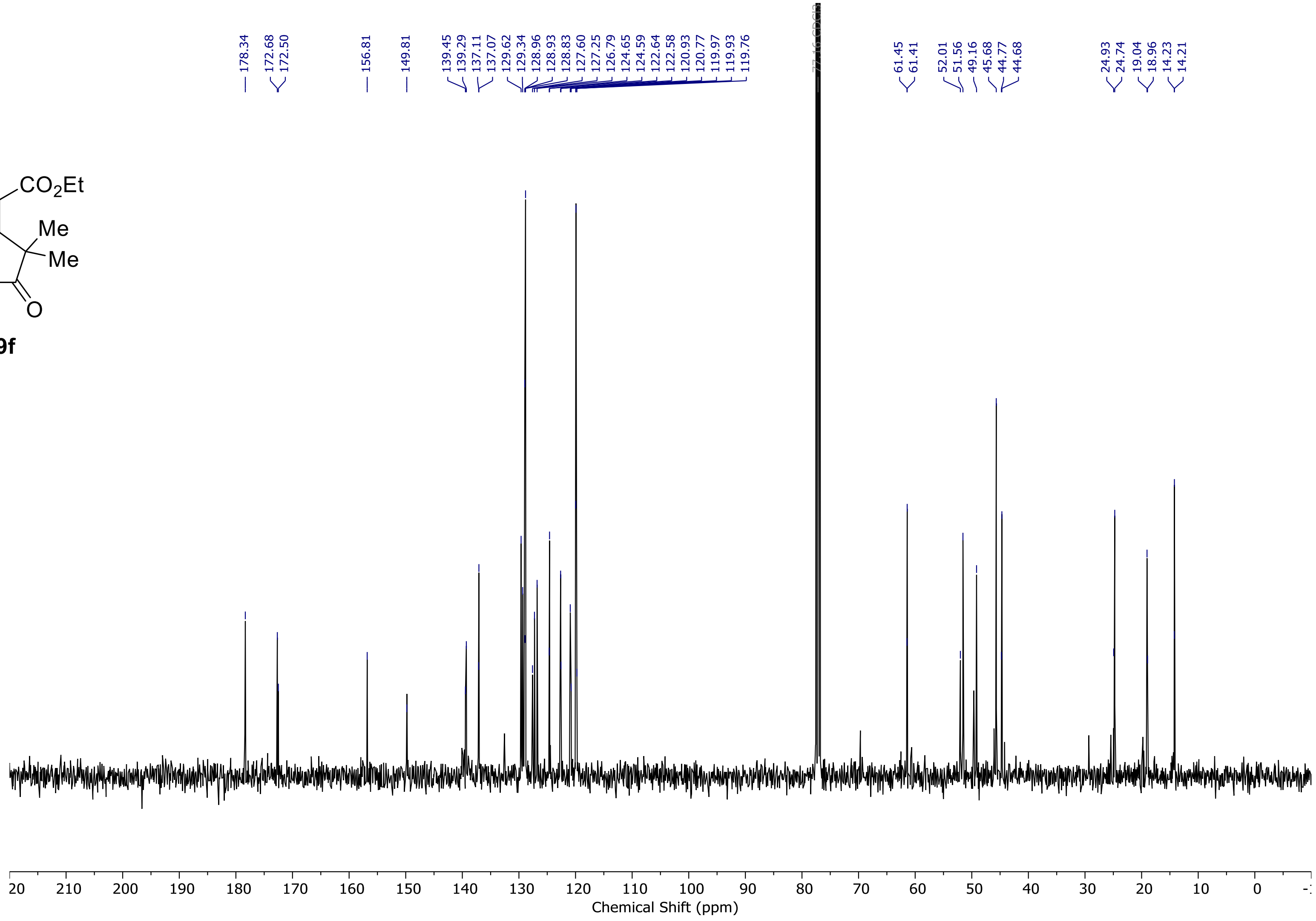

^{1}H NMR (500 MHz, $CDCl_3$) of 2-(Phenyl-2,6-d_2)pyridine-6-d (d_2-8n)

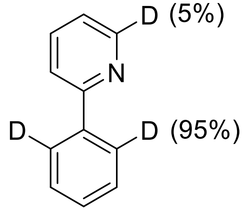

d_2-8n

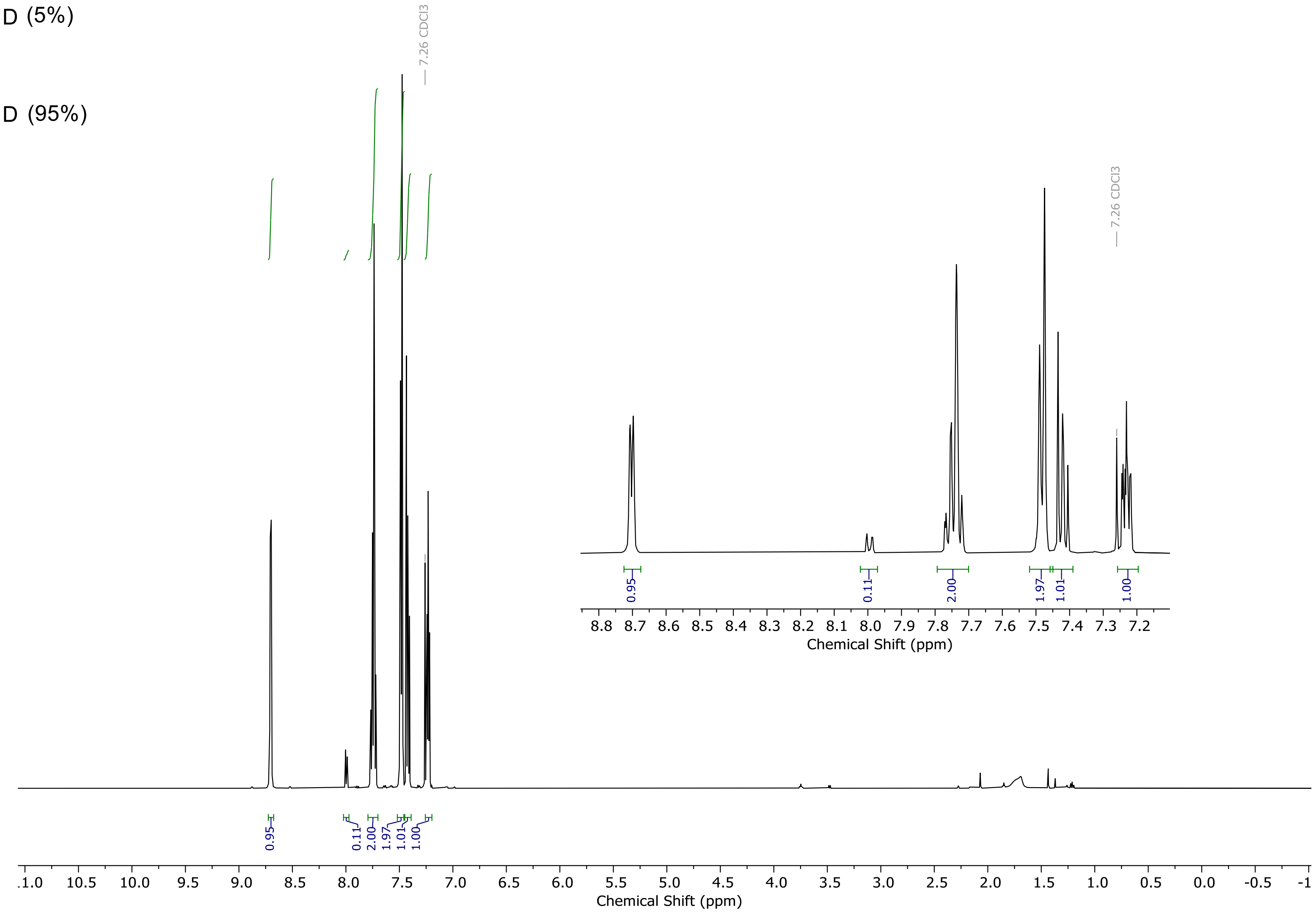

^{2}D NMR (77 MHz, $CHCl_3$) of 2-(Phenyl-2,6-d_2)pyridine-6-d (d_2-8n)

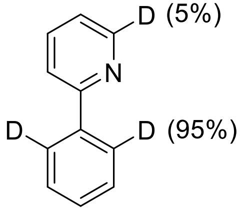

d_2-8n

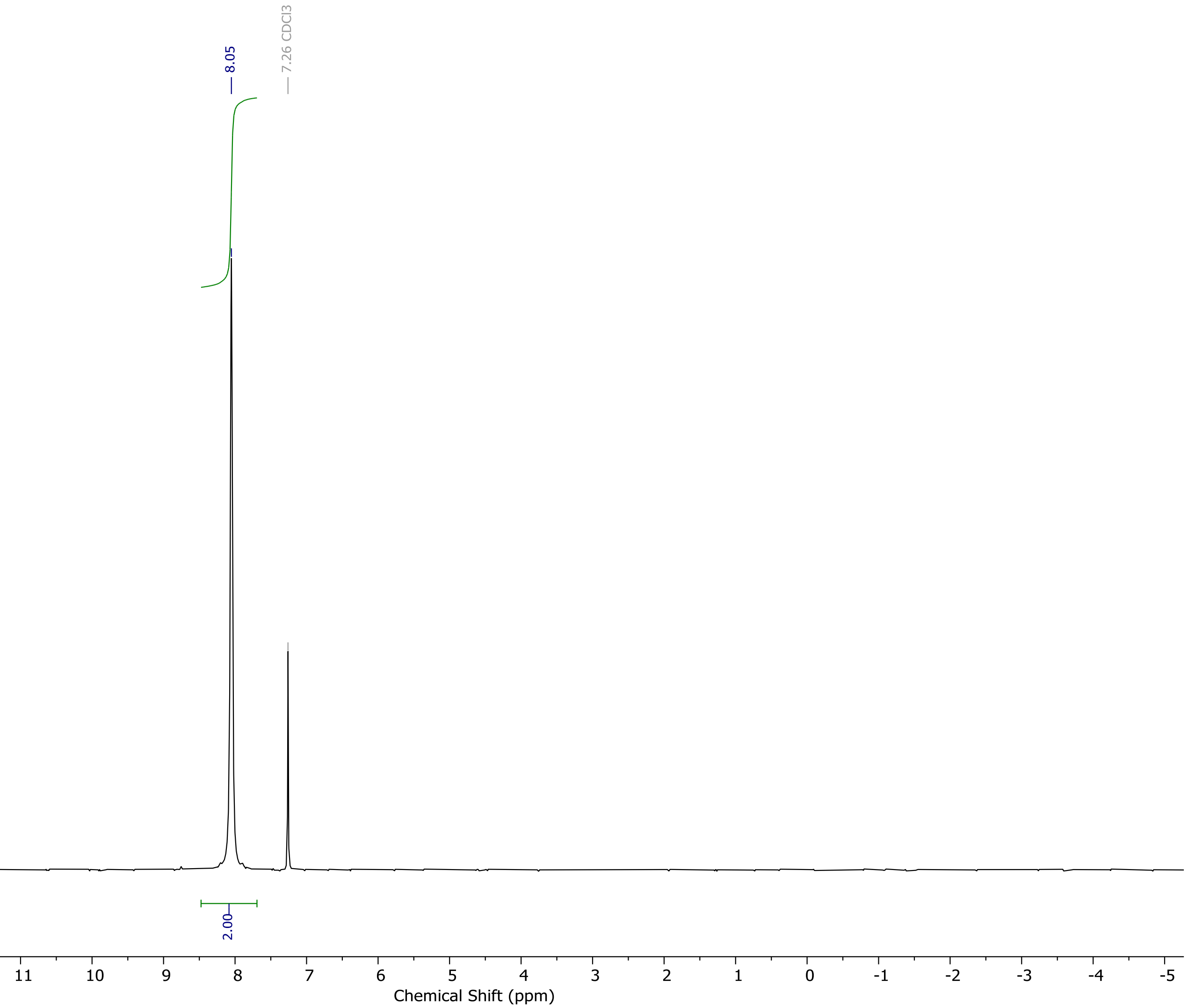

^{13}C NMR (126 MHz, $CDCl_3$) of 2-(Phenyl-2,6-d_2)pyridine-6-d (d_2-8n)

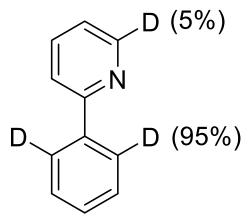

d_2-8n

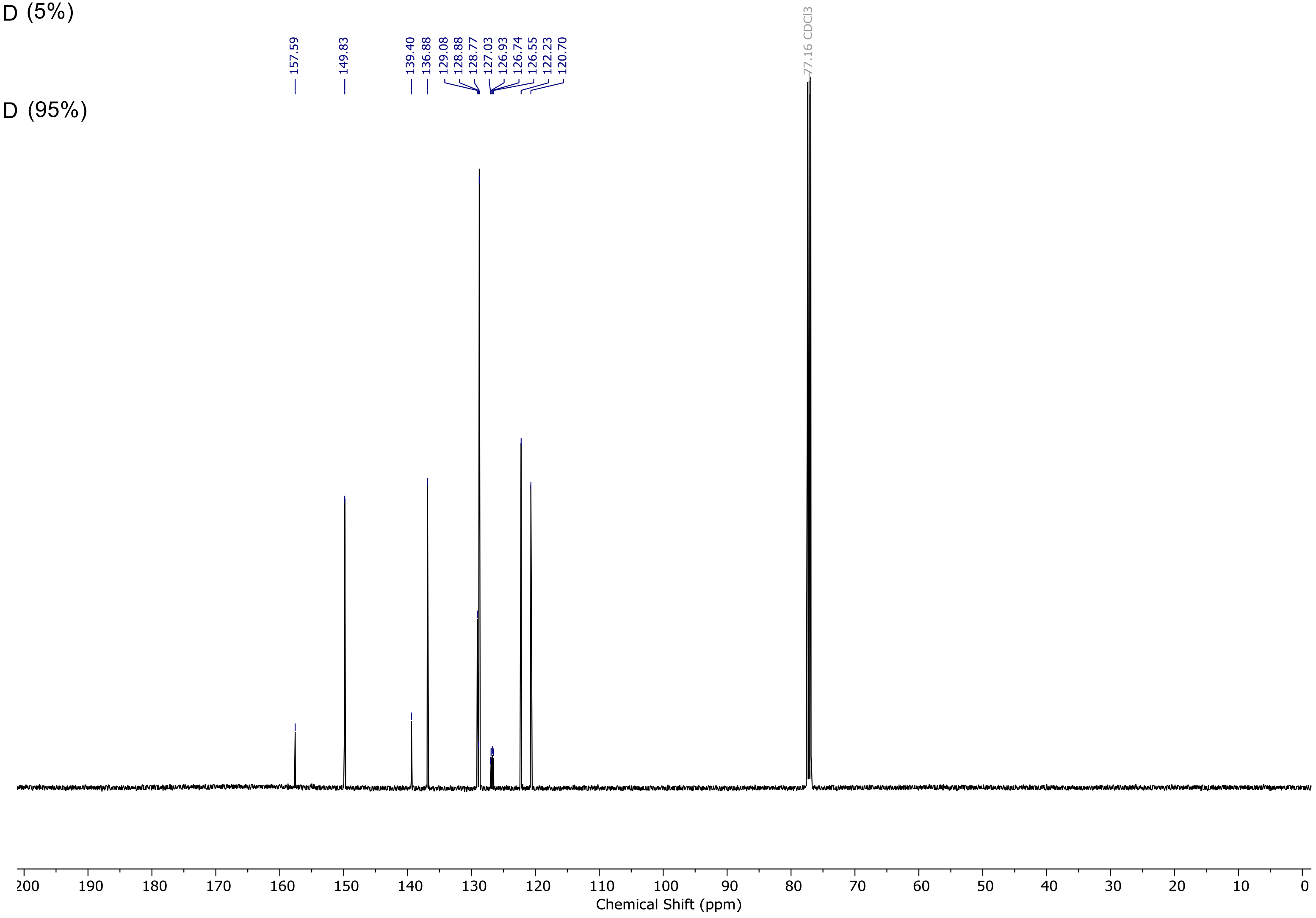

^1H NMR (400 MHz, $CDCl_3$) of 1-(phenyl-2,6-d_2)-1H-pyrazole (d_2-8h)

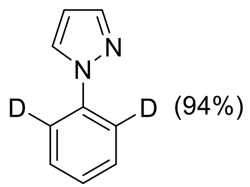

d_2-8h

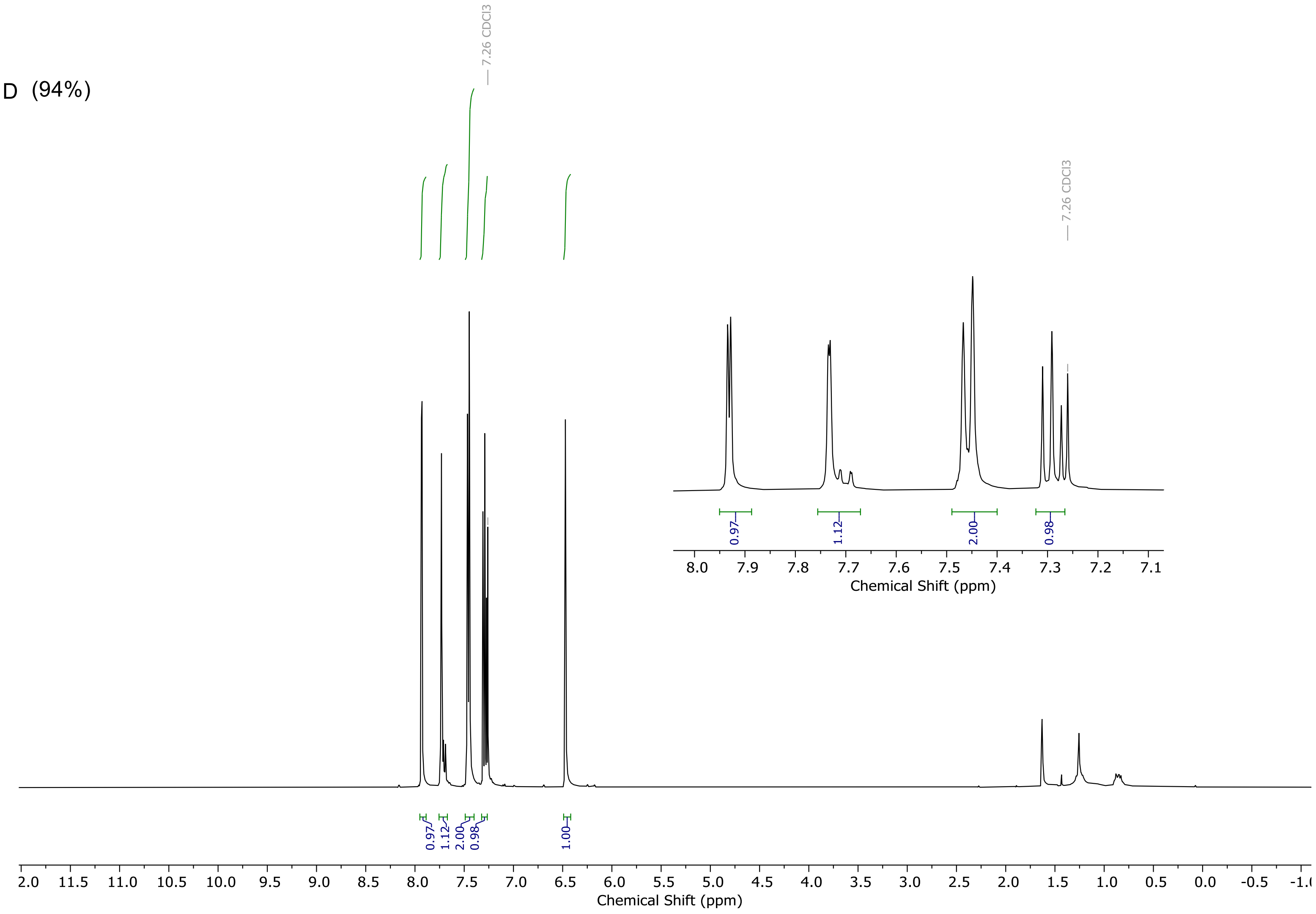

^{2}D NMR (400 MHz, $CDCl_3$) of 1-(phenyl-2,6-d_2)-1H-pyrazole (d_2-8h)

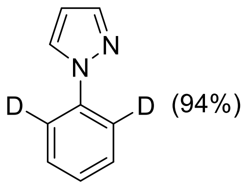

d_2-8h

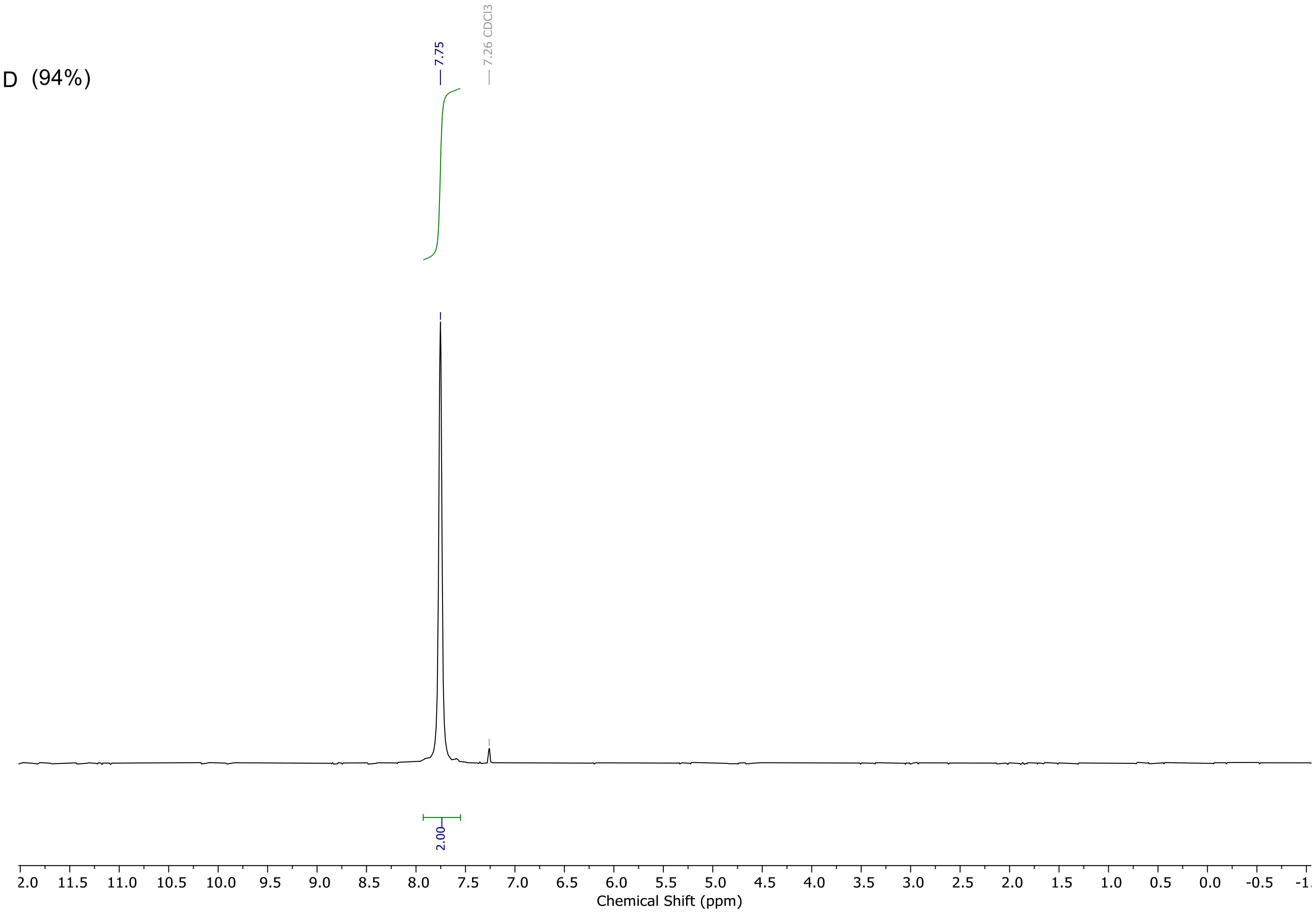

^{13}C NMR (126 MHz, $CDCl_3$) of (1-(phenyl-2,6-d_2)-1H-pyrazole (d_2-8h)

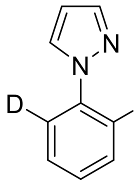

(94%)

d_2-8h

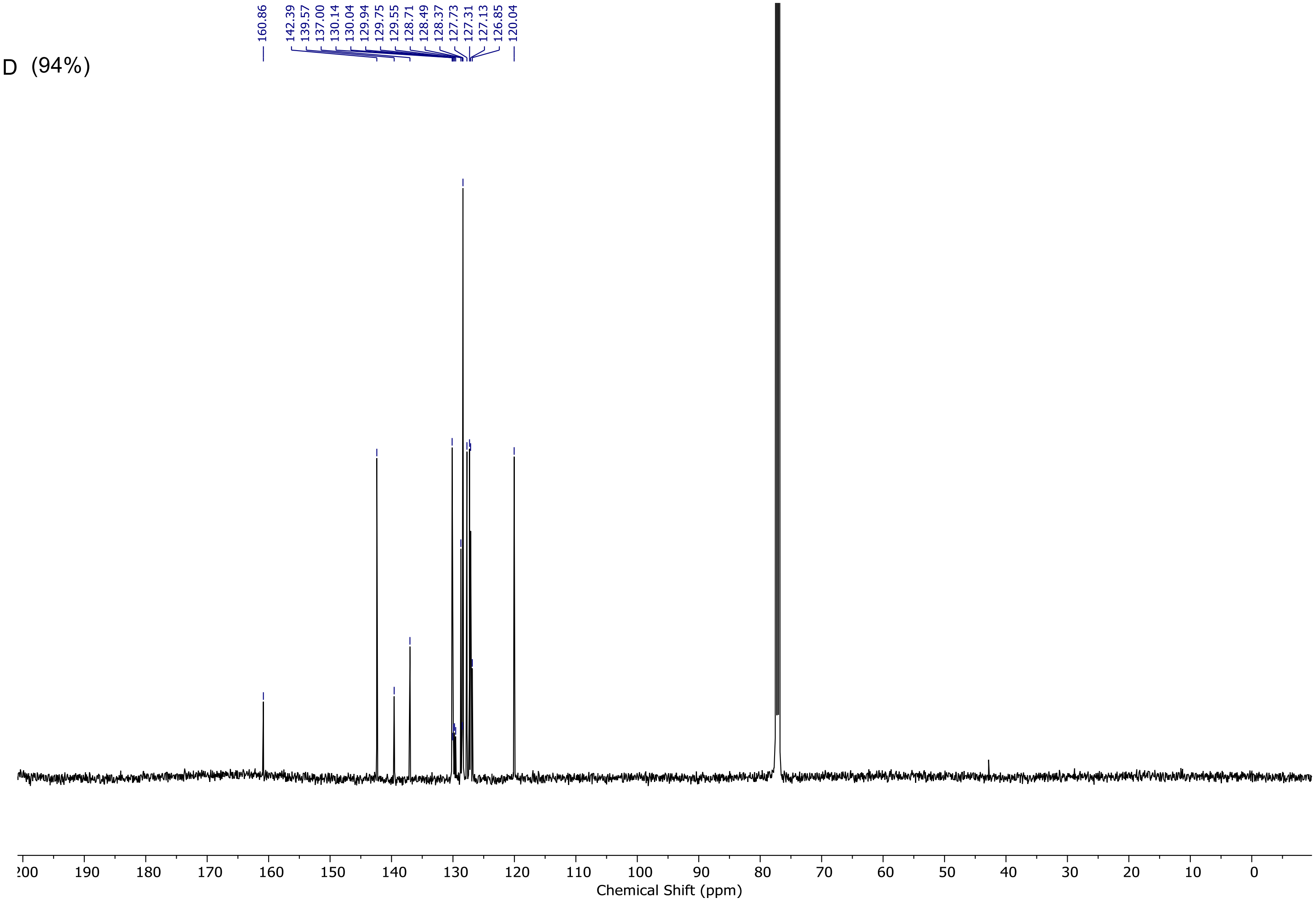

^{1}H NMR (400 MHz, $CDCl_3$) of benzoic-2,6-d_2 acid-d (d_2-22)

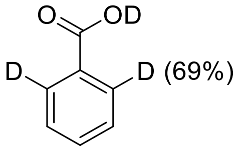

d_2-22

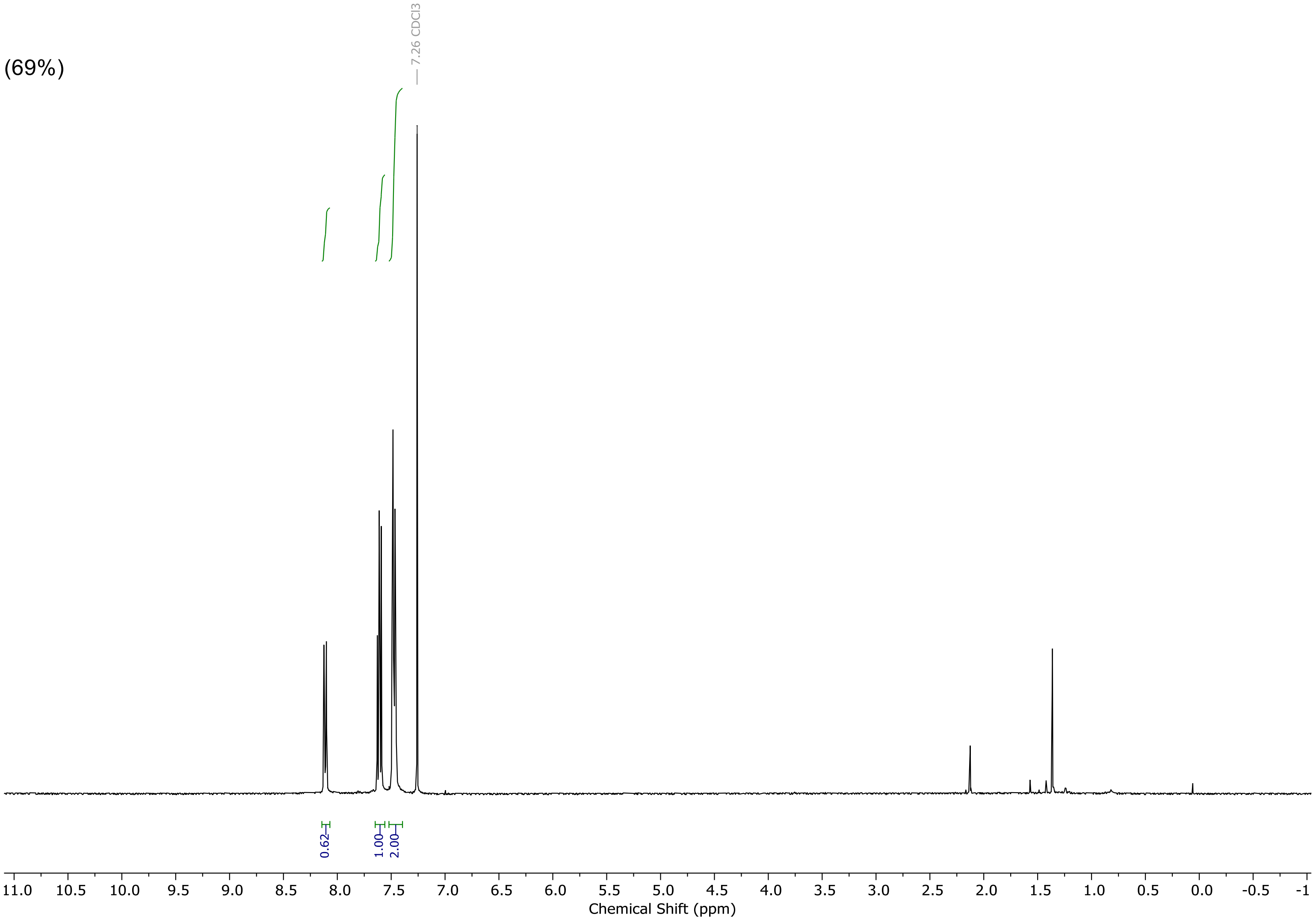

^{2}D NMR (77 MHz, $CHCl_3$) of benzoic-2,6-d_2 acid-d (d_2-22)

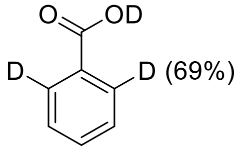

d_2-22

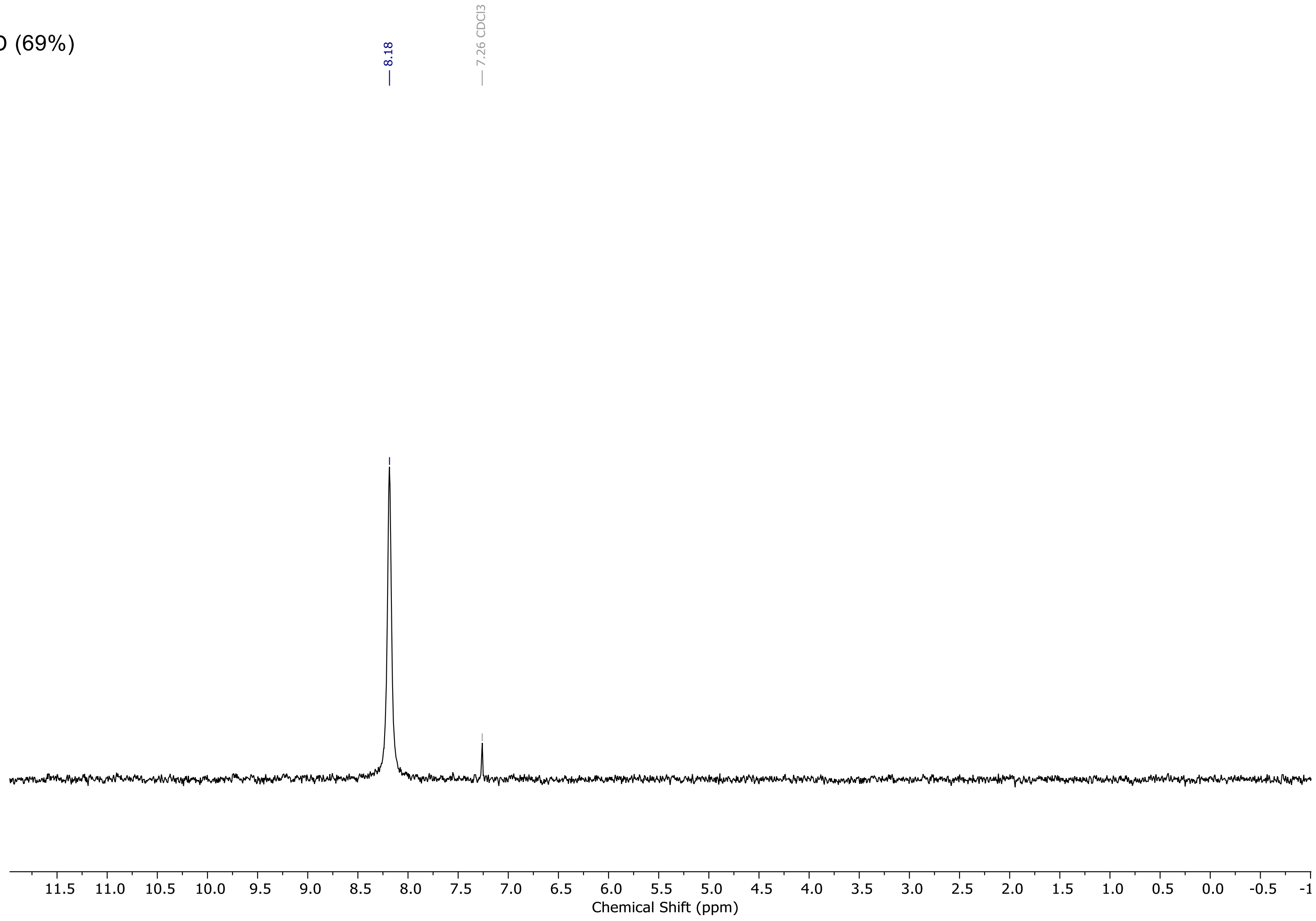

^{13}C NMR (101 MHz, $CDCl_3$) of benzoic-2,6-d_2 acid-d (d_2-22)

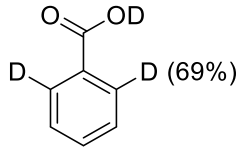

d_2-22

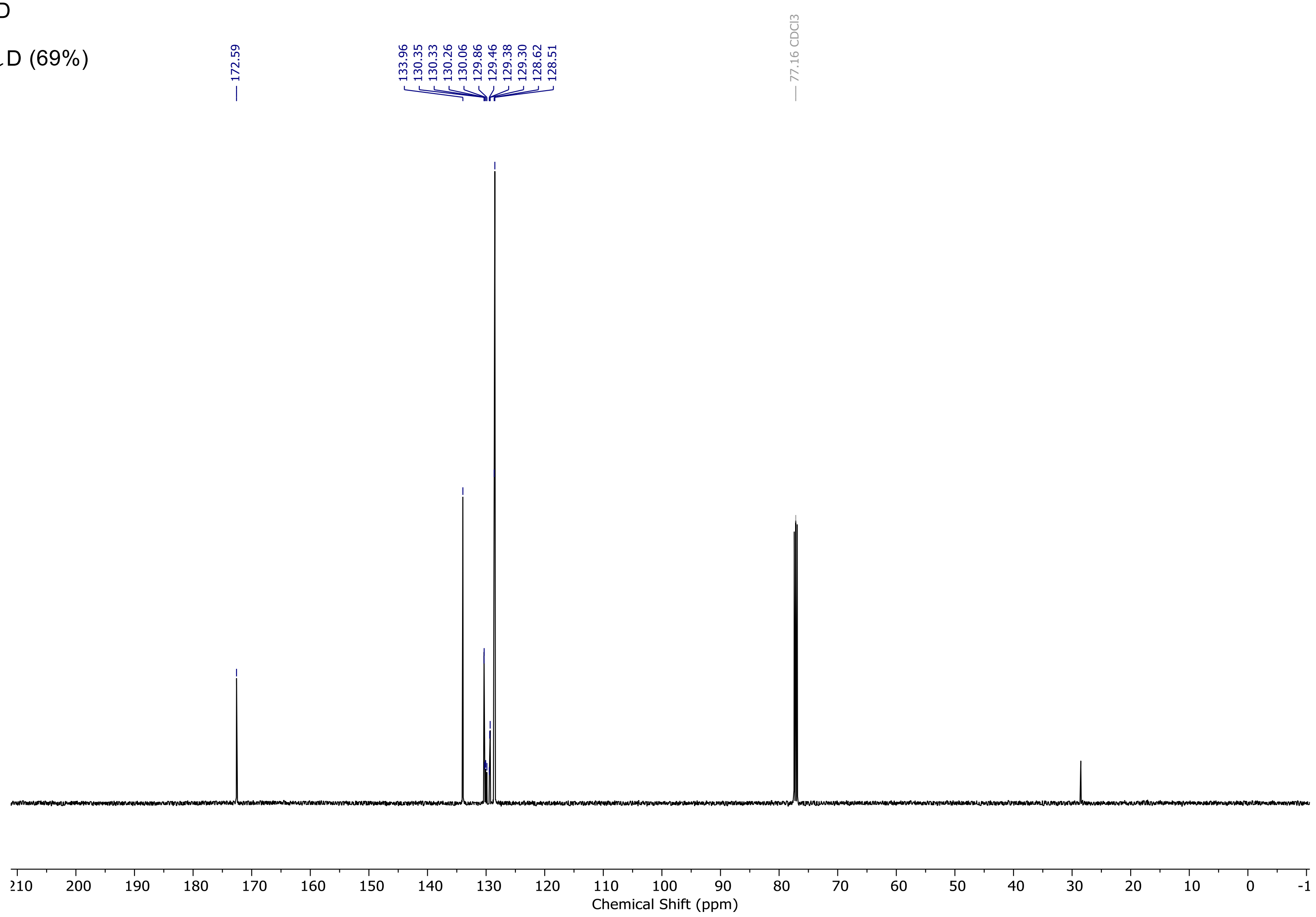

^{1}H NMR (500 MHz, $CDCl_3$) of 2-(phenyl-2,6-d2)benzo[d]thiazole (d_2-8p)

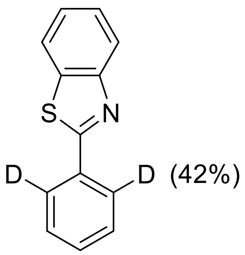

d_2-8p

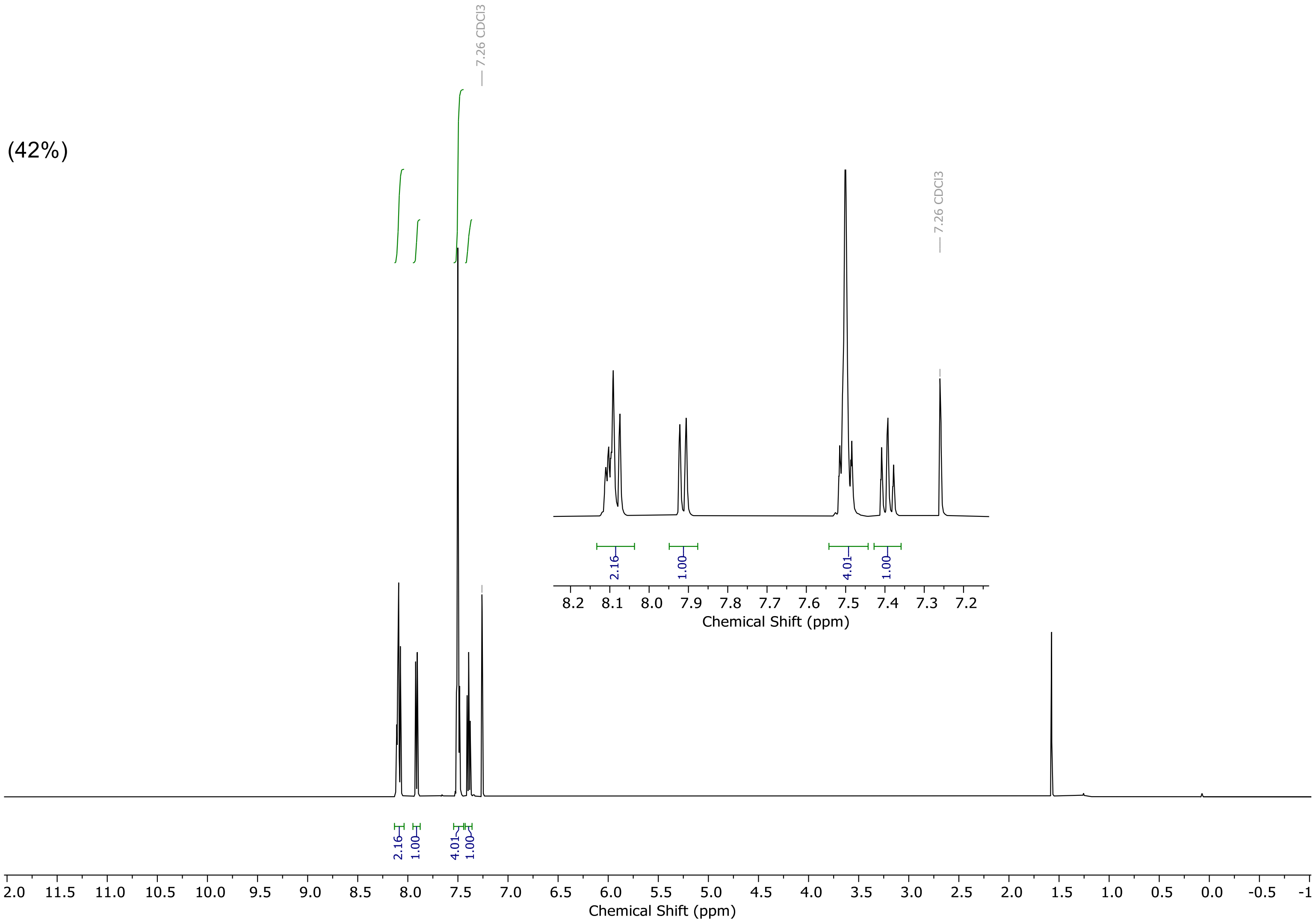

^{2}D NMR (77 MHz, $CHCl_3$) of 2-(phenyl-2,6-d2)benzo[d]thiazole (d_2-8p)

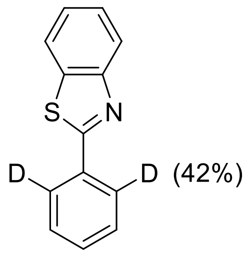

d_2-8p

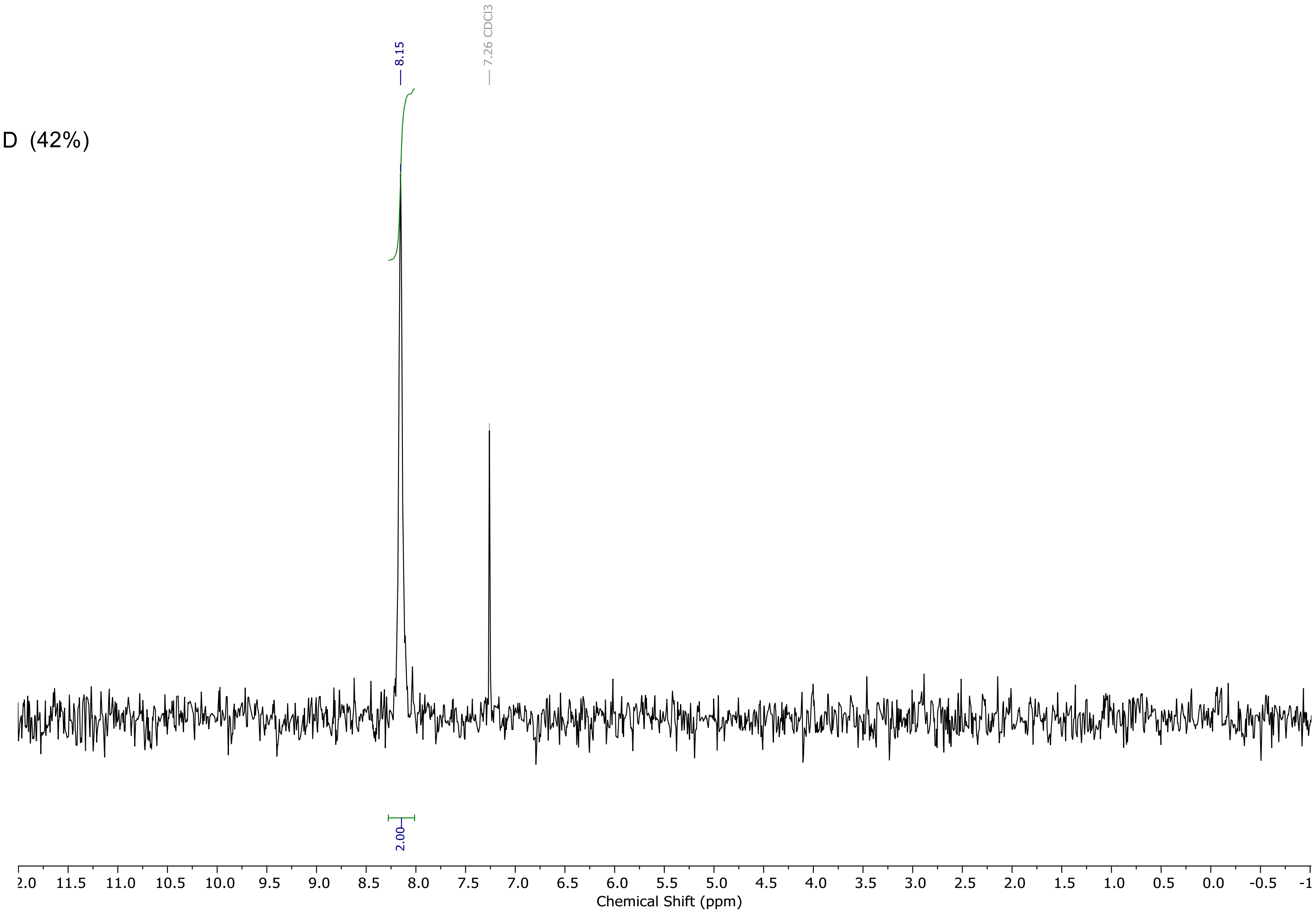

^{13}C NMR (126 MHz, CHCl$_3$) of 2-(phenyl-2,6-d2)benzo[d]thiazole (d$_2$-8p)

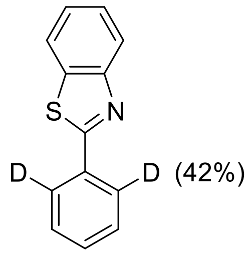

d$_2$-8p

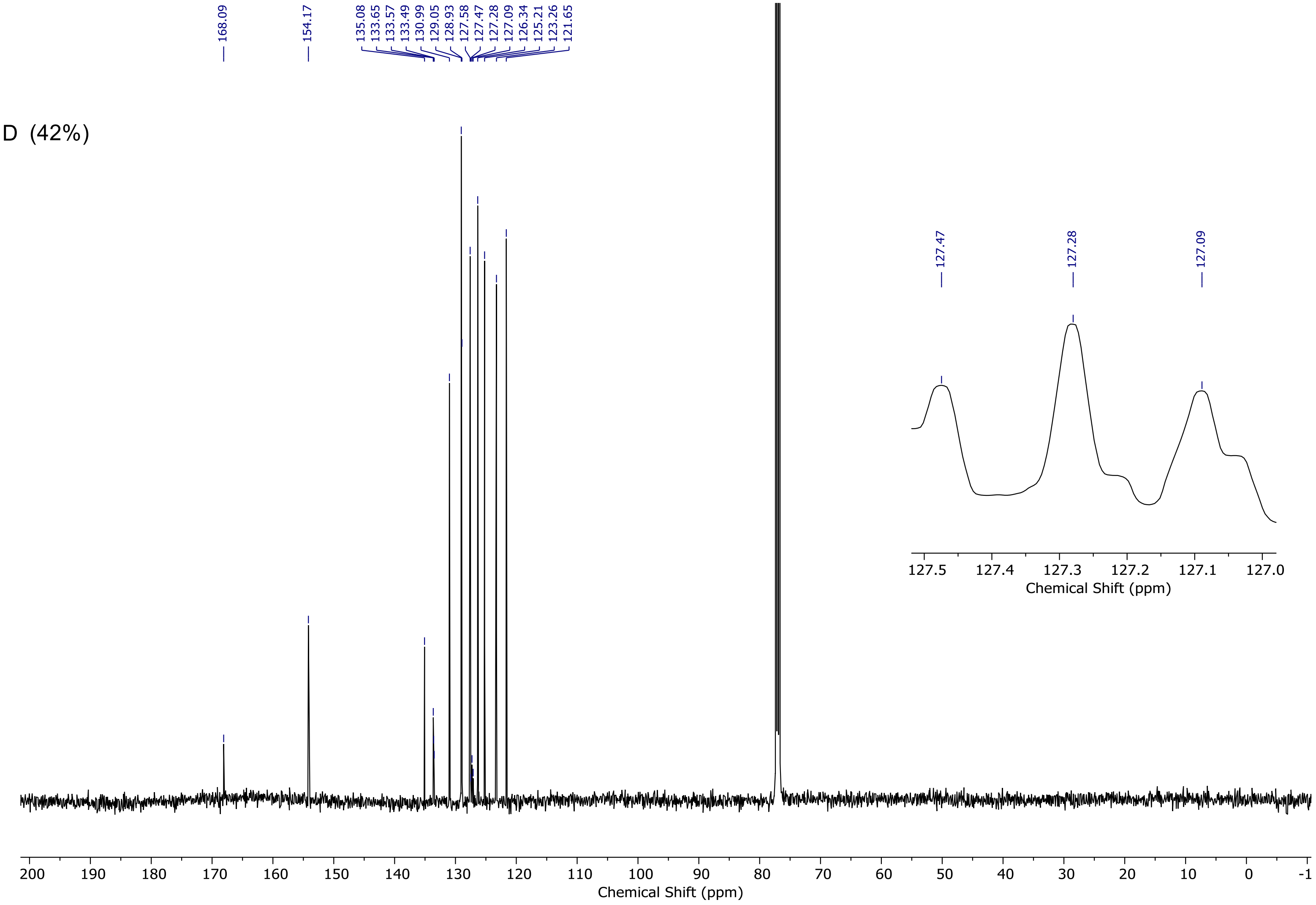

^{1}H NMR (400 MHz, $CDCl_3$) of 1-(phenyl-2,6-d_2)isoquinoline-3-d (d_2-8g)

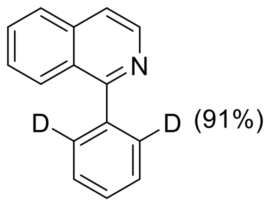

d_2-8g

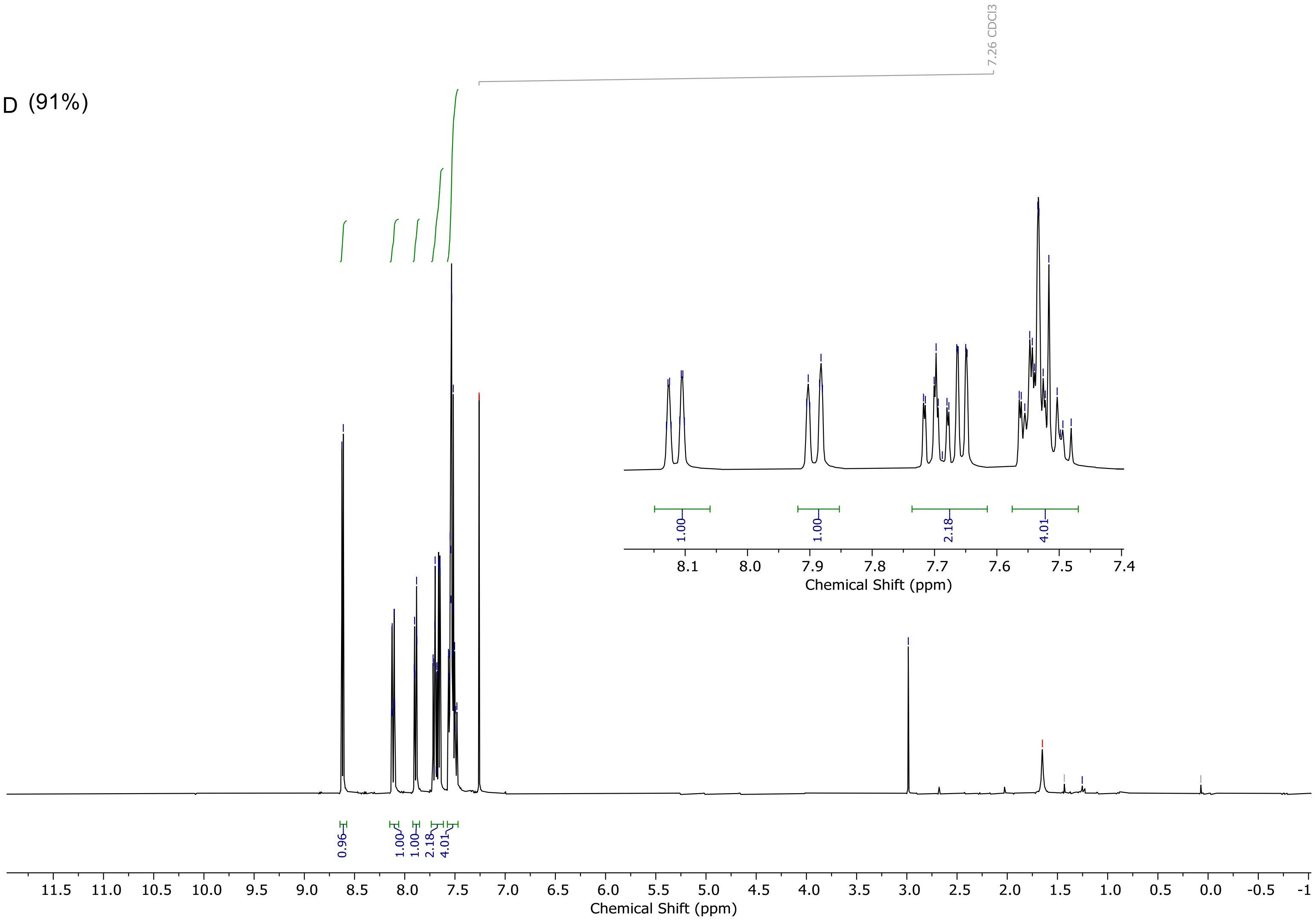

^{2}D NMR (77 MHz, $CHCl_3$) of 1-(phenyl-2,6-d_2)isoquinoline-3-d (d_2-8g)

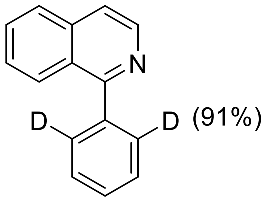

d_2-8g

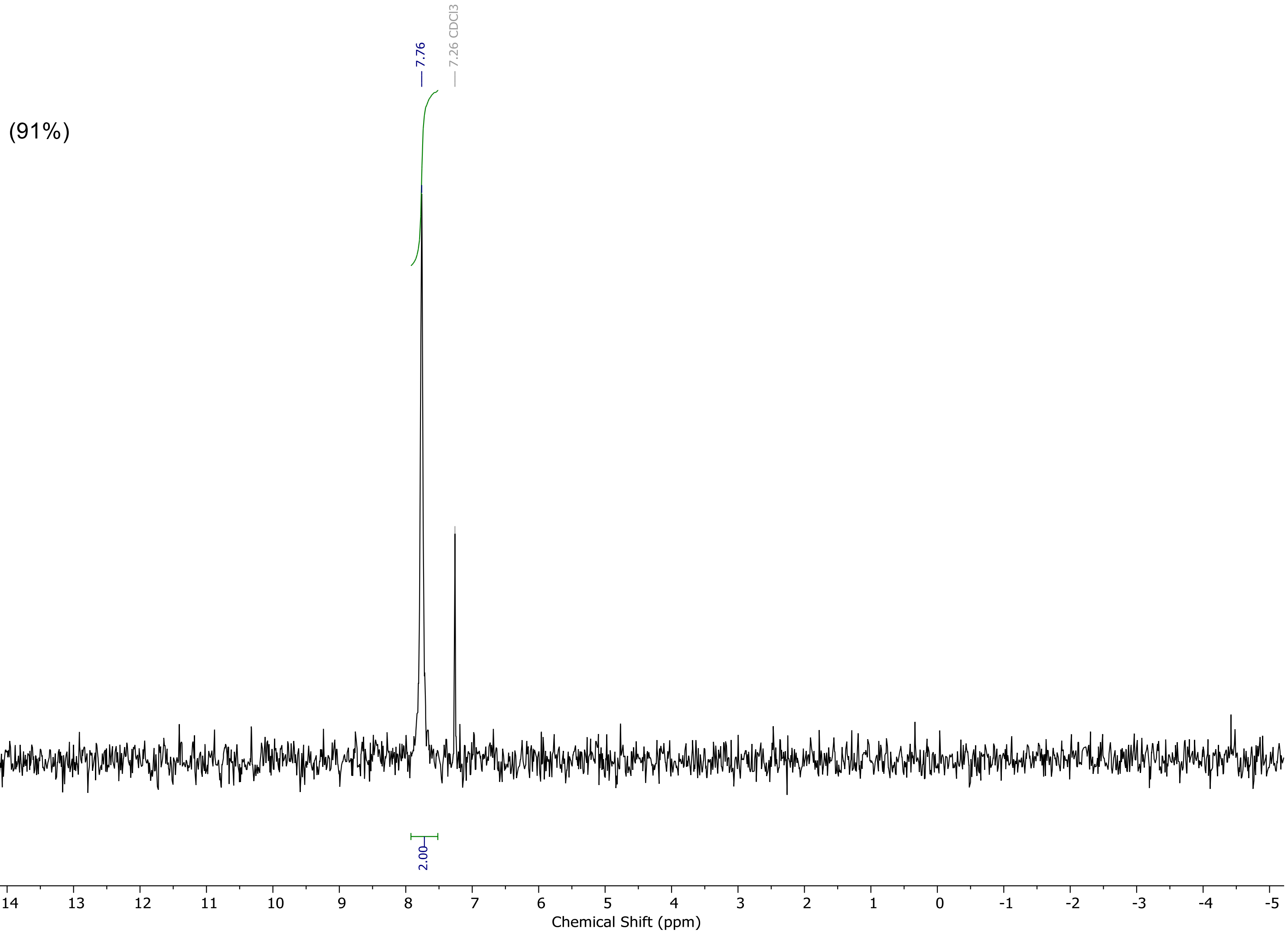

^{13}C NMR (126 MHz, $CDCl_3$) of 1-(phenyl-2,6-d_2)isoquinoline-3-d (d_2-8g)

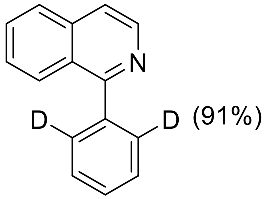

d_2-8g

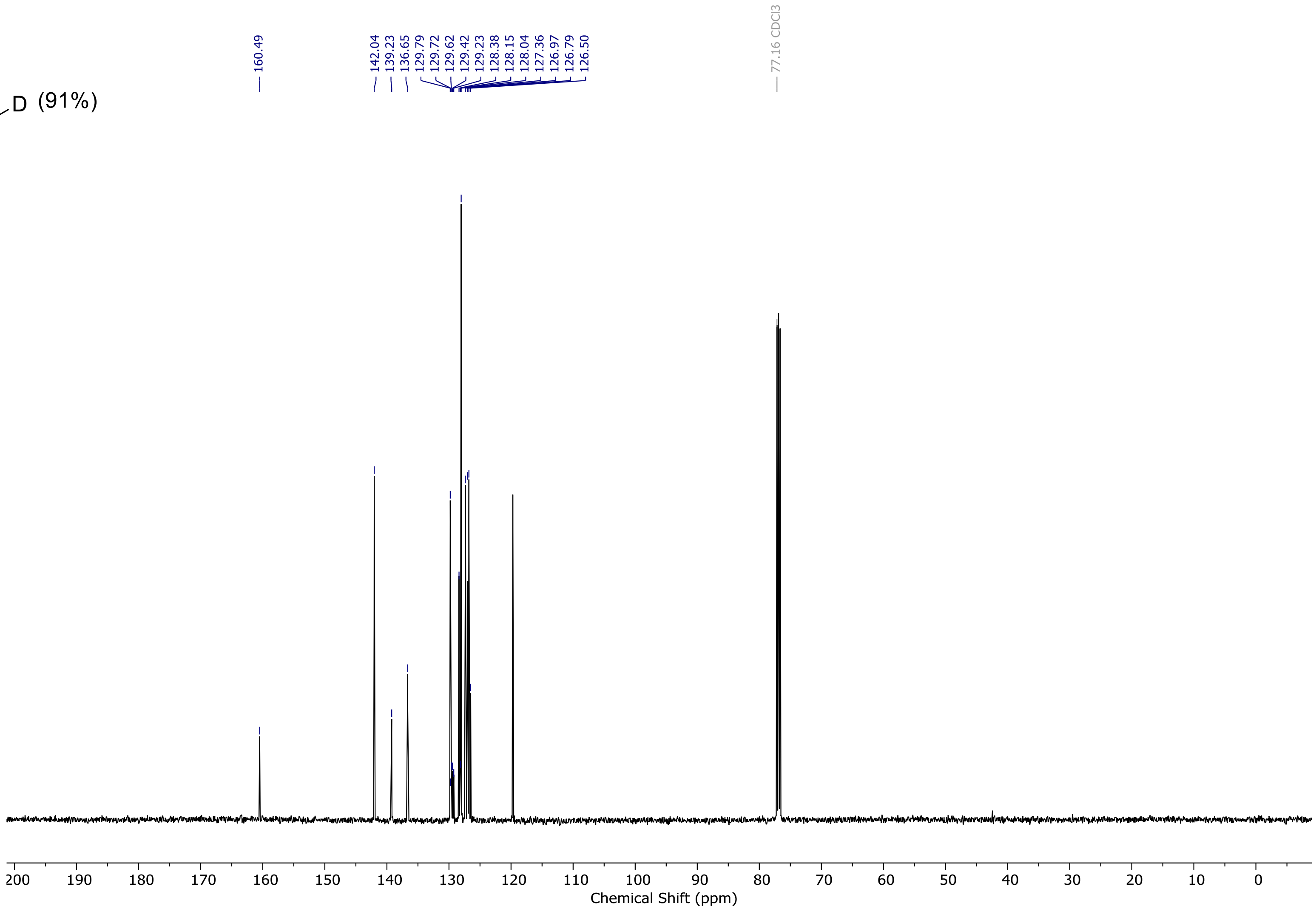

^{1}H NMR (500 MHz, $CDCl_3$) of (E)-2-methoxy-4-(prop-1-en-1-yl)phenol 24

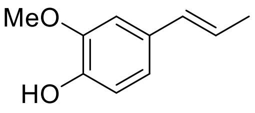

24

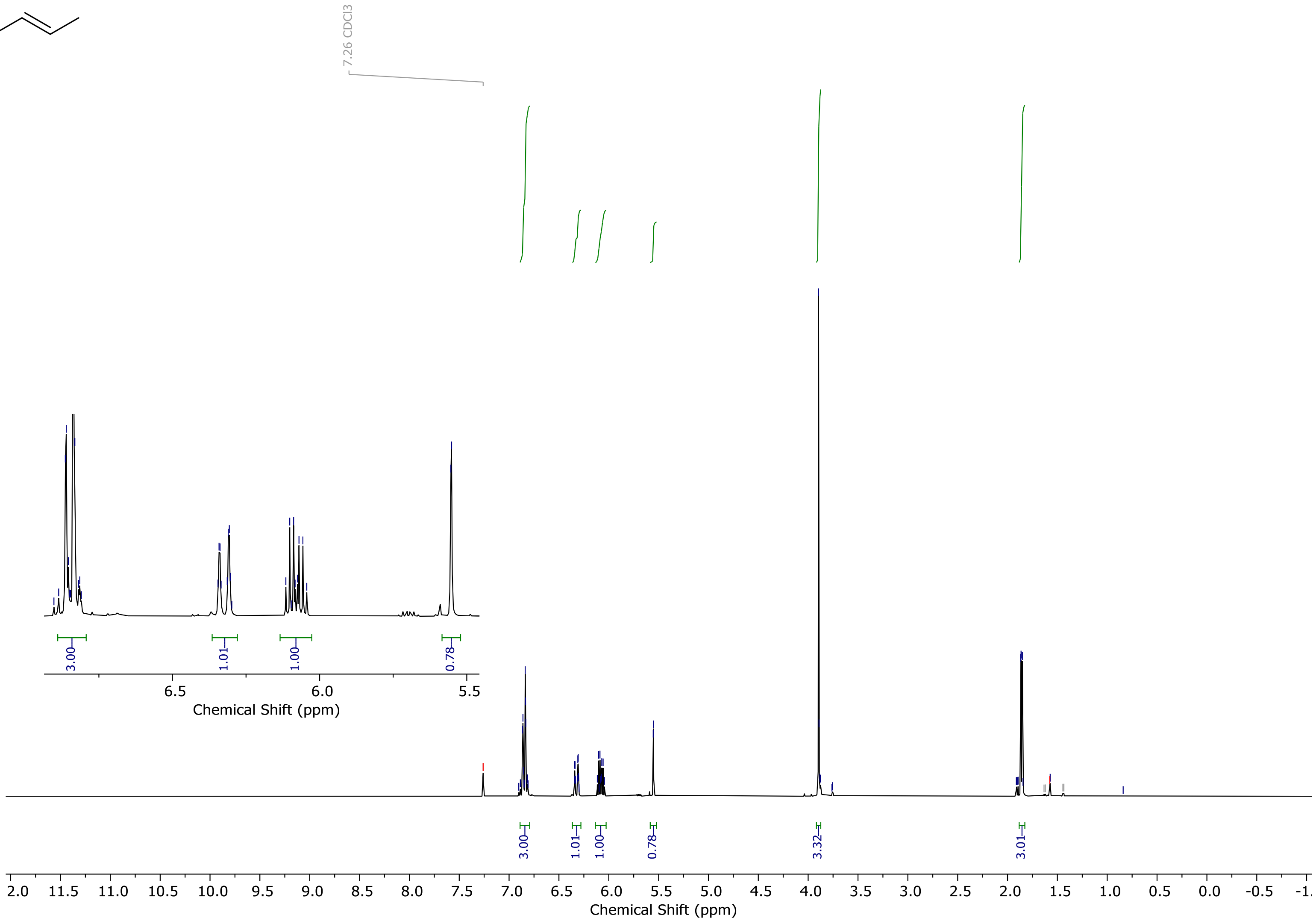

^{13}C NMR (126 MHz, CDCl$_3$) of (E)-2-methoxy-4-(prop-1-en-1-yl)phenol 24

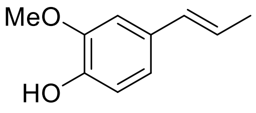

24

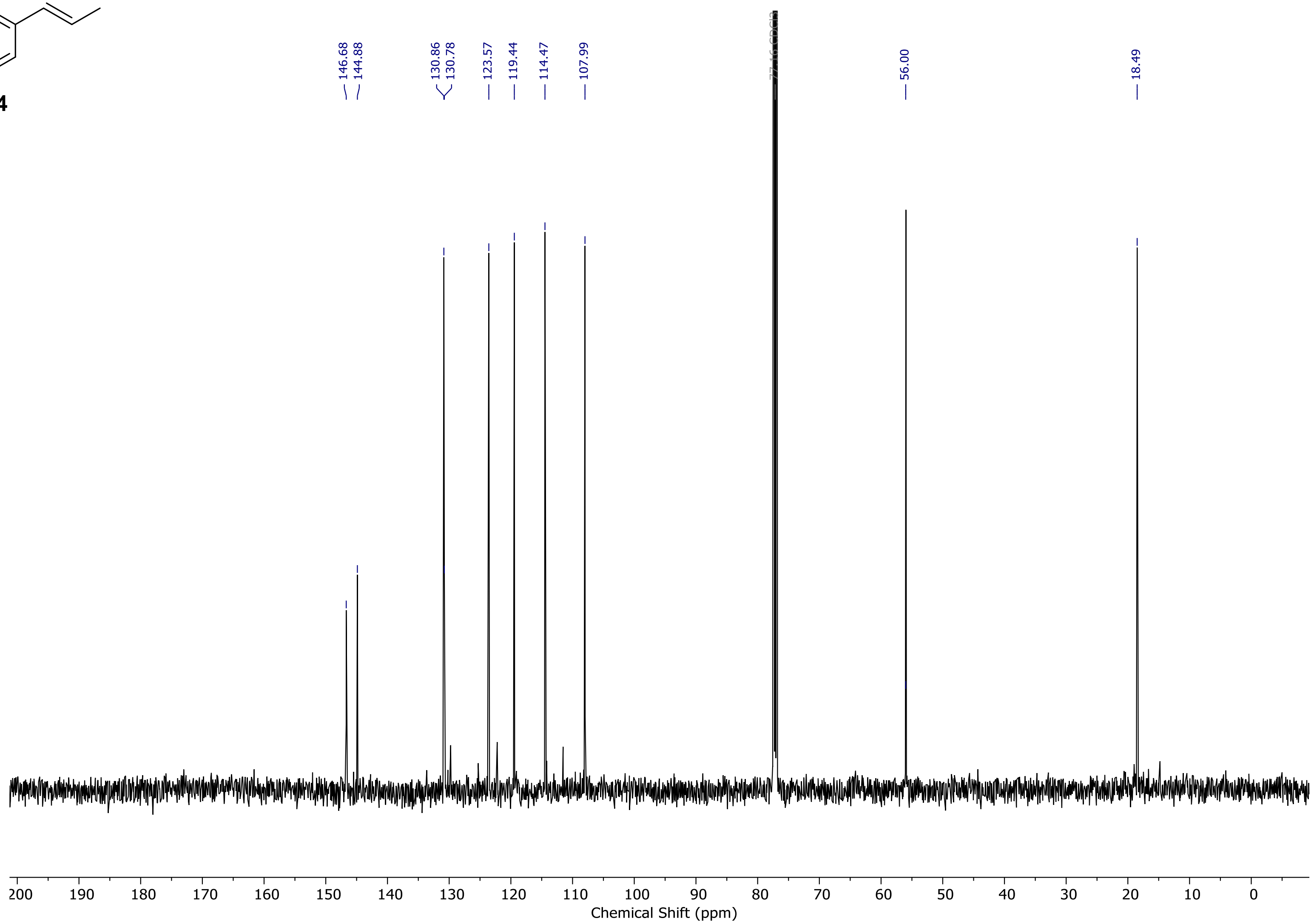

^{1}H NMR (500 MHz, $CDCl_3$) of (*E*)-But-1-en-3-yne-1,4-diyldibenzene 26

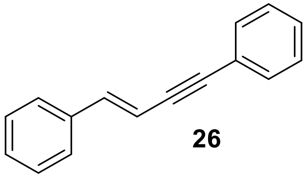

26

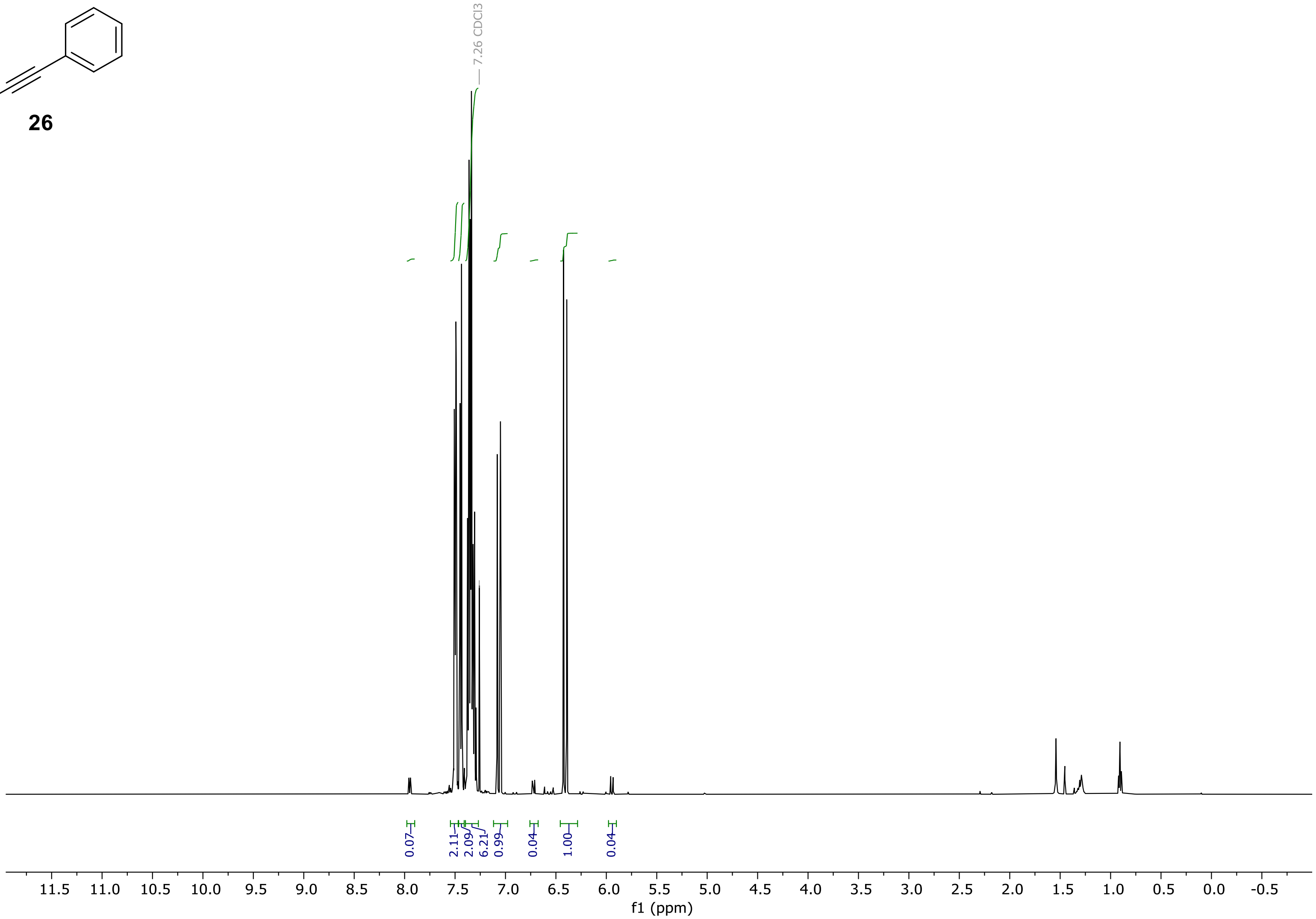

^{13}C NMR (126 MHz, CDCl$_3$) of (*E*)-But-1-en-3-yne-1,4-diyldibenzene 26

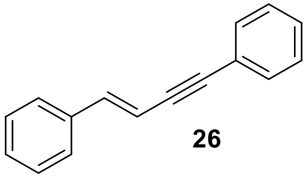

26

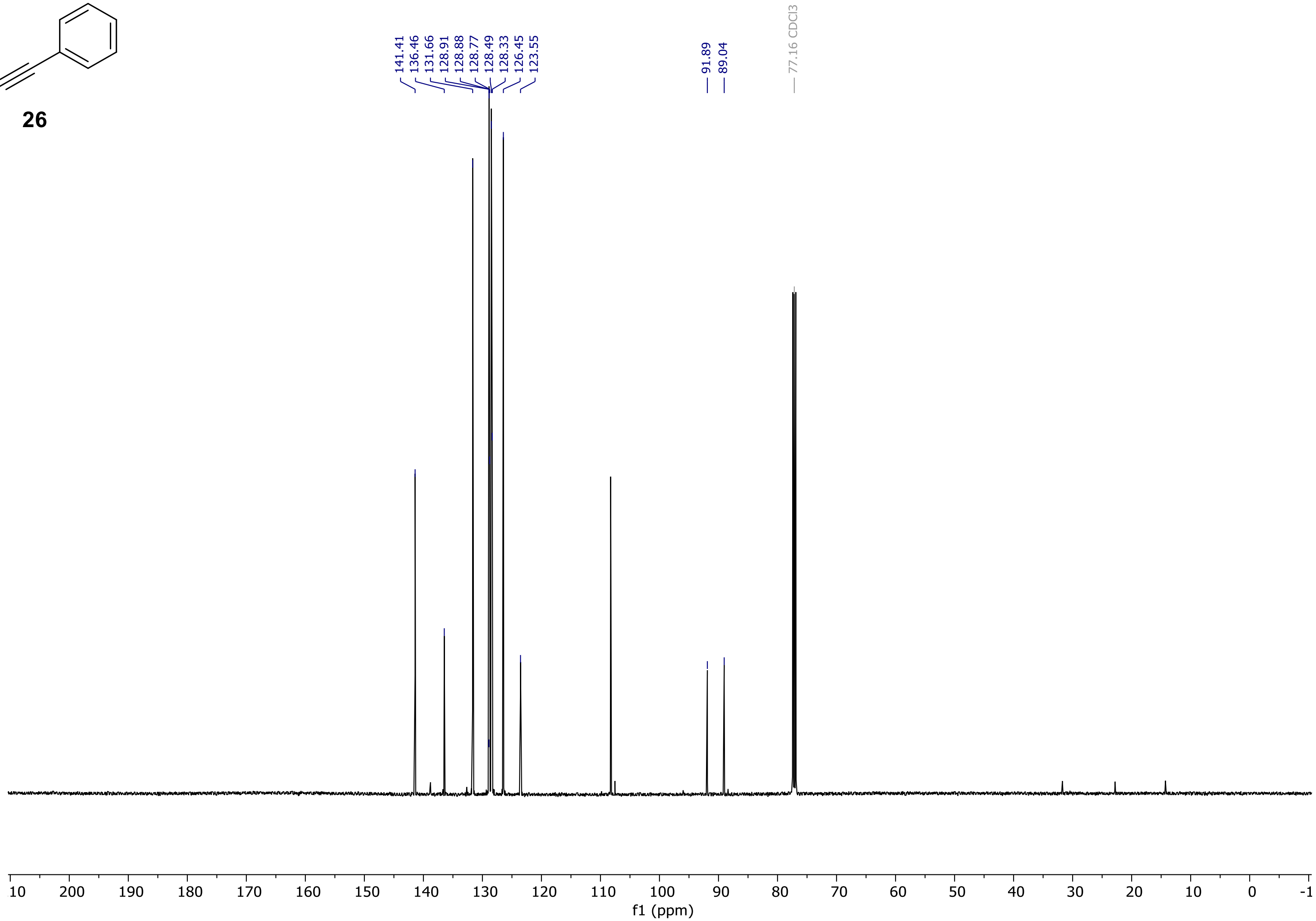

^{1}H NMR (500 MHz, $CDCl_3$) of Adamantan-1-ol 28a

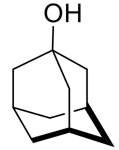

28a

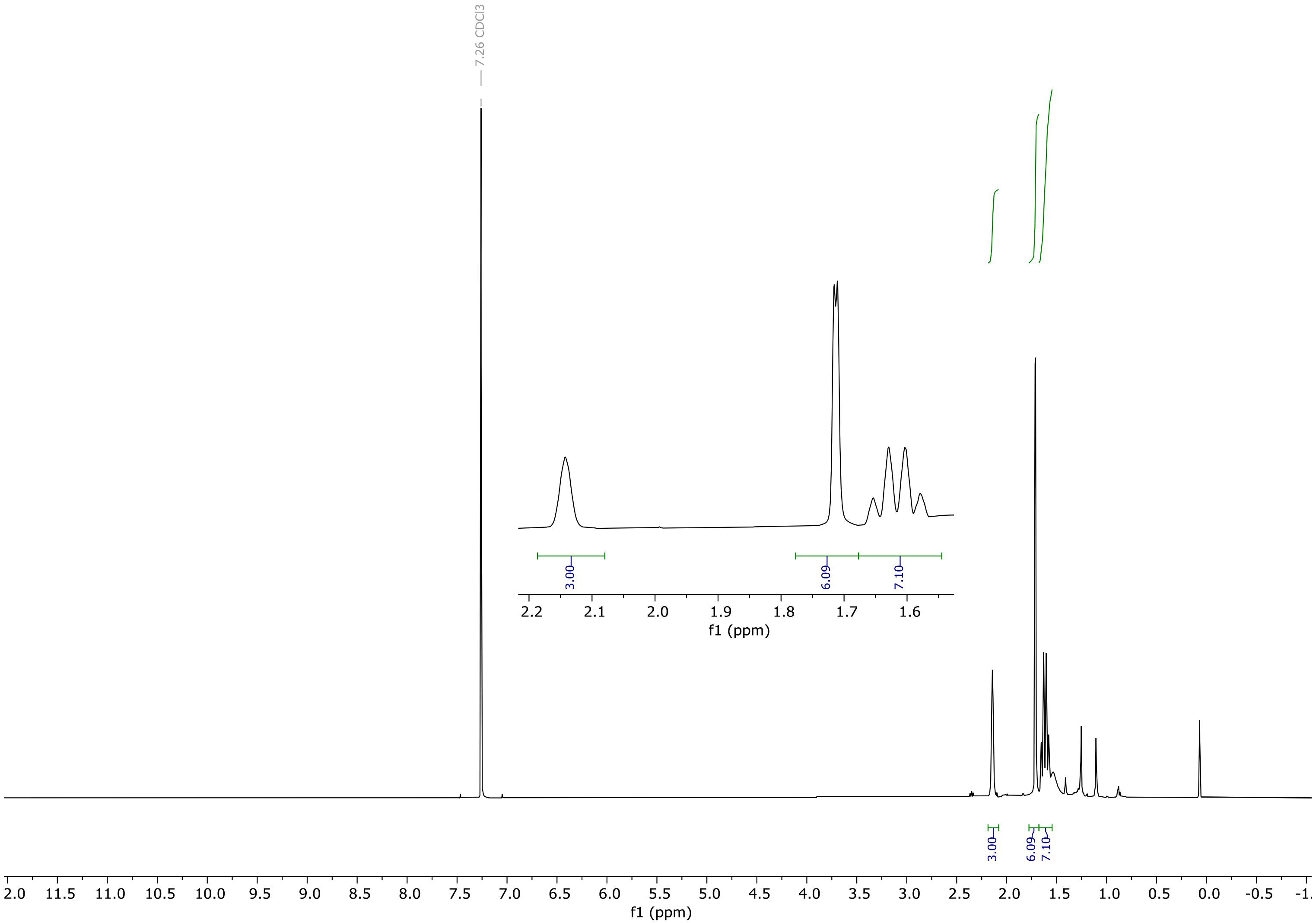

^{13}C NMR (101 MHz, CDCl$_3$) of Adamantan-1-ol 28a

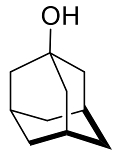

28a

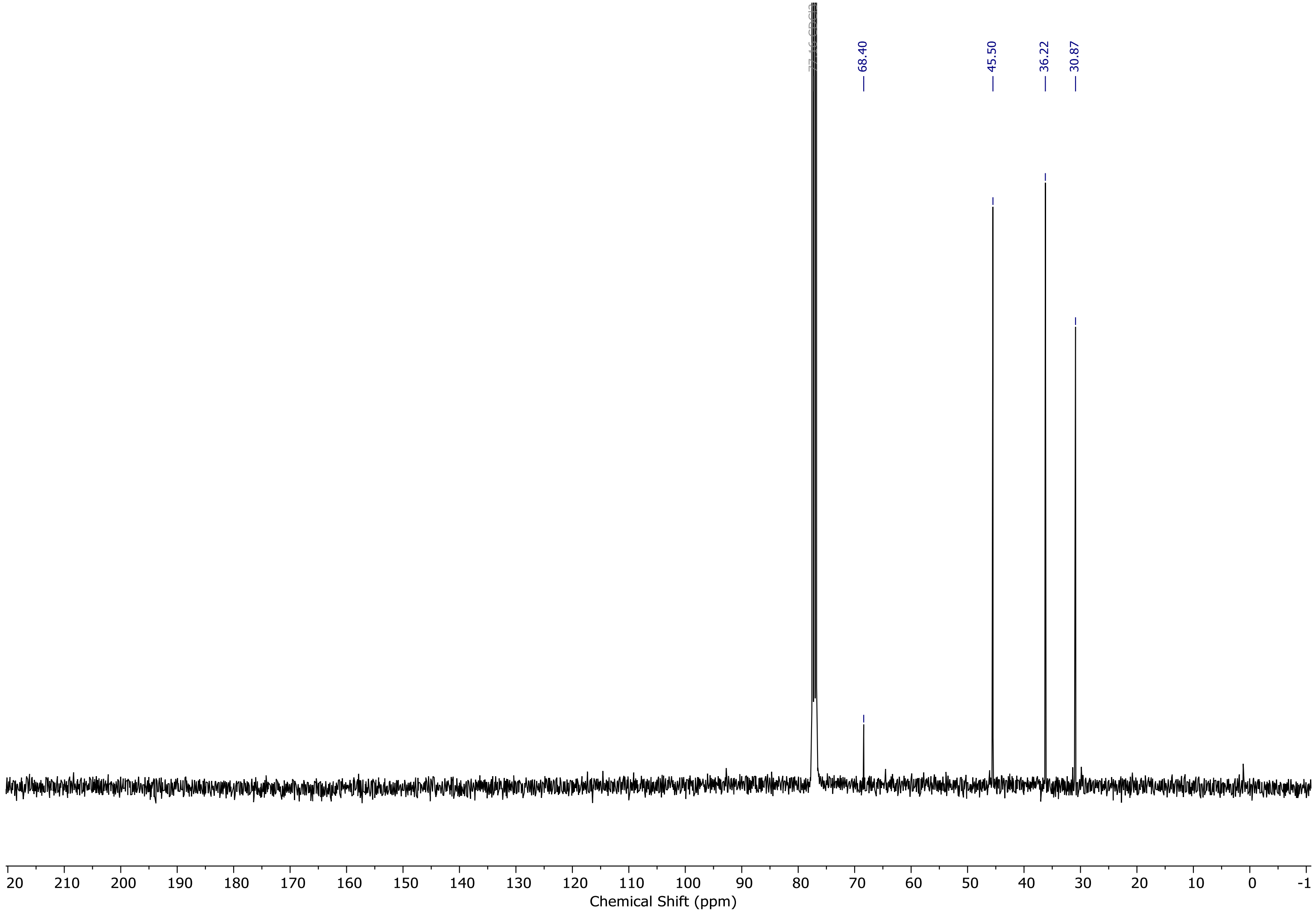

^{1}H NMR (500 MHz, d_4-MeOD) of adamantane-1,3-diol 28b

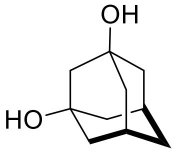

28b

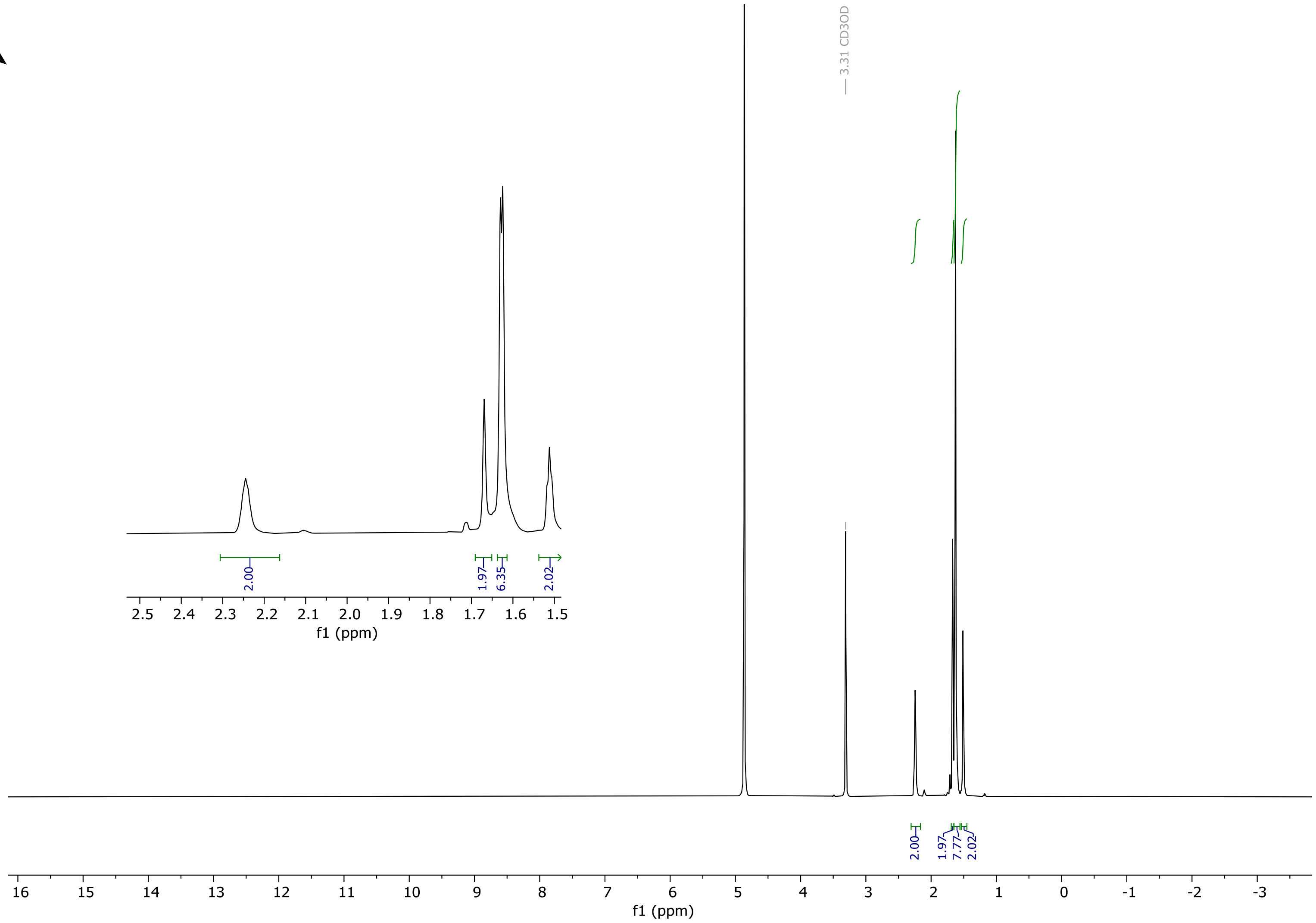

^{13}C NMR (126 MHz, d_4-MeOD) of adamantane-1,3-diol 28b

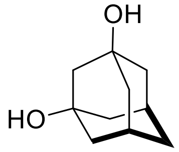

28b

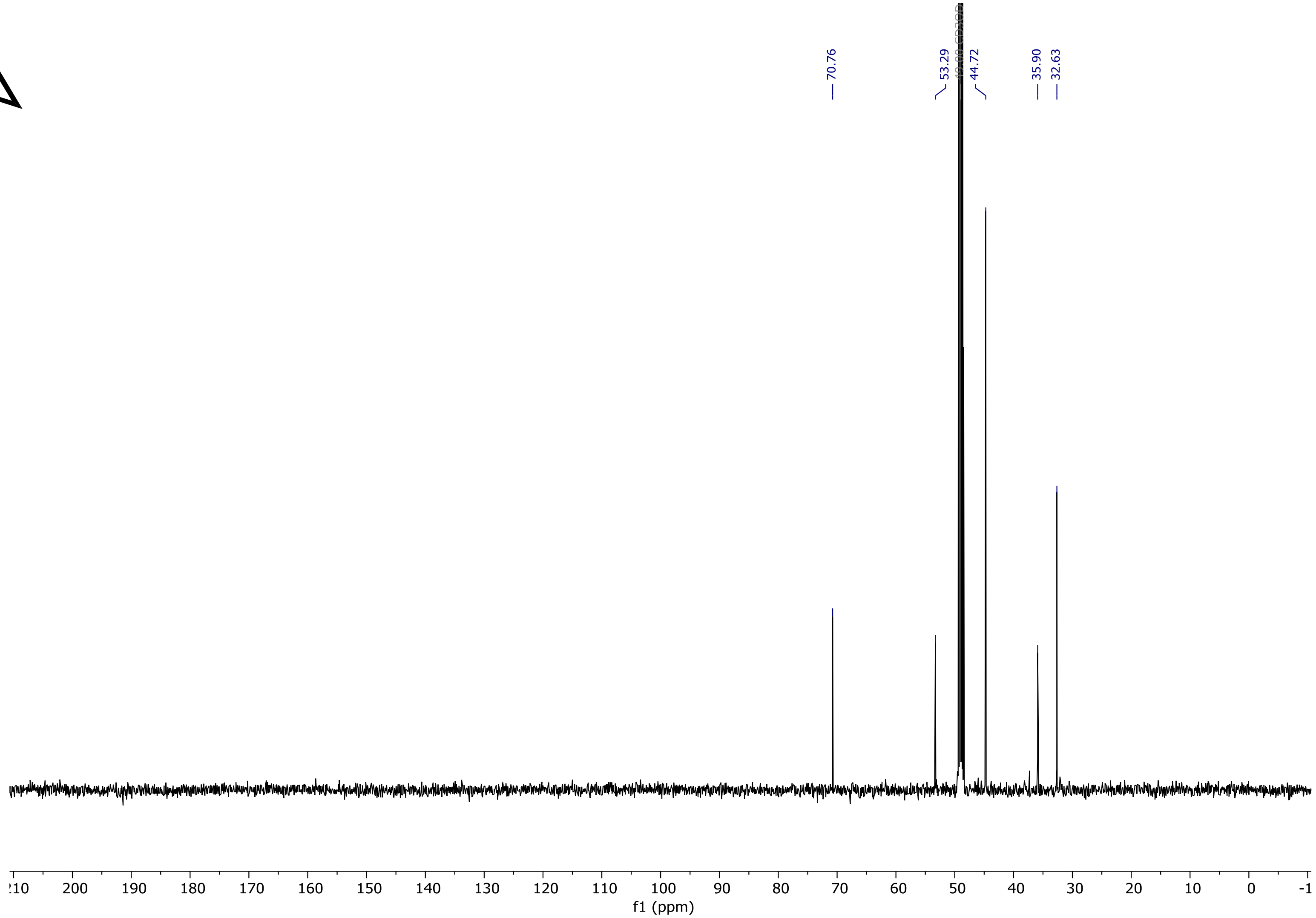

^{1}H NMR (500 MHz, $CDCl_3$) of Starting Material 29

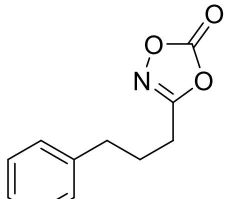

29

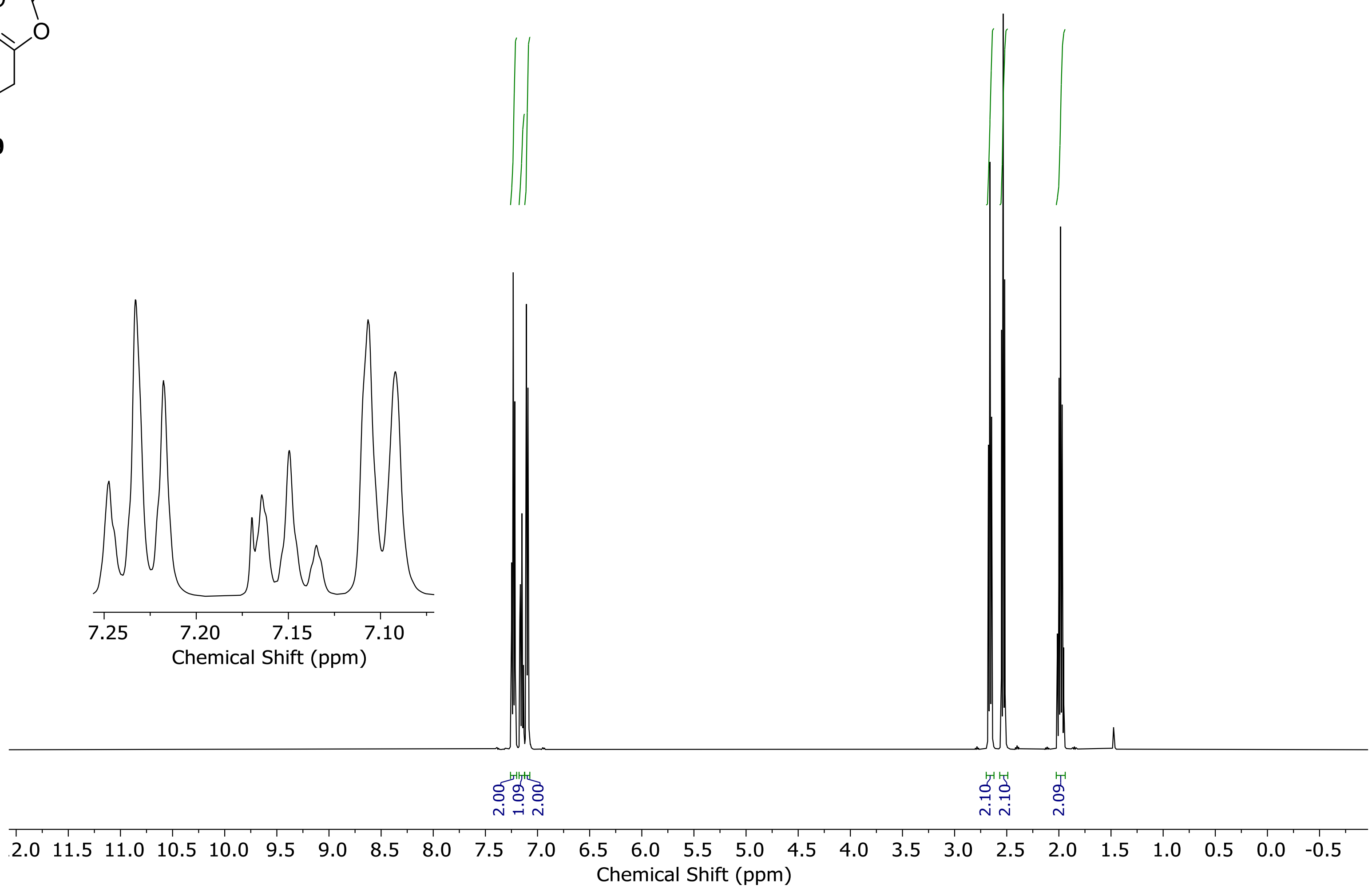

^{13}C NMR (126 MHz, $CDCl_3$) of Starting Material 29

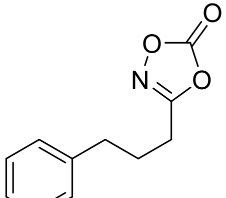

29

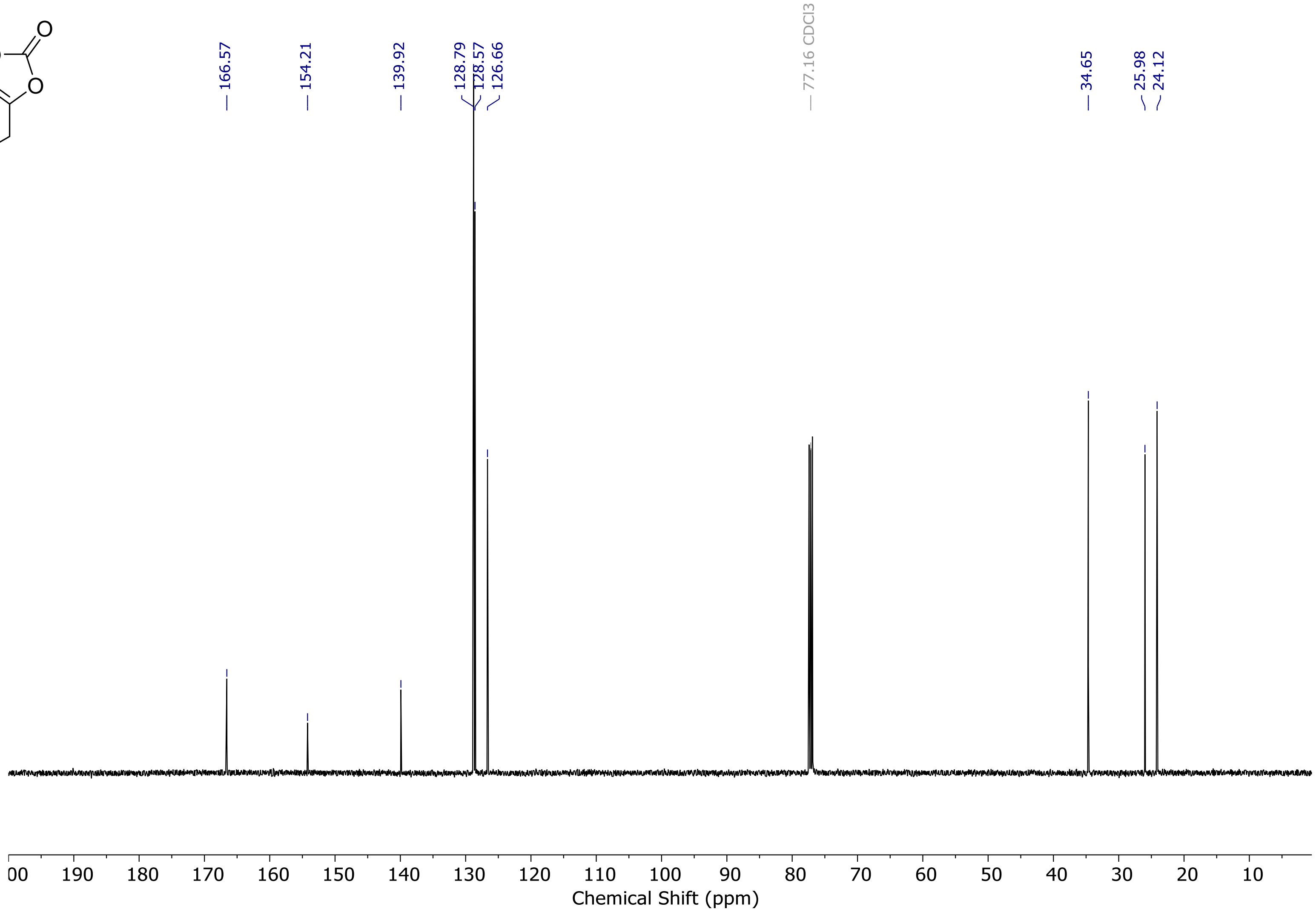

^{1}H NMR (500 MHz, $CDCl_3$) of (3-isocyanatopropyl)benzene 30

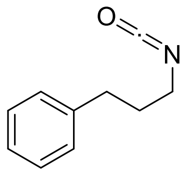

30

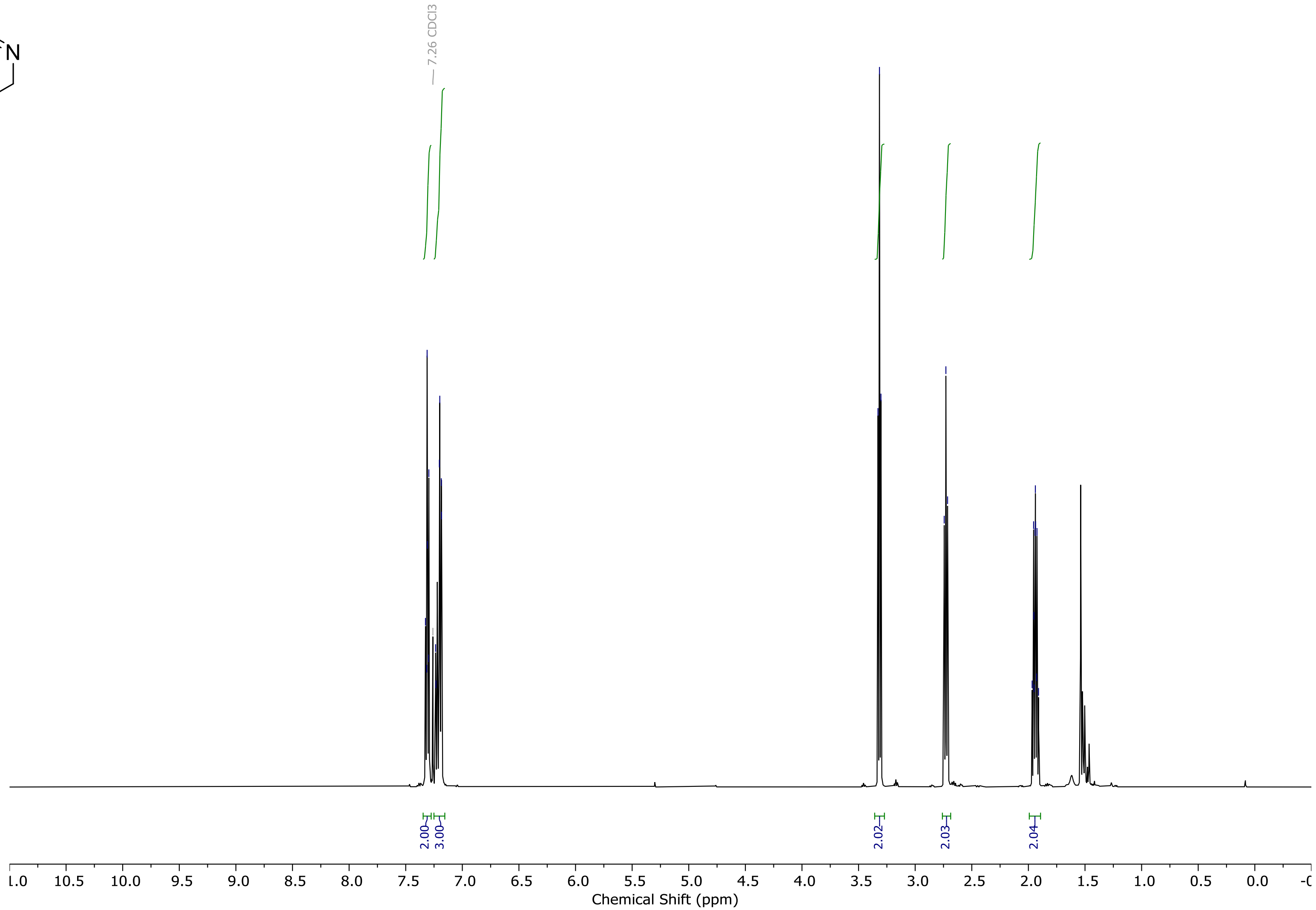

^{13}C NMR (126 MHz, $CDCl_3$) of (3-isocyanatopropyl)benzene 30

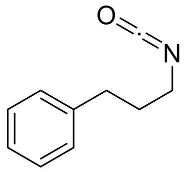

30

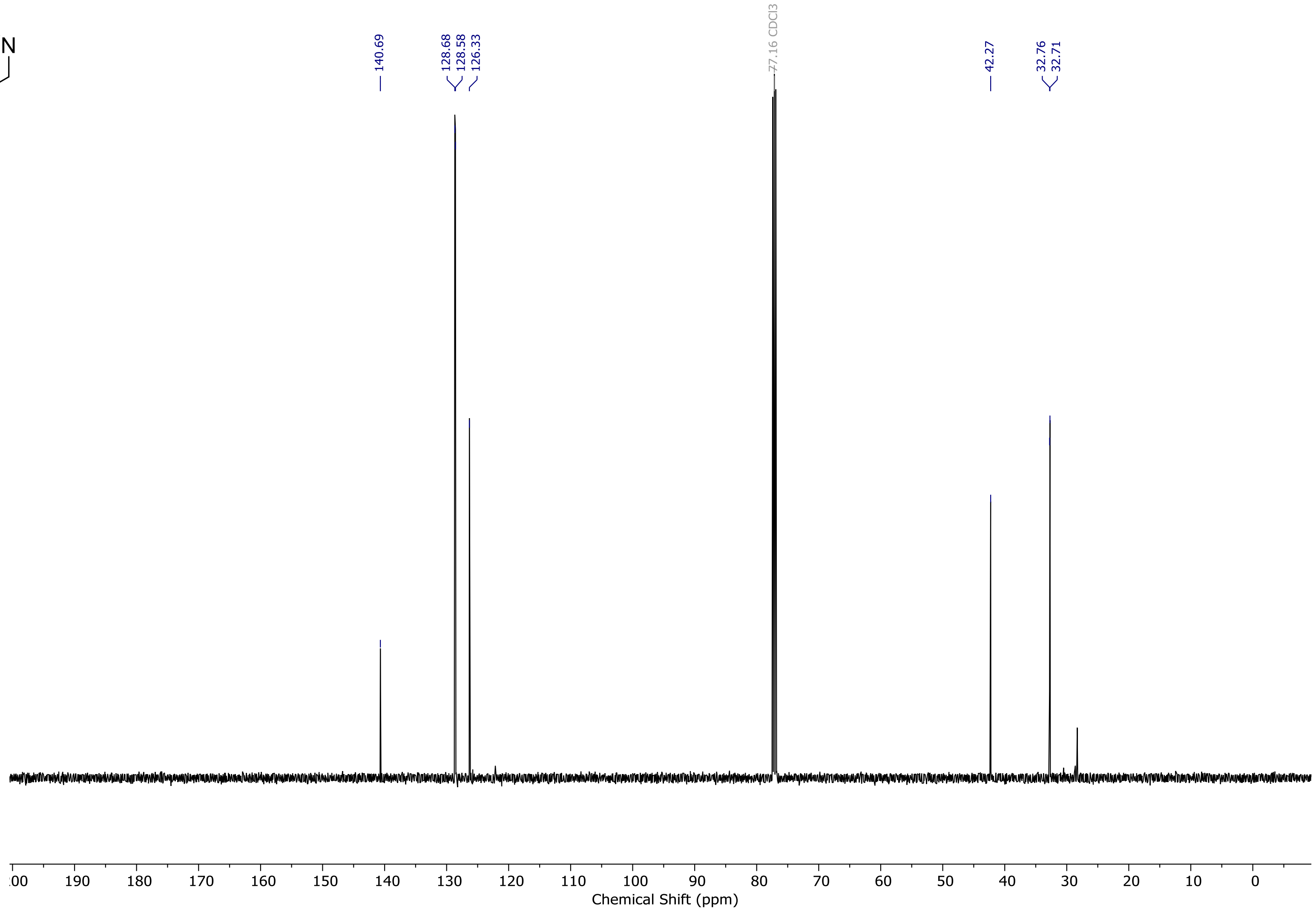

^{1}H NMR (500 MHz, $CDCl_3$) of benzaldehyde 32a

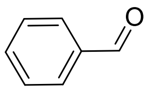

32a

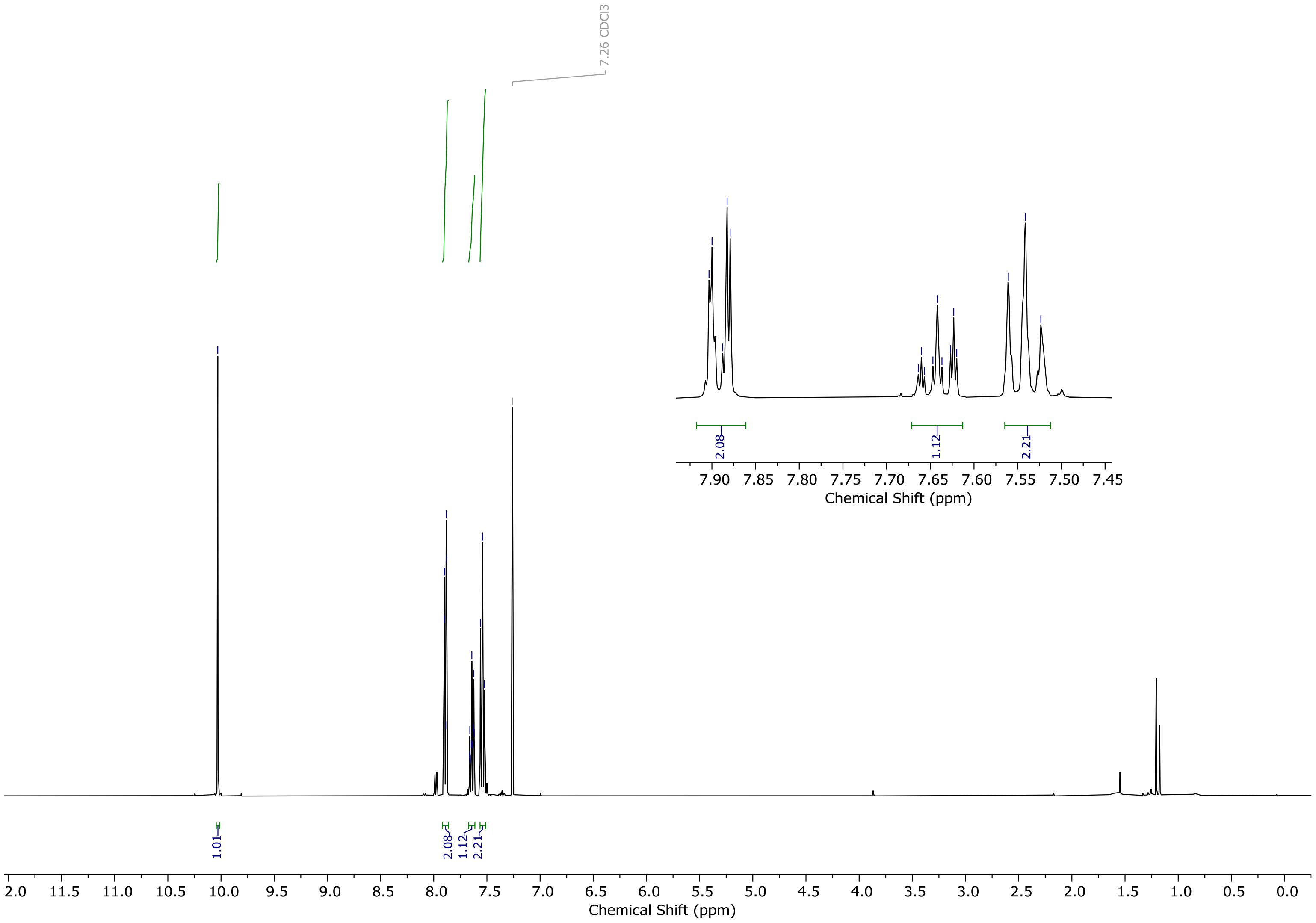

^{13}C NMR (101 MHz, $CDCl_3$) of benzaldehyde 32a

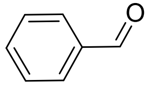

32a

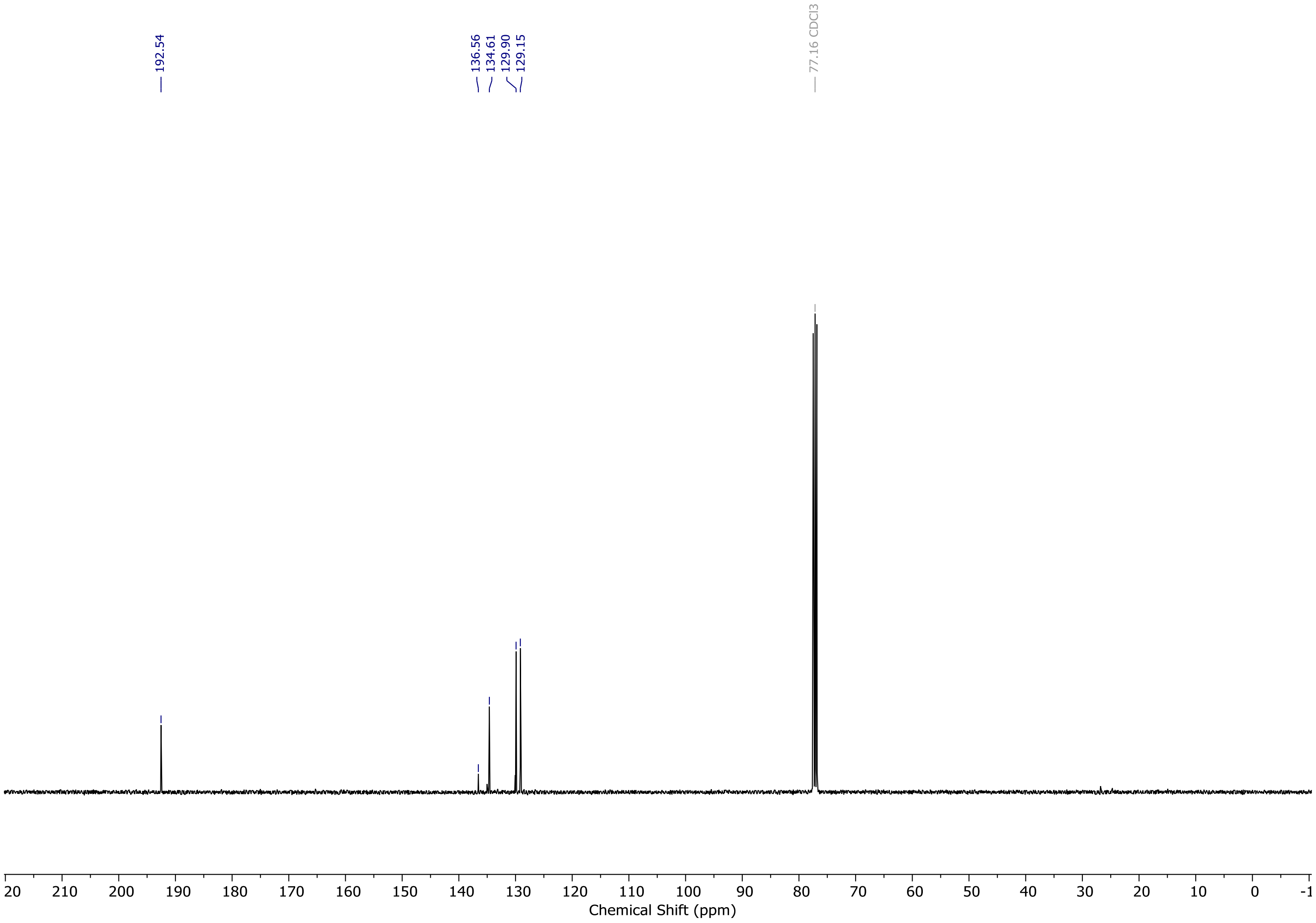

^{1}H NMR (500 MHz, $CDCl_3$) of cyclopentane-1,3-dicarbaldehyde 32b

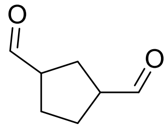

32b

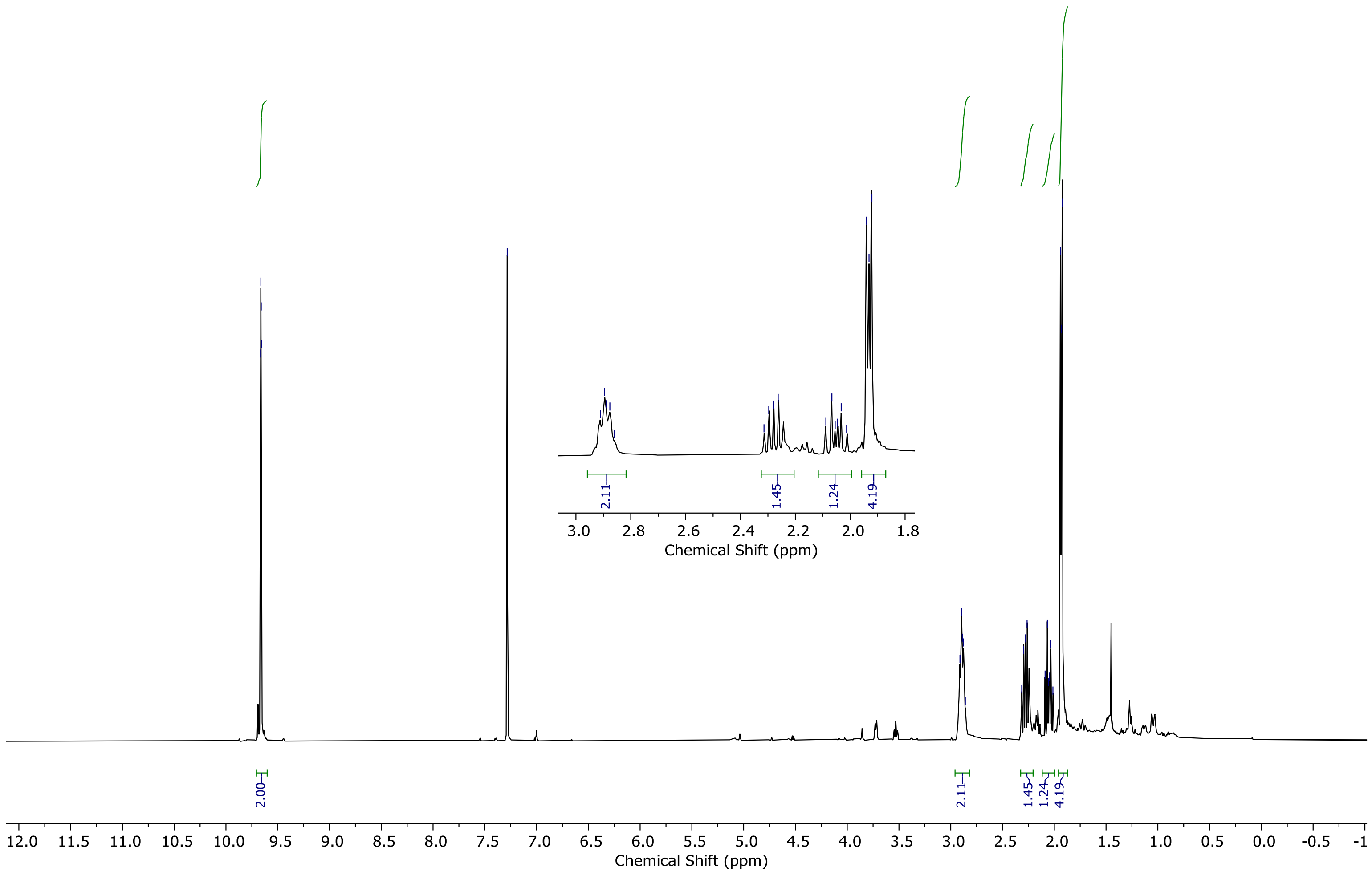

^{13}C NMR (101 MHz, $CDCl_3$) of cyclopentane-1,3-dicarbaldehyde 32b

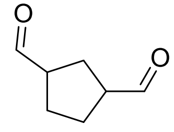

32b

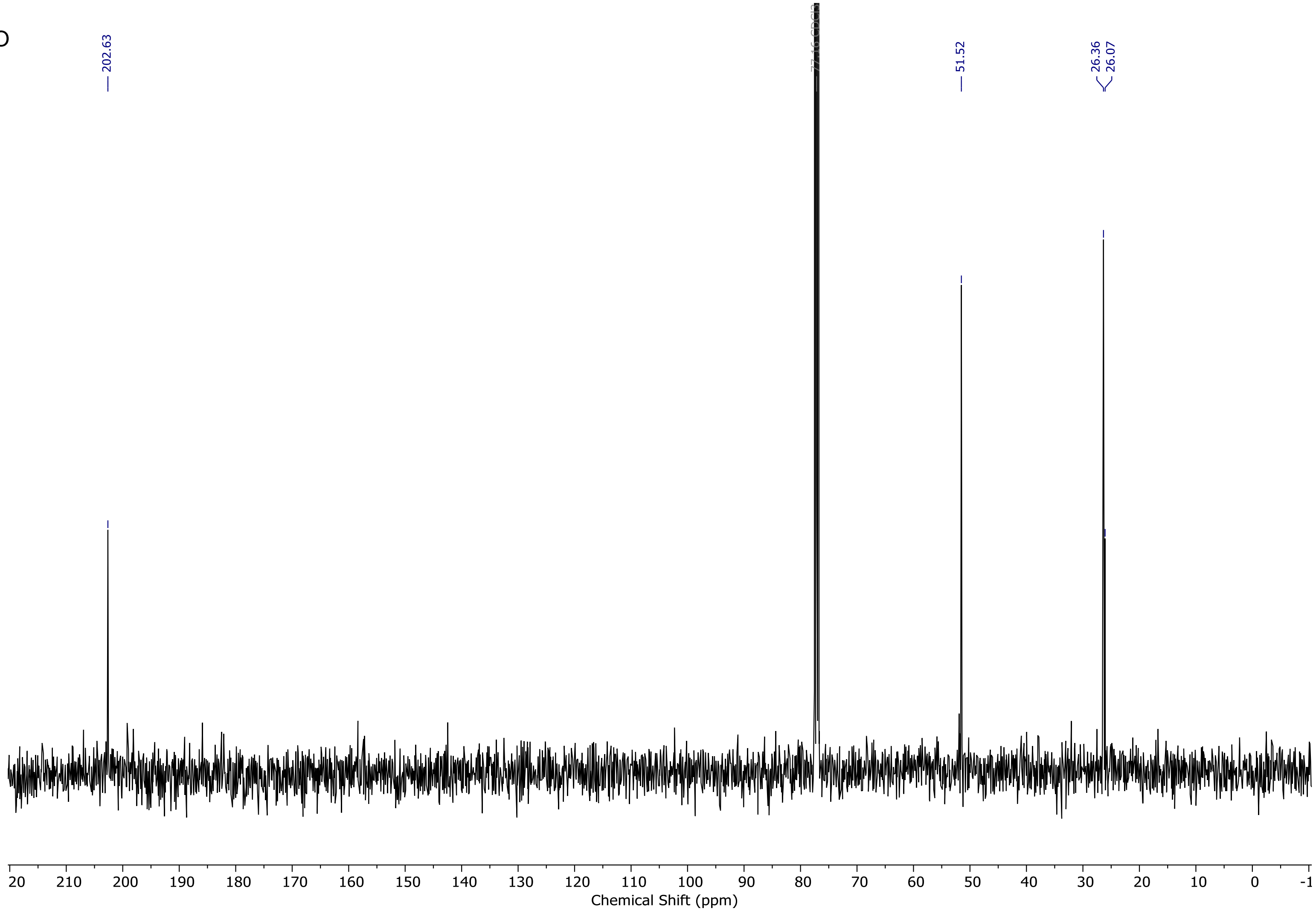

^{1}H NMR (500 MHz, $CDCl_3$) of pyridin-2-yl(p-tolyl)methanone 32c

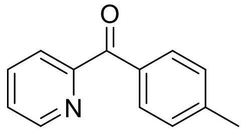

32c

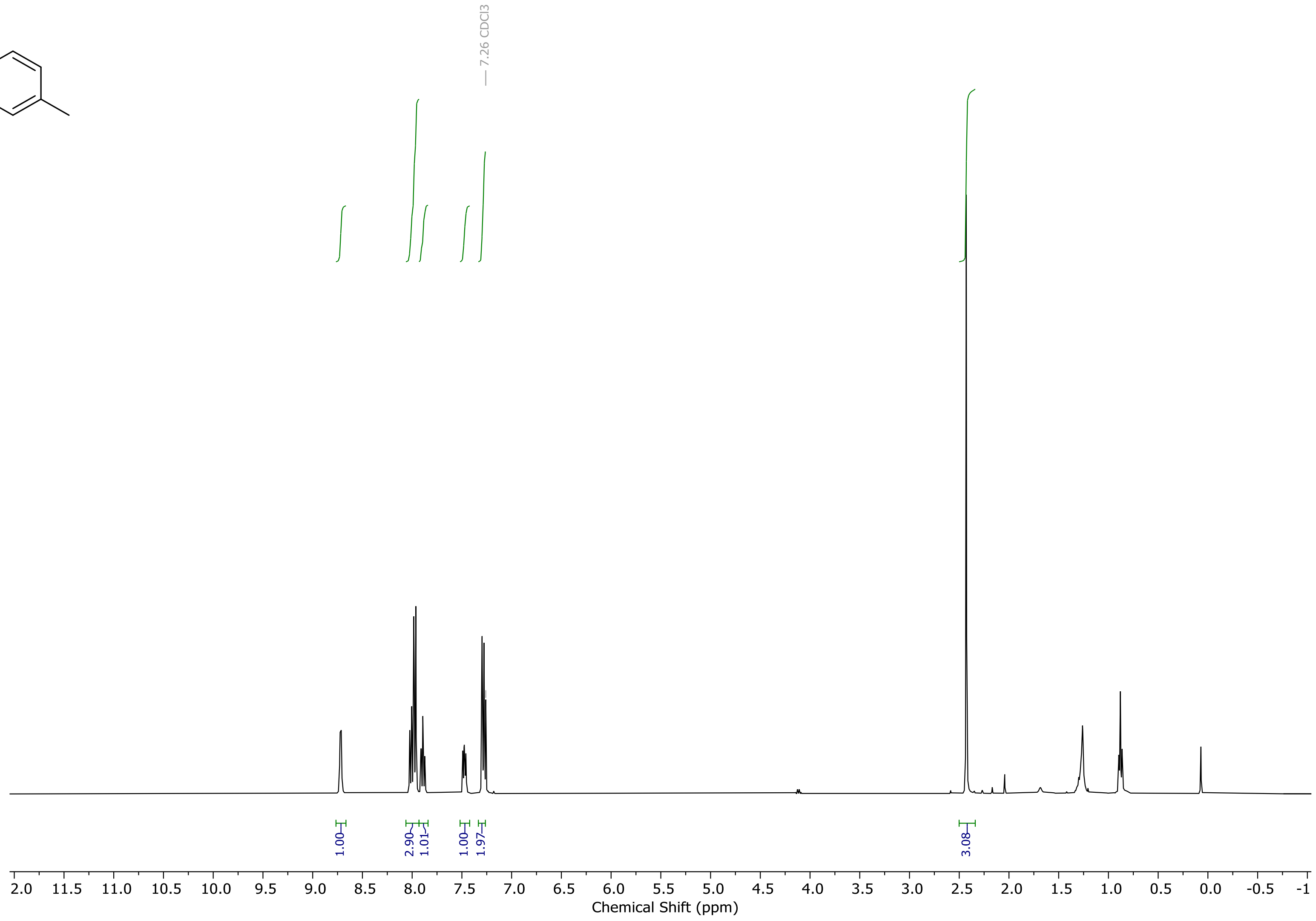

^{13}C NMR (101 MHz, $CDCl_3$) of pyridin-2-yl(p-tolyl)methanone 32c

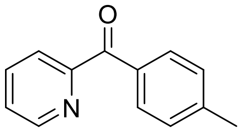

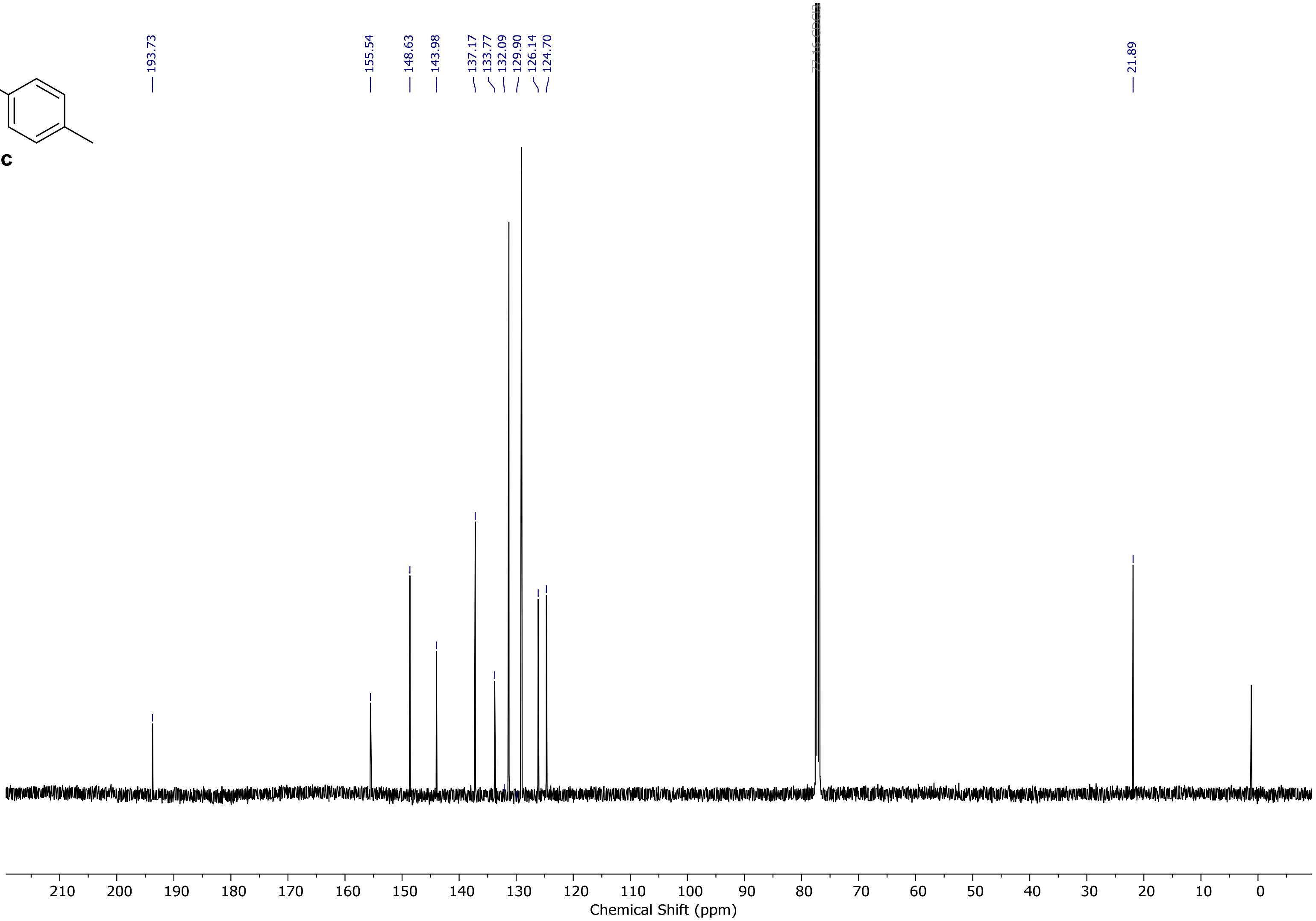

^{1}H NMR (400 MHz, $CDCl_3$) of 2,7-dichloro-4-(2-(dibutylamino)-1-hydroxyethyl)-9H-fluoren-9-one 32d

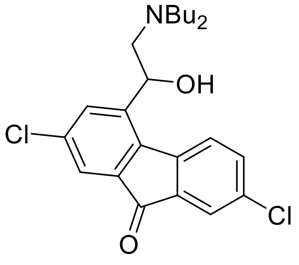

32d

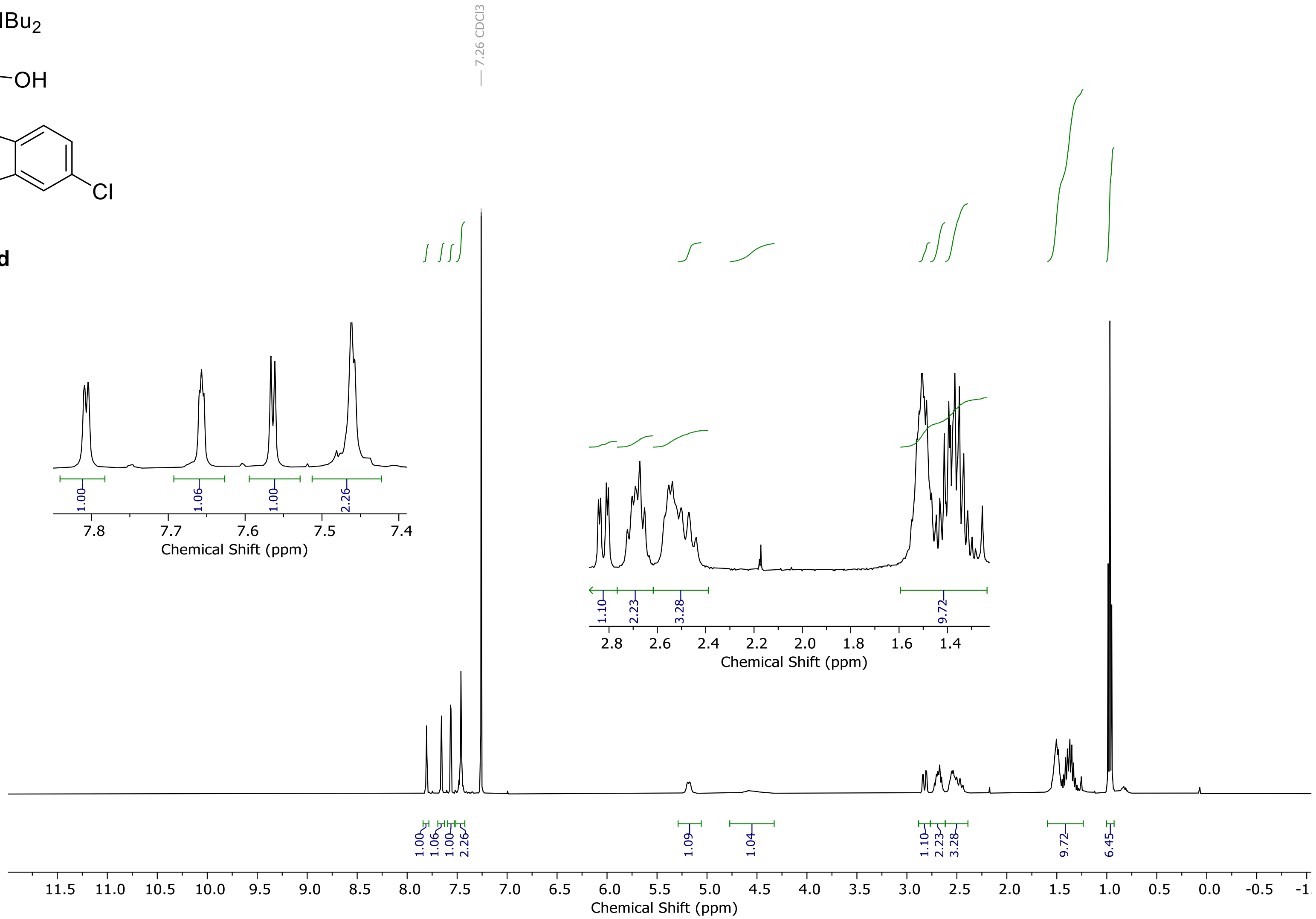

^{13}C NMR (101 MHz, $CDCl_3$) of 2,7-dichloro-4-(2-(dibutylamino)-1-hydroxyethyl)-9H-fluoren-9-one 32d

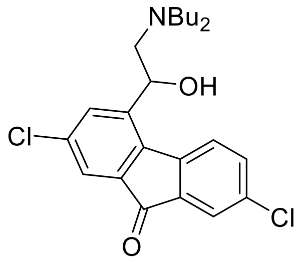

32d

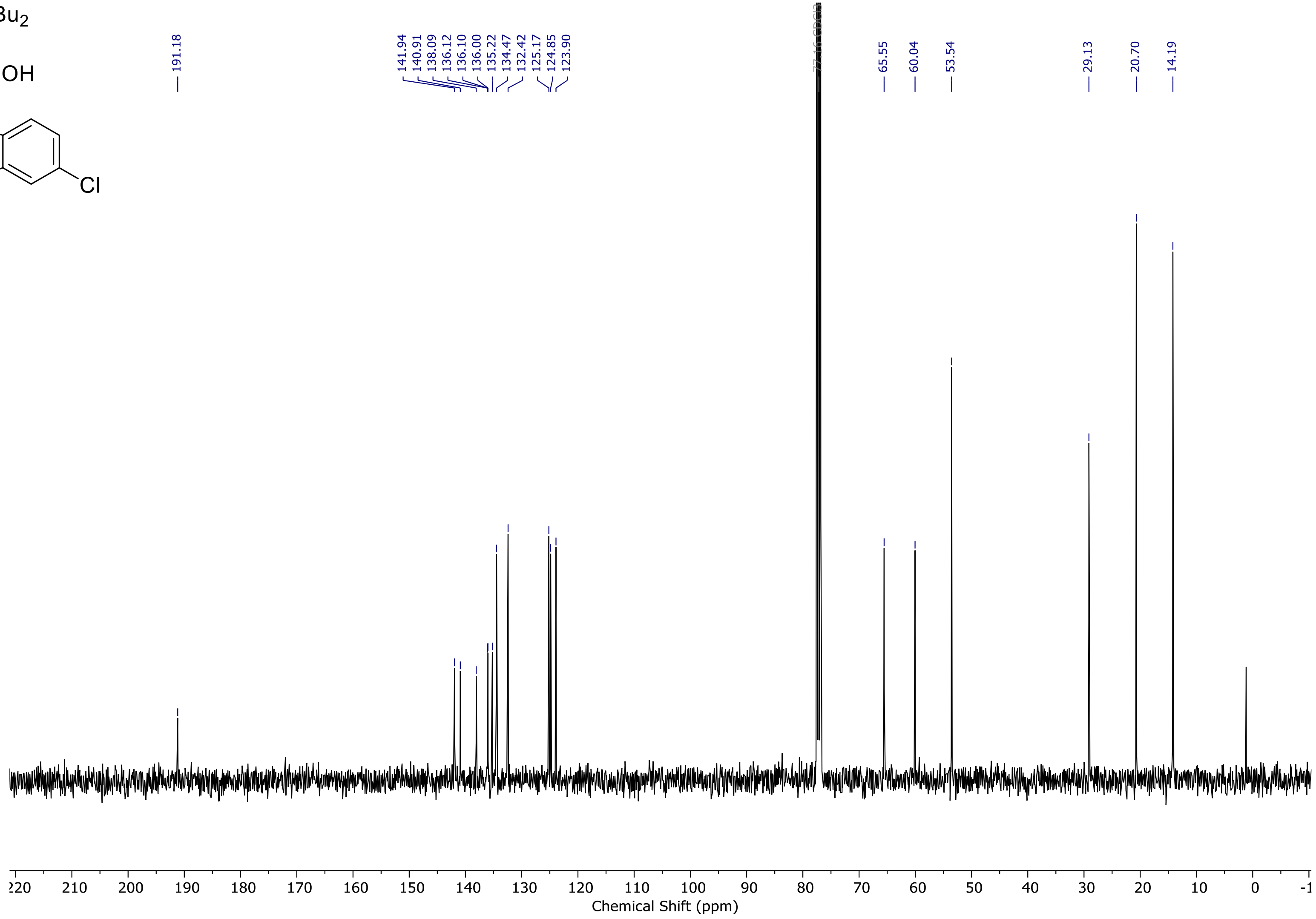

^{1}H NMR (500 MHz, $CDCl_3$) of 4-chlorobenzaldehyde 32d'

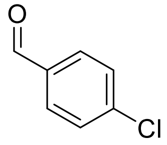

32d'

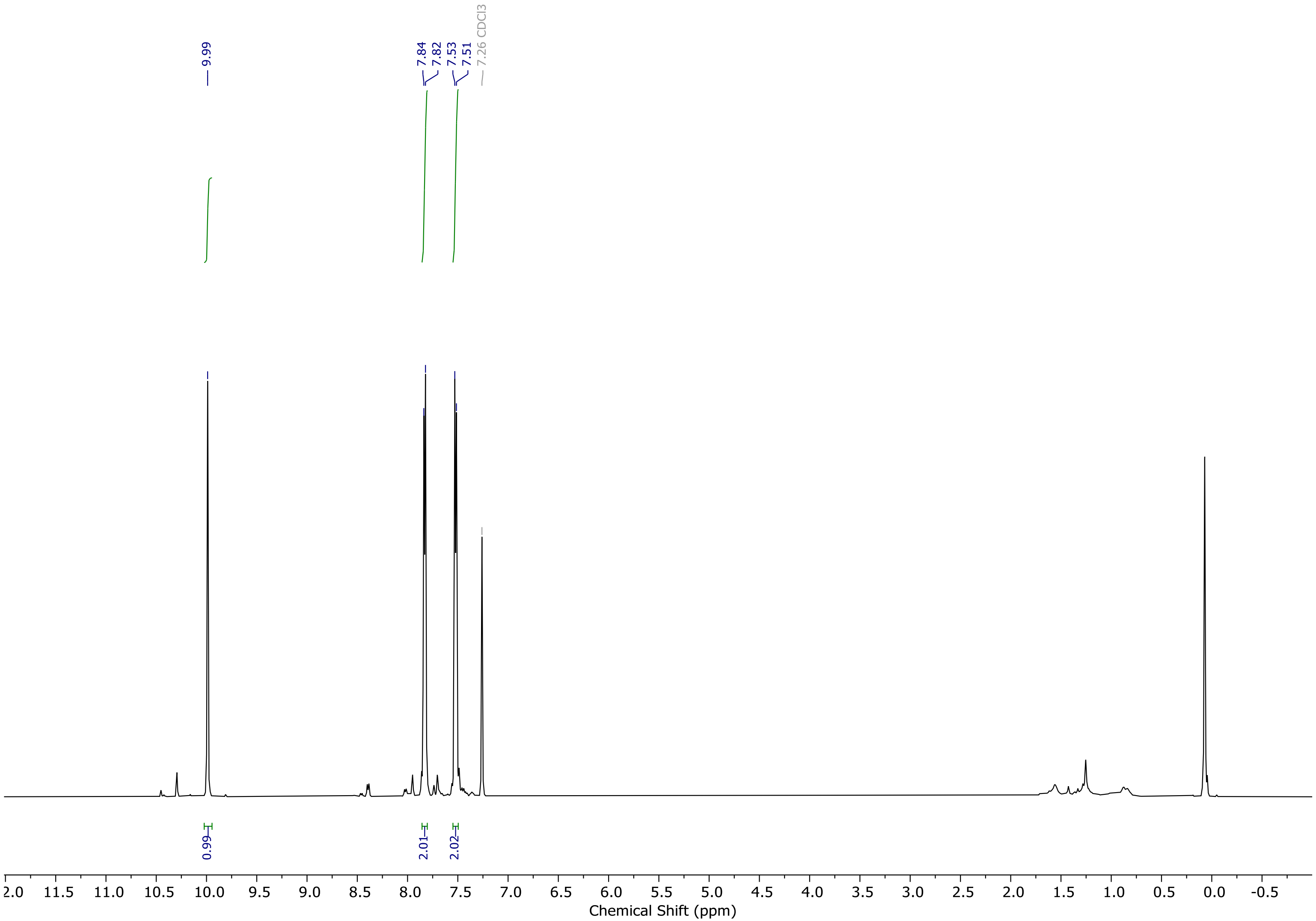

^{13}C NMR (126 MHz, $CDCl_3$) of 4-chlorobenzaldehyde 32d'

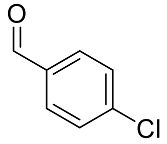

32d'

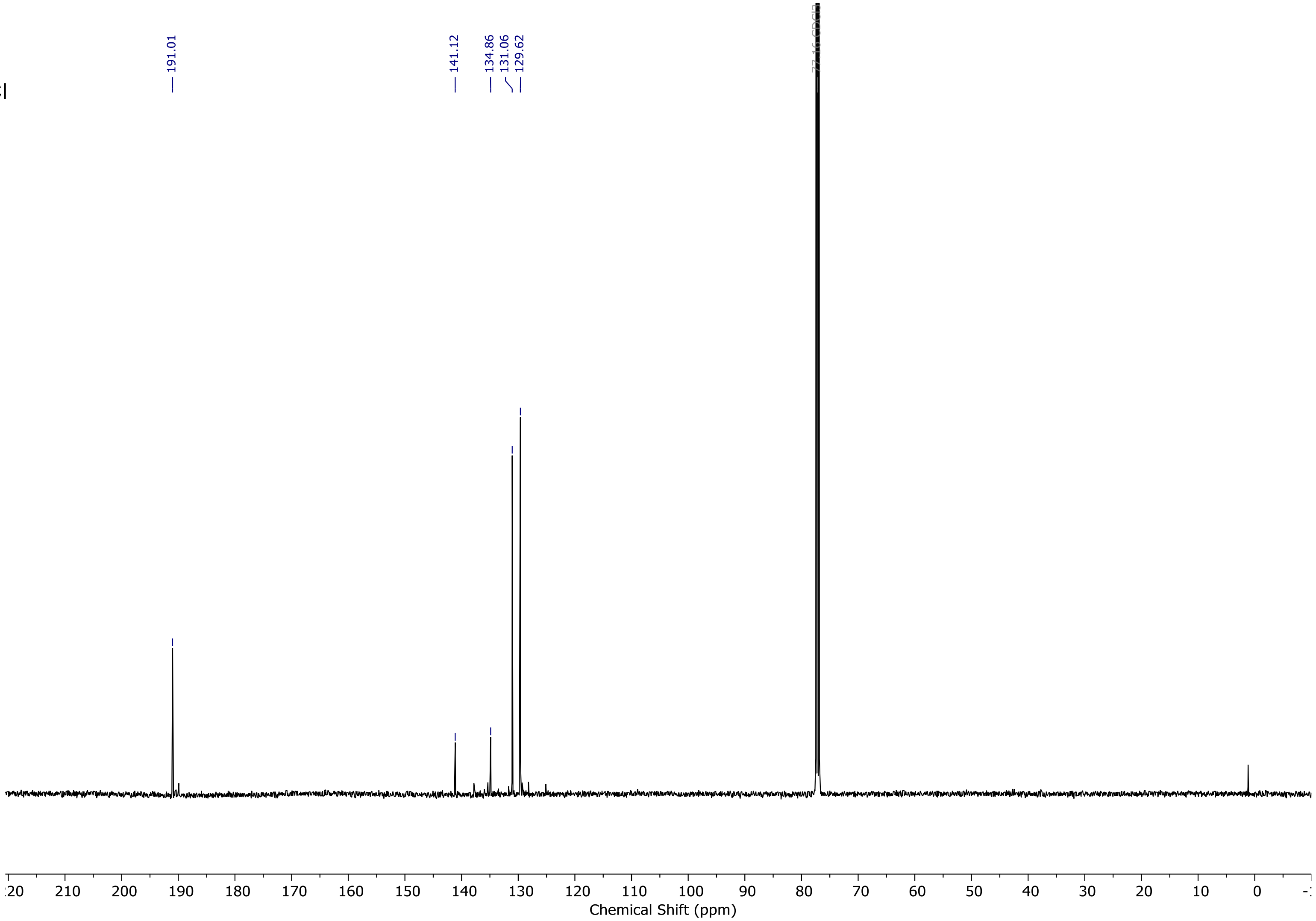

^{1}H NMR (400 MHz, $CDCl_3$) of 4-methoxybenzaldehyde 32e

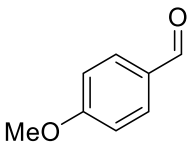

32e

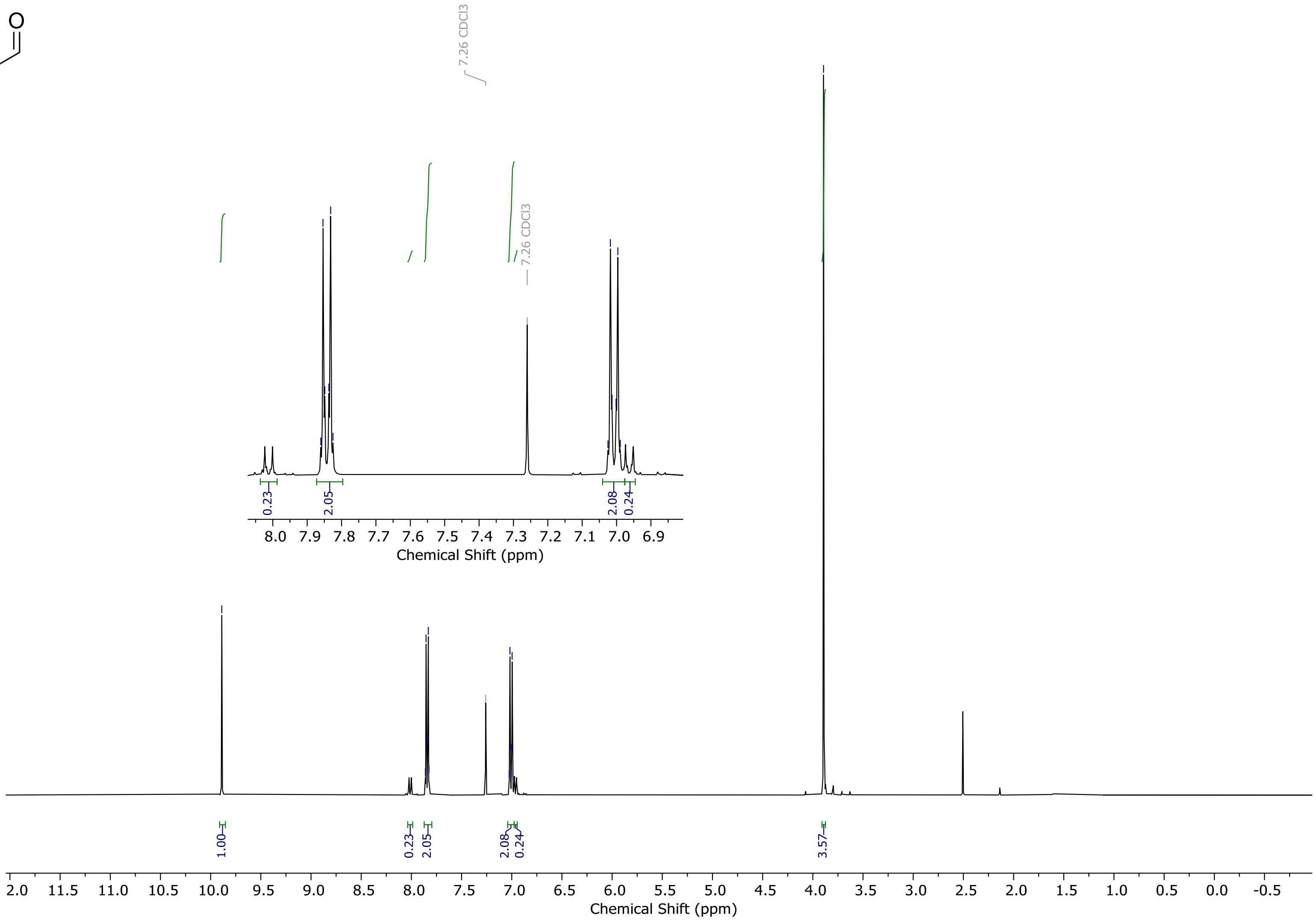

^{13}C NMR (101 MHz, $CDCl_3$) of 4-methoxybenzaldehyde 32e

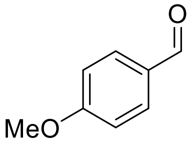

32e

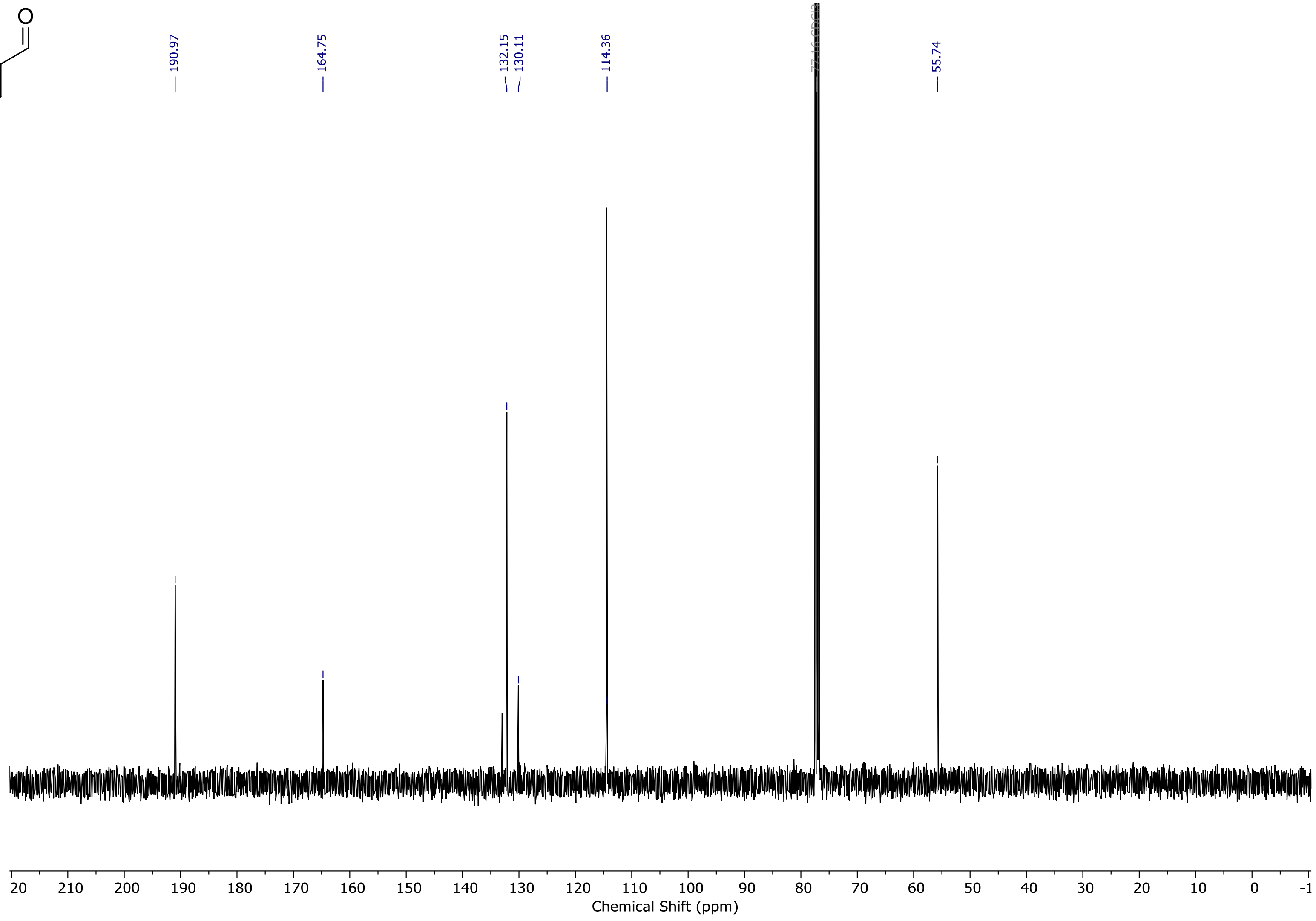

^{1}H NMR (400 MHz, $CDCl_3$) of 1-(4-(trifluoromethyl)phenyl)ethan-1-ol 34a (from transfer hydrogenation)

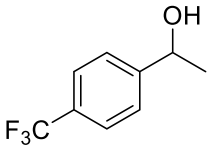

34a

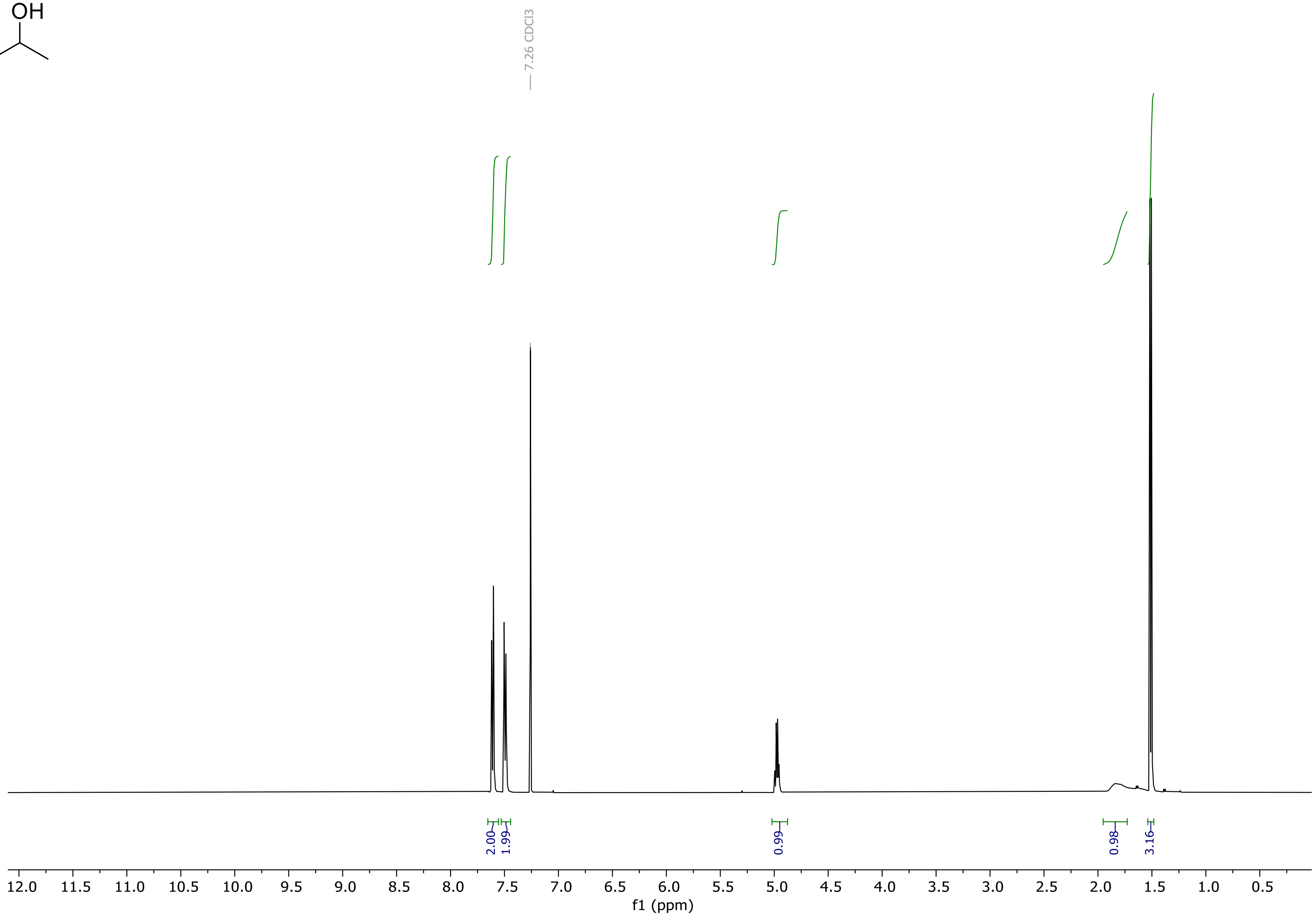

^{13}C NMR (101 MHz, $CDCl_3$) of 1-(4-(trifluoromethyl)phenyl)ethan-1-ol 34a (from transfer hydrogenation)

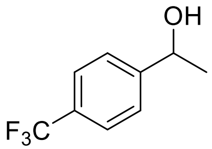

34a

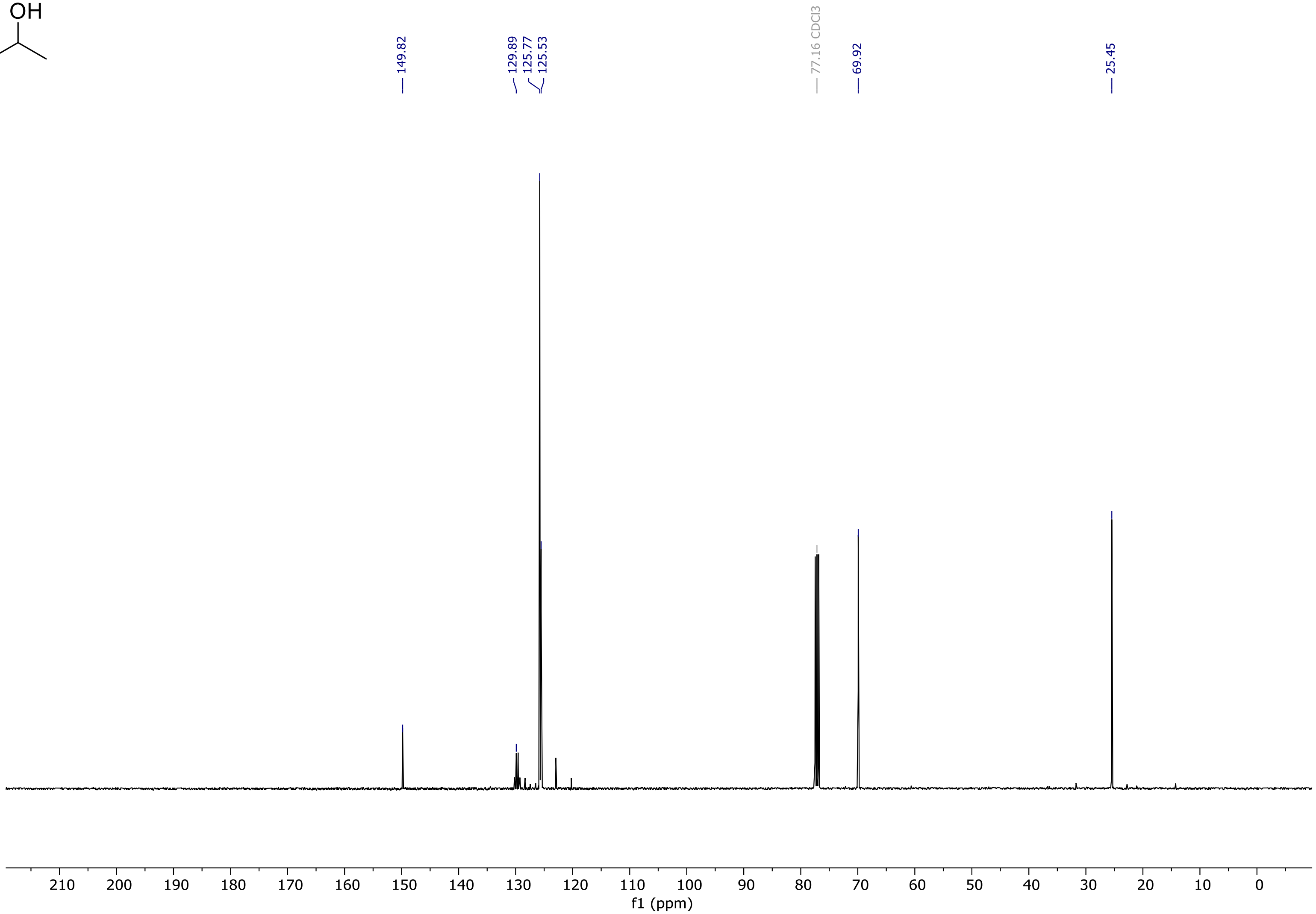

^{19}F NMR (376 MHz, $CDCl_3$) of 1-(4-(trifluoromethyl)phenyl)ethan-1-ol 34a (from transfer hydrogenation)

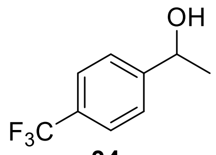

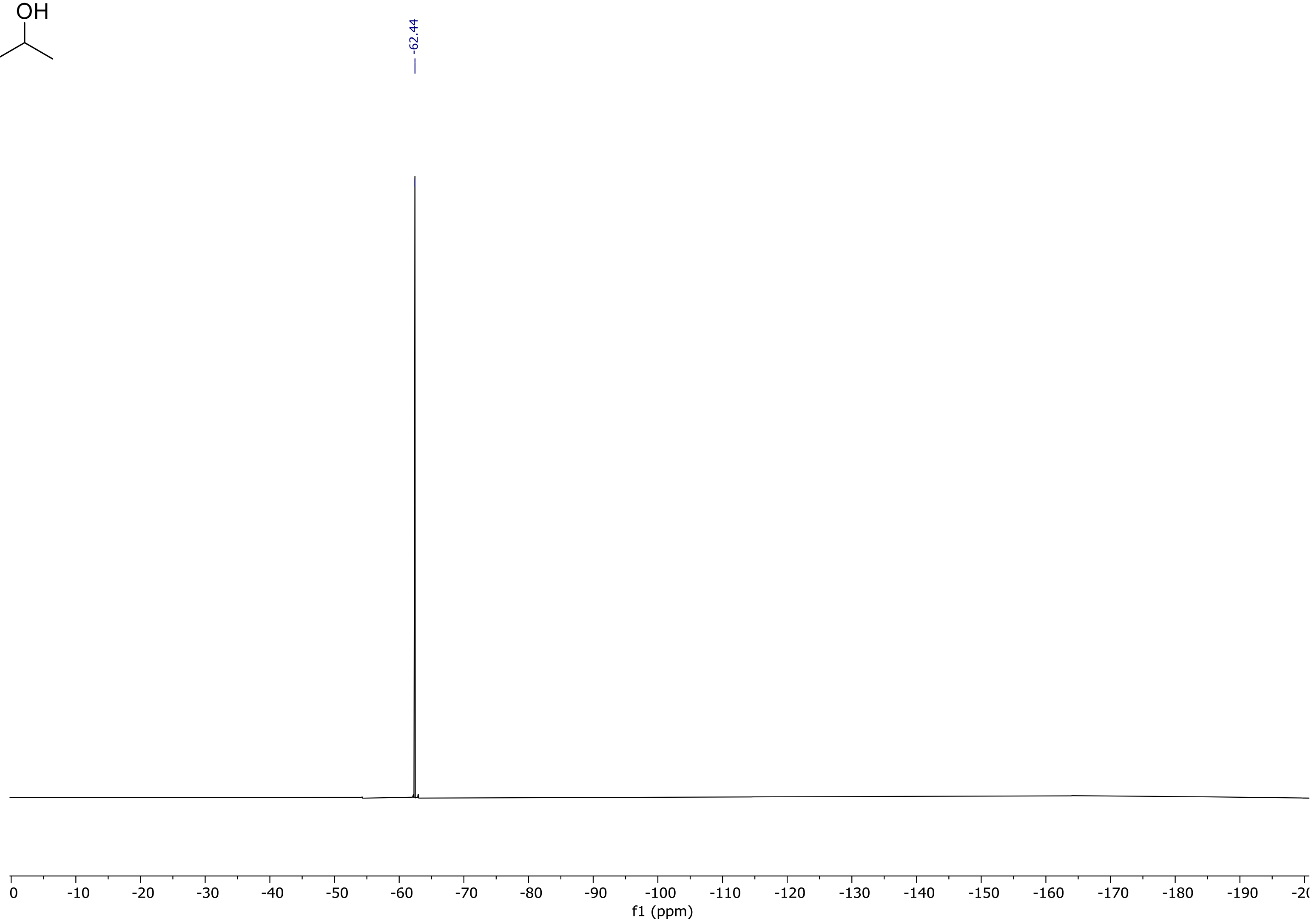

^{1}H NMR (500 MHz, $CDCl_3$) of 1-(Naphthalen-1-yl)ethan-1-ol 34b

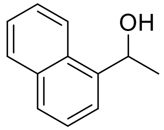

34b

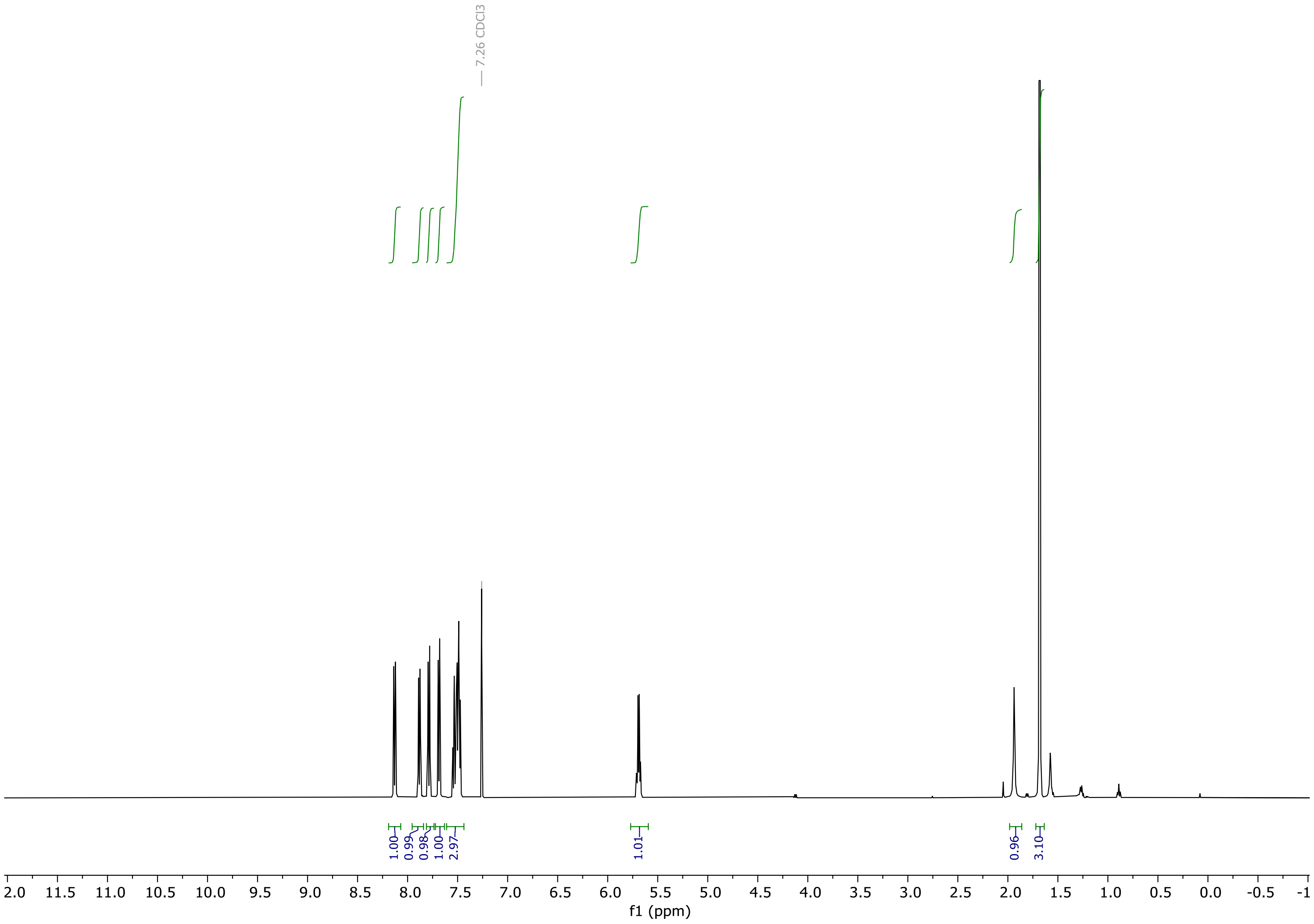

^{13}C NMR (126 MHz, $CDCl_3$) of 1-(Naphthalen-1-yl)ethan-1-ol 34b

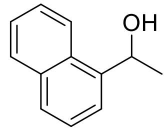

34b

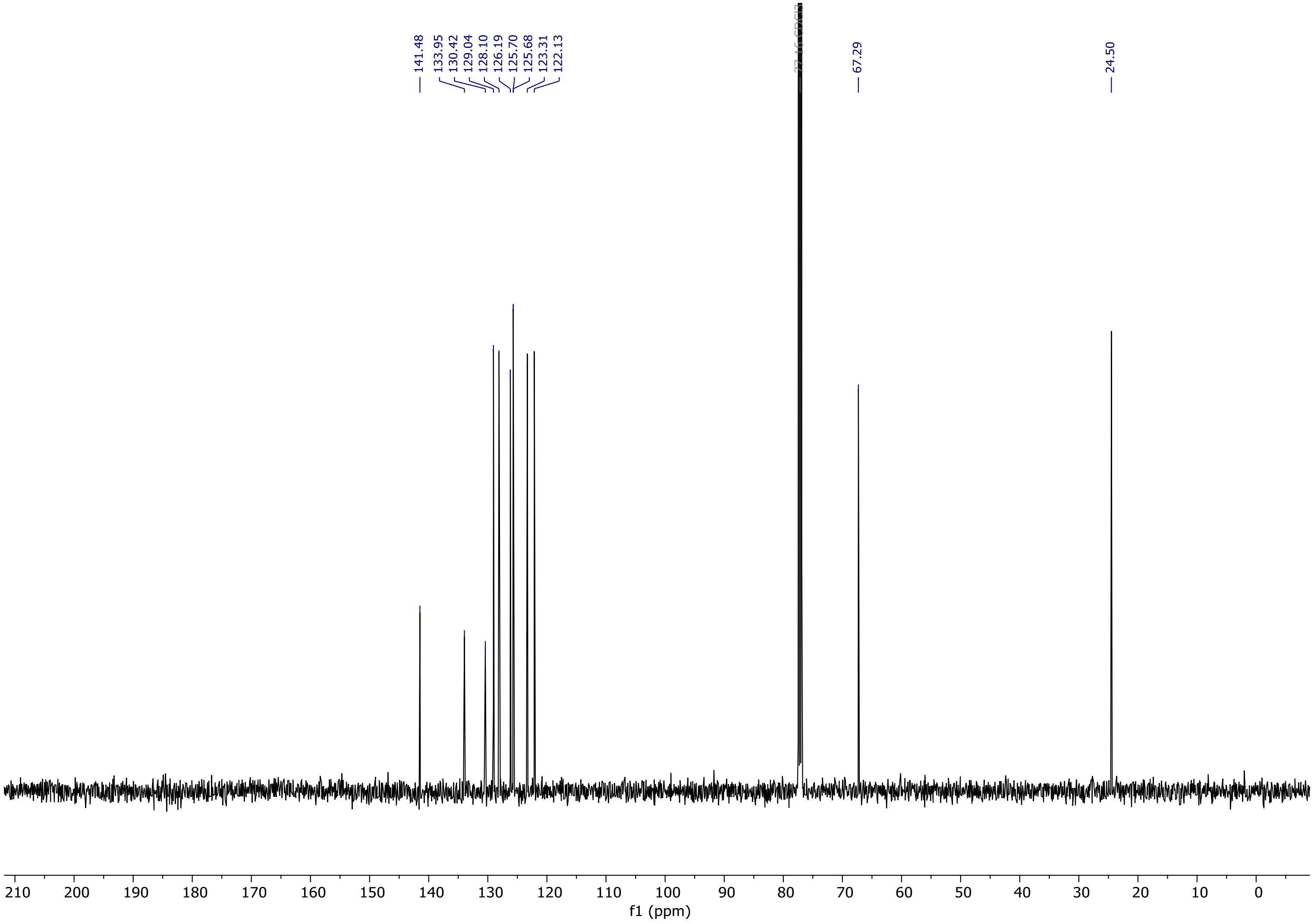

^{1}H NMR (500 MHz, $CDCl_3$) of 1-(4-Methoxyphenyl)ethan-1-ol 34c

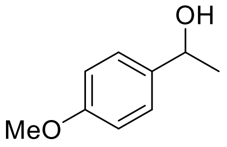

34c

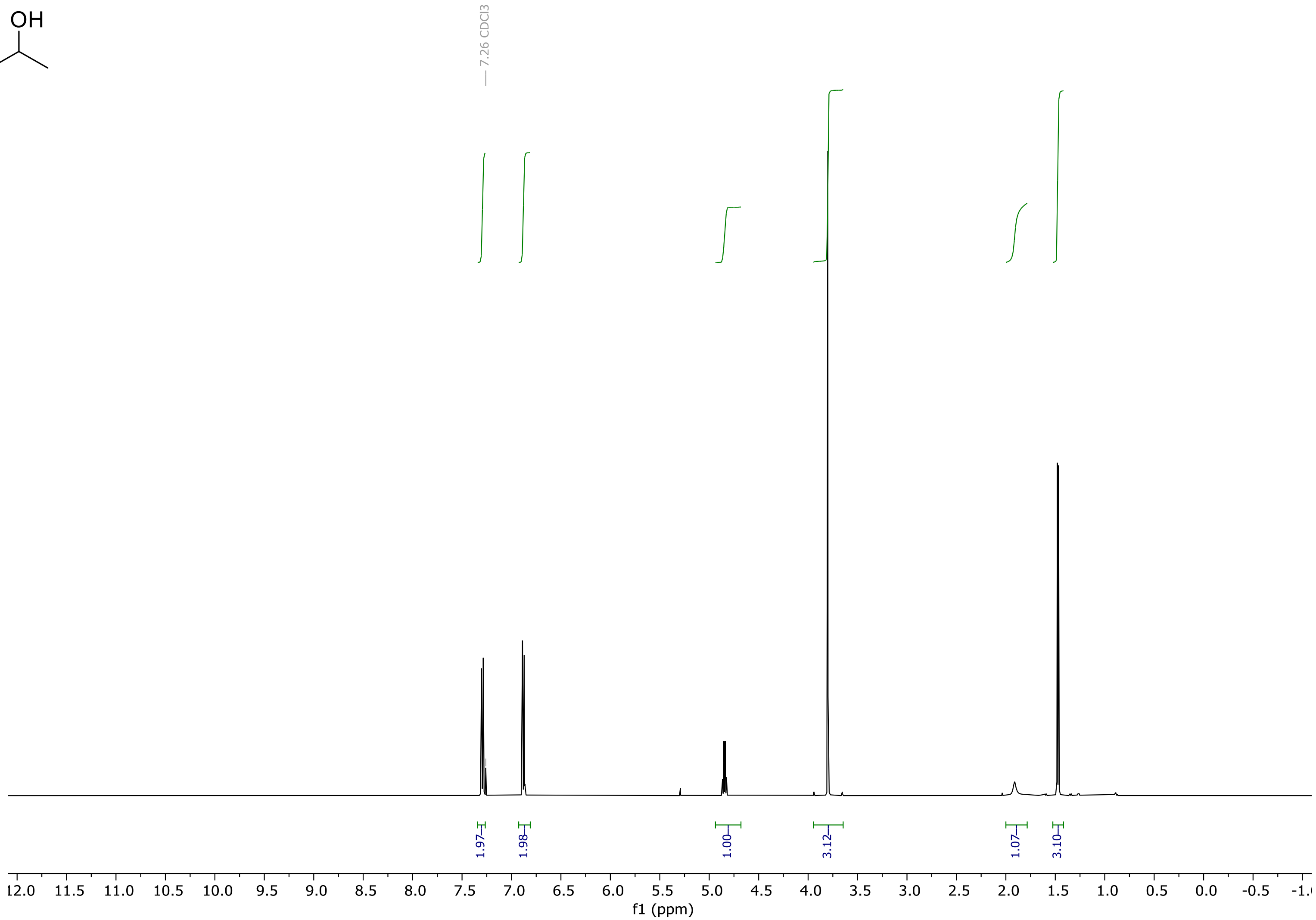

^{13}C NMR (126 MHz, $CDCl_3$) of 1-(4-Methoxyphenyl)ethan-1-ol 34c

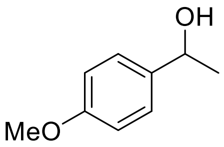

34c

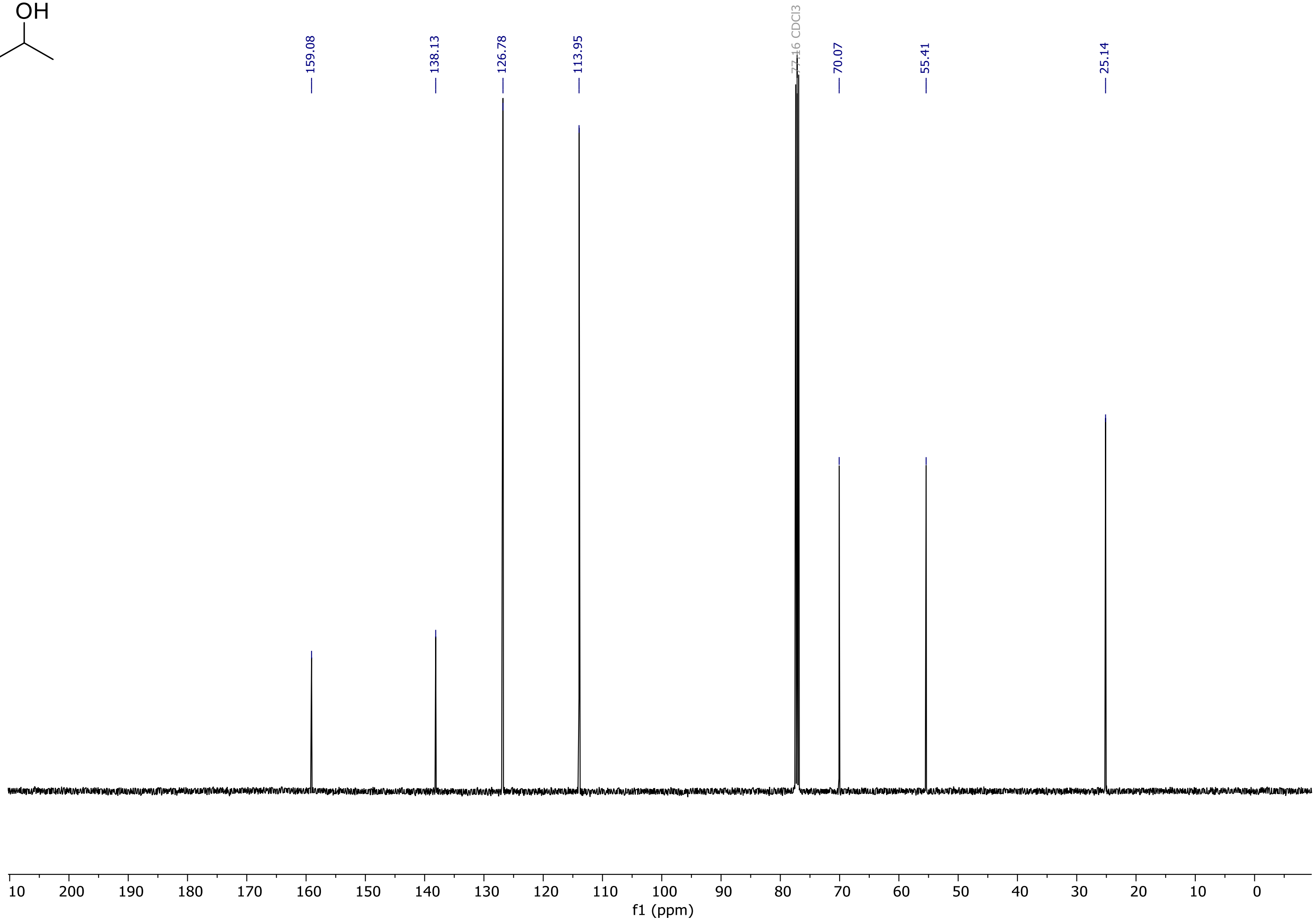

^{1}H NMR (500 MHz, $CDCl_3$) of 1-(3,5-Dimethylphenyl)ethan-1-ol 34d

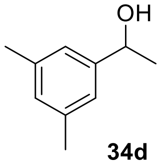

34d

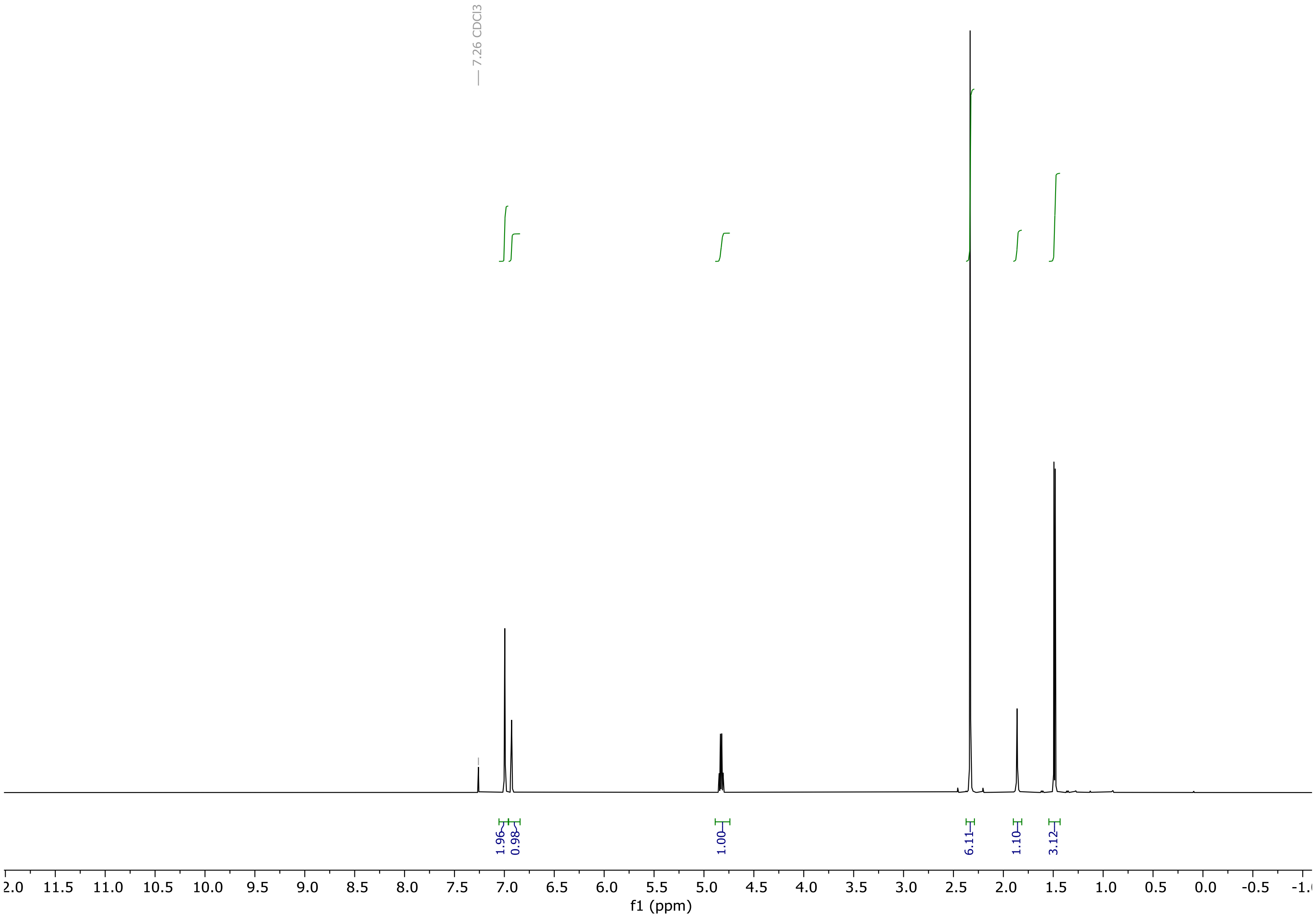

^{13}C NMR (126 MHz, $CDCl_3$) of 1-(3,5-Dimethylphenyl)ethan-1-ol 34d

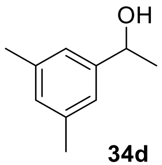

34d

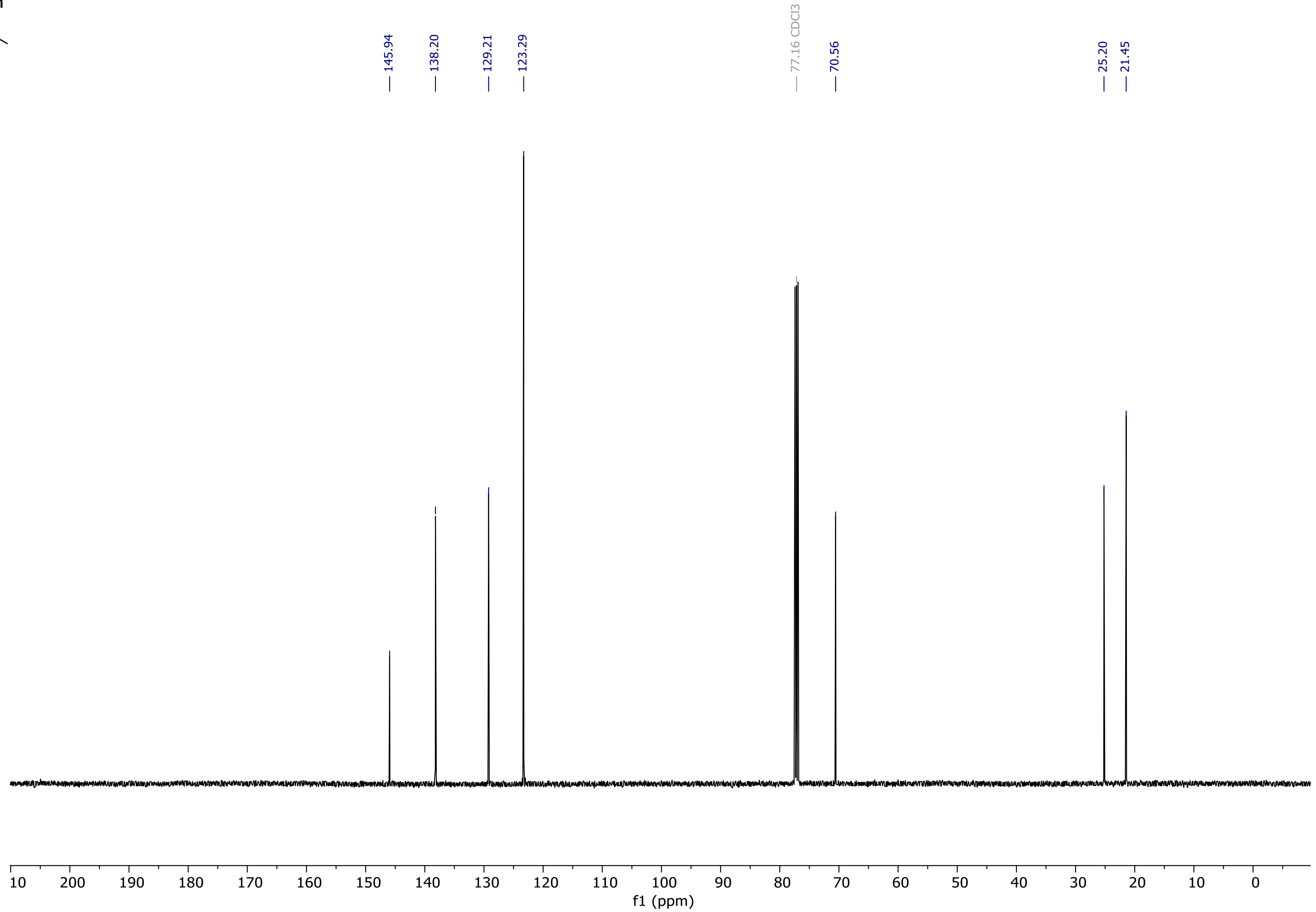

^{1}H NMR (500 MHz, $CDCl_3$) of Isopropyl 2-{4-[(4-chlorophenyl)(hydroxy)methyl]phenoxy}-2-methylpropanoate 34e

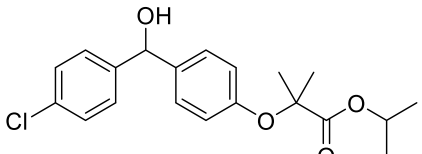

34e

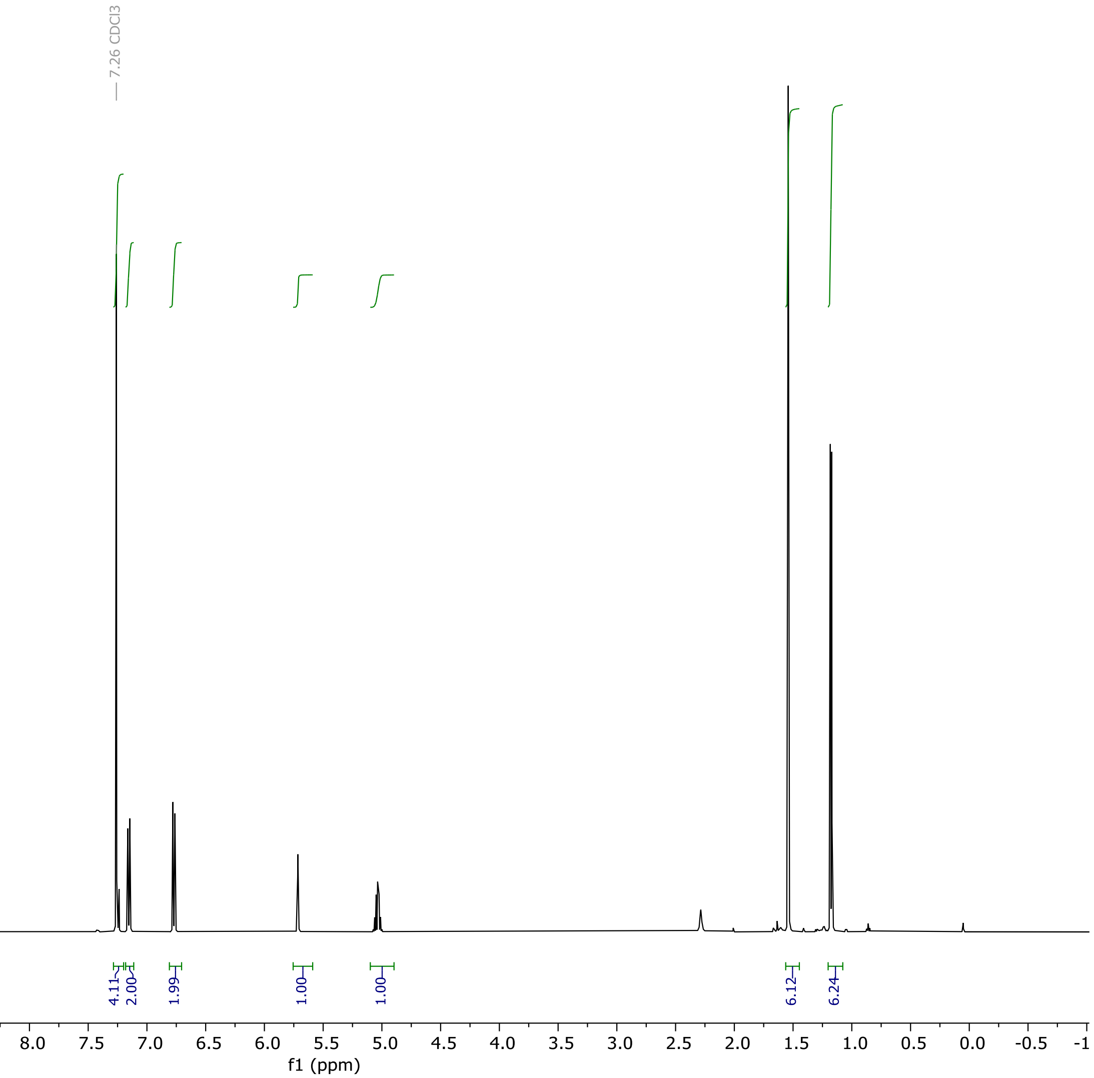

^{13}C NMR (126 MHz, $CDCl_3$) of Isopropyl 2-{4-[(4-chlorophenyl)(hydroxy)methyl]phenoxy}-2-methylpropanoate 34e

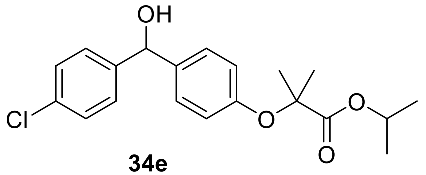

34e

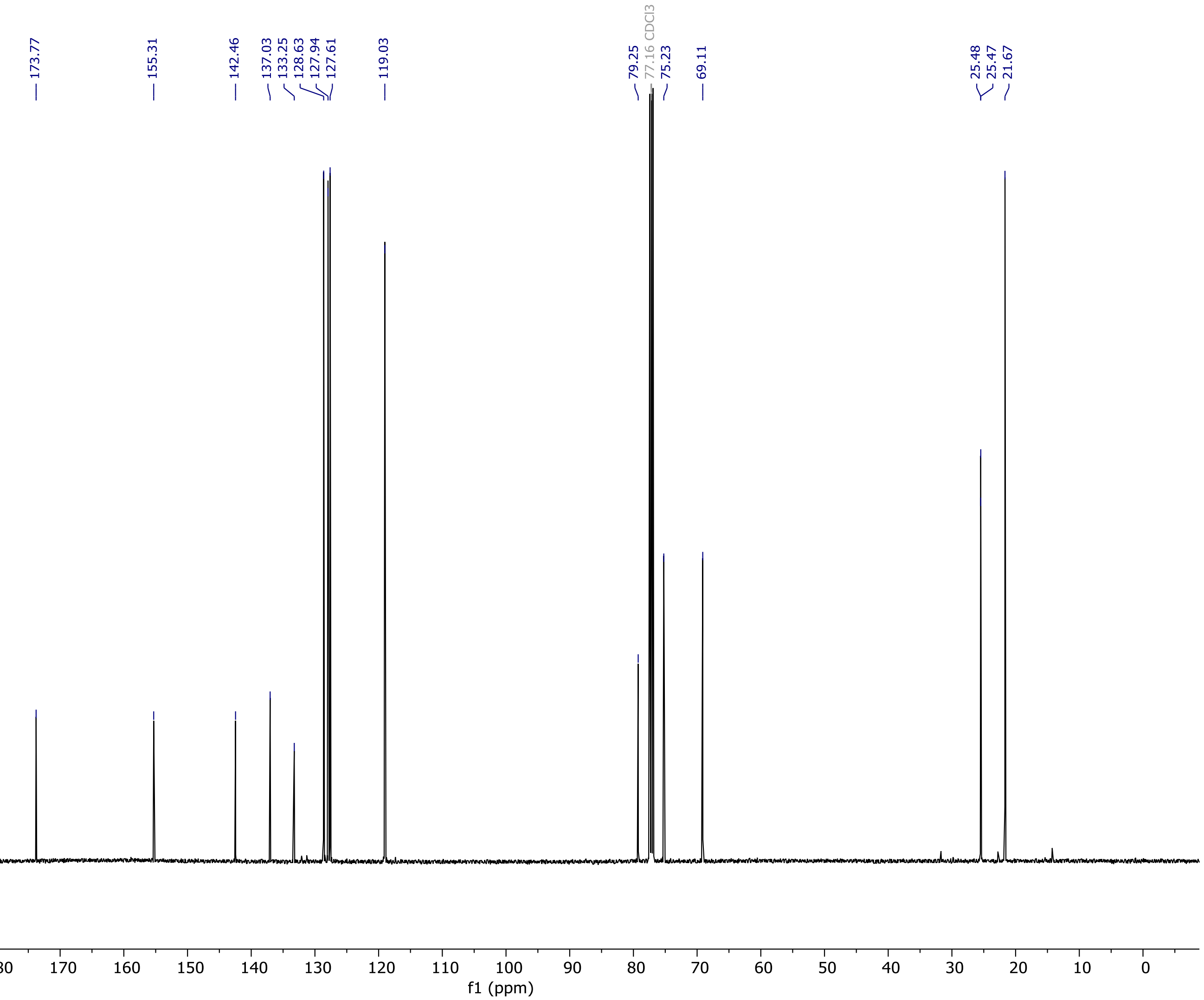

^{1}H NMR (400 MHz, DMSO) of Tris(2,2′-bipyridine)ruthenium(II) tetrafluoroborate 35

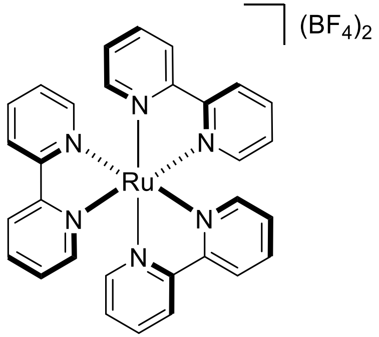

35

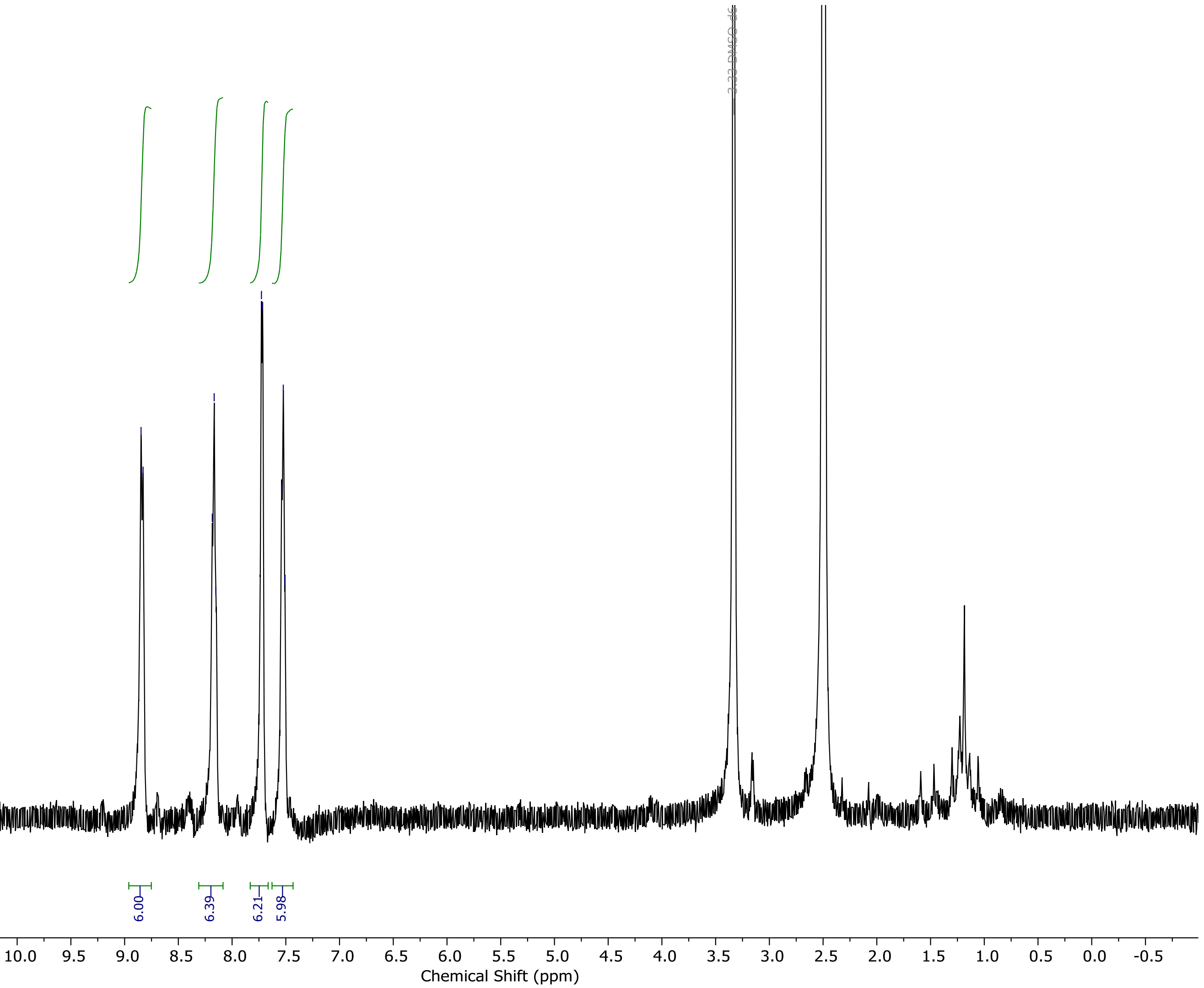

^{13}C NMR (126 MHz, DMSO) of Tris(2,2′-bipyridine)ruthenium(II) tetrafluoroborate 35

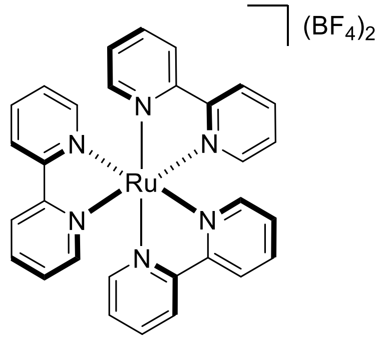

35

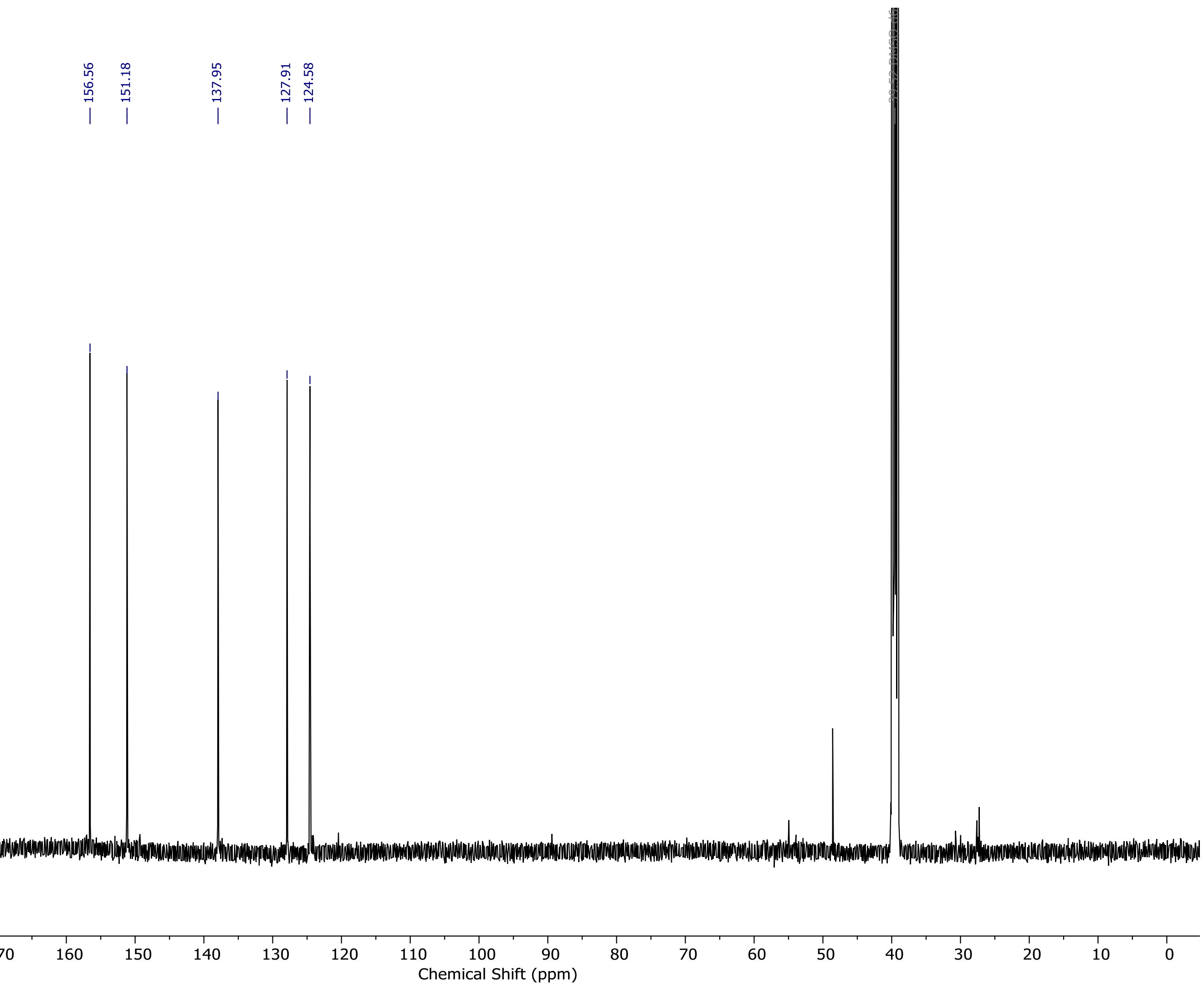

^{1}H NMR (500 MHz, DMSO) of Tris(*p*-Me-2,2′-bipyridine)ruthenium(II) tetrafluoroborate 36

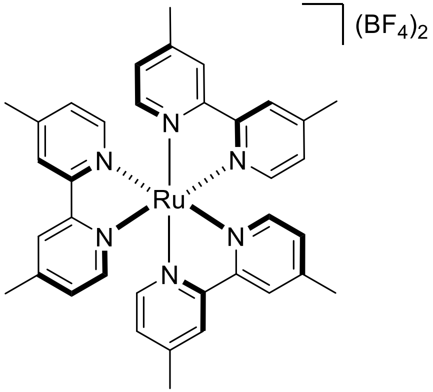

36

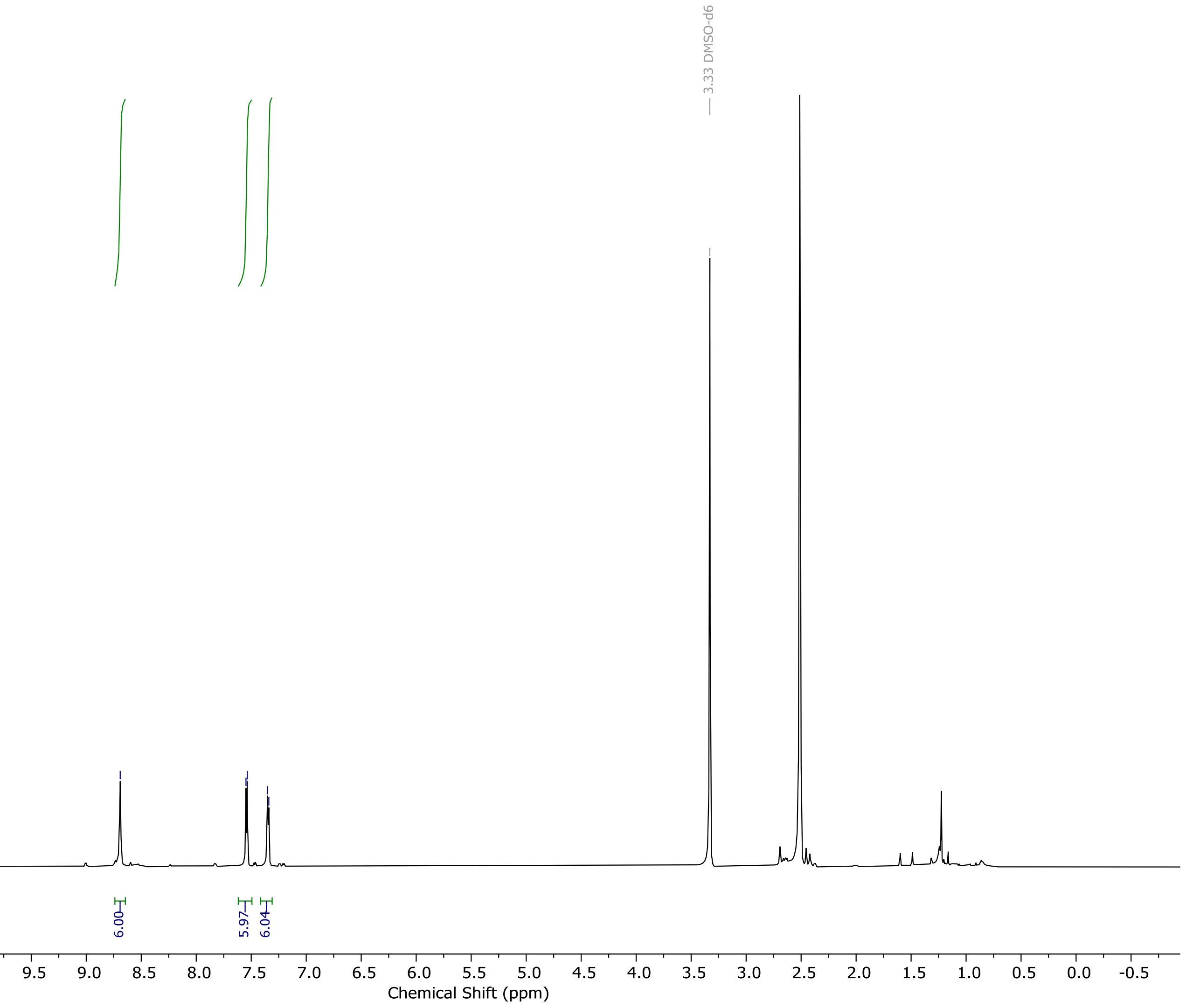

^{13}C NMR (126 MHz, DMSO) of Tris(*p*-Me-2,2′-bipyridine)ruthenium(II) tetrafluoroborate 36

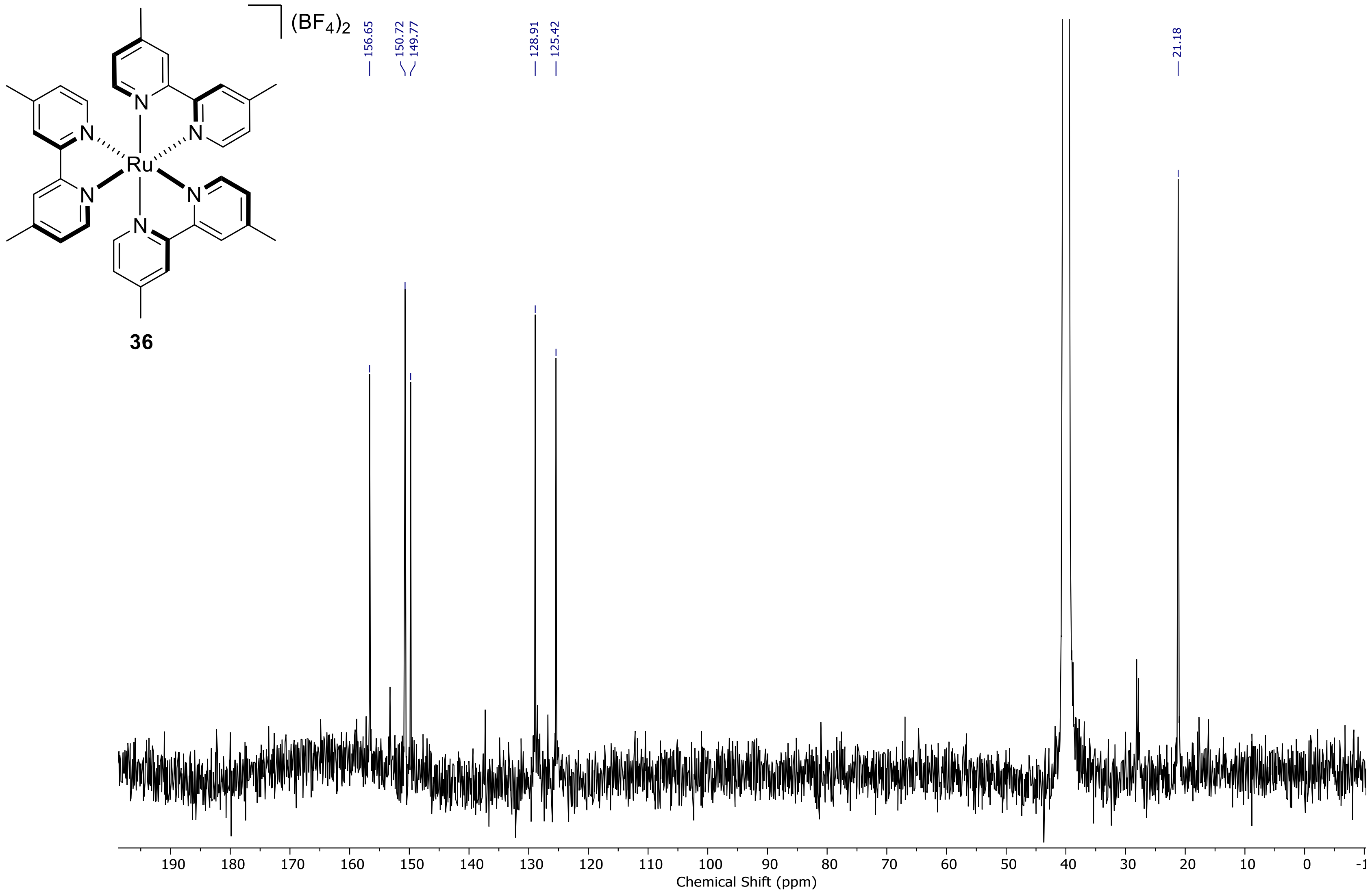

^{1}H NMR (400 MHz, DMSO) of Tris(*p*-[t]Bu-2,2'-bipyridine)ruthenium(II) tetrafluoroborate 37

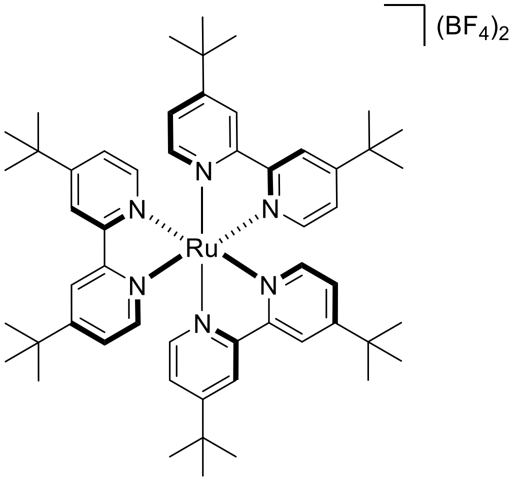

37

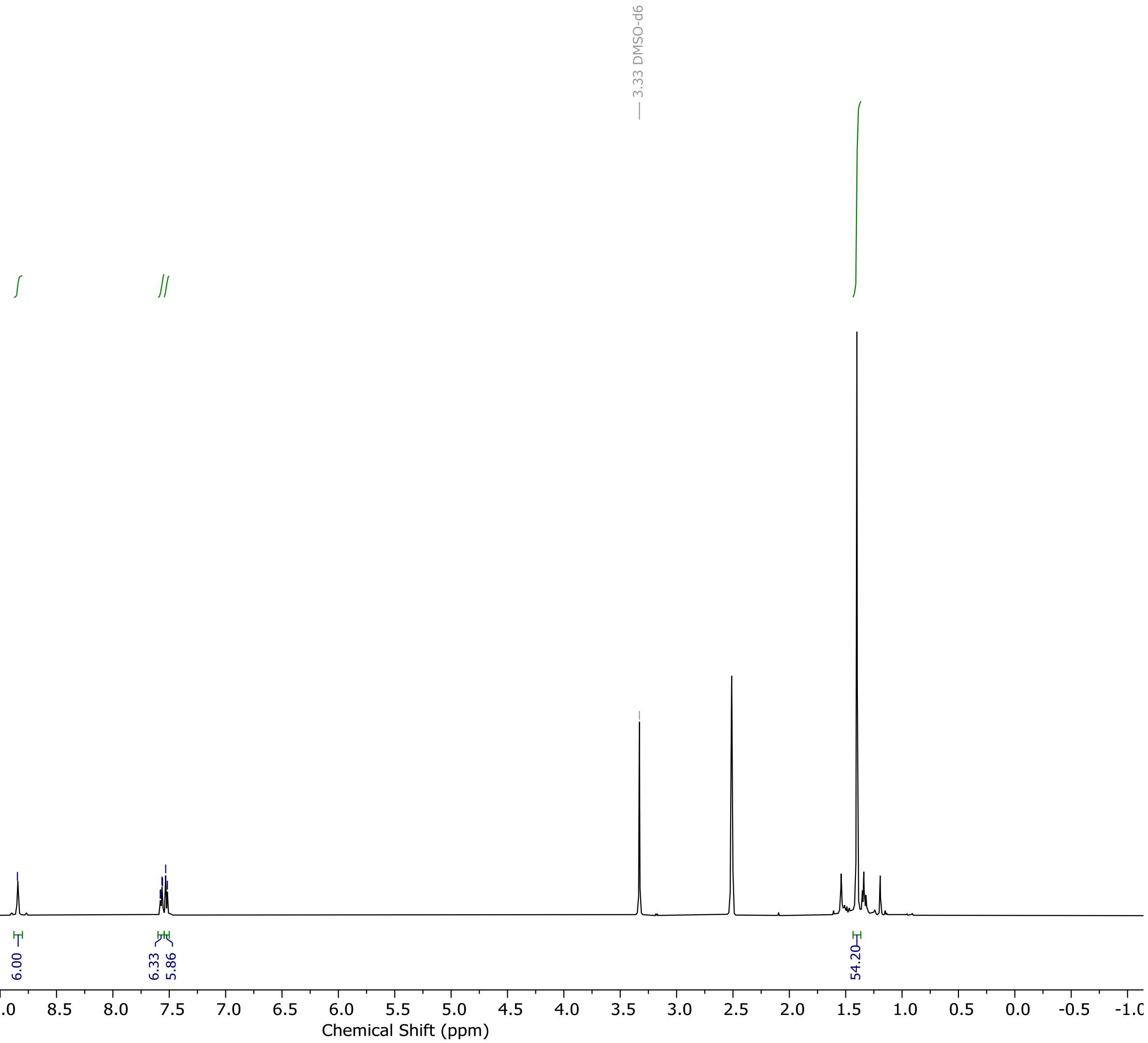

^{13}C NMR (101 MHz, DMSO) of Tris(*p*-^{t}Bu-2,2′-bipyridine)ruthenium(II) tetrafluoroborate 37

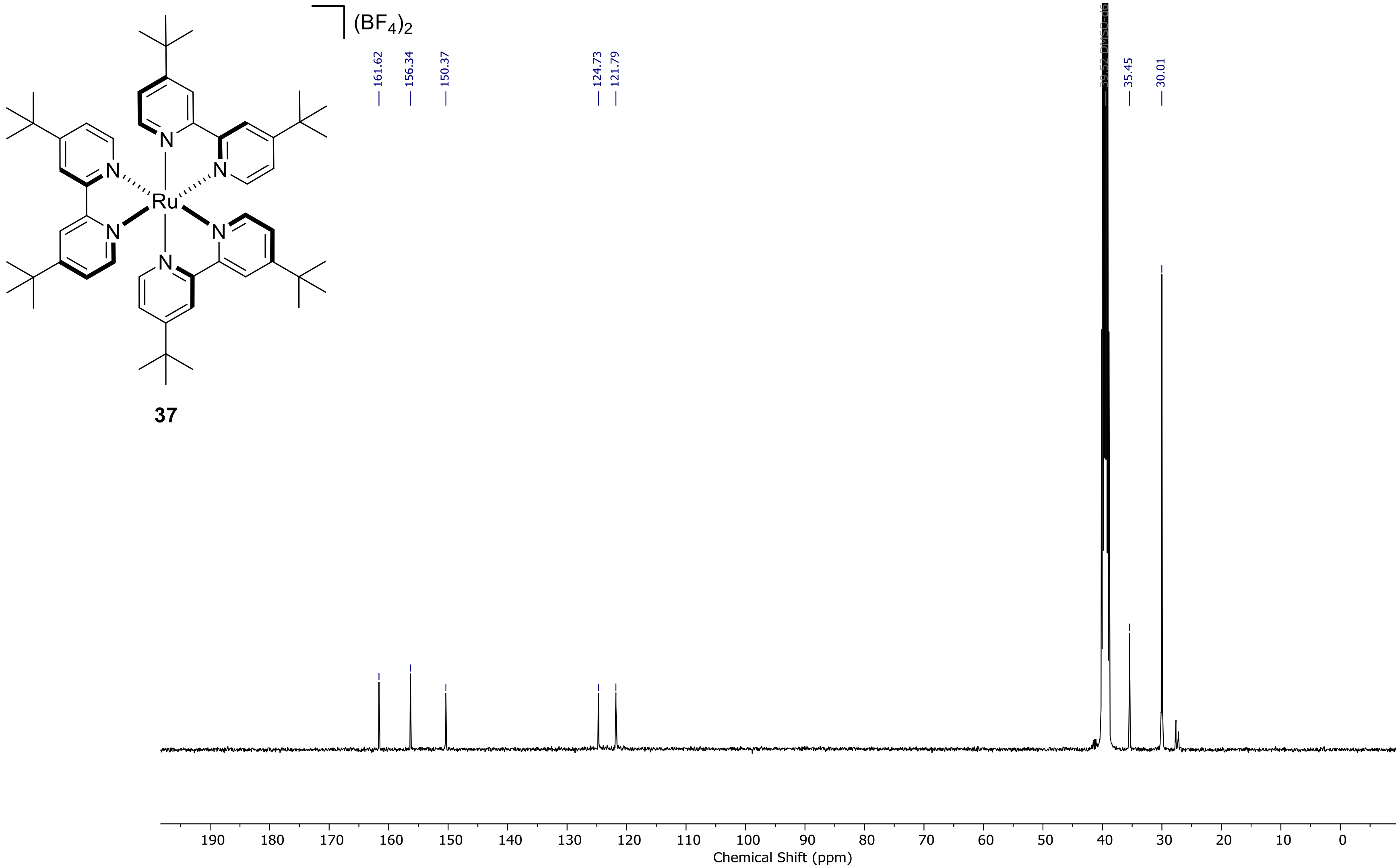

^{1}H NMR (500 MHz, DMSO) of Tris(1,10-phenanthroline)ruthenium(II) tetrafluoroborate 38

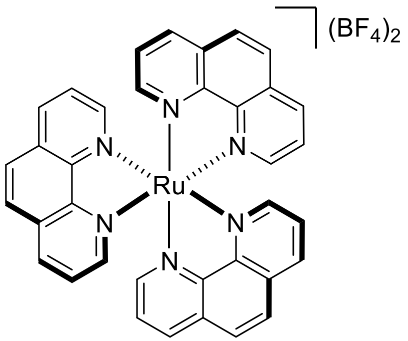

38

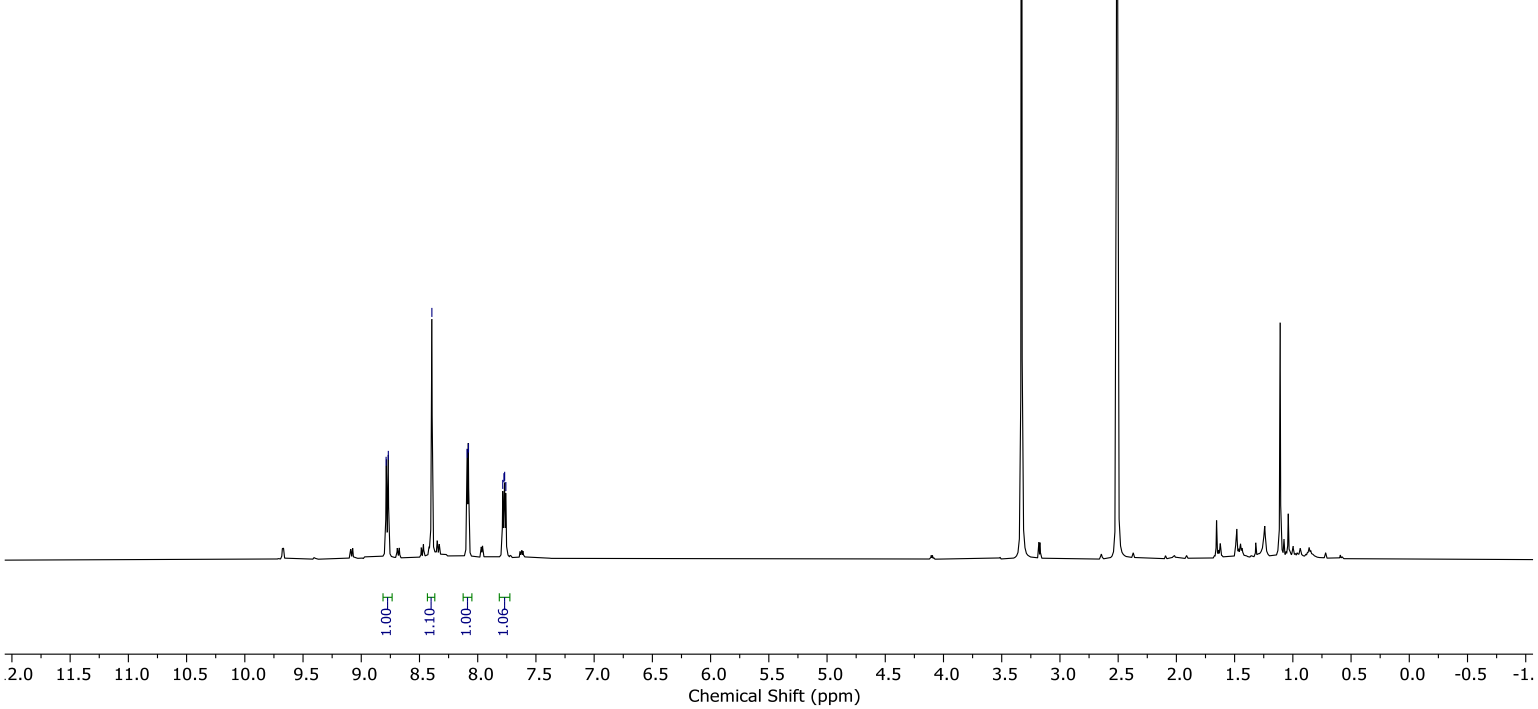

^{13}C NMR (126 MHz, DMSO) of Tris(1,10-phenanthroline)ruthenium(II) tetrafluoroborate 38

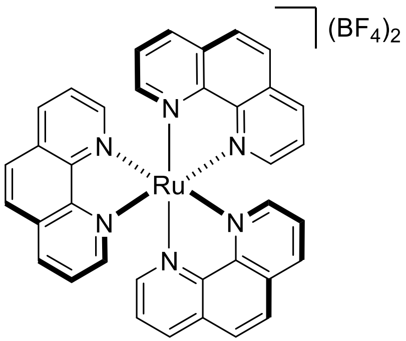

38

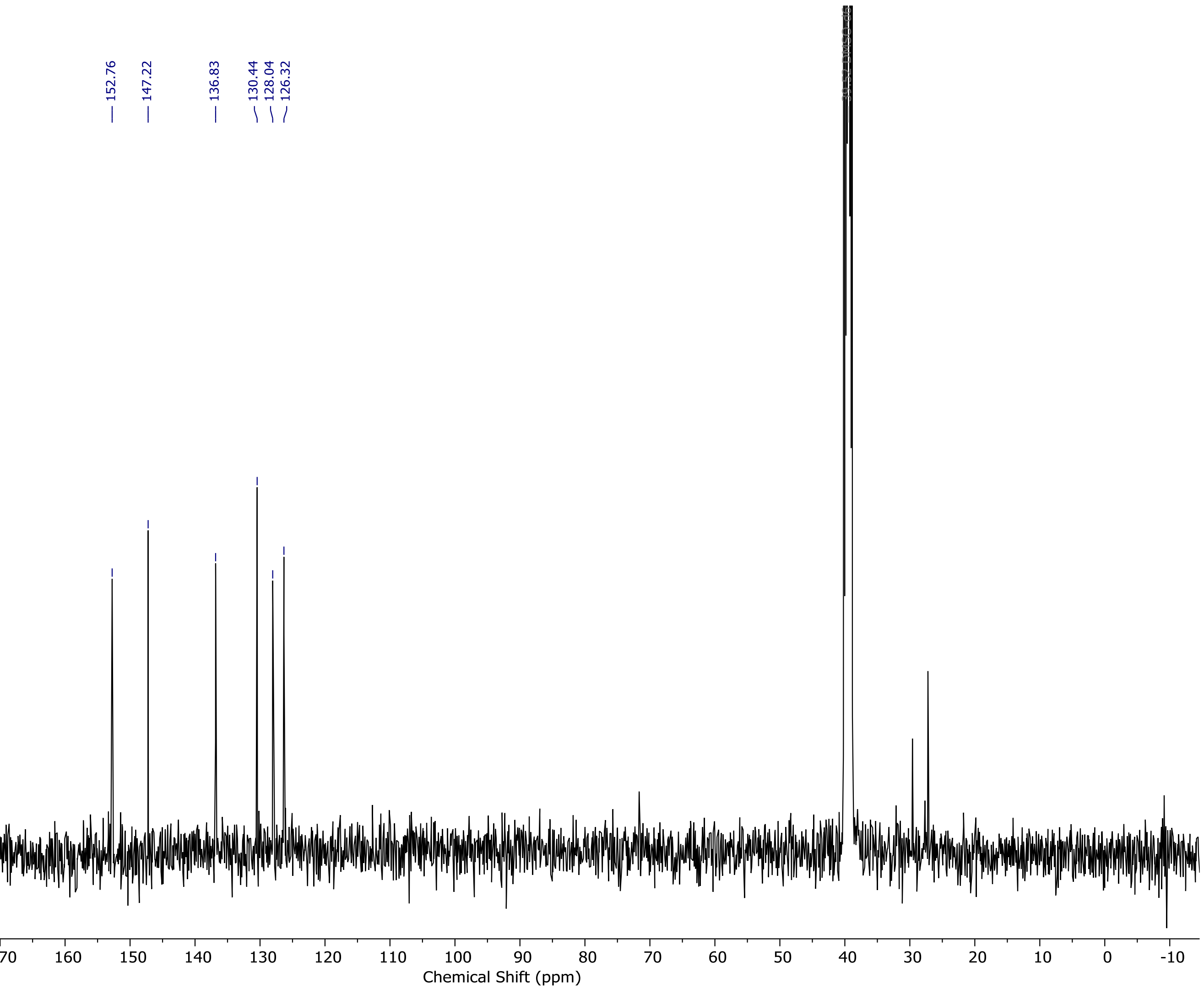

^{1}H NMR (400 MHz, DMSO) of Tris(3,4,7,8-tetramethyl phenanthroline)ruthenium(II) tetrafluoroborate 39

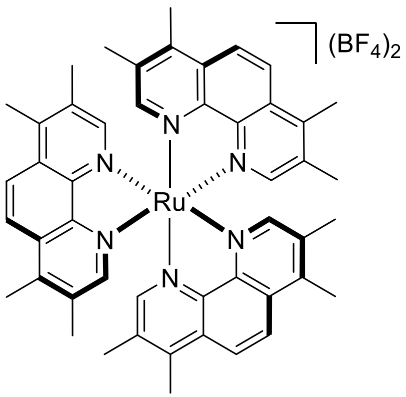

39

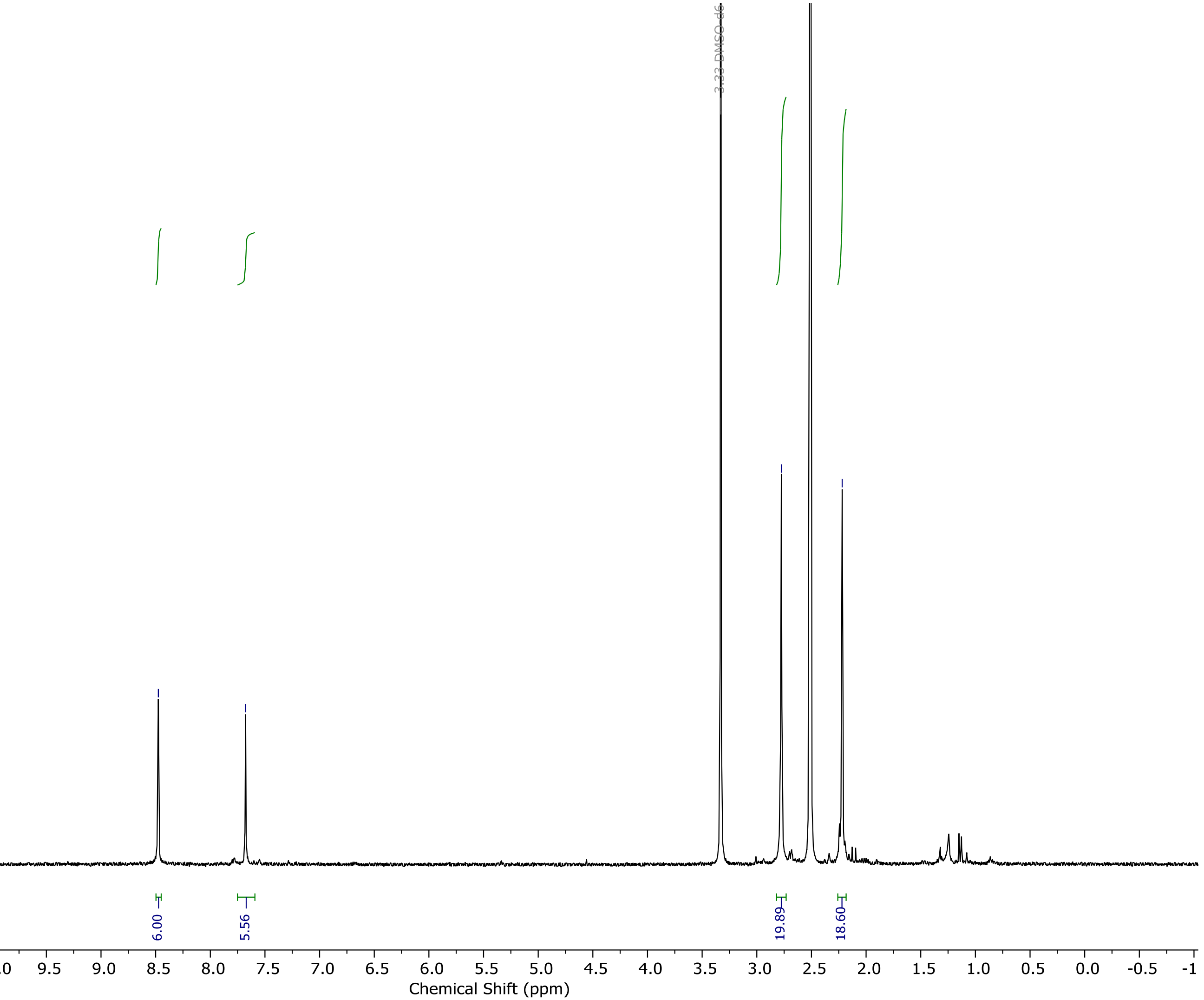

^{13}C NMR (101 MHz, DMSO) of Tris(3,4,7,8-tetramethyl phenanthroline)ruthenium(II) tetrafluoroborate 39

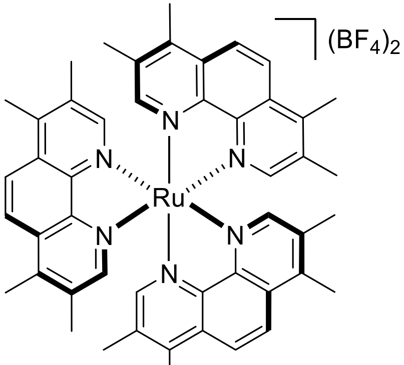

39

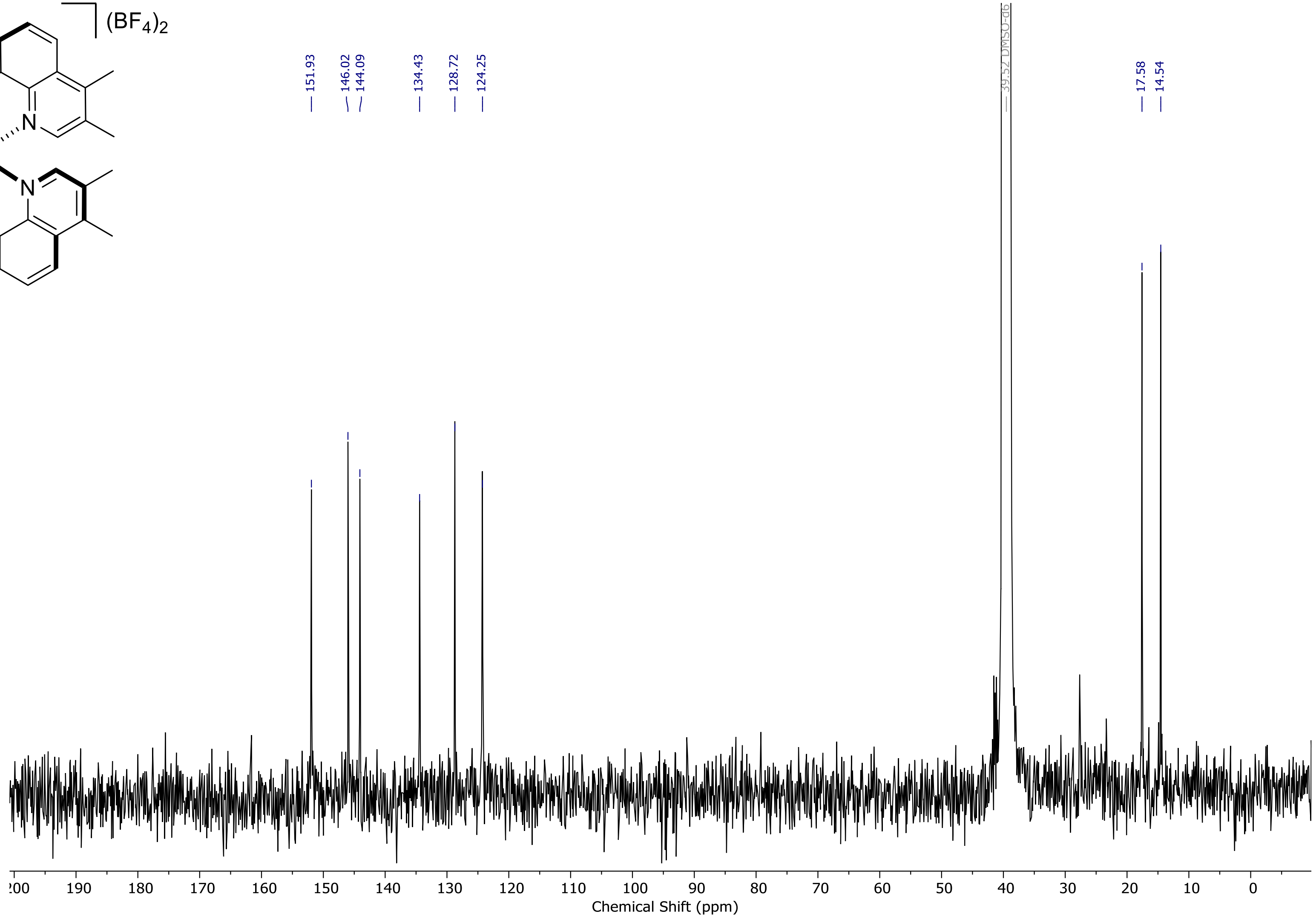

^{1}H NMR (400 MHz, DMSO) of Tris(bathophenanthroline)ruthenium(II) tetrafluoroborate 40

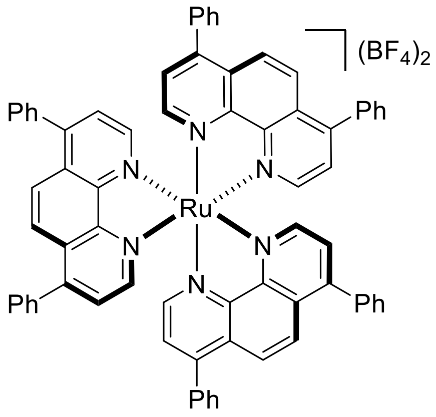

40

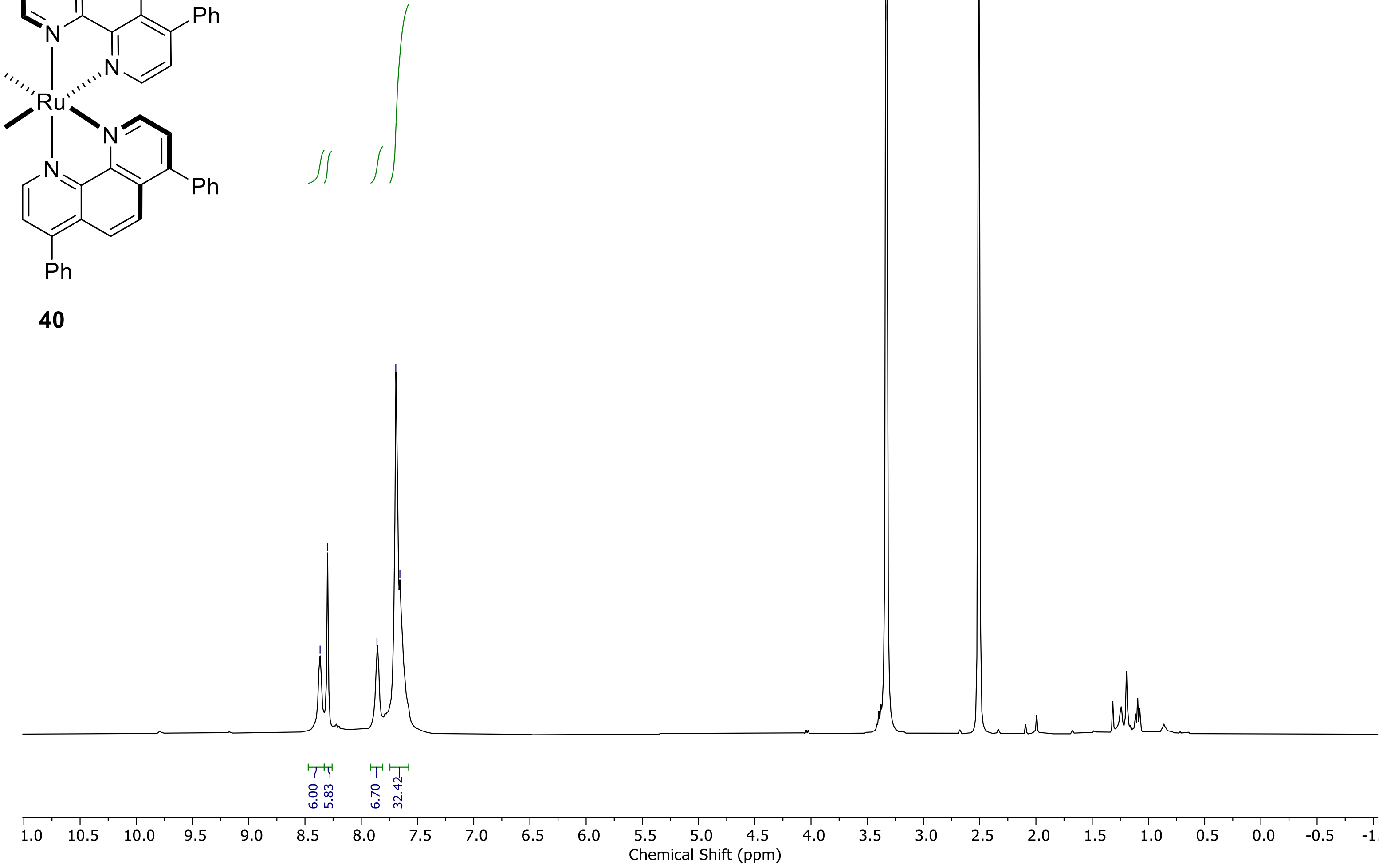

^{13}C NMR (101 MHz, DMSO) of Tris(bathophenanthroline)ruthenium(II) tetrafluoroborate 40

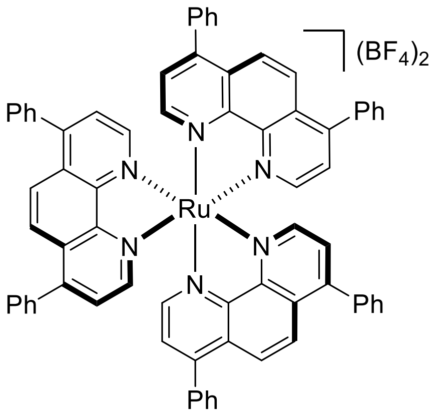

40

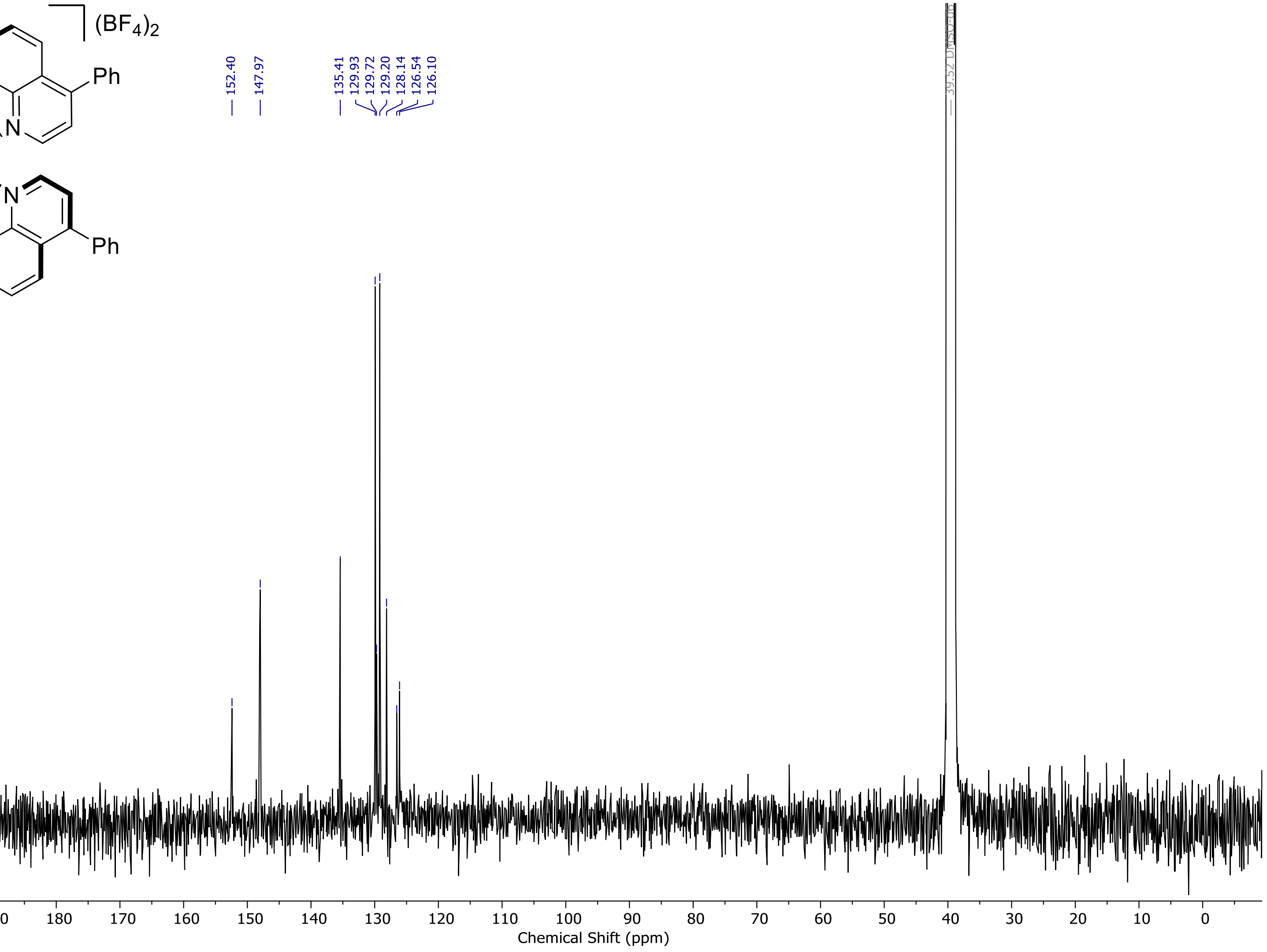

^{19}F NMR (471 MHz, d_4-MeOD) of Tris(bathophenanthroline)ruthenium(II) tetrafluoroborate 40

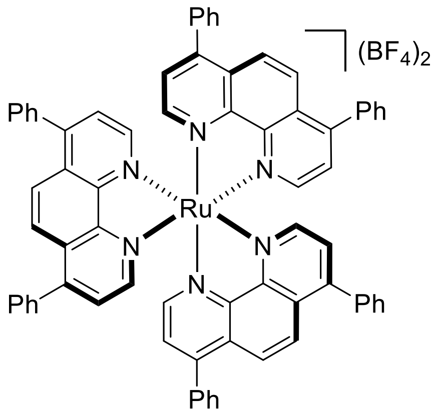

40

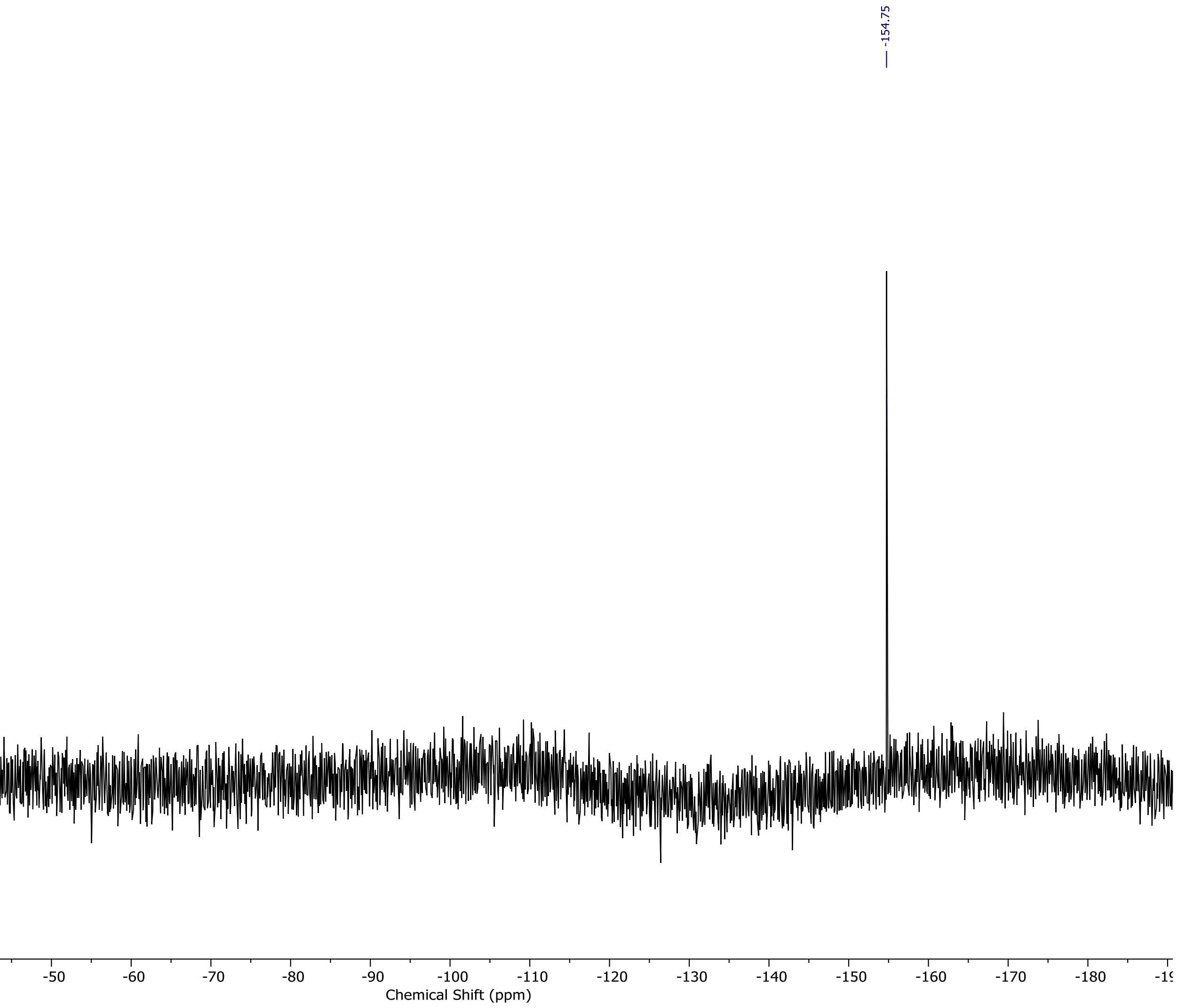

^{1}H NMR (400 MHz, $CDCl_3$) of octyl(phenyl)silane 43

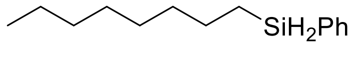

43

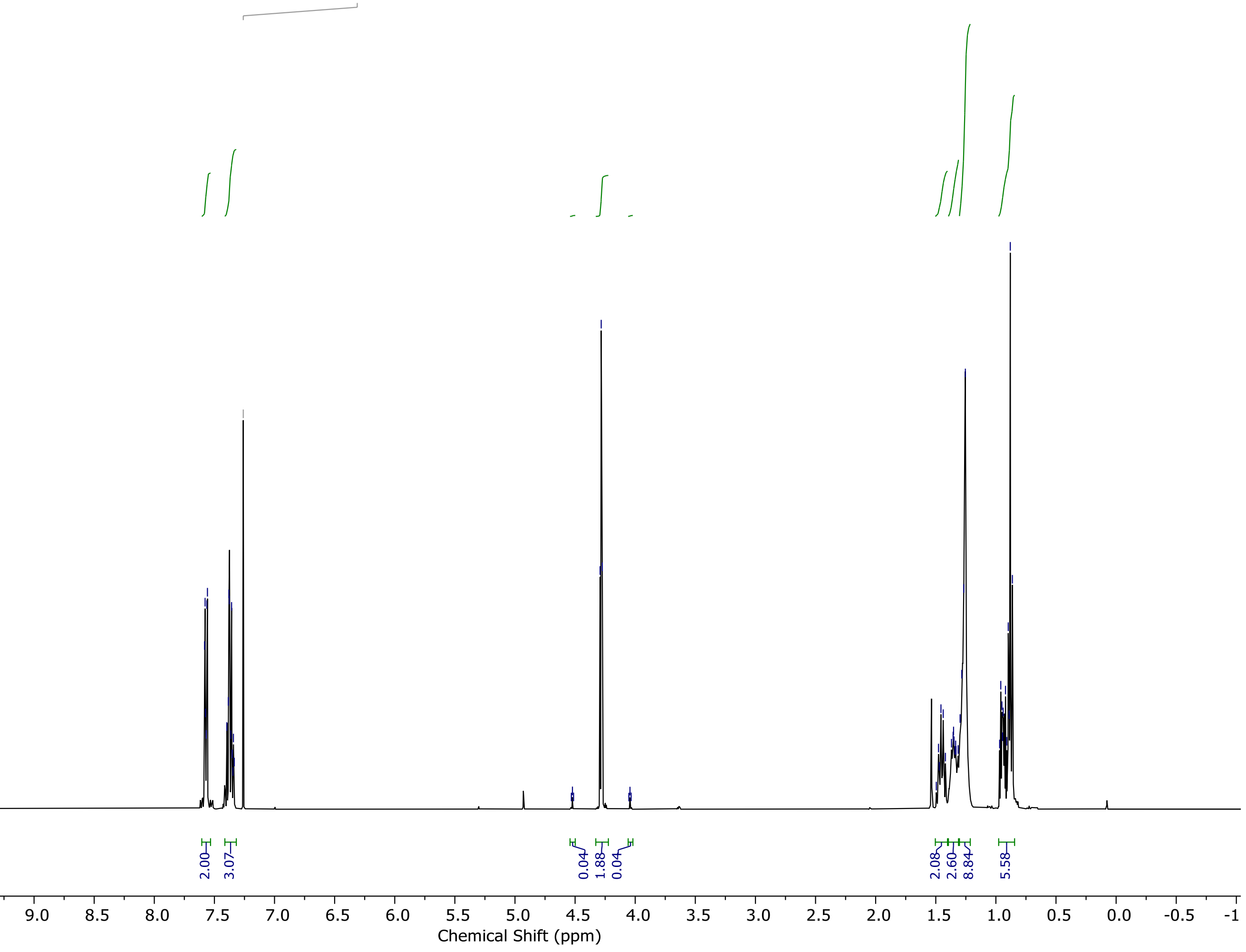

^{13}C NMR (101 MHz, $CDCl_3$) of octyl(phenyl)silane 43

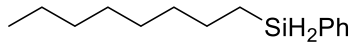

43

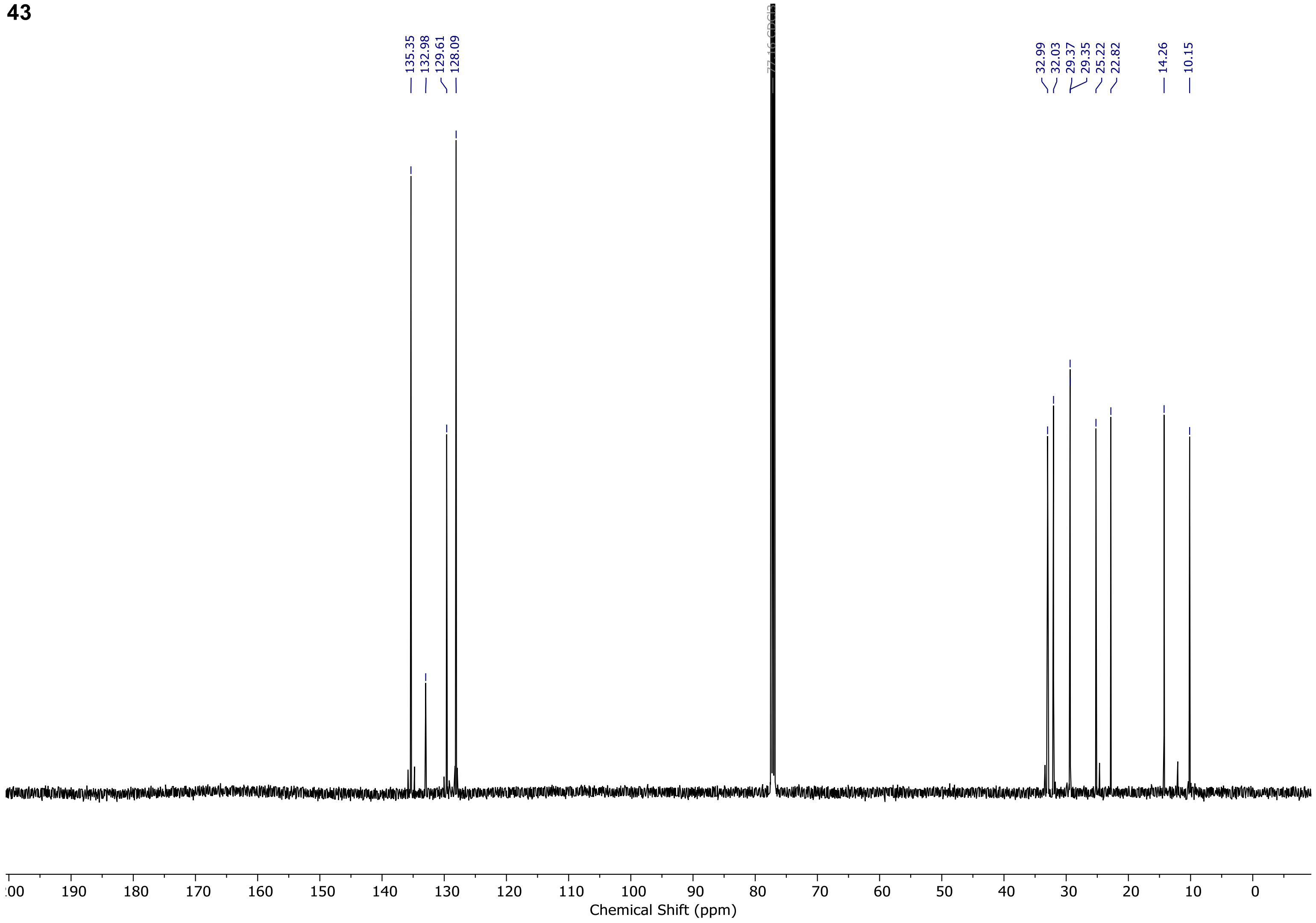

^{1}H NMR (500 MHz, d_6-acetone) of phenylmethanol 44 (from hydrosilylation)

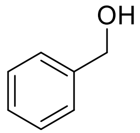

44

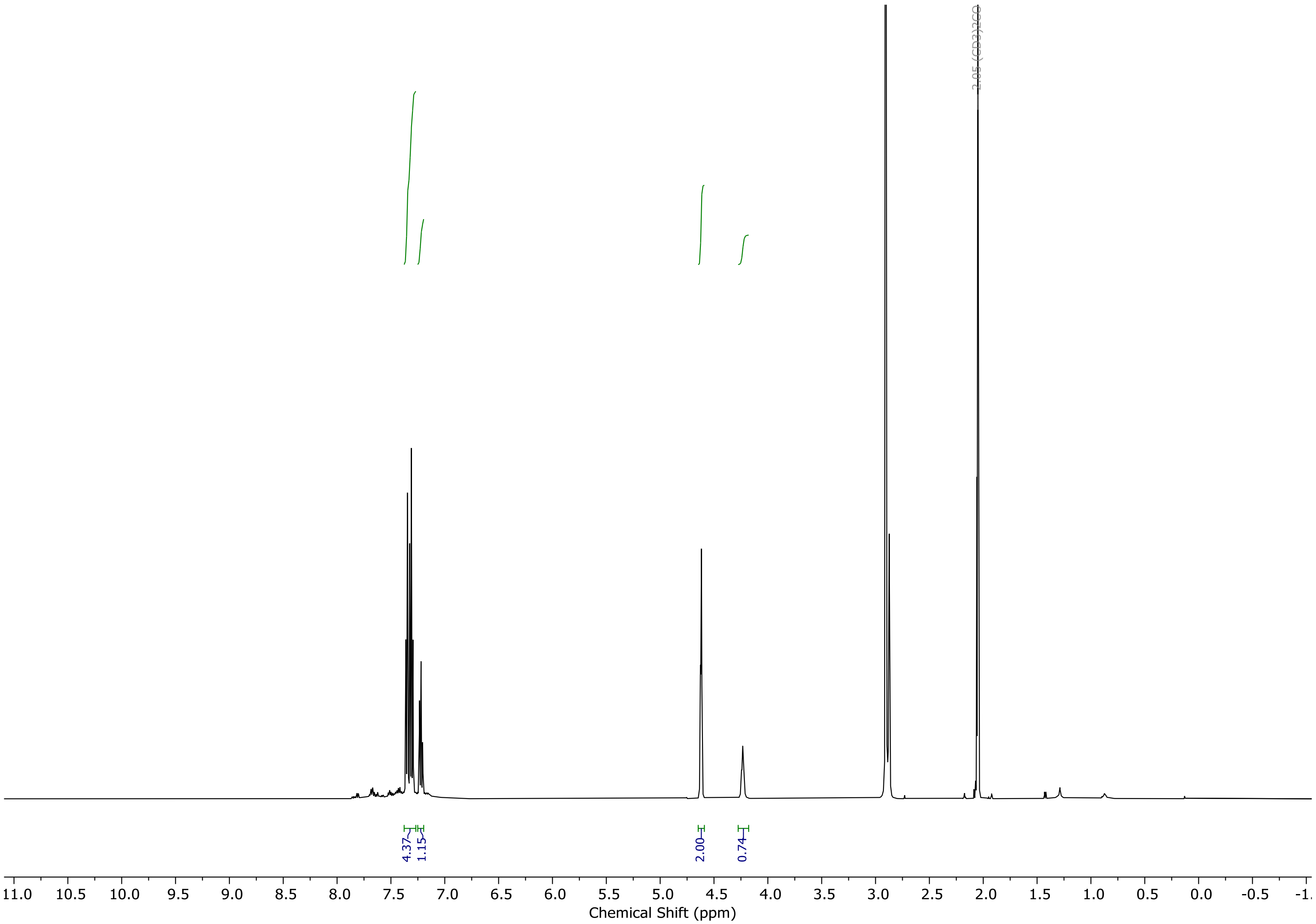

^{13}C NMR (101 MHz, CDCl3) of phenylmethanol 44 (from hydrosilylation)

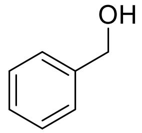

44

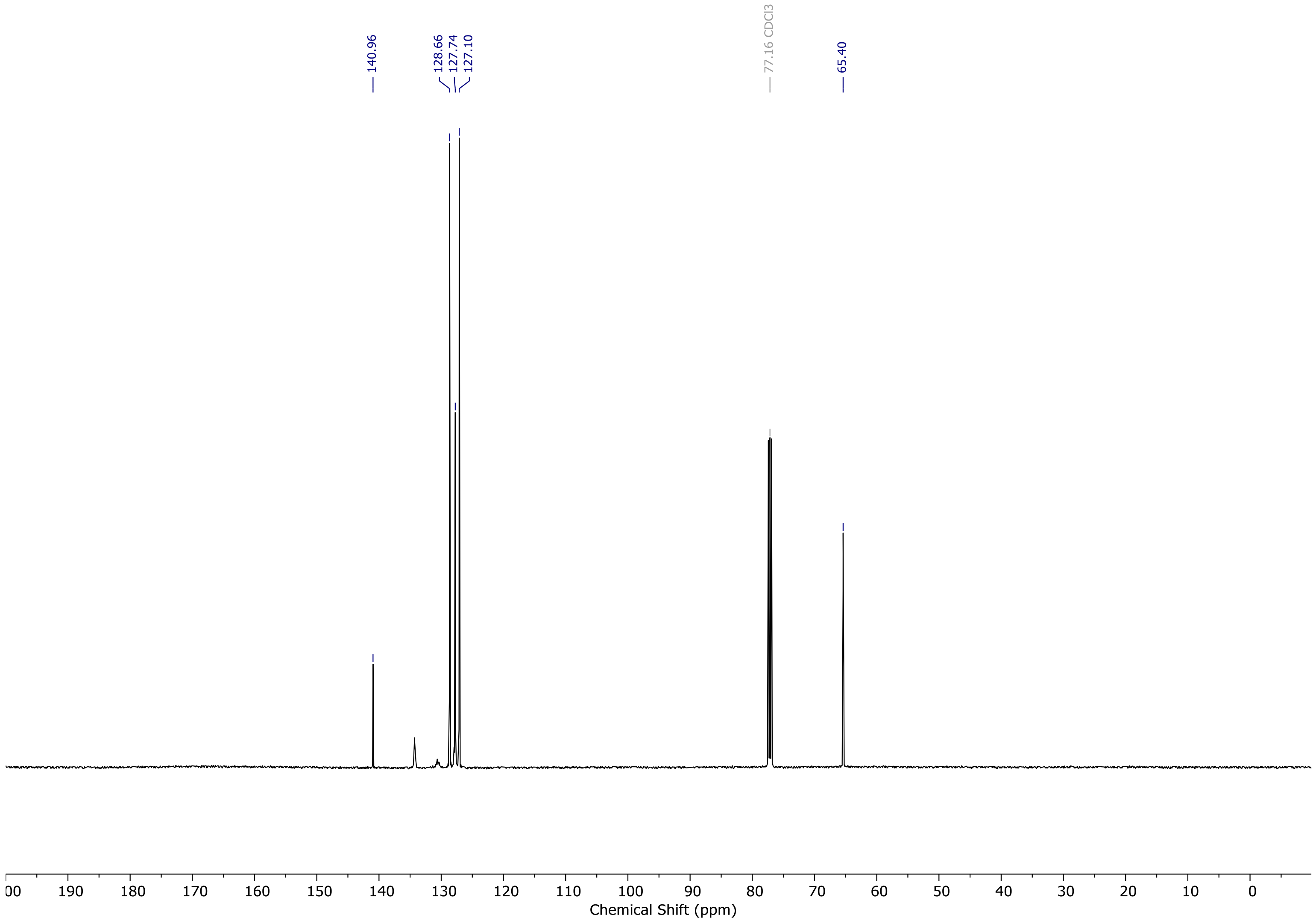

^{1}H NMR (500 MHz, $CDCl_3$) of 1-(4-(trifluoromethyl)phenyl)ethan-1-ol 34a (from hydrosilylation)

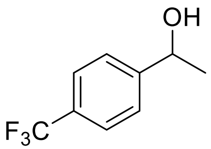

34a

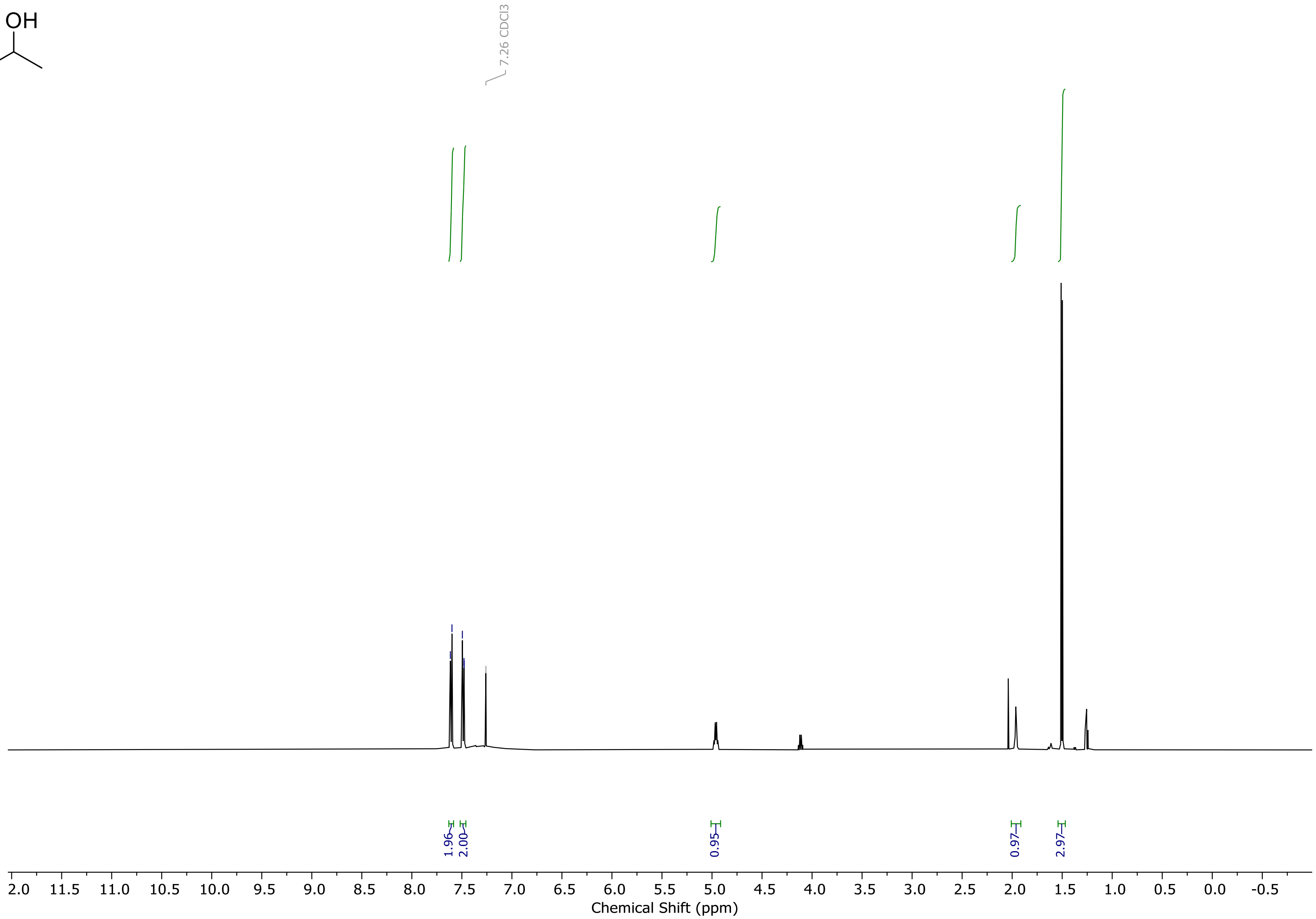

^{13}C NMR (101 MHz, $CDCl_3$) of 1-(4-(trifluoromethyl)phenyl)ethan-1-ol 34a (from hydrosilylation)

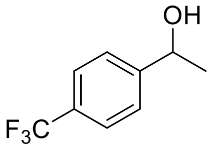

34a

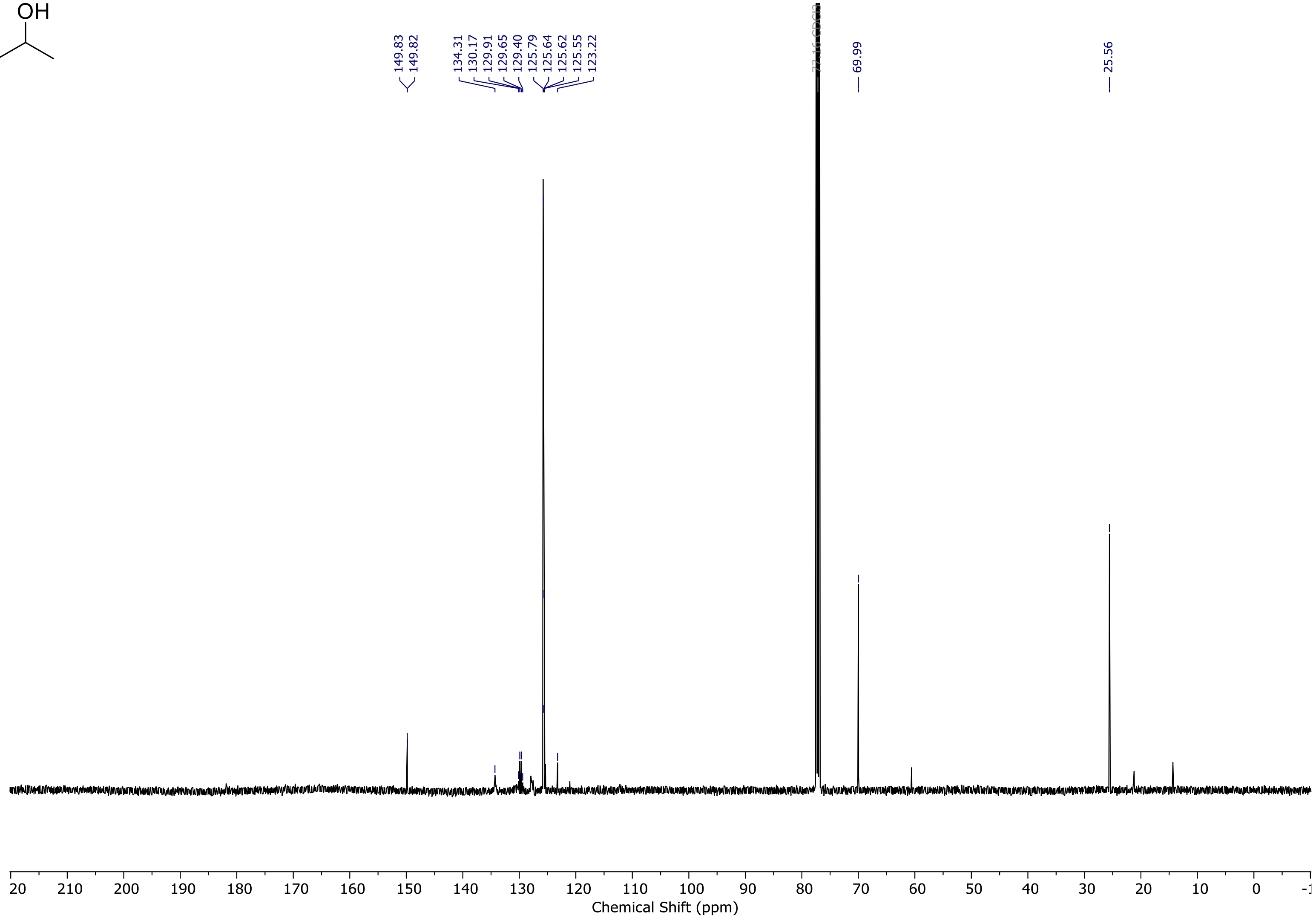

^{1}H NMR (400 MHz, $CDCl_3$) of 4,4,5,5-tetramethyl-2-(2-phenylpropyl)-1,3,2-dioxaborolane 45

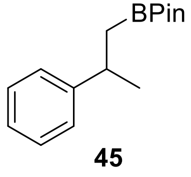

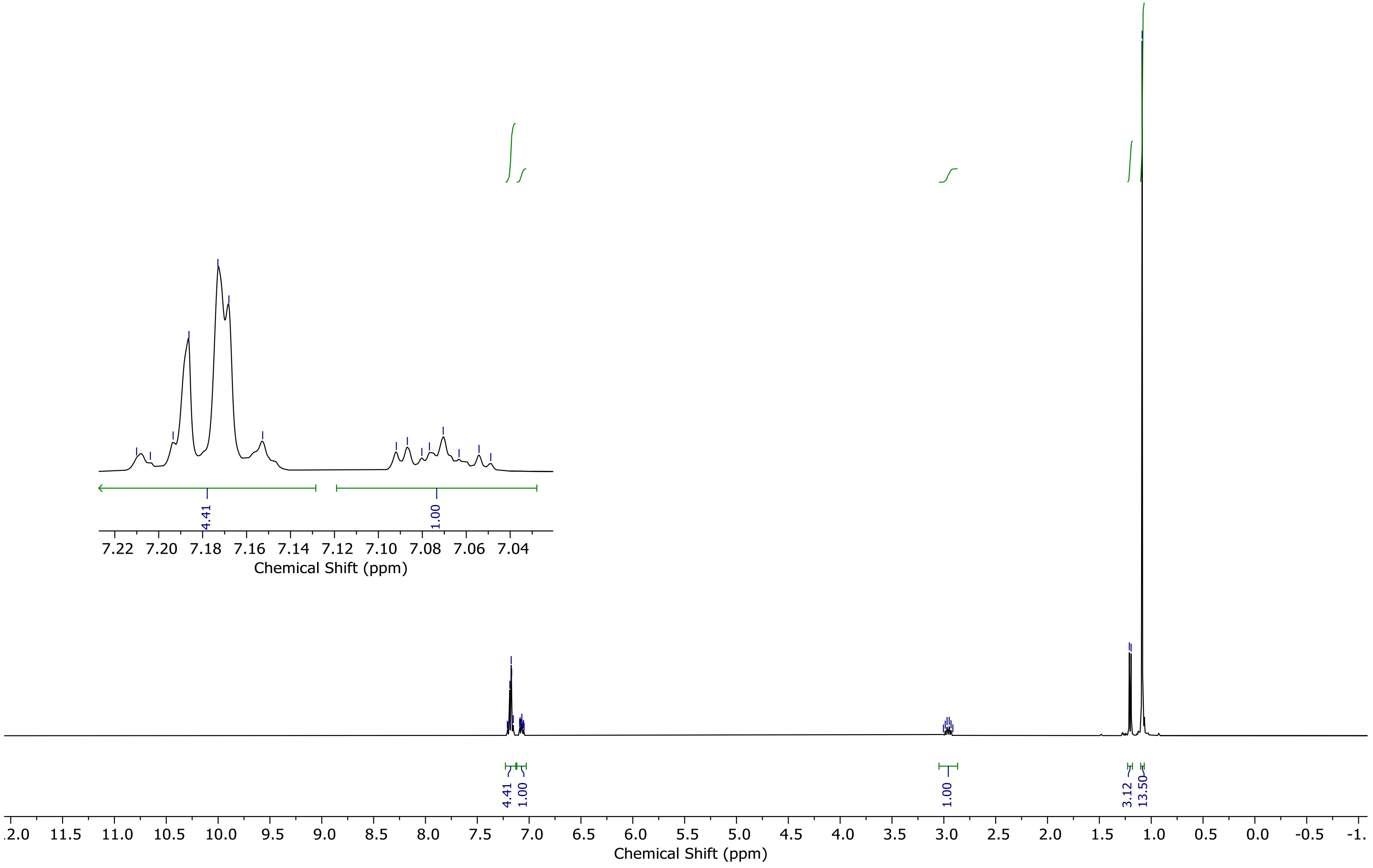

^{11}B NMR (128 MHz, $CDCl_3$) of 4,4,5,5-tetramethyl-2-(2-phenylpropyl)-1,3,2-dioxaborolane 45

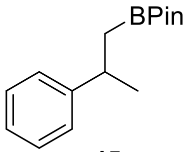

45

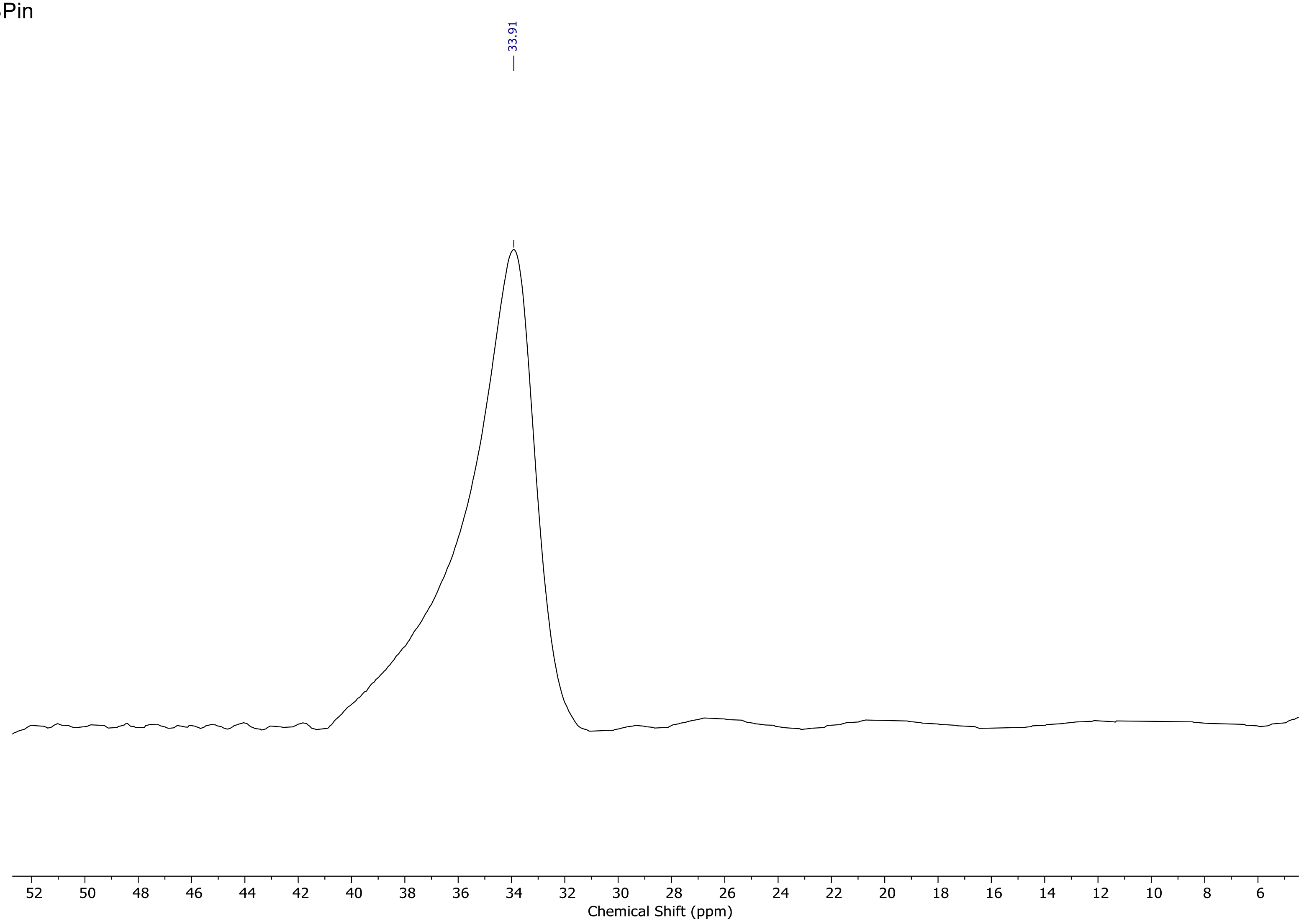

^{13}C NMR (101 MHz, $CDCl_3$) of 4,4,5,5-tetramethyl-2-(2-phenylpropyl)-1,3,2-dioxaborolane 45

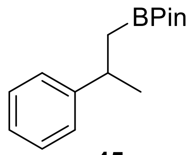

45

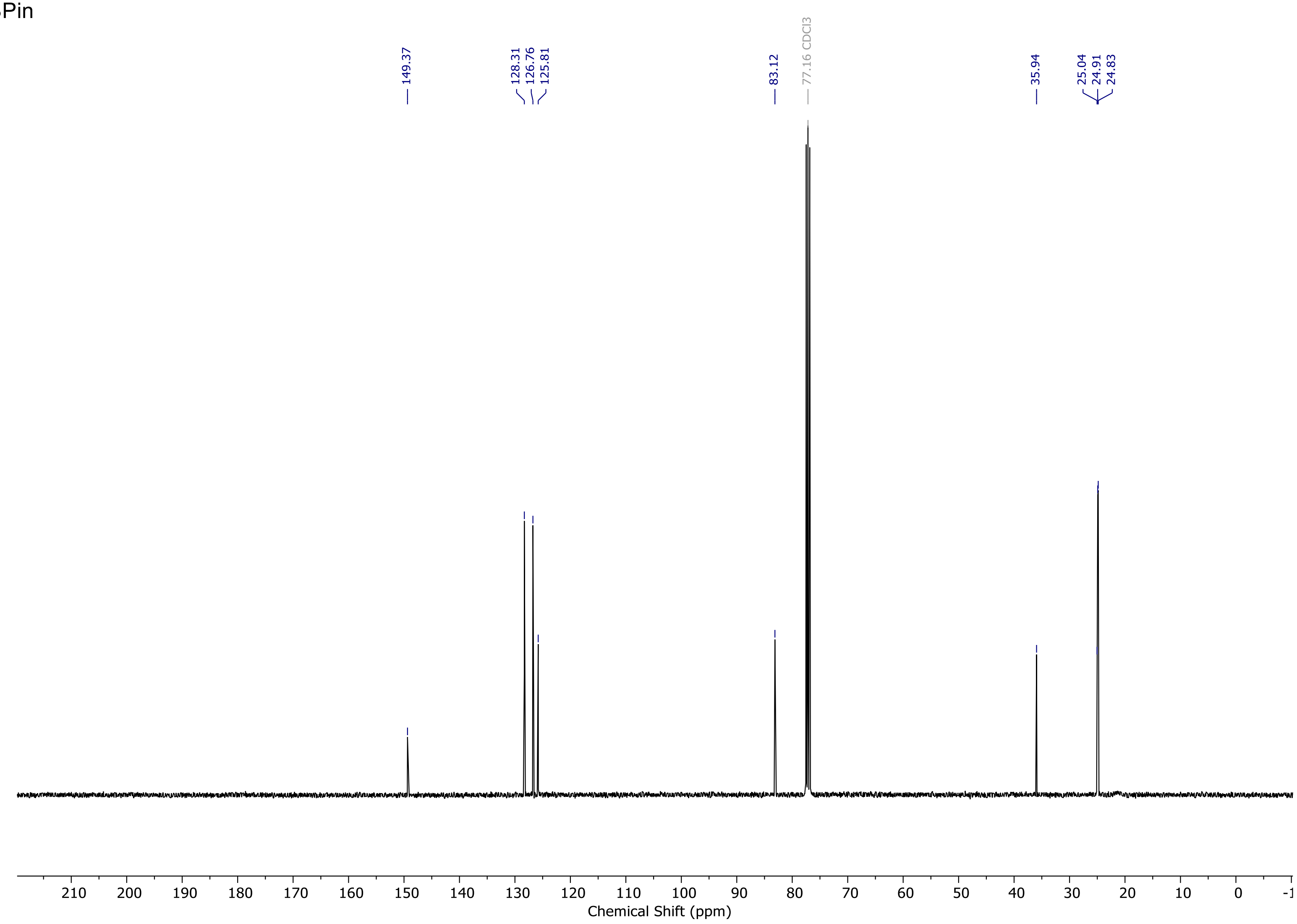

^{1}H NMR (400 MHz, $CDCl_3$) of 1,2,3,4-tetrahydroquinoline 46

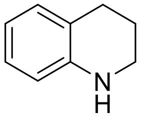

46

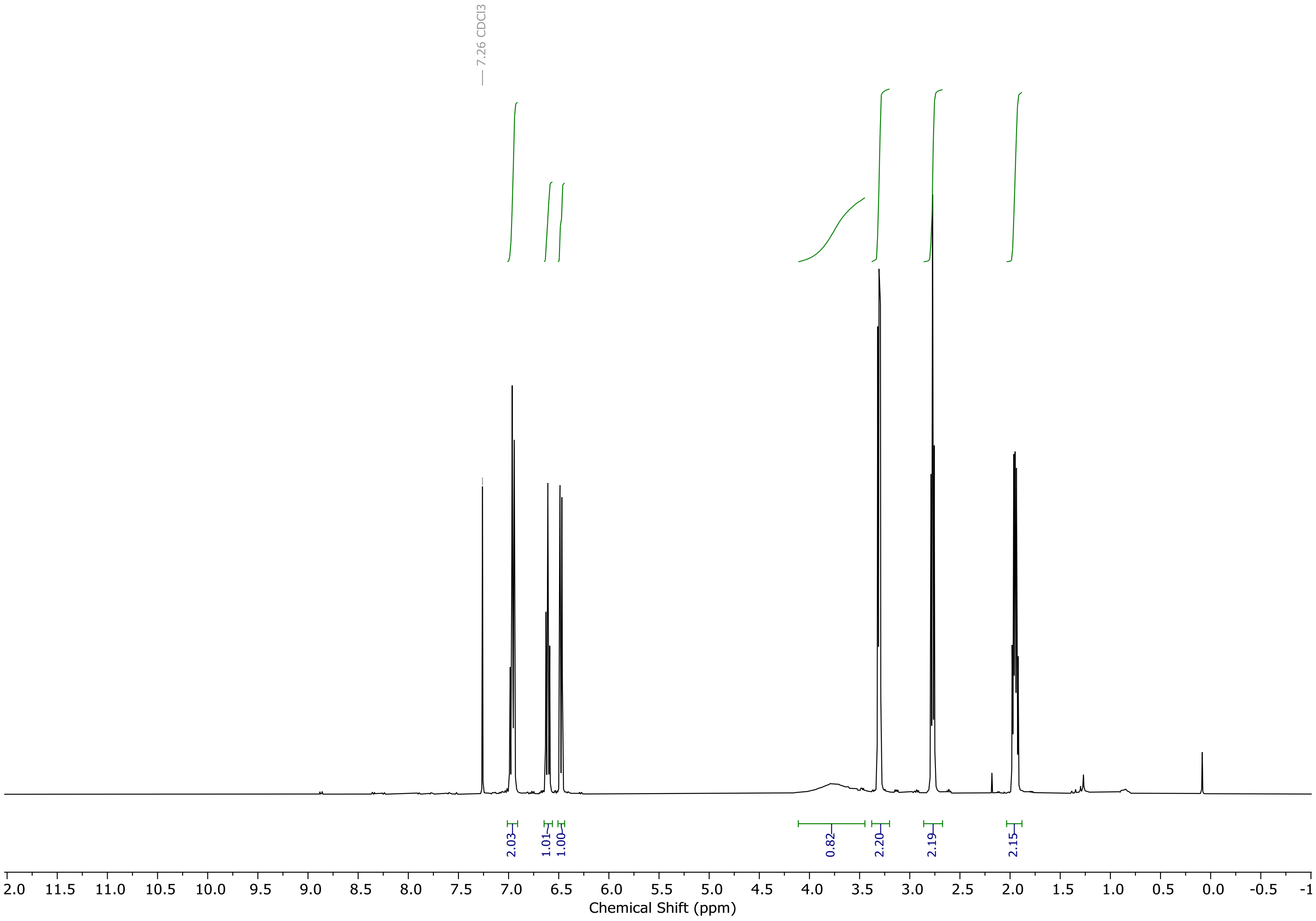

^{13}C NMR (101 MHz, $CDCl_3$) of 1,2,3,4-tetrahydroquinoline 46

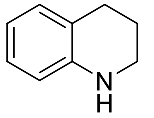

46

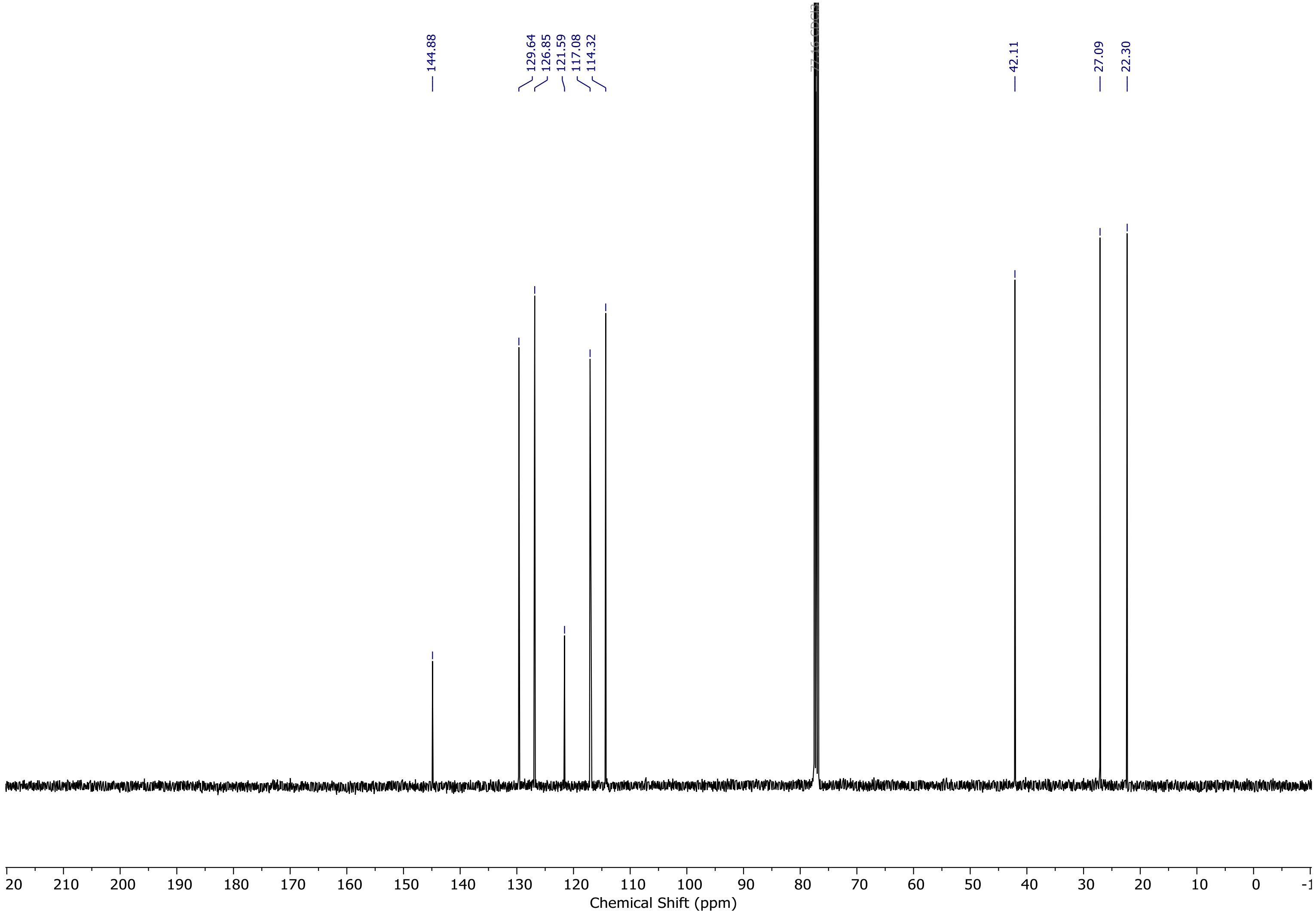

^{1}H NMR (500 MHz, $CDCl_3$) of phenylmethanol 44 (from hydroboration)

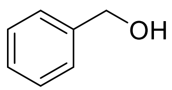

44

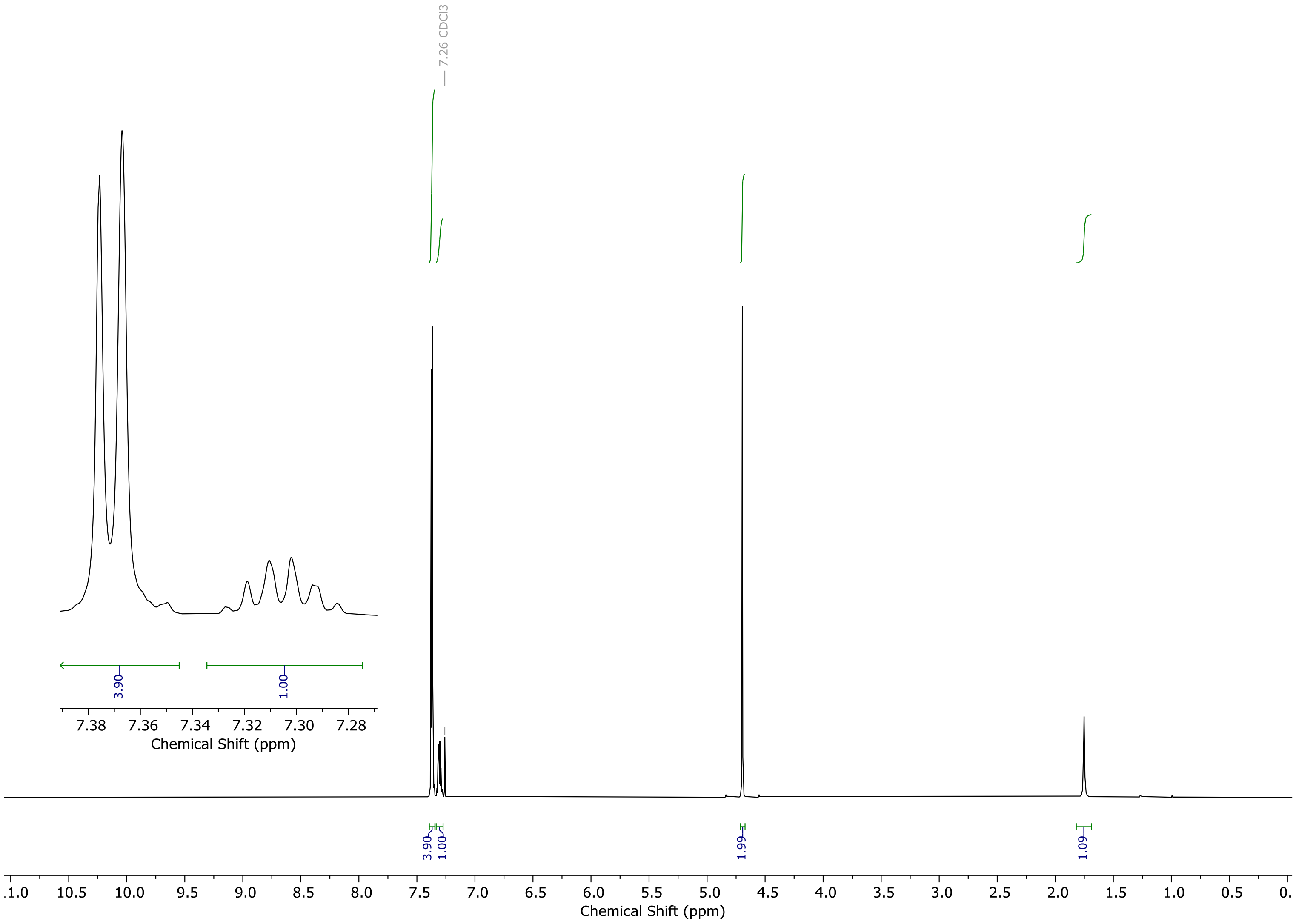

^{13}C NMR (126 MHz, $CDCl_3$) of phenylmethanol 44 (from hydroboration)

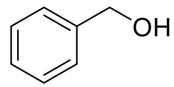

44

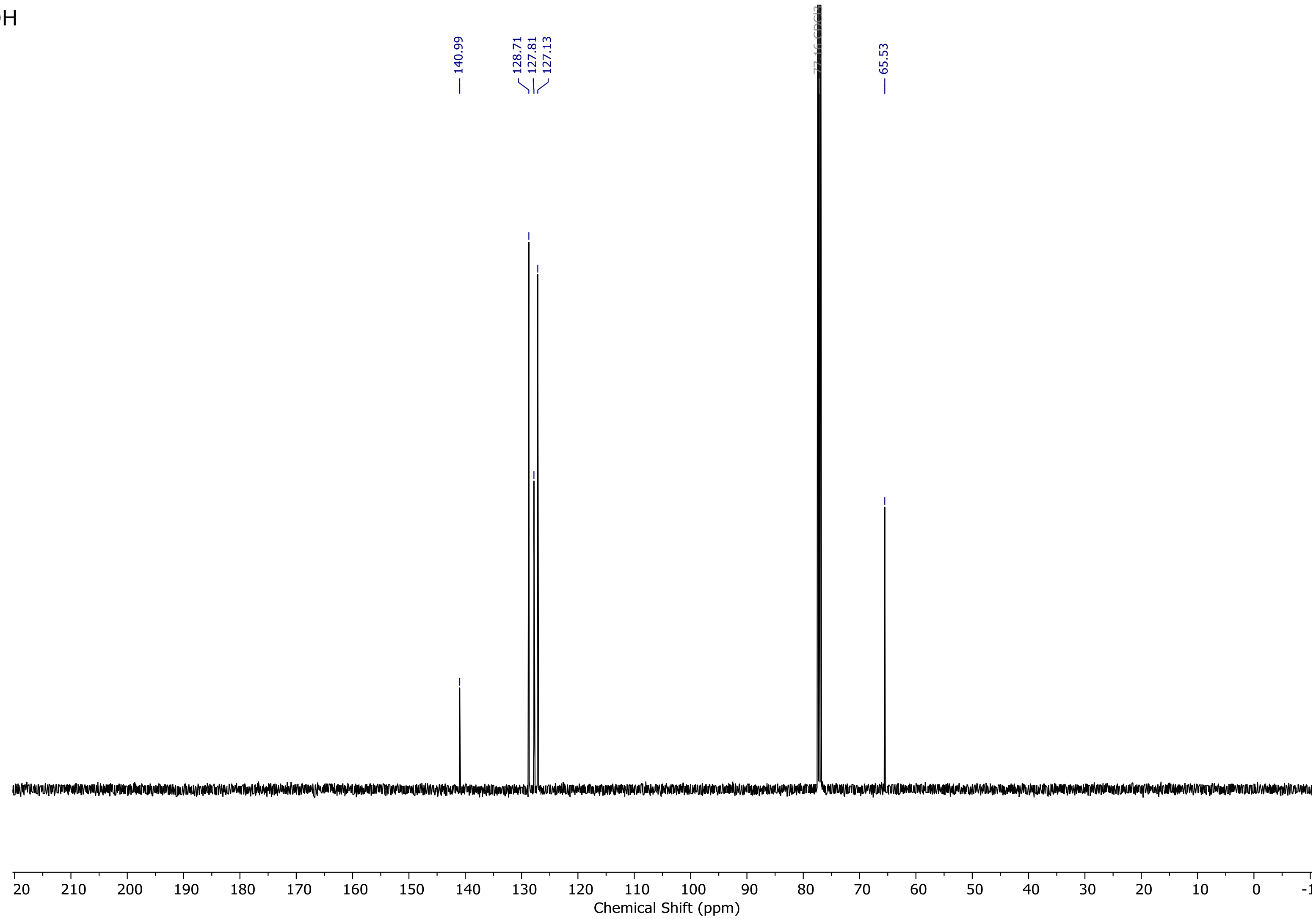

Crystallographic section.

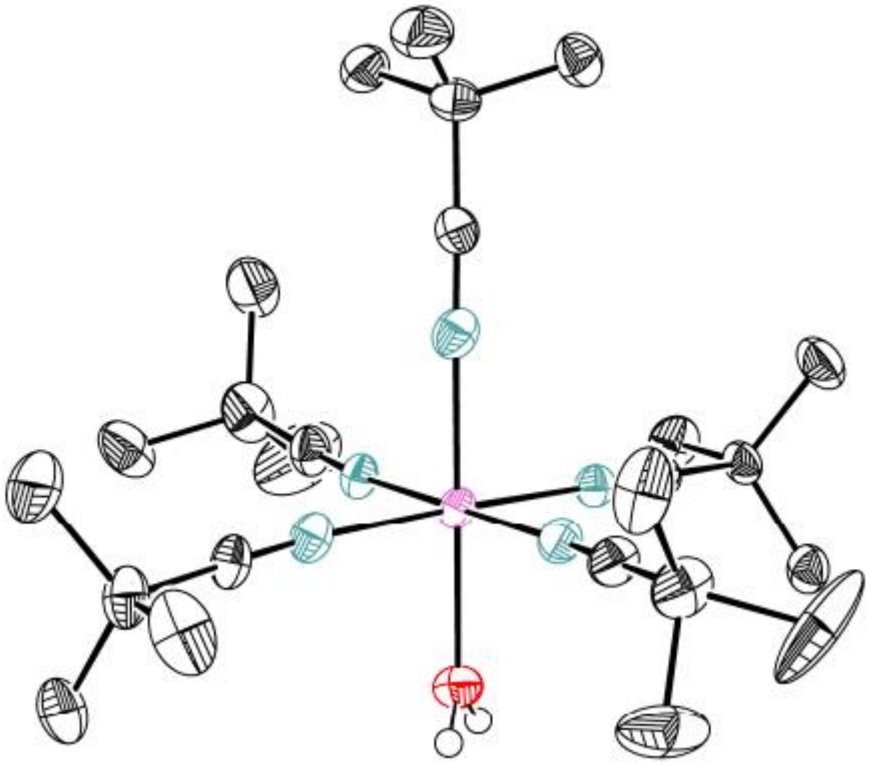

Data collection: X-ray diffraction data were collected for compounds **RuAqua** on a dual source Rigaku FR-X rotating anode at 100 K with Cu-Kα (1.54184 Å) radiation, equipped with a Hypix6000HE detector and Oxford cryosystem. X-ray data were collected using CrysAlisPro software.

Crystal structure determination and refinements: X-ray data were processed and reduced using CrysAlisPro. Absorption correction was performed using empirical methods (SCALE3 ABSPACK) based upon symmetry-equivalent reflections combined with measurements at different azimuthal angles. The crystal structure was solved and refined against all F^2 values using the SHELX and Olex2 suite of programmes.[1,2] All atoms were refined anisotropically. Hydrogen atoms were placed in calculated positions and refined using idealised geometries and assigned fixed isotropic displacement parameters. BF_4 anions, CH_2Cl_2 solvent molecules and some tBuCN ligands were disordered and modelled over two positions. The B-F, C-Cl and C-C bond distances were restrained using same distance SHELX restraints (SADI). Atomic displacement parameters were also restrained using rigid body and similar SHELX restraints (RIGU and SIMU).

Crystallographic data have been deposited with the CCDC 2264775.

Table 6. Crystallographic parameters for **RuAqua**

Identification code	**RuAqua**
Empirical formula	$C_{27}H_{51}B_2Cl_4F_8N_5ORu$
Formula weight	878.21
Temperature/K	100.01(10)
Crystal system	monoclinic
Space group	$P2_1/n$
a/Å	15.83631(16)
b/Å	25.2115(3)
c/Å	21.9795(2)
α/°	90
β/°	100.6654(10)
γ/°	90
Volume/Å^3	8623.88(16)
Z	8
ρ_{calc}g/cm^3	1.353
μ/mm^{-1}	5.766
F(000)	3600.0
Crystal size/mm^3	0.08 × 0.06 × 0.05
Radiation	Cu Kα (λ = 1.54184)
2Θ range for data collection/°	5.388 to 152.638
Index ranges	-18 ≤ h ≤ 19, -30 ≤ k ≤ 31, -27 ≤ l ≤ 27
Reflections collected	58681
Independent reflections	17604 [R_{int} = 0.0412, R_{sigma} = 0.0468]
Data/restraints/parameters	17604/991/1255
Goodness-of-fit on F^2	1.065
Final R indexes [I>=2σ (I)]	R_1 = 0.0506, wR_2 = 0.1465
tFinal R indexes [all data]	R_1 = 0.0653, wR_2 = 0.1605
Largest diff. peak/hole / e Å^{-3}	1.42/-1.57

References

1. Tran, V. T., Li, Z.-Q., Apolinar, O., Derosa, J., Joannou, M. V., Wisniewski, S. R., Eastgate, M. D., & Engle, K. M. Ni(COD)(DQ): An Air-Stable 18-Electron Nickel(0)–Olefin Precatalyst. *Angew. Chemie. Int. Ed.* **59**, 7409–7413 (2020).

2. Simonetti, M., Cannas, D. M., Just-Baringo, X., Vitorica-Yrezabal, I. J., & Larrosa, I. Cyclometallated ruthenium catalyst enables late-stage directed arylation of pharmaceuticals. *Nature Chem.* **10**, 724–731 (2018).

3. Zhong, L., Zong, Z.-H. & Wang, Z.-C. N-Heterocyclic Carbene Enabled Rhodium-Catalysed ortho C(sp^2)-H Borylation at Room Temperature. *Tetrahedron* **75**, 2547–2552 (2019).

4. Dan, W.-J., Tuong, T.-M.-L., Wang, D.-C., Li, D., Zhang, A.-L., & Gao, J.-M. Natural products as sources of new fungicides (V): Design and synthesis of acetophenone derivatives against phytopathogenic fungi in vitro and in vivo. *Bioorganic & Medicinal Chemistry Letters* **28**, 2861–2864 (2018).

5. Hogg, A., Wheatley, M., Domingo-Legarda, P., Carral-Menoyo, A., Cottam, N., & Larrosa, I. Ruthenium-Catalysed Monoselective C–H Methylation and d_3-Methylation of Arenes. *JACS Au* **2**, 2529–2538 (2022).

6. Ackermann, L., Vicente, R. & Althammer, A. Assisted Ruthenium-Catalyzed C−H Bond Activation: Carboxylic Acids as Cocatalysts for Generally Applicable Direct Arylations in Apolar Solvents. *Org. Lett.,* **10**, 2299–2302 (2008).

7. Woo, H., Cho, S., Han, Y., Chae, W.-S., Ahn, D.-R., You, Y., and Nam, W. Synthetic Control Over Photoinduced Electron Transfer in Phosphorescence Zinc Sensors. *J. Am. Chem. Soc.* **135**, 4771−4787 (2013).

8. Yang, Q.-L., Li, C.-Z., Zhang, L.-W., Li, Y.-Y., Tong, X., Wu, X.-Y., and Mei, T.-S. Palladium Catalysed Electrochemical C–H Alkylation of Arenes. *Organometallics* **38**, 1208−1212 (2019).

9. Yoshii, R., Hirose, A., Tanaka, K., and Chujo, Y. Boron Diiminate with Aggregation-Induced Emission and Crystallization-Induced Emission-Enhancement Characteristics. *Chem. Eur. J.* **20**, 8320–8324 (2014).

10. Zeng, X., Batsanov, A.S., and Bryce, M.R. Calix[6]arene Derivatives Selectively Functionalized at Alternate Sites on the Smaller Rim with 2-Phenylpyridine and 2-Fluorenylpyridine Substituents to Provide Deep Cavities. *J. Org. Chem.* **71**, 9589–9594 (2006).

11. Gorecka, E., Vaupotič, N., Zep, A., Pociecha, D., Yoshioka, J., Yamamoto, J., Takezoe, H. A Twist-Bend Nematic (NTB) Phase of Chiral Materials. *Angew. Chemie. Int. Ed.* **54**, 10155–10159 (2015).

12. Wheatley, M., Findlay, M. T., López-Rodríguez, R., Cannas, D. M., Simonetti, M., & Larrosa, I. RuAqua Catalysed room-temperature alkylation and late-stage alkylation of arenes with primary alkyl bromides. *Chem. Catalysis* **1**, 691–703 (2021).

13. Mizuno, H., Takaya, J., Iwasawa, N. Rhodium(I)-Catalysed Direct Carboxylation of Arenes with CO_2 via Chelation-Assisted C−H Bond Activation. *J. Am. Chem. Soc.* **133**, 1251–1253 (2011).

14. Wang, G.-W., Wheatley, M., Simonetti, M., Cannas, D. M., & Larrosa, I. Cyclometalated Ruthenium Catalyst Enables Ortho-Selective C–H Alkylation with Secondary Alkyl Bromides. *Chem* **6**, 1459–1468 (2020).

15. Sargent, B. T. & Alexanian, E. J. Palladium-Catalysed Alkoxycarbonylation of Unactivated Secondary Alkyl Bromides at Low Pressure. *J. Am. Chem. Soc.* **138***,* 7520–7523 (2016).

16. Zhou, Q., Hong, X., Cui, H.-Z., Huang, S., Yi, Y., & Hou, X.-F. The Construction of C–N, C–O, and C(Sp^2)–C(Sp^3) Bonds from Fluorine-Substituted 2-Aryl Benzazoles for Direct Synthesis of N-, O-, C-Functionalized 2-Aryl Benzazole Derivatives. *J. Org. Chem.* **83**, 6363–6372 (2018).

17. Zhang, Y., Zhao, Y., Luo, Y., Xiao, L., Huang, Y., Li, X., Peng, Q., Liu, Y., Yang, B., Zhu, C., Zhou, X., Zhang, J. Directed Aromatic C–H Activation/Acetoxylation Catalysed by Pd Nanoparticles Supported on Graphene Oxide. *Org. Lett.* **19**, 6470–6473 (2017).

18. Gandeepan, P., Koeller, J., Korvorapun, K., Mohr, J., & Ackermann, L. Visible-Light-Enabled Ruthenium-Catalysed meta-C−H Alkylation at Room Temperature. *Angew. Chem. Int. Ed.* **58**, 9820–9825 (2019).

19. Connelly, R. L., Knowles, J. P., & Booker-Milburn, K. I. A Rapid Synthetic Approach to the ABCD Core of the Stemona Alkaloids. *Org. Lett.* **21**, 18–21 (2018).

20. Theodorou, A., & Kokotos, C. G. Green Organocatalytic Synthesis of Indolines and Pyrrolidines from Alkenes. *Adv. Synth. Catal*. **359**, 1577–1581 (2017).

21. Wei, W., Yu, H., Zangarelli, A., & Ackermann, L. Deaminative meta-C–H alkylation by ruthenium(ii) catalysis *Chem. Sci.* **12**, 8073–8078 (2021).

22. Gou, X.-Y., Li, Y., Luan, Y.-Y., Shi, W.-Y., Wang, C.-T., An, Y., Zhang, B.-S., & Liang, Y.-M. Ruthenium-Catalysed Radical Cyclization/meta-Selective C–H Alkylation of Arenes via σ-Activation Strategy. *ACS Catal.* **11**, 4263–4270 (2021).

23. Piola, L., Fernández-Salas, J. A., Manzini, S., & Nolan, S. P. Regioselective ruthenium catalysed H–D exchange using D_2O as the deuterium source. *Org. Biomol. Chem.* **12**, 8683–8688 (2014).

24. Atzrodt, J., Derdau, V., Kerr, W. J., Reid, M., Rojahn, P., & Weck, R. Expanded applicability of iridium(I) NHC/phosphine catalysts in hydrogen isotope exchange processes with pharmaceutically-relevant heterocycles. *Tetrahedron*, **71**, 1924–1929 (2015).

25. Sanz-Navarro, S., Mon, M., Doménech-Carbó, A., Greco, R., Sánchez-Quesada, J., Espinós-Ferri, E., Leyva-Pérez, A. Parts–per–million of ruthenium catalyze the selective chain–walking reaction of terminal alkenes. *Nat. Commun.* **13**, 2831–2839 (2022).

26. Sun, P., Yan, H., Lu, L., Liu, D., Rong, G. & Mao, J. Ligand-accelerating low-loading copper-catalyzed effective synthesis of (E)-1,3-enynes by coupling between vinyl halides and alkynes performed in water. *Tetrahedron*, **69**, 6969–6974 (2013).

27. Mack, J., Gipson, J. D., Du Bois, J. & Sigman, M. S. Ruthenium-Catalyzed C–H Hydroxylation in Aqueous Acid Enables Selective Functionalization of Amine Derivatives. *J. Am. Chem. Soc.*, **139**, 9503–9506 (2017).

28. Shuler, W. G., Johnson, S. L., & Hilinski, M. K. Organocatalytic, Dioxirane-Mediated C–H Hydroxylation under Mild Conditions Using Oxone. *Org. Lett.* **19**, 4790–4793 (2017).

29. Zhou, Z., Chen, S., Hong, Y., Winterling, E., Tan, Y., Hemming, M., Harms, K., Houk, K. N., & Meggers, E. Non-C2-Symmetric Chiral-at-Ruthenium Catalyst for Highly Efficient Enantioselective Intramolecular C(sp^3)–H Amidation. *J. Am. Chem. Soc.* **141**, 19048–19057 (2019).

30. Shing, T. K. M., Tai, V. W.-F. & Tam, E. K. W. Practical and Rapid Vicinal Hydroxylation of Alkenes by Catalytic Ruthenium Tetraoxide. *Angew. Chem. Int. Ed.* **33**, 2312–2313 (1994).

31. Lie, S.-T., Reddy, K. V., Lai, R.-Y. Oxidative cleavage of alkenes catalysed by a water/organic soluble manganese porphyrin complex. *Tetrahedron*, **63**, 1821–1825 (2007).

32. Moorthy, J. N., Singhala, N., & Senapati, K. Oxidative cleavage of vicinal diols: IBX can do what Dess–Martin periodinane (DMP) can. *Org. Biomol. Chem.* **5**, 767–771 (2007).

33. Ruffoni, A., Hampton, C., Simonetti, M., & Leonori, D. Photoexcited nitroarenes for the oxidative cleavage of alkenes. *Nature*, **610**, 81–86 (2022).

34. Dolui, P., Verma, A., Saini, P., Nair, A., Thomas, S. P., & Elias, A. J. Synthesis, Characterization, and Catalysis of Water-Soluble Trimeric and Monomeric Palladium Complexes of 8-Aminoquinolines. *Eur. J. Inorg. Chem.* **26**, e202200559 (2023).

35. Choi, G., Kim, H. E., Hwang, S., Jang, H., & Chung, W.-J. Phosphorus(III)-Mediated, Tandem Deoxygenative Geminal Chlorofluorination of 1,2-Diketones. *Org. Lett.* **22**, 4190–4195 (2020).

36. Bubert, C. Blacker, J., Brown, S. M. Crosby, J., Fitzjohn, S., Muxworthy, J. P., Thorpe, T. & Williams, J. M. J. Synthesis of water-soluble aminosulfonamide ligands and their application in enantioselective transfer hydrogenation. *Tetrahedron Letters*, **42**, 4037–4039 (2001).

37. Wang, R., Han, X., Xu, J., Liu, P., & Li, F. Transfer Hydrogenation of Ketones and Imines with Methanol under Base-Free Conditions Catalysed by an Anionic Metal–Ligand Bifunctional Iridium Catalyst. *J. Org. Chem.* **85**, 2242–2249 (2020).

38. Hackl, L., Ho, L. P., Bockhardt, D., Bannenberg, T., & Tamm, M. Tetraaminocyclopentadienone Iron Complexes as Hydrogenation Catalysts. *Organometallics* **41**, 836–851 (2022).

39. Ramachandran, P. V., Alawaed, A. A., & Hamann, H. J. $TiCl_4$-Catalysed Hydroboration of Ketones with Ammonia Borane. *J. Org. Chem.* **87**, 13259–13269 (2022).

40. Liu, X., Wang, Q., Han, C., Feng, X., Du, H. Chiral Frustrated Lewis Pairs Catalysed Highly Enantioselective Hydrosilylations of Ketones. *Chin. J. Chem*. **37**, 663–666 (2019).

41. Zhong, R., Wei, Z., Zhang, W., Liu, S., & Liu, Q. A Practical and Stereoselective In Situ NHC-Cobalt Catalytic System for Hydrogenation of Ketones and Aldehydes. *Chem* **5**, 1552–1566 (2019).

42. van der Westhuizen, D., von Eschwege, K. G., & Conradie, J. Electrochemistry and spectroscopy of substituted $[Ru(phen)_3]^{2+}$ and $[Ru(bpy)_3]^{2+}$ complexes. *Electrochimica Acta* **320**, 134540–134549 (2019).

43. Hu, L., Li, K., Shang, W., Zhu, X. & Liu, M. Emerging Cubic Chirality in γCD-MOF for Fabricating Circularly Polarized Luminescent Crystalline Materials and the Size Effect. *Angew. Chem. Int. Ed.* **59**, 4953–4958 (2020).

44. Wu, X., Ding, G., Lu, W., Yang, L., Wang, J., Zhang, Y., Xie, X., & Zhang, Z. Nickel-Catalysed Hydrosilylation of Terminal Alkenes with Primary Silanes via Electrophilic Silicon–Hydrogen Bond Activation. *Org. Lett.* **23**, 1434–1439 (2021).

45. Zhang, G., Scott, B. L., Hanson, S. K. Mild and Homogeneous Cobalt-Catalysed Hydrogenation of C=C, C=O, and C=N Bonds. *Angew. Chem. Int. Ed.* **51**, 12102–12106 (2012).

46. Liu, Y., Zhou, Y., Wang, H., & Qu, J. $FeCl_2$-catalysed hydroboration of aryl alkenes with bis(pinacolato)diboron. *RSC Adv.* **5**, 73705–73713 (2015).

47. Chakraborty, S., Brennessel, W. W., & Jones, W. D. A Molecular Iron Catalyst for the Acceptorless Dehydrogenation and Hydrogenation of N-Heterocycles. *J. Am. Chem. Soc.* **136**, 8564–8567 (2014).

48. Constantin, T., Zanini, M., Regni, A., Sheikh, N. S., Juliá, F., & Leonori, D. Aminoalkyl radicals as halogen-atom transfer agents for activation of alkyl and aryl halides. *Science*, **367**, 1021–1026 (2020).
